# Analysis of essential gene dynamics under antibiotic stress in *Streptococcus sanguinis*

**DOI:** 10.1099/mic.0.000595

**Published:** 2018-01-09

**Authors:** Fadi El-Rami, Xiangzhen Kong, Hardik Parikh, Bin Zhu, Victoria Stone, Todd Kitten, Ping Xu

**Affiliations:** ^1^​ Philips Institute for Oral Health Research, Virginia Commonwealth University, Richmond, VA, USA; ^2^​ Department of Microbiology and Immunology, Virginia Commonwealth University, Richmond, VA, USA

**Keywords:** essential genes, antibiotic stress, *Streptococcus sanguinis*, transcriptomics, proteomics

## Abstract

The paradoxical response of *Streptococcus sanguinis* to drugs prescribed for dental and clinical practices has complicated treatment guidelines and raised the need for further investigation. We conducted a high throughput study on concomitant transcriptome and proteome dynamics in a time course to assess *S. sanguinis* behaviour under a sub-inhibitory concentration of ampicillin. Temporal changes at the transcriptome and proteome level were monitored to cover essential genes and proteins over a physiological map of intricate pathways. Our findings revealed that translation was the functional category in *S. sanguinis* that was most enriched in essential proteins. Moreover, essential proteins in this category demonstrated the greatest conservation across 2774 bacterial proteomes, in comparison to other essential functional categories like cell wall biosynthesis and energy production. In comparison to non-essential proteins, essential proteins were less likely to contain ‘degradation-prone’ amino acids at their N-terminal position, suggesting a longer half-life. Despite the ampicillin-induced stress, the transcriptional up-regulation of amino acid-tRNA synthetases and proteomic elevation of amino acid biosynthesis enzymes favoured the enriched components of essential proteins revealing ‘proteomic signatures’ that can be used to bridge the genotype–phenotype gap of *S. sanguinis* under ampicillin stress. Furthermore, we identified a significant correlation between the levels of mRNA and protein for essential genes and detected essential protein-enriched pathways differentially regulated through a persistent stress response pattern at late time points. We propose that the current findings will help characterize a bacterial model to study the dynamics of essential genes and proteins under clinically relevant stress conditions.

## Introduction

*Streptococcus sanguinis* SK36 is a Gram-positive, facultative anaerobic bacterium that is described as a Janus-faced micro-organism. On one hand, it is an oral commensal that competes with pathogenic bacteria for colonization of the oral cavity [1] through the production of bactericidal hydrogen peroxide that has been shown to eliminate an etiologic agent of dental caries, namely *Streptococcus mutans* [2]. On the other hand, *S. sanguinis* has been related to the formation of biofilms in the oral cavity, also called dental plaque [3, 4], and has been defined as an opportunistic pathogen that is among the leading etiologic agents of infective endocarditis in patients with heart valve defects [5, 6] and bacteremia in neutropenic patients [7]. Understanding bacterial behaviour during disease necessitates in-depth analysis of transcriptomic and proteomic profiles under clinically relevant conditions. After sequencing the genome [8] and identifying the essential genes that are indispensable for survival of *S. sanguinis* SK36 *in vitro* (brain–heart infusion (BHI) media) [9], the current challenge is to identify the dynamics of its underlying cellular components, such as mRNA and proteins, especially the essential ones, in clinically relevant conditions, to define ‘pathogenesis signatures’ as promising therapeutic targets.

By colonizing the oral cavity in abundance, *S. sanguinis* are exposed to antibiotics which persist at sub-inhibitory concentrations for long periods of time, either directly through antibiotic ingestion by patients or indirectly through anthropogenic antibiotic usage and consumption of antibiotics through animal food products [10, 11]. It was estimated that out of the total antibiotic prescriptions for clinical purposes, 7 and 10 % of total prescriptions are provided by dentists in the UK [12] and Spain [13], respectively. Ampicillin and amoxicillin are β-lactam antibiotics that differ only in one hydroxyl group but share the same spectrum of activity against Gram-positive bacteria, despite different intestinal absorption rates [14]. They are considered the drugs of choice for many dental practices [15] and are prophylactic drugs for infective endocarditis-susceptible patients [16, 17]. Misuse and abuse of antibiotics by many dentists worldwide exacerbate the situation by further exposing the oral microbiota, including *S. sanguinis*, to these drugs [18]. It was shown that almost half of the prescribed antibiotics are excreted in an active form, which raises questions about the diluted residual doses and their impact on bacterial communities in the host [19].

Since 1946, early observations noted the paradoxical behaviour of *S. sanguinis* in response to drugs prescribed prophylactically against infective endocarditis *in vitro*, where they were shown to be susceptible, versus *in vivo*, where they demonstrated resistance against the same drugs [20]. Many hypotheses were formulated to explain this observation. One interpretation attributed this antibacterial resistance pattern *in vivo* to the physical barriers that block the access of antibiotics to the bacteria, such as aggregated platelets and fibrin on damaged heart valves and biofilm structures [21]. However, this concept was challenged by findings that antibiotics can in fact successfully diffuse through biofilms, weakening the barrier argument of ‘protected niches’ [22]. Another hypothesis to explain the *in vivo* antibiotic resistance pattern was first described as persistence [23], where bacteria modulate their metabolism and growth rate to cope with the environmental stress, such as antibiotics, thus becoming tolerant to antibiotics [24, 25]. After clearance of antibiotics, persistent bacteria relapse albeit with an antibiotic susceptible profile [26]. This scenario is consistent with findings for *S. sanguinis*, where cases of β-lactam resistance have been rare [27, 28]. Surprisingly, no homologues for the *Escherichia coli* persistence elements were identified in the *S. sanguinis* proteome, such as the initiation toxin–antitoxin pair *hipA–hipB* [29], toxin–antitoxin systems *YafQ/DinJ* and *MqsR–MqsA* [30], the persister formation peptide *tisB* [31], bringing us back to the question: how does *S. sanguinis* respond to sub-inhibitory concentrations of antibiotics? Put differently, how does *S. sanguinis* modulate its genetic regulatory network and pathways to thrive in a stressful environment which happens to be the norm rather than the exception?

Essential genes present a promising potential for addressing a plethora of biological questions. They are the keys for essential functions and have survived the evolutionary purifying selection by evolving at a slower rate to sustain the cell’s survival [32, 33]. Their evolutionary robustness is due to their engagement in multiple functional pathways, in addition to their evolved capacity to re-wire genetic and protein networks to compensate for any emerging stress [34]. In this sense, environmental specificity (stress) provides the dominant explanation for existence of an essential gene set. After defining the essential gene set of *S. sanguinis*, the current challenge is understanding the coping mechanism of this bacterium with antibiotic stress by highlighting the behaviour of essential genes in response to this specific environmental assault, especially when coupled with high-throughput approaches with global coverage of essential genes and proteins.

Despite the tremendous advances in sequencing technologies and the consequent dissection of complete genomes, our understanding of complex molecular interactions driving physiological mechanisms within a bacterial cell under the effect of antibiotics is fragmentary [35]. The main aim of the current project is to investigate the transcriptomic and proteomic profiles of essential genes and proteins, using RNA-sequencing (RNA-seq) and mass spectrometric analysis respectively, under treatment of a sub-inhibitory concentration of a commonly prescribed antibiotic in dental practices, ampicillin, to elucidate the *S. sanguinis* stress response mechanisms on a temporal basis and define ‘pathogenesis signatures’ as potential therapeutic targets. By simultaneously studying the transcript and protein levels of all essential genes and half of the essential proteins under stress, our study will help characterize a bacterial model to better understand the dynamics of essential genes under clinically relevant stress factors and to assist in designing evidence-based guidelines for drug prescription in clinical practice.

## Methods

### Bacterial strains, media and growth conditions

*S. sanguinis* strain SK36 was routinely grown in BHI broth (BD, San Jose, CA, USA) under micro-aerobic conditions (7.2 % H_2_, 7.2 % CO_2_, 79.6 % N_2_ and 6 % O_2_) at 37 °C as previously described [36]. For stress response studies, three replicates of bacterial samples were exposed to the MIC of ampicillin (0.25 µg ml^−1^), MICx0.5 and MICx0.25 doses at the mid-exponential growth phase. The MIC value was reported in the Clinical and Laboratory Standards Institute document M100-S25 [37]. To collect enough cells for extraction of mRNA or protein, we added ampicillin at an OD_600_ value of 0.6. The two lower concentrations of ampicillin resulted in slight growth defects in comparison to the untreated samples (Fig. S1, available in the online version of this article). It was decided that 0.125 µg ml^−1^ was the best dose as it was the lowest dose that significantly impacted *S. sanguinis* growth and it was selected to treat *S. sanguinis* cells at the mid-exponential growth phase for 10, 20 or 30 min. A triplicate of bacterial samples was left untreated as a control.

### Reagents and buffers

All buffers and solutions were prepared using ultrapure water and analytical grade reagents. All prepared reagents were stored at room temperature unless indicated otherwise. Protease Inhibitor Cocktail Set II (Calbiochem, EMD Millipore, cat. no. 539132) was prepared as a stock solution by adding to each vial of lyophilized protease inhibitor cocktail 1 ml of DMSO followed by 4 ml of ultrapure water. The stock solution was stored at −20 °C. dl-Dithiothreitol (Sigma, cat. no. D9779 SIGMA) was prepared as a 1 M stock solution and stored at 4 °C. Incomplete lysis buffer was prepared as follows: 50 mM tris(hydroxymethyl)aminomethane (Tris) (pH 7.4), 150 mM NaCl, SDS 0.1 % (w/v). Immediately before use, 1 ml of complete lysis buffer for each sample was prepared by mixing 100 µl of reconstituted protease inhibitor solution, 1 µl of 1 M DTT (stock) and 900 µl incomplete lysis buffer. The complete lysis buffer was stored on ice.

### Data mining from databases

Pathways that contain essential genes were searched as described by the Kyoto Encyclopedia of Genes and Genomes (KEGG) database. The functional annotation tool of DAVID Bioinformatics Resources 6.7 [38] was used for functional enrichment analysis of our gene and protein dataset. To visualize the maximal number of essential genes on a single map that encompasses all pathways harbouring essential genes and integrate the gene expression profiles at different time points, a network was constructed based on data acquired from KEGG using the Cytoscape 3.4 software platform [39]. Physiochemical characteristics (molecular weight, amino acid length, instability index and hydropathy values) of *S. sanguinis* SK36 proteins were determined using Biopython scripts. Scatter plots for clustering the clusters of orthologous groups' (COG) annotations of essential and non-essential genes were designed using Prism 5 software. Circos plots were designed to visualize the differential regulation of essential genes and proteins in transcriptomic and proteomic data, respectively [40].

### Transcriptome analysis by RNA-seq

For RNA-seq, 12 replicates of *S. sanguinis* SK36 samples were cultured for 16 h in BHI broth at 37 °C in microaerophilic conditions. The next day, cells were diluted 100-fold into 4 ml BHI broth and grown in a 37 °C incubator for 4.5–5 h until OD_600_ readings of samples reached 0.6. Except for one triplicate of *S. sanguinis* SK36 samples that was saved as a control, all other samples were grouped into triplicates where each triplicate was treated with sub-inhibitory concentration of ampicillin (0.125 µg ml^−1^) for one time period (10, 20 or 30 min). RNAprotect Bacteria Reagent (cat. no. 76506, Qiagen, CA, USA) was added to each bacterial culture (nine ampicillin-treated samples and three untreated samples). Cells were incubated for 5 min at room temperature, centrifuged and the pellet stored at −80 °C. Cell lysates were collected using RNeasy mini kit (cat. no. 74106, Qiagen, CA, USA) and bead milling conducted with 2 ml Lysing matrix B beads in the Fast Prep 24 for 45 s at level 6. All samples were DNA-depleted using DNase I RNase-Free DNase Set (cat. no. 79254, Qiagen, CA, USA). Total RNA concentrations were measured using a NanoDrop 2000 UV-Vis Spectrophotometer (Thermo fisher, DE, USA) with accepted thresholds for absorbance ratios 260/280 and 260/230 of 2.0 and 2–2.2 respectively. For depletion of ribosomal RNA, all samples were treated with Illumina Ribo-zero Magnetic Kit for Bacteria (cat. no. MRZB12424, Roche, USA) and the rRNA-depleted samples were purified using Qiagen RNeasy MinElute Cleanup Kit (cat. no. 74204, Qiagen, CA, USA). RNA concentrations were measured in rRNA-depleted samples using NanoDrop 2000 UV-Vis Spectrophotometer with cutoff values for RNA concentration of 10 ng µl^−1^. Actinomycin D (cat. no. A1410-2MG, Sigma-Aldrich, MO, USA) was used for RNA fragmentation and RNA libraries were prepared with NEBNext Ultra Directional RNA Library Prep Kit NEB (cat. no. E7420L, New England Biolabs, MA, USA) and NEBNext Multiplex Oligos for Illumina Index Primers Set 1 and set 2 (cat. nos E7335L and E7500L respectively, New England Biolabs, MA, USA). The final cDNA products were purified with AMPure XP Beads (cat. no. A63880, Beckman Coulter, CA, USA) and band sizes were checked by gel electrophoresis. The quality of the constructed cDNA library was determined using Agilent Bioanalyzer-High Sensitivity DNA Chip and Ribosome Integrity Numbers (RIN) were determined for all samples with a cutoff value of 10. Library sequencing was performed by the Nucleic Acids Research Facilities at Virginia Commonwealth University using Illumina HiSeq2000. Reads obtained from sequencing were aligned against the *S. sanguinis* SK36 genome using Rockhopper v. 2.03 software [41] and counts of transcripts along with statistical calculations were provided. Transcriptome profiles were analysed for enriched pathways and functionally related genes using DAVID v. 6.8 Beta [42].

### Gene expression data

The RNA-seq data was deposited in the Gene Expression Omnibus database (www.ncbi.nlm.nih.gov/geo/) under the accession number GSE97218 for ampicillin-treated samples and untreated samples.

### Measurement of essential protein conservation patterns

A bioinformatics approach was developed to measure the conservation ratio of every experimentally detected essential protein in *S. sanguinis* SK36. In brief, we extracted the amino acid sequences of all proteins from 2774 bacterial species deposited in the National Center for Biotechnology Information (NCBI) database. We designed a program based on a reciprocal hit approach to detect orthologues of every *S. sanguinis* SK36 essential protein (as a query) against all bacterial proteins (as a subject), and vice versa, using Basic Local Alignment Search Tool (BlastP). We accepted an orthologue as a significant match when any sequence alignment had the following cutoff values: minimal sequence identity of 50 % and E value ≤1e^−5^.

### Examination of stressed growth *in vitro*

Overnight cultures of *S. sanguinis* SK36 were diluted 100-fold in BHI and grown for 4 h in microaerophilic conditions, and then diluted 20-fold into microplate wells containing fresh BHI and treated with a sub-inhibitory concentration of ampicillin at mid-log phase (OD_600_=0.6). Each sample was tested in triplicate. Growth rates were determined by measuring the OD_600_ using a Synergy H1 Hybrid Reactor microplate reader (BioTek, VT, USA) every 5 min under aerobic conditions for 12 h of untreated and ampicillin-treated triplicate samples. The experiment was performed in triplicate.

### Protein extraction and quantification

Protein samples were prepared from bacterial lysates as follows: overnight cultures of *S. sanguinis* SK36 were diluted 100-fold into 50 ml BHI for 5 h of growth under micro-aerobic conditions to achieve an OD_600_ reading of 0.6. A triplicate of bacterial samples was left untreated as a control, while other triplicates were treated with ampicillin for 10, 20 or 30 min (the same procedure as transcriptomic-profiled samples but different sample preparation). Cytoplasmic proteins were extracted as described previously [43]. All bacterial cells were centrifuged at 4 °C for 10 min at 2200 ***g***, washed twice with cold PBS, and mixed with lysis buffer (50 mM Tris-HCl, 150 mM NaCl, 1 % SDS, 1 mM dithiothreitol) supplemented with protease inhibitor cocktail (Sigma P8430). After 30 min on ice, the pellets were bead homogenized using a Fast Prep 24 for 40 s at level 4.5 twice. Soluble proteins were recovered from the supernatant after centrifugation at 4 °C for 15 min at 10 000 ***g***. Soluble proteins were quantitated using a Pierce BCA Protein Assay kit (cat, no. 23227, IL, USA).

### Sample preparation for quantitative mass spectrometry

Proteins were acetone-precipitated and incubated for 1 h at −20 °C. After centrifugation for 10 min at 13 000 ***g***, the protein pellet was re-suspended in 100 µl RapiGest SF working solution and vortexed thoroughly to dissolve the protein pellet. Samples were reduced with 4 µl of 10 mM dithiothreitol in 0.1 M ammonium bicarbonate at room temperature for 30 min, then the samples were alkylated with 4 µl 50 mM iodoacetamide in 0.1 M ammonium bicarbonate at room temperature for 30 min. Finally, samples were digested with 1 µg trypsin overnight and then quenched with 5 % (v/v) glacial acetic acid.

### Label-free protein analysis by mass spectrometry

Samples were analysed by a Waters Synapt G2Si mass spectrometer system with a nanospray ion source interfaced to a Waters M-Class C18 reversed-phase capillary column. MS^E^ scout runs were performed on each sample with spiked internal standards to determine the amount of protein on the column. The injection volume was adjusted to achieve 200 ng protein on the column for each analysis using ion mobility separation. Each sample was run in triplicate using this technique.

For proper spectral processing and database searching conditions, the peak list-generating software and search engine included at Progenesis QI for Proteomics software package v.2.0 (Non-Linear Dynamics, Liverpool, UK) were used. The UNIPROT protein databank with specific annotations for *S. sanguinis* SK36 was used, and the search conditions for the relative quantification of proteins were based on the following criteria: the maximum number of allowed missed cleavages by trypsin was set to 1; fixed modifications by carbamidomethyl (C), variable oxidation (M) were allowed. The refining of peptide identifications deleted all peptides with a score <5, mass <400 ppm and mass error <15 ppm, as calculated by the Progenesis QIP software. Statistical analyses were performed with the quantitative measurements of at least two peptides per protein, four fragments per peptide, ten fragments per protein, according to the standard Progenesis QIP processing capability. The mass spectrometry proteomics data have been deposited in the ProteomeXchange Consortium [44] via the PRIDE partner repository [45] with the dataset identifier PXD006479.

### Statistical analysis

Chi-square analysis was used for the measurement of statistical significance between the amino acid composition in essential and non-essential proteins. The sem was used for the depiction of proteins’ conservation within a functional category. This parameter defines the relationship between the dispersion of individual observations around the population mean (the sd) for a given sample size. For proteomic analysis, we used Progenesis QI software with advanced statistical tools, such as ANOVA, *P*-value cutoff of 0.05 and *Q*-value cutoff of 0.01 (for false discovery rate) for peptide identification and multivariate statistics for protein measurements. For transcriptomic analysis, we used the Rockhopper v. 2.03 software which is based on a negative binomial distribution as its statistical model with a *P*-value cutoff of 0.05 and *Q*-value cutoff of 0.01 (using the Benjamini–Hochberg procedure).

## Results

### Functional categorization and conservation of *S. sanguinis* SK36 essential proteins

Using a single gene knockout technique, we previously identified 218 essential genes in *S. sanguinis* cultured in BHI medium [44]. Based on GenBank COG functional categories, we found that *S. sanguinis* essential genes are unevenly distributed in functional categories (Table 1), biased towards translation (33.2 % of total essential genes), replication and repair (10.7 %), lipid metabolism (9 %) and cell wall/membrane/envelope biogenesis (7.6 %). If classified by a specific COG functional category, lipid metabolism (20 essential genes vs 20 non-essential genes) and translation (74 essential genes vs 79 non-essential genes) have nearly an equal number of essential and non-essential genes. With 203 (COG category: R) and 184 (COG category: S) non-essential genes defined as hypothetical genes, the functional category most enriched with non-essential genes is ‘General functional prediction only’ (11.7 % of non-essential genes).

**Table 1.** Functional categorization of essential and non-essential genes based on COG annotations

COG	Description	EG*	% Total genes	Non-EG*	% Total genes
C	Energy production and conversion	10	4.5	60	3.5
D	Cell cycle control and mitosis	7	3.1	16	0.9
E	Amino acid metabolism and transport	2	0.9	191	11.0
F	Nucleotide metabolism and transport	9	4.0	69	4.0
G	Carbohydrate metabolism and transport	13	5.8	156	9.0
H	Coenzyme metabolism	12	5.4	76	4.4
I	Lipid metabolism	20	9.0	20	1.1
J	Translation	74	33.2	79	4.6
K	Transcription	5	2.2	150	8.7
L	Replication and repair	24	10.7	87	5.0
M	Cell wall/membrane/envelop biogenesis	17	7.6	100	5.8
O	Post-translational modification, protein turnover, chaperone functions	6	2.7	56	3.2
P	Inorganic ion transport and metabolism	3	1.3	88	5.0
P	Inorganic ion transport and metabolism	3	1.3	88	5.0
Q	Secondary structure	3	1.3	23	1.3
R	General functional prediction only	11	4.9	203	11.7
T	Signal transduction	1	0.4	55	3.2
U	Intracellular trafficking and secretion	6	2.7	35	2.0
N	Cell motility, secretion and vesicular transport	0	0.0	16	0.9
S	Function unknown	0	0.0	184	10.6
V	Defense mechanisms	0	0.0	65	3.7

*Some genes are assigned in multiple COG categories.

**Fig. 1.**
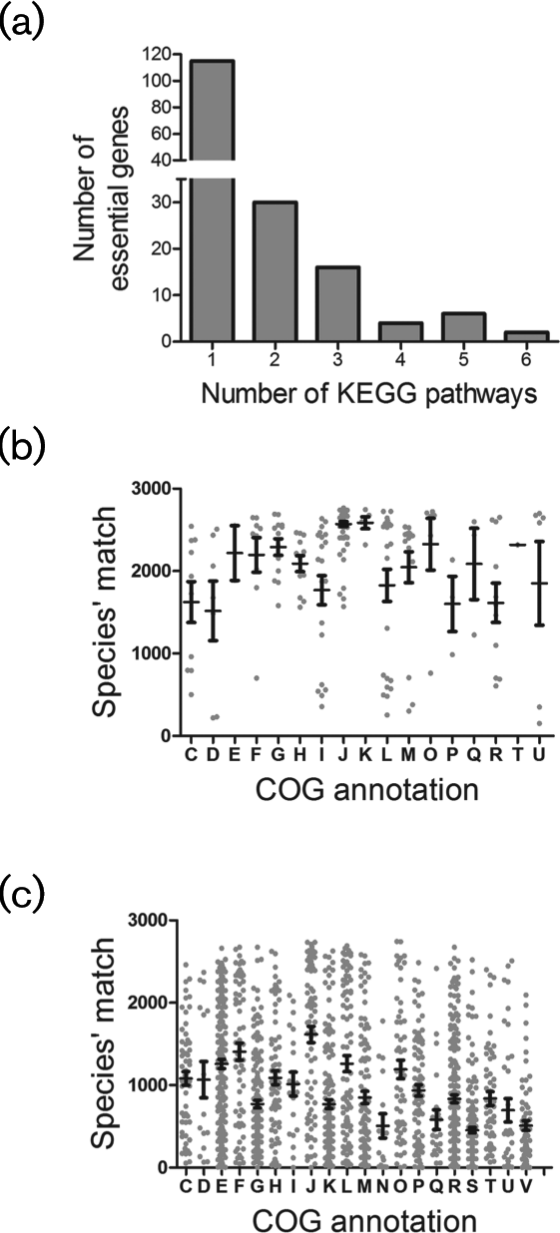
Bioinformatics analysis of functional categories and conservation of essential proteins. (a) Involvement of essential genes in *S. sanguinis* physiological pathways is shown in this bar chart. The number of essential genes (y-axis and number at the top of every chart bar) involved in the number of KEGG pathways (x-axis) is shown. Conservation of *S. sanguinis* essential (b) and non-essential (c) proteins across 2774 bacterial proteomes in relation to their COG annotations. Every dot on the scatter plots represents an (b) essential or (c) non-essential *S. sanguinis* protein. Proteins were clustered based on their functional categories as described by their COG annotations. Conservation of *S. sanguinis* proteins was determined by orthologues in 2774 bacterial proteomes. The average number of detected orthologues for every *S. sanguinis* protein in each COG category can be inferred from the y-axis projection of the mean (central horizontal bar) and the sem (vertical bar).

To further identify the pleiotropic functions of *S. sanguinis* essential genes, we measured the number of pathways in which every essential gene is involved, as described by the KEGG database (Fig. 1a). Although the majority of essential genes (115 genes) were shown to be involved in only one pathway, many may possess additional ‘moonlighting’ functions that are yet to be experimentally defined. Of special emphasis are many components of the genetic processing machinery (ribosomal components of 50S and 30S subunits, threonyl-tRNA synthetase, alanyl-tRNA synthetase and leucyl-tRNA synthetase), which have been established as pleiotropic players in many model species [46, 47]. Another major factor to consider is the absence of ‘genetic pathways’ in the KEGG database, which alternatively puts more focus on metabolic pathways.

Furthermore, we investigated the conservation of *S. sanguinis* essential and non-essential proteins across 2774 proteomes from bacteria with completely sequenced genomes deposited in the NCBI (Fig. 1b, c). We used scatter plots to display the conservation patterns of essential proteins through detecting their orthologues among 2774 bacterial species (Fig. 1b). All COG groups of essential proteins were conserved among more than 1000 species, so we intuitively considered 2000 species as a threshold. Similarly, for COG groups of non-essential proteins, all COG groups were conserved among less than 2000 bacterial species. The intuitive approach was to consider 1000 species as a threshold. It was obvious that the essential proteins identified in *S. sanguinis* are highly conserved among bacterial proteomes, albeit at different rates. Conservation was shown to be related to COG-based categorization: essential proteins belonging to ten COG groups (E, F, G, H, J, K, M, O, Q, T) showed the highest mean conservation values (conserved in more than 2000 species), with translation (COG group: J) and transcription (COG group: K) being top-ranked functional categories on the conservation list. The remaining essential proteins belonging to COG groups: C, D, I, L, P, R, S and U, showed mean conservation values between 1000 and 2000 species. In contrast, *S. sanguinis* non-essential proteins were shown to be less conserved than essential proteins among bacterial proteomes (Fig. 1c), with the most conserved non-essential genes belonging to eight COG groups (C, D, E, F, H, J, L, O) and displaying mean conservation values between 1000–2000 species, and the rest belonging to COG groups with mean conservation values below 1000 species. All in all, as functionally categorized groups, non-essential proteins displayed low mean conservation values, with their orthologues recovered in less than 2000 species, while *S. sanguinis* essential proteins showed remarkably high mean conservation values, with the vast majority of their orthologues detected in more than half of the investigated bacterial species.

### General overview of the transcriptome analysis

**Fig. 2.**
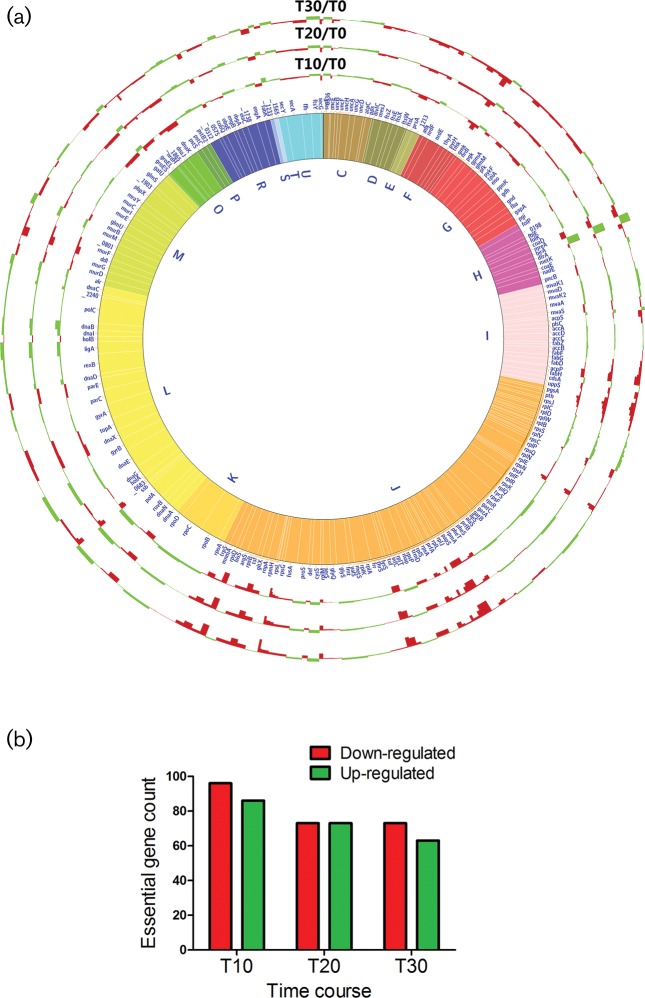
Differential expression of essential genes in antibiotic-treated *S. sanguinis* cells. (a) Circos plot representing the differential mRNA expression of essential genes at T_10_, T_20_, T_30_ indicative of 10, 20 and 30 min respectively post-treatment with a sub-inhibitory dose of ampicillin in comparison to T_0_ (untreated cells) in *S. sanguinis* SK36 strains. Green bars indicate a statistically significant up-regulation of gene transcription; red bars indicate a statistically significant down-regulation of gene transcription. Functional clustering was based on COG annotation, and further grouped into three essential functions as follows: G (green) for genetic information processing; C (blue) for cell wall biosynthesis; E (red) for energy production. (b) Bar chart showing the counts of up-regulated (green) and down-regulated (red) expression of essential genes at three time points.

RNA-seq analysis conducted on a temporal basis revealed the impact of gene regulation on a global basis. Considering the doubling time of *S. sanguinis* being around 20 min, and assuming a slight growth delay after ampicillin treatment, we decided to collect cells for RNA or protein extraction at early (T_10_), mid (T_20_) and late phases (T_30_) post-treatment with sub-inhibitory concentration of ampicillin (Fig. 2a, b, Table S1). Functional enrichment analysis (Table 2) of differentially regulated genes was conducted using the functional annotation tool of DAVID Bioinformatics Resources 6.7. ‘Phosphotransferase system’ was the functional category most enriched (22 non-essential genes) among the total up-regulated genes at T_10_ (total genes: 736 genes), T_20_ (total genes: 722 genes) and T_30_ (total genes: 652 genes). ‘Hydrogen ion transport’ and ‘signal transduction through two-component system’ (eight genes each) were enriched among up-regulated genes at T_10_ and T_20_ only. The enriched functional classes among up-regulated genes point towards modulating gene expression in a direction that potentiates an early stress response mechanism based on sensing the environmental cues, reducing internal proton buildup and importing energy resources.

**Table 2.** Functional enrichment and clustering of significantly up- and down-regulated genes and proteins in ampicillin-stressed cells

mRNA measurements					Protein measurements			
Time	Regulation	Functional category	*P*-value		Time	Regulation	Functional category	*P*-value
T_10_	Up-regulated	Phosphotransferase system	9.85E-05		T_10_	Up-regulated	Phosphorylation	7.1E-02
		Hydrogen ion transport	1.4E-02			Down-regulated	Ribosomal biogenesis	2.70E-30
		Signal transduction	2.2E-02				Glycolysis	1.20E-06
	Down-regulated	Ribosomal biogenesis	2.82E-06				Aminoacyl-tRNA biosynthesis	4.60E-05
		Peptidoglycan biosynthesis	1.25E-04				Protein folding	22E-04
		GTP-binding	8.8E-03		T_20_	Up-regulated	Ribosomal biogenesis	8.90E-05
T_20_	Up-regulated	Phosphotransferase system	4.84E-04			Down-regulated	Ribosomal biogenesis	9.50E-24
		Signal transduction	2.02E-02				Glycolysis	1.00E-05
		Hydrogen ion transport	4.33E-02				Aminoacyl-tRNA biosynthesis	1.30E-05
	Down-regulated	Ribosomal biogenesis	7.05E-04				Protein folding	1.40E-04
		Peptidoglycan biosynthesis	1.8E-03				Oxidoreductase activity	3.4E-02
		DNA replication	1.04E-02		T_30_	Up-regulated	Purine nucleoside-binding	9.62E-02
		Aminoacyl-tRNA biosynthesis	4.84E-02			Down-regulated	Ribosomal biogenesis	1.80E-29
T_30_	Up-regulated	Phosphotransferase system	8.39E-06				Glycolysis	1.50E-07
		Metal-binding	4.4E-02				Aminoacyl-tRNA biosynthesis	9.30E-05
	Down-regulated	Ribosomal biogenesis	3.2E-03				Cell division	1.01E-02
		Fatty acid biosynthesis	3.7E-03				RNA polymerase	1.42E-02
		ATP-binding	1.07E-02				Translational elongation	3.49E-02

‘Ribosomal biogenesis’ was the functional category most enriched (31, 19 and 25 genes) among the total down-regulated genes at T_10_ (total genes: 611), T_20_ (total genes: 445) and T_30_ (total genes: 644 genes), respectively. ‘Peptidoglycan biosynthesis’ was enriched among down-regulated genes at T_10_ (nine genes) and T_20_ (seven genes) only, ‘DNA replication’ at T_20_ (seven genes) and ‘fatty acid metabolism’ at T_30_ (eight genes). The enriched functional classes among down-regulated genes demonstrate a global transcriptional inclination towards slowing cell growth as the bacteria acclimate to the antibiotic (Table 2).

### Global overview of the proteome analysis

**Fig. 3.**
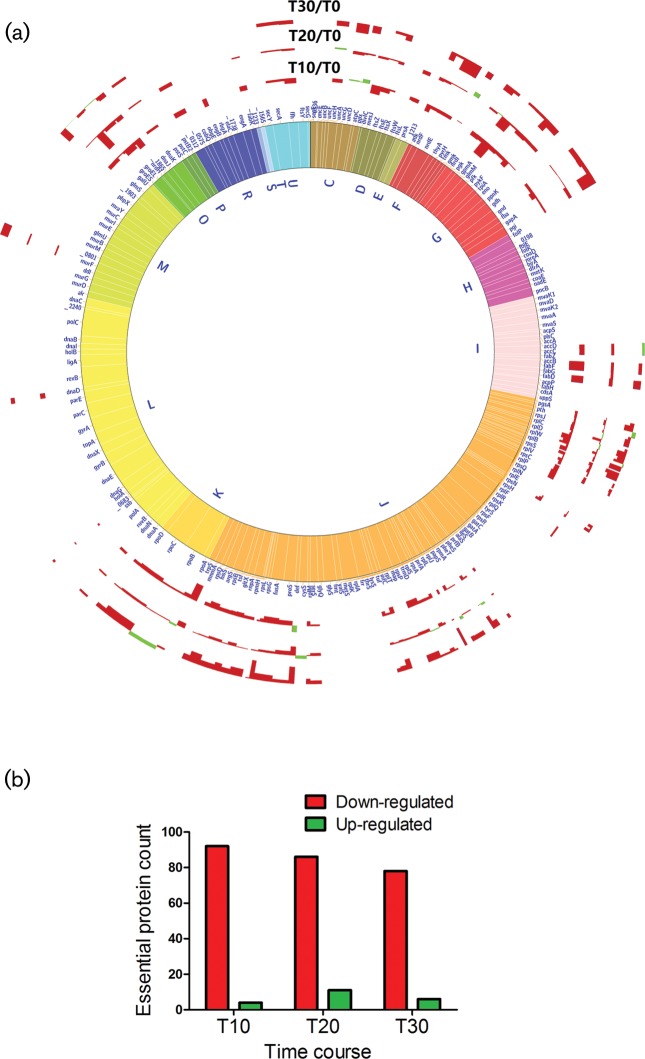
Differential expression of essential proteins in antibiotic-treated *S. sanguinis* SK36 cells. (a) Circos plot representing the differential expression of essential proteins at T_10_, T_20_, T_30_, indicative of 10, 20 and 30 min respectively post-treatment with a sub-inhibitory dose of ampicillin in comparison to T_0_ (untreated cells) in *S. sanguinis* SK36 samples. (b) Bar chart showing the counts of up-regulated (green) and down-regulated (red) expression of essential proteins at three time points.

Our proteomic study identified a total (essential and non-essential) of 269 proteins at T_10_, 268 proteins at T_20_ and 202 proteins at T_30_ with at least two unique peptides per protein. Almost half of the *S. sanguinis* essential proteins were detected at each of the three time points (Fig. 3, Table S1). A functional annotation analysis of these detected proteins using DAVID Gene Functional Annotation Clustering tool identified the following top over-represented functional groups among the up-regulated proteins: at T_10_, ‘phosphorylation’; at T_20_, ‘ribosomal biogenesis’; at T_30_, ‘purine nucleoside-binding’. Top over-represented functional groups among the down-regulated proteins at all time points were ‘ribosomal biogenesis’, ‘glycolysis’ and ‘aminoacyl-tRNA biosynthesis’ (Table 2). The 9.5-fold up-regulation of RelA enzyme, a major (p)ppGpp synthase [48, 49], at T_20_ suggests the orchestration of a stress response that impacts growth and persistence under stressful conditions by controlling sugar metabolism, ribosomal biogenesis and cell wall biosynthesis.

### Pathway-dependent interpretation of transcriptomic and proteomic profiles of essential genes and proteins

#### Ribosomal biogenesis

Ribosomes are the main energy consumers in the cell [50]. At T_10_, most essential genes encoding ribosomal proteins (28 genes) showed significant reduction in transcript level that extended to T_30_ (Fig. 2a, Table S1), except for nine genes: *rpsN* (SSA_2391), *rplP* (SSA_0114), *rpsQ* (SSA_0116), *rplN* (SSA_117), *rplE* (SSA_0119), *rpsH* (SSA_0120), *rplF* (SSA_0122), *rplR* (SSA_0123) and *rpsE* (SSA_0124). Proteomic findings showed the reduction in 29 ribosomal protein levels at all the time points, showing concurrence with their reduced transcription. Five ribosomal proteins (SSA_1105, SSA_0110, SSA_1104, SSA_1265, SSA_0113) were up-regulated only at T_20_, and two ribosomal proteins (SSA_0108, SSA_0117) were up-regulated only at T_30_.

#### Amino acid biosynthesis

**Fig. 4.**
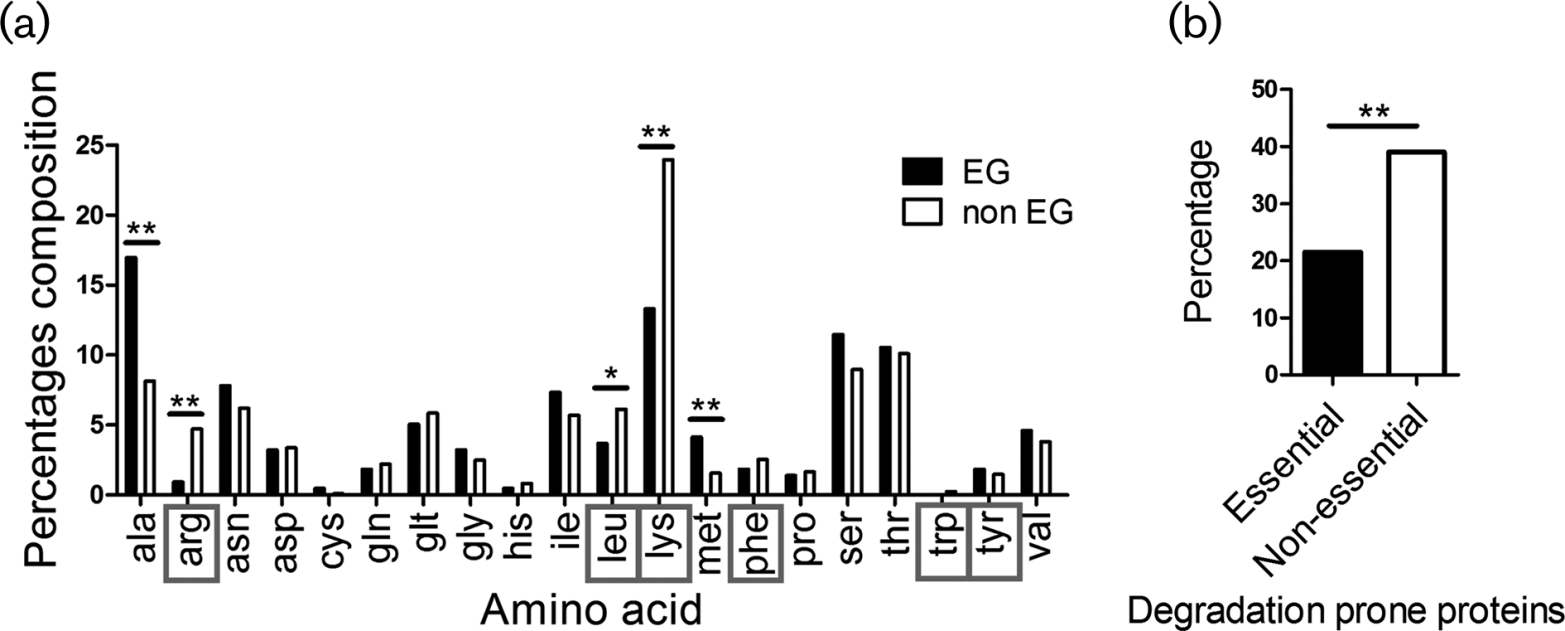
Determination of amino acids at the N-terminal positions in essential and non-essential proteins. (a) The localization of every amino acid in the predicted mature N-terminal position for essential and non-essential *S. sanguinis* proteins was counted and averaged using python scripts. For every amino acid, the difference between the composition percentage in essential versus non-essential proteins was tested for statistical significance. The six amino acids enclosed in a red square are the degradation-prone amino acids. EG, proteins encoded by essential genes; non EG, proteins encoded by non-essential genes. (b) Percentage of essential and non-essential proteins that possess a degradation-prone amino acid at their N-terminal position. *P-value<0.05; **P-value<0.001.

To better understand the transcriptomic and proteomic bias in protein biosynthesis, as demonstrated through up-regulation of amino acid-tRNA synthetases and amino acid biosynthetic enzymes, we measured the amino acid composition of essential and non-essential proteins at the predicted mature N-terminal position (amino acid number two; Fig. 4a, Tables 2 and 3). We postulated that essential proteins are less likely to contain ‘degradation-prone’ amino acids at their N-terminal position [51], due to their need to persist longer than non-essential proteins in the cell to secure execution of essential functions. In other words, we expected essential proteins to possess less of the following amino acids at the predicted mature N-terminal position: tyrosine, tryptophan, leucine, phenylalanine, lysine and arginine. We found that essential proteins do contain less of these amino acids at this position (21.5 %) than the non-essential proteins (39 %) (Fig. 4b, Table S4), suggesting a longer half-life for essential proteins.

**Fig. 5.**
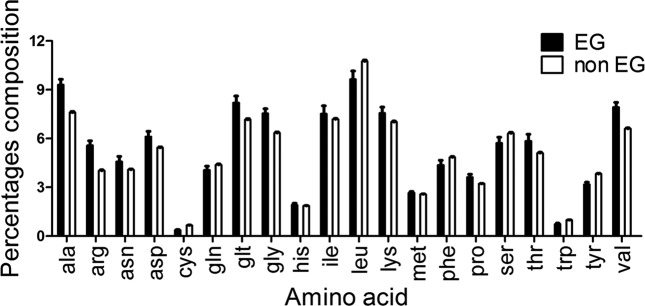
Amino acid composition of essential and non-essential proteins in *S. sanguinis*. Amino acid sequences were extracted from the NCBI database. Amino acid composition of essential and non-essential proteins was averaged from individual protein compositions.

We further investigated the total amino acid composition of essential (Table S5) and non-essential proteins (Table S6), looking for potential bias in amino acid composition (Fig. 5). We detected a significantly higher contribution of alanine, glutamate, arginine and valine to the composition of essential proteins than to the non-essential ones. Lysine and tyrosine were the only amino acids contributing significantly more to the composition of non-essential proteins than to the essential ones. It was interesting to note that although arginine and lysine, degradation-prone amino acids, are enriched more in the essential proteins than in the non-essential ones, they were less localized at the N-terminal position in essential proteins (arg: 0.9 %; lys: 3.6 %) than in the non-essential proteins (arg: 4. 7 %; lys: 6.1 %). This reflects a delicate selection of amino acids for the structural composition of essential proteins in a way to govern protein longevity and persistence of essential functions.

Dissecting the amino acid composition of essential proteins (Fig. 5) provided clues about the biased transcriptomic up-regulation of genes encoding specific amino acid-tRNA synthetases: alanine-tRNA synthetase (SSA_0756), glycine-tRNA synthetase (SSA_1879), methionine-tRNA synthetase (SSA_1848), prolyl-tRNA synthetase (SSA_2069), isoleucyl-tRNA synthetase (SSA_0661), threonyl-tRNA synthetase (SSA_1571) and valine-tRNA synthetase (SSA_1819). Given the fact that alanine, glycine, methionine and valine have higher abundance in the composition of essential proteins than the non-essential proteins, this may be a factor contributing to the transcription bias of these amino acid-tRNA synthetases.

Unfortunately, we did not detect the protein levels of these enzymes within our proteome data, and therefore the link between expression of an amino acid-tRNA synthetase and its respective up-regulation in the essential proteome could not be confirmed.

Previously, we have shown that enzymes involved in amino acid biosynthesis would be essential if chemically defined medium was used instead of the nutritionally enriched BHI medium [9]. We investigated the biosynthesis pathways of all amino acids using the KEGG maps and identified a transcriptomic and proteomic down-regulation of most amino acid biosynthesis enzymes. Two exceptions from the proteomic data at T_10_ were noted: first, the strategically positioned IlvE enzyme (SSA_1225; E.C. 2.6.1.42) at the rate-determining step of the biosynthetic pathways for valine (Fig. S2) was elevated 3.5-fold. Second, the GlnA enzyme (SSA_0307) responsible for interconversion between glutamate and glutamine was up-regulated threefold. Interestingly, valine and glutamate are more abundant among the essential proteome than among the non-essential one, and this may be a contributor to their biosynthesis at times of energy scarcity.

#### Glycolysis

**Fig. 6.**
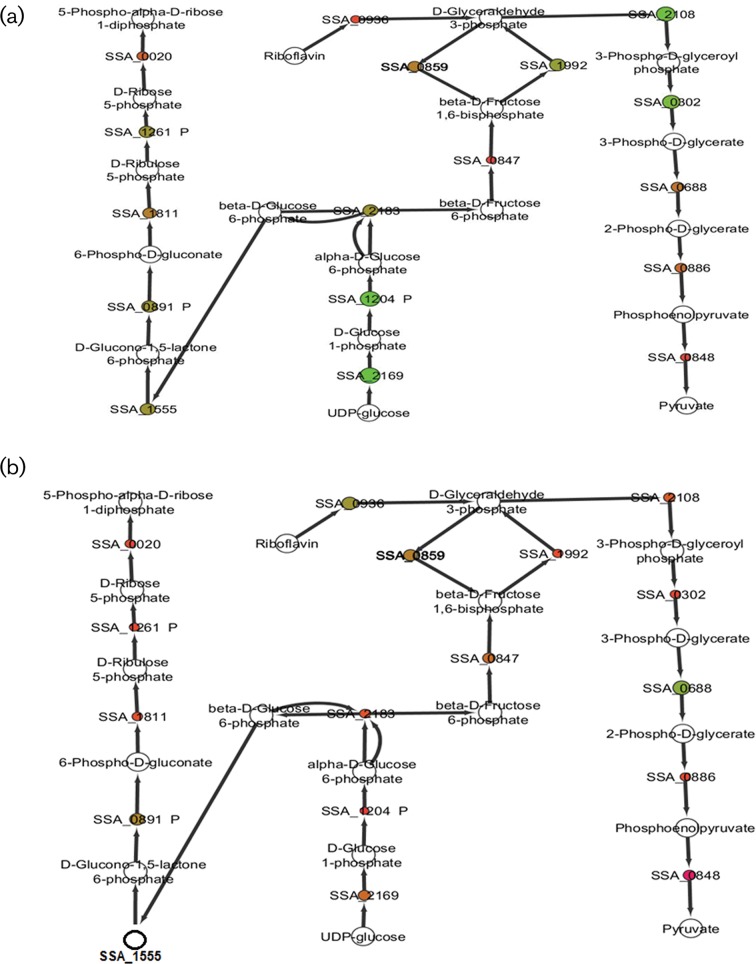
Glycolysis pathway map showing differential expression of (a) essential genes and (b) essential proteins in *S. sanguinis* exposed to a sub-inhibitory dose of ampicillin for 20 min. The genes/proteins (circles) are size and colour-coded based on an intensity spectrum where a large green circle indicates up-regulation, a small red circle indicates down-regulation and a blank circle shows no significant detection. Non-essential genes/proteins are labelled with ‘P’ after gene/protein name.

Glycolysis represents a fundamental source of energy production and supplier of products for many anabolic pathways. A significant transcriptional up-regulation of essential genes involved in conversion of UDP-glucose to 3-phospho-d-glycerate (SSA_2169, SSA_2183, SSA_0302) was observed, with the remaining three enzymes leading to pyruvate production being transcriptionally down-regulated (SSA_0688, SSA_0886, SSA_0848) across all three time points (Fig. 6a). However, proteomic findings revealed down-regulation of eight glycolytic enzymes across all time points, except for SSA_0688 which showed a slight protein increase at T_20_ (Fig. 6b). At T_10_ and T_30_, all proteins involved in glycolysis were down-regulated.

#### Cell wall biosynthesis

Terpenoid, peptidoglycan, amino sugar, glycerophospholipid and phosphatidyl glycerol biosynthesis pathways converge to produce the cell wall in *S. sanguinis* (Fig. S3). Transcriptomic data across the three time points demonstrated a general down-regulation of most essential genes encompassed in the amino sugar, phosphatidyl glycerol, glycerophospholipid and peptidoglycan biosynthetic pathways, with minor exceptions. Four genes involved in the terpenoid pathway (SSA_0334, SSA_0335, SSA_0336, SSA_0337) were transcriptionally up-regulated at all three time points, although the enzyme of rate-limiting reaction (SSA_2073) in the pathway was significantly down-regulated. Proteomic findings showed complete down-regulation of proteins belonging to the peptidoglycan, amino sugar and glycerophospholipid biosynthesis. Unfortunately, no proteins of the terpenoid biosynthesis pathway were detected.

#### Nucleic acid biosynthesis

Transcriptomic analysis of genes involved in the pentose phosphate pathway, RNA biosynthesis and DNA biosynthesis showed a general down-regulation. TetR (SSA_0927) was shown to be elevated 11.8-fold at T_30_. TetR repressors have been linked to antibiotic resistance [52, 53]. The impact of these transcriptional regulators on RNA polymerase activity under stress warrants further investigation.

#### Correlation between transcriptome and proteome

We calculated the correlation between the mRNA and protein levels of every gene and protein, respectively, which displayed statistically significant expression (Table 3). The mRNA/protein correlation was significantly higher (*P*-value: 0.0047) among the essential genes/proteins than among their non-essential counterparts (*P*-value: 0.0678), and this correlation increased with time in essential and non-essential categories. This may be explained by the chronological frame of events where transcription precedes translation and protein synthesis. In addition, a bacterial mRNA on average has a half-life less than 10 min [54] while proteins enjoy more longevity, although dependent on post-translational modifications, protein folding and degradation machinery [55]. At T_10_, correlation was observed in essential gene ratios only (in comparison to T_0_). It is noteworthy to emphasize that with essential gene ratios, the mRNA/protein correlation percentage was never less than 50 % at any of the three time points, while the opposite scenario happened during T_10_ with the non-essential genes. Moreover, the essential gene correlation percentages at T_20_ and T_30_ were much greater than 50 %, peaking at T_30_, unlike the non-essential genes at T_20_ and T_30_ where the percentages were lower.

**Table 3.** Correlation analysis between mRNA and proteins in ampicillin-stressed cells at three time points

Count (%) of correlated mRNA/protein	T_10_/T_0_	T_20_/T_0_	T_30_/T_0_	*P*-value**
Correlated essential mRNA/protein expressions*	111 (50 %)	131 (59 %)	117 (66 %)	0.0047
Non-correlated essential mRNA/protein expressions	111 (50 %)	90 (41 %)	60 (34 %)	
Correlated non-essential mRNA/protein expressions	59 (43 %)	73 (53 %)	60 (57 %)	0.0678
Non-correlated non-essential mRNA/protein expressions	79 (57 %)	66 (47 %)	45 (43 %)	

*All expression values of mRNAs and proteins at different time points were normalized to untreated *S. sanguinis* SK36 samples.

**P-value<0.05 was considered significant.

## Discussion

This is the first study to address concomitantly in a high throughput approach the transcriptomic and proteomic dynamics of essential genes and proteins on a temporal basis in *S. sanguinis* under ampicillin-induced stress. The complex response of *S. sanguinis* to antibiotic stress is indispensable for survival through adaptive transcription and protein synthesis, necessitating the use of a systems biology approach through RNA-seq and mass spectrometry to examine the dynamics of mRNA levels and the protein inventory at different time points. Combining transcriptomic and proteomic analysis under the same cultivation conditions and same time points we aimed to correlate our protein abundance findings with their corresponding transcriptional profiles, to define ‘pathogenesis profiles’ as novel therapeutic targets.

Drawing a correlation between mRNA and protein levels sheds light on the biological intricacies of transcription, translation, mRNA stability and protein degradation [54, 56], drawing a framework for gene/protein regulation that can be therapeutically targeted. Although bacteria lack the complex regulatory mechanisms harnessed by eukaryotic systems, such as poly-ubiquitination and proteasomes, it has been shown that bacteria have lower mRNA/protein correlations [57–59] in comparison to eukaryotes [60, 61]. Interestingly, using the functional pathways approach, it was shown that kinases, cell cycle genes, signalling and metabolic proteins display the highest mRNA/protein correlations in the yeast *Schizosaccharomyces pombe* [62]. We hypothesized that since these proteins are conducting essential functions, it is worth testing the essential mRNA/protein correlation in our bacterial model under stress, which from an evolutionary perspective, shapes the essential genome. Moreover, codon usage has been linked to higher mRNA/protein correlation [57]. Since highly expressed genes, including essential genes, have optimized their codon usage for a high sustainable expression [63, 64], added to the observed high conservation rates across species and high structural stability (low instability index below 40) (Table S1) of essential proteins, we expected these factors to enhance the essential mRNA/protein ratios. Our findings support this rationale by essential genes exhibiting better mRNA/protein ratios than non-essential genes at all time point measurements (Table 3), and it improved with time as the bacteria adjusted to the antibiotic shock. The correlation was not perfect for many reasons. First, the proteome was not completely measured and less than half of the essential proteins were covered due to the low sensitivity of the mass spectrometry used and the protein extraction protocol was focused on cytoplasmic proteins. Second, recent findings in *E. coli* stressed the role of mRNA secondary structure, more than codon usage, in modulating gene expression [65]. Third, the set of essential genes identified from *S. sanguinis* cultured in BHI may vary from that of ampicillin-treated *S. sanguinis*. Finally, the moonlighting behaviour of essential genes complicates analysis of their differential behaviour, such as the glycolytic enzymes enolase [66] and pyruvate oxidase [67, 68]. Further investigation is needed for a better understanding of this biological equation.

Adopting the pathway-dependent approach (Fig. S3) facilitated the task of delineating the topological distribution of essential genes and proteins. The temporal factor integrated into our experimental design highlighted the chronological cascade of events, where genes exhibited an immediate stress response against ampicillin at T_10_ through adaptive regulation and mRNA expression, while protein changes at T_20_ and T_30_ demonstrated the time lag between transcription and adaptive production of translated proteins as bacterial cells were undergoing replication. Bioinformatic analyses suggested that the immediate transcriptomic responses correlate with short-lived transcripts while the ‘slower’ protein responses correlate with a more persistent and conserved response, as exemplified through findings in yeast [69] and *Caenorhabditis elegans* [70]. Comparing conservation of *S. sanguinis* essential proteins involved in transcription versus translation, we have shown that translation is more enriched in essential and conserved proteins than transcription. Moreover, the measured stress response essential proteins, especially the ones involved in translation, demonstrated to a large degree a high conservation rate across species and low instability index (Table S1). This further highlighted the reliability of translation-related essential proteins as ‘proteomic signatures’ that dictate the cell’s physiology and even the energy status, especially since it was shown earlier that translation proteins consume more than 70 % of the cellular ATP pool [50]. In addition to their major role in protein synthesis, ribosomes have been implicated in pleiotropic functions, ranging from antibiotic adaptation [71] to fatty acid biosynthesis [72], in which we postulate these exceptionally up-regulated ribosomal proteins may be involved. Whether the pattern of differentially expressed *S. sanguinis* proteins during the stress response is conserved across other bacterial species is currently unclear. Such a biological question is worth further investigation, because if the conservation of essential proteins projects as a conserved regulation of stress responses across diverse bacterial species, then essential proteins’ dynamics under stress will provide a wealth of proteomic signatures that may serve as potential therapeutic targets.

Upon exposure to a sub-inhibitory concentration of ampicillin, transition to slow growth was observed (Fig. S1) accompanied by extensive reprogramming of gene expression across all major essential pathways, including glycolysis, purine and pyrimidine synthesis, cell wall biosynthesis, transcription and translation. Although we did not conduct any metabolomic experiments, we postulate that amino acids are in demand differentially based on their abundance in essential proteins, with cysteine as the least abundant constituent and leucine, alanine and valine the most. Essential genes tend to favour the less ‘degradation-prone’ amino acids as demonstrated by the N-rule as this enhances their persistence. This may explain in part the up-regulation of biosynthetic enzymes for isoleucine and valine (branched amino acids), glutamine and glutamate, where the stressed cells seem not to rely solely on the recycling of degraded proteins nor peptide import through ABC transporters to satisfy their need for building blocks of essential proteome. The need for branched amino acids has also been demonstrated in acid-stressed *Streptococcus mutans* [73] and *Streptococcus suis* isolated from porcine cerebrospinal fluid [74], showing promise for establishing a potential ‘stress proteomic signature’ based on amino acid composition of the essential proteome.

Taken together, this work is the first global study that monitors time-dependent changes of essential genes and proteins encountering antibiotic stress. Our study also emphasizes crucial switches for the adaptation of metabolic, cell wall biosynthesis and genetic information processing pathways.

## Supplementary Data

1023 Supplementary File 1
