# Supplementary material for: Analysis of essential gene dynamics under antibiotic stress in Streptococcus sanguinis
Source: Microbiology (Reading). 2018 Jan 9;164(2):173–85. doi: 10.1099/mic.0.000595 (PMC5882076; doi:10.1099/mic.0.000595)
Supplement: Supplementary File 1 [file mic-164-173-s001.pdf]

Analysis of Essential Gene Dynamics under Antibiotic Stress in *Streptococcus sanguinis*

Fadi El-Rami [1,2], Xiangzhen Kong[1], Hardik Parikh[2], Bin Zhu[1], Victoria Stone[1], Todd Kitten[1,2], Ping Xu [1,2 *]

Supporting information

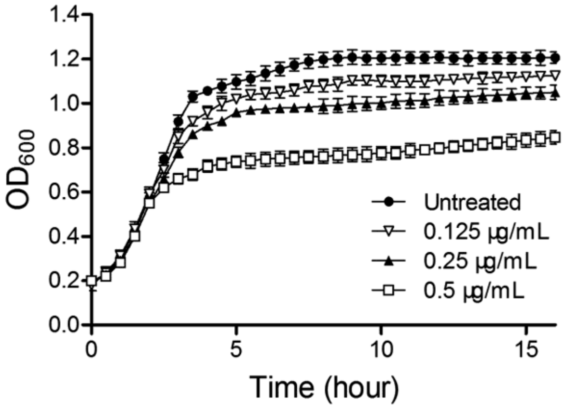

Supplementary Fig. 1. Growth rates of *S. sanguinis* samples with different ampicillin concentrations. Ampicillin was added at OD_{600} = 0.6. All experiments were conducted in triplicate.

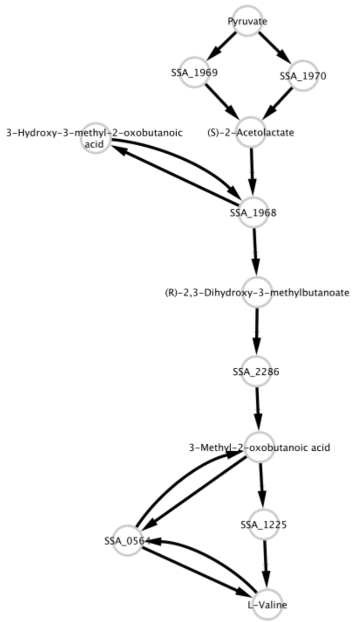

Supplementary Fig. 2. Valine biosynthesis pathway in *S. sanguinis* as described by KEGG database. The genes (circles) and the enzymatic reactions (arrows) are indicated.

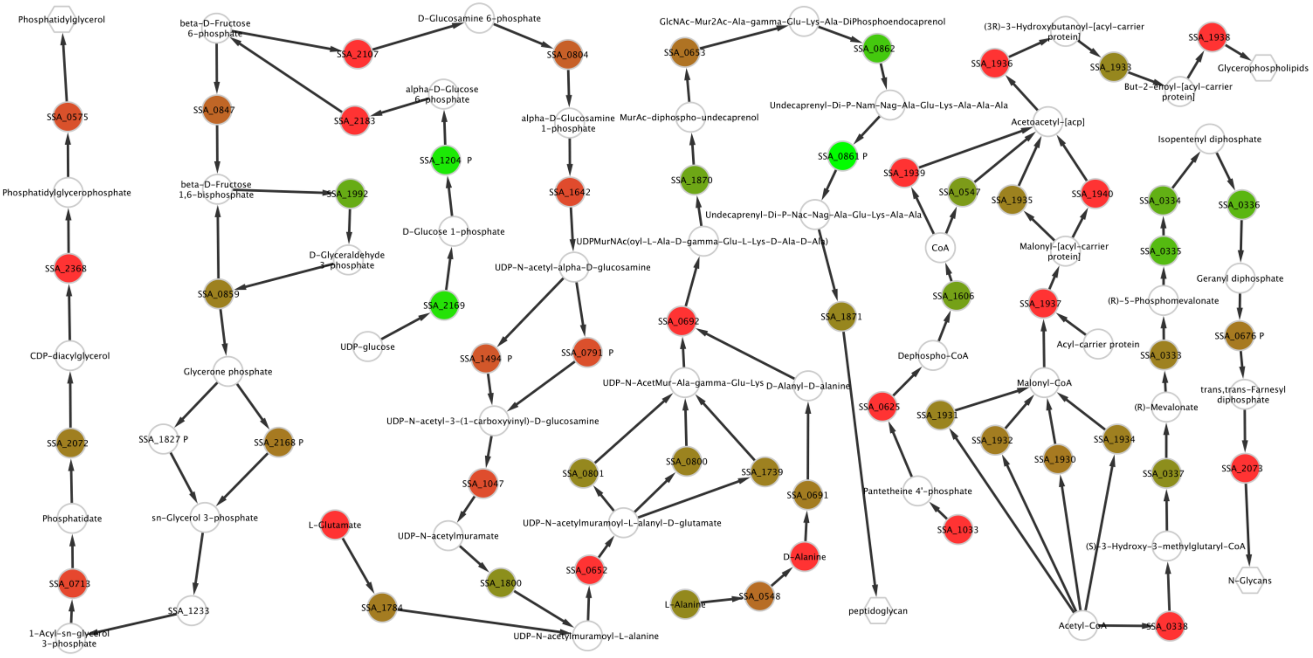

Supplementary Fig. 3. Transcriptional profiles of genes involved in cell biosynthesis pathways in *S. sanguinis* as described by KEGG database. The genes (circles) are color-coded green for up-regulation, red for down-regulation at T_{20}. Non-essential genes/proteins are labelled with “P” after gene/protein name.

Supplementary Table 1. Differential transcriptomic and proteomic expression profiles in *S. sanguinis* under antibiotic stress.

				mRNA fold change with respect to T1			protein fold change with respect to T1						
Name	Synonym	Product	COG annotation	T10/T0	T20/T0	T30/T0	T10/T0	T20/T0	T30/T0	Essentiality	Protein instability index ∞	Hydropathy values §	Protein conservation Ω
dnaA	SSA_0001	chromosomal replication initiation protein	COG0593L	0.636363636	ϕ	0.363636364				Yes	39.92935556	-0.29133333	2530
dnaN	SSA_0002	DNA polymerase III subunit beta	COG0592L	1.00625	0.85	0.83125	0.48555	0.6	0.642951	Yes	36.25452381	-0.07195767	2555
-	SSA_0004	lipoprotein	COG4808S	0.646551724		0.448275862				No	39.29515337	-0.61288344	240
ychF	SSA_0005	GTP-dependent nucleic acid-binding protein EngD	COG0012J	0.472222222	0.222222222	0.125	0.716537			No	33.15552561	-0.26684636	2733
pth	SSA_0006	peptidyl-tRNA hydrolase	COG0193J		0.346153846	0.230769231				Yes	18.00740741	-0.21904762	2567
trcF	SSA_0008	transcription-repair coupling factor	COG1197LK	0.852941176		0.5				No	37.15425021	-0.39562982	2425
-	SSA_0009	ribosome-associated heat shock protein (S4)	COG1188J	0.758333333		0.5				No	38.87727273	-0.48409091	1327
divIC	SSA_0010	cell division protein DivIC	COG2919D	0.792857143	0.735714286					Yes	63.64836066	-0.81557377	218
-	SSA_0011	hypothetical protein	-	0.911111111	0.8	0.811111111				No	36.57619048	-1.09047619	105
-	SSA_0012	Beta-lactamase class A	COG2367V	0.897435897	0.829059829	0.837606838				No	33.69955504	-0.44918033	218
mesJ	SSA_0013	PP family ATPase	COG0037D	0.840909091			0.084913	0.498801		Yes	43.12117647	-0.43058824	2435
hpt	SSA_0014	hypoxanthine guanine phosphoribosyltransferase	COG0634F	0.662921348		0.342696629				No	23.25163043	-0.09728261	1985
ftsH	SSA_0015	membrane ATPase FtsH	COG0465O							No	39.57738998	-0.29711684	2743
comX	SSA_0016	ComX1, transcriptional regulator of competence-specific genes	-							No	36.74596273	-1.09068323	154
mreC	SSA_0017	rod shape-determining protein MreC	COG1792M							No	27.76236162	-0.13357934	1550
mreD	SSA_0018	cell-shape determining protein MreD	COG2891M							No	46.15421687	1.331325301	123
pcsB	SSA_0019	secreted antigen GbpB/SagA; peptidoglycan hydrolase; PcsB	COG3883S		0.256673511	0.202258727				No	39.73366337	-0.3740099	403
prsA	SSA_0020	ribose-phosphate pyrophosphokinase	COG0462FE				0.344099	0.288609		Yes	48.90031153	0.013084112	2550
-	SSA_0021	hypothetical protein	-	1.163179916	1.213389121	1.125523013				No	57.81481481	-0.60846561	2
srtB	SSA_0022	surface protein cell wall anchor	COG4509S			0.580645161				No	45.58828571	-0.70107143	218
aspC	SSA_0023	aromatic amino acid aminotransferase	COG0436E	0.847457627	0.788135593					No	33.48464103	-0.05410256	1277
recO	SSA_0025	DNA repair protein RecO	COG1381L	0.968503937	0.94488189	0.905511811				No	37.8077821	-0.2151751	664
plsX	SSA_0026	glycerol-3-phosphate acyltransferase PlsX	COG0416I	1.016393443	0.975409836	0.909836066				No	21.49731928	-0.11024096	2019
acp	SSA_0027	acyl carrier protein	COG0236IQ	1.185840708						No	50.2097561	-0.36585366	1509
purC	SSA_0028	phosphoribosylaminoimidazole-succinocarboxamide synthase	COG0152F							No	24.99834043	-0.34553192	2262
purL	SSA_0030	phosphoribosylformylglycinamidine synthase	COG0046F							No	33.11447749	-0.18054662	2330
purF	SSA_0031	amidophosphoribosyltransferase	COG0034F							No	35.34891441	-0.2611691	2317
purM	SSA_0032	phosphoribosylaminoimidazole synthetase	COG0150F							No	29.43882353	0.033529412	2333
purN	SSA_0033	phosphoribosylglycinamide formyltransferase	COG0299F							No	25.44545455	-0.12032086	2226
-	SSA_0034	hypothetical protein	-							No	47.09370079	-0.37913386	2
purH	SSA_0035	bifunctional phosphoribosylaminoimidazolecarboxamide formylt	COG0138F							No	31.68559615	-0.16346154	2259
-	SSA_0036	hypothetical protein	COG3942R							No	26.86040062	-0.6248074	64
purD	SSA_0037	phosphoribosylamine--glycine ligase	COG0151F				0.315186			No	33.68695238	-0.08857143	2379
purE	SSA_0039	phosphoribosylaminoimidazole carboxylase catalytic subunit	COG0041F							No	34.44567901	0.22654321	2326
purK	SSA_0040	phosphoribosylaminoimidazole carboxylase ATPase subunit	COG0026F							No	43.38870523	-0.01515152	2002
-	SSA_0041	hypothetical protein	-	1.166666667	1.19047619	1.095238095				No	32.516	-0.12133333	20
-	SSA_0042	amino acid recemase	COG1794M				0.449048	0.187696	0.198689	No	38.58323276	0.018534483	842
-	SSA_0043	hypothetical protein	-	1.083333333	1.1	1.233333333				No	31.72933333	-0.11333333	43
-	SSA_0044	hypothetical protein	-	1	1	0.926829268				No	46.47965608	-0.35185185	3
-	SSA_0045	hypothetical protein	-							No	27.70617978	-0.02078652	4
purB	SSA_0046	adenylosuccinate lyase	COG0015F	0.949152542	1.093220339	1.059322034	0.42341	0.196272		No	46.57656613	-0.40208817	2570
-	SSA_0047	NAD(P)H dehydrogenase (quinone)	COG2249R	2.816666667	4.433333333	6.8				No	36.06648936	-0.31542553	806
-	SSA_0048	TetR/AcrR family transcriptional regulator	COG1309K	2.948717949	4.820512821	6.897435897				No	37.16212121	-0.21818182	436
-	SSA_0049	dihydroxyacetone kinase subunit DhaK	COG2376G	3.8	10.2	12.6				No	23.76474164	0.096960486	847
-	SSA_0050	dihydroxyacetone kinase phosphatase domain-containing prote	COG2376G	3.25	8.5	13.25				No	19.18020833	-0.18072917	713
-	SSA_0051	PTS system mannnose-specific transporter subunit IIA	COG3412S	3	9	16				No	14.82741935	0.372580645	422
-	SSA_0052	GntR family transcriptional regulator	COG2188K	1.603723404	2.234042553	2.75		0.077581	0.363886	No	24.4992437	-0.62478992	390
-	SSA_0053	beta-galactosidase	COG1874G	3.75	5.625	7.75				No	34.06220168	-0.38941177	215
-	SSA_0054	phosphotransferase system sugar-specific EIIB component	COG3444G	4.6	10.4	14.6	0.490418	0.270572	0.674577	No	34.68227848	-0.01139241	234
-	SSA_0055	PTS system transporter subunit IIC	COG3715G	3.5	8.5	10.83333333				No	32.07740864	0.709966777	286
-	SSA_0056	phosphotransferase system sugar-specific EIID component	COG3716G	2.833333333	7	10.83333333				No	32.05839416	0.367883212	197
-	SSA_0057	phosphotransferase system sugar-specific EII component	COG2893G	2	5.5	8.166666667				No	45.19328358	0.06119403	405
agaS	SSA_0060	tagatose-6-phosphate ketose/aldose isomerase	COG2222M				0.136219	0.512572		No	32.59362245	-0.0622449	349
-	SSA_0061	tagatose 1,6-diphosphate aldolase	COG3684G							No	32.17623188	-0.2173913	217
galM	SSA_0062	aldose 1-epimerase	COG2017G							No	27.52158046	-0.35172414	1073
ruvB	SSA_0063	Holliday junction DNA helicase RuvB	COG2255L	0.964285714	1.017857143	0.839285714				Yes	37.71629518	-0.20271084	2538
-	SSA_0064	hypothetical protein	COG3575S	1.114754098	1.229508197	1.163934426				No	57.96772487	-0.68624339	354
-	SSA_0065	low molecular weight phosphotyrosine protein phosphatase	COG0394T	0.64		0.24				No	45.70357143	-0.55428571	1635
-	SSA_0066	hypothetical protein	COG4642S	0.934782609		0.630434783				No	4.281094891	-0.42919708	338
-	SSA_0067	acyltransferase	COG1835I	1.2	1.2	1.181818182				No	36.64101498	0.424126456	1049
adhE	SSA_0068	bifunctional acetaldehyde-CoA/alcohol dehydrogenase	COG1454C	5.578431373	9.132352941	8.882352941	0.385399	0.778409	0.292068	No	31.62218326	-0.22952489	1048
-	SSA_0069	hypothetical protein	-							No	38.89654867	0.866814159	3
axe1	SSA_0070	acetylxylan esterase	COG3458Q							No	23.32492492	-0.18258258	277
nanE	SSA_0071	N-acetylmannosamine-6-phosphate 2-epimerase	COG3010G	66	176	243				No	37.69353448	0.031034483	727
-	SSA_0072	sugar ABC transporter	COG3839G	46.5	139.5	231.5				No	29.93159151	-0.20503979	766
-	SSA_0073	hypothetical protein	COG2731G	54	161	276	0.527101	1.599143		No	29.89	-0.19666667	574
-	SSA_0074	sugar ABC transporter substrate-binding protein	COG1653G	28.5	92.5	189.5				No	27.63779018	-0.36741071	871
-	SSA_0075	sugar ABC transporter permease	COG1175G	17	63	131				No	27.19493197	0.758843537	989
-	SSA_0076	sugar ABC transporter permease	COG0395G	9	37	92				No	30.09212996	0.911552347	1386
-	SSA_0077	oxidoreductase	COG0673R	6	28	87				No	31.25967302	-0.3386921	1531
-	SSA_0078	N-acetylneuraminate lyase	COG0329EM	3	10	36				No	42.15085246	-0.03344262	590
glk	SSA_0079	ROK family protein	COG1940KG							No	29.94323129	0.070408163	503
blpT	SSA_0080	superfamily I DNA/RNA helicase	-							No	57.00376147	-0.17614679	52
-	SSA_0081	RpiR family transcriptional regulator	COG1737K				5.635206			No	52.84628975	-0.28833922	1369
-	SSA_0083	hypothetical protein	-	0.720779221		0.441558442				No	45.01869159	-0.72523365	38
ntpI	SSA_0085	V-type ATP synthase subunit I	COG1269C	0.903381643	0.898550725	0.845410628				No	36.1955914	0.213671275	548
ntpK	SSA_0086	V-type ATP synthase subunit K	COG0636C	0.936708861	1.03164557	1.037974684				No	31.71006289	0.886163522	336
ntpE	SSA_0087	V-type sodium ATPase subunit E	COG1390C	1.019900498	1.089552239	1.134328358				No	59.47959184	-0.725	70
ntpC	SSA_0088	V-type sodium ATPase subunit C	COG1527C	1.03271028	1.158878505	1.163551402				No	40.86709199	-0.11691395	175
ntpG	SSA_0089	V-type ATP synthase subunit F	COG1436C	1.03930131	1.187772926	1.240174672				No	39.88396226	-0.01981132	170
-	SSA_0090	acetyltransferase	COG1670J	1.067924528	1.260377358	1.366037736				No	34.75454545	-0.77272727	786
ntpA	SSA_0091	V-type ATP synthase subunit A	COG1155C	1.115107914	1.381294964	1.514388489				No	35.2847512	-0.14751204	553
ntpB	SSA_0092	V-type ATP synthase subunit B	COG1156C	1.148997135	1.46991404	1.759312321				No	31.10625	-0.27112069	627
ntpD	SSA_0093	V-type ATP synthase subunit D	COG1394C	1.0657277	1.32629108	1.457746479				No	58.36618357	-0.49613527	385
-	SSA_0094	cell wall metabolism, LysM type protein	COG5373S	0.777777778	0.416666667	0.416666667				No	46.75454795	-0.29452055	303
thrC	SSA_0095	threonine synthase	COG0498E	1.032520325	0.894308943	0.772357724	0.399651			No	31.78605263	-0.07004049	1477

norN	SSA_0097	MATE family transporter	COG0534V	1.025	1.1	0.95				No	24.71625882	0.898588235	973
-	SSA_0098	HAD superfamily hydrolase	COG0546R							No	31.39607843	-0.28431373	426
-	SSA_0099	hypothetical protein	-							No	43.09509317	0.213664596	5
polA	SSA_0100	DNA polymerase I	COG0749L	1.391752577	1.659793814	1.773195876				Yes	38.03172727	-0.28772727	2576
-	SSA_0101	hypothetical protein	COG1832R	1.52173913	1.739130435	1.913043478	0.36278	0.526449	0.296252	No	29.46551724	-0.34068966	1096
-	SSA_0102	hypothetical protein	COG0563F	1.440559441	1.776223776	2.363636364				No	42.83711765	-0.57176471	117
-	SSA_0103	hypothetical protein	COG5523S	1.052631579						No	37.23258741	0.413636364	304
tgt	SSA_0104	queuine tRNA-ribosyltransferase	COG0343J	0.7		0.466666667				No	45.86423684	-0.39973684	2476
-	SSA_0105	uridine kinase	COG0572F	0.923076923		0.630769231				No	51.56754717	-0.31320755	58
rpsJ	SSA_0106	30S ribosomal protein S10	COG0051J	0.659043659	0.388773389	0.288981289	0.477509	0.920523	0.429872	Yes	25.15490196	-0.44215686	2726
rplC	SSA_0107	50S ribosomal protein L3	COG0087J	0.732580038		0.380414313	0.519203	0.504673	0.146391	Yes	27.61105769	-0.24134615	2757
rplD	SSA_0108	50S ribosomal protein L4	COG0088J	0.805687204			0.59434	0.906504	2.208756	Yes	34.65024155	-0.08309179	2596
rplW	SSA_0109	50S ribosomal protein L23	COG0089J	0.920955882	0.722426471		0.453559	0.684874	0.5648	Yes	20.01326531	-0.23571429	2326
rplB	SSA_0110	50S ribosomal protein L2	COG0090J	0.916886544			0.399533	1.309309	0.331395	Yes	40.57436823	-0.54440433	2761
rpsS	SSA_0111	30S ribosomal protein S19	COG0185J	1	0.894132653	0.854591837	0.650716	0.055465		Yes	37.96129032	-0.85591398	2723
rplV	SSA_0112	50S ribosomal protein L22	COG0091J	1.061011905	1.03125	1.098214286	0.480851	0.809249	0.576419	Yes	41.81578947	-0.16754386	2680
rpsC	SSA_0113	30S ribosomal protein S3	COG0092J	1.043126685	1.041778976	0.994609164	0.062283	1.501916	0.13692	Yes	27.41981567	-0.44884793	2759
rplP	SSA_0114	50S ribosomal protein L16	COG0197J	1.038825758	1.104166667	1.112689394	0.137425	0.756757	0.209936	Yes	43.11751825	-0.48832117	2614
rpmC	SSA_0115	50S ribosomal protein L29	COG0255J	1.104895105	1.242424242	1.268065268	0.292347	0.505938	0.371103	No	40.25147059	-0.79264706	2058
rpsQ	SSA_0116	30S ribosomal protein S17	COG0186J	1.131278539	1.287671233	1.311643836	0.0605	0.219095	0.115806	Yes	27.07674419	-0.58372093	2647
rplN	SSA_0117	50S ribosomal protein L14	COG0093J	1.06	1.16	1.115714286	0.580348	0.71978	1.311321	Yes	23.51721311	-0.11967213	2740
rplX	SSA_0118	50S ribosomal protein L24	COG0198J	1.132127955	1.372739917	1.385257302				No	4.775247525	-0.38415842	2559
rplE	SSA_0119	50S ribosomal protein L5	COG0094J	1.131024096	1.353915663	1.370481928	0.127666	0.573171	0.074437	Yes	20.89722222	-0.17111111	2751
rpsH	SSA_0120	30S ribosomal protein S8	COG0096J	1.151476252	1.405648267	1.42618742	0.146749	0.295841	0.052314	Yes	28.72878788	-0.24545455	2747
-	SSA_0121	hypothetical protein	-	1.254237288	1.647457627	1.822033898				No	30.62033898	0.211864407	1
rplF	SSA_0122	50S ribosomal protein L6	COG0097J	1.191542289	1.514925373	1.60199005	0.723473	0.487952	0.088699	Yes	24.73483146	-0.44494382	2744
rplR	SSA_0123	50S ribosomal protein L18	COG0256J	1.167272727	1.585454545	1.646060606	0.25328	0.356719		Yes	15.42118644	-0.41949153	2569
rpsE	SSA_0124	30S ribosomal protein S5	COG0098J	1.114790287	1.458057395	1.488962472	0.42158	0.647059	0.677686	Yes	28.13902439	0.15	2759
rpmD	SSA_0125	50S ribosomal protein L30	COG1841J	1.087093389	1.465897167	1.573976915	0.14018	0.772905	0.130354	No	29.35166667	0.143333333	2028
rplO	SSA_0126	50S ribosomal protein L15	COG0200J	1.066978193	1.381619938	1.406542056	0.977612	1.354839	0.428736	No	30.80205479	-0.4390411	2601
secY	SSA_0127	preprotein translocase subunit SecY	COG0201U	1.104201681	1.430252101	1.490756303				Yes	27.19128736	0.625747126	2675
adk	SSA_0128	adenylate kinase	COG0563F	0.815165877	0.672985782	0.36492891	0.123899	0.350267	0.161429	Yes	30.01132075	-0.45707547	2645
infA	SSA_0129	translation initiation factor IF-1	COG0361J	0.846715328		0.444038929				Yes	25.80416667	-0.24166667	2562
rpsM	SSA_0130	30S ribosomal protein S13	COG0099J	0.964420893			0.526745	0.756345	0.474131	No	41.38512397	-0.46198347	2710
rpsK	SSA_0131	30S ribosomal protein S11	COG0100J	0.99673913	0.741304348	0.45	0.273932	0.404181	0.540104	Yes	47.06692913	-0.33070866	2730
rpoA	SSA_0132	DNA-directed RNA polymerase subunit alpha	COG0202K				0.361496	1.767123	0.705939	Yes	34.24839744	-0.18974359	2596
rplQ	SSA_0133	50S ribosomal protein L17	COG0203J	1.083251714	0.851126347	0.490695397	0.251553	0.354041	0.628571	Yes	51.89765625	-0.5953125	2576
mrcB	SSA_0134	membrane carboxypeptidase	COG0744M	0.876190476	0.771428571					No	32.71181592	-0.33358209	306
adcR	SSA_0135	multiple antibiotic resistance operon transcription repressor Ma	COG1846K			0.514285714				No	34.27421769	-0.29251701	248
adcC	SSA_0136	zinc ABC transporter	COG1121P	0.844827586						No	38.65898305	-0.64364407	898
adcB	SSA_0137	zinc ABC transporter permease	COG1108P	0.952380952						No	40.95783582	1.198880597	658
adcA	SSA_0138	Zn-binding lipoprotein	COG3443R	0.963636364	0.909090909	0.836363636				No	33.33964	-0.6078	998
-	SSA_0139	copper transport operon or penicillinase transcriptional repress	COG3682K							No	47.37897959	-0.55034014	360
ctpA	SSA_0140	copper-translocating P-type ATPase	COG2217P							No	29.70669786	0.30868984	2487
-	SSA_0141	copper chaperone	COG2608P				0.009866		0.012177	No	2.1	-0.27083333	275
-	SSA_0142	hypothetical protein	-	0.676923077		0.369230769				No	42.57118644	0.228813559	1
-	SSA_0143	hypothetical protein	-	0.648044693		0.301675978	1.129823		545.31	No	10.04333333	-0.34504505	12
-	SSA_0144	TetR family transcriptional regulator	COG1309K	1.024193548	0.862903226	0.774193548				No	41.44421053	-0.24684211	15
-	SSA_0145	TetR family transcriptional regulator	COG1309K	1.450980392	1.274509804	1.117647059				No	55.202	-0.3225	29
-	SSA_0146	DNA repair ATPase	COG0419L							No	43.73977415	-0.51681305	34
-	SSA_0148	sugar ABC transporter ATP-binding protein	COG3839G	2.141025641	2.794871795	2.5				No	30.16515957	-0.2087766	541
-	SSA_0149	hypothetical protein	-							No	29.49508475	0.708474576	1
-	SSA_0150	hypothetical protein	-							No	35.00895782	-0.43225807	1
-	SSA_0151	hypothetical protein	-							No	38.91462069	0.837931034	1
-	SSA_0152	hypothetical protein	COG3505U							No	44.49466316	-0.41547368	26
-	SSA_0153	hypothetical protein	-							No	43.58435484	0.075	5
-	SSA_0154	hypothetical protein	COG1293K					0.304848		No	45.61046512	-0.56773256	11
-	SSA_0155	hypothetical protein	-							No	49.44628099	-0.15041322	6
-	SSA_0156	chaperone ATPase	COG0542O							No	37.48873041	-0.36661442	17
-	SSA_0157	hypothetical protein	-	1.344262295		0.704918033				No	29.24574468	0.821276596	1
-	SSA_0158	hypothetical protein	-							No	47.53551637	-0.48866499	19
-	SSA_0159	hypothetical protein	COG3846U	1.333333333	1.208333333	1.041666667				No	22.27782675	0.060638298	24
-	SSA_0160	hypothetical protein	-							No	43.86581197	-0.85854701	12
-	SSA_0161	hypothetical protein	-							No	37.9531938	-0.40806202	78
-	SSA_0162	hypothetical protein	-							No	29.3095045	0.376126126	1
-	SSA_0163	hypothetical protein	-							No	33.80810811	-0.8981982	1
-	SSA_0164	hypothetical protein	COG1705NU							No	36.57413361	-0.32317328	29
-	SSA_0165	hypothetical protein	-							No	38.96169154	-0.54527363	12
-	SSA_0166	hypothetical protein	-	0.916666667	0.916666667	0.916666667				No	39.07801418	0.217021277	1
-	SSA_0167	hypothetical protein	COG5373S	1.35	1.575	1.65				No	37.10870871	-1.22432432	1
-	SSA_0168	hypothetical protein	-							No	55.24205521	-0.42883436	18
-	SSA_0169	hypothetical protein	-	1.086956522	0.97826087	0.847826087				No	44.0671875	-0.3671875	1
-	SSA_0170	hypothetical protein	-	0.891304348	0.717391304					No	36.99322917	-0.37239583	1
-	SSA_0171	Cro family transcriptional regulator	COG1476K	0.74						No	17.64920635	-0.20476191	74
-	SSA_0172	XRE family transcriptional regulator	COG1396K							No	42.72933333	-0.26133333	191
rrmA	SSA_0173	23S rRNA m(1)G745 methyltransferase	COG2226H							No	50.13193548	-0.36200717	980
tyrS	SSA_0174	tyrosyl-tRNA synthetase	COG0162J	0.993421053	0.927631579	0.828947368	0.170099			Yes	39.91291169	-0.37231504	2582
pbp1b	SSA_0175	penicillin-binding protein 1B	COG0744M	0.875	0.776785714					No	33.75426667	-0.3436	141
rpoB	SSA_0176	DNA-directed RNA polymerase subunit beta	COG0085K	1.125			0.660057	0.805955	2.063871	Yes	32.87586513	-0.41517303	2675
rpoC	SSA_0177	DNA-directed RNA polymerase subunit beta'	COG0086K	1.226415094	1.335429769	1.28721174	0.308139	0.475096	0.176715	Yes	34.74741564	-0.28074074	2747
epsC	SSA_0178	UDP-N-acetylglucosamine 2-epimerase	COG0381M	1.677419355	1.161290323	0.612903226		0.187773	0.002996	No	40.96651948	-0.33454546	1506
-	SSA_0179	hypothetical protein	-	1.902439024	1.804878049	1.12195122				No	34.49709924	0.130534351	31
-	SSA_0180	hypothetical protein	-	1.92	2	1.24				No	29.99148936	1.09787234	8
-	SSA_0181	glycosyl transferase family protein	COG1215M	2.066666667	2.555555556	1.622222222				No	33.64885321	0.180045872	1496
-	SSA_0182	endoglucanase	COG3405G	1.689655172	2.586206897	1.74137931				No	25.48663743	-0.5880117	361
-	SSA_0183	hypothetical protein	COG4699S							No	61.11587302	-0.93730159	237
comYA	SSA_0184	competence protein ComYA	COG2804NU							No	43.21118211	-0.24217252	443
comYB	SSA_0185	competence protein ComYB	COG1459NU							No	40.10781437	0.309281437	404
comYC	SSA_0186	competence protein ComYC	COG4537U							No	12.32761905	0.088571429	301
comYD	SSA_0187	competence protein ComYD	COG2165NU							No	26.49642857	-0.13714286	128
-	SSA_0188	hypothetical protein	-							No	58.20519481	-0.02077922	107

-	SSA_0189	competence protein ComGF	COG4940U							No	26.82605634	-0.48450704	133
-	SSA_0190	hypothetical protein	-							No	38.58780702	-0.82631579	94
-	SSA_0191	adenine-specific DNA methylase	COG0827L	0.375	0.208333333	0.208333333				No	38.78009346	-0.26728972	427
ackA	SSA_0192	acetate kinase	COG0282C	1.093457944	0.897196262	0.598130841	0.085358	1.335079	1.587064	No	25.7511335	-0.14937028	1526
-	SSA_0193	CAAX amino terminal protease family protein	-							No	33.45422222	1.062222222	240
-	SSA_0195	hypothetical protein	-							No	38.10146226	0.841037736	12
folP	SSA_0197	dihydropteroate synthase	COG0294H	0.822222222						Yes	36.73312303	-0.03596215	2213
-	SSA_0198	dihydrofolate synthetase	COG0285H	1.032258065	1.032258065	1.096774194				Yes	40.81185345	-0.19482759	1631
folE	SSA_0199	GTP cyclohydrolase I	COG0302H	1.010309278	1.164948454	1.092783505				Yes	28.88211957	-0.18586957	1950
folK	SSA_0200	bifunctional folate synthesis protein	COG0801H	1.087719298	1.228070175	1.210526316				Yes	45.6937037	-0.17296296	2161
-	SSA_0201	multidrug ABC transporter	COG1131V							No	41.36553191	-0.09404255	243
-	SSA_0202	hypothetical protein	-							No	32.50610687	0.688549618	28
-	SSA_0203	hypothetical protein	-							No	25.25166667	0.97875	12
nisR	SSA_0204	nisin biosynthesis two-component response transcriptional regu	COG0745TK							No	46.58280172	-0.28103448	141
nisK	SSA_0205	sensor-receptor histidine kinase NisK	COG0642T							No	49.16501449	-0.11275362	233
-	SSA_0206	hypothetical protein	-							No	29.02777778	1.277777778	3
-	SSA_0207	hypothetical protein	-							No	37.925	0.498611111	14
-	SSA_0208	hypothetical protein	COG4868S	0.99103139	0.932735426	0.923766816	0.033116	0.541506	0.130585	No	36.96720648	-0.29271255	235
pepA	SSA_0209	glutamyl aminopeptidase	COG1363G	1.18852459	1.415300546	1.418032787				No	32.44491525	-0.02288136	931
-	SSA_0210	hypothetical protein	-	0.490384615	0.288461538	0.153846154				No	35.37578947	-0.11368421	119
trxA2	SSA_0211	hypothetical protein	COG0526OC	0.598214286	0.366071429	0.205357143		0.277092		No	14.2027027	-0.33963964	554
-	SSA_0212	phenylalanyl-tRNA synthetase subunit beta	COG0073R	0.675	0.475	0.375	0.391942	0.390519		No	30.83846154	-0.12596154	457
-	SSA_0213	hypothetical protein	-							No	50.96717949	0.069230769	1
ssb2	SSA_0214	single-stranded DNA-binding protein	COG0629L							No	45.57251908	-0.42366412	505
rbsB	SSA_0215	periplasmic sugar-binding protein (ribose porter)	COG1879G	0.696969697		0.727272727	0.209051	0.816792		No	37.40338415	-0.18140244	1091
-	SSA_0216	histidine kinase	COG4585T	1	1.084337349	1.228915663				No	38.00354691	0.114874142	777
-	SSA_0217	two-component response transcriptional regulator	COG2197TK	1.091743119	1.183486239	1.403669725				No	32.49867841	0.032599119	1112
-	SSA_0218	sugar ABC transporter periplasmic protein	COG1653G	1.176	1.352	1.504				No	34.90545667	-0.29906323	421
-	SSA_0219	PTS system sugar-specific transporter subunit IIA	COG2893G	1.292035398	1.008849558	1.061946903				No	26.83517241	0.429655172	68
-	SSA_0220	PTS system mannose-specific transporter subunit IIB	COG3444G	1.457142857	1.371428571	1.7	0.176444	0.327694	0.071294	No	23.47987805	-0.32317073	70
-	SSA_0221	PTS system mannose-specific transporter subunit IIC	COG3715G	1.529032258	1.593548387	2.096774194				No	19.11925267	0.841992883	129
-	SSA_0222	PTS system mannose-specific transporter subunit IID	COG3716G	1.31557377	1.5	1.942622951				No	15.13780576	0.394964029	94
-	SSA_0224	hypothetical protein	-	1.501976285	1.66798419	2.233201581				No	25.925	0.578125	52
groES	SSA_0225	co-chaperonin GroES	COG0234O	0.643006263			0.484675	0.60199	0.653335	Yes	12.94408602	0.152688172	2430
groEL	SSA_0226	molecular chaperone GroEL	COG0459O				0.148883	0.277221	1.124138	Yes	34.86777778	0.019444444	2706
-	SSA_0227	collagen-binding surface protein	COG4932M	0.791666667	0.791666667	0.875				No	26.20779579	-0.39902755	75
-	SSA_0228	hypothetical protein	-							No	82.06744186	-0.46976744	1
-	SSA_0229	hypothetical protein	-							No	49.459375	-1	1
-	SSA_0230	hypothetical protein	COG4292S	1.333333333	1.0625	0.9375				No	32.30404199	0.66824147	87
-	SSA_0231	hypothetical protein	-	1.312280702	1.256140351	0.989473684				No	18.6951049	0.326806527	25
-	SSA_0232	hypothetical protein	-	1.463636364	1.440909091	1.313636364				No	9.62295082	1.13442623	1
-	SSA_0233	permease	COG0628R	1.459227468	1.394849785	1.141630901				No	30.13120805	1.132550336	153
-	SSA_0234	hypothetical protein	COG3247S	0.944099379	0.888198758	0.720496894				No	22.70584795	1.631578947	22
int	SSA_0235	integrase/recombinase, phage associated	COG4974L							No	41.59234829	-0.4353562	608
cshA	SSA_0236	recombination factor protein RarA	COG2256L							No	36.83009479	-0.25829384	2275
-	SSA_0238	hypothetical protein	-							No	34.86423077	-0.28653846	168
-	SSA_0239	7,8-dihydro-8-oxoguanine-triphosphatase	COG1051F							No	37.23866667	-0.29333333	196
-	SSA_0240	acetyltransferase	COG4552R							No	46.245	-0.62692308	185
prmA	SSA_0241	50S ribosomal protein L11 methyltransferase	COG2264J							No	38.57327044	-0.10503145	1938
-	SSA_0242	16S ribosomal RNA methyltransferase RsmE	COG1385S							No	35.1308	-0.0144	2127
yfkN	SSA_0243	bifunctional 2',3'-cyclic nucleotide 2'-phosphodiesterase/3'-nucl	COG0737F	0.931034483	0.793103448	0.965517241				No	24.65816936	-0.31743462	992
-	SSA_0244	hypothetical protein	-							No	35.2	-0.51923077	15
-	SSA_0245	hypothetical protein	-							No	43.31384615	-0.05692308	2
-	SSA_0246	hypothetical protein	-			0.505494505		13.24098		No	46.55046729	-0.68317757	5
-	SSA_0247	hypothetical protein	-	0.715909091					0.230961	No	36.5917226	-0.52572707	75
-	SSA_0248	hypothetical protein	-	0.710784314	0.705882353	0.808823529			0.733682	No	81.3008209	-1.39179105	1
-	SSA_0249	hypothetical protein	-	0.802973978	0.855018587	0.944237918				No	23.74893617	-0.45	3
relA	SSA_0250	GTP pyrophosphokinase	COG0317TK	0.785714286	0.7	0.714285714		9.514924		No	36.04751015	-0.50392422	2333
dtd	SSA_0251	D-tyrosyl-tRNA(Tyr) deacylase	COG1490J	0.985074627	0.902985075	0.873134328				No	35.63884354	-0.17278912	1769
-	SSA_0252	hypothetical protein	-							No	52.43509434	-0.39433962	51
-	SSA_0253	CAAX amino terminal protease family protein	-							No	29.17843137	0.904901961	30
-	SSA_0254	hypothetical protein	-							No	41.04326241	-0.54184397	84
-	SSA_0255	multiple antibiotic resistance operon transcription repressor Ma	COG1846K							No	30.1124183	-0.19477124	604
-	SSA_0256	Fe/Mn-dependent transcriptional repressor ScaR	COG1321K			0.711111111	0.008346			No	39.23302326	-0.34976744	1338
-	SSA_0257	N-acetylmuramidase/lysin	-	0.929577465	0.908450704	0.936619718				No	23.85072917	-0.55625	60
-	SSA_0258	hypothetical protein	-							No	52.53671233	-0.70082192	92
tpx	SSA_0259	thiol peroxidase	COG2077O	2.868263473	2.892215569	3.071856287	0.026572	0.05305		No	21.99375	-0.09090909	1764
ssaB	SSA_0260	Mn/Zn ABC transporter substrate-binding protein	COG0803P	1.393143691	1.886943837	2.457330416				No	37.09517799	-0.51909385	1398
ssaC	SSA_0261	Mn/Zn ABC transporter permease	COG1108P	1.378947368	1.825101215	2.377327935				No	29.48818815	1.250174216	1842
ssaA	SSA_0262	Mn/Zn ABC transporter ATPase	COG1121P	1.2	1.590909091	1.859848485				No	26.17389558	0.071084337	1328
pepO	SSA_0263	Zinc metalloproteinase in scaA 5'region	COG3590O	1.094736842	1.168421053	1.178947368				No	35.12020635	-0.40142857	458
-	SSA_0264	PEP phosphonomutase-like protein	COG0520E	3.64516129	5.677419355	5.935483871				No	41.67852349	0.095637584	146
-	SSA_0265	ORFA, transposon ISSsa1	COG2963L							No	42.98876404	-1.05617978	254
-	SSA_0266	ORFB, transposon ISSsa1	COG2801L	1.166666667	1.375	1.375				No	44.89915842	-0.68069307	733
-	SSA_0267	ROK family protein	COG1940KG	3.777777778	6.222222222	6.333333333			1.121698	No	37.35201005	-0.10929648	417
ptcB	SSA_0268	PTS system cellobiose-specific transporter subunit IIB	COG1440G		5.4	6.8				No	32.14380952	0.112380952	611
-	SSA_0269	PTS system cellobiose-specific transporter subunit IIA	COG1447G		4.428571429	5.714285714				No	37.16	-0.32571429	220
-	SSA_0270	PTS system cellobiose-specific transporter subunit IIC	COG1455G	2.333333333		5.666666667				No	30.98410596	0.774392936	308
-	SSA_0271	glycosyl hydrolase family protein	COG1874G							No	37.32692568	-0.42989865	80
-	SSA_0272	aldo/keto reductase	COG4989R							No	33.24530744	-0.17022654	1859
-	SSA_0273	hypothetical protein	COG4982I							No	39.33651805	-0.46772824	2
-	SSA_0274	hypothetical protein	COG0551L							No	38.63507692	-0.57784615	235
-	SSA_0276	glycosyl transferase family protein	COG1215M	0.711111111		0.4				No	50.04086505	-0.31107266	1331
-	SSA_0277	pyruvate formate-lyase 3-activating protein	COG1180O							No	33.89779026	-0.27827715	520
-	SSA_0278	sugar metabolism transcriptional repressor	COG1349KG	1.162162162						No	28.57368421	-0.24898785	689
-	SSA_0279	sugar-binding transcriptional repressor (DeoR)	COG2390K	1.236842105	1.342105263	1.184210526				No	34.88575851	-0.22631579	923
-	SSA_0281	hypothetical protein	-	1.322580645						No	18.98301887	0.726415094	21
-	SSA_0282	PTS system cellobiose-specific transporter subunit IIA	COG1447G							No	55.3271028	-0.10934579	201
-	SSA_0283	PTS system cellobiose-specific transporter subunit IIB	COG1440G							No	19.14271845	0.410679612	144
ptcC	SSA_0284	PTS system cellobiose-specific transporter subunit IIC	COG1455G							No	31.79630485	0.923556582	600
-	SSA_0285	formate acetyltransferase 3	COG1882C							No	36.43471095	-0.34907749	425

-	SSA_0286	fructose-6-phosphate aldolase	COG0176G				0.112623	0.268889		No	23.70855856	0.010810811	1192
gldA	SSA_0287	glycerol dehydrogenase	COG0371C							No	33.11434066	0.052747253	990
-	SSA_0288	hypothetical protein	COG2020O	0.423076923	0.230769231	0.230769231				No	23.45080214	0.45026738	367
leuS	SSA_0289	leucyl-tRNA synthetase	COG0495J	0.902857143	0.834285714					Yes	31.99989247	-0.40011947	2667
-	SSA_0290	hypothetical protein	COG4292S							No	28.74	0.678740157	202
-	SSA_0291	short chain dehydrogenase	COG0300R							No	29.11818182	-0.07127273	1684
-	SSA_0292	AraC family transcriptional regulator	COG2207K							No	27.38487654	-0.13240741	646
-	SSA_0293	hypothetical protein	COG2008E	1.19047619	1.30952381	1.714285714				No	5.463529412	-0.06235294	226
-	SSA_0294	hypothetical protein	COG2008E	1.259259259	1.185185185	1.222222222				No	40.07647059	-0.15	3
mleR	SSA_0295	LysR family transcriptional regulator	COG0583K							No	41.92721649	-0.19381443	1046
-	SSA_0296	XRE family transcriptional regulator	COG3620K							No	45.96206897	-0.60603448	608
mleS	SSA_0297	malate dehydrogenase	COG0281C							No	25.09817006	-0.19796673	1514
-	SSA_0298	malate permease	COG0679R							No	35.6551312	0.82303207	663
-	SSA_0299	hypothetical protein	COG1814S	1.173076923	1.141025641	0.955128205				No	28.26347305	0.423353293	585
-	SSA_0300	hypothetical protein	-	1.797101449	2.405797101	2.47826087				No	23.94630841	-0.38504673	125
-	SSA_0301	hypothetical protein	-	1.598540146	2.328467153	2.96350365				No	22.79219895	-0.38115183	17
pgk	SSA_0302	phosphoglycerate kinase	COG0126G	1.246621622	1.397522523	1.356981982	0.274229	0.542975	0.249353	Yes	17.65203518	-0.03467337	2691
sspC	SSA_0303	surface protein C	COG1196D	1.003184713	0.939490446					No	27.25652722	-0.53313413	71
-	SSA_0304	cell wall degradation protein	COG3942R	0.318584071	0.203539823	0.185840708				No	14.72217195	-0.23800905	117
-	SSA_0305	hypothetical protein	COG4129S	1.288461538	1.346153846					No	31.58683908	0.621264368	173
glnR	SSA_0306	transcription regulator (glutamine synthetase repressor)	COG0789K	0.776699029		0.430420712				No	61.94297521	-0.87603306	890
glnA	SSA_0307	glutamine synthetase	COG0174E				3.106557	0.330065	0.183925	No	42.22477679	-0.23258929	2433
-	SSA_0308	hypothetical protein	COG0456R							No	45.62597122	-0.37194245	184
-	SSA_0309	glyoxalase/bleomycin resistance protein/dioxygenase	-							No	59.61877698	-0.14316547	132
-	SSA_0310	hypothetical protein	COG1309K			0.64				No	34.74461538	-0.34051282	767
-	SSA_0311	hypothetical protein	COG4335L			0.607142857				No	49.90846774	-0.47903226	224
-	SSA_0312	metallo-beta-lactamase superfamily hydrolase	COG0595R	1.190930788	1.23150358	1.205250597	0.247899	0.295159		Yes	36.01035714	4.76E-17	1686
-	SSA_0313	hypothetical protein	COG5503S	1.040322581		0.596774194				No	70.64618421	-0.85657895	402
wcaG	SSA_0314	nucleoside-diphosphate-sugar epimerase	COG0451MG							No	33.132	-0.34	676
-	SSA_0315	MarR family transcriptional regulator	COG1846K	0.68115942		0.391304348				No	55.65006494	-0.25519481	634
-	SSA_0316	hypothetical protein	COG1214O	1	0.963855422	0.855421687				No	46.91802632	0.111403509	2280
rimI	SSA_0317	ribosomal protein alanine acetyl transferase	COG0456R	1.2	1.12	1.12				No	53.3354	-0.19133333	1693
gcp	SSA_0318	DNA-binding/iron metalloprotein/AP endonuclease	COG0533O	1.1	1.25	1.24				No	33.60119048	0.096428571	2742
-	SSA_0319	branched-chain amino acid permease	COG1296E	1.218181818	1.472727273	1.563636364				No	34.61212121	0.851948052	1388
-	SSA_0320	hypothetical protein	COG4392S	1.319587629	1.525773196	1.608247423				No	26.42053571	1.154464286	331
ubiE	SSA_0321	methylase	COG2226H							No	31.42903226	-0.2983871	1439
-	SSA_0322	TipA family transcription regulator	COG0789K							No	46.50530612	-0.55265306	1746
-	SSA_0323	flavoprotein	COG2081R							No	30.03173913	-0.11841432	1554
-	SSA_0324	hypothetical protein	-	0.67768595		0.396694215	0.054941	0.262743	0.183417	No	47.43963415	-0.75548781	143
ugpQ	SSA_0325	membrane-anchored glycerophosphoryl diester phosphodiester	COG4781C	1.027027027	0.972972973	1.027027027				No	29.31609589	0.243835616	744
-	SSA_0326	hypothetical protein	COG0718S	0.890710383	0.825136612	0.754098361				No	26.67272727	-0.31616162	1954
-	SSA_0327	glycosyltransferase	-		0.257142857	0.2		0.76843		No	33.3096	-0.536	10
pepXP	SSA_0328	x-prolyl-dipeptidyl aminopeptidase	COG2936R	1	0.953488372	1.023255814				No	35.11708661	-0.5492126	355
-	SSA_0329	glycerol uptake facilitator/aquaporin protein	COG0580G	0.513605442	0.275510204	0.210884354				No	24.2448505	0.488039867	264
-	SSA_0330	hypothetical protein	COG3247S							No	25.45550351	0.166978923	15
cppA	SSA_0331	C3-degrading proteinase	-	0.6		0.384615385				No	52.37012295	-0.34344262	133
-	SSA_0332	hypothetical protein	COG1680V	0.730769231						No	36.19810289	-0.28424437	791
mvaK1	SSA_0333	mevalonate kinase	COG1577I	0.972222222	1	0.986111111				Yes	31.70650685	0.159589041	559
mvaD	SSA_0334	diphosphomevalonate decarboxylase	COG3407I	1.11	1.24	1.37				Yes	45.60606349	-0.36190476	488
mvaK2	SSA_0335	phosphomevalonate kinase	COG1577I	1.162162162	1.306306306	1.45045045				Yes	30.69469027	-0.00678466	355
-	SSA_0336	isopentenyl pyrophosphate isomerase	COG1304C	1.194174757	1.378640777	1.40776699				Yes	44.4498806	-0.28746269	962
mvaA	SSA_0337	hydroxymethylglutaryl-CoA reductase	COG1257I	1.163934426	1.155737705	1.213114754				Yes	29.46629717	-0.08254717	540
mvaS	SSA_0338	hydroxymethylglutaryl-CoA synthase	COG3425I	0.831168831		0.792207792				Yes	24.42372449	-0.21887755	622
-	SSA_0339	hypothetical protein	COG3610S	0.962962963	0.851851852					No	39.65548387	1.218064516	414
-	SSA_0341	hypothetical protein	COG2966S		0.6	0.533333333				No	34.66360465	0.36627907	904
-	SSA_0342	pyruvate formate-lyase	COG1882C	6.455108359	12.09287926	15.42724458	0.181519	1	0.352326	No	34.72529183	-0.41971466	905
dinP	SSA_0343	DNA polymerase IV	COG0389L							No	25.75762712	-0.37655367	2036
-	SSA_0345	hypothetical protein	COG2966S	0.928571429	1.238095238	1.571428571			1.408489	No	51.50277778	-0.55925926	2
-	SSA_0346	hypothetical protein	-	1.387096774	1.935483871	2.451612903				No	38.71102273	0.593560606	30
-	SSA_0348	CAAX amino protease	-	1.470588235	1.911764706	2.382352941				No	36.22258065	0.666935484	32
-	SSA_0349	TetR/AcrR family transcriptional regulator	COG1309K	1.12962963	1.259259259	1.351851852				No	35.81745283	-0.36698113	1406
recD	SSA_0350	helicase	COG0507L	1.15	1.225	1.425				No	33.4502665	-0.29302031	1827
spi	SSA_0351	Signal peptidase I	COG0681U	1.00952381	0.923809524	0.819047619				No	19.92296651	-0.32631579	1738
rnhC	SSA_0352	ribonuclease HIII	COG1039L	1.011764706	0.858823529	0.658823529				No	42.66618012	-0.15403727	580
-	SSA_0353	hypothetical protein	-							No	45.12395833	-0.72291667	138
-	SSA_0354	hypothetical protein	COG1286R	1.14	1.03	0.92				No	34.85699454	0.878142077	332
mutS2	SSA_0355	DNA mismatch repair protein	COG1193L	1.086956522	1.101449275	1.101449275				No	45.05303732	-0.35675676	1066
-	SSA_0356	dipeptidase	COG4690E	1.296296296	1.716049383	2.086419753				No	35.57586864	-0.56355932	308
-	SSA_0357	thioredoxin	COG3118O	1.254794521	1.347945205	1.098630137	0.373823	0.525822	0.654449	No	15.71634615	0.030769231	2054
-	SSA_0358	hypothetical protein	-							No	44.07639053	-0.38047337	117
-	SSA_0359	LysR family transcriptional regulator	-							No	54.77036232	-0.28478261	7
-	SSA_0360	hypothetical protein	-	0.762711864						No	51.81044444	-0.34296296	43
-	SSA_0362	thioredoxin	COG3118O							No	17.20945946	-0.18378378	2
dagA	SSA_0363	D-alanine/glycine/Na permease	COG1115E							No	27.35977528	0.883820225	1453
dctA	SSA_0364	serine/threonine transporter SstT	COG3633E							No	28.24666667	0.746376812	1837
-	SSA_0365	small-conductance mechanosensitive efflux channel	COG0668M	0.843137255		0.588235294				No	28.6697561	0.360278746	2310
-	SSA_0366	hypothetical protein	-	1						No	33.05432692	-0.35096154	26
-	SSA_0367	hypothetical protein	COG5504O							No	42.09833333	-0.22133333	156
-	SSA_0368	hypothetical protein	-							No	39.71311475	-0.8852459	12
-	SSA_0369	NADP-specific glutamate dehydrogenase	COG0334E	1.17057903	1.211267606	1.361502347				No	19.94912281	0.287719298	1
-	SSA_0370	acetyltransferase	COG1246E	1.113172542	1.209647495	1.471243043				No	39.10372671	-0.33043478	963
gdhA	SSA_0371	glutamate dehydrogenase	COG0334E	0.973577236	1.028455285	1.152439024				No	31.77948661	-0.2234375	1851
pyrDA	SSA_0373	dihydroorotate dehydrogenase 1A	COG0167F	1	0.971830986	0.845070423				No	36.27407051	-0.11730769	511
-	SSA_0374	peptide methionine sulfoxide reductase msrA/msrB	COG0229O	1.593220339	1.677966102	1.711864407				No	43.2022508	-0.54437299	1105
-	SSA_0375	lipoprotein transporter	COG1464P	0.714285714		0.309791332				No	29.92172414	-0.46551724	1590
-	SSA_0376	methionine ABC transporter ATPase	COG1135P	0.847058824		0.470588235				No	29.57749296	-0.07830986	1579
-	SSA_0377	methionine ABC transporter permease	COG2011P	0.879828326	0.699570815	0.557939914				No	18.93130435	0.943043478	1573
-	SSA_0378	chlorohydrolase	COG0402FR							No	35.67210402	-0.09007092	1497
-	SSA_0379	PTS system beta-glucoside-specific transporter subunit EII	COG1263G	15	37	63.5				No	21.98771384	0.625660964	680
-	SSA_0380	enzyme of poly-gamma-glutamate biosynthesis (capsule format	COG2843M							No	35.69978261	-0.45173913	720
-	SSA_0381	hypothetical protein	-							No	20.16780269	-0.11838565	1

-	SSA_0382	AraC family transcriptional regulator	COG4977K							No	32.05037931	-0.47241379	1491
bglA	SSA_0383	Beta-glucosidase	COG2723G							No	24.86438413	-0.37682672	505
-	SSA_0384	hypothetical protein	COG5279D							No	38.58519041	-0.63836389	236
opuAb	SSA_0385	glycine/betaine ABC transporter permease	COG4176E	0.976	0.804	0.864				No	25.86637631	0.179616725	978
opuAa	SSA_0386	glycine-betaine ABC transporter ATPase component	COG4175E		0.392638037	0.423312883				No	32.00656566	-0.29671717	1107
-	SSA_0387	GntR family transcriptional regulator	COG0490P		0.307692308	0.230769231				No	40.41971831	-0.31455399	329
-	SSA_0388	mismatch repair ATPase	COG0249L							No	42.76530142	-0.04609929	248
-	SSA_0389	hypothetical protein	COG0561R							No	48.09242754	-0.09891304	421
-	SSA_0390	hypothetical protein	COG2755E							No	37.77619048	-0.17761905	405
spxB	SSA_0391	pyruvate oxidase	COG0028EH	4.061538462	4.184615385	4.553846154				No	28.18291032	-0.25262267	816
-	SSA_0392	hypothetical protein	COG2764S							No	30.2913913	0.00173913	140
-	SSA_0393	bacteriocin ABC transporter permease/ATP-binding protein	COG1132V	0.9375	0.90625	0.953125				No	30.01244275	0.123091603	95
-	SSA_0394	hypothetical protein	-	0.834586466						No	30.94130435	0.548913043	96
arb	SSA_0395	6-phospho-beta-glucosidase	COG2723G							No	26.01989562	-0.43820459	403
-	SSA_0396	hypothetical protein	COG0596R							No	28.90638095	-0.17809524	367
-	SSA_0397	hypothetical protein	-							No	32.48737557	0.561538462	26
-	SSA_0398	hypothetical protein	COG1680V							No	31.14057432	-0.13006757	145
-	SSA_0400	hypothetical protein	COG1680V							No	28.48686341	-0.17403035	90
-	SSA_0401	two-component response transcriptional regulator	COG0745TK	1	1.044117647	1.044117647				No	47.75	-0.28628319	142
-	SSA_0402	histidine kinase	COG0642T	1.061538462	1.123076923	1.2				No	41.66773196	-0.10137457	77
-	SSA_0403	CAAX amino protease	-							No	40.71530686	0.499638989	67
-	SSA_0405	XRE family transcriptional regulator	COG1396K		0.533333333	0.466666667				No	41.41809756	0.43902439	322
-	SSA_0406	hypothetical protein	-							No	18.465	0.789166667	3
-	SSA_0407	multidrug ABC transporter ATPase	COG1131V							No	27.54967742	-0.27612903	463
-	SSA_0408	hypothetical protein	COG0474P							No	29.12649351	0.977922078	2
-	SSA_0409	multidrug ABC transporter ATPase	COG1131V							No	23.8205298	-0.2705298	284
-	SSA_0410	hypothetical protein	-			2.583333333				No	41.21617391	0.90173913	2
-	SSA_0411	hypothetical protein	-							No	23.23936508	0.891666667	38
-	SSA_0412	multidrug ABC transporter ATPase	COG1131V							No	37.23245033	-0.14536424	156
pabB	SSA_0413	anthranilate/para-aminobenzoate synthases component I/ chori	COG0147EH							No	37.46893543	-0.48184991	750
-	SSA_0414	hypothetical protein	-							No	57.46512195	0.704065041	4
-	SSA_0415	permease	COG0628R							No	38.44416667	0.881388889	422
metE	SSA_0416	5-methyltetrahydropteroyltriglutamate/homocysteine S-methyltra	COG0620E							No	33.79973333	-0.22626667	1520
metF	SSA_0417	5,10-methylenetetrahydrofolate reductase	COG0685E							No	48.51886986	-0.13116438	1441
-	SSA_0418	AraC family transcriptional regulator	COG2207K	0.59375	0.5	0.40625				No	42.48017731	-0.28510638	760
-	SSA_0419	alpha-galactosidase	COG3345G							No	37.22339597	-0.42778524	472
-	SSA_0420	HAD superfamily hydrolase	COG0561R							No	26.01841935	-0.15774194	161
-	SSA_0421	phosphoglycerate mutase family protein	COG0406G							No	31.5980303	-0.53383838	316
-	SSA_0422	phosphoglycerate mutase family protein	COG0406G		1.526315789	1.578947368				No	29.56005	-0.351	267
-	SSA_0423	hypothetical protein	-							No	42.31132075	0.273584906	35
-	SSA_0424	exopolysaccharide biosynthesis protein	COG4632G							No	22.01419142	-0.16468647	383
-	SSA_0425	glycosyltransferase	COG0463M							No	19.22180516	0.237535817	456
-	SSA_0426	hypothetical protein	COG2339S	1.647058824	1.529411765	1.647058824				No	26.19531136	0.668864469	111
-	SSA_0427	SARP family transcriptional regulator	COG3899R		1.444444444	1.722222222				No	46.89271739	-0.34426878	265
hutG	SSA_0428	formimidoylglutamase	COG0010E	0.823529412	0.773109244					No	31.25912929	-0.11794195	1351
hutH	SSA_0429	histidine ammonia-lyase	COG2986E							No	36.6505814	-0.08391473	1026
-	SSA_0430	cationic amino acid transporter	COG0531E							No	21.82868889	0.867777778	1043
-	SSA_0431	hypothetical protein	COG3758S							No	44.21407035	-0.29296482	99
-	SSA_0432	formate--tetrahydrofolate ligase	COG2759F							No	29.75547576	0.046499102	518
-	SSA_0433	methenyltetrahydrofolate cyclohydrolase	COG3404E	4	18	58				No	36.04951923	-0.04134615	256
-	SSA_0434	glutamate formiminotransferase	COG3643E	6	31	83				No	38.41973244	-0.18227425	156
hutU	SSA_0435	urocanate hydratase	COG2987E							No	32.22011834	-0.33949704	953
hutI	SSA_0436	imidazolonepropionase	COG1228Q	1.208333333	1.541666667	1.875				No	29.66745843	-0.07553444	1124
rpsF	SSA_0437	30S ribosomal protein S6	COG0360J	0.486744433	0.268292683	0.191940615	0.712	0.785563	0.354854	Yes	55.79375	-0.334375	2407
ssb	SSA_0438	single-stranded DNA-binding protein	COG0629L	0.56855792	0.302600473	0.256501182				Yes	30.27012195	-0.69085366	2157
rpsR	SSA_0440	30S ribosomal protein S18	COG0238J	0.623467601		0.334500876	0.385594	0.765152	0.745235	Yes	55.77468354	-0.74556962	2551
-	SSA_0441	hypothetical protein	COG1309K						0.884619	No	51.12406863	-0.38235294	745
-	SSA_0442	multidrug ABC transporter ATPase	COG1131V							No	24.92447257	-0.10464135	729
-	SSA_0443	ABC transporter permease	-							No	37.60972015	0.845149254	64
-	SSA_0445	hypothetical protein	-							No	35.35652174	0.61884058	2
-	SSA_0446	hypothetical protein	COG4858S	0.744897959						No	38.43763393	0.125446429	258
-	SSA_0447	magnesium/cobalt transporter CorA	COG0598P	0.423529412	0.247058824	0.247058824				No	54.94493631	0.013375796	990
uvrA	SSA_0448	excinuclease ABC subunit A	COG0178L	1.253731343	1.268656716	1.313432836				No	35.79788971	-0.26914104	2590
pepP	SSA_0449	aminopeptidase	COG0006E	0.857142857	0.746031746	0.587301587				No	30.99235127	0.004815864	2283
efp	SSA_0450	elongation factor P	COG0231J	0.688		0.408		2.520229		No	24.99413978	-0.21236559	2588
-	SSA_0451	hypothetical protein	COG1302S	0.823529412		0.532085561				No	27.40542636	0.258914729	458
nusB	SSA_0452	transcription antitermination protein NusB	COG0781K	0.878698225	0.715976331		0.351532	0.107215		No	38.57635135	-0.19797297	2395
-	SSA_0453	Type II secretory pathway, pullulanase PulA glycosidase	COG1523G							No	36.75418491	-0.56918086	742
scrR	SSA_0454	lactose operon transcriptional repressor, LacI family	COG1609K							No	31.45922118	-0.06978193	1072
scrB	SSA_0455	sucrose-6-phosphate hydrolase	COG1621G							No	31.78188017	-0.48450413	658
scrA	SSA_0456	phosphotransferase system IIC components, glucose/maltose/N	COG1263G							No	20.28492138	0.497327044	392
scrK	SSA_0457	fructokinase	COG1940KG	1	1	0.763636364				No	21.38628763	-0.05451505	379
-	SSA_0458	peptide deformylase	COG0242J	1.065217391		0.695652174				No	38.96617647	-0.09779412	700
-	SSA_0459	hypothetical protein	-	0.939655172	0.801724138	0.547413793				No	30.27297297	1.342342342	21
-	SSA_0460	multiple antibiotic resistance operon transcription repressor Ma	COG1846K	0.53164557	0.379746835	0.329113924				No	48.93296053	-0.50197368	397
-	SSA_0461	multidrug ABC transporter ATPase/permease	COG1132V	0.962790698	0.860465116	0.823255814				No	30.23539116	0.170748299	320
-	SSA_0462	multidrug ABC transporter ATPase/permease	COG1132V	1.102766798	1.102766798	1.059288538				No	27.02396581	0.258803419	249
-	SSA_0463	cobyrinic acid a,c-diamide synthase	COG1797H	1.033333333	1.333333333	1.5			0.178272	No	26.18411504	-0.20951327	1083
-	SSA_0464	cobalamin biosynthesis protein cobD	COG1270H	1.147058824	1.5	1.676470588				No	34.47661392	0.422151899	1175
cbiC	SSA_0465	cobalt-precorrin-8X methylmutase	COG2082H			1.689655172	0.030476	0.126722		No	46.70344828	-0.00775862	906
cbiC	SSA_0466	cobalt-precorrin-8X methylmutase	COG2082H			1.696969697				No	33.92307692	-0.0491453	60
cbiD	SSA_0467	cobalt-precorrin-6A synthase	COG1903H	0.976190476	1.261904762	1.452380952		3.890624		No	30.06773333	-0.06293333	707
-	SSA_0468	cobalt-precorrin-6Y C(5)-methyltransferase	COG2241H			1.372093023				No	32.6447619	0.014761905	301
cobL	SSA_0469	cobalt-precorrin-6Y C(15)-methyltransferase	COG2242H							No	32.63333333	-0.25185185	1004
cobM	SSA_0470	precorrin-4 methylase	COG2875H	0.93220339	1.084745763	1.169491525				No	41.62299611	-0.1766537	991
-	SSA_0471	cobalamin biosynthesis protein CbiG	COG2073H	0.9	1.05	1.133333333				No	33.6673297	0.052043597	456
-	SSA_0472	precorrin-3B C(17)-methyltransferase	COG1010H	0.892857143	1.071428571	1.178571429				No	26.21701245	0.101244813	973
-	SSA_0473	precorrin-6x reductase	COG2099H	0.90625	1.03125	1.125				No	31.30562249	0.092771084	733
-	SSA_0474	uroporphyrin-III C-methyltransferase	COG0007H	0.98630137	1.232876712	1.328767123				No	31.1874739	-0.04968685	1765
-	SSA_0475	CbiK protein	COG4822H	0.978947368	1.2	1.252631579				No	49.47215686	-0.30470588	262
-	SSA_0476	cobalt-precorrin-2 C(20)-methyltransferase	COG2243H	1	1.155339806	1.223300971				No	33.27042918	0.077253219	815

cbiM	SSA_0477	cobalt ABC transporter ATP-binding protein	COG0310P	1.064102564	1.307692308	1.320512821				No	20.44538462	0.893076923	544
cbiN	SSA_0478	cobalt transport protein cbiN	COG1930P	1.142857143	1.37755102	1.540816327				No	45.59613861	-0.0960396	319
-	SSA_0479	CbiQ protein	COG0619P		0.458333333	0.333333333				No	38.93446429	0.547767857	290
-	SSA_0480	cobalt ABC transporter ATP-binding protein	COG1122P			0.68				No	33.5199631	-0.24059041	337
-	SSA_0481	cobyric acid synthase	COG1492H	0.972972973	1.081081081	1.027027027				No	38.73066132	-0.24308617	1233
-	SSA_0482	ATP:cobalamin adenosyl transferase	COG2096S							No	48.25968586	-0.48115183	558
-	SSA_0483	siroheme synthase	COG1648H							No	44.3713986	-0.11118881	539
-	SSA_0484	glutamyl-tRNA reductase	COG0373H	1.024390244	1.219512195	1.487804878				No	35.15635492	-0.21654676	1888
-	SSA_0485	porphobilinogen deaminase	COG0181H	0.966101695	1.152542373	1.372881356				No	39.77712418	-0.15359477	2168
hemD	SSA_0486	uroporphyrinogen-III synthase	COG1587H	1.069767442	1.255813953	1.372093023				No	50.36431535	-0.23360996	332
-	SSA_0487	delta-aminolevulinic acid dehydratase	COG0113H	1	1.170212766	1.212765957	0.107369	4.066229		No	40.88086687	-0.12972136	2155
-	SSA_0488	glutamate-1-semialdehyde 2,1-aminomutase	COG0001H	0.97826087	1.152173913	1.217391304				No	33.92314815	-0.03402778	1981
cobU	SSA_0489	adenosylcobinamide kinase	COG2087H		1.166666667	1.314814815				No	43.82525773	-0.3314433	1143
-	SSA_0490	cobalamin 5'-phosphate synthase	COG0368H	0.962264151	1.169811321	1.377358491				No	28.91285124	0.788429752	1095
-	SSA_0491	alpha-ribazole-5'-phosphate phosphatase	COG0406G	1	1.19047619	1.46031746				No	43.49163158	-0.37894737	1070
-	SSA_0492	NADH-dependent flavin oxidoreductase	COG1902C	1.020408163	1.285714286	1.428571429				No	43.95395095	-0.30572207	1495
-	SSA_0493	peptide ABC transporter periplasmic protein	COG0747E	1.037735849	1.245283019	1.471698113				No	21.46086106	-0.37123288	633
-	SSA_0494	peptide ABC transporter ATPase	COG0444EP	0.891304348	1.130434783	1.304347826				No	41.70192308	-0.05615385	11
-	SSA_0495	peptide ABC transporter ATPase	COG1123R	0.911111111	1.088888889	1.288888889				No	32.29882813	-0.28945313	33
-	SSA_0496	succinylglutamate desuccinylase/aspartoacylase family protein	COG3608R	0.9375	1.104166667	1.333333333				No	37.26269113	-0.1733945	326
-	SSA_0497	nickel ABC transporter	COG0601EP	0.948717949	1.128205128	1.435897436				No	29.77438066	0.692749245	318
-	SSA_0498	peptide ABC transporter permease	COG1173EP	0.956521739	1.217391304	1.5				No	43.35167286	0.726394052	695
-	SSA_0499	peptide ABC transporter periplasmic protein	COG0747E	1.044444444	1.288888889	1.711111111				No	21.19317269	-0.72730924	1949
-	SSA_0500	peptide ABC transporter, permease protein	COG0601EP							No	40.33769968	0.561022364	2153
-	SSA_0502	peptide ABC transporter, permease protein	COG1173EP							No	37.19344978	0.770742358	1500
-	SSA_0503	peptide ABC transporter, ATP-binding protein	COG0444EP							No	56.85931559	-0.13802281	47
amiF	SSA_0504	peptide ABC transporter, ATP-binding protein	COG1124EP							No	33.11822034	-0.01525424	203
-	SSA_0505	hypothetical protein	COG1853R			1.44				No	42.80366492	-0.33036649	385
-	SSA_0506	(Fe-S)-binding protein	COG1600C							No	37.85614035	-0.23684211	1419
-	SSA_0507	hypothetical protein	-	0.7	0.575	0.425				No	29.55967136	0.950704225	1
-	SSA_0508	hypothetical protein	-	5	11.63636364	14.36363636				No	32.66757991	-0.5890411	171
-	SSA_0509	propanediol utilization protein	COG4917E	3.727272727	9.363636364	14.36363636				No	31.87020833	-0.19166667	265
-	SSA_0510	L-threonine-O-3-phosphate decarboxylase	COG0079E	1.846153846	3.846153846	5.846153846				No	58.52258242	-0.21675824	857
pduX	SSA_0511	propanediol utilization kinase	COG4542Q	1.518518519	2.814814815	4.37037037				No	51.98298611	-0.21979167	223
-	SSA_0512	nicotinate-nucleotide--dimethylbenzimidazole phosphoribosyltra	COG2038H	1.482758621	2.448275862	4		0.900205		No	25.92853261	-0.00842391	1077
-	SSA_0513	ATP:cob(I)alamin adenosyltransferase	COG2096S	1.36	2	3.16				No	37.50703518	-0.38442211	704
-	SSA_0514	PduQ protein	COG1454C	4.133333333	8.866666667	12.36666667				No	32.27236842	-0.01684211	989
-	SSA_0515	propanediol utilization protein PduU	COG4810E	1.579617834	2.031847134	2.038216561				No	25.01481481	0.621296296	271
-	SSA_0516	two-component response regulator	COG3707T	1.677419355	2.329032258	2.464516129				No	32.43560209	0.017277487	741
-	SSA_0517	sensor histidine kinase	COG3920T	1.814285714	2.542857143	2.957142857				No	45.37719665	-0.38054393	659
eutA	SSA_0518	reactivating factor for ethanolamine ammonia lyase	COG4819E	4.709677419	9.967741935	12.12903226				No	28.56310273	0.070230608	227
-	SSA_0519	ethanolamine ammonia-lyase large subunit	COG4303E	2.872727273	6.854545455	11.58181818				No	25.73815789	-0.20460526	551
-	SSA_0520	ethanolamine ammonia-lyase small subunit	COG4302E	2.173076923	5.326923077	9.711538462				No	38.18954248	-0.22418301	542
eutL	SSA_0521	ethanolamine utilization protein EutL	COG4816E	1.627118644	3.694915254	6.474576271				No	33.03479452	0.200913242	217
-	SSA_0522	ethanolamine utilization protein	COG4577QC	1.517241379	3.379310345	6.827586207				No	37.78609626	-0.27272727	54
-	SSA_0523	aldehyde dehydrogenase	COG1012C	1.276923077	2.415384615	4.430769231	0.28891	1.141242	0.061496	No	39.38965447	-0.02337398	232
-	SSA_0524	microcompartment protein	COG4577QC	1.094594595	1.959459459	3.472972973	0.32664	0.681159	0.063905	No	10.15698925	0.466666667	203
-	SSA_0525	microcompartment protein	COG4577QC	1.032258065	1.752688172	3.129032258	0.099443	0.264387	0.063855	No	8.043956044	0.573626374	288
-	SSA_0526	hypothetical protein	COG4812E	1.074626866	1.686567164	3.104477612	0.681986			No	36.72676056	-0.34507042	5
pduL	SSA_0527	propanediol utilization protein PduL	COG4869Q	0.977011494	1.436781609	2.540229885				No	43.34759615	-0.2875	305
-	SSA_0528	hypothetical protein	COG0647G	1.048387097	1.435483871	2.403225806				No	45.07865116	-0.24697674	54
-	SSA_0529	ethanolamine utilization protein	COG4576QC	1.027777778		2.277777778				No	20.89139785	-0.02903226	376
eutH	SSA_0530	ethanolamine transporter	COG3192E	0.948051948	1.272727273	1.74025974				No	14.81895604	1.06510989	267
eutQ	SSA_0531	ethanolamine utilization protein EutQ	COG4766E	1.011363636	1.227272727	1.715909091				No	25.00939597	-0.22416107	287
pduB	SSA_0532	propanediol utilization protein PduB	COG4816E							No	31.04529915	0.082051282	165
pduC	SSA_0533	glycerol dehydratase large subunit pduC	COG4909Q							No	32.85696203	-0.26618445	157
-	SSA_0535	propanediol utilization: dehydratase, medium subunit	-							No	40.37882883	-0.2509009	145
-	SSA_0536	B12-dependent diol dehydratase small subunit	COG4910Q							No	39.44269006	-0.56374269	157
-	SSA_0537	propanediol utilization:dioldehydratase reactivation	COG0248FP							No	22.55679612	0.166990291	151
pduH	SSA_0538	PduH protein	-							No	33.08095238	0.014285714	139
pduO	SSA_0539	PduO protein	COG3193R							No	46.85815217	-0.18967391	274
-	SSA_0540	glycerol uptake facilitator protein	COG0580G							No	17.7249789	0.824894515	1068
-	SSA_0541	acetate kinase	COG0282C							No	30.94160401	-0.00300752	623
secA	SSA_0543	preprotein translocase subunit SecA	COG0653U				0.543841	0.668991	0.501244	Yes	39.94816448	-0.5147795	2586
aroG	SSA_0544	phospho-2-dehydro-3-deoxyheptonate aldolase	COG0722E	0.981099656	1.056701031	1.180412371	0.439086	1.117057	0.254931	No	33.16909621	-0.44548105	843
aro	SSA_0546	phospho-2-dehydro-3-deoxyheptonate aldolase	COG0722E	1.010666667	1.144	1.28				No	37.07437318	-0.44956268	776
acpS	SSA_0547	4'-phosphopantetheinyl transferase	COG0736I	1.108974359	1.378205128	1.602564103				Yes	47.48833333	-0.22583333	1772
alr	SSA_0548	alanine racemase	COG0787M	1.053254438	1.325443787	1.550295858				Yes	26.99864499	0.089159892	2280
recG	SSA_0549	ATP-dependent DNA helicase RecG	COG1200LK	1.078212291	1.396648045	1.553072626				No	43.30090909	-0.21549926	2243
ansB	SSA_0551	L-asparaginase	COG0252EJ	0.84	0.76		0.272274			No	24.6021875	0.0371875	1767
-	SSA_0552	Cof family protein	COG4696S	0.764705882		0.482352941				No	41.76344086	-0.40129032	409
-	SSA_0553	hypothetical protein	-							No	30.75603448	-0.37068966	7
-	SSA_0554	hypothetical protein	-		0.3	0.266666667				No	43.00782609	-1.20434783	2
-	SSA_0555	hypothetical protein	-							No	43.22607204	-0.64511149	89
-	SSA_0556	hypothetical protein	-							No	43.73048	-0.4488	2
-	SSA_0557	hypothetical protein	-	0.724137931						No	28.22262774	-0.79489051	1
-	SSA_0558	hypothetical protein	-	0.741935484						No	24.95673077	-0.39519231	18
-	SSA_0559	hypothetical protein	-	0.84375						No	51.62888	-0.2112	15
-	SSA_0560	hypothetical protein	-	0.903225806	0.806451613	0.935483871				No	49.91390244	-0.10569106	1
-	SSA_0561	RNA:NAD 2'-phosphotransferase	COG1859J				0.623729			No	41.20342541	-0.58121547	333
-	SSA_0562	hypothetical protein	-	1.114285714	1.028571429	1.085714286			8.917017	No	59.10300971	-0.6631068	1
-	SSA_0563	universal stress protein	COG0589T	2.725609756	3.859756098	4.603658537				No	30.706	-0.16533333	1475
-	SSA_0564	aminotransferase	COG0436E	0.692810458		0.388888889				No	23.48217822	-0.2039604	1081
-	SSA_0565	hypothetical protein	COG5271R	0.951219512		0.609756098	0.415211	0.504489		No	31.3060511	-0.63449477	59
codY	SSA_0566	CodY family transcriptional regulator	COG4465K	0.843373494	0.807228916	0.56626506				No	40.69541985	-0.20381679	487
-	SSA_0567	isochorismitase family protein	COG1335Q	0.903225806	0.88172043	0.817204301				No	28.48491892	-0.10378378	1620
aspS	SSA_0568	aspartyl-tRNA synthetase	COG0173J	0.885714286	0.742857143	0.628571429				No	41.08081315	-0.38512111	43
gatC	SSA_0569	aspartyl/glutamyl-tRNA amidotransferase subunit C	COG0721J	0.978723404		0.70212766	0.165074	0.498775	0.262892	Yes	45.642	-0.355	1718
gatA	SSA_0570	aspartyl/glutamyl-tRNA amidotransferase subunit A	COG0154J	1.075757576	1.060606061	1				Yes	36.07459016	-0.13053279	2400
gatB	SSA_0571	aspartyl/glutamyl-tRNA amidotransferase subunit B	COG0064J	1.120879121	1.148351648	1.137362637		0.571245		Yes	44.80463312	-0.46415094	2251
-	SSA_0572	dehydrogenase	COG1063ER	1.65060241	2.481927711	2.759036145	0.273105	1.439538	0.070444	No	32.05632184	0.09137931	1449

-	SSA_0573	hypothetical protein	-							No	17.9	-0.53421053	7
-	SSA_0574	hypothetical protein	COG0398S							No	36.41875	0.741826923	642
-	SSA_0575	HAD superfamily hydrolase	COG2179R	0.566037736	0.339622642	0.283018868				Yes	16.0112426	-0.35857988	689
-	SSA_0576	GTP-binding protein YqeH	COG1161R	0.767123288		0.438356164				No	30.50464674	-0.23967391	496
-	SSA_0577	hypothetical protein	COG1534J	0.884615385		0.58974359				No	35.95728155	-0.40582524	1298
nadD	SSA_0578	nicotinic acid mononucleotide adenylyltransferase	COG1057H	0.976190476	0.738095238	0.630952381				No	38.89142857	-0.22571429	2199
-	SSA_0579	HAD superfamily hydrolase	COG1713H	0.920792079	0.683168317	0.524752475		0.61899		No	37.70301508	-0.38492462	753
-	SSA_0580	isochorismatase family protein	COG1335Q	0.920634921		0.571428571				No	34.74377246	-0.34610778	731
-	SSA_0581	hypothetical protein	COG0799S	0.855263158		0.473684211				No	41.5553719	0.011570248	2377
-	SSA_0582	methylase	COG2227H							No	37.14877049	-0.34508197	1499
-	SSA_0583	hypothetical protein	COG1323R	1.021276596	1.170212766	1.234042553				No	30.07447802	-0.18104396	634
-	SSA_0584	hypothetical protein	-							No	29.22213483	-0.70561798	7
-	SSA_0585	hypothetical protein	-							No	35.70123596	-0.53033708	1
-	SSA_0586	hypothetical protein	COG0217S							No	16.46302521	-0.35210084	2520
hipO1	SSA_0587	metal-dependent amidase/aminoacylase/carboxypeptidase	COG1473R	1.137931034	1.275862069	1.310344828				No	37.41610526	-0.065	1539
-	SSA_0588	L-cystine ABC transporter substrate-binding component	COG0834ET	0.440898345						No	33.94	-0.57454546	929
dapE	SSA_0589	acetylornithine deacetylase	COG0624E	0.782467532			0.311785	0.340605	0.526533	No	50.96482609	-0.32804348	1208
-	SSA_0590	hypothetical protein	-	0.97338403	0.91634981	0.904942966				No	39.12761421	0.734517766	141
-	SSA_0591	hypothetical protein	COG1912S	0.944606414	0.833819242	0.87755102	0.682562	0.03248	0.070292	No	27.44911032	-0.13274021	700
-	SSA_0592	hypothetical protein	COG4720S	1.029498525	0.973451327	0.908554572				No	26.30222222	0.734444444	288
-	SSA_0593	hypothetical protein	COG1054R	1.7	1.425	1.25				No	29.30426829	-0.58323171	1444
-	SSA_0594	AraC family transcriptional regulator	COG2207K							No	59.14419476	-0.49550562	357
-	SSA_0595	hypothetical protein	COG2764S							No	38.00963504	-0.26861314	712
-	SSA_0596	hypothetical protein	-							No	41.88319328	-0.37394958	78
-	SSA_0597	hypothetical protein	-							No	42.5662623	-0.29803279	40
-	SSA_0599	hypothetical protein	-							No	34.05	0.654651163	15
-	SSA_0601	phosphorylase Pnp/Udp family protein	COG2820F							No	38.18501976	-0.00434783	309
-	SSA_0602	cobalt ABC transporter ATPase	COG1122P	1.045454545	1.113636364	1.181818182				No	31.83869875	-0.21871658	795
-	SSA_0603	cobalt ABC transporter	COG0619P							No	31.69863309	0.463669065	423
-	SSA_0604	hypothetical protein	-							No	43.70594771	-0.28300654	3
gidB	SSA_0605	16S rRNA methyltransferase GidB	COG0357M							No	38.23050633	-0.29451477	2361
-	SSA_0606	peptide ABC transporter ATPase	COG1136V				0.830623			No	39.73643725	-0.16882591	85
-	SSA_0607	ABC transporter permease	COG3127Q							No	35.6416581	0.121722365	56
mefE	SSA_0608	macrolide-efflux protein	-							No	26.77751756	0.886651054	908
-	SSA_0609	TetR/AcrR family transcriptional regulator	COG1309K							No	48.78243902	-0.21804878	914
lemA	SSA_0610	LemA-like protein	COG1704S	1.094594595	1.18018018	1.207207207				No	29.4010582	-0.10529101	1265
htpX	SSA_0611	heat shock protein HtpX	COG0501O	1.099378882	1.136645963	1.093167702				No	46.11748322	0.286577181	1832
rgg	SSA_0612	Rgg protein	COG1426S							No	36.66969283	-0.1559727	145
gtfP	SSA_0613	glucosyltransferase	COG5263R							No	22.6625	-0.67735969	111
-	SSA_0614	transporter	-							No	37.90823245	0.810169492	96
rggD	SSA_0615	RggD	-							No	33.24224913	-0.32352941	57
-	SSA_0616	hypothetical protein	COG1399R	0.4375	0.25	0.125				No	58.76704545	-0.38011364	312
-	SSA_0617	membrane protease subunit	COG2268S	0.787878788			0.561055	1.122951		No	36.33069106	-0.43963415	580
-	SSA_0618	hypothetical protein	-			2				No	55.17948454	-0.87216495	39
-	SSA_0620	hypothetical protein	-			1.722222222				No	41.29919355	-0.47096774	127
-	SSA_0621	SOS responce UmuC protein	COG0389L							No	39.02721868	-0.29639066	497
-	SSA_0622	XRE family transcriptional regulator	COG2932K							No	33.77511111	-0.45333333	324
-	SSA_0623	hypothetical protein	-							No	43.22981481	0.12654321	6
-	SSA_0624	hypothetical protein	COG0742L	0.563636364	0.272727273	0.218181818				No	41.52072626	-0.19832402	2497
coaD	SSA_0625	phosphopantetheine adenylyltransferase	COG0669H	0.75	0.533333333	0.433333333				Yes	39.2970122	-0.31158537	2350
-	SSA_0626	hypothetical protein	COG3480T	0.839622642						No	29.52596542	-0.26599424	1321
-	SSA_0627	hypothetical protein	COG4470S	0.846846847	0.882882883	0.810810811				No	64.8938342	-1.52435233	420
-	SSA_0628	ribosomal RNA large subunit methyltransferase N	COG0820R	0.929824561	1.005847953	0.970760234				No	38.73621547	-0.37486188	2171
-	SSA_0629	hypothetical protein	COG4767V	0.958083832	1.011976048	1				No	28.32793296	0.41452514	195
trpB2	SSA_0631	tryptophan synthase subunit beta	COG0133E							No	30.75549618	-0.18931298	164
trpE	SSA_0632	anthranilate synthase component I	COG0147EH							No	40.52900442	-0.27323009	2148
trpG	SSA_0633	anthranilate synthase component II	COG0512EH							No	46.34812834	-0.13796791	2036
trpD	SSA_0634	anthranilate phosphoribosyltransferase	COG0547E							No	33.96170659	0.014670659	2116
trpC	SSA_0635	indole-3-glycerol phosphate synthase	COG0134E							No	34.39960784	-0.07058824	2126
trpF	SSA_0636	N-(5'-phosphoribosyl)anthranilate isomerase	COG0135E							No	39.27734807	0.004972376	1454
trpB	SSA_0637	tryptophan synthase subunit beta	COG0133E							No	30.23199017	-0.22579853	2171
trpA	SSA_0638	tryptophan synthase subunit alpha	COG0159E							No	24.59150579	0.077992278	2194
-	SSA_0639	hypothetical protein	-							No	14.88245614	1.152631579	1
-	SSA_0640	hypothetical protein	COG2378K							No	20.6458194	-0.30769231	147
-	SSA_0641	hypothetical protein	COG3708S							No	50.97053691	-0.26845638	5
pilD	SSA_0642	Type 4 prepilin peptidase	COG1989NOU							No	35.23686364	1.072272727	178
-	SSA_0643	hypothetical protein	-							No	12.57	0.255	1
dps	SSA_0644	DNA protection protein	COG0783P	2.274068869	3.11243851	3.557273366				No	29.31403509	-0.21929825	1648
-	SSA_0646	hypothetical protein	COG4483S	0.581818182		0.363636364				No	49.64861111	-0.36111111	347
-	SSA_0647	hypothetical protein	COG0607P	0.5859375		0.328125				No	32.04444444	-0.20079365	1005
-	SSA_0648	hypothetical protein	-	0.654135338	0.458646617	0.315789474				No	23.76666667	1.085333333	45
-	SSA_0649	ribosomal small subunit pseudouridine synthase A	COG1187J							No	34.34564315	-0.40539419	132
typA	SSA_0650	GTP-binding protein TypA	COG1217T	0.774058577				0.423913		No	36.8399696	-0.23069909	2306
-	SSA_0651	hypothetical protein	-	1.043715847	0.819672131	0.737704918				No	24.67539326	1.096629213	118
murD	SSA_0652	UDP-N-acetylmuramoyl-L-alanyl-D-glutamate synthetase	COG0771M	0.785714286						Yes	27.86424444	-0.02688889	2453
murG	SSA_0653	undecaprenyldiphospho-muramoylpentapeptide beta-N- acetylg	COG0707M	0.958333333	0.916666667	0.84375				Yes	38.09019663	-0.07134832	2444
divIB	SSA_0654	cell division protein DivIB	COG1589M	0.942528736	0.816091954	0.747126437				No	64.13042394	-0.79201995	437
ftsA	SSA_0655	cell division protein FtsA	COG0849D	0.994285714	0.862857143		0.238474	1.411392	0.479058	No	39.51283186	-0.07566372	1941
ftsZ	SSA_0656	cell division protein FtsZ	COG0206D	1.06805293	0.950850662		0.267969	0.734973	0.596965	Yes	36.48588235	-0.17694118	2509
-	SSA_0657	pyridoxal 5'-phosphate dependent enzymes class III	COG0325R	1.066860465	0.933139535					No	41.54753363	-0.39686099	2193
-	SSA_0658	hypothetical protein	COG1799S	0.979166667	0.8671875					No	62.28140704	-0.74221106	810
-	SSA_0659	hypothetical protein	COG2302S	1	0.88172043					No	41.69467433	-0.2605364	636
divIVA	SSA_0660	cell division protein DivIVA	COG3599D	1.062893082	1.171907757	1.102725367	0.278874	0.795049	0.022782	No	52.72840909	-0.73484849	646
ileS	SSA_0661	isoleucyl-tRNA synthetase	COG0060J	1.305164319	1.384976526	1.427230047				Yes	35.93337634	-0.28688172	2730
-	SSA_0662	multiple antibiotic resistance operon transcription repressor Ma	COG1846K							No	44.93112583	-0.40331126	540
-	SSA_0663	acetyltransferase	COG2153R							No	56.93474576	-0.38898305	985
-	SSA_0664	hypothetical protein	-	1.230529595	1.208722741	1.124610592				No	45.813	-0.146	172
-	SSA_0665	MutT/NUDIX family NTP pyrophosphohydrolase	COG0494LR		0.431818182	0.340909091	1.114085	1.200136	0.046828	No	27.35114094	-0.64899329	731
-	SSA_0666	hypothetical protein	-							No	61.60885714	-0.84285714	1
-	SSA_0667	transcriptional regulator	COG2378K							No	36.20468227	-0.30334448	620
-	SSA_0668	hypothetical protein	COG4832S							No	39.65593137	-0.52254902	262

clpE	SSA_0669	ATP dependent protease	COG0542O	1.452631579	1.505263158	1.494736842		0.79137		No	39.22832011	-0.45238095	1420
-	SSA_0670	hypothetical protein	COG4703S	0.774137931	0.618965517	0.203448276				No	31.51973684	-0.16710526	259
folD	SSA_0671	bifunctional 5,10-methylene-tetrahydrofolate dehydrogenase/ 5,	COG0190H		0.558823529	0.441176471				No	34.82922535	-0.10140845	2596
-	SSA_0672	hypothetical protein	COG0561R			0.576923077				No	42.39381323	-0.19922179	112
-	SSA_0673	hypothetical protein	COG0063G							No	36.64035714	0.059642857	1924
xseA	SSA_0674	exodeoxyribonuclease VII large subunit	COG1570L	0.692307692	0.5	0.403846154				No	44.90807175	-0.37757848	2328
xseB	SSA_0675	exodeoxyribonuclease VII small subunit	COG1722L	0.794871795		0.602564103				No	21.05714286	-0.55714286	1462
ispA	SSA_0676	farnesyl diphosphate synthase	COG0142H	0.907692308	0.846153846	0.8				No	36.35089347	-0.03264605	2624
hlyA	SSA_0677	rRNA methylase	COG1189J	0.961038961	0.948051948	0.909090909				No	21.04649446	-0.16125461	1439
ahrC	SSA_0678	ArgR family transcriptional regulator	COG1438K	1.1	1.1375	1.1875				No	43.29300699	-0.11888112	204
recN	SSA_0679	DNA repair and genetic recombination	COG0497L	1.151162791	1.197674419	1.26744186				No	35.27755435	-0.34764493	2275
pphA	SSA_0680	serine/threonine protein phosphatase	COG0639T	1.103448276	1.172413793	1.172413793				No	33.21822314	-0.33305785	991
-	SSA_0682	hypothetical protein	COG1307S	0.712643678		0.425287356				No	34.00577617	-0.04693141	491
-	SSA_0683	DNA-binding protein HU	COG0776L	0.842688843			0.609223	0.6914	0.60596	Yes	16.18901099	-0.30879121	2328
-	SSA_0684	fibril-like structure subunit FibA	-							No	21.39867243	-0.53220738	5
-	SSA_0685	hypothetical protein	-							No	22.02924528	1.240566038	14
fur	SSA_0686	Fe2+/Zn2+ uptake regulation protein	COG0735P	1.406779661		0.762711864				No	28.72244898	-0.45238095	2111
-	SSA_0687	hypothetical protein	-	1	1.043010753	0.903225806				No	45.0959919	0.234008097	1
gpmA	SSA_0688	phosphoglyceromutase	COG0588G	0.783517835			0.333178	1.22479	0.547126	Yes	34.20782609	-0.55130435	1580
pbp2b	SSA_0689	penicillin-binding protein 2B	COG0768M	0.797468354						No	25.24377907	-0.36656977	1498
recR	SSA_0690	recombination protein RecR	COG0353L	0.962962963	0.814814815					No	34.31823232	-0.02121212	2464
ddl	SSA_0691	D-alanyl-alanine synthetase A	COG1181M	0.539325843	0.337078652	0.235955056				Yes	39.84084986	-0.08583569	2361
murF	SSA_0692	D-Ala-D-Ala adding enzyme	COG0770M	0.75			0.667939	0.627635	0.181782	Yes	25.79517544	-0.1872807	2444
mutT	SSA_0694	Mutator protein	COG1051F	0.89010989	0.846153846	0.802197802				No	49.86359606	-0.39162562	698
-	SSA_0695	hypothetical protein	COG5522S	1.1	1.1	1.128571429				No	37.56807018	0.676315789	105
-	SSA_0696	hypothetical protein	-	1.267605634	1.352112676	1.507042254				No	44.33162281	0.572368421	43
-	SSA_0697	hypothetical protein	-							No	43.5	0.997297297	1
prfC	SSA_0698	peptide chain release factor 3	COG4108J	0.84375						No	38.91285992	-0.4303502	1618
-	SSA_0699	methyltransferase	-							No	30.78658537	-0.72317073	7
-	SSA_0700	hypothetical protein	-	1.056179775	1.04494382	1.101123596				No	29.17251969	0.861417323	15
-	SSA_0701	cation transporter	COG0598P	1.235294118	1.117647059	1.019607843				No	33.33311258	-0.13543046	243
citB	SSA_0702	aconitate hydratase	COG1048C							No	35.10024803	-0.19064262	1745
-	SSA_0703	citrate synthase	COG0372C							No	48.87153226	-0.19865591	2058
-	SSA_0704	isocitrate dehydrogenase	COG0538C							No	32.27839196	-0.08567839	1379
-	SSA_0705	hypothetical protein	-		0.36	0.16				No	36.14022727	0.540909091	18
rheA	SSA_0706	RNA helicase	COG0513LKJ	1.23255814	0.88372093	0.674418605	0.416161	0.484167		No	37.77971311	-0.65655738	2391
-	SSA_0707	lactose phosphotransferase system transcriptional repressor	COG1349KG	0.991304348	1.113043478	1.417391304				No	29.09322581	-0.31693548	746
-	SSA_0708	hypothetical protein	COG1755S	1.192307692		0.884615385				No	29.64355932	0.750847458	387
-	SSA_0709	hypothetical protein	COG2827L	1.322580645	1.806451613	2.64516129				No	31.14137931	-0.77471264	1146
epsH	SSA_0710	exopolysaccharide biosynthesis acetyltransferase	COG0110R	1.175	1.5	2.05				No	43.60659722	-0.27430556	226
-	SSA_0711	hypothetical protein	COG4123R		1.678571429	2.107142857				No	31.975	-0.30282258	1064
ctpE	SSA_0712	P-type ATPase-metal cation transport	COG0474P	1	0.909090909	0.954545455				No	31.88139923	0.319640565	387
plsC	SSA_0713	1-acyl-sn-glycerol-3-phosphate acyltransferase	COG0204I		0.472222222	0.277777778				Yes	43.20613821	-0.06341463	1372
-	SSA_0714	hypothetical protein	-							No	50.76628571	0.877714286	45
comEA	SSA_0715	DNA uptake protein	COG1555L							No	20.32876106	-0.39911504	1595
comEC	SSA_0716	competence protein	COG2333R							No	41.83030831	0.230428954	1558
-	SSA_0718	hypothetical protein	COG3152S							No	65.62307692	-0.39203297	308
holA	SSA_0720	DNA polymerase III subunit delta	COG1466L	0.894736842	0.736842105	0.596491228				Yes	32.54396552	-0.15143678	736
sodA	SSA_0721	Mn/Fe-dependent superoxide dismutase	COG0605P	2.161458333	2.221354167	2.1875		0.97287		No	33.38905473	-0.25820896	2276
-	SSA_0722	hypothetical protein	COG2367V	0.742857143		0.371428571				No	34.89976684	-0.35362694	72
-	SSA_0723	hypothetical protein	-	1.314285714	1.257142857	0.857142857				No	18.44782609	0.176086957	1
-	SSA_0724	multidrug ABC transporter ATPase/permease	COG1132V							No	30.03005703	0.013307985	103
-	SSA_0725	hypothetical protein	-							No	31.4621118	1.065838509	1
-	SSA_0726	FmtA-like protein	COG1680V							No	19.47801336	-0.14974958	392
-	SSA_0727	metal-dependent membrane protease	COG1266R							No	34.1460219	0.756569343	109
-	SSA_0728	protease	COG4449R							No	38.07330677	0.769322709	231
-	SSA_0729	hypothetical protein	-							No	34.9469697	0.46969697	1
-	SSA_0730	arsenical resistance operon repressor ArsR	COG0640K	1.096153846		0.75				No	45.16741573	-0.3258427	1648
-	SSA_0731	hypothetical protein	-	1.144927536	1.231884058	1.202898551				No	56.72846154	0.882840237	3
-	SSA_0732	transposase	-							No	27.15901639	0.163934426	1
-	SSA_0733	hypothetical protein	COG4377S	0.817391304	0.895652174	0.860869565				No	37.50007634	0.825954198	170
-	SSA_0734	arsenical resistance operon repressor ArsR	COG0640K							No	48.10410959	-0.26575343	130
-	SSA_0735	hypothetical protein	-	1	1.065789474	1.105263158				No	24.11460606	0.744848485	102
-	SSA_0736	catabolite gene activator and regulatory subunit of cAMP-depen	COG0664T	1	1.011363636	0.875				No	40.39210526	-0.29692983	1189
sagP	SSA_0737	arginine deiminase	COG2235E	7.516666667	14.28333333	15.38888889	0.176189	0.372935	0.282803	No	39.18508557	-0.37726161	866
arc	SSA_0738	ornithine carbamoyltransferase	COG0078E	4.847826087	11.72463768	16.88405797				No	28.37988166	-0.22307692	2266
arcC	SSA_0739	carbamate kinase	COG0549E	7.625	22.375	34.125				No	30.25431746	-0.07269841	815
-	SSA_0740	C4-dicarboxylate anaerobic carrier, arginine transporter	COG1288S	5.038461538	13.34615385	23				No	24.73343936	0.813916501	662
arcT	SSA_0741	hypothetical protein	COG0624E	2.461538462	5.769230769	9.923076923				No	33.45235955	-0.12808989	1095
argR	SSA_0743	transcriptional repressor (arginine synthesis)	COG1438K	1.076086957	0.945652174	0.913043478				No	56.23724359	-0.09038462	782
queA	SSA_0744	S-adenosylmethionine--tRNA ribosyltransferase-isomerase	COG0809J	0.957746479	0.76056338	0.647887324				No	24.97809942	-0.16871345	2025
-	SSA_0745	hypothetical protein	-							No	37.80273973	1.393150685	1
nagB	SSA_0746	glucosamine-6-phosphate deaminase	COG0363G							No	33.84982979	-0.23446809	1366
dacA	SSA_0747	DD-carboxypeptidase	COG1686M	0.653846154	0.403846154	0.288461538				No	30.99359202	-0.4059867	732
-	SSA_0748	major facilitator superfamily sugar transporter	COG2814G							No	18.36277635	0.838560411	392
coiA	SSA_0749	competence protein	COG4469R							No	49.42857595	-0.4335443	414
-	SSA_0750	hypothetical protein	-							No	29.26953125	-0.21328125	50
-	SSA_0751	oligoendopeptidase F	COG1164E	1.298013245	1.291390728	1.258278146				No	38.37653333	-0.49866667	1162
-	SSA_0752	hypothetical protein	COG4122R	1.322404372	1.37704918	1.316939891	0.237113	1.35693		No	42.6277533	-0.16651982	1314
prsA	SSA_0753	foldase protein PrsA	COG0760O	1.05026455	0.944444444	0.822751323		0.25228		No	27.4540597	-0.53343284	713
-	SSA_0755	hypothetical protein	COG4894S	0.909836066	0.81147541	0.606557377				No	29.92180124	-0.24658385	202
alaS	SSA_0756	alanyl-tRNA synthetase	COG0013J	1.085714286	1.1	1.023809524				Yes	36.29989679	-0.28027523	2747
argC	SSA_0757	N-acetyl-gamma-glutamyl-phosphate reductase	COG0002E							No	34.17647059	-0.06264706	2089
argJ	SSA_0758	bifunctional ornithine acetyltransferase/N-acetylglutamate synth	COG1364E							No	33.03979849	0.058690176	1445
argB	SSA_0759	acetylglutamate kinase	COG0548E							No	23.90897959	0.264081633	2013
-	SSA_0760	acetylornithine aminotransferase	COG4992E							No	34.60105263	0.018421053	2273
-	SSA_0761	XRE family transcriptional regulator	COG1476K							No	31.99428571	-0.22285714	744
-	SSA_0762	hypothetical protein	-							No	40.87795276	0.453149606	92
-	SSA_0763	transcription repressor	-							No	34.01722222	0.703333333	96
-	SSA_0765	hypothetical protein	COG1266R							No	45.42103004	0.78583691	206
-	SSA_0766	hypothetical protein	-							No	29.03317972	0.956682028	181

-	SSA_0767	diacylglycerol kinase catalytic subunit	COG1597IR							No	30.65901695	-0.14135593	846
nrdF	SSA_0768	ribonucleotide-diphosphate reductase subunit beta	COG0208F	1.349544073	1.142857143	1.17325228	0.426017	0.460737	0.630547	Yes	27.95708464	-0.3984326	2020
-	SSA_0769	hypothetical protein	-							No	52.45861111	-0.63333333	1
nrdE	SSA_0770	ribonucleotide-diphosphate reductase subunit alpha	COG0209F	1.035087719	0.75		0.401353	0.791165	0.065575	Yes	35.41421419	-0.3286509	2450
nrdH	SSA_0771	glutaredoxin-like protein	COG0695O	0.986206897	0.582758621	0.194827586	0.400136	0.588302	0.503819	Yes	24.34166667	-0.44722222	761
ptsH	SSA_0772	phosphocarrier protein HPr	COG1925G	1.258972943	1.324130315	1.244064053	0.254144	1.309179	0.384615	No	15.58045977	0.128735632	1836
ptsI	SSA_0773	PTS enzyme I	COG1080G	1.099315068	1.092465753	1.036986301	5.19885	2.287805	0.593219	No	26.7440208	-0.13275563	2177
gapN	SSA_0774	NADP-dependent glyceraldehyde-3-phosphate dehydrogenase	COG1012C	0.737704918		0.31147541	0.321677	0.467826	0.217942	No	30.23185654	0.047257384	2288
glgB	SSA_0775	glycogen branching protein	COG0296G	4.318181818	8.409090909	10.90909091				No	45.84863208	-0.51996855	1585
glgC	SSA_0776	glucose-1-phosphate adenylyltransferase	COG0448G	2.78125	6.1875	10.4375				No	39.29315789	-0.25736842	1639
glgD	SSA_0777	glycogen biosynthesis protein GlgD	COG0448G	1.974358974	4.307692308	7.358974359				No	41.21638522	-0.3292876	268
glgA	SSA_0778	glycogen synthase	COG0297G	1.536585366	2.975609756	4.951219512				No	35.43113445	-0.13088235	1719
glgP	SSA_0779	glycogen phosphorylase	COG0058G	1.244444444	1.577777778	2	0.47848	1.116585		No	32.20929825	-0.35225564	1298
-	SSA_0780	acid phosphatase	-							No	32.87536145	0.690361446	146
pmi	SSA_0781	mannose-6-phosphate isomerase	COG1482G	1.064516129	1.021505376	0.88172043				No	35.27383387	-0.25271566	833
uncE	SSA_0782	ATP synthase F0F1 subunit C	COG0636C	0.758349705	0.677799607	0.36935167				Yes	55.27272727	1.168181818	502
uncB	SSA_0783	ATP synthase F0F1 subunit A	COG0356C	0.957317073	0.829268293					Yes	25.12731092	0.521008403	2225
uncF	SSA_0784	ATP synthase F0F1 subunit B	COG0711C	1.03087886	0.986539984	0.873317498				Yes	32.75426829	-0.09390244	1727
uncH	SSA_0785	ATP synthase F0F1 subunit delta	COG0712C	1.032397408	0.953563715	0.813174946				Yes	52.97252809	-0.30898876	794
uncA	SSA_0786	proton-translocating ATPase, F1 sector subunit alpha	COG0056C	0.974188177	0.964196503		0.35931	1.638037	0.35575	Yes	38.42711454	-0.02422908	2379
uncG	SSA_0787	ATP synthase F0F1 subunit gamma	COG0224C	1.015933232	1.036418816					Yes	22.85836177	-0.23515358	2371
uncD	SSA_0788	ATP synthase F0F1 subunit beta	COG0055C	1.039522744	1.127516779	1.070096943	1.386651	0.373684	0.139202	Yes	37.07008547	-0.07628205	2543
atpC	SSA_0789	ATP synthase F0F1 subunit epsilon	COG0355C	1.081306018	1.202944942	1.212548015	1.165182	0.554109		Yes	38.00928571	-0.22142857	1936
-	SSA_0790	hypothetical protein	-							No	8.103947368	1.152631579	146
murA	SSA_0791	UDP-N-acetylglucosamine 1-carboxyvinyltransferase	COG0766M	0.871794872	0.769230769					No	36.3915493	-0.07159624	2205
-	SSA_0792	hypothetical protein	-	0.903225806	0.870967742					No	31.21	0.778333333	107
endA	SSA_0793	DNA-entry nuclease	-	1.097560976	1.048780488	0.926829268				No	33.10734266	-0.5972028	174
-	SSA_0794	Zn-dependent protease	COG5549O							No	26.16870293	-0.28661088	149
-	SSA_0795	hypothetical protein	-							No	41.43479339	0.930578512	39
uup	SSA_0796	ABC transporter ATPase	COG0488R	0.744186047	0.697674419					No	42.01537242	-0.48399366	1622
-	SSA_0797	transcriptional regulator	-	0.975						No	32.65334495	-0.38675958	104
-	SSA_0798	ABC transporter permease	COG2814G	1.069767442	1.023255814	1.11627907				No	30.66819338	0.689058524	637
-	SSA_0799	hypothetical protein	COG5416S							No	24.23294118	0.802352941	29
cobQ	SSA_0800	glutamine amidotransferase	COG3442R	1.139240506	1.037974684	1.075949367				Yes	27.56015326	-0.46934866	702
-	SSA_0801	Mur ligase family protein	COG0769M	0.954545455	0.833333333	0.772727273				Yes	32.15727069	-0.05592841	707
-	SSA_0802	hypothetical protein	COG1624S	0.692307692		0.346153846				No	35.83829787	0.358156028	1097
-	SSA_0803	hypothetical protein	COG4856S	0.773584906		0.424528302				No	32.38685259	-0.21155379	410
glmM	SSA_0804	phosphoglucosamine mutase	COG1109G	0.933333333	0.76		0.009648	0.024687		Yes	22.99311111	-0.142	2558
-	SSA_0805	collagen-binding surface protein	COG4932M	1.106951872	1.188948307					No	15.89462366	-0.51487455	28
-	SSA_0806	hypothetical protein	-	0.590163934	0.360655738	0.098360656				No	-3.007142857	1.069047619	81
obgE	SSA_0807	GTPase ObgE	COG0536R	0.81300813		0.536585366				Yes	31.86766055	-0.33279817	2652
-	SSA_0808	hypothetical protein	-				6.97205			No	27.59622642	-0.95471698	129
-	SSA_0809	hypothetical protein	COG0251J	0.647058824	0.5	0.426470588				No	41.8468254	0.226190476	2141
-	SSA_0810	hypothetical protein	COG1660R	0.85	0.7875	0.8				No	45.42469595	-0.3847973	1726
-	SSA_0811	hypothetical protein	COG0391S	0.928	0.976	1.04				No	31.85510769	-0.12338462	1299
-	SSA_0812	hypothetical protein	COG1481S	1.009009009	1.09009009	1.189189189				No	38.21716172	-0.24752475	968
-	SSA_0813	thioredoxin reductase	COG0492O	0.680555556	0.402777778	0.291666667				No	40.46586957	-0.14503106	814
-	SSA_0814	pyridine nucleotide-disulfide oxidoreductase	COG1249C	1.476190476	1.632653061	1.707482993				No	38.23561644	0.038812785	639
-	SSA_0815	hypothetical protein	COG3759S	1.882352941		0.882352941				No	42.29754237	1.108474576	470
-	SSA_0816	copper transport operon or penicillinase transcription repressor	COG3682K		1.608695652					No	41.5928	-0.5528	265
-	SSA_0817	antirepressor regulating drug resistance	COG4219KT	1.202898551	1.536231884	1.753623188				No	44.93014433	-0.07463918	287
-	SSA_0818	SPX domain-containing protein	COG5036P							No	25.5399177	-0.73662551	80
-	SSA_0819	hypothetical protein	COG1292M							No	26.79079295	0.518942731	169
rpsU	SSA_0820	30S ribosomal protein S21	-	0.761645193	0.755698712					No	74.61224138	-1.46724138	1838
mscL	SSA_0822	large conductance mechano-sensitive ion channel	COG1970M	1.323076923	0.723076923	0.384615385				No	21.0023622	0.533070866	1738
dnaG	SSA_0824	DNA primase	COG0358L	0.914473684	0.723684211					Yes	41.3061794	-0.46976744	2619
rpoD	SSA_0825	RNA polymerase sigma factor RpoD	COG0568K	1.027472527	0.912087912	0.857142857	0.445952	0.723259	0.424286	Yes	44.85378378	-0.51405405	2595
-	SSA_0826	hypothetical protein	COG2151R	1.06	0.988	0.852				No	48.13125	-0.16428571	1281
-	SSA_0827	hypothetical protein	-	0.989361702	1.010638298	0.617021277				No	40.75594982	-0.60860215	94
srpA	SSA_0829	platelet-binding glycoprotein	COG5422	1.033033033	1.036036036					No	27.14130462	0.030646154	14
-	SSA_0830	glycosyltransferase	COG1442M	0.820512821	0.807692308					No	43.35199255	-0.23836127	579
-	SSA_0831	hypothetical protein	COG0438M	0.87826087	0.982608696	1.043478261				No	47.42730539	-0.12335329	102
secY	SSA_0832	preprotein translocase subunit SecY	COG0201U	0.942622951	1.147540984	1.262295082				No	42.99461916	0.736117936	87
-	SSA_0833	accessory secretory protein Asp1	-	0.938596491	1.157894737	1.377192982				No	45.30288973	-0.4730038	111
-	SSA_0834	accessory secretory protein Asp2	-	0.895522388	1.089552239	1.253731343				No	38.04583497	-0.39410609	110
-	SSA_0835	accessory secretory protein Asp3	-	1.019607843	1.254901961	1.666666667				No	43.6836478	-0.41761006	110
secA2	SSA_0836	preprotein translocase subunit SecA	COG0653U	0.941935484	1.129032258	1.4				No	38.46508197	-0.3776797	97
-	SSA_0837	glucosyltransferase	COG0438M	1.01242236	1.254658385	1.583850932				No	46.80363636	-0.33675889	984
-	SSA_0838	GtfB	-	1.068571429	1.314285714	1.782857143				No	48.35592841	-0.33489933	110
-	SSA_0839	hypothetical protein	-		1.49137931	1.681034483				No	25.11183333	0.118333333	17
-	SSA_0841	hypothetical protein	-	1.225433526	1.526011561	1.930635838				No	22.9439726	1.04109589	13
-	SSA_0842	effector of murein hydrolase LrgA/holin-like protein	COG1380R							No	25.61666667	1.273015873	795
-	SSA_0843	hypothetical protein	COG1346M	0.766666667						No	19.62478992	1.01092437	1109
-	SSA_0844	hypothetical protein	-	1.076923077	1.012820513	1.025641026				No	27.97487365	0.913718412	157
-	SSA_0845	multidrug ABC transporter ATPase	COG1131V	0.666666667		0.333333333				No	50.45880851	-0.22553192	660
dnaE	SSA_0846	DNA polymerase III DnaE	COG0587L	1.145833333	1.541666667	1.916666667				Yes	36.6262956	-0.30767072	2581
pfk	SSA_0847	6-phosphofructokinase	COG0205G				0.303351	0.59115	0.620767	Yes	35.24017857	-0.02380952	1880
pykF	SSA_0848	pyruvate kinase	COG0469G	0.902949572			0.428358	0.245802	0.585915	Yes	19.73013972	-0.23592814	2455
sip	SSA_0849	Signal peptidase I	COG0681U	1.163636364	1.4	1.4				No	37.36324324	-0.34108108	220
-	SSA_0850	ubiquitin C-terminal hydrolase	-							No	37.95951417	-0.59311741	9
-	SSA_0851	cation efflux family protein	COG0053P	0.904761905	0.880952381	0.904761905				No	35.39021531	0.014114833	1963
pcrA	SSA_0852	ATP-dependent DNA helicase	COG0210L	0.921568627	0.823529412	0.843137255				No	41.8706168	-0.46456693	2633
-	SSA_0854	hypothetical protein	COG0494LR	1.022727273	0.988636364					No	49.79090909	-0.28636364	580
cysD	SSA_0855	O-acetylhomoserine sulfhydrylase	COG2873E							No	24.23920188	0.012441315	1541
gtrB	SSA_0856	glycosyl transferase family protein	COG0463M	0.80952381		0.404761905				No	34.83931818	0.123051948	1731
-	SSA_0857	hypothetical protein	-	1.027027027		0.459459459				No	43.49292383	0.763882064	34
rmlD	SSA_0858	dTDP-L-rhamnose synthase	COG1091M	1.02739726	0.767123288	0.575342466				No	24.93105634	-0.50739437	1590
tpiA	SSA_0859	triosephosphate isomerase	COG0149G	0.934900542	0.839059675		0.144384	0.35394	0.079689	Yes	24.92322835	0.017322835	2521
-	SSA_0860	N-acetylmuramidase/lysin	COG3757M	0.964157706						No	26.40401633	-0.66436526	270
murN	SSA_0861	hypothetical protein	COG2348V	1.320754717	1.641509434	2.254716981				No	41.79158151	-0.56690998	224
murM	SSA_0862	MurM protein	COG2348V	1.259259259	1.611111111	2.064814815				Yes	38.26750611	-0.54327628	301

-	SSA_0863	Cof family protein	COG0561R	1.271604938	1.530864198	1.740740741				No	36.53799257	-0.12973978	1150
-	SSA_0864	hypothetical protein	COG1078R	1.257575758	1.393939394	1.363636364				No	41.06055427	-0.38406467	913
-	SSA_0865	hypothetical protein	COG4506S							No	26.02238462	-0.45076923	134
pacL	SSA_0866	cation transporter E1-E2 family ATPase	COG0474P	1.555555556	1.746031746	1.587301587				No	32.35056399	0.171041215	1338
-	SSA_0867	hypothetical protein	-							No	54.9608365	-0.4418251	182
-	SSA_0868	hypothetical protein	COG0420L							No	43.20699301	-0.51783217	218
prfB	SSA_0869	peptide chain release factor 2	COG1186J	0.952941176	0.788235294	0.570588235				Yes	36.224	-0.56307692	2404
ftsE	SSA_0870	cell division protein FtsE	COG2884D	1.046511628	0.976744186	0.841860465	0.655285	0.404556	0.272077	Yes	39.07565217	-0.31652174	1675
ftsX	SSA_0871	cell division protein FtsX	COG2177D	1.186046512	1.097674419	1.041860465				Yes	29.69285714	0.227922078	1487
-	SSA_0872	Zn-dependent hydrolases, including glyoxylases	COG0491R	0.859375		0.34375				No	39.3434434	-0.16415094	2193
dinG	SSA_0873	ATP-dependent DNA helicase; DNA polymerase III, epsilon sub	COG1199KL	0.966101695	0.881355932	0.762711864				No	42.022543	-0.2495086	1622
-	SSA_0874	hypothetical protein	COG2855S	1.16	1.173333333	1.026666667				No	35.50184049	0.974846626	1369
rodA	SSA_0875	rod shape determining protein (cell-cycle protein)	COG0772D	1.217948718	1.384615385	1.397435897				No	33.432	0.734146341	1900
thiJ	SSA_0876	4-methyl-5(beta-hydroxyethyl)-thiazole monophosphate synthes	COG0693R	1.147826087	1.304347826	1.182608696				No	36.3021978	0.058241758	1455
-	SSA_0877	phosphoglycolate phosphatase	COG0546R	0.87654321		0.469135802				No	28.99638743	-0.25706806	266
gyrB	SSA_0878	DNA gyrase subunit B	COG0187L	1.113821138	1.073170732	0.959349593				Yes	29.83590139	-0.42850539	2581
ezrA	SSA_0879	septation ring formation regulator EzrA	COG4477D	0.939481268	0.76945245		0.346068	1.595271	0.160439	No	61.16324042	-0.45278746	423
-	SSA_0880	hypothetical protein	-							No	36.61422222	-0.44977778	4
-	SSA_0881	hypothetical protein	-							No	22.82262443	-0.44208145	4
serB	SSA_0882	phosphoserine phosphatase	COG0560E							No	16.61395349	-0.04837209	1530
grk	SSA_0883	glycerate kinase	COG1929G	0.975	1.1	1.2				No	43.11376344	0.113172043	1339
-	SSA_0884	hypothetical protein	-	1.115384615	0.884615385	0.692307692				No	31.81785714	-0.41964286	14
-	SSA_0885	hypothetical protein	COG5506S							No	54.47120805	-0.37583893	204
eno	SSA_0886	phosphopyruvate hydratase	COG0148G				0.834862	0.359873	0.25656	Yes	27.7643318	-0.2062212	2687
-	SSA_0887	hypothetical protein	-							No	41.12121212	-0.45454546	1
-	SSA_0888	magnesium/cobalt transporter	COG2239P	0.825396825		0.428571429				No	37.05721973	0.380941704	1668
-	SSA_0889	isoleucyl-tRNA synthetase	-	1.466666667	1.433333333	1.183333333	0.169618	0.514995	0.473358	No	20.09365079	-0.68412698	50
-	SSA_0891	3-carboxymuconate cyclase	COG2706G	1.083333333	1.083333333	1.055555556				No	29.72116071	-0.34345238	958
-	SSA_0892	ATP-binding cassette lipoprotein	-							No	32.59444444	-0.45	26
-	SSA_0893	ATP-binding cassette transporter-like protein	COG0845M							No	31.46769596	-0.68836105	259
-	SSA_0894	ATP-binding cassette protein	COG1136V							No	41.14776786	-0.284375	764
-	SSA_0895	ATP-binding cassette transporter-like protein	COG0577V							No	37.51481481	0.195061728	711
-	SSA_0896	two-component response transcriptional regulator	COG0745TK	1	1.106382979					No	54.78807339	-0.17614679	245
-	SSA_0897	two component system histidine kinase	COG0642T	1.096774194	1.241935484	1.35483871				No	38.72846827	-0.24967177	286
-	SSA_0898	hypothetical protein	-							No	60.55806452	-0.26774194	1
-	SSA_0899	permease	COG0628R	1.052631579	0.969924812	0.819548872				No	28.12579082	0.817091837	1872
-	SSA_0900	hypothetical protein	COG2956G	1.095238095	1.103174603	1				No	40.61589242	-0.30440098	497
aldB	SSA_0901	alpha-acetolactate decarboxylase	COG3527Q	0.980519481	0.857142857	0.603896104				No	25.91171548	-0.25481172	576
-	SSA_0903	NAD(P)H dehydrogenase (quinone)	COG0655R	0.672131148	0.557377049	0.426229508				No	43.37607362	-0.30797546	503
crpA	SSA_0904	CshA-like fibrillar surface protein A	-							No	17.90983612	-0.51264214	45
crpB	SSA_0905	CshA-like fibrillar surface protein B	-	1.112903226	1.048387097	1.048387097				No	24.28931841	-0.52639878	35
crpC	SSA_0906	CshA-like fibrillar surface protein C	-	0.994818653	1.062176166	1.202072539				No	23.94654178	-0.54162608	29
-	SSA_0907	fibronectin-binding protein A	COG1293K							No	44.27287796	-0.48797814	1027
-	SSA_0908	ABC transporter periplasmic protein	COG2984R	0.884615385	0.692307692	0.557692308				No	23.75164179	-0.12358209	685
-	SSA_0909	AbrB family transcriptional regulator	COG2002K	0.905660377	0.685534591	0.27672956				No	23.48888889	-0.35873016	149
-	SSA_0910	multidrug ABC transporter ATPase	COG1131V	1.539823009	2.185840708	2.707964602				No	40.80746888	-0.20871369	1753
-	SSA_0911	hypothetical protein	COG1511S	1.510309278	2.12371134	2.494845361				No	41.14638211	1.028455285	85
pheS	SSA_0912	phenylalanyl-tRNA synthetase subunit alpha	COG0016J	0.925	0.7125	0.5625				Yes	49.6304298	-0.3765043	2751
-	SSA_0913	acetyltransferase	COG0456R	0.983870968		0.709677419				No	31.61366864	-0.5147929	777
pheT	SSA_0914	phenylalanyl-tRNA synthetase subunit beta	COG0072J	1.17	1.15	1.09				Yes	38.10076155	-0.02359551	2705
-	SSA_0915	hypothetical protein	COG3272S							No	38.04076923	-0.49076923	335
-	SSA_0916	2-hydroxy-6-oxo-6-phenylhexa-2,4-dienoate hydrolase	COG0596R							No	47.628	-0.05309091	651
-	SSA_0917	cobalamin-independent methionine synthase II	COG0620E							No	30.28196382	-0.51989664	404
-	SSA_0918	hypothetical protein	-	1.386138614	1.96039604	2.762376238				No	22.91225806	-0.78451613	5
-	SSA_0920	hypothetical protein	-		1.578947368	1.894736842				No	37.63903226	-0.15483871	1
adhB	SSA_0921	Zn-dependeant threonine dehydrogenase	COG1063ER	1	1.021052632	0.947368421				No	30.60492754	-0.06202899	1280
-	SSA_0922	hypothetical protein	COG3933K							No	46.906639	-0.44356847	38
-	SSA_0923	TetR/AcrR family transcriptional regulator	COG1309K							No	50.76638614	-0.41287129	202
-	SSA_0924	peptide ABC transporter permease	COG4591M	0.833333333		0.527777778				No	37.70508929	0.442261905	484
-	SSA_0925	peptide ABC transporter ATPase	COG1136V							No	23.31809955	-0.28823529	355
-	SSA_0926	histone acetyltransferase HPA2-like acetyltransferase	COG3153R							No	61.28775362	-0.34130435	602
-	SSA_0927	TetR/AcrR family transcriptional regulator	COG1309K						11.85434	No	46.51724138	-0.43842365	322
-	SSA_0928	multidrug ABC transporter ATPase/permease	COG1132V							No	32.00462585	0.214795918	325
-	SSA_0929	multidrug ABC transporter ATPase/permease	COG1132V							No	28.8664716	0.192598967	471
-	SSA_0931	hypothetical protein	-							No	57.7308642	-0.50740741	4
-	SSA_0932	hypothetical protein	COG4487S	0.74137931				1.087018		No	44.10262411	-0.79763593	225
-	SSA_0933	acetyltransferase	COG0456R							No	33.10687075	-0.19115646	464
-	SSA_0934	HydD	COG0596R	1.017857143	1	1.142857143				No	25.84868914	-0.08651685	1075
truB	SSA_0935	tRNA pseudouridine synthase B	COG0130J	1.057142857	1.114285714	1.214285714				No	31.19832192	-0.27739726	2621
mreA	SSA_0936	bifunctional riboflavin kinase/FMN adenylyltransferase	COG0196H	0.571428571		0.342857143				Yes	25.26709677	-0.29193548	2459
spxA	SSA_0937	Spx family transcriptional regulator	COG1393P	0.892086331	0.834532374	0.676258993				No	76.71052632	-0.27819549	1179
-	SSA_0938	hypothetical protein	COG4476S							No	24.9673913	-0.63804348	380
suhB	SSA_0939	inositol monophosphatase	COG0483G	0.982758621	1.017241379	0.948275862				No	23.23763838	-0.17712177	2046
-	SSA_0940	NOL1/NOP2/sun family protein	COG0144J	0.948717949	1.012820513	1.025641026				No	31.45394009	-0.35737327	788
pstS	SSA_0941	phosphate ABC transporter substrate-binding protein	COG0226P	1.047619048	1.063492063	1.071428571				Yes	26.3334471	-0.14163823	1684
pstC1	SSA_0942	phosphate ABC transporter transmembrane protein	COG0573P	1.184	1.288	1.336				No	31.74885246	0.799016393	2094
pstC	SSA_0943	phosphate ABC transporter permease	COG0581P	1.237037037	1.37037037	1.437037037				Yes	31.11054422	0.695918367	2137
pstB2	SSA_0944	phosphate ABC transporter ATP-binding protein	COG1117P	1.291970803	1.481751825	1.686131387				Yes	36.43898876	-0.40037453	985
pstB1	SSA_0945	phosphate transporter ATP-binding protein	COG1117P	1.282208589	1.441717791	1.595092025				No	37.84396887	-0.28326848	1611
phoU	SSA_0946	phosphate transporter PhoU	COG0704P	1.282352941	1.458823529	1.423529412	0.253888			No	35.3816129	-0.33087558	2010
-	SSA_0947	hypothetical protein	-							No	38.99695431	-0.37817259	1
-	SSA_0948	hypothetical protein	-							No	27.86598985	-0.38071066	4
-	SSA_0949	hypothetical protein	-							No	33.70346535	-0.36881188	5
-	SSA_0950	hypothetical protein	-	0.902439024	0.707317073	0.585365854				No	29.66540598	-0.60384615	8
-	SSA_0952	hypothetical protein	-	0.936170213		0.531914894				No	52.01326531	-0.15408163	7
-	SSA_0954	hypothetical protein	-	0.980769231		0.596153846	0.463062			No	59.42314815	-1.51759259	5
pepN	SSA_0955	aminopeptidase	COG0308E	1.016528926	0.958677686	0.867768595	0.751513	0.628624	0.57278	No	33.79540189	-0.27553192	1595
sspD	SSA_0956	surface protein D	COG0419L	0.958333333	0.8125	0.916666667				No	53.80460248	-0.75827863	26
-	SSA_0957	dehydrogenase	COG0300R							No	32.85867159	0.042804428	58
-	SSA_0958	hypothetical protein	-							No	56.32987013	-1.25519481	12
ciaR	SSA_0959	two-component response transcriptional regulator	COG0745TK	1.204545455	1.352272727	1.147727273				No	20.6946875	-0.07767857	1457

ciaH	SSA_0960	sensor protein ciaH	COG0642T	1.333333333	1.648148148	1.666666667				No	30.37066955	-0.36933045	421
-	SSA_0961	ketopantoate reductase PanE/ApbA	COG1893H	1.296296296	1.574074074	1.481481481				No	35.08557692	-0.09903846	100
lguL	SSA_0962	lactoylglutathione lyase	COG0346E							No	21.70242188	-0.45859375	1357
-	SSA_0963	peptidoglycan N-acetylglucosamine deacetylase A	COG0726G	1	1.0375	1.1375				No	37.43275269	-0.40107527	1107
deaD	SSA_0964	DEAD/DEAH box helicase	COG0513LKJ	1.12195122	1.036585366	1.024390244				No	33.95080311	-0.18238342	336
mocA	SSA_0965	oxidoreductase	COG0673R	0.968253968	0.904761905	0.841269841				No	18.86904762	-0.14613095	1210
udK	SSA_0966	uridine kinase	COG0572F	0.545454545	0.363636364	0.272727273			0.179543	No	49.55348837	-0.45627907	1220
-	SSA_0967	hypothetical protein	COG3619S							No	36.65004505	0.646846847	359
-	SSA_0968	hypothetical protein	-							No	36.08666667	-0.92666667	7
-	SSA_0969	hypothetical protein	-	0.972972973	0.675675676	0.378378378				No	55.76413793	-0.56413793	5
-	SSA_0970	hypothetical protein	-		1.565217391					No	23.43421053	-0.48289474	13
-	SSA_0971	hypothetical protein	-	1.114285714	1.385714286	1.457142857				No	25.62202749	-0.50635739	17
-	SSA_0972	hypothetical protein	-		1.320987654	1.419753086				No	28.2592233	-0.23592233	9
-	SSA_0973	hypothetical protein	-	1.008	1.216	1.296				No	46.83786408	-1.13592233	3
leuA	SSA_0975	2-isopropylmalate synthase	COG0119E	0.941176471	0.941176471	0.941176471				No	27.00104895	-0.06853147	2126
leuB	SSA_0977	3-isopropylmalate dehydrogenase	COG0473CE	0.947368421	1.026315789	1.078947368		0.269674		No	40.30898551	0.037681159	2074
-	SSA_0978	hypothetical protein	COG3326S	1.076923077	1.269230769	1.346153846				No	32.61477273	0.486363636	243
leuC	SSA_0980	isopropylmalate isomerase large subunit	COG0065E	0.944444444	1.138888889	1.25				No	35.68391304	-0.20369565	2109
leuD	SSA_0981	isopropylmalate isomerase small subunit	COG0066E							No	21.97602041	-0.44642857	2068
-	SSA_0983	hypothetical protein	COG0009J	0.659090909						No	38.20698842	-0.45019305	88
-	SSA_0984	SlyA-like protein	COG1846K							No	41.46558621	-0.52	386
-	SSA_0985	hypothetical protein	COG4922S							No	25.24527559	-0.5515748	229
-	SSA_0986	choline transporter	COG1125E							No	51.53264463	-0.1838843	1180
proW	SSA_0987	choline ABC transporter permease	COG1732M	1.391304348	1.434782609	1.652173913				No	32.28735178	0.183794466	1294
-	SSA_0988	TfoX domain-containing protein	COG3070K							No	56.7247619	-0.17428571	167
-	SSA_0989	transcriptional regulator	-			0.538461538				No	48.41674208	-0.25882353	143
-	SSA_0990	hypothetical protein	-							No	32.18571429	0.763673469	189
-	SSA_0991	deoxyribonuclease	-							No	23.38660079	-0.59525692	144
-	SSA_0992	hypothetical protein	COG2320S							No	43.1507772	-0.66632124	371
-	SSA_0993	hypothetical protein	COG2226H							No	37.77892157	0.057843137	793
-	SSA_0994	peptidase E	COG3340E							No	30.51660194	-0.13543689	468
-	SSA_0995	alpha/beta hydrolase	COG0596R							No	23.94157303	-0.51516854	54
-	SSA_0996	cGMP-specific phosphodiesterase	COG1956T		0.477272727	0.318181818				No	28.87036364	0.003030303	1136
dnaX	SSA_0997	DNA polymerase III subunits gamma and tau	COG2812L	0.724637681						Yes	37.25485612	-0.2307554	2723
-	SSA_0998	hypothetical protein	-	0.78125		0.541666667				No	32.96349206	0.392063492	94
birA	SSA_0999	biotin--protein ligase	COG0340H	2.52	4.52	5.64				Yes	36.65729904	-0.2221865	2234
galR	SSA_1000	LacI family transcriptional repressor	COG1609K	2.772727273	4.545454545	4.727272727	0.366897	0.601134	0.629684	No	41.25135135	-0.3021021	493
msmR	SSA_1001	arabinose (multiple sugar metabolism) operon transcriptional re	COG2207K							No	39.76386207	-0.43241379	768
-	SSA_1002	alpha-galactosidase	COG3345G					0.309893		No	33.00322222	-0.36444444	128
-	SSA_1003	sugar ABC transporter substrate-binding protein	COG1653G							No	30.99572792	-0.4026253	1062
msmF	SSA_1004	sugar ABC transporter permease	COG1175G							No	28.83930556	0.846527778	1237
msmG	SSA_1005	sugar ABC transporter permease	COG0395G							No	23.6166065	0.652707581	557
gtfA	SSA_1006	dextransucrase	COG0366G							No	38.7975052	-0.46507277	392
-	SSA_1007	sugar ABC transporter ATP-binding protein	COG3839G					0.171746		No	35.46269841	-0.28042328	594
galK	SSA_1008	galactokinase	COG0153G	16.41666667	41.75	58				No	27.01201531	-0.27806122	1146
galT	SSA_1009	galactose-1-phosphate uridylyltransferase	COG4468G	10.7	34.5	57.5				No	42.72375254	-0.37281947	359
galE	SSA_1010	UDP-glucose 4-epimerase	COG1087M	2.131578947	6.184210526	10.71052632				No	28.08821326	-0.17348703	2318
-	SSA_1011	hypothetical protein	-							No	30.78020408	0.632653061	119
-	SSA_1012	phosphoenolpyruvate synthase	COG0574G	1.037037037	1.222222222	1.296296296				No	45.5556077	-0.26702768	314
-	SSA_1013	phosphatidylserine synthase	COG1183I							No	42.91904762	0.853333333	1397
-	SSA_1014	phosphatidylserine decarboxylase	COG0688I		1.464285714	1.714285714				No	33.73344828	-0.16310345	983
ubiA	SSA_1015	prenyltransferase	COG0382H	1.085714286	1.457142857	1.885714286				No	29.54314381	0.610367893	18
-	SSA_1016	phosphoenolpyruvate synthase	COG0574G	1.096153846	1.384615385	1.730769231				No	47.67426844	-0.2662636	197
-	SSA_1017	hypothetical protein	-	1.105263158	1.421052632	1.666666667				No	42.21132394	-0.23633803	220
-	SSA_1018	Zinc metalloprotease zmpC	COG3583S							No	31.22078438	-0.5599278	157
-	SSA_1019	collagen-binding surface protein	COG4932M	1.158682635	1.341317365	1.547904192				No	21.10882134	-0.50186104	51
-	SSA_1020	hypothetical protein	-							No	28.77483871	0.779354839	1
-	SSA_1021	hypothetical protein	COG1363G	0.741935484		0.5				No	29.66123563	-0.20574713	492
-	SSA_1022	group 1 glycosyl transferase	COG0438M	0.696969697		0.515151515				No	36.9574124	-0.27385445	261
-	SSA_1023	von Willebrand factor A	-	0.908045977	0.83908046	0.862068966				No	40.08497194	-0.61582492	14
amy	SSA_1024	alpha-amylase	COG0366G	1		0.483870968				No	27.31561475	-0.56782787	445
-	SSA_1026	multidrig ABC transporter ATPase	COG1131V	0.939393939	0.919191919	0.757575758				No	28.381417	-0.10607287	401
-	SSA_1027	hypothetical protein	COG2194R	1.170212766	1.255319149	1.191489362				No	35.76412844	0.814862385	125
-	SSA_1028	XRE family transcriptional regulator	COG2932K	1						No	34.04280156	-0.52023346	68
-	SSA_1029	hypothetical protein	COG1307S							No	30.91039286	-0.07964286	166
-	SSA_1030	TetR family transcriptional regulator	COG1309K	1.007751938	0.84496124	0.790697674				No	31.20162304	-0.46806283	288
flaR	SSA_1031	topology modulation protein	COG0563F							No	48.42381503	-0.82716763	510
rpsT	SSA_1032	30S ribosomal protein S20	COG0268J				2.173634	1.094225		No	36.68846154	-0.74358974	1986
coaA	SSA_1033	pantothenate kinase	COG1072H	1.28		0.88				No	43.24052288	-0.40784314	1042
-	SSA_1034	hypothetical protein	COG2813J	0.903225806		0.35483871				No	30.80153846	-0.18410256	1362
pdp	SSA_1035	pyrimidine-nucleoside phosphorylase	COG0213F	0.9	0.88	0.6				No	26.10117647	0.110588235	1379
deoC	SSA_1036	deoxyribose-phosphate aldolase	COG0274F	0.946564885	0.79389313	0.580152672				No	21.53681818	-0.01227273	1684
cdd	SSA_1037	cytidine deaminase	COG0295F	1	0.926174497	0.77852349				No	18.64263566	0.23255814	1517
-	SSA_1038	lipoprotein	COG1744R	1.003968254	1.083333333	1.043650794				No	20.78034188	-0.38290598	919
-	SSA_1039	sugar ABC transporter ATP-binding protein	COG3845R	0.843537415	0.863945578	0.870748299	0.696552			No	32.662818	-0.09549902	1625
-	SSA_1040	sugar ABC transporter permease	COG4603R	0.913580247	1.00617284	0.969135802				No	36.26923077	0.800274725	1079
-	SSA_1041	sugar ABC transporter permease	COG1079R	0.953488372	1.011627907	0.988372093				No	22.77672956	0.950314465	1064
-	SSA_1042	xylanase/chitin deacetylase	COG0726G	0.74	0.47	0.35				No	31.15890966	-0.49096573	1098
hom	SSA_1043	homoserine dehydrogenase	COG0460E	0.765765766		0.378378378	0.065384	0.667726		No	28.33598131	0.115420561	1831
thrB	SSA_1044	homoserine kinase	COG0083E	0.970149254	0.71641791	0.604477612				No	38.11875	-0.10659722	1603
-	SSA_1045	hypothetical protein	-	1.05	0.95	0.7				No	30.18877888	0.311551155	18
murB	SSA_1047	UDP-N-acetylenolpyruvoylglucosamine reductase	COG0812M	0.775862069		0.465517241				Yes	25.40664452	-0.07009967	2451
potA	SSA_1048	spermidine/putrescine ABC transporter ATP-binding protein	COG3842E	0.984615385	0.984615385	0.953846154				No	52.31846753	-0.31298701	1787
potB	SSA_1049	spermidine/putrescine ABC transporter transmembrane protein	COG1176E	1.126984127	1.19047619	1.333333333				No	38.06791045	0.635074627	1883
potC	SSA_1050	spermidine/putrescine ABC transporter permease	COG1177E	1.176470588	1.367647059	1.529411765				No	27.45568093	0.826459144	1708
potD	SSA_1051	spermidine/putrescine ABC transporter substrate-binding protei	COG0687E	1.158536585	1.280487805	1.329268293				No	37.13542135	-0.41264045	1523
-	SSA_1052	hypothetical protein	-	0.753303965						No	29.53623188	-0.61304348	25
-	SSA_1053	pyruvate phosphate dikinase	COG0574G					0.087235		No	42.78912944	-0.21741123	1051
-	SSA_1054	hypothetical protein	COG0517R		0.571428571	0.571428571				No	43.26842105	0.075119617	429
-	SSA_1055	hypothetical protein	COG1806S				0.101264		0.315619	No	39.92132353	-0.16617647	1308
-	SSA_1056	Firmicute fructose-1,6-bisphosphatase	COG3855G	1.022727273	1.022727273	1.159090909				No	47.89341195	-0.30424528	317

-	SSA_1057	class V aminotransferase	COG1104E	0.634615385		0.403846154				No	37.55947368	-0.07026316	1099
thiI	SSA_1058	thiamine biosynthesis protein ThiI	COG0301H	0.777777778						No	42.76955446	-0.24851485	1249
-	SSA_1059	hypothetical protein	COG2020O							No	29.45728324	0.537572254	326
-	SSA_1060	hypothetical protein	-							No	23.32405	0.0745	3
rplU	SSA_1061	50S ribosomal protein L21	COG0261J	0.981748319		0.391930836	0.884941	0.662651	0.92236	No	17.02115385	-0.30769231	2517
rpmA	SSA_1062	50S ribosomal protein L27	COG0211J	1.135640786	0.923292797		0.365068	0.626263	0.394774	Yes	26.31237113	-0.57938144	2570
-	SSA_1063	peptidoglycan binding domain-containing protein	COG1240H							No	30.26246667	-0.27733333	71
-	SSA_1064	hypothetical protein	COG5263R							No	31.77044715	-0.75528455	10
-	SSA_1065	Beta-hexosamidase A	COG1472G	2.2		7.2				No	31.79001075	-0.14053763	1831
aliB	SSA_1066	peptide ABC transporter	COG4166E	0.467336683	0.296482412	0.256281407				No	33.19019908	-0.60597244	380
-	SSA_1067	hypothetical protein	COG0456R		0.545454545	0.431818182				No	38.08333333	-0.22430556	338
-	SSA_1068	LysR family transcriptional regulator	COG0583K	0.830508475	0.677966102	0.474576271				No	36.16003226	-0.46516129	1683
lspA	SSA_1069	lipoprotein signal peptidase	COG0597MU	1		0.637931034				No	15.62903226	0.787741935	2253
-	SSA_1070	ribosomal large subunit pseudouridine synthase D	COG0564J	1.090909091	0.95959596	0.757575758				No	26.0909396	-0.35469799	2591
proB	SSA_1072	gamma-glutamyl kinase	COG0263E		0.541666667	0.458333333				No	23.76452575	-0.06504065	2001
proA	SSA_1073	gamma-glutamyl phosphate reductase	COG0014E	0.979591837	0.816326531	0.755102041				No	26.93619048	-0.11214286	1952
proC	SSA_1074	pyrroline-5-carboxylate reductase	COG0345E	0.979591837	0.87755102					No	41.21348315	0.079775281	2256
hemN	SSA_1075	coproporphyrinogen III oxidase	COG0635H	0.833333333		0.583333333				No	40.43803191	-0.32898936	2430
-	SSA_1076	hypothetical protein	COG3884I	0.974683544	0.860759494	0.772151899			0.074554	No	41.32849594	-0.30081301	407
nagD	SSA_1077	N-acetyl-glucosamine matabolism	COG0647G	1.00952381	0.961904762	0.857142857	0.206535	0.019248		No	23.81640625	-0.09375	1321
-	SSA_1078	hypothetical protein	COG4478S	1.157142857	1.1	1.085714286				No	25.15023923	0.699043062	295
-	SSA_1079	hypothetical protein	-	1.197183099	1.126760563	0.985915493				No	42.08966361	0.632415902	169
fruR	SSA_1080	fructose operon transcriptional repressor	COG1349KG							No	36.33076923	-0.19311741	1052
fruB	SSA_1081	1-phosphofructokinase	COG1105G							No	22.47128713	-0.06006601	1411
fruA	SSA_1082	PTS system fructose specific transporter subunit II ABC	COG1299G	2.5		7.75				No	29.84540091	0.394099849	1769
-	SSA_1083	hypothetical protein	COG4835S	0.45	0.2375	0.1125				No	35.80806452	-0.25564516	204
-	SSA_1084	hypothetical protein	COG1307S	0.659574468	0.361702128	0.265957447				No	38.26531915	0.133333333	626
dapB	SSA_1085	dihydrodipicolinate reductase	COG0289E	0.765217391	0.495652174	0.339130435	0.178419	1.197387	0.105064	No	34.48784314	-0.04039216	2415
papS	SSA_1086	tRNA CCA-pyrophosphorylase	COG0617J	0.979166667	0.854166667	0.75				Yes	41.50626566	-0.2245614	2476
-	SSA_1087	antibiotic ABC transporter ATPase	COG0488R	1.039215686	0.980392157	0.921568627				No	38.83231511	-0.41784566	1849
-	SSA_1088	hypothetical protein	COG1246E							No	38.20080882	-0.89191177	475
-	SSA_1089	hypothetical protein	-							No	18.23142857	0.394285714	1
glcK	SSA_1090	glucokinase	COG1940KG	0.941176471	0.948529412	0.75	0.283896	0.261013	0.295181	No	30.95394984	0.115673981	1800
thyA	SSA_1091	thymidylate synthase	COG0207F	0.911764706	0.573529412	0.294117647				Yes	40.7978853	-0.39247312	1799
dfrA	SSA_1092	dihydrofolate reductase	COG0262H	0.721428571		0.314285714				Yes	39.70882353	-0.48705882	1941
clpX	SSA_1093	ATP-dependent protease ATP-binding subunit ClpX	COG1219O	0.810810811			0.395322	0.415089	0.275791	No	53.13618582	-0.20977995	2487
engB	SSA_1094	ribosome biogenesis GTP-binding protein YsxC	COG0218R	0.844067797	0.725423729		0.100179			Yes	35.47538462	-0.4974359	2053
mur2	SSA_1095	peptidoglycan hydrolase	COG1705NU		0.194029851	0.104477612				No	40.09137931	-0.48577586	762
mmuM	SSA_1096	homocysteine methyltransferase	COG2040E							No	44.51746032	-0.31587302	1706
-	SSA_1098	formate/nitrate transporter	COG2116P	3.333333333	3	1.916666667				No	36.21283019	0.736603774	644
-	SSA_1099	calcium binding hemolysin-like protein	COG2931Q							No	28.06168585	-0.42830061	612
-	SSA_1100	hemolysin exporter, ATPase component	COG2274V	0.852459016	0.950819672	1.06557377				No	30.16203938	0.252039381	1062
-	SSA_1101	multidrug resistance efflux pump/hemolysin secretion transmem	COG1566V	0.902597403	1.006493506	1.136363636				No	33.39427861	-0.18830846	948
-	SSA_1102	hypothetical protein	-							No	45.4399115	0.186725664	33
-	SSA_1103	hypothetical protein	-							No	36.90485577	0.021153846	1
rplJ	SSA_1104	50S ribosomal protein L10	COG0244J	0.810204082			0.638498	1.904412	0.227318	Yes	45.31204819	0.230120482	2571
rplL	SSA_1105	50S ribosomal protein L7/L12	COG0222J	0.939220183	0.790137615		0.2646	1.176471	0.051328	Yes	37.77213115	0.287704918	2552
iga	SSA_1106	IgA-specific metalloendopeptidase	COG3583S	1	0.852459016	0.852459016				No	33.53661686	-0.49039488	115
-	SSA_1107	Lipid/multidrug/protein-type ABC exporter, ATP binding/membr	COG1132V	0.75						No	31.22821799	0.10017301	567
-	SSA_1109	ABC transporter ATP-binding protein/permease	COG1132V	0.90625	0.9375	0.9375				No	28.58162162	0.135810811	1400
-	SSA_1110	hypothetical protein	-	1.056603774						No	54.88391304	-0.50652174	141
brnQ	SSA_1111	branched chain amino acid ABC transporter carrier protein	COG1114E							No	22.96696833	0.932352941	1076
-	SSA_1112	cell wall surface anchor family protein	-						0.499684	No	24.31641905	-0.51238095	55
-	SSA_1113	two-component response transcriptional regulator	COG0745TK							No	41.87931034	-0.19008621	258
-	SSA_1114	histidine kinase	COG5002T							No	48.09476744	0.02122093	819
-	SSA_1115	cytochrome C-type biogenesis protein	COG0785O							No	31.14067797	0.947457627	1179
-	SSA_1116	hypothetical protein	-							No	56.79339623	-1.22924528	7
-	SSA_1117	hypothetical protein	COG1225O							No	31.76042781	-0.41016043	549
-	SSA_1118	peptide methionine sulfoxide reductase	COG0229O				2.42771	1.305164		No	36.95582656	-0.62764228	1450
-	SSA_1119	two-component response transcriptional regulator	COG4753T							No	28.54303279	-0.31147541	962
-	SSA_1120	two-component sensor kinase	COG2972T	0.96	0.8					No	40.00723104	-0.23368607	1343
-	SSA_1121	cytochrome C-type biogenesis protein	COG0785O		0.52	0.36				No	26.09404255	0.966808511	552
-	SSA_1122	thioredoxin family protein	COG0526OC	1.268292683		0.853658537				No	40.38062827	-0.26335079	1425
-	SSA_1124	oxidoreductase	COG2461S	2.050632911	3.506329114		0.475595	0.102677	0.029003	No	28.5589372	-0.40555556	370
-	SSA_1125	NADPH-dependent FMN reductase	COG0431R	2.737373737	4.494949495	5.797979798	0.197	0.843023	0.389552	No	45.86069652	-0.1079602	922
apbE	SSA_1126	thiamine biosynthesis lipoprotein	COG1477H	2.359223301	3.145631068	3.233009709				No	38.54469453	-0.03987138	1460
nox	SSA_1127	H2O-forming NADH dehydrogenase	COG0446R	3.925531915	3.734042553	2.840425532	0.26689	0.976303	0.380827	No	30.0869214	-0.12358079	1804
-	SSA_1128	voltage gated chloride channel EriC	COG0038P							No	35.06795367	0.637065637	1456
-	SSA_1129	periplasmic iron transport lipoprotein	COG2822P							No	22.10931034	-0.54551724	434
-	SSA_1130	Iron-dependent peroxidase	COG2837P							No	29.09977612	-0.5619403	825
-	SSA_1131	high-affinity Fe 2+/Pb2+ permease	COG0672P							No	32.62556886	0.225748503	584
tatC	SSA_1132	TatC, sec-independent protein translocase	COG0805U							No	41.09644	0.8072	1932
tatA	SSA_1133	TatA, sec-independent protein secretion pathway component	COG1826U	1.232394366	1.330985915	1.330985915				No	47.88870968	-0.10806452	450
xpt	SSA_1134	xanthine phosphoribosyltransferase	COG0503F							No	25.26336634	0.197524752	681
norM	SSA_1135	Na+-driven multidrug efflux pump	COG0534V							No	25.16872483	0.717449664	1293
-	SSA_1136	ATPases with chaperone activity, ATP-binding subunit	COG0542O		2.8	3.1				No	32.32330656	-0.28995984	44
pdhD	SSA_1137	dihydrolipoamide dehydrogenase	COG1249C	3.5	7	11				No	34.47713004	0.0367713	1061
pdhA	SSA_1138	pyruvate dehydrogenase, TPP-dependent E1 component alpha	COG1071C	2.8	5.6	9.4				No	36.72492997	-0.40504202	650
pdhB	SSA_1139	pyruvate dehydrogenase E1 component beta subunit	COG0022C	1.833333333	3.5	6				No	32.39912536	0.031778426	1227
pdhC	SSA_1140	branched-chain alpha-keto acid dehydrogenase subunit E2	COG0508C	1.5	3	5.5				No	29.849642	-0.06205251	1365
norM2	SSA_1143	Na+-driven multidrug efflux pump	COG0534V							No	25.82296214	0.888864143	584
-	SSA_1144	beta-N-acetylhexosaminidase	COG3525G							No	36.3742879	-0.36156202	378
-	SSA_1145	hypothetical protein	-							No	22.34213793	-0.96068966	11
ptcA	SSA_1146	phosphotransferase system cellobiose-specific component IIA	COG1447G							No	39.53181818	-0.14818182	301
-	SSA_1147	Beta-glucosides PTS, EIIB	COG1440G							No	46.11875	0.15625	48
-	SSA_1148	Beta-glucosides PTS, EIIC	COG1455G							No	34.05862069	0.734712644	55
-	SSA_1149	glycosyl hydrolase family protein	COG2723G							No	35.61036559	-0.48150538	625
xylH	SSA_1150	4-oxalocrotonate tautomerase	COG1942R							No	50.895	-0.425	611
tdk	SSA_1151	thymidine kinase	COG1435F	0.483870968	0.387096774	0.290322581				No	34.55789474	-0.30842105	1307
prfA	SSA_1152	peptide chain release factor 1	COG0216J	0.803921569	0.774509804	0.784313725				Yes	39.03454039	-0.66963788	2591
hemK	SSA_1153	HemK protein	COG2890J	0.99	1.02	1.05				No	30.97681159	-0.2173913	2634

-	SSA_1154	hypothetical protein	COG0009J	1	0.933333333	0.877777778				No	38.83265306	-0.12040816	2578
glyA	SSA_1155	serine hydroxymethyltransferase	COG0112E	1.084337349	1.277108434	1.337349398	0.951683	0.804348	0.68785	No	35.57214286	-0.05095238	2662
-	SSA_1156	hypothetical protein	-	1.151162791	1.372093023	1.593023256				No	37.57561728	-0.46327161	205
-	SSA_1157	PvaA-like protein	COG0741M	1.160377358	1.386792453	1.462264151				No	29.67	-0.2875	247
-	SSA_1158	hypothetical protein	COG4814R							No	41.39478873	-0.42147887	216
-	SSA_1161	hypothetical protein	COG4086S	0.838235294	0.485294118	0.352941176				No	24.16779141	-0.19815951	331
-	SSA_1162	fimbrial assembly protein fimC	-							No	40.60849802	0.682213439	70
guaA	SSA_1163	GMP synthase	COG0519F	0.728070175		0.377192982				No	31.40211538	-0.15230769	2503
-	SSA_1164	hypothetical protein	-							No	39.59767442	0.21627907	1
-	SSA_1165	GntR family transcriptional regulator	COG2188K	0.675925926	0.453703704	0.268518519				No	34.58538793	-0.49224138	1656
ylxM	SSA_1166	DNA-binding protein	COG2739S	0.612903226	0.35483871	0.225806452				No	63.57088496	-0.53539823	642
ffh	SSA_1167	SRP54, signal recognition particle GTPase protein	COG0541U	0.852272727		0.443181818	0.236258	0.52796	0.453871	Yes	43.73167939	-0.36374046	2698
-	SSA_1168	hypothetical protein	-	1.130952381	0.952380952	0.892857143				No	32.4259325	-0.38223801	3
-	SSA_1169	hypothetical protein	COG4814R							No	27.63394834	-0.34243542	147
-	SSA_1170	hypothetical protein	-							No	43.19444444	-0.65438597	1
xerS	SSA_1171	site-specific tyrosine recombinase XerS	COG4974L							No	34.67952247	-0.45365169	2433
-	SSA_1172	hypothetical protein	-							No	36.33683962	-0.36509434	4
lplA	SSA_1173	lipoate protein ligase A	COG0095H	2.166666667	2.261904762	2.785714286				No	37.46352584	-0.34711246	1250
acoL	SSA_1174	dihydrolipoamide dehydrogenase	COG1249C	2.222222222	2.333333333	2.444444444	0.693283	0.923079	0.443791	No	31.88292254	0.062147887	1675
acoC	SSA_1175	dihydrolipoamide acetyltransferase	COG0508C	2.40625	2.21875	2.40625				No	32.81527378	-0.05792507	1248
acoB	SSA_1176	acetoin dehydrogenase, E1 component subunit beta	COG0022C	3.047619048	3.285714286	3.904761905				No	33.6257764	-0.06118012	1386
acoA	SSA_1178	acetoin dehydrogenase, E1 component subunit alpha	COG1071C	2.833333333	3.277777778	3.222222222				No	35.14164596	-0.2515528	1115
-	SSA_1179	carbamoylphosphate synthase large subunit / biotin carboxylase	COG0439I	1.066666667	1.066666667	1.177777778				No	35.04796392	-0.38994845	245
-	SSA_1180	hypothetical protein	COG4947S							No	40.51670732	-0.30813008	174
-	SSA_1181	alpha/beta hydrolase	COG2819R		0.375	0.291666667				No	46.78507299	-0.45510949	336
gid	SSA_1182	tRNA (uracil-5-)-methyltransferase Gid	COG1206J	0.936170213	0.659574468	0.574468085	0.665573	1.3718	0.231878	No	36.62774775	-0.28963964	855
-	SSA_1183	hypothetical protein	-							No	25.17515528	-0.44161491	54
topA	SSA_1184	DNA topoisomerase I	COG0550L	0.784313725		0.431372549				Yes	34.64496413	-0.62725968	2723
smf	SSA_1185	DNA processing Smf protein	COG0758LU							No	38.76435714	-0.13857143	2277
-	SSA_1186	acetyltransferase	COG0110R							No	31.40540107	-0.1342246	563
licT	SSA_1187	BglG family transcriptional antiterminator	COG3711K							No	40.29892857	-0.32178571	602
rnhB	SSA_1188	ribonuclease HII	COG0164L							No	30.55669291	-0.31771654	2584
rbgA	SSA_1189	ribosomal biogenesis GTPase	COG1161R			0.68				Yes	29.72159011	-0.34982332	1098
-	SSA_1190	hypothetical protein	COG1328F	0.690140845	0.471830986	0.274647887				No	27.63571429	-0.21517857	117
-	SSA_1191	hypothetical protein	-							No	5.845454545	-1	1
-	SSA_1192	hypothetical protein	-							No	34.55427136	-0.21708543	2
dapA	SSA_1193	dihydrodipicolinate synthase	COG0329EM	0.875		0.53125	0.926128	0.541856	0.118178	No	37.22090032	-0.00836013	2484
asd	SSA_1194	aspartate-semialdehyde dehydrogenase	COG0136E	0.526785714	0.214285714	0.116071429	0.018654	1.485163	0.061322	No	31.14078212	-0.0150838	2496
-	SSA_1196	acetyltransferase	COG1670J							No	39.09086207	-0.61781609	249
alkD	SSA_1197	DNA alkylation repair protein	COG4912L							No	30.09351852	-0.46481482	292
-	SSA_1198	twitching mobility protein PilT	-							No	29.22019704	-0.40591133	43
-	SSA_1199	phosphinothricin acetyltransferase	COG1247M							No	37.63023256	-0.23116279	1318
fhs	SSA_1200	formate--tetrahydrofolate ligase	COG2759F							No	31.46366906	0.050179856	534
-	SSA_1201	phosphopantothenate--cysteine ligase	COG0452H				0.092551	0.427483		No	31.39339207	-0.069163	289
dfp	SSA_1202	phosphopantothenoylcysteine decarboxylase	COG0452H				0.581132	0.087152		No	22.31912568	0.074863388	2319
-	SSA_1203	hypothetical protein	COG4684S	1.423076923		1.038461538				No	25.0828877	1.187165775	293
pgm	SSA_1204	phosphoglucomutase	COG1109G	1.341013825	1.534562212	1.585253456	0.525084	0.811321	0.133745	No	30.93814685	-0.17412587	1854
bta	SSA_1205	hypothetical protein	-	1.714285714	1.946428571	1.589285714	0.040618	0.985915	0.024966	No	39.28695652	-0.15304348	186
mutY	SSA_1206	A/G-specific adenine glycosylase	COG1194L							No	45.74455959	-0.50699482	2122
eutD	SSA_1207	phosphotransacetylase	COG0280C	1.266666667	1.369230769	1.343589744	0.213554	0.054184	0.076431	No	35.084	0.02	2150
-	SSA_1208	23S rRNA pseudouridine synthase	COG0564J	1.345679012	1.209876543	1.049382716				No	35.218	-0.39932203	537
ppnK	SSA_1209	inorganic polyphosphate/ATP-NAD kinase	COG0061G	1.232876712	1.082191781	0.794520548				Yes	29.2620922	-0.34964539	2534
-	SSA_1210	GTP pyrophosphokinase	COG2357S	1.164179104	0.925373134	0.582089552				No	37.81793722	-0.64843049	667
-	SSA_1211	hypothetical protein	COG4116S							No	44.3973545	-0.58835979	387
prs	SSA_1212	ribose-phosphate pyrophosphokinase	COG0462FE	0.824427481		0.511450382	0.264333	0.515219		No	40.07169811	-0.0918239	227
-	SSA_1213	pyridoxal-phosphate dependent aminotransferase	COG1104E	0.925	0.775	0.641666667				Yes	40.69775253	-0.26237374	1887
-	SSA_1214	hypothetical protein	-	1.057692308		0.673076923				No	36.9766087	-0.29217391	221
-	SSA_1215	hypothetical protein	-	0.982758621	0.862068966	0.931034483				No	28.51369863	-0.05890411	54
-	SSA_1216	redox-sensing transcriptional repressor Rex	COG2344R	1.670454545	2.181818182	2.238636364				No	30.31860465	-0.15209302	787
-	SSA_1217	hypothetical protein	COG2071R	1.684210526	2.390977444	3.030075188				No	44.087	-0.19130435	606
radC	SSA_1218	DNA repair protein RadC	COG2003L							No	52.2753304	-0.25462555	1801
srtA	SSA_1219	sortase	COG3764M	1.101449275	1.086956522	1.082125604				No	29.99203187	-0.23067729	340
gyrA	SSA_1220	DNA gyrase subunit A	COG0188L	1.076923077	1.043956044	0.923076923				Yes	32.61549637	-0.32651332	2644
ldh	SSA_1221	L-lactate dehydrogenase	COG0039C	0.924117205	0.930879038		0.230866	0.629696	0.501441	No	26.33780488	0.012804878	1886
-	SSA_1222	NTP pyrophosphohydrolases including oxidative damage repair	COG1051F							No	36.51666667	-0.27463768	497
rpsA	SSA_1223	30S ribosomal protein S1	COG0539J	0.424427481	0.134351145	0.079389313	0.784314	0.754206	0.38266	Yes	25.07256858	-0.13965087	2530
-	SSA_1224	hypothetical protein	-	0.810344828	0.603448276	0.198275862				No	25.94223684	-0.41973684	151
ilvE	SSA_1225	branched-chain amino acid aminotransferase	COG0115EH	0.592356688		0.356687898	3.528031	0.042625		No	34.0720059	-0.16961652	2111
parC	SSA_1226	DNA topoisomerase IV subunit A	COG0188L	1.062937063	1.223776224	1.27972028				Yes	39.31029412	-0.31568628	496
-	SSA_1227	aminoglycoside adenylyltransferase	-	1.078125	1.234375	1.3828125				No	36.94779412	-0.27095588	195
-	SSA_1229	hypothetical protein	-	1.177083333	1.333333333	1.729166667				No	22.38571429	-0.50571429	1
-	SSA_1230	hypothetical protein	-	0.980582524	0.980582524	1.029126214	0.59478	0.609004	1.929586	No	30.40040323	-0.57217742	30
-	SSA_1231	hypothetical protein	-	0.981481481	0.851851852	0.944444444				No	43.19566396	0.468834688	1
parE	SSA_1232	DNA topoisomerase IV subunit B	COG0187L	0.758064516						Yes	28.74577812	-0.4642527	571
-	SSA_1233	glycerol-3-phosphate acyltransferase PlsY	COG0344S							Yes	32.54259259	0.89537037	1879
-	SSA_1234	5'-nucleotidase	COG0737F	0.917582418	0.901098901	0.862637363				No	33.35173853	-0.34617524	1156
pyrC	SSA_1235	dihydroorotase	COG0044F	1.123595506	1.415730337	1.730337079				No	22.4535545	0.011137441	2151
mutX	SSA_1236	Mutator protein	COG1051F	1.189873418	1.506329114	1.924050633				No	16.21824675	-0.38051948	534
ung	SSA_1237	uracil-DNA glycosylase	COG0692L	1.203703704	1.574074074	1.907407407				No	33.55115207	-0.10645161	1936
-	SSA_1238	hypothetical protein	-	1.166666667	1.5	1.814814815				No	51.1324	-0.248	104
-	SSA_1239	hypothetical protein	COG1807M	1.107142857	1.25	1.25				No	35.69572266	0.319726563	67
pyrE	SSA_1240	orotate phosphoribosyltransferase	COG0461F							No	26.84736842	-0.09425837	1898
pyrF	SSA_1241	orotidine 5'-phosphate decarboxylase	COG0284F							No	36.43917391	-0.17782609	1698
pyrD	SSA_1242	dihydroorotate dehydrogenase 1B	COG0167F							No	33.57115385	0.147435897	2042
pyrK	SSA_1243	dihydroorotate dehydrogenase electron transfer subunit	COG0543HC							No	45.4391791	0.042910448	1177
-	SSA_1244	hypothetical protein	-							No	42.04936709	0.294936709	2
cpsY	SSA_1245	LysR family transcriptional regulator	COG0583K	1.139534884	1.046511628	0.837209302				No	36.67983444	-0.21854305	1140
-	SSA_1246	hypothetical protein	-	0.934782609	1.195652174	1.717391304				No	26.48273684	-0.59873684	28
-	SSA_1247	hypothetical protein	COG1332L	0.947368421	1.368421053	2.236842105				No	26.20164021	-0.6968254	82
-	SSA_1248	hypothetical protein	COG1567L	1.029411765	1.558823529	2.588235294				No	34.1536755	-0.34701987	64
-	SSA_1249	hypothetical protein	COG1337L	1.176470588	1.911764706	3.117647059	0.466458	1.891827	0.068109	No	26.15762712	-0.51779661	182

-	SSA_1250	hypothetical protein	COG1421L	1.225806452	2.193548387	3.612903226				No	40.38693182	-0.86761364	55
-	SSA_1251	HD superfamily hydrolase	COG1353R	2.05	3.85	5.05				No	37.19414379	-0.34013072	151
-	SSA_1252	hypothetical protein	COG5551S	3.083333333	4.75	5.208333333				No	49.89102459	-0.32336066	108
-	SSA_1253	hypothetical protein	COG1343L							No	46.30359551	-0.30224719	154
-	SSA_1254	hypothetical protein	-							No	47.94125	-0.4825	3
-	SSA_1255	hypothetical protein	COG1518L							No	41.28179389	-0.19122137	767
-	SSA_1256	NAD-dependent deacetylase	COG0846K	0.981132075	1.018867925	0.943396226				No	36.11028571	-0.0922449	1684
-	SSA_1257	hypothetical protein	-	1.086206897	1.24137931	1.344827586				No	52.47790514	-0.29051383	65
deoD	SSA_1258	purine nucleoside phosphorylase	COG0813F	1.062992126	1.338582677	1.57480315	0.102331	0.895118		No	33.83905303	0.142424242	1306
punA	SSA_1259	purine nucleoside phosphorylase	COG0005F	1.081632653	1.244897959	1.489795918				No	19.2867037	0.010740741	1428
deoB	SSA_1260	phosphopentomutase	COG1015G	1.062992126	1.25984252	1.527559055	0.273373	0.668249	0.274403	No	29.5471464	-0.30024814	1074
rpiA	SSA_1261	ribose-5-phosphate isomerase A	COG0120G	0.880733945	0.844036697	0.76146789				No	19.23111111	-0.02133333	1750
trmE	SSA_1262	tRNA modification GTPase TrmE	COG0486R	0.982758621	0.810344828	0.75862069				No	39.14945295	-0.19277899	2276
-	SSA_1264	hypothetical protein	COG0596R							No	39.5922449	-0.03102041	39
rplS	SSA_1265	50S ribosomal protein L19	COG0335J	0.588942308		0.25	0.225006	2.694524	0.337376	Yes	27.32913386	-0.28110236	2569
crcB1	SSA_1266	camphor resistance protein CrcB	COG0239D							No	27.91732759	0.903448276	1131
crcB2	SSA_1267	camphor resistance protein CrcB	COG0239D							No	23.97177419	0.880645161	857
aroH	SSA_1268	hypothetical protein	COG1605E							No	42.73563218	-0.35517241	355
-	SSA_1269	hypothetical protein	COG0719O		4.75	5				No	36.93804348	-0.38934783	303
flaV	SSA_1270	flavodoxin	COG0716C	0.48447205	0.322981366	0.167701863				No	15.17205479	0.006849315	1357
-	SSA_1271	hypothetical protein	COG0618R	0.86746988		0.530120482				No	33.15369775	-0.32829582	1143
rpmE2	SSA_1272	50S ribosomal protein L31	COG0254J	0.58986731	0.375150784	0.252110977	0.133362	0.682081	0.169406	No	29.855125	-0.935	2385
-	SSA_1274	hypothetical protein	COG0810M							No	28.41844961	-1.14930233	22
-	SSA_1275	hypothetical protein	-							No	34.9034375	0.34375	1
-	SSA_1276	hypothetical protein	COG1917S	1.035714286	1.071428571	1.142857143				No	28.21979167	-0.1375	116
-	SSA_1277	D-alanyl-D-alanine carboxypeptidase	COG1876M							No	34.20203252	-0.66666667	430
-	SSA_1278	rhodanese-like domain-containing protein	COG0607P		1.694915254					No	43.42211538	-0.5	1630
-	SSA_1279	oxidoreductase	COG0604CR	1.272727273	1.606060606	1.742424242				No	34.6090625	-0.090625	1294
-	SSA_1280	hypothetical protein	COG3760S	1.136363636	1.393939394	1.393939394			0.505059	No	30.66851852	-0.33765432	336
-	SSA_1281	uracil-DNA glycosylase	COG1573L	1.148148148	1.388888889	1.574074074				No	45.22381443	-0.48814433	509
pepV	SSA_1282	dipeptidase PepV	COG0624E	1.100401606	1.152610442	1.164658635				No	26.64786325	-0.2775641	840
nrd	SSA_1283	nitroreductase	COG0778C	0.984693878	1.010204082	0.841836735	0.014838	0.72279	0.324841	No	39.7779902	-0.36323529	1546
-	SSA_1284	hypothetical protein	COG5620S							No	30.63502825	0.054237288	52
-	SSA_1285	hypothetical protein	-							No	17.68544304	-0.42278481	7
-	SSA_1286	hypothetical protein	-							No	40.99751553	-0.11118012	10
-	SSA_1287	hypothetical protein	-							No	28.37875	-0.535	4
-	SSA_1288	hypothetical protein	-							No	61.9038125	-0.4375	4
-	SSA_1289	hypothetical protein	-	1.061538462						No	14.06012658	-0.41898734	10
-	SSA_1291	hypothetical protein	COG5660S	0.845070423	0.85915493	0.887323944	0.005363	0.639595		No	29.42375533	-0.68477952	10
-	SSA_1292	hypothetical protein	-							No	42.33522013	-0.37798742	4
-	SSA_1293	hypothetical protein	-			1.255813953				No	44.0038961	-0.40476191	4
-	SSA_1294	hypothetical protein	-							No	49.66495496	-0.64864865	3
-	SSA_1296	hypothetical protein	-							No	38.3679021	-0.7041958	3
uvrC	SSA_1297	excinuclease ABC subunit C	COG0322L	1.074074074	1.148148148	1.148148148				No	41.08693944	-0.4797054	2571
malX	SSA_1298	maltose/maltodextrin ABC transporter sugar-binding protein Ma	COG2182G	2.041420118	3.023668639	3.230769231	0.019886	0.227288		No	27.56578947	-0.42464115	825
malF	SSA_1299	maltose/maltodextrin ABC transport system	COG1175G	1.977272727	3.340909091	4.795454545				No	26.54469027	0.657079646	852
-	SSA_1300	maltose ABC transporter permease	COG3833G	2	3.347826087	4.956521739				No	17.1892446	0.720143885	1027
-	SSA_1301	hypothetical protein	COG4372S							No	34.16166471	-0.38522861	16
trmD	SSA_1302	tRNA (guanine-N(1)-)-methyltransferase	COG0336J	1	0.805194805	0.532467532				Yes	50.30875	-0.5075	2568
rimM	SSA_1303	16S rRNA-processing protein RimM	COG0806J	1.068181818	0.992424242	0.696969697	0.981386	0.657724		No	35.41401163	-0.34127907	1972
-	SSA_1304	hypothetical protein	-	1.163793103	1.155172414	0.948275862				No	50.80666667	-0.665	39
-	SSA_1305	hypothetical protein	-	1.101010101	1.161616162	0.929292929				No	62.88282609	-0.53315217	24
trkA2	SSA_1306	Trk transporter NAD+ binding protein-K+ transport	COG0569P	0.927272727						No	32.19773756	0.302262443	1196
trkH2	SSA_1307	Trk transporter membrane-spanning protein-K+ transport	COG0168P	1.051282051	1	0.974358974				No	27.12383074	0.71091314	1084
-	SSA_1308	hypothetical protein	-							No	55.38142077	-0.47650273	11
-	SSA_1309	RNA-binding protein	COG1837R	0.70528109	0.439522998	0.32197615	1.199652			No	26.88101266	-0.04050633	980
rpsP	SSA_1310	30S ribosomal protein S16	COG0228J	0.614849188	0.364269142	0.26450116	0.261495	0.715163	0.016683	Yes	36.20566667	-0.69444444	2562
-	SSA_1311	HAD superfamily hydrolase	COG1011R	0.775		0.575				No	33.78803279	-0.24808743	1080
-	SSA_1312	RADC-like protein	COG2274V							No	34.88798701	-0.33603896	33
-	SSA_1313	hypothetical protein	COG3708S							No	37.44440433	-0.25270758	726
-	SSA_1314	Fe-S-cluster oxidoreductase	COG0727R							No	40.97439024	-0.56219512	210
-	SSA_1315	hypothetical protein	-							No	31.14906542	-0.55093458	18
-	SSA_1316	nicotinamide mononucleotide transporter	COG3201H							No	22.86623574	0.274904943	187
-	SSA_1317	hypothetical protein	-	1.351351351	1.27027027	0.972972973				No	17.17708333	0.952083333	4
lepA	SSA_1318	GTP-binding protein LepA	COG0481M	0.921875	0.796875					No	34.08868852	-0.12393443	2569
-	SSA_1319	hypothetical protein	COG0561R	0.842105263		0.368421053				No	47.05686347	-0.11771218	404
-	SSA_1320	hypothetical protein	-							No	52.56446281	-0.75867769	52
hemH	SSA_1321	ferrochelatase	COG0276H							No	73.57753425	-0.39671233	1583
-	SSA_1322	glycosyl transferase family protein	COG1819GC	1.172413793	1.224137931	1.103448276				No	42.7475644	-0.19063232	172
-	SSA_1323	hypothetical protein	-	1.106060606	1.196969697	1.196969697				No	41.60818182	-0.26636364	18
-	SSA_1324	ceramide glucosyltransferase	COG1215M	1.090909091	1.181818182	1.159090909				No	40.84376263	0.177020202	43
-	SSA_1325	ketoacyl reductase hetN	COG0300R							No	44.25117188	0.10859375	245
pepT	SSA_1326	peptidase T	COG2195E	1.049586777	1.033057851	0.975206612				No	30.90862408	-0.34914005	1032
-	SSA_1327	hypothetical protein	-	1.135416667	1.21875	1.427083333				No	35.08688525	-0.43333333	4
-	SSA_1328	hypothetical protein	-							No	38.27125	-0.7	23
-	SSA_1329	hypothetical protein	-	1.06122449	1.081632653	1.163265306				No	35.95753425	-0.06678082	6
-	SSA_1330	hypothetical protein	-							No	45.61419355	-0.56193548	6
-	SSA_1331	hypothetical protein	-							No	49.74292237	-0.53789954	32
-	SSA_1332	hypothetical protein	-	1	0.846153846					No	33.10992958	-0.69788732	5
-	SSA_1333	hypothetical protein	-							No	39.78306452	-0.46209677	38
-	SSA_1334	hypothetical protein	-							No	28.08240506	-0.50696203	8
-	SSA_1335	ankyrin repeat-containing protein	COG0666R			0.653846154				No	34.39752475	-0.19009901	65
-	SSA_1336	ankyrin repeat-containing protein	COG0666R	0.791666667						No	30.95082873	-0.31160221	134
-	SSA_1337	hypothetical protein	-							No	47.45778894	-0.13467337	2
-	SSA_1338	hypothetical protein	-							No	39.74114286	-0.14342857	4
phpA	SSA_1339	histidine triad protein D	-							No	46.1957917	-0.69915326	36
-	SSA_1340	Zn/Mn ABC transporter	COG0803P							No	43.47310897	-0.39391026	332
carB	SSA_1341	carbamoyl phosphate synthase large subunit	COG0458EF							No	38.58566572	-0.09301228	2379
carA	SSA_1342	carbamoyl phosphate synthase small subunit	COG0505EF							No	34.71546392	-0.20902062	2370
pyrB	SSA_1343	aspartate carbamoyltransferase	COG0540F							No	42.30918033	-0.27540984	2381
pyrP	SSA_1344	xanthine/uracil permease	COG2233F							No	29.86419204	0.937470726	1618

pyrR	SSA_1345	bifunctional pyrimidine regulatory protein PyrR/uracil phosphoribo	COG2065F							No	25.93081395	-0.27034884	1299
-	SSA_1346	hypothetical protein	-	0.875		0.464285714				No	30.51819048	0.76	194
phnA	SSA_1347	PhnA protein	COG2824P	0.785714286	0.571428571	0.380952381				No	31.93189655	-0.54913793	1248
-	SSA_1348	hypothetical protein	-							No	26.17770115	1.084482759	191
-	SSA_1349	biotin repressor family transcriptional regulator	COG1827R							No	43.14795322	-0.32280702	420
-	SSA_1350	hypothetical protein	COG1051F							No	57.48258065	-0.54258065	921
-	SSA_1352	hypothetical protein	-	1.333333333	1.291666667	1.333333333				No	33.93581081	0.691891892	8
-	SSA_1353	hypothetical protein	COG3797S							No	43.61287293	-0.44530387	342
-	SSA_1354	hypothetical protein	COG0456R							No	39.53163636	-0.18060606	923
-	SSA_1355	hypothetical protein	-							No	22.59383721	-0.28023256	5
uvrB	SSA_1356	excinuclease ABC subunit B	COG0556L	0.722222222		0.5				No	40.14637462	-0.43761329	2602
-	SSA_1357	hypothetical protein	-		1.6					No	35.52352381	0.820952381	218
-	SSA_1358	hypothetical protein	-							No	31.24841935	0.989354839	123
-	SSA_1359	arginine/histidine ABC transporter permease	COG0765E	0.941176471	0.781512605	0.756302521				No	27.23462604	-0.03518006	1333
-	SSA_1360	arginine/histidine ABC transporter ATPase	COG1126E	1.149758454	1.178743961	1.144927536				No	24.16304878	-0.18048781	1679
-	SSA_1361	ORFA, transposon ISSsa2	COG2963L							No	42.98876404	-1.05617978	254
-	SSA_1362	ORFB, transposon ISSsa2	COG2801L							No	46.3099505	-0.70792079	1076
-	SSA_1363	FmtA-like protein	COG1680V							No	26.43293919	-0.06469595	53
-	SSA_1364	hypothetical protein	-							No	27.20446494	0.187822878	1
-	SSA_1365	FmtA-like protein	COG1680V							No	36.51047619	-0.35365079	206
-	SSA_1366	alkaline D-stereospecific endopeptidase	COG1680V							No	28.87472527	-0.01604396	1
-	SSA_1367	hypothetical protein	-							No	1.92745098	-0.28235294	1
-	SSA_1368	hypothetical protein	COG1680V							No	17.32201835	-0.08623853	1
-	SSA_1369	FmtA-like protein	COG1680V							No	29.20470588	-0.10957983	280
-	SSA_1370	CAAX amino protease	COG1266R							No	38.82549839	0.401286174	61
-	SSA_1371	FmtA-like protein	COG1680V							No	20.73389545	-0.08937605	276
-	SSA_1372	hypothetical protein	-							No	43.91269841	0.076190476	1
-	SSA_1373	ABC transporter ATPase	COG0488R							No	32.31043307	-0.47322835	84
-	SSA_1374	multidrug ABC transporter permease/ATPase	COG1132V	1	1.137931034	1.137931034				No	37.32512111	0.205190311	151
-	SSA_1375	multidrug ABC transporter permease/ATPase	COG1132V							No	35.12810345	0.165689655	168
pmrB	SSA_1376	transporter	COG2814G	1.075471698	1.018867925	0.867924528				No	32.55144304	0.635696203	271
asnC	SSA_1377	asparaginyl-tRNA synthetase	COG0017J	0.918367347						No	39.72973214	-0.35022321	1586
-	SSA_1378	hypothetical protein	COG0346E	0.714285714	0.510204082	0.346938776				No	35.70675	-0.30333333	462
-	SSA_1379	hypothetical protein	-	1.137055838	0.893401015	0.756345178				No	41.7887931	-0.81896552	1
-	SSA_1380	hypothetical protein	-	1.135338346	0.864661654	0.706766917				No	46.37411168	-0.33045685	1
-	SSA_1381	hypothetical protein	-	0.927083333	0.677083333	0.46875				No	35.54244604	-0.4647482	1
-	SSA_1382	hypothetical protein	-							No	42.92229299	-0.59745223	2
aspB	SSA_1383	aspartate aminotransferase	COG0436E	0.833333333						No	28.91888041	-0.07862595	1726
-	SSA_1384	hypothetical protein	COG5353S	0.740740741		0.333333333	0.049675	1.188144	0.964715	No	24.13271605	-0.18024691	260
-	SSA_1385	multiple antibiotic resistance operon transcription repressor Ma	COG1846K							No	32.76756757	-0.31621622	1419
-	SSA_1386	NADPH-dependent FMN reductase	COG0431R	1.670682731	1.827309237	1.895582329	1.254658	1.173297	0.064992	No	36.79450549	0.107692308	494
-	SSA_1387	hypothetical protein	COG4495S	1.523809524	1.428571429	1.619047619				No	43.11515152	-0.3010101	34
-	SSA_1388	hypothetical protein	-		1.285714286	1.428571429				No	24.76685083	0.201657459	1
-	SSA_1389	hypothetical protein	-							No	32.92116667	0.334444444	1
-	SSA_1390	hypothetical protein	-							No	30.42124224	-0.38571429	6
-	SSA_1391	hypothetical protein	-							No	42.96380952	-0.23476191	2
-	SSA_1392	hypothetical protein	COG1579R	1	0.964285714	0.892857143				No	34.26316759	-0.50976059	16
-	SSA_1393	hypothetical protein	-	1	0.92					No	46.84537815	-1.29159664	8
-	SSA_1394	hypothetical protein	-	0.961538462	0.846153846					No	46.60879121	-0.5	13
-	SSA_1395	hypothetical protein	COG3022S							No	38.26615702	-0.1785124	1048
-	SSA_1396	hypothetical protein	-							No	44.23520619	0.765463918	106
-	SSA_1397	hypothetical protein	-	4.466257669	6.018404908	5.705521472				No	40.87407407	-0.34166667	229
hlyIII	SSA_1398	hemolysin III-like protein	COG1272R	4.279329609	6.910614525	7.463687151				No	23.47470588	0.87239819	1574
-	SSA_1399	hypothetical protein	COG4720S		0.363636364	0.295454545				No	33.07287234	0.917021277	172
pdxK	SSA_1400	pyridoxamine kinase	COG2240H	0.526315789	0.315789474	0.228070175				No	39.03548611	0.019097222	351
-	SSA_1401	GntR family transcriptional regulator	COG1167KE							No	49.20617312	-0.44031891	1494
-	SSA_1402	multidrig ABC transporter ATPase/permease	COG1132V							No	43.83956822	0.071329879	1568
-	SSA_1403	multidrug ABC transporter permease/ATPase	COG1132V							No	34.82725862	0.044482759	904
-	SSA_1404	hypothetical protein	COG2315S							No	35.46636771	-0.57309417	251
cutC	SSA_1405	copper homeostasis protein CutC	COG3142P							No	34.04075829	0.044549763	842
-	SSA_1406	histone acetyltransferase HPA2-like acetyltransferase	COG1247M							No	46.42493007	-0.53356643	91
-	SSA_1408	hypothetical protein	-	0.808510638		0.553191489				No	38.83065327	0.231155779	27
rmlB	SSA_1409	dTDP-glucose-4,6-dehydratase	COG1088M	1.067278287	1.152905199	1.226299694				No	27.48936782	-0.51666667	2005
rmlC	SSA_1410	dTDP-4-keto-6-deoxyglucose-3,5-epimerase	COG1898M	1.066037736	1.08490566	1.056603774	0.437439	0.51751	0.162632	No	15.30964467	-0.42538071	1511
rmlA	SSA_1411	glucose-1-phosphate thymidylyltransferase	COG1209M	1.025316456	1.012658228	0.966244726		0.589337		No	40.19726644	-0.23114187	1803
-	SSA_1412	hypothetical protein	-							No	31.76363636	-0.65757576	1
-	SSA_1413	GufA-like protein	COG0428P	0.952941176	1.082352941	1.047058824				No	34.00259124	0.850364964	977
-	SSA_1414	hypothetical protein	COG1051F							No	39.86217949	-0.78269231	228
-	SSA_1415	oxidoreductase	COG0665E	0.873015873	0.888888889	0.920634921				No	38.02532967	-0.18406593	1034
-	SSA_1416	hypothetical protein	COG0327S	0.764705882						No	32.01701887	-0.02641509	1936
-	SSA_1417	SAM-dependent methyltransferase	COG2384R		0.575	0.475				No	56.40266376	-0.29781659	668
-	SSA_1418	glutathione S-transferase	COG0625O	1.213333333	1.34	1.386666667	0.703019			No	26.69160305	-0.16564886	867
dnaD	SSA_1419	hypothetical protein	COG3935L	1.261146497	1.27388535	1.229299363	0.243812	0.274854		Yes	40.49207965	-0.33539823	480
metA	SSA_1420	homoserine O-succinyltransferase	COG1897E	1.126760563	1.112676056	1.084507042				No	46.99335463	-0.37891374	904
apt	SSA_1421	adenine phosphoribosyltransferase	COG0503F	0.79787234		0.510638298	0.332693	0.732029	0.328046	No	30.29176471	0.041764706	2093
-	SSA_1422	hypothetical protein	-	1.236363636	1.163636364	1.054545455				No	39.48421053	-0.30526316	1
recJ	SSA_1423	single-stranded DNA-specific exonuclease, 5'-3'	COG0608L	1.051282051	1.025641026	1.153846154				No	33.59190541	-0.15986487	2212
-	SSA_1424	hydrolase	COG1896R	1.290909091	1.563636364	1.527272727				No	34.86020408	-0.34795918	357
-	SSA_1428	hypothetical protein	COG2227H	1.307692308	1.673076923	1.846153846				No	36.0784322	-0.19449153	924
-	SSA_1429	hypothetical protein	COG0300R	1.225806452	1.5	1.806451613				No	30.57928287	0.014741036	796
elaC	SSA_1430	ribonuclease Z	COG1234R	1.184615385	1.461538462	1.676923077				Yes	21.00970874	-0.21909385	1591
-	SSA_1431	hypothetical protein	COG4468G	1.266666667	1.577777778	1.822222222				No	47.6784141	-0.2814978	134
hflX	SSA_1432	HflX GTPase	COG2262R	1.122807018	1.228070175	1.280701754				No	36.64203884	-0.33592233	2309
miaA	SSA_1433	tRNA delta(2)-isopentenylpyrophosphate transferase	COG0324J	0.973684211						No	40.5442517	-0.38503401	2503
-	SSA_1434	hypothetical protein	-	1.136407301	1.192122959	0.856868396				No	46.91964286	-0.09464286	135
-	SSA_1435	hypothetical protein	-							No	39.92163399	-0.29411765	5
-	SSA_1436	hypothetical protein	COG2378K							No	50.31532468	-0.35487013	502
-	SSA_1438	acetyltransferase	COG0456R	0.892857143	1	1.107142857				No	44.55134615	-0.19871795	862
hisK	SSA_1439	hypothetical protein	COG1387ER							No	42.66706827	-0.41526104	220
hisE	SSA_1440	phosphoribosyl-ATP pyrophosphatase	COG0140E	0.766666667	0.933333333	1.066666667				No	42.60961538	-0.63846154	982
hisI	SSA_1441	phosphoribosyl-AMP cyclohydrolase	COG0139E	0.75	1.071428571	1.392857143				No	43.31578947	-0.44035088	2033

hisF	SSA_1442	imidazole glycerol phosphate synthase subunit HisF	COG0107E				0.300839			No	26.30793651	0.091269841	2056
hisA	SSA_1443	1-(5-phosphoribosyl)-5-[(5-phosphoribosylamino)methylidenea	COG0106E							No	19.09752066	0.038016529	2019
hisH	SSA_1444	imidazole glycerol phosphate synthase subunit HisH	COG0118E							No	35.95942029	0.087439614	2053
hisB	SSA_1445	imidazoleglycerol-phosphate dehydratase	COG0131E		1.227272727					No	32.04329897	-0.20051546	2055
hisD	SSA_1446	histidinol dehydrogenase	COG0141E							No	47.42395349	-0.12488372	2044
hisG	SSA_1447	ATP phosphoribosyltransferase	COG0040E							No	33.11214953	0.039719626	2043
hisZ	SSA_1448	ATP phosphoribosyltransferase	COG3705E							No	42.21246201	-0.3006079	685
hisC	SSA_1449	histidinol-phosphate aminotransferase	COG0079E							No	34.74860795	-0.07698864	2033
-	SSA_1450	hypothetical protein	-			1.545454545				No	42.68019802	-0.52178218	79
rexA	SSA_1451	exonuclease RexA	COG1074L	1.076923077	1.269230769	1.5				No	46.27732026	-0.41176471	906
rexB	SSA_1452	second subunit of major exonuclease	COG3857L	0.981132075	1.056603774	1.113207547				Yes	40.32754803	-0.33000915	590
-	SSA_1453	hypothetical protein	-							No	54.33495146	-0.68834952	50
-	SSA_1454	hypothetical protein	-	1.090909091	1.227272727	1.257575758				No	30.82775801	-0.4797153	64
-	SSA_1455	hypothetical protein	COG4283S	1.109090909	1.145454545	1.2				No	33.73666667	-0.87611111	167
-	SSA_1456	hypothetical protein	-	1.047619048	1.095238095	0.880952381				No	50.73205128	-0.35641026	53
dexB	SSA_1457	neopullulanase	COG0366G							No	36.71746575	-0.38476027	1005
-	SSA_1458	hypothetical protein	COG3173R	1.129032258	1.225806452					No	28.77945205	-0.5130137	164
-	SSA_1459	RNA methyltransferase	COG2265J							No	33.26210526	-0.17828947	1693
-	SSA_1460	LytR family transcriptional regulator	COG1316K	0.980392157	1.019607843	0.993464052				No	33.66770601	-0.6752784	304
pheA	SSA_1462	prephenate dehydratase	COG0077E	1.096774194	1.193548387	1.301075269				No	32.31083032	-0.15776173	2125
aroK	SSA_1463	shikimate kinase	COG0703E	1.028571429	1.038095238	1.038095238	0.134753	0.421262		No	43.10949367	-0.28924051	2085
aroA	SSA_1464	3-phosphoshikimate 1-carboxyvinyltransferase	COG0128E	0.857142857	0.816326531	0.734693878				No	34.33325527	-0.07096019	2426
-	SSA_1465	hypothetical protein	COG3679S	1.023809524	1.057142857	0.90952381	0.780664	0.210512	0.022051	No	57.90535714	-0.425	457
tyrA	SSA_1466	prephenate dehydrogenase	COG0287E	1.162790698	1.395348837	1.565891473				No	41.86986413	-0.1576087	1554
aroC	SSA_1467	chorismate synthase	COG0082E	1.043478261	1.210144928	1.282608696				No	35.98634021	-0.2757732	2455
aroB	SSA_1468	3-dehydroquinate synthase	COG0337E	0.990196078	1.058823529	1.009803922				No	30.12140845	-0.05323944	2335
aroE	SSA_1469	shikimate 5-dehydrogenase	COG0169E	0.949367089	0.810126582	0.772151899				No	38.64507042	-0.01232394	2454
aroD	SSA_1470	3-dehydroquinate dehydratase	COG0710E	0.979166667	0.84375	0.71875				No	41.54093333	-0.24133333	731
-	SSA_1471	hypothetical protein	COG1092R	0.744186047		0.337209302	0.383277	0.648526		No	29.42767442	-0.25891473	1462
-	SSA_1472	hypothetical protein	-							No	21.77142857	-0.125	1
-	SSA_1473	hypothetical protein	COG1566V							No	36.98253968	-0.03968254	1
-	SSA_1474	lipoprotein	-							No	37.92633929	-0.48392857	2
-	SSA_1475	hypothetical protein	-							No	40.46666667	0.06875	1
-	SSA_1476	phosphoglycerol transferase alkaline phosphatase superfamily	COG1368M	1.172413793	1.120689655	1.163793103				No	30.4425855	-0.2497948	760
-	SSA_1477	transposase	COG3464L							No	33.83125	-0.36160714	56
-	SSA_1478	transposase	-							No	29.75490196	-1.04E-16	7
-	SSA_1479	transposase	COG3464L							No	48.42923445	-0.43397129	395
-	SSA_1480	hypothetical protein	COG0596R							No	31.56607774	-0.36325088	1518
-	SSA_1481	FmtA-like protein	COG1680V							No	26.37359532	-0.15652174	287
pulI	SSA_1482	pullulanase	COG1523G	1.269736842	1.539473684	1.848684211				No	35.93898039	-0.49267974	393
-	SSA_1483	lipid kinase	COG1597IR	1.275229358	1.605504587	1.95412844				No	22.02816456	-0.14208861	903
ligA	SSA_1484	NAD-dependent DNA ligase LigA	COG0272L	1.224299065	1.551401869	1.682242991				Yes	41.20245399	-0.24110429	2577
ccl	SSA_1485	citrulline cluster-linked gene	COG4708S		0.4	0.4				No	14.94339181	0.802923977	401
mesH	SSA_1486	hypothetical protein	COG2246S							No	20.46232877	0.552054795	252
-	SSA_1487	hypothetical protein	COG3382S							No	43.34595745	-0.33914894	434
-	SSA_1488	multiple antibiotic resistance operon transcription repressor Ma	COG1846K							No	49.61610738	-0.45302013	377
-	SSA_1489	hypothetical protein	-							No	34.37738095	-0.67589286	4
-	SSA_1490	ribonuclease BN	COG1295S							No	45.12118812	0.597689769	946
map	SSA_1491	methionine aminopeptidase	COG0024J	0.888888889	0.759259259	0.62962963				Yes	34.66280702	-0.27684211	2695
-	SSA_1492	hypothetical protein	COG4109K	0.965517241						No	43.95846868	-0.07842227	526
-	SSA_1493	hypothetical protein	COG1670J							No	28.58196721	-0.3989071	360
murZ	SSA_1494	UDP-N-acetylglucosamine 1-carboxyvinyltransferase	COG0766M		0.441176471	0.323529412				No	33.54322196	0.064916468	688
metK	SSA_1495	S-adenosylmethionine synthetase	COG0192H	0.945205479	0.835616438	0.712328767				Yes	28.55631313	-0.22222222	2439
-	SSA_1496	hypothetical protein	-	1.228070175						No	29.08328671	0.565034965	5
comEB	SSA_1497	dCMP deaminase	COG2131F	0.942307692		0.538461538				No	29.23935484	-0.11290323	1061
rplT	SSA_1498	50S ribosomal protein L20	COG0292J	0.955645161	0.802419355	0.689516129	0.275444	0.509325	0.720027	Yes	47.50672269	-0.45966387	2580
rpmI	SSA_1499	50S ribosomal protein L35	COG0291J	0.906862745	0.767156863	0.713235294				No	43.53939394	-1.18636364	1989
infC	SSA_1500	translation initiation factor IF-3	COG0290J	0.781144781		0.336700337	0.495522	0.312086	0.430805	Yes	29.77386364	-0.575	2548
cmk	SSA_1501	cytidylate kinase	COG0283F	0.958333333	1.035714286	0.922619048				No	29.38977778	-0.29066667	2355
-	SSA_1502	hypothetical protein	-	1.005319149	1.026595745	0.920212766				No	42.18489655	-0.22551724	124
fer	SSA_1503	ferredoxin	COG1141C							No	25.66432836	-0.2238806	167
-	SSA_1504	hypothetical protein	-	0.867924528	0.811320755	0.710691824				No	29.45185185	0.193209877	107
-	SSA_1505	hypothetical protein	COG1368M	1.164383562	1.232876712	1.328767123				No	36.14967933	0.063776722	337
rgpFc	SSA_1506	lipopolysaccharide biosynthesis protein	COG3754M	0.988505747	0.988505747	1				No	41.54068345	-0.34892086	125
rgpD	SSA_1507	sugar ABC transporter ATPase	COG1134GM	1.060344828	0.922413793	0.853448276				No	29.33643392	-0.38379052	1429
rgpC	SSA_1508	sugar ABC transporter permease	COG1682GM	1.013157895	0.921052632	0.842105263				No	23.6130597	0.842164179	1184
rgpB	SSA_1509	polysaccharide biosynthesis protein/ rhamnosyltransferase	COG0463M	0.848214286						No	31.5588746	-0.43376206	442
rgpA	SSA_1510	rhamnosyltransferase	COG0438M	0.797979798						No	23.59191099	-0.40471204	282
-	SSA_1511	glycosyltransferase	COG0438M	1.215053763	1.559139785	1.774193548				No	41.58554217	-0.3026506	612
-	SSA_1512	hypothetical protein	COG4713S	1.082352941	1.317647059	1.447058824				No	29.45552311	0.670316302	110
rgpF	SSA_1513	glycosyltransferase	COG3754M	0.986842105	1.092105263	1.157894737				No	41.19600201	-0.37683787	332
-	SSA_1514	cell-wall biogenesis glycosyltransferase	COG0463M	0.941176471	0.926470588	0.882352941				No	39.38189189	-0.4034749	55
-	SSA_1515	hypothetical protein	COG2456S							No	42.30672269	0.9	121
-	SSA_1516	cell-wall biogenesis glycosyltransferase	COG1216R	0.915492958	0.788732394	0.661971831				No	43.6042735	-0.0982906	992
cpsIaJ	SSA_1517	cell-wall biogenesis glycosyltransferase	COG0463M	0.8		0.428571429				No	30.47928793	-0.24613003	835
rgpE	SSA_1518	glycosyl transferase family protein	COG0463M							No	24.60387302	-0.13079365	483
-	SSA_1519	polysaccharide/teichoic acid transporter	COG2244R							No	31.47051887	0.771698113	107
tuf	SSA_1520	elongation factor Tu	COG0050J				0.452703	1.278351	0.674877	Yes	37.37188442	-0.2959799	2765
ppc	SSA_1521	phosphoenolpyruvate carboxylase	COG2352C	1.168949772	1.237442922	1.242009132				No	42.95264768	-0.34345992	1291
ftsW	SSA_1522	cell division protein FtsW	COG0772D	0.892473118	0.731182796	0.548387097				Yes	33.33463415	0.708536585	2060
basA	SSA_1523	glutathione peroxidase	COG0386O							No	47.31019108	-0.54140127	1406
-	SSA_1525	1,4-beta-N-acetylmuramidase	COG3757M	0.757281553		0.40776699				No	45.20319298	-0.58	201
-	SSA_1526	hypothetical protein	COG2035S							No	24.8637931	0.928965517	575
ebsC	SSA_1527	hypothetical protein	COG2606S	2.153846154	2.153846154		0.240049	0.532006		No	37.09006211	-0.40062112	1495
gpm	SSA_1528	phosphoglycerate mutase	COG0406G	1.941176471	1.823529412	1.823529412				No	32.10189573	-0.45687204	582
lysS	SSA_1529	lysyl-tRNA synthetase	COG1190J	0.705426357	0.434108527	0.341085271	0.147622	0.379166	0.219281	Yes	36.86108871	-0.39939516	2416
-	SSA_1530	peptide ABC transporter permease	COG0577V	1.056179775	1.08988764	1.08988764				No	32.66018957	0.238862559	1108
salX	SSA_1531	peptide ABC transporter ATPase	COG1136V	1.02	1.02	0.98		16.4582		No	40.64698276	-0.20603448	1972
acrA	SSA_1532	membrane-fusion protein / periplasmic component of efflux syst	COG0845M	0.893333333	0.826666667	0.773333333				No	21.26614987	-0.33359173	1102
gor	SSA_1533	glutathione reductase	COG1249C	1.424657534	1.287671233	1.136986301				No	24.74444444	-0.10662768	1081
-	SSA_1535	hypothetical protein	COG1878R							No	47.80082305	-0.35432099	627

bioY	SSA_1536	biotin synthase	COG1268R							No	39.2321547	1.093922652	1237
-	SSA_1537	hypothetical protein	COG4443S	1.08		0.673333333				No	34.68	-0.55857143	275
-	SSA_1538	hypothetical protein	-	0.954198473	0.832061069	0.847328244				No	50.39152542	-0.60084746	77
-	SSA_1540	hypothetical protein	COG4357S	0.640776699	0.436893204	0.349514563				No	54.94017094	-0.48888889	179
-	SSA_1541	U32 family peptidase	COG0826O	0.647058824		0.359477124				No	44.98955711	-0.33240093	1584
-	SSA_1542	U32 family peptidase	COG0826O		0.333333333	0.222222222				No	41.22544892	-0.26191951	283
-	SSA_1543	hypothetical protein	-	0.741482966						No	67.461	-0.034	119
-	SSA_1544	hypothetical protein	-	1.242320819	1.276450512	1.245733788	0.186464			No	48.216875	-0.865625	122
-	SSA_1545	hypothetical protein	COG4768R	1.145833333	1.034722222	0.861111111				No	12.01515152	0.356818182	319
lgt	SSA_1546	prolipoprotein diacylglyceryl transferase	COG0682M	0.918918919	0.736486486	0.587837838				No	42.94065134	0.552873563	2476
ptsK	SSA_1547	HPr kinase/phosphorylase	COG1493T	0.8046875	0.5703125	0.375				No	42.35051447	-0.27202572	1031
-	SSA_1548	NTP pyrophosphohydrolases including oxidative damage repair	COG1051F							No	34.62883436	-0.43803681	628
-	SSA_1549	hypothetical protein	COG1983KT	0.975	0.775	0.55				No	46.86234694	0.273469388	228
-	SSA_1550	hypothetical protein	COG3091S	1.260416667	1.25	1.333333333				No	54.54965517	-0.73241379	418
tex	SSA_1551	transcriptional accessory ribonuclease (YqgFc, S1)	COG2183K	0.928571429	0.857142857	0.857142857				No	36.63183099	-0.15746479	1668
-	SSA_1552	hypothetical protein	-							No	59.77777778	-1.46888889	51
-	SSA_1553	permease	COG0701R	1.291262136	1.291262136	1.087378641				No	28.12192691	0.948172757	902
-	SSA_1554	hypothetical protein	COG3689S	1.429906542	1.345794393	1.280373832				No	24.68786765	-0.03125	306
gdh	SSA_1555	glucose-6-phosphate 1-dehydrogenase	COG0364G	0.947761194		0.537313433				Yes	42.51997942	-0.44032922	1860
ftsY	SSA_1557	SRPR, signal recognition particle-docking protein	COG0552U	1.135802469	1.413580247	1.635802469				Yes	42.71582031	-0.50371094	2647
-	SSA_1558	HAD superfamily hydrolase	COG0561R	1.073333333	1.293333333	1.426666667				No	34.01838235	-0.21029412	394
-	SSA_1559	hypothetical protein	COG0561R	1.048387097	1.14516129	1.193548387				No	25.85685606	-0.04734849	550
smc	SSA_1560	structural maintenance of chromosome protein (chromosome s	COG1196D	0.905172414	0.853448276	0.844827586				No	47.81240238	-0.55526316	1854
rnc	SSA_1561	ribonuclease III	COG0571K		0.285714286	0.196428571				No	28.80689655	-0.36810345	2554
-	SSA_1562	hypothetical protein	COG2832S	1.15503876	1.23255814	1.046511628				No	27.11428571	0.772268908	453
vicX	SSA_1563	beta-lactamase superfamily hydrolase	COG1235R	1.083769634	1.068062827	0.879581152				No	32.72150376	-0.3962406	1098
-	SSA_1564	histidine kinase	COG5002T	1.049751244	1.004975124	0.830845771				No	33.57040089	-0.23608018	2401
-	SSA_1565	two-component response transcriptional regulator	COG0745TK	0.941704036	0.775784753	0.520179372				Yes	35.86609442	-0.31545064	2319
-	SSA_1566	polar amino acid ABC transporter ATP-binding protein	COG1126E		0.293103448	0.275862069	0.035264	0.686651	1.108357	No	19.343083	-0.10197629	913
-	SSA_1567	polar amino acid ABC transporter amino acid-binding protein	COG0834ET		0.393442623					No	24.60222222	-0.25962963	1391
-	SSA_1568	arginine/histidine ABC transporter permease	COG0765E							No	24.44876106	0.698230088	565
-	SSA_1569	arginine/histidine ABC transporter permease	COG0765E	0.824324324	0.783783784	0.945945946				No	33.84977169	0.803196347	637
-	SSA_1570	hypothetical protein	-	1	0.931034483	1.034482759				No	20.39318182	-0.55606061	9
thrS	SSA_1571	threonyl-tRNA synthetase	COG0441J	1.235294118	1.171122995	1.085561497		0.607366		Yes	33.49307573	-0.53925811	2745
-	SSA_1572	hypothetical protein	COG0237H	0.708860759	0.430379747	0.253164557				No	55.26762846	-0.34387352	28
-	SSA_1573	hypothetical protein	-	1.240740741						No	31.4520625	0.22875	1
-	SSA_1574	glycosyl transferase family protein	COG0438M	1.075949367	1.012658228	0.835443038				No	33.99047727	-0.05386364	2137
cpoA	SSA_1575	glycosyl transferase family protein	COG0438M	1.428571429	1.528571429	1.185714286				No	41.72635204	-0.0494898	482
ccpA	SSA_1576	catabolite control protein A	COG1609K	2.546255507	3.330396476	2.845814978	0.463876	0.567901		No	28.58532934	-0.29431138	1428
pepQ	SSA_1577	proline dipeptidase	COG0006E	1.247933884	1.553719008	1.801652893				No	26.08447222	-0.14388889	1036
-	SSA_1578	Fe3+-siderophore ABC transporter permease	COG0609P							No	24.25721264	1.05	1787
-	SSA_1579	Fe3+-siderophore ABC transporter ATPase	COG1120PH							No	25.83346614	-0.21713147	1015
fatB	SSA_1581	metal-binding ABC transporter	COG0614P							No	35.03274854	-0.41345029	1068
-	SSA_1582	alpha-beta hydrolase superfamily esterase	COG2819R							No	34.71613043	-0.35608696	84
ogt	SSA_1583	6-O-methylguanine DNA methyltransferase	COG0350L				0.653218			No	41.44311377	-0.19341317	2320
-	SSA_1584	ring-cleavage extradiol dioxygenase	COG2514R							No	37.63826087	-0.36335404	604
-	SSA_1585	hypothetical protein	COG2910R							No	30.67177033	-0.03349282	655
-	SSA_1586	hypothetical protein	COG1959K	0.794871795		0.435897436				No	28.674	0.032666667	726
-	SSA_1587	hypothetical protein	COG0622R	0.766666667		0.566666667				No	47.31631206	-0.34361702	485
-	SSA_1588	peptide ABC transporter permease	COG0577V	1.108695652	1.173913043	1.152173913				No	34.54984565	-0.10463065	477
-	SSA_1589	peptide ABC transporter ATPase	COG1136V							No	40.44038627	-0.19828326	659
-	SSA_1590	TetR/AcrR family transcriptional regulator	COG1309K							No	51.67048387	-0.38064516	407
-	SSA_1591	dipeptidase	COG4690E	3.666666667	7.888888889	9.333333333				No	34.44648094	-0.35175953	38
-	SSA_1592	hypothetical protein	COG2339S							No	25.64142857	0.63992674	21
-	SSA_1593	dipeptidase	COG4690E							No	39.33122744	-0.45902527	61
-	SSA_1594	metalloendopeptidase	COG3590O							No	34.09380261	-0.70275762	303
tehB	SSA_1595	tellurite resistance protein TehB	COG3615P							No	43.15195122	-0.43484321	871
-	SSA_1596	hypothetical protein	-		0.541666667	0.5				No	31.00626959	-0.42570533	1
-	SSA_1597	hypothetical protein	-							No	44.7478882	-0.69968944	2
-	SSA_1598	hypothetical protein	-							No	43.14525478	-0.72643312	1
-	SSA_1599	hypothetical protein	-				0.140179	0.364094	1.187266	No	31.89845679	-0.66635803	5
-	SSA_1600	hypothetical protein	-							No	20.034375	0.009375	1
smpB	SSA_1601	SsrA-binding protein	COG0691O	1.028169014						No	41.15483871	-0.83935484	2569
vacB	SSA_1602	VacB/Rnb family exoribonuclease	COG0557K	1.021276596	0.914893617	0.936170213				No	35.47778772	-0.52659847	2240
-	SSA_1603	hypothetical protein	-							No	56.07347826	-0.53429952	7
secG	SSA_1604	preprotein translocase subunit SecG	COG1314U	0.704545455		0.386363636				Yes	29.14285714	0.927272727	350
pmrA	SSA_1605	major facilitator superfamily multi-drug resistance efflux pump P	COG2814G	1.076086957	1.195652174	1.130434783				No	34.29173442	0.685365854	1609
coaE	SSA_1606	dephospho-CoA kinase	COG0237H	1.019607843	1.205882353	1.323529412				Yes	50.82575758	-0.36414141	2467
mutM	SSA_1607	formamidopyrimidine-DNA glycosylase	COG0266L	1.044247788	1.159292035	1.230088496				No	43.26284672	-0.33357664	1955
-	SSA_1608	hypothetical protein	COG1051F	1.057471264	1.195402299	1.402298851				No	29.94557692	-0.29102564	1189
-	SSA_1610	hypothetical protein	COG3177S	0.891304348	0.967391304	1				No	37.31528239	-0.38305648	982
era	SSA_1611	GTP-binding protein Era	COG1159R	0.855555556	0.9	0.744444444				No	27.58327759	-0.2735786	2424
dgk	SSA_1612	diacylglycerol kinase	COG0818M	1.125		0.671875				No	26.18257576	0.635606061	1298
-	SSA_1613	metalloprotease	COG0319R	1.016393443	0.819672131	0.459016393				No	46.46848485	-0.5969697	2301
-	SSA_1614	acetyltransferase	COG1670J							No	41.80904762	-0.4968254	615
-	SSA_1615	alanine dehydrogenase	COG0686E	3.533333333		6.366666667	0.201314	0.435274	0.377017	No	28.01378378	0.135405405	1771
phoH	SSA_1616	PhoH-like protein	COG1702T	1.048192771	0.891566265	0.698795181				No	37.02933333	-0.05633333	2219
-	SSA_1617	hypothetical protein	COG4479S	1.166666667	1.104166667	1.0625				No	34.1156338	-0.75774648	386
-	SSA_1618	hypothetical protein	COG2996S	1.102564103		0.794871795				No	22.22644366	-0.40211268	793
frr	SSA_1619	ribosome recycling factor	COG0233J	0.902097902		0.559440559	0.152234	0.516624	0.114228	Yes	35.93945946	-0.50324324	2582
pyrH	SSA_1620	uridylate kinase	COG0528F	0.893401015	0.69035533	0.52284264	0.888149	4.270677	0.264632	Yes	27.24190871	-0.0593361	2649
-	SSA_1621	amino acid transporter	COG0531E							No	26.0378882	0.589026915	504
rplA	SSA_1622	50S ribosomal protein L1	COG0081J	0.54251497	0.274251497	0.189221557	0.572563	0.686131	0.365206	Yes	8.530567686	-0.16375546	2728
rplK	SSA_1623	50S ribosomal protein L11	COG0080J	0.534775889	0.214837713	0.132921175	0.272389	0.497908	0.466195	Yes	42.48723404	-0.01276596	2746
-	SSA_1624	hypothetical protein	-							No	32.41756522	1.108695652	117
-	SSA_1625	lactoylglutathione lyase	COG0346E							No	52.45632813	-0.61875	692
ftsK	SSA_1626	DNA translocase ftsK	COG1674D	0.838709677						No	33.38681462	-0.05326371	2369
-	SSA_1627	hypothetical protein	COG1983KT	0.684210526		0.421052632				No	25.27833333	0.736666667	801
-	SSA_1628	MutT/nudix family protein	COG0494LR							No	55.70215827	-0.4028777	216
ppiA	SSA_1629	cyclophilin type peptidyl-prolyl cis-trans isomerase	COG0652O	0.609756098	0.390243902	0.243902439				No	31.79301471	-0.58566177	874
-	SSA_1630	hypothetical protein	-	0.855345912		0.433962264				No	71.46666667	-0.57307692	73

srtC	SSA_1631	sortase	COG3764M	1.058823529	1.088235294	1.196078431				No	35.47898305	-0.18474576	426
-	SSA_1632	surface protein	COG4932M	1.101694915	1.076271186	1.194915254				No	22.14592902	-0.22881002	121
-	SSA_1633	FimA fimbrial subunit-like protein	COG4932M	0.860215054	0.709677419	0.731182796				No	23.54908142	-0.21085595	29
-	SSA_1634	Heme utilization/adhesion exoprotein	COG4932M	0.686746988						No	25.16152263	-0.32078189	15
-	SSA_1635	hypothetical protein	-	0.491803279	0.295081967	0.245901639				No	32.95734398	-0.48853411	10
-	SSA_1636	antibiotic ABC transporter ATPase	COG0488R	0.887323944	0.830985915	0.85915493				No	35.10725146	-0.37699805	459
-	SSA_1638	hypothetical protein	COG4269S	0.838461538		0.453846154				No	7.883333333	0.382352941	117
pfs	SSA_1639	5'-methylthioadenosine/S-adenosylhomocysteine nucleosidase	COG0775F	0.771653543		0.448818898				No	21.85478261	0.189565217	1547
-	SSA_1640	hypothetical protein	-	1.025751073	0.939914163	0.793991416				No	76.62642857	-0.66142857	114
-	SSA_1641	MutT/nudix family protein	COG0494LR	0.93258427	0.814606742					No	34.76398907	-0.5147541	1968
glmU	SSA_1642	bifunctional N-acetylglucosamine-1-phosphate uridyltransferase	COG1207M	0.932960894	0.726256983	0.497206704				Yes	23.05424837	-0.18082789	2457
-	SSA_1643	hypothetical protein	-	0.584070796	0.407079646	0.292035398				No	42.78684932	-0.73607306	1
-	SSA_1644	hypothetical protein	-							No	26.46666667	-0.32708333	1
-	SSA_1645	hypothetical protein	COG1695K							No	35.88317308	-0.32644231	154
-	SSA_1646	hypothetical protein	COG4405S							No	25.79060403	-0.31342282	486
-	SSA_1647	hypothetical protein	-							No	31.37565217	-0.64782609	16
-	SSA_1648	hypothetical protein	COG1247M							No	47.11255061	-0.2951417	131
-	SSA_1649	hypothetical protein	COG0584C							No	31.49202454	-0.44110429	1131
fabG	SSA_1650	3-ketoacyl-ACP reductase	COG1028IQR							No	25.70521552	0.060775862	390
-	SSA_1651	hypothetical protein	-							No	29.775	-0.32386364	164
-	SSA_1652	acetyltransferase	COG1670J							No	28.31048387	-0.39274194	136
-	SSA_1653	hypothetical protein	-							No	32.88846875	-0.724375	4
-	SSA_1655	hypothetical protein	COG3153R							No	40.35758865	-0.19432624	164
-	SSA_1656	nisin resistance protein	COG0793M							No	30.7797619	-0.38720238	575
-	SSA_1657	hypothetical protein	-							No	58.81910112	0.224719101	10
-	SSA_1658	cationic amino acid transporter	COG0531E							No	25.83137339	0.789055794	1841
-	SSA_1659	peptide ABC transporter permease	COG3127Q							No	33.30383686	0.517522659	400
-	SSA_1660	peptide ABC transporter ATPase	COG1136V							No	33.57628459	-0.14664032	519
-	SSA_1661	hypothetical protein	-							No	54.35802469	-0.66790124	3
-	SSA_1662	NADH-dependent oxidoreductase	COG1902C							No	46.94481013	-0.2678481	662
cpbA	SSA_1663	collagen-binding protein A	COG4932M	0.875717017	0.950286807	1.036328872				No	18.55937128	-0.51211119	220
-	SSA_1664	phosphatidylethanolamine N-methyltransferase	COG2226H							No	24.48093496	-0.40609756	959
-	SSA_1665	hypothetical protein	-							No	44.30465116	0.769767442	1
-	SSA_1666	collagen-binding surface protein	COG4932M							No	27.42048062	-0.35162791	95
-	SSA_1667	hypothetical protein	-							No	52.98360656	-0.76885246	2
-	SSA_1668	NrdI protein	COG1780F				0.477961			No	36.69316239	-0.21965812	62
-	SSA_1669	hypothetical protein	-							No	32.12695652	1.513913043	3
-	SSA_1670	TetR/AcrR family transcriptional regulator	COG1309K							No	46.04832512	-0.44827586	624
-	SSA_1671	hypothetical protein	-							No	39.68741325	-0.06309148	16
-	SSA_1672	hypothetical protein	-							No	8.75625	1.40625	1
-	SSA_1673	hypothetical protein	-							No	22.86964286	0.183928571	1
-	SSA_1675	hypothetical protein	-							No	49.59178082	-0.21232877	1
-	SSA_1676	LytTr family transcriptional regulator	COG3279KT							No	29.8410596	-0.19933775	207
cylB	SSA_1678	multidrig ABC transporter permease	COG1511S							No	28.0847331	0.672241993	172
cylA	SSA_1679	multidrig ABC transporter ATPase	COG1131V							No	30.04652778	-0.20833333	529
-	SSA_1680	antibiotic ABC transporter permease	-							No	27.65481982	0.334084084	133
-	SSA_1681	antibiotic ABC transporter ATPase	COG1136V							No	31.90540541	-0.16177606	405
-	SSA_1682	hypothetical protein	-							No	31.86944223	0.696812749	85
-	SSA_1683	NrdI protein	COG1780F	1.172839506	1.407407407	1.530864198				No	49.83658537	-0.71788618	125
-	SSA_1684	histidine kinase	COG0642T	1.206896552	1.431034483	1.431034483				No	29.19785276	0.010736196	553
-	SSA_1685	two-component response transcriptional regulator	COG0745TK	1.016666667	1.116666667	1.1				No	30.58377193	-0.05394737	490
-	SSA_1686	hypothetical protein	COG3319Q	0.833333333		0.5625				No	39.44876238	-0.33935644	200
-	SSA_1687	NADH-binding ferric-oxidoreductase	COG4097P	1.227272727	1.090909091	1.204545455				No	25.23851759	0.209547739	879
-	SSA_1689	hypothetical protein	-	0.787313433		0.406716418	3.836153			No	50.62473118	-0.60376344	10
-	SSA_1690	hypothetical protein	-	2.5625	3.479166667	3.458333333				No	14.47028571	0.770285714	11
-	SSA_1691	XRE family transcriptional regulator	COG3655K	2.164835165	3.10989011	3.010989011				No	13.60869565	0.162318841	586
lacG	SSA_1692	6-phospho-beta-galactosidase	COG2723G	3.666666667	13	34.16666667				No	33.91438034	-0.55064103	334
lacE	SSA_1693	phosphotransferase system lactose-specific component IIBC	COG1455G	8.4	26.2	55.8				No	28.53292254	0.508802817	367
lacF	SSA_1694	PTS system lactose-specific transporter subunit IIA	COG1447G	14.5	44.5	85.75				No	40.67238095	-0.02190476	284
-	SSA_1695	BglG family transcriptional antiterminator	COG3711K	20.66666667	50.66666667	71.33333333				No	43.30833935	-0.44909747	150
lacD	SSA_1696	tagatose 1,6-diphosphate aldolase	COG3684G	1	1.807692308	3.192307692				No	39.26349693	-0.33680982	266
lacC	SSA_1697	tagatose-6-phosphate kinase	COG1105G	1.142857143	2.285714286	5.047619048				No	24.2602589	0.030420712	439
lacB	SSA_1698	galactose-6-phosphate isomerase subunit LacB	COG0698G	1.166666667	2.791666667	5.75	5.097782	0.192402		No	26.42105263	-0.20760234	1275
lacA	SSA_1699	galactose-6-phosphate isomerase subunit LacA	COG0698G	1.368421053	3	5.842105263				No	22.15034965	0.045454545	465
-	SSA_1700	hypothetical protein	-							No	14.75675676	0.9	1
lacR	SSA_1701	sugar metabolism transcriptional repressor	COG1349KG							No	32.66494024	-0.30079681	444
-	SSA_1702	hypothetical protein	-							No	28.93824228	-0.63111639	27
metS	SSA_1703	methionyl-tRNA synthetase	COG0143J	1.042372881	0.889830508					Yes	30.74297297	-0.27432432	2740
-	SSA_1704	hypothetical protein	COG2323S			0.458333333				No	34.3447619	0.341428571	728
-	SSA_1705	hypothetical protein	-	0.422222222	0.244444444	0.244444444				No	29.34715232	0.041059603	107
exoA	SSA_1706	exodeoxyribonuclease	COG0708L	1.263157895	0.973684211	0.894736842	0.822438			No	34.94285714	-0.36242236	2125
-	SSA_1707	hypothetical protein	COG0346E	1.436363636						No	44.11335938	-0.20859375	178
-	SSA_1708	hypothetical protein	-				0.479638			No	8.524390244	0.52195122	1
-	SSA_1709	hypothetical protein	-							No	21.11515152	-0.04242424	1
-	SSA_1710	XRE family transcriptional regulator	COG1476K							No	49.84624365	0.185786802	435
nth	SSA_1711	endonuclease III	COG0177L	0.831168831		0.61038961				No	46.7861244	-0.18277512	2607
-	SSA_1712	arsenate reductase	COG1393P	0.767857143		0.535714286				No	18.43846154	-0.37777778	982
serA	SSA_1713	D-3-phosphoglycerate dehydrogenase	COG0111HE	0.861111111		0.638888889				No	33.52736573	-0.11943734	717
serC	SSA_1715	phosphoserine aminotransferase	COG1932HE			0.548387097	0.305948	0.266324		No	47.78267218	-0.19779614	1556
-	SSA_1716	restriction endonuclease SsuRB	-	0.872340426		0.382978723				No	39.72597403	-0.35	112
spnIM	SSA_1717	modification methylase DpnIIB	COG0863L		0.529411765	0.323529412				No	39.40749064	-0.55543071	1256
dam	SSA_1718	site-specific DNA-methyltransferase	COG0338L			0.5				No	38.28881119	-0.55629371	887
-	SSA_1719	hypothetical protein	COG0313R	1.075471698	1.113207547	1.113207547				No	38.0000692	-0.23079585	2509
-	SSA_1720	DNA replication intiation control protein YabA	COG4467S	0.980582524						No	40.00295238	-0.77238095	442
holB	SSA_1721	DNA polymerase III subunit delta'	COG0470L	0.886075949	0.797468354	0.835443038				Yes	63.03280405	-0.27162162	695
tmk	SSA_1722	thymidylate kinase	COG0125F	0.702702703		0.418918919				Yes	33.1759434	-0.14198113	2429
-	SSA_1723	hypothetical protein	COG1284S	0.461538462	0.323076923	0.246153846				No	22.64952542	0.66779661	807
-	SSA_1724	hypothetical protein	COG0517R	2.260869565	4.108695652	5.326086957				No	31.20458716	0.03853211	1158
livF	SSA_1725	branched chain amino acid ABC transporter ATP-binding protei	COG0410E	1.105263158	1.210526316	1.184210526				No	32.15762712	-0.03347458	1849
livG	SSA_1726	branched chain amino acid ABC transporter ATP-binding protei	COG0411E	1.012820513	1.128205128	1.038461538	0.313653	1.343624	0.549431	No	29.41496063	0.000787402	1632
livM	SSA_1727	branched-chain amino acid ABC transporter permease	COG4177E	1	0.983333333	0.966666667				No	26.06154574	1.038485804	1436

livH	SSA_1728	branched-chain amino acid ABC transporter permease	COG0559E	0.909090909						No	22.65224913	1.080622837	1439
livJ	SSA_1729	branched-chain amino acid ABC transporter substrate-binding	COG0683E	0.589473684	0.352631579	0.252631579	0.244464	0.504909	0.386202	No	24.49041451	-0.23963731	1355
-	SSA_1730	hypothetical protein	COG4471S		0.326923077	0.211538462				No	30.89438202	-0.73820225	300
clpP	SSA_1731	ATP-dependent Clp protease proteolytic subunit	COG0740OU	1.143236074	1.063660477	0.870026525	0.146016			No	28.96173469	-0.11530612	2509
upp	SSA_1732	uracil phosphoribosyltransferase	COG0035F	0.81595092			0.2111	0.189902	0.184628	No	33.68851675	0.122009569	2109
-	SSA_1733	hypothetical protein	-							No	60.63235294	0.255882353	3
-	SSA_1734	cation (Mg/Ni uptake) transport ATPase	COG0474P	1.127659574	1.404255319	1.574468085				No	36.59764108	0.248871332	689
-	SSA_1735	UreX	COG0625O							No	48.62955665	-0.36699507	525
-	SSA_1736	L-cysteine desulfhydrase	COG1168E	1.098039216	1.235294118	1.31372549				No	31.40850515	-0.35412371	1091
metB	SSA_1737	cystathionine gamma-synthase	COG0626E	1.042553191	1.021276596	1.021276596				No	38.0294026	-0.10051948	1436
-	SSA_1738	polysaccharide biosynthesis protein	COG2244R	1.06741573	1.056179775	0.887640449				Yes	34.42107011	0.78597786	607
murE	SSA_1739	UDP-N-acetylmuramoylalanyl-D-glutamate--L-lysine ligase	COG0769M	1.123595506	1.123595506	1.011235955				Yes	32.66972973	-0.13700624	2402
-	SSA_1740	hypothetical protein	-							No	33.07632184	0.507662835	65
-	SSA_1741	Fe3+-siderophores ABC transporter ATPase	COG1120PH							No	25.22950192	-0.03409962	1546
-	SSA_1742	ferrichrome-binding protein	COG0614P							No	31.03350482	-0.33440515	944
fhuB	SSA_1743	Fe3+-siderophore ABC transporter permease	COG0609P							No	23.62713147	0.997609562	257
-	SSA_1744	iron ABC transporter permease	COG0609P							No	36.34523952	0.69251497	651
csbD	SSA_1745	general stress response protein CsbD	COG3237S	1.595876289	1.711340206	1.443298969				No	14.3609375	-0.6234375	141
-	SSA_1746	hypothetical protein	-			0.592592593				No	49.62124424	-0.43824885	124
-	SSA_1747	hypothetical protein	-							No	35.31482353	-0.28470588	129
ppx	SSA_1748	manganese-dependent inorganic pyrophosphatase	COG1227C	0.835497835			2.908333	0.65873	0.223283	Yes	26.46302251	0.083601286	796
pflA	SSA_1749	pyruvate formate-lyase-activating enzyme	COG1180O	4.880952381	6.619047619	4.857142857				No	32.50483271	-0.31821561	1115
-	SSA_1750	extracellular nuclease	COG2374R		1.238095238	1.523809524				No	37.11311081	-0.42736983	421
dexS	SSA_1751	dextran glucosidase	COG0366G							No	28.66044405	-0.45133215	1546
-	SSA_1752	phosphotransferase system, trehalose-specific IIBC component	COG1263G	1.166666667	1.458333333	2	0.101416	0.116652	0.268837	No	34.41937409	0.381804949	697
treR	SSA_1753	GntR family transcriptional regulator	COG2188K							No	33.68067227	-0.59537815	659
-	SSA_1754	hypothetical protein	-	1.097560976	1.329268293	1.817073171				No	64.42051282	-1.1982906	2
-	SSA_1755	hypothetical protein	-	1.021126761	1.26056338	1.654929577				No	51.31649485	-0.23402062	6
-	SSA_1756	hypothetical protein	-	1	1.228070175	1.570175439				No	37.64488095	-0.4890873	4
-	SSA_1757	hypothetical protein	-	1.089552239	1.432835821	1.71641791				No	49.71935644	-0.32376238	2
-	SSA_1758	hypothetical protein	-							No	29.25153465	-0.33069307	4
-	SSA_1759	hypothetical protein	-							No	35.59613861	-0.73861386	1
-	SSA_1760	hypothetical protein	-							No	45.11365385	0.486538462	1
hlyX	SSA_1761	hemolysin	COG1253R	0.701492537		0.335820896				No	39.60941704	0.014798206	2539
-	SSA_1762	permease	COG0628R	1.158415842	1.297029703	1.405940594				No	31.64946524	0.777005348	591
-	SSA_1763	molybdenum ABC transporter ATPase	COG1119P				0.442222			No	34.85079545	-0.20454546	911
-	SSA_1764	hypothetical protein	-	1.233333333	1.266666667	1.366666667				No	31.30775	0.435769231	22
-	SSA_1765	hypothetical protein	COG2226H	1.275	1.3					No	33.51854331	-0.51889764	515
-	SSA_1766	bacitracin ABC transporter permease	COG1277R							No	27.65149798	0.82145749	68
-	SSA_1767	bacitracin ABC transporter ATP-binding protein	COG1131V				1.590847			No	29.20662461	-0.36246057	399
-	SSA_1768	TetR/AcrR family transcriptional regulator	COG1309K							No	36.10520202	-0.1979798	155
-	SSA_1769	hypothetical protein	COG2265J	0.884057971						No	25.92404372	-0.04535519	645
-	SSA_1770	hypothetical protein	COG1242R			0.459459459				No	33.58320635	-0.45809524	1112
-	SSA_1771	DNA methyltransferase protein	COG3695L							No	28.44607843	-0.14411765	855
-	SSA_1772	hypothetical protein	COG0671I	1.170731707		0.780487805				No	36.98342593	0.522222222	1728
-	SSA_1773	hypothetical protein	COG3601S	1.1875		0.666666667				No	29.25410811	1.084324324	538
cspR	SSA_1774	rRNA methylase	COG0219J							No	43.04027174	-0.48641304	2051
trkA	SSA_1775	potassium transporter peripheral membrane protein	COG0569P	0.921296296	0.74537037					No	29.47305122	0.012694878	1369
trkH	SSA_1776	Trk family potassium uptake protein	COG0168P	1.268041237	1.316151203	1.436426117				No	27.19039666	0.865553236	1082
rluB	SSA_1777	ribosomal large subunit pseudouridine synthase B	COG1187J	1.09929078	1.255319149	1.177304965				No	24.43347107	-0.55165289	2210
scpB	SSA_1778	segregation and condensation protein B	COG1386K	1.091743119	1.23853211	1.229357798				No	44.73968254	-0.10740741	1681
scpA	SSA_1779	segregation and condensation protein A	COG1354S	1.040650407	1.056910569	1.06504065				No	37.48220339	-0.20211864	1575
xerD	SSA_1780	site-specific tyrosine recombinase XerD	COG4974L	1.141732283	1.11023622	1.078740157				No	45.53621399	-0.34279835	179
-	SSA_1781	hypothetical protein	COG4109K	1.046666667	0.946666667	0.826666667				No	32.90660131	-0.09215686	414
-	SSA_1782	hypothetical protein	COG0622R	1.06993007	0.923076923	0.804195804				No	34.37121387	-0.37456647	887
-	SSA_1783	deoxyribonucleotide triphosphate pyrophosphatase/unknown do	COG0127F	1.065217391	0.876811594	0.710144928				No	27.58083832	-0.41736527	2486
murI	SSA_1784	glutamate racemase	COG0796M	0.92	0.77	0.62				Yes	42.11405303	0.007954545	2113
-	SSA_1786	hypothetical protein	COG3763S	0.680232558	0.581395349	0.337209302		0.658512		No	46.22926829	-0.10487805	286
lysA	SSA_1787	diaminopimelate decarboxylase	COG0019E	0.890410959	0.780821918					No	29.22211538	-0.21081731	2286
-	SSA_1788	integral membrane protein, receptor	COG0670R	1.359649123	1.495614035	1.495614035				No	28.06660793	0.927753304	1479
-	SSA_1789	HD superfamily metal-dependent phosphohydrolase	COG1418R	1.424242424	1.676767677	1.555555556				No	32.41478528	-0.66441718	263
-	SSA_1790	SpoU rRNA methylase family protein	COG0566J	1.095238095			0.005005	0.083973		No	37.13780488	0.038211382	1659
acyP	SSA_1791	acylphosphatase	COG1254C							No	23.30326087	-0.09782609	1065
yidC	SSA_1792	OxaA-like protein precursor	COG0706U	0.720720721						No	41.45472313	0.033224756	410
-	SSA_1793	histidine kinase (sensor protein)	COG0642T	1.216216216	1.378378378	1.540540541				No	30.41442822	-0.17080292	227
-	SSA_1794	two-component response transcriptional regulator	COG0745TK							No	38.94358974	-0.17478633	500
-	SSA_1795	guanosine 3',5'-bis-pyrophosphate (ppGpp) synthetase	COG2357S							No	36.80339806	-0.60097087	198
greA	SSA_1796	transcription elongation factor GreA	COG0782K	0.733727811		0.331360947	0.345875	0.811422	0.495876	No	31.33375	-0.395625	2488
pabC	SSA_1797	aminodeoxychorismate lyase	COG1559R	1.037542662	1.081911263	1.109215017				No	38.75052104	-0.47715431	2084
-	SSA_1798	hypothetical protein	COG3153R	1.107981221	1.281690141	1.338028169				No	41.6005848	-0.23508772	90
-	SSA_1799	acetyltransferase	COG3153R	1.043010753	1.125448029	1.168458781				No	30.64058824	-0.12823529	589
murC	SSA_1800	UDP-N-acetylmuramate--L-alanine ligase	COG0773M	1.030042918	1.128755365	1.261802575				Yes	27.41103604	-0.25067568	2460
-	SSA_1801	hypothetical protein	-	0.961538462	0.916083916	0.86013986				No	57.43454545	-1.11454546	121
snf	SSA_1802	Snf2 family protein	COG0553KL	1.098039216	1.098039216	0.931372549				No	44.51164569	-0.31616651	1467
engA	SSA_1803	GTP-binding protein EngA	COG1160R	1.189393939	1.265151515	1.234848485				Yes	29.09270642	-0.16605505	2620
-	SSA_1804	NADPH-flavin oxidoreductase	COG0778C	1.257142857	1.335714286	1.292857143	0.262607	0.04703		No	39.74457983	-0.42310924	1052
dnaI	SSA_1805	primosomal protein DnaI	COG1484L	1.216783217	1.300699301	1.230769231				Yes	42.99063545	-0.43578595	670
dnaB	SSA_1806	replication initiation/membrane attachment protein DnaB	COG3611L	1.152173913	1.043478261	0.934782609				Yes	29.04393401	-0.52106599	253
nrdR	SSA_1807	NrdR family transcriptional regulator	COG1327K	0.8		0.433333333				No	64.64140127	-0.94267516	2034
rgfB	SSA_1808	hypothetical protein	COG3568R	1.314285714	1.6	1.8				No	41.01590406	-0.23800738	230
ptsG	SSA_1809	PTS system glucose-specific EIIC BA component (EIICBA-Glc)	COG1263G	1.291139241	1.506329114	1.556962025				No	25.05013699	0.407123288	1029
csrR	SSA_1810	two-component response transcriptional regulator	COG0745TK	0.969465649	0.721374046	0.553435115				No	30.49868996	-0.08427948	147
gnd	SSA_1811	6-phosphogluconate dehydrogenase	COG0362G	0.857142857		0.421245421	0.444448	0.8	0.351281	Yes	40.52575949	-0.25443038	2000
-	SSA_1812	modification methylase	COG0270L	1.1	1.178571429	1.392857143				No	43.72009804	-0.42083333	800
-	SSA_1813	hypothetical protein	-	1.074074074	1.175925926	1.324074074				No	38.18809061	-0.18381877	2
-	SSA_1814	hypothetical protein	-	1.008403361	1.100840336	1.394957983				No	35.54026549	-0.34690266	8
-	SSA_1815	hypothetical protein	-	0.956896552	1.060344828	1.206896552				No	39.6492233	-0.21488673	4
-	SSA_1816	hypothetical protein	-	0.908333333	1.041666667	1.3				No	33.48481595	-0.36610429	19
-	SSA_1817	hypothetical protein	-	0.789473684	0.905263158	1.094736842				No	38.4679558	-0.44198895	131
-	SSA_1818	very short patch repair endonuclease	COG3727L							No	43.10860927	-0.8615894	505
valS	SSA_1819	valyl-tRNA synthetase	COG0525J	1.024390244	1.056910569	1.008130081				Yes	36.30991011	-0.38842697	2757

-	SSA_1820	hypothetical protein	-							No	35.56249211	0.545741325	3
rimL	SSA_1821	ribosomal protein N-acetylase	COG1670J	0.833333333	0.685185185	0.537037037				No	44.77219251	-0.45080214	1038
-	SSA_1822	hypothetical protein	-	0.71875	0.59375	0.4375				No	40.22142857	-0.75119048	131
-	SSA_1823	hypothetical protein	COG2461S	0.68627451		0.431372549				No	43.61988962	-0.48211921	277
-	SSA_1824	hypothetical protein	-		0.277777778	0.194444444				No	17.51447368	0.269736842	134
-	SSA_1825	hypothetical protein	COG3711K							No	44.67667368	-0.26273684	116
glpK	SSA_1826	glycerol kinase	COG0554C							No	30.76894422	-0.25318725	1946
glp	SSA_1827	alpha-glycerophosphate oxidase	COG0578C							No	36.14789474	-0.31529605	1621
glpF	SSA_1828	glycerol uptake facilitator protein	COG0580G							No	21.57649573	0.866666667	543
-	SSA_1829	RNA methyltransferase	COG2265J	1	1	0.79245283				No	39.62877193	-0.32894737	1037
-	SSA_1830	aminoglycoside phosphotransferase	COG3231J							No	44.81988372	-0.32364341	149
recX	SSA_1831	recombination regulator RecX	COG2137R							No	44.49922481	-0.74922481	874
-	SSA_1832	hypothetical protein	COG3557J	0.756218905	0.432835821	0.243781095				No	39.88079096	-0.65536723	469
-	SSA_1833	hypothetical protein	-	1.263636364	1.018181818	0.890909091				No	28.12095238	-0.06190476	135
raiA	SSA_1834	30S ribosomal interface protein S30EA	COG1544J	2.90989011	4.901098901	7.12967033				No	42.25222222	-0.56055556	1961
comFC	SSA_1835	late competence protein	COG1040R							No	48.56153846	-0.40633484	1248
comFA	SSA_1836	superfamily II ATP-dependent DNA/RNA helicase	COG4098L				0.377822	0.356575		No	41.21275463	-0.25092593	448
-	SSA_1837	hypothetical protein	COG1739S							No	24.76619048	-0.21666667	1836
cysK	SSA_1839	cysteine synthase	COG0031E	0.668308703		0.325123153	0.522799	0.372366	0.584823	No	20.41812298	0.018446602	2319
-	SSA_1840	hypothetical protein	COG1098J	1.326241135	1.475177305	1.446808511				No	37.78583333	-0.5175	596
pplB	SSA_1841	Cof family protein/peptidyl-prolyl cis-trans isomerase, cyclophili	COG0652O	1.278350515	1.309278351	1.216494845				No	29.09849785	-0.21266094	1599
-	SSA_1842	two-component response transcriptional regulator	COG2197TK	1.179104478	1.28358209	1.179104478				No	37.83761905	-0.14190476	1428
-	SSA_1843	histidine kinase	COG4585T	1.208333333	1.291666667	1.270833333				No	43.11533923	-0.16578171	1382
-	SSA_1844	hypothetical protein	COG4758S	1.125		0.770833333				No	25.60779221	0.445454545	347
pkn	SSA_1845	serine/threonine protein kinase	COG0515RTKL	1.034722222	1.03125	1.020833333				No	46.07732577	-0.336953	1839
pppL	SSA_1846	phosphoprotein phosphatase	COG0631T	1.035573123	0.916996047	0.794466403	0.067696	0.277401	0.16506	No	33.21869919	-0.3995935	1503
sunL	SSA_1847	rRNA methyltransferase RsmB	COG0144J	1.067567568	1.135135135	1.148648649				No	46.62366197	-0.21774648	2118
fmt	SSA_1848	methionyl-tRNA formyltransferase	COG0223J	0.986842105	1.092105263	1.157894737				Yes	33.02090032	-0.06398714	2546
priA	SSA_1849	primosome assembly protein PriA	COG1198L	0.930232558	0.976744186	1				No	41.67900629	-0.39358491	2450
rpoZ	SSA_1850	DNA-directed RNA polymerase subunit omega	COG1758K	0.560240964	0.325301205	0.168674699	0.185429	0.154096	0.640021	No	63.65192308	-0.925	614
gmk	SSA_1851	guanylate kinase	COG0194F	0.410447761	0.201492537	0.089552239				Yes	33.44142857	-0.39428571	2515
-	SSA_1852	hypothetical protein	COG1418R	0.826714801		0.281588448				No	37.27839851	-0.50614525	1109
luxS	SSA_1853	S-ribosylhomocysteine lyase	COG1854T	0.831168831		0.506493506		0.640209		No	39.78962264	-0.2745283	1070
-	SSA_1854	hypothetical protein	COG4372S	1.070175439	0.988304094	0.98245614	0.058482	0.336406	0.675497	No	45.27703934	-0.69337474	147
-	SSA_1855	hypothetical protein	COG0116L	0.940594059	0.782178218	0.732673267				No	27.64401042	-0.29453125	1520
-	SSA_1856	hypothetical protein	-	0.872340426		0.542553191				No	54.57906977	-0.47209302	1
-	SSA_1857	DivIVA domain-containing protein	COG3599D	0.616216216		0.275675676				No	46.64	-0.68416667	415
-	SSA_1858	hypothetical protein	COG4474S					0.357827		No	19.86748571	-0.54628571	416
recU	SSA_1859	Holliday junction-specific endonuclease	COG3331R	0.985714286		0.671428571				No	44.22155	-0.395	527
pbp1A	SSA_1860	penicillin-binding protein 1A	COG0744M	1.006535948	0.823529412	0.816993464				No	33.63858345	-0.50182328	1149
pepC	SSA_1861	aminopeptidase	COG3579E	1.220657277	1.248826291	1.164319249				No	30.23831081	-0.42657658	417
-	SSA_1862	hypothetical protein	COG1670J	0.982608696	1.052173913	1.052173913				No	39.46026882	-0.25698925	886
nadE	SSA_1863	NAD synthetase	COG0171H	1	1.032258065	1.032258065				Yes	36.25948905	-0.28029197	1677
pncB	SSA_1864	nicotinate phosphoribosyltransferase	COG1488H	0.8875	0.8875	0.9125				Yes	32.50432099	-0.31481482	1561
-	SSA_1865	thioredoxin reductase	COG0492O	1.026086957	0.869565217	0.686956522	0.065259	0.233502	0.351308	Yes	35.12138158	-0.15888158	2720
-	SSA_1866	hypothetical protein	-	0.844444444		0.511111111				No	36.33513514	0.954054054	129
-	SSA_1867	polar amino acid ABC transporter ATPase	COG1126E	1.076923077	0.756410256	0.628205128	0.535837			No	32.95060729	-0.23846154	223
-	SSA_1868	arginine/histidine ABC transporter permease	COG0765E	1		0.528301887				No	31.17164179	0.618656716	494
rheB	SSA_1869	ATP-dependent RNA helicase	COG0513LKJ	1.040540541	0.905405405	0.783783784				No	31.04675615	-0.49574944	516
mraY	SSA_1870	phospho-N-acetylmuramoyl-pentapeptide-transferase	COG0472M	1.216814159	1.296460177	1.362831858				Yes	20.42486239	0.86146789	2536
pbpX	SSA_1871	penicillin-binding protein 2X	COG0768M	0.960674157	0.876404494	0.859550562				Yes	27.89566929	-0.41272966	2428
ftsL	SSA_1872	cell division protein	COG4839D	0.6	0.47826087	0.391304348	0.41166			Yes	49.26831776	-0.57383178	232
mraW	SSA_1873	S-adenosyl-methyltransferase MraW	COG0275M	0.518987342	0.303797468	0.202531646				No	39.50664557	-0.47025317	2584
yorfE	SSA_1874	hypothetical protein	COG1476K							No	46.22394366	-0.73380282	768
-	SSA_1875	hypothetical protein	-							No	48.49556818	0.221022727	11
-	SSA_1876	hypothetical protein	-							No	49.41622222	0.300555556	61
-	SSA_1877	hypothetical protein	-							No	43.30862069	0.689655172	8
-	SSA_1878	hypothetical protein	COG4224S	1.151815182	1.303630363	1.330033003				No	54.50470588	-1.25764706	515
glyS	SSA_1879	glycyl-tRNA synthetase subunit beta	COG0751J	1.123762376	1.193069307	1.227722772				Yes	31.85861561	-0.13166421	1664
glyQ	SSA_1880	glycyl-tRNA synthetase subunit alpha	COG0752J	0.970149254	0.910447761	0.843283582				Yes	39.39344262	-0.45770492	1791
-	SSA_1881	hypothetical protein	-	0.55	0.3	0.233333333				No	39.21901186	-0.39841897	32
prtS	SSA_1882	subtilisin-like serine proteases	COG1404O	0.914893617	1.021276596	1.234042553				No	27.97798805	-0.4312749	940
-	SSA_1883	hypothetical protein	-	1.123188406	1.166666667	1.398550725				No	34.86128099	-0.47603306	24
-	SSA_1884	hypothetical protein	-	1.132867133	1.230769231	1.468531469	0.228738	0.223736	0.179115	No	40.18767123	-0.40228311	16
-	SSA_1888	hypothetical protein	-	1.04	1.08	1.02				No	39.92189711	-0.50578778	39
-	SSA_1889	hypothetical protein	-							No	38.33691756	-0.57777778	14
-	SSA_1890	acetyltransferase	COG0456R	1.106382979	1.244680851	1.117021277				No	45.6434	-0.42333333	202
-	SSA_1891	aldo/keto reductase	COG0656R	1.069306931	1.198019802	0.97029703	0.27022		0.010066	No	24.54483986	-0.24661922	1678
-	SSA_1892	hypothetical protein	-	1.129032258	1.193548387	0.774193548				No	25.15625	0.54375	12
nagA	SSA_1893	N-acetylglucosamine-6-phosphate deacetylase	COG1820G	1.21978022	1.428571429	1.395604396				No	31.79976501	-0.1381201	1589
rbfA	SSA_1895	ribosome-binding factor A	COG0858J	1.042553191	1.042553191		331.07			No	31.39137931	-0.64568966	2170
infB	SSA_1896	translation initiation factor IF-2	COG0532J	1.071428571	1.103896104	1.142857143				No	41.11494624	-0.7227957	2613
-	SSA_1897	hypothetical protein	COG1358J							No	24.23469388	-0.04081633	565
-	SSA_1899	hypothetical protein	COG2740K	0.955752212			0.133119	0.422		No	36.445	-0.6744898	687
nusA	SSA_1900	transcription elongation factor NusA	COG0195K	0.850574713	0.75862069					No	49.49285714	-0.42547619	2628
-	SSA_1901	hypothetical protein	COG0779S	0.745454545	0.636363636	0.509090909				No	39.64237288	-0.19830509	1834
trmB	SSA_1902	tRNA (guanine-N(7)-)-methyltransferase	COG0220R	0.738095238						No	31.55566038	-0.47688679	2230
-	SSA_1903	hypothetical protein	COG0510M	0.666666667					0.073749	Yes	45.08598485	-0.44242424	378
-	SSA_1904	multidrug ABC transporter permease	COG4473U	1.163265306	1.040816327	0.93877551				No	37.12100575	0.659770115	417
-	SSA_1905	multidrug ABC transporter ATPase	COG1131V	0.906976744		0.627906977				No	35.00792453	0.035849057	610
hit	SSA_1906	diadenosine tetraphosphate (Ap4A) hydrolase	COG0537FGR	0.802816901		0.577464789				No	22.78676471	-0.03970588	2674
-	SSA_1907	hypothetical protein	-				0.361756	0.774609		No	44.29368421	-0.51368421	120
-	SSA_1909	LytR family transcriptional regulator	COG1316K	0.835106383						No	33.2973301	-0.32961165	957
-	SSA_1910	hypothetical protein	COG1670J	0.706521739		0.358695652				No	39.01907514	-0.03468208	959
-	SSA_1911	hypothetical protein	COG0802R	0.602040816	0.367346939	0.234693878				No	23.82123288	-0.31575343	2476
-	SSA_1912	hypothetical protein	-							No	22.89714286	0.551428571	1
-	SSA_1913	xanthine/uracil permease family protein	COG2252R							No	19.37378436	0.886680761	1784
-	SSA_1914	hypothetical protein	COG4330S							No	35.43149606	0.701574803	105
-	SSA_1915	hypothetical protein	-							No	23.21851852	0.742592593	1
-	SSA_1916	hypothetical protein	COG0561R	0.686567164		0.373134328				No	26.88896296	-0.4037037	821
adh	SSA_1917	alcohol dehydrogenase	COG1064R	7.647058824	15.35294118	18				No	16.62625369	0.15280236	1721

-	SSA_1918	phosphotransferase system, mannose-specific EIIAB	COG3444G	1.0625	0.875		0.004572	0.499841	0.27245	No	28.05898204	-0.07694611	700
-	SSA_1919	phosphotransferase system, mannose-specific EIIC	COG3715G	1.273584906	1.333333333	1.323899371				No	23.87313433	1.071268657	581
-	SSA_1920	phosphotransferase system, mannose-specific EIID	COG3716G	1.479768786	1.722543353	1.936416185				No	29.64984026	0.298083067	646
-	SSA_1921	hypothetical protein	COG4687S	0.696969697						No	21.43841667	-0.21166667	277
-	SSA_1922	hypothetical protein	COG0456R	1.166666667	1.35	1.366666667				No	24.07290476	-0.07476191	67
-	SSA_1923	NADPH-quinone reductase	COG2249R	1.092307692	1.323076923	1.507692308				No	42.59953125	-0.22447917	259
-	SSA_1924	TetR/AcrR family transcriptional regulator	COG1309K	0.923076923	1.015384615	1.046153846				No	41.49843243	-0.28	180
serS	SSA_1925	seryl-tRNA synthetase	COG0172J	1.085470085	0.923076923	0.888888889	0.648928	0.424951	0.480944	Yes	32.39931765	-0.492	2733
-	SSA_1926	hypothetical protein	-							No	12.68292683	-0.25853659	1
shetA	SSA_1927	exfoliative toxin	COG1275P	0.947019868	1.062913907	1.261589404				No	37.6217377	0.860983607	284
-	SSA_1928	acyl-CoA dehydrogenase	COG1960I	0.75	0.778688525	0.81557377				No	45.36073446	-0.05932203	913
mip	SSA_1929	macrophage infectivity potentiator protein	COG2128S				0.045832	0.301567	0.372207	No	21.41236559	-0.04892473	521
accA	SSA_1930	acetyl-CoA carboxylase subunit alpha	COG0825I	0.932721713	0.905198777					Yes	30.70429688	-0.21054688	1872
accD	SSA_1931	acetyl-CoA carboxylase subunit beta	COG0777I	1.029801325	1.052980132	0.897350993				Yes	21.758223	-0.20348432	2469
accC	SSA_1932	acetyl-CoA carboxylase biotin carboxylase subunit	COG0439I	0.974683544	0.996835443	0.85443038	0.237504	0.409092	1.640307	Yes	31.21035165	-0.05978022	2444
fabZ	SSA_1933	(3R)-hydroxymyristoyl-ACP dehydratase	COG0764I	0.965635739	0.945017182	0.862542955				Yes	28.78221429	0.107857143	2142
accB	SSA_1934	acetyl-CoA carboxylase biotin carboxyl carrier protein subunit	COG0511I	1.056818182	0.96969697	0.946969697				Yes	55.90185185	-0.11296296	2123
fabF	SSA_1935	3-oxoacyl-ACP synthase	COG0304IQ	0.989285714	0.817857143	0.728571429	0.013765	0.415299	0.4992	Yes	33.15439024	-0.19634146	2435
fabG	SSA_1936	3-ketoacyl-ACP reductase	COG1028IQR	0.890070922				0.450505		Yes	24.86393443	0.28852459	2598
fabD	SSA_1937	malonyl-CoA:ACP transacylase	COG0331I	0.87804878		0.479674797	0.035334	0.312855	0.210202	Yes	32.88140523	0.091830065	2348
fabK	SSA_1938	enoyl-acyl carrier protein(ACP) reductase	COG2070R	0.707407407		0.281481481	0.252937	0.681252		Yes	28.91666667	0.040123457	1464
acpP	SSA_1939	acyl carrier protein	COG0236IQ	0.821011673		0.451361868				Yes	61.20675676	-0.13378378	1226
fabH	SSA_1940	3-oxoacyl-ACP synthase	COG0332I	0.71875	0.35625	0.25				Yes	37.22469136	-0.03611111	2299
-	SSA_1941	hypothetical protein	COG1846K	0.704761905	0.319047619	0.204761905				No	55.56635514	-0.5682243	990
phaB	SSA_1942	enoyl-CoA hydratase	COG1024I	0.617647059	0.323529412	0.205882353				No	29.90570342	0.02661597	1880
lysC	SSA_1943	aspartate kinase	COG0527E	0.842105263		0.526315789				No	36.29804348	-0.14565217	2526
-	SSA_1944	oligopeptide transport ATP-binding protein	COG1123R	0.955555556	0.961111111	0.811111111				No	23.48866883	-0.31071429	2095
-	SSA_1945	oligopeptide transport ATP-binding protein	COG0444EP	1.07826087	1.004347826	0.869565217				No	36.6048169	-0.1428169	2257
-	SSA_1946	oligopeptide transport system permease	COG1173EP	1.005952381	0.81547619	0.696428571				No	20.88344156	0.485714286	1424
-	SSA_1947	oligopeptide transport system permease	COG0601EP	0.822695035		0.432624113				No	40.40044177	0.009437751	280
-	SSA_1948	oligopeptide-binding lipoprotein	COG4166E	0.540909091	0.25	0.181818182				No	32.04555219	-0.64886536	355
-	SSA_1949	AliA protein	COG4166E	1.797468354	2.696202532	2.784810127				No	29.65396341	-0.62179878	444
-	SSA_1950	peptide ABC transporter periplasmic protein	COG4166E		0.239130435	0.260869565				No	30.74166922	-0.55313936	330
pbp3	SSA_1951	penicillin-binding protein 3	COG1686M	1.055555556	0.814814815	0.648148148				No	22.09307876	-0.21193317	1453
-	SSA_1952	ABC transporter permease	COG0719O	1.946078431	2.235294118	2.549019608				No	31.16340426	-0.38404255	1767
nifU	SSA_1953	NifU family protein	COG0822C	2.031446541	2.20754717	2.433962264				No	35.95931034	-0.24344828	1952
-	SSA_1954	class V aminotransferase	COG0520E	1.861445783	2.018072289	2.072289157				No	28.98490244	-0.09463415	2377
-	SSA_1955	ABC transporter permease	COG0719O	1.869565217	1.881987578	1.894409938				No	37.49621429	-0.16690476	454
-	SSA_1956	ABC transporter ATPase	COG0396O	1.774011299	1.649717514	1.338983051				No	29.54257813	-0.09765625	2002
rgpG	SSA_1957	glycosyl transferase N-acetylglucosaminyltransferase	COG0472M	1.192982456	1.385964912	1.216374269				No	30.84126289	0.859793814	1687
mecA	SSA_1958	adaptor protein	COG4862OTN	1.075	1.158333333	1.033333333				No	42.31684	-0.3864	419
bacA	SSA_1959	undecaprenyl pyrophosphate phosphatase	COG1968V	0.912280702	0.824561404					No	28.67614286	0.788214286	2093
-	SSA_1960	hypothetical protein	COG4907S	1.090909091	1.060606061	0.939393939				No	39.35107765	-0.06006339	173
-	SSA_1961	amino acid ABC transporter substrate-binding protein	COG0765E			0.458333333				No	24.45662188	0.036660269	1297
-	SSA_1962	amino acid ABC transporter ATP-binding protein	COG1126E							No	27.8947561	-0.33373984	225
-	SSA_1964	hypothetical protein	-	1.055865922	1.041340782	0.748603352	0.129347	0.178249	0.087992	No	19.67760563	-0.54507042	60
-	SSA_1965	stomatin/prohibitin-like membrane protease subunits	COG0330O	0.944444444	1.041666667	0.986111111				No	36.06354839	0.000967742	1895
ilvA	SSA_1967	threonine dehydratase	COG1171E	1.16	1.2	1.32				No	30.10096154	-0.17596154	2092
ilvC	SSA_1968	ketol-acid reductoisomerase	COG0059EH	1.10989011	1.10989011	1.065934066	0.314604	0.310052	0.550238	No	26.62176471	-0.19117647	2146
ilvH	SSA_1969	acetolactate synthase 3 regulatory subunit	COG0440E	0.694915254	0.406779661	0.322033898				No	49.64303797	-0.07974684	1943
ilvB	SSA_1970	acetolactate synthase catalytic subunit	COG0028EH	0.666666667	0.395833333	0.270833333				No	28.5169788	-0.0795053	2166
-	SSA_1971	hypothetical protein	-	0.731707317		0.463414634				No	39.2112931	1.165517241	20
-	SSA_1972	two-component response transcriptional regulator	COG2197TK							No	42.84824121	-0.20703518	564
-	SSA_1973	histidine kinase	COG4585T							No	35.57434426	0.086065574	622
-	SSA_1974	multidrug ABC transporter permease	COG0842V							No	43.18036885	0.850819672	452
-	SSA_1975	multidrug ABC transporter ATPase	COG1131V							No	42.21443299	-0.31271478	328
-	SSA_1976	hypothetical protein	-		0.333333333	0.272727273				No	58.86336634	-0.2980198	6
-	SSA_1978	dihydroxyacetone kinase family protein	COG1461R	1.14556962	1.136075949	0.955696203	0.236521	0.631111	0.237679	No	35.15288809	-0.07906137	909
asp	SSA_1979	alkaline-shock protein	COG1302S	1.08994709	1.031746032	0.698412698				No	14.95867769	0.133884298	633
rpmB	SSA_1980	50S ribosomal protein L28	COG0227J	0.723154362						No	9.201612903	-0.58548387	1897
-	SSA_1981	hypothetical protein	-	1.65	3	3.65				No	14.95181818	0.479545455	161
-	SSA_1982	LytR/AlgR family transcriptional regulator	COG3279KT	1.368421053	2.894736842	4.684210526				No	48.66972973	-0.35743243	269
-	SSA_1984	cell surface SD repeat-containing protein	COG5099J							No	40.00663317	-0.54030151	1
-	SSA_1985	hypothetical protein	COG5406							No	29.39162011	-0.46494413	1
-	SSA_1986	acetyltransferase	COG1246E							No	49.95736842	-0.25460526	122
-	SSA_1987	hypothetical protein	COG3694R							No	32.83452107	0.65440613	411
-	SSA_1988	ABC transporter permease	COG4587R							No	41.43426471	0.916176471	360
-	SSA_1989	ABC transporter ATPase	COG4586R							No	45.67215152	-0.32484849	451
-	SSA_1990	Zn-porter lipoprotein	COG0803P							No	39.86888525	-0.45377049	371
phtA	SSA_1991	histidine triad protein A	-							No	37.15840741	-0.61950617	71
fba	SSA_1992	fructose-bisphosphate aldolase	COG0191G				0.2125	0.899563	0.324103	Yes	28.97133106	-0.11945393	2155
-	SSA_1993	hypothetical protein	COG3708S	1.06010929	1.032786885	0.918032787				No	36.74191176	-0.67279412	175
pyrG	SSA_1994	CTP synthetase	COG0504F	1.034883721	0.918604651	0.802325581				No	31.08901304	-0.20204842	2648
-	SSA_1995	hypothetical protein	COG3382S	0.818181818	0.863636364					No	43.32368421	-0.19912281	42
rpoE	SSA_1996	DNA-directed RNA polymerase subunit delta	COG3343K	0.596810934		0.387243736				No	57.53486188	-1.15027624	435
-	SSA_1997	membrane spanning protein	-							No	26.95309237	0.962650602	6
tig	SSA_1998	trigger factor	COG0544O				0.450622	0.43728	0.191738	No	25.37985948	-0.4028103	2423
-	SSA_1999	hypothetical protein	COG4475S	0.948051948	0.909090909	0.863636364				No	26.46737968	0.29197861	403
-	SSA_2000	hypothetical protein	COG4720S	0.983606557	0.93442623	0.926229508				No	17.5294375	0.7875	283
thiD	SSA_2001	phosphomethylpyrimidine kinase	COG0351H	0.860869565	0.756521739	0.72173913				No	28.99765625	0.095703125	2215
truA	SSA_2002	tRNA pseudouridine synthase A	COG0101J	0.72519084						No	41.28955823	-0.56987952	2632
zmpB	SSA_2004	Zinc metalloprotease zmpB	COG3583S	1.097484277	1.213836478	1.43081761				No	30.38541492	-0.43965336	43
dnaJ	SSA_2005	molecular chaperone DnaJ	COG0484O	0.81754386	0.747368421	0.733333333				Yes	29.3127321	-0.69787798	2664
-	SSA_2006	4-methyl-5(beta-hydroxyethyl)-thiazole monophosphate synthes	COG0693R	0.710691824						No	36.02809524	0.096666667	240
dnaK	SSA_2007	molecular chaperone DnaK	COG0443O				0.5	0.669767	0.587874	Yes	25.79244663	-0.34351396	2677
grpE	SSA_2008	heat shock protein GrpE	COG0576O	0.188183807	0.144420131	0.124726477	0.197329	0.83921	0.182244	No	60.5747191	-0.82247191	2568
hrcA	SSA_2009	heat-inducible transcription repressor	COG1420K	0.284671533	0.199513382	0.155717762				No	34.57619186	-0.08459302	1728
-	SSA_2010	multidrug ABC transporter permease	-							No	39.42900826	0.938016529	39
-	SSA_2011	multidrug ABC transporter ATPase	COG1131V	1.161290323						No	34.70505376	-0.08781362	562
-	SSA_2012	hypothetical protein	-							No	43.80578947	-0.02368421	160
-	SSA_2013	hypothetical protein	COG1695K							No	62.32345794	-0.48504673	751

-	SSA_2014	D-alanyl-D-alanine carboxypeptidase	COG1876M	1.204301075	1.204301075	1				No	44.94885496	-0.42213741	255
-	SSA_2015	phosphoglycerate mutase	COG0406G	1.057471264	1.057471264	0.793103448				No	25.37931034	-0.19568966	508
-	SSA_2016	phosphoglycerate mutase	COG0406G							No	17.59613734	-0.26909871	147
-	SSA_2017	hypothetical protein	COG2194R							No	41.75107383	0.330872483	4
-	SSA_2018	hypothetical protein	COG2848S			0.451612903				No	19.00988764	0.260224719	580
-	SSA_2019	hypothetical protein	COG3830T							No	41.81590909	0.284090909	558
-	SSA_2020	hypothetical protein	COG3087D	8		33				No	26.87646377	-0.4094686	66
-	SSA_2021	hypothetical protein	COG3804S							No	32.47645349	-0.08459302	151
-	SSA_2022	MerR family transcriptional regulator	COG0789K							No	59.24715827	-0.54748201	505
-	SSA_2023	fructan beta-fructosidase	COG1621G							No	33.14743772	-0.49302491	241
-	SSA_2024	hypothetical protein	-							No	23.33636364	-0.48909091	38
-	SSA_2025	GTPase protein	COG3597S	1.055045872	1.055045872	1.064220183				No	35.42195767	0.012433862	171
cadD1	SSA_2026	cadmium resistance transporter	COG4300P							No	33.13605769	0.994711538	186
copA	SSA_2027	P-type ATPase-metal/cation transport	COG2217P	1.280788177	1.374384236	1.359605911				No	39.79724891	-0.04512373	144
-	SSA_2028	hypothetical protein	-	1.407002188	1.592997812	1.509846827				No	21.1423913	-0.29891304	69
-	SSA_2029	transposase	COG3328L							No	34.42526718	-0.54122137	553
-	SSA_2030	hypothetical protein	-							No	36.57435897	-0.3974359	2
-	SSA_2031	hypothetical protein	-	1.430646333	1.806826434	1.936819172	0.461277	0.460977	0.040907	No	23.11782178	-0.11039604	67
-	SSA_2032	phage integrase family integrase/recombinase	COG4974L					2.140308		No	58.01780822	-0.62465753	74
rpsI	SSA_2033	30S ribosomal protein S9	COG0103J	0.78198567		0.357215967	0.124154	0.254107	0.330962	No	35.70846154	-0.48461539	2682
rplM	SSA_2034	50S ribosomal protein L13	COG0102J	0.693295292		0.335235378	0.258342	0.818713	0.332795	Yes	22.59256757	-0.27432432	2644
-	SSA_2035	hypothetical protein	COG1307S	1.1875	1.0625	0.854166667		0.988142		No	26.9989547	0.016376307	538
-	SSA_2036	hypothetical protein	COG3688R							No	47.51791908	-0.50462428	535
trmH	SSA_2037	tRNA (guanosine-2'-O-)-methyltransferase	COG0566J			0.6				No	17.63595041	-0.07066116	2462
-	SSA_2038	hypothetical protein	COG4129S							No	47.45378125	0.0803125	362
-	SSA_2039	aminoacid specific permease	COG0531E							No	32.95757732	0.580584192	593
-	SSA_2040	sugar ABC transporter ATP-binding protein	COG3839G				0.190232	0.376055		No	28.04893617	-0.24148936	424
lrp	SSA_2041	hypothetical protein	COG2508TQ	1.184713376	1.414012739	1.522292994				No	45.8814433	-0.25704467	586
-	SSA_2042	hypothetical protein	COG1939S	1.106508876	1.366863905	1.420118343				No	35.9858209	-0.42014925	657
cysS	SSA_2044	cysteinyl-tRNA synthetase	COG0215J	1.15	1.378571429	1.492857143		2.672498		Yes	32.42796421	-0.3689038	2722
-	SSA_2045	reductase	COG0262H	1.191666667	1.35	1.491666667				No	44.47132948	-0.20520231	581
-	SSA_2046	uridine phosphorylase	COG2820F	1.056962025	1.202531646	1.253164557				No	41.84850394	-0.23897638	46
-	SSA_2047	hypothetical protein	COG4430S	1.01734104	1.167630058	1.387283237				No	40.78472222	-0.84305556	23
cysE	SSA_2048	serine acetyltransferase	COG1045E	0.916666667	0.944444444	0.923611111				No	26.46682927	-0.17268293	2006
pnpA	SSA_2049	polynucleotide phosphorylase	COG1185J	0.848920863	0.712230216					No	31.15770805	-0.27530696	2556
-	SSA_2050	arabinose efflux permease	COG2814G							No	28.76865342	0.828697572	1944
-	SSA_2051	oligoendopeptidase	COG1164E	1.03125	0.9375	0.84375	0.283765	0.174596		No	35.50856187	-0.44264214	277
-	SSA_2052	thioredoxin	COG3118O	2.4	2.4	2.333333333				No	19.90192308	-0.01153846	808
-	SSA_2053	hypothetical protein	-	2.470588235	2.470588235	2.588235294				No	65.00958904	-0.48493151	64
-	SSA_2054	hypothetical protein	-							No	59.09457143	0.074285714	1
-	SSA_2055	hypothetical protein	-							No	79.16351351	-1.75	99
-	SSA_2056	cinnamoyl ester hydrolase	COG2267I	1.285714286	1.228571429	1.342857143				No	38.48022727	-0.24058442	1023
rpsO	SSA_2058	30S ribosomal protein S15	COG0184J	1.084525358	1.078023407	0.88556567				No	45.42040816	-0.98469388	2559
-	SSA_2059	16S rRNA uridine-516 pseudouridylate synthase	COG1187J				0.351782	0.90208	0.036658	No	30.58719008	-0.23595041	1163
-	SSA_2060	arabinose efflux permease	COG2814G	0.84375						No	29.73064267	0.830077121	359
def	SSA_2061	peptide deformylase	COG0242J	0.74015748		0.433070866	6.327501	0.055231	0.028605	Yes	38.53861905	-0.26904762	2202
-	SSA_2062	hypothetical protein	COG2261S	1.232	1.632	1.824				No	18.6	1.086842105	137
pepS	SSA_2063	aminopeptidase	COG2309E	1.102362205	1.31496063	1.37007874	0.246791	0.173012	0.211278	No	25.53486683	-0.25641647	718
-	SSA_2064	hypothetical protein	-							No	16.102	0.066	39
-	SSA_2065	hypothetical protein	COG2226H	1.197530864	1.382716049	1.469135802				No	28.7326087	-0.29	1347
polC	SSA_2066	DNA polymerase III PolC	COG2176L	0.958333333	0.979166667	1.020833333				Yes	35.88502736	-0.30902873	1307
-	SSA_2067	hypothetical protein	-	0.938271605	0.802469136					No	58.42061111	-0.84666667	3
-	SSA_2068	superfamily I DNA/RNA helicase	-			0.666666667				No	39.94444444	-0.10476191	367
proS	SSA_2069	prolyl-tRNA synthetase	COG0442J	1.165803109	1.21761658	1.134715026	0.284362	0.275085	0.529658	Yes	33.59237013	-0.26314935	2405
eep	SSA_2070	Zinc metalloprotease	COG0750M	1.139784946	0.967741935	0.849462366				No	20.51437799	0.263875598	2402
-	SSA_2071	hypothetical protein	-	1.013245033		0.721854305	0.23948			No	38.14821429	-0.38392857	1
cdsA	SSA_2072	phosphatidate cytidylyltransferase	COG0575I	1.126865672	1.059701493	1				Yes	35.12588015	0.946816479	2541
uppS	SSA_2073	undecaprenyl pyrophosphate synthase	COG0020I	0.827956989		0.440860215				Yes	41.86188755	-0.31726908	2637
yajC	SSA_2074	preprotein translocase subunit YajC	COG1862U	0.411764706	0.228373702	0.093425606				No	29.01171171	0.134234234	1215
tkt	SSA_2075	transketolase	COG0021G	1.005181347	1.046632124	1.077720207	0.280622	0.246067	0.097158	No	35.01550152	-0.15243161	2526
-	SSA_2076	L-ascorbate 6-phosphate lactonase	COG2220R	10.14285714	39	69.85714286				No	35.00192837	-0.34435262	636
-	SSA_2077	hypothetical protein	COG1266R	1.783783784	2.027027027	2				No	32.74882114	0.467073171	235
-	SSA_2078	hypothetical protein	COG3711K	1.558139535	1.627906977	1.488372093				No	44.11211573	-0.18028933	550
-	SSA_2079	acetyltransferase	COG0110R							No	30.24401099	-0.18846154	998
-	SSA_2080	hypothetical protein	COG1082G							No	34.7717004	-0.17206478	193
-	SSA_2081	hypothetical protein	-							No	58.72727273	-0.38484849	1
-	SSA_2082	carbohydrate kinase	COG1070G							No	46.65384615	-0.17475345	889
-	SSA_2083	hypothetical protein	COG2159R							No	26.13244838	-0.31681416	113
-	SSA_2084	PTS system galactitol-specific transporter subunit IIC	COG3775G							No	37.70331263	0.819254658	440
-	SSA_2085	glyoxylate reductase, NADH-dependent	COG1052CHR							No	42.82801887	-0.16603774	2257
-	SSA_2086	hypothetical protein	-							No	32.78457726	-0.24344023	26
-	SSA_2087	hypothetical protein	COG1609K	0.59602649	0.357615894	0.251655629				No	36.96513761	-0.28715596	405
araD	SSA_2088	L-ribulose-5-phosphate 4-epimerase	COG0235G	6	25	94				No	39.78949153	-0.41355932	1565
sga	SSA_2089	L-xylulose 5-phosphate 3-epimerase	COG3623G	8	38	125				No	42.67491289	-0.31289199	611
ulaD	SSA_2090	3-keto-L-gulonate-6-phosphate decarboxylase	COG0269G	7.5	35.5	106				No	27.71900452	-0.02171946	661
ptxA	SSA_2091	phosphotransferase system sugar-specific EII component	COG1762GT	9.5	41	105.5				No	56.65361446	-0.10421687	737
ptxB	SSA_2092	phosphotransferase system sugar-specific EII component	COG3414G	9.571428571	39.28571429	92.14285714				No	18.31304348	0.089130435	543
sgaT	SSA_2093	PTS system ascorbate-specific transporter subunit IIC	COG3037S	17	60.4	106.2				No	28.50187629	0.768247423	707
-	SSA_2094	hypothetical protein	-	0.622641509	0.339622642	0.18081761	0.488887			No	25.66941176	-0.24	8
clpL	SSA_2096	ATP-dependent protease, ATP-binding subunit	COG0542O							No	31.8741573	-0.45182584	501
-	SSA_2097	amino acid ABC transporter ATP-binding protein	COG1126E							No	49.09291339	-0.13228347	741
-	SSA_2098	arginine/histidine ABC transporter permease	COG0765E							No	29.27723684	0.91754386	97
-	SSA_2099	arginine/histidine ABC transporter permease	COG0765E							No	29.90178261	0.855217391	59
-	SSA_2101	amino acid ABC transporter substrate-binding protein	COG0834ET							No	18.64931973	-0.48911565	65
-	SSA_2102	hypothetical protein	COG0407H							No	28.18648649	0.048948949	57
-	SSA_2103	hypothetical protein	-							No	40.8030303	6.73E-17	2
-	SSA_2105	hypothetical protein	COG0407H							No	50.72876437	-0.25143678	19
-	SSA_2106	flavin monoxygenase	COG2141C							No	30.2469914	-0.27020057	1045
glmS	SSA_2107	glucosamine--fructose-6-phosphate aminotransferase	COG0449M				0.31974	0.818653	1.003859	Yes	25.80200663	-0.06036484	2525
gapA	SSA_2108	glyceraldehyde 3-phosphate dehydrogenase	COG0057G				0.641549	0.835227	0.467241	Yes	18.75283582	-0.08477612	2559
fusA	SSA_2109	elongation factor G	COG0480J	0.91588785			0.181217	0.499029	0.2625	Yes	38.98816739	-0.3010101	2725

rpsG	SSA_2110	30S ribosomal protein S7	COG0049J	0.860346585			0.395259	0.765546	0.019217	Yes	41.99679487	-0.53589744	2745
rpsL	SSA_2111	30S ribosomal protein S12	COG0048J	0.80375			0.684307	0.747678	0.86828	Yes	33.61167883	-0.6919708	2733
-	SSA_2112	hypothetical protein	-	0.707692308						No	5.28	-0.14666667	4
-	SSA_2113	hypothetical protein	-	0.609375	0.34375	0.21875				No	55.9038961	-0.14220779	12
purR	SSA_2114	pur operon repressor	COG0503F	0.528301887	0.283018868	0.150943396				No	45.51637011	0.093594306	678
cbf	SSA_2116	CMP-binding-factor 1	COG3481R	1.147239264	1.263803681	1.208588957				No	35.8383121	-0.3611465	668
rmuC	SSA_2117	DNA recombination protein RmuC	COG1322S	1.15503876	1.263565891	1.294573643				No	46.34043062	-0.53205742	1055
-	SSA_2118	thiamine pyrophosphokinase	COG1564H	1.095588235	1.183823529	1.132352941				No	34.78684211	-0.04122807	792
rpe	SSA_2119	pentose-5-phosphate-3-epimerase	COG0036G	1	0.961165049	0.980582524				No	27.95022831	0.076255708	2514
-	SSA_2120	GTPase	COG1162R	0.951219512	0.829268293	0.792682927				No	46.48455399	-0.33896714	1935
-	SSA_2121	cell wall surface anchor family protein	COG4932M	0.811634349						No	24.06957079	-0.62934017	130
-	SSA_2122	hypothetical protein	-							No	42.22518519	1.001481481	7
ksgA	SSA_2123	dimethyladenosine transferase	COG0030J	1.085714286	1.2	1.2				No	37.18448276	0.031724138	2733
-	SSA_2124	dehydrogenase-like protein	COG1028IQR	1.14893617	1.106382979	1.085106383				No	37.74049296	-0.35950704	644
-	SSA_2125	small primase-like protein	COG1658L	1.016129032	0.967741935					No	36.62681818	-0.53232323	567
tatD	SSA_2126	hypothetical protein	COG0084L	0.891304348		0.586956522				No	47.32539063	-0.303125	2692
-	SSA_2127	hypothetical protein	-							No	40.9375	-0.103125	2
labT	SSA_2128	hypothetical protein	-							No	41.99544794	0.889104116	77
-	SSA_2129	arsenical resistance operon repressor ArsR	COG1497K		0.433333333	0.366666667				No	37.86648199	-0.29168975	66
-	SSA_2130	hypothetical protein	-							No	33.43544304	0.889873418	11
-	SSA_2131	DNA-binding protein	COG3655K							No	32.18472222	-0.17916667	113
-	SSA_2132	Ure cluster protein	-							No	42.67887473	0.741401274	17
-	SSA_2133	3-methyladenine DNA glycosylase	COG2818L							No	39.81075269	-0.50806452	852
-	SSA_2134	hypothetical protein	COG0259H							No	33.76051948	-0.12727273	29
-	SSA_2135	DeoR family transcriptional regulator	COG2378K							No	51.24297561	-0.3097561	152
rpmH	SSA_2136	50S ribosomal protein L34	-	0.699714013		0.431839847				Yes	80.81136364	-1.40454546	2138
-	SSA_2137	hypothetical protein	-							No	46.93147619	-0.40809524	79
jag	SSA_2138	RNA-binding protein	COG1847R	0.864		0.536				No	31.52085044	-0.46979472	906
-	SSA_2139	membrane protein (preprotein translocase) oxaA 1	COG0706U	0.573770492	0.31147541	0.245901639				No	36.61697417	0.146125461	2447
rnpA	SSA_2140	ribonuclease P protein component	COG0594J	0.268292683	0.097560976	0.073170732				Yes	19.36818182	-0.41590909	1568
argH	SSA_2141	argininosuccinate lyase	COG0165E							No	39.98347826	-0.21804348	2225
argG	SSA_2142	argininosuccinate synthase	COG0137E							No	32.94588972	-0.14185464	2163
-	SSA_2143	hypothetical protein	-	0.808510638		0.468085106				No	27.67635071	-0.65971564	47
gltX	SSA_2144	glutamyl-tRNA synthetase	COG0008J	1.016064257	0.955823293	0.923694779	0.442076	0.875678	0.191992	Yes	42.74350515	-0.50680412	2756
estA	SSA_2145	tributyrin esterase	COG0627R	0.960629921	0.874015748	0.826771654				No	36.15289575	-0.51428571	1192
-	SSA_2146	metallo-beta-lactamase	COG0595R	0.901098901	0.71978022					No	31.91693694	-0.08054054	390
-	SSA_2147	hypothetical protein	-	2.253968254	2.825396825	2.936507937	0.100323	0.571358	1.04896	No	27.49850746	-0.93880597	187
-	SSA_2148	alkaline shock stress response protein	COG1302S	2.196078431	2.470588235	2.215686275	0.831314	0.406173	1.195352	No	19.46989796	-0.32142857	342
-	SSA_2149	hypothetical protein	-	1.959183673	2.183673469	1.857142857				No	46.44772021	0.360621762	111
-	SSA_2150	hypothetical protein	COG2261S	1.976190476	2.119047619	1.547619048				No	16.47088608	1.407594937	310
mga	SSA_2151	M protein trans-acting positive transcriptional regulator	COG3711K							No	36.53470588	-0.15638945	139
-	SSA_2152	ABC transporter ATPase	COG1101R	0.933333333		0.6				No	27.53611111	-0.20119048	624
-	SSA_2153	ABC transporter permease	COG4120R	0.810810811	0.621621622	0.459459459				No	15.91527778	1.002777778	731
-	SSA_2154	hypothetical protein	COG0288P			0.593220339				No	45.26606061	-0.09939394	786
-	SSA_2155	hypothetical protein	COG2013S	0.75	0.806306306					No	29.21601732	-0.09134199	446
-	SSA_2156	hypothetical protein	COG2013S	0.673913043		0.391304348				No	37.82487395	-0.08277311	173
radA	SSA_2157	DNA repair protein RadA	COG1066O	1.054545455	0.927272727	0.727272727				No	33.70455919	-0.03123426	2463
-	SSA_2158	methyltransferase	COG2226H							No	48.27666667	-0.01	70
-	SSA_2159	hypothetical protein	COG0406G	0.833333333		0.547619048				No	30.73976608	-0.17894737	91
dut	SSA_2160	deoxyuridine 5'-triphosphate nucleotidohydrolase	COG0756F		0.512820513	0.333333333				No	32.62380952	-0.18435374	2038
-	SSA_2161	NTP pyrophosphohydrolases including oxidative damage repair	COG1051F		0.419354839	0.161290323				No	29.49671141	-0.42147651	369
-	SSA_2162	hypothetical protein	-							No	56.83404255	-0.85744681	2
-	SSA_2164	glutamine amidotransferase	COG2071R	0.277486911	0.188481675	0.185863874				No	42.84502183	-0.25851528	674
oppA	SSA_2165	peptide ABC transporter periplasmic protein	COG4166E	0.851851852	0.833333333	0.925925926				No	35.25403349	-0.56940639	93
-	SSA_2166	multidrug ABC transporter ATPase/permease	COG1132V	1	1	0.951807229				No	33.50495756	0.199830221	267
-	SSA_2167	multidrug ABC transporter ATPase/permease	COG1132V	1	0.960784314	0.843137255				No	35.204	0.214782609	387
gpsA	SSA_2168	NAD(P)H-dependent glycerol-3-phosphate dehydrogenase	COG0240C	1.240384615	1.259615385	0.980769231				No	24.27617647	-0.14205882	2461
galU	SSA_2169	glucose-1-phosphate uridylyltransferase	COG1210M	1.451807229	1.692771084	1.710843373	0.751751	0.002083	0.590011	Yes	31.65924765	-0.34733542	2052
-	SSA_2170	hypothetical protein	COG0705R	1.23853211	1.47706422	1.587155963				No	42.59339207	0.992070485	1270
-	SSA_2171	hypothetical protein	COG0212H	1.124087591	1.277372263	1.394160584				No	35.76910112	-0.31460674	1327
hipO	SSA_2173	aminoacylase/N-acyl-L-amino acid amidohydrolase/hippurate h	COG1473R	1.016304348	1.086956522	1.10326087				No	33.31143236	-0.11140584	533
dapD	SSA_2174	2,3,4,5-tetrahydropyridine-2,6-carboxylate N-succinyltransferase	COG2171E	0.816455696	0.82278481	0.746835443				No	35.0012931	0.231465517	1875
-	SSA_2175	hypothetical protein	-							No	44.65134228	-0.48825503	3
-	SSA_2176	hypothetical protein	-							No	44.97067568	-0.77398649	1
-	SSA_2177	hypothetical protein	-		0.423076923	0.461538462				No	50.29513761	-0.83027523	1
-	SSA_2178	hypothetical protein	-							No	47.22689655	-0.70804598	2
-	SSA_2179	hypothetical protein	-	0.880952381	0.785714286	0.746031746				No	58.75875371	-0.59940653	5
-	SSA_2181	hypothetical protein	-	0.736842105	0.473684211	0.355263158				No	45.29135048	-0.85530547	7
-	SSA_2182	hypothetical protein	-	0.563636364	0.272727273	0.181818182				No	29.66877193	-0.7045614	9
pgi	SSA_2183	glucose-6-phosphate isomerase	COG0166G	1.075			0.62	0.34058	0.037155	Yes	34.25934783	-0.28934783	2310
-	SSA_2184	hypothetical protein	COG0590FJ							No	44.95128205	-0.21602564	2401
purA	SSA_2185	adenylosuccinate synthetase	COG0104F	0.809248555						No	32.48953488	-0.30930233	2470
-	SSA_2186	bifunctional glutamate--cysteine ligase/glutathione synthetase	COG2918H	1.13559322	1.06779661	1.033898305				No	42.88271638	-0.28548602	1029
-	SSA_2187	membrane associated protein	COG4795U		0.447368421	0.315789474				No	56.74950178	-0.67580071	77
-	SSA_2188	hypothetical protein	COG3853P	0.779069767		0.5	0.051505	0.470378	0.228087	No	44.63423445	-0.39641148	232
-	SSA_2189	hypothetical protein	COG1397O							No	28.04338235	0.048529412	682
hslO	SSA_2190	Hsp33-like chaperonin	COG1281O	0.68		0.42				No	29.95348276	-0.26448276	1551
nifR	SSA_2191	hypothetical protein	COG0042J	1	0.735849057	0.735849057	0.40945	0.712647	0.163809	No	29.95615142	-0.17223975	2444
-	SSA_2192	hypothetical protein	COG3846U	6.551020408	17.40816327	21.34693878				No	46.937751	-0.67269076	10
-	SSA_2193	ADP-ribose pyrophosphatase	COG1051F							No	35.91038462	-0.36230769	774
-	SSA_2194	nicotinamide mononucleotide transporter	COG3201H							No	26.75317343	0.121402214	492
nadR	SSA_2195	ATPase/kinase	COG3172H							No	36.74090909	-0.49147727	439
-	SSA_2196	hypothetical protein	-	1.368983957	1.663101604	1.663101604				No	22.3662406	-0.41804511	136
-	SSA_2197	hypothetical protein	COG1480R	1.350993377	1.688741722	1.953642384				No	36.95583333	1.106410256	140
-	SSA_2198	hypothetical protein	-	1.160377358	1.29245283	1.311320755				No	51.76801619	-0.33441296	48
clpC	SSA_2199	ATP-dependent Clp protease, ATP-binding subunit	COG0542O	0.925531915	0.914893617	0.85106383				No	37.87033375	-0.29085291	1778
ctsR	SSA_2200	CtsR family transcriptional regulator	COG4463K		0.428571429	0.228571429				No	43.44155844	-0.14935065	525
-	SSA_2201	hypothetical protein	-	1.428571429	1.987012987	2.701298701				No	29.55603448	0.765517241	10
tsf	SSA_2202	elongation factor Ts	COG0264J	0.791469194			0.278203	0.371743	0.349309	Yes	24.4945245	-0.17175793	2570
rpsB	SSA_2203	30S ribosomal protein S2	COG0052J	0.438880707	0.272459499	0.216494845	0.319588	0.803571	0.189453	Yes	46.64884615	-0.34384615	2753
-	SSA_2204	hypothetical protein	-		0.46875	0.5				No	40.72051181	-0.6519685	1

nusG	SSA_2205	transcription antitermination protein NusG	COG0250K	0.758241758			0.547109	0.435521	0.26654	No	34.75955056	-0.3241573	2563
-	SSA_2206	hypothetical protein	-		2.454545455					No	57.26779661	-0.99915254	1
-	SSA_2207	hypothetical protein	-							No	38.50769231	0.476923077	1
secE	SSA_2208	preprotein translocase subunit SecE	COG0690U	1.047619048	0.904761905	0.717948718				Yes	11.13220339	0.462711864	152
pbp2a	SSA_2209	penicillin-binding protein 2A	COG0744M	0.879310345	0.715517241					No	34.15636119	-0.37183288	1695
-	SSA_2210	ribosomal large subunit pseudouridine synthase D	COG0564J							No	27.27424749	-0.3645485	388
-	SSA_2211	transmembrane protein	COG3274S							No	35.78469136	0.677469136	12
-	SSA_2212	polysaccharide transport protein	COG0534V	1.119047619	1.238095238	1.142857143				No	27.22036952	0.796997691	33
-	SSA_2213	nucleotide sugar dehydratase	COG0451MG	0.991304348	1.026086957	1.095652174				No	36.9449435	-0.17683616	630
-	SSA_2214	2-C-methyl-D-erythritol 4-phosphate cytidylyltransferase	COG1211I	1.097345133	1.194690265	1.256637168				No	31.89504167	-0.01958333	2090
-	SSA_2215	oligosaccharide repeat-containing polymerase	-	1.071428571	1.224489796	1.234693878				No	26.78741497	0.700226757	23
licD1	SSA_2216	LPS biosynthesis protein	COG3475M	1.008849558	1.123893805	1.168141593				No	50.27397394	-0.6009772	230
-	SSA_2217	Cps9H	COG0463M	0.939130435	1.069565217	1.069565217				No	26.9984326	-0.28871473	146
-	SSA_2218	glycosyltransferase (cell wall biogenesis) Cps9G	COG1215M	0.906976744	0.96124031	1.046511628				No	48.66102941	-0.36102941	440
-	SSA_2219	UDP-glucose 4-epimerase	COG0451MG	0.870229008	0.900763359	0.854961832				No	46.10986014	-0.52552448	539
-	SSA_2220	galactosyltransferase	COG2148M	0.785714286	0.75					No	32.04084577	-0.14726368	1812
capD	SSA_2221	Cps9E	COG1086MG	0.782945736						No	24.12185	0.068333333	1323
-	SSA_2222	tyrosine-protein kinase Wze	COG0489D	0.714285714		0.396825397	0.029712	0.090853		No	36.8125	-0.07543103	2275
-	SSA_2223	capsular polysaccharide biosynthesis protein Wzd (chain length	COG3944M	0.672566372	0.415929204	0.353982301				No	56.13419913	0.177056277	445
-	SSA_2224	phosphotyrosine-protein phosphatase	COG4464GM	0.692307692	0.403846154	0.326923077				No	38.50617284	-0.28765432	521
cpsA	SSA_2225	LytR family transcriptional regulator	COG1316K	0.643835616	0.397260274	0.301369863				No	29.25458167	-0.17649402	203
nrdG	SSA_2226	organic radical activating chaperone	COG0602O	1.242574257	1.089108911	0.693069307				No	30.93830846	-0.40945274	969
-	SSA_2227	acetyl transferase	COG3981R	1.231578947	1.168421053	0.815789474		0.043474	0.542204	No	58.54475138	-0.58066298	178
-	SSA_2228	acetyltransferase	COG3981R	1.166666667	1.038461538	0.769230769				No	44.84363636	-0.27030303	219
-	SSA_2229	hypothetical protein	-	1.287334594	1.183364839	0.988657845				No	32.71929825	-1.41578947	151
nrdD	SSA_2230	anaerobic ribonucleoside triphosphate reductase	COG1328F	1.346666667	1.133333333			0.760061	0.146077	No	31.45049248	-0.52407661	1238
-	SSA_2231	Heme/copper-type cytochrome/quinol oxidases subunit 1	COG1807M	0.839416058						No	43.54009709	0.317281553	133
-	SSA_2233	hypothetical protein	-							No	44.06382979	-1.1212766	1
cls	SSA_2234	phosphatidylserine/phosphatidylglycerophosphate/cardiolipin sy	COG1502I	1.096774194	1.161290323	1.032258065				No	29.80277887	0.015851272	1682
-	SSA_2235	hypothetical protein	-	1.25	1.33974359	1.198717949				No	55.11006289	-0.4591195	48
-	SSA_2237	dihydrofolate:folylpolyglutamate synthetase	COG0285H	1.089552239	1.134328358	1.119402985				No	37.10414634	-0.13463415	892
-	SSA_2239	hypothetical protein	COG3906S	1.266149871	1.444444444	1.42377261				No	49.60679612	-0.87864078	441
-	SSA_2240	Holliday junction resolvase-like protein	COG0816L	1.178832117	1.222627737	1.01459854				Yes	21.28201439	-0.23884892	2302
-	SSA_2241	hypothetical protein	COG4472S	1.099585062	1.087136929	0.883817427				No	16.46704545	-0.80909091	571
-	SSA_2242	hypothetical protein	-	0.417910448	0.23880597	0.179104478				No	48.57222222	-0.34555556	9
-	SSA_2243	hypothetical protein	-	0.73553719		0.537190083				No	39.69736842	-0.59736842	41
spxA	SSA_2244	Spx family transcriptional regulator	COG1393P	1.111814346	1.023206751	0.852320675	0.889447	2.097925		No	41.53863636	-0.28560606	267
recA	SSA_2245	recombinase A	COG0468L	1.200873362	1.187772926	0.965065502	0.470607	0.976636	0.421783	No	30.43667539	-0.3052356	2661
cinA	SSA_2246	competence damage-inducible protein A	COG1058R	1.102040816	1.040816327	0.918367347				No	33.81902715	-0.02556561	2253
-	SSA_2247	hypothetical protein	COG1813K							No	44.71772059	0.008088235	152
-	SSA_2248	hypothetical protein	-							No	27.76386503	-0.34355828	15
-	SSA_2249	peptide ABC transporter ATPase	COG1136V							No	23.91835749	-0.15362319	220
-	SSA_2250	peptide ABC transporter permease	COG4652S							No	29.31139394	0.277727273	35
-	SSA_2251	hypothetical protein	COG3620K							No	44.06528369	-0.46489362	132
-	SSA_2252	hypothetical protein	-	1.064516129						No	41.59931034	0.980689655	99
tag	SSA_2253	3-methyladenine DNA glycosylase	COG2818L							No	32.05548913	-0.48967391	707
ruvA	SSA_2254	Holliday junction DNA helicase RuvA	COG0632L	0.75		0.475				No	27.26173469	0.00255102	2511
-	SSA_2255	XRE family transcriptional regulator	COG1476K							No	31.66309524	-0.23690476	533
-	SSA_2256	hypothetical protein	-							No	31.92245902	0.319672131	19
mutL	SSA_2257	DNA mismatch repair protein	COG0323L	1	1	0.975903614				No	42.57851623	-0.38361669	2091
-	SSA_2258	hypothetical protein	COG4260S	1.194444444	1.444444444	1.694444444				No	38.07545872	-0.20481651	205
-	SSA_2259	hypothetical protein	COG4307S							No	23.05064935	-0.15974026	25
mutS	SSA_2260	DNA mismatch repair protein MutS	COG0249L	0.97260274	0.931506849	0.863013699				No	42.42628975	-0.20435807	2135
-	SSA_2261	transcriptional repressor (arginine synthesis)	COG1438K	0.516666667	0.266666667	0.166666667				No	34.91931034	-0.23103448	546
argS	SSA_2262	arginyl-tRNA synthetase	COG0018J	0.803738318			0.350381	0.445693	0.334065	Yes	36.83010676	-0.34234875	2728
nrdI	SSA_2263	flavoprotein NrdI	COG1780F	0.651376147		0.321100917				Yes	37.85555556	-0.31234568	700
-	SSA_2264	hypothetical protein	-	0.697478992		0.31092437				No	24.31110465	0.648837209	58
malP	SSA_2265	maltodextrin phosphorylase	COG0058G		1.421052632	1.473684211				No	34.25857902	-0.19654715	451
malQ	SSA_2266	4-alpha-glucanotransferase	COG1640G							No	37.32445328	-0.42007952	1390
malR	SSA_2267	lactose operon transcriptional repressor, LacI family	COG1609K	2.461538462	3.538461538	3.846153846				No	37.46211009	-0.26850153	737
pulA	SSA_2268	Type II secretory pathway, pullulanase PulA glycosidase	COG1523G		2.833333333	3.5				No	38.07557803	-0.37153179	639
-	SSA_2269	hypothetical protein	COG1284S	1.156521739	1.095652174	1.052173913				No	29.27466238	0.362379421	678
aspS	SSA_2270	aspartyl-tRNA synthetase	COG0173J	0.896825397						No	40.49881849	-0.33270548	2551
-	SSA_2271	hypothetical protein	-	1.03030303		0.575757576				No	21.17285714	0.942857143	43
-	SSA_2272	hypothetical protein	COG4495S	1.189189189	1.135135135	1.108108108				No	43.71939394	-0.4030303	119
-	SSA_2273	hypothetical protein	-							No	37.69331839	0.267264574	5
-	SSA_2274	hypothetical protein	-	0.923076923	1.038461538	1.076923077				No	21.65503597	-0.37248201	23
-	SSA_2275	hypothetical protein	-		1.272727273				0.981173	No	62.5338843	-0.9231405	4
-	SSA_2276	hypothetical protein	-							No	40.62421875	-0.1109375	14
-	SSA_2277	DNA segregation ATPase FtsK/SpoIIIE family protein	COG1674D	1.024390244	1.207317073	1.219512195				No	36.94350746	-0.30176391	481
ukp	SSA_2278	Ukp protein	COG4499S	1.081632653	1.204081633	1.040816327		0.8	3.561399	No	36.69689737	-0.37398568	178
-	SSA_2279	hypothetical protein	COG5417S	1.227272727	1.068181818	0.863636364				No	81.51012658	-0.3835443	30
-	SSA_2281	hypothetical protein	-	0.872340426		0.5				No	33.89605263	-0.20855263	16
-	SSA_2282	Phage infection protein	COG1511S	0.833333333						No	26.14127789	-0.1637931	162
-	SSA_2283	hypothetical protein	COG4842S	1.027317429	1.031506101		0.1768	0.576642	0.534392	No	27.64947368	-0.33894737	213
hisS	SSA_2284	histidyl-tRNA synthetase	COG0124J	1.020833333	1.111111111	1.131944444	0.17083	0.72332	0.109667	Yes	38.58147887	-0.35586855	2735
-	SSA_2285	hypothetical protein	COG2151R	0.363636364	0.181818182	0.136363636				No	36.53628319	-0.10088496	252
ilvD	SSA_2286	dihydroxy-acid dehydratase	COG0129EG				0.164506	0.598394	0.015842	No	32.69143357	-0.05786713	2152
rpmF	SSA_2287	50S ribosomal protein L32	COG0333J	0.691699605		0.399209486				No	61.285	-1.03666667	1625
cadD2	SSA_2288	cadmium resistance transporter	COG4300P							No	33.95931373	1.010294118	189
cadX	SSA_2289	cadmium efflux system transcriptional regulator (ArsR-like)	COG0640K							No	44.145625	-0.17857143	1003
-	SSA_2290	hypothetical protein	-							No	48.80083682	-0.31736402	35
-	SSA_2291	hypothetical protein	-							No	69	-1.07852349	19
-	SSA_2292	DNA segregation ATPase FtsK/SpoIIIE-like protein	COG1674D							No	35.29562842	-0.06557377	134
-	SSA_2293	hypothetical protein	-							No	32.59104478	-0.03233831	57
-	SSA_2294	hypothetical protein	-							No	60.38810945	-0.62835821	60
-	SSA_2295	phage integrase family integrase/recombinase	COG4974L	1.054054054	1.027027027	1.135135135				No	21.11567696	-0.46532067	835
-	SSA_2296	XRE family transcriptional regulator	COG1476K	1.181818182	1.484848485	1.848484848				No	32.83513514	-0.57162162	444
-	SSA_2297	hypothetical protein	-	1.208333333	1.375	1.75				No	22.45616162	0.893434343	12
-	SSA_2298	serine protease	COG3480T	1.194444444	1.222222222	1.083333333				No	26.5755814	-0.18313954	113
-	SSA_2299	hypothetical protein	-	1.282352941	1.447058824	1.482352941				No	29.55996622	0.934459459	1

-	SSA_2300	hypothetical protein	COG0474P	1.171875	1.21875	1.09375				No	33.53855263	0.857894737	1
lytB	SSA_2301	S-layer protein	COG4193G	1.154639175	1.036082474	0.958762887				No	30.38396985	-0.09095477	164
-	SSA_2302	Type IV fimbrial biogenesis protein, prepilin cysteine protease (	COG1989NOU	1.026315789	0.957894737	0.810526316				No	29.12520325	0.934552846	1172
-	SSA_2303	hypothetical protein	COG2720V	0.966244726	0.839662447	0.801687764				No	40.43169492	-0.3019774	3
-	SSA_2304	hypothetical protein	COG3881S	0.895104895	0.772727273					No	50.23130194	-0.07368421	20
-	SSA_2305	hypothetical protein	-	0.794594595	0.708108108					No	49.38960139	-0.32305026	1
-	SSA_2307	hypothetical protein	COG5309G	0.95	1.05	1.15				No	31.88467803	-0.53882576	1
-	SSA_2308	hypothetical protein	-							No	47.51968912	-0.42797928	13
-	SSA_2309	fimbrial assembly protein	COG4972NU	0.961538462						No	41.62682979	-0.36744681	90
-	SSA_2310	hypothetical protein	COG2165NU							No	23.62121212	-0.36707071	10
norD	SSA_2311	fused nitric oxide reductase NorD/von Willebrand factor type A	COG4795U							No	22.15968468	-0.34504505	13
-	SSA_2312	hypothetical protein	-							No	29.6191358	-0.32839506	2
-	SSA_2313	hypothetical protein	COG2165NU							No	19.22380952	-0.20612245	1
-	SSA_2314	hypothetical protein	COG2165NU	0.895348837	0.872093023	0.965116279				No	27.23892617	-0.34161074	5
-	SSA_2315	hypothetical protein	COG2165NU	0.994845361	1.154639175	1.417525773				No	26.99868421	-0.27960526	10
-	SSA_2316	general secretory pathway protein F	COG1459NU	0.886363636	0.840909091	0.863636364				No	26.61105392	0.225735294	1332
-	SSA_2317	Tfp pilus assembly protein, pilus retraction ATPase PilT	COG2805NU	0.863636364		0.818181818	0.292588			No	32.37259887	-0.14491525	1350
-	SSA_2318	PilB-like pili biogenesis ATPase	COG2804NU	0.642857143						No	37.64107143	-0.16553571	1779
-	SSA_2320	hypothetical protein	COG5422	1.003616637	1.209764919	1.428571429				No	34.16495704	-0.32963918	4
czcD	SSA_2321	cation (Co/Zn/Cd) efflux protein	COG1230P							No	32.27525773	0.423367698	1539
-	SSA_2322	TetR/AcrR family transcriptional regulator	COG1309K							No	45.60410405	-0.34450867	453
-	SSA_2323	transporter	COG2807P							No	36.15649485	0.931443299	885
-	SSA_2324	hypothetical protein	COG0793M							No	36.06333333	-0.54619048	44
-	SSA_2325	3-methyladenine DNA glycosylase	COG2094L							No	48.29509901	-0.41881188	937
-	SSA_2327	hypothetical protein	-	0.760869565		0.565217391				No	41.5221374	-0.30152672	2
fcsR	SSA_2328	sugar metabolism transcriptional repressor	COG1349KG	2.75	8.083333333	15.08333333				No	33.16822581	-0.32096774	317
-	SSA_2329	hypothetical protein	COG0561R	1.739130435	5.173913043	11.7826087				No	35.12367273	-0.06472727	380
-	SSA_2330	acetyltransferase	-	1.032258065	1.14516129					No	37.24782609	-0.35403727	434
dltD	SSA_2331	D-alanine transfer protein	COG3966M	1.137931034	1.245689655	1.267241379				No	45.85075829	-0.52322275	399
dltC	SSA_2332	D-alanine--poly(phosphoribitol) ligase subunit 2	COG0236IQ	1.117948718	1.153846154	1.153846154				No	48.42405063	-0.06835443	455
dltB	SSA_2333	hypothetical protein	COG1696M	1.074418605	1.023255814	1.004651163				No	32.99157005	0.468115942	940
dltA	SSA_2334	D-alanine--poly(phosphoribitol) ligase subunit 1	COG1020Q	0.879227053	0.801932367	0.719806763				No	36.75757752	-0.14844961	2416
dltX	SSA_2335	D-Ala-teichoic acid biosynthesis protein	-		0.444444444	0.259259259				No	12.6372093	0.472093023	115
-	SSA_2336	PadR family transcriptional regulator	COG1695K	1.625	2.025	2.775				No	47.38125	-0.42321429	840
-	SSA_2337	hypothetical protein	COG4709S	1.723404255	2.361702128	3.042553191				No	25.27291457	0.557286432	204
-	SSA_2338	hypothetical protein	-	1.490566038	1.924528302	2.433962264				No	30.31791908	-0.19537572	107
-	SSA_2339	microcin C7 resistance protein	COG1619V							No	39.29842271	-0.16971609	1060
-	SSA_2340	hypothetical protein	-							No	20.93041475	-0.17511521	124
-	SSA_2342	SPX domain-containing protein	COG5036P							No	40.70759494	-0.52067511	91
-	SSA_2343	hypothetical protein	COG1511S	0.895582329						No	19.61747212	0.043122677	742
-	SSA_2344	hypothetical protein	-							No	14.48979592	-0.25306122	11
-	SSA_2345	hypothetical protein	COG1309K	0.602941176	0.338235294	0.176470588				No	59.3791018	-0.42874252	258
-	SSA_2346	3-oxoacyl-ACP synthase	COG0332I	1.336734694	1.489795918	1.418367347				No	27.00063694	0.019745223	119
-	SSA_2347	coenzyme F390 synthetase	COG1541H	1.228571429	1.304761905	1.361904762				No	40.98816279	-0.33139535	547
-	SSA_2348	metal dependent hydrolase	COG0491R	1.058823529	1.014705882	0.941176471				No	46.69323843	-0.11850534	550
galE1	SSA_2349	dTDP-4-dehydrorhamnose 3,5-epimerase	COG0451MG	0.810344828		0.431034483				No	36.79746356	-0.12419825	801
rpsD	SSA_2350	30S ribosomal protein S4	COG0522J	0.370808679	0.163708087	0.116370809	0.379228	0.856338	0.305862	Yes	43.44493878	-0.28204082	2761
-	SSA_2351	nitrate/sulfonate/bicarbonate ABC transporter ATPase	COG1116P							No	46.20583333	-0.045	666
-	SSA_2352	nitrate/sulfonate/bicarbonate ABC transporter periplasmic prote	COG0715P					0.880468		No	32.24	-0.52072072	760
-	SSA_2353	nitrate/sulfonate/bicarbonate ABC transporter permease	COG0600P							No	39.04724	0.96	1529
-	SSA_2354	hypothetical protein	COG1011R							No	48.53580952	-0.05428571	737
-	SSA_2355	hypothetical protein	COG4466S	1.00862069	1.163793103	1.00862069				No	38.39230769	-0.73956044	498
dnaC	SSA_2356	replicative DNA helicase	COG0305L	1.020100503	1.145728643	1.145728643				Yes	47.98713969	-0.27272727	2591
rplI	SSA_2357	50S ribosomal protein L9	COG0359J	1.050359712	1.172661871	1.194244604				No	34.53333333	-0.36333333	2420
-	SSA_2358	hypothetical protein	COG3887T	1.035714286	0.982142857					No	39.18796215	-0.01455604	682
gidA	SSA_2359	tRNA uridine 5-carboxymethylaminomethyl modification protein	COG0445D	1.07	0.86	0.77				No	34.24740157	-0.39212598	2256
mnmA	SSA_2360	tRNA-specific 2-thiouridylase MnmA	COG0482J	0.857142857	0.626373626	0.527472527				Yes	34.23115282	-0.42091153	2529
sdaB	SSA_2361	L-serine dehydratase beta subunit	COG1760E							No	35.79506726	-0.0587444	555
sdaA	SSA_2362	L-serine dehydratase alpha subunit	COG1760E							No	38.23382759	0.238965517	1705
-	SSA_2363	phosphoglycolate phosphatase	COG0546R				1.299441			No	42.44748837	-0.21069767	2024
-	SSA_2364	hypothetical protein	COG0739M		0.353221957	0.346062053				No	18.75408163	-0.08418367	696
-	SSA_2365	cobalt transport protein cbiQ	COG0619P	1.263157895	1.434210526	1.434210526				No	21.01098485	0.734469697	1108
cbiO	SSA_2366	cobalt transporter ATP-binding subunit	COG1122P	1.194444444	1.25	1.166666667				No	28.96523297	-0.00358423	869
cbiO	SSA_2367	cobalt transporter ATP-binding subunit	COG1122P	1.090909091	1.051948052	0.987012987				No	34.26847458	-0.32033898	832
pgsA	SSA_2368	CDP-diacylglycerol--glycerol-3-phosphate 3-phosphatidyltransf	COG0558I	0.944444444		0.652777778				Yes	25.23240223	0.936312849	2411
-	SSA_2369	hypothetical protein	COG1426S	0.810810811		0.459459459				No	39.33035211	-0.22746479	1042
-	SSA_2370	zinc-dependent peptidase	COG0612R	0.733333333	0.533333333	0.4				No	26.35454756	-0.23990719	2087
-	SSA_2371	Zn-dependent peptidase	COG0612R		0.357142857	0.214285714		5.858995	0.167325	No	41.1251074	-0.24558473	738
-	SSA_2372	hypothetical protein	COG2501S	1.019230769	0.932692308	0.913461538				No	58.99545455	-0.82424242	730
recF	SSA_2373	recombination protein F	COG1195L	1.130952381	1.035714286	1.30952381				No	36.30604396	-0.33489011	2141
guaB	SSA_2374	inosine 5'-monophosphate dehydrogenase	COG0516F	0.526315789	0.289473684	0.184210526	0.501315	0.362018	0.140886	No	28.44077079	-0.03630832	2485
trpS	SSA_2375	tryptophanyl-tRNA synthetase II	COG0180J	0.548076923	0.326923077	0.240384615	0.008841	0.192132		Yes	41.3914956	-0.34897361	2738
-	SSA_2376	ABC transporter ATPase	COG0488R	0.647619048	0.4	0.276190476				No	33.10881057	-0.35881057	1420
-	SSA_2377	copper ABC transporter permease	COG4485S							No	26.1252381	0.197444832	331
comE	SSA_2378	two-component system LytR/AlgR family transcriptional regulat	COG3279KT							No	27.02527559	-0.49488189	385
comD	SSA_2379	signal transduction protein	COG2972T							No	39.54054054	0.254054054	383
-	SSA_2380	rRNA large subunit methyltransferase	COG1576S							No	35.02075472	-0.18867925	1854
htrA	SSA_2381	DegP protein	COG0265O	0.727272727	0.439393939	0.242424242				No	28.18871795	-0.15358974	2543
-	SSA_2382	chromosome partitioning protein ParB	COG1475K	0.925	0.7125	0.4375				No	51.39540541	-0.4972973	2186
doc	SSA_2383	prophage maintenance system killer protein (DOC: death-on-cu	COG3654R							No	30.60742647	0.013235294	236
-	SSA_2384	acetyltransferase	COG0456R							No	24.7971831	-0.39084507	204
-	SSA_2385	hypothetical protein	-							No	22.66854839	1.143548387	24
-	SSA_2386	hypothetical protein	COG5547S	2.147058824	2.529411765	1.941176471				No	22.09285714	0.9	121
-	SSA_2387	arsenical resistance operon transcription repressor	COG0640K							No	38.87877049	-0.09508197	278
-	SSA_2388	hypothetical protein	-							No	60.6254902	-0.71764706	6
-	SSA_2389	arsenical resistance operon transcription repressor	COG0640K				0.617731	0.5957	0.504866	No	43.56169643	-0.14107143	357
-	SSA_2390	hypothetical protein	COG4481S	1.104651163		0.918604651				No	42.0234375	-0.5671875	575
rpsN	SSA_2391	30S ribosomal protein S14	COG0199J	1.152671756	1.419847328	1.658669575				Yes	16.80983607	-0.66557377	2543
rpmJ	SSA_2392	50S ribosomal protein L36	COG0257J	0.976721629	0.824442289	0.58971872	0.401487	0.761062	0.793071	No	53.92894737	-0.64210526	1759
-	SSA_2393	XRE family transcriptional regulator	COG1476K							No	51.40746269	-0.5761194	476
comC	SSA_2394	competence stimulating peptide	-	1.161290323	1.290322581	1.451612903				No	18.31142857	-5.08E-17	1

∞ Instability Index was calculated using the method of Guruprasad et al. [113] Any value > 40 indicates protein instability and short half-life *in vitro*.

§ The hydropathy values range from -2 (least hydrophobic) to +2 (most hydrophobic) for most proteins.

Ω Protein conservation was measured across 2774 proteomes of bacteria with fully sequenced genomes.

ϕ Empty spaces in mRNA and protein fold changes with respect to T0 indicate either a statistically non-significant value or an undetected measure.

Supplementary Table 2. Amino acids located at the N-terminal position in *S. sanguinis* proteins.

Non-essential protein	N-terminal amino acid	Essential protein	N-terminal amino acid
SSA_0005	A	SSA_0106	A
SSA_0051	A	SSA_0108	A
SSA_0052	A	SSA_0112	A
SSA_0053	A	SSA_0119	A
SSA_0068	A	SSA_0124	A
SSA_0080	A	SSA_0129	A
SSA_0083	A	SSA_0131	A
SSA_0085	A	SSA_0133	A
SSA_0102	A	SSA_0226	A
SSA_0125	A	SSA_0302	A
SSA_0130	A	SSA_0312	A
SSA_0145	A	SSA_0437	A
SSA_0158	A	SSA_0440	A
SSA_0160	A	SSA_0543	A
SSA_0168	A	SSA_0683	A
SSA_0172	A	SSA_0787	A
SSA_0187	A	SSA_0825	A
SSA_0188	A	SSA_1105	A
SSA_0190	A	SSA_1167	A
SSA_0205	A	SSA_1189	A
SSA_0213	A	SSA_1232	A
SSA_0222	A	SSA_1310	A
SSA_0231	A	SSA_1498	A
SSA_0235	A	SSA_1520	A
SSA_0238	A	SSA_1606	A
SSA_0271	A	SSA_1619	A
SSA_0299	A	SSA_1622	A
SSA_0300	A	SSA_1623	A
SSA_0311	A	SSA_1803	A
SSA_0321	A	SSA_1931	A
SSA_0324	A	SSA_1939	A
SSA_0349	A	SSA_1992	A
SSA_0353	A	SSA_2109	A
SSA_0364	A	SSA_2202	A
SSA_0374	A	SSA_2203	A
SSA_0379	A	SSA_2356	A
SSA_0390	A	SSA_2391	A
SSA_0401	A	SSA_2107	C
SSA_0434	A	SSA_0199	D
SSA_0451	A	SSA_0200	D
SSA_0455	A	SSA_0334	D
SSA_0476	A	SSA_0785	D
SSA_0489	A	SSA_1784	D
SSA_0521	A	SSA_1903	D
SSA_0522	A	SSA_2262	D
SSA_0524	A	SSA_0100	E
SSA_0525	A	SSA_0116	E
SSA_0531	A	SSA_0176	E
SSA_0539	A	SSA_0289	E
SSA_0546	A	SSA_0783	E
SSA_0566	A	SSA_0846	E
SSA_0567	A	SSA_1094	E

SSA_0572	A	SSA_1805	E
SSA_0574	A	SSA_1936	E
SSA_0578	A	SSA_2263	E
SSA_0582	A	SSA_2350	E
SSA_0593	A	SSA_0127	F
SSA_0596	A	SSA_0713	F
SSA_0609	A	SSA_1932	F
SSA_0637	A	SSA_2073	F
SSA_0655	A	SSA_0110	G
SSA_0671	A	SSA_0111	G
SSA_0677	A	SSA_0113	G
SSA_0702	A	SSA_0770	G
SSA_0704	A	SSA_0804	G
SSA_0706	A	SSA_0862	G
SSA_0721	A	SSA_1557	G
SSA_0739	A	SSA_0943	H
SSA_0751	A	SSA_0002	I
SSA_0772	A	SSA_0013	I
SSA_0806	A	SSA_0117	I
SSA_0809	A	SSA_0123	I
SSA_0839	A	SSA_0132	I
SSA_0901	A	SSA_0438	I
SSA_0962	A	SSA_0547	I
SSA_0980	A	SSA_0691	I
SSA_0987	A	SSA_0824	I
SSA_0988	A	SSA_0871	I
SSA_1000	A	SSA_1491	I
SSA_1002	A	SSA_1571	I
SSA_1006	A	SSA_1739	I
SSA_1028	A	SSA_1933	I
SSA_1032	A	SSA_2044	I
SSA_1037	A	SSA_2183	I
SSA_1053	A	SSA_0198	K
SSA_1115	A	SSA_0335	K
SSA_1128	A	SSA_0337	K
SSA_1139	A	SSA_0569	K
SSA_1140	A	SSA_0652	K
SSA_1151	A	SSA_0653	K
SSA_1174	A	SSA_0661	K
SSA_1175	A	SSA_0692	K
SSA_1178	A	SSA_0756	K
SSA_1188	A	SSA_0801	K
SSA_1193	A	SSA_0847	K
SSA_1214	A	SSA_0999	K
SSA_1244	A	SSA_1220	K
SSA_1248	A	SSA_1302	K
SSA_1255	A	SSA_1452	K
SSA_1296	A	SSA_1484	K
SSA_1306	A	SSA_1500	K
SSA_1333	A	SSA_1522	K
SSA_1360	A	SSA_1565	K
SSA_1434	A	SSA_1721	K
SSA_1438	A	SSA_1722	K
SSA_1456	A	SSA_1806	K
SSA_1463	A	SSA_1871	K
SSA_1527	A	SSA_1935	K

SSA_1537	A	SSA_2069	K
SSA_1552	A	SSA_2136	K
SSA_1558	A	SSA_2208	K
SSA_1566	A	SSA_2284	K
SSA_1572	A	SSA_2368	K
SSA_1589	A	SSA_0114	L
SSA_1601	A	SSA_0225	L
SSA_1626	A	SSA_0720	L
SSA_1706	A	SSA_0914	L
SSA_1724	A	SSA_1062	L
SSA_1726	A	SSA_1870	L
SSA_1756	A	SSA_1925	L
SSA_1768	A	SSA_2140	L
SSA_1782	A	SSA_0174	M
SSA_1796	A	SSA_0336	M
SSA_1802	A	SSA_0548	M
SSA_1808	A	SSA_0789	M
SSA_1810	A	SSA_0936	M
SSA_1823	A	SSA_1213	M
SSA_1833	A	SSA_1233	M
SSA_1857	A	SSA_1848	M
SSA_1883	A	SSA_1851	M
SSA_1895	A	SSA_0109	N
SSA_1899	A	SSA_0128	N
SSA_1906	A	SSA_0571	N
SSA_1921	A	SSA_0782	N
SSA_1946	A	SSA_0784	N
SSA_1953	A	SSA_0848	N
SSA_1964	A	SSA_0859	N
SSA_1968	A	SSA_0941	N
SSA_1978	A	SSA_1152	N
SSA_1980	A	SSA_1223	N
SSA_1989	A	SSA_1265	N
SSA_2008	A	SSA_1819	N
SSA_2015	A	SSA_1934	N
SSA_2024	A	SSA_1940	N
SSA_2045	A	SSA_2005	N
SSA_2089	A	SSA_2034	N
SSA_2127	A	SSA_2169	N
SSA_2145	A	SSA_0010	P
SSA_2176	A	SSA_0575	P
SSA_2197	A	SSA_2111	P
SSA_2210	A	SSA_0768	Q
SSA_2222	A	SSA_1047	Q
SSA_2229	A	SSA_1430	Q
SSA_2235	A	SSA_1938	Q
SSA_2245	A	SSA_1086	R
SSA_2260	A	SSA_2240	R
SSA_2268	A	SSA_0020	S
SSA_2283	A	SSA_0063	S
SSA_2287	A	SSA_0122	S
SSA_2294	A	SSA_0625	S
SSA_2296	A	SSA_0786	S
SSA_2300	A	SSA_0788	S
SSA_2309	A	SSA_0807	S
SSA_2318	A	SSA_0870	S

SSA_2338	A
SSA_2340	A
SSA_2342	A
SSA_1010	C
SSA_2164	C
SSA_0008	D
SSA_0016	D
SSA_0023	D
SSA_0030	D
SSA_0050	D
SSA_0066	D
SSA_0089	D
SSA_0143	D
SSA_0154	D
SSA_0329	D
SSA_0380	D
SSA_0385	D
SSA_0425	D
SSA_0428	D
SSA_0430	D
SSA_0460	D
SSA_0468	D
SSA_0498	D
SSA_0520	D
SSA_0553	D
SSA_0562	D
SSA_0604	D
SSA_0617	D
SSA_0699	D
SSA_0775	D
SSA_0940	D
SSA_0958	D
SSA_1045	D
SSA_1126	D
SSA_1134	D
SSA_1154	D
SSA_1156	D
SSA_1191	D
SSA_1256	D
SSA_1280	D
SSA_1294	D
SSA_1309	D
SSA_1324	D
SSA_1336	D
SSA_1413	D
SSA_1458	D
SSA_2384	D
SSA_1479	D
SSA_1508	D
SSA_1579	D
SSA_1608	D
SSA_1612	D
SSA_1643	D
SSA_1645	D
SSA_1684	D
SSA_1700	D
SSA_0886	S
SSA_1104	S
SSA_1184	S
SSA_1226	S
SSA_1495	S
SSA_1555	S
SSA_1738	S
SSA_1748	S
SSA_1863	S
SSA_1872	S
SSA_1880	S
SSA_2007	S
SSA_2061	S
SSA_2066	S
SSA_2072	S
SSA_2110	S
SSA_2360	S
SSA_0001	T
SSA_0107	T
SSA_0333	T
SSA_0338	T
SSA_0570	T
SSA_0656	T
SSA_0869	T
SSA_0878	T
SSA_0912	T
SSA_0944	T
SSA_1091	T
SSA_1092	T
SSA_1209	T
SSA_1419	T
SSA_1529	T
SSA_1642	T
SSA_1703	T
SSA_1800	T
SSA_1811	T
SSA_1930	T
SSA_1937	T
SSA_2144	T
SSA_2375	T
SSA_0006	V
SSA_0120	V
SSA_0177	V
SSA_0197	V
SSA_0688	V
SSA_0771	V
SSA_0800	V
SSA_1620	V
SSA_1879	V
SSA_2108	V
SSA_0997	Y
SSA_1604	Y
SSA_1864	Y
SSA_1865	Y

SSA_1707	D
SSA_1720	D
SSA_1779	D
SSA_1824	D
SSA_1841	D
SSA_1852	D
SSA_1856	D
SSA_1878	D
SSA_1913	D
SSA_1999	D
SSA_2018	D
SSA_2098	D
SSA_2168	D
SSA_2190	D
SSA_2205	D
SSA_2322	D
SSA_2332	D
SSA_2365	D
SSA_0011	E
SSA_0061	E
SSA_0086	E
SSA_0139	E
SSA_0170	E
SSA_0134	E
SSA_0201	E
SSA_0204	E
SSA_2393	E
SSA_0207	E
SSA_0253	E
SSA_0255	E
SSA_0261	E
SSA_0268	E
SSA_0269	E
SSA_0278	E
SSA_0282	E
SSA_0286	E
SSA_0298	E
SSA_0308	E
SSA_0330	E
SSA_0350	E
SSA_0360	E
SSA_0412	E
SSA_0418	E
SSA_0443	E
SSA_0458	E
SSA_0462	E
SSA_0487	E
SSA_0503	E
SSA_0528	E
SSA_0532	E
SSA_0538	E
SSA_0540	E
SSA_0552	E
SSA_0576	E
SSA_0615	E
SSA_0636	E

SSA_0670	E
SSA_0694	E
SSA_0696	E
SSA_0709	E
SSA_0779	E
SSA_0791	E
SSA_0797	E
SSA_0888	E
SSA_0895	E
SSA_0899	E
SSA_0909	E
SSA_0915	E
SSA_0954	E
SSA_0956	E
SSA_0957	E
SSA_0966	E
SSA_0981	E
SSA_1018	E
SSA_1052	E
SSA_1056	E
SSA_1060	E
SSA_1063	E
SSA_1070	E
SSA_1118	E
SSA_1121	E
SSA_1146	E
SSA_1148	E
SSA_1169	E
SSA_1205	E
SSA_1261	E
SSA_1279	E
SSA_1287	E
SSA_1331	E
SSA_1347	E
SSA_1450	E
SSA_1453	E
SSA_1466	E
SSA_1502	E
SSA_1506	E
SSA_1538	E
SSA_1551	E
SSA_1561	E
SSA_1569	E
SSA_1614	E
SSA_1616	E
SSA_1627	E
SSA_1635	E
SSA_1641	E
SSA_1656	E
SSA_1672	E
SSA_1761	E
SSA_1763	E
SSA_1766	E
SSA_1799	E
SSA_1817	E
SSA_1821	E

SSA_1827	E
SSA_1837	E
SSA_1846	E
SSA_1881	E
SSA_1958	E
SSA_1970	E
SSA_1971	E
SSA_1975	E
SSA_1991	E
SSA_2012	E
SSA_2031	E
SSA_2037	E
SSA_2051	E
SSA_2117	E
SSA_2132	E
SSA_2135	E
SSA_2386	E
SSA_2178	E
SSA_2181	E
SSA_2223	E
SSA_2227	E
SSA_2228	E
SSA_2279	E
SSA_2295	E
SSA_2325	E
SSA_2372	E
SSA_0034	F
SSA_0099	F
SSA_0118	F
SSA_0161	F
SSA_0179	F
SSA_0274	F
SSA_0397	F
SSA_0406	F
SSA_0461	F
SSA_0479	F
SSA_0523	F
SSA_0529	F
SSA_0561	F
SSA_0607	F
SSA_0622	F
SSA_0623	F
SSA_0629	F
SSA_0666	F
SSA_0687	F
SSA_0701	F
SSA_0718	F
SSA_0749	F
SSA_0829	F
SSA_0844	F
SSA_0874	F
SSA_0893	F
SSA_0931	F
SSA_1015	F
SSA_1157	F
SSA_1166	F

SSA_1239	F
SSA_1357	F
SSA_1376	F
SSA_1475	F
SSA_1560	F
SSA_1649	F
SSA_1659	F
SSA_1680	F
SSA_1723	F
SSA_1737	F
SSA_1762	F
SSA_2385	F
SSA_1793	F
SSA_1909	F
SSA_1911	F
SSA_1959	F
SSA_1993	F
SSA_2065	F
SSA_2071	F
SSA_2252	F
SSA_2301	F
SSA_2362	F
SSA_0014	G
SSA_0149	G
SSA_0159	G
SSA_0182	G
SSA_0248	G
SSA_0249	G
SSA_0376	G
SSA_0383	G
SSA_0420	G
SSA_0586	G
SSA_0695	G
SSA_0794	G
SSA_0808	G
SSA_0904	G
SSA_0905	G
SSA_0906	G
SSA_0995	G
SSA_1076	G
SSA_1096	G
SSA_1103	G
SSA_1113	G
SSA_1131	G
SSA_1133	G
SSA_1194	G
SSA_1266	G
SSA_1323	G
SSA_1380	G
SSA_1381	G
SSA_1392	G
SSA_1503	G
SSA_1544	G
SSA_1640	G
SSA_1701	G
SSA_1732	G

SSA_1736	G
SSA_1759	G
SSA_2387	G
SSA_2048	G
SSA_2054	G
SSA_2068	G
SSA_2175	G
SSA_2200	G
SSA_2212	G
SSA_2213	G
SSA_2241	G
SSA_2258	G
SSA_2259	G
SSA_2305	G
SSA_2344	G
SSA_2366	G
SSA_2376	G
SSA_0166	H
SSA_0368	H
SSA_0381	H
SSA_0413	H
SSA_0484	H
SSA_0852	H
SSA_0883	H
SSA_0896	H
SSA_1011	H
SSA_1135	H
SSA_1180	H
SSA_1281	H
SSA_1543	H
SSA_1685	H
SSA_1861	H
SSA_1941	H
SSA_2231	H
SSA_0041	I
SSA_0043	I
SSA_0046	I
SSA_0055	I
SSA_0069	I
SSA_0073	I
SSA_0098	I
SSA_0181	I
SSA_0203	I
SSA_0211	I
SSA_0212	I
SSA_0217	I
SSA_0228	I
SSA_0240	I
SSA_0247	I
SSA_0277	I
SSA_0285	I
SSA_0309	I
SSA_0313	I
SSA_0317	I
SSA_0320	I
SSA_0382	I

SSA_0423	I
SSA_0450	I
SSA_0490	I
SSA_0497	I
SSA_0515	I
SSA_0519	I
SSA_0601	I
SSA_0633	I
SSA_0642	I
SSA_0643	I
SSA_0705	I
SSA_0733	I
SSA_0736	I
SSA_0796	I
SSA_0836	I
SSA_0838	I
SSA_0858	I
SSA_0864	I
SSA_0903	I
SSA_0916	I
SSA_0926	I
SSA_0937	I
SSA_0952	I
SSA_0959	I
SSA_0986	I
SSA_0993	I
SSA_1026	I
SSA_1057	I
SSA_1081	I
SSA_1111	I
SSA_1155	I
SSA_1262	I
SSA_1275	I
SSA_1293	I
SSA_1319	I
SSA_1356	I
SSA_1365	I
SSA_1370	I
SSA_1378	I
SSA_1379	I
SSA_1390	I
SSA_1393	I
SSA_1397	I
SSA_1423	I
SSA_1432	I
SSA_1504	I
SSA_1512	I
SSA_1516	I
SSA_1545	I
SSA_1546	I
SSA_1549	I
SSA_1554	I
SSA_1564	I
SSA_1630	I
SSA_1651	I
SSA_1655	I

SSA_1691	I
SSA_1697	I
SSA_1730	I
SSA_1731	I
SSA_1733	I
SSA_1746	I
SSA_1781	I
SSA_1798	I
SSA_1812	I
SSA_1813	I
SSA_1816	I
SSA_1818	I
SSA_1820	I
SSA_1845	I
SSA_1867	I
SSA_1914	I
SSA_1922	I
SSA_1986	I
SSA_1996	I
SSA_2388	I
SSA_2053	I
SSA_2062	I
SSA_2078	I
SSA_2125	I
SSA_2128	I
SSA_2131	I
SSA_2134	I
SSA_2153	I
SSA_2191	I
SSA_2192	I
SSA_2217	I
SSA_2224	I
SSA_2230	I
SSA_2233	I
SSA_2244	I
SSA_2288	I
SSA_2290	I
SSA_2302	I
SSA_2347	I
SSA_0004	K
SSA_0015	K
SSA_0019	K
SSA_0021	K
SSA_0026	K
SSA_0033	K
SSA_0035	K
SSA_0036	K
SSA_0037	K
SSA_0039	K
SSA_0042	K
SSA_0047	K
SSA_0048	K
SSA_0049	K
SSA_0062	K
SSA_0065	K
SSA_0070	K

SSA_0074	K
SSA_0076	K
SSA_0079	K
SSA_0091	K
SSA_0094	K
SSA_0115	K
SSA_0121	K
SSA_0126	K
SSA_2392	K
SSA_0138	K
SSA_0141	K
SSA_0142	K
SSA_0146	K
SSA_0150	K
SSA_0167	K
SSA_0169	K
SSA_0173	K
SSA_0178	K
SSA_0186	K
SSA_0193	K
SSA_0202	K
SSA_0206	K
SSA_0208	K
SSA_0210	K
SSA_0218	K
SSA_0219	K
SSA_0227	K
SSA_0233	K
SSA_0234	K
SSA_0245	K
SSA_0251	K
SSA_0257	K
SSA_0260	K
SSA_0265	K
SSA_0266	K
SSA_0272	K
SSA_0279	K
SSA_0281	K
SSA_0296	K
SSA_0301	K
SSA_0303	K
SSA_0304	K
SSA_0306	K
SSA_0316	K
SSA_0318	K
SSA_0323	K
SSA_0325	K
SSA_0348	K
SSA_0351	K
SSA_0356	K
SSA_0367	K
SSA_0378	K
SSA_0394	K
SSA_0398	K
SSA_0400	K
SSA_0414	K

SSA_0415	K
SSA_0422	K
SSA_0424	K
SSA_0426	K
SSA_0433	K
SSA_0445	K
SSA_0447	K
SSA_0453	K
SSA_0459	K
SSA_0463	K
SSA_0477	K
SSA_0478	K
SSA_0485	K
SSA_0488	K
SSA_0491	K
SSA_0493	K
SSA_0500	K
SSA_0505	K
SSA_0507	K
SSA_0509	K
SSA_0510	K
SSA_0512	K
SSA_0516	K
SSA_0537	K
SSA_0564	K
SSA_0565	K
SSA_0568	K
SSA_0573	K
SSA_0580	K
SSA_0581	K
SSA_0584	K
SSA_0587	K
SSA_0592	K
SSA_0595	K
SSA_0599	K
SSA_0602	K
SSA_0608	K
SSA_0614	K
SSA_0626	K
SSA_0628	K
SSA_0634	K
SSA_0640	K
SSA_0641	K
SSA_0646	K
SSA_0664	K
SSA_0668	K
SSA_0672	K
SSA_0684	K
SSA_0685	K
SSA_0686	K
SSA_0707	K
SSA_0723	K
SSA_0725	K
SSA_0726	K
SSA_0730	K
SSA_0731	K

SSA_0732	K
SSA_0735	K
SSA_0741	K
SSA_0746	K
SSA_0747	K
SSA_0750	K
SSA_0753	K
SSA_0755	K
SSA_0758	K
SSA_0759	K
SSA_0762	K
SSA_0763	K
SSA_0766	K
SSA_0776	K
SSA_0777	K
SSA_0778	K
SSA_0780	K
SSA_0793	K
SSA_0799	K
SSA_0803	K
SSA_0805	K
SSA_0816	K
SSA_0817	K
SSA_0818	K
SSA_0830	K
SSA_0831	K
SSA_0832	K
SSA_0835	K
SSA_0842	K
SSA_0857	K
SSA_0865	K
SSA_0872	K
SSA_0876	K
SSA_0877	K
SSA_0880	K
SSA_0882	K
SSA_0897	K
SSA_0908	K
SSA_0917	K
SSA_0921	K
SSA_0922	K
SSA_0927	K
SSA_0928	K
SSA_0942	K
SSA_0945	K
SSA_0947	K
SSA_0948	K
SSA_0949	K
SSA_0963	K
SSA_0967	K
SSA_0969	K
SSA_0978	K
SSA_0984	K
SSA_0991	K
SSA_0994	K
SSA_1003	K

SSA_1004	K
SSA_1005	K
SSA_1012	K
SSA_1013	K
SSA_1016	K
SSA_1019	K
SSA_1022	K
SSA_1023	K
SSA_1024	K
SSA_1031	K
SSA_1036	K
SSA_1044	K
SSA_1048	K
SSA_1049	K
SSA_1050	K
SSA_1054	K
SSA_1066	K
SSA_1067	K
SSA_1069	K
SSA_1072	K
SSA_1082	K
SSA_1084	K
SSA_1088	K
SSA_1089	K
SSA_1095	K
SSA_1101	K
SSA_1106	K
SSA_1109	K
SSA_1112	K
SSA_1114	K
SSA_1116	K
SSA_1117	K
SSA_1120	K
SSA_1122	K
SSA_1124	K
SSA_1129	K
SSA_1144	K
SSA_1145	K
SSA_1147	K
SSA_1161	K
SSA_1173	K
SSA_1196	K
SSA_1200	K
SSA_1203	K
SSA_1207	K
SSA_1211	K
SSA_1216	K
SSA_1230	K
SSA_1234	K
SSA_1238	K
SSA_1246	K
SSA_1247	K
SSA_1250	K
SSA_1251	K
SSA_1252	K
SSA_1253	K

SSA_1267	K
SSA_1269	K
SSA_1276	K
SSA_1277	K
SSA_1285	K
SSA_1289	K
SSA_1298	K
SSA_1300	K
SSA_1307	K
SSA_1308	K
SSA_1326	K
SSA_1328	K
SSA_1329	K
SSA_1339	K
SSA_1340	K
SSA_1342	K
SSA_1345	K
SSA_1348	K
SSA_1352	K
SSA_1355	K
SSA_1358	K
SSA_1359	K
SSA_1361	K
SSA_1362	K
SSA_1363	K
SSA_1368	K
SSA_1369	K
SSA_1371	K
SSA_1374	K
SSA_1375	K
SSA_1382	K
SSA_1383	K
SSA_1384	K
SSA_1385	K
SSA_1388	K
SSA_1394	K
SSA_1395	K
SSA_1396	K
SSA_1399	K
SSA_1400	K
SSA_1402	K
SSA_1408	K
SSA_1411	K
SSA_1415	K
SSA_1433	K
SSA_1436	K
SSA_1451	K
SSA_1454	K
SSA_1457	K
SSA_1460	K
SSA_1462	K
SSA_1464	K
SSA_1468	K
SSA_1470	K
SSA_1474	K
SSA_1476	K

SSA_1477	K
SSA_1481	K
SSA_1483	K
SSA_1485	K
SSA_1486	K
SSA_1487	K
SSA_1489	K
SSA_1490	K
SSA_1494	K
SSA_1501	K
SSA_1509	K
SSA_1510	K
SSA_1511	K
SSA_1513	K
SSA_1514	K
SSA_1518	K
SSA_1519	K
SSA_1526	K
SSA_1528	K
SSA_1531	K
SSA_1532	K
SSA_1533	K
SSA_1542	K
SSA_1567	K
SSA_1570	K
SSA_1573	K
SSA_1581	K
SSA_1585	K
SSA_1588	K
SSA_1591	K
SSA_1593	K
SSA_1596	K
SSA_1599	K
SSA_1600	K
SSA_1602	K
SSA_1621	K
SSA_1625	K
SSA_1629	K
SSA_1631	K
SSA_1632	K
SSA_1633	K
SSA_1634	K
SSA_1639	K
SSA_1647	K
SSA_1648	K
SSA_1652	K
SSA_1653	K
SSA_1657	K
SSA_1661	K
SSA_1668	K
SSA_1675	K
SSA_1682	K
SSA_1687	K
SSA_1689	K
SSA_1690	K
SSA_1698	K

SSA_1705	K
SSA_1716	K
SSA_1727	K
SSA_1729	K
SSA_1734	K
SSA_1735	K
SSA_1742	K
SSA_1744	K
SSA_1747	K
SSA_1750	K
SSA_1764	K
SSA_1770	K
SSA_1772	K
SSA_1775	K
SSA_1780	K
SSA_1787	K
SSA_1792	K
SSA_1830	K
SSA_1831	K
SSA_1832	K
SSA_1840	K
SSA_1842	K
SSA_1843	K
SSA_1849	K
SSA_1855	K
SSA_1862	K
SSA_1869	K
SSA_1875	K
SSA_1876	K
SSA_1888	K
SSA_1890	K
SSA_1892	K
SSA_1900	K
SSA_1904	K
SSA_1907	K
SSA_1917	K
SSA_1943	K
SSA_1947	K
SSA_1948	K
SSA_1949	K
SSA_1951	K
SSA_1960	K
SSA_1961	K
SSA_1972	K
SSA_1974	K
SSA_1976	K
SSA_1981	K
SSA_1984	K
SSA_1985	K
SSA_1987	K
SSA_1990	K
SSA_1995	K
SSA_1997	K
SSA_2001	K
SSA_2004	K
SSA_2006	K

SSA_2011	K
SSA_2014	K
SSA_2019	K
SSA_2022	K
SSA_2389	K
SSA_2029	K
SSA_2032	K
SSA_2036	K
SSA_2041	K
SSA_2055	K
SSA_2056	K
SSA_2058	K
SSA_2060	K
SSA_2064	K
SSA_2067	K
SSA_2077	K
SSA_2080	K
SSA_2082	K
SSA_2091	K
SSA_2094	K
SSA_2113	K
SSA_2116	K
SSA_2121	K
SSA_2122	K
SSA_2124	K
SSA_2126	K
SSA_2129	K
SSA_2139	K
SSA_2143	K
SSA_2157	K
SSA_2158	K
SSA_2159	K
SSA_2160	K
SSA_2167	K
SSA_2171	K
SSA_2177	K
SSA_2195	K
SSA_2196	K
SSA_2198	K
SSA_2199	K
SSA_2201	K
SSA_2204	K
SSA_2209	K
SSA_2211	K
SSA_2218	K
SSA_2219	K
SSA_2226	K
SSA_2243	K
SSA_2246	K
SSA_2247	K
SSA_2248	K
SSA_2250	K
SSA_2255	K
SSA_2264	K
SSA_2266	K
SSA_2269	K

SSA_2270	K
SSA_2275	K
SSA_2281	K
SSA_2282	K
SSA_2286	K
SSA_2289	K
SSA_2297	K
SSA_2298	K
SSA_2304	K
SSA_2307	K
SSA_2310	K
SSA_2312	K
SSA_2321	K
SSA_2323	K
SSA_2324	K
SSA_2327	K
SSA_2330	K
SSA_2335	K
SSA_2339	K
SSA_2348	K
SSA_2353	K
SSA_2357	K
SSA_2369	K
SSA_2378	K
SSA_2394	K
SSA_2380	K
SSA_2381	K
SSA_0025	L
SSA_0137	L
SSA_0152	L
SSA_0156	L
SSA_0162	L
SSA_0185	L
SSA_0195	L
SSA_0327	L
SSA_0343	L
SSA_0352	L
SSA_0354	L
SSA_0363	L
SSA_0377	L
SSA_0396	L
SSA_0402	L
SSA_0408	L
SSA_0410	L
SSA_0442	L
SSA_0449	L
SSA_0472	L
SSA_0480	L
SSA_0494	L
SSA_0504	L
SSA_0506	L
SSA_0556	L
SSA_0557	L
SSA_0559	L
SSA_0590	L
SSA_0603	L

SSA_0606	L
SSA_0611	L
SSA_0612	L
SSA_0616	L
SSA_0669	L
SSA_0679	L
SSA_0690	L
SSA_0715	L
SSA_0722	L
SSA_0724	L
SSA_0734	L
SSA_0745	L
SSA_0795	L
SSA_0814	L
SSA_0819	L
SSA_0822	L
SSA_0849	L
SSA_0850	L
SSA_0894	L
SSA_0913	L
SSA_0929	L
SSA_0939	L
SSA_0946	L
SSA_0960	L
SSA_0965	L
SSA_0972	L
SSA_0973	L
SSA_0989	L
SSA_1001	L
SSA_1017	L
SSA_1020	L
SSA_1059	L
SSA_1080	L
SSA_1107	L
SSA_1125	L
SSA_1132	L
SSA_1164	L
SSA_1165	L
SSA_1187	L
SSA_1206	L
SSA_1235	L
SSA_1264	L
SSA_1284	L
SSA_1364	L
SSA_1366	L
SSA_1373	L
SSA_1391	L
SSA_1404	L
SSA_1416	L
SSA_1424	L
SSA_1440	L
SSA_1442	L
SSA_1459	L
SSA_1482	L
SSA_1578	L
SSA_1615	L

SSA_1665	L
SSA_1671	L
SSA_1673	L
SSA_1678	L
SSA_1679	L
SSA_1699	L
SSA_1708	L
SSA_1709	L
SSA_1712	L
SSA_1728	L
SSA_1752	L
SSA_1769	L
SSA_1797	L
SSA_1814	L
SSA_1834	L
SSA_1836	L
SSA_1847	L
SSA_1866	L
SSA_1950	L
SSA_1965	L
SSA_1967	L
SSA_2028	L
SSA_2039	L
SSA_2046	L
SSA_2084	L
SSA_2088	L
SSA_2097	L
SSA_2112	L
SSA_2150	L
SSA_2173	L
SSA_2207	L
SSA_2206	L
SSA_2272	L
SSA_2313	L
SSA_2314	L
SSA_2315	L
SSA_2331	L
SSA_2343	L
SSA_2345	L
SSA_2358	L
SSA_2382	L
SSA_0064	M
SSA_0241	M
SSA_0326	M
SSA_0375	M
SSA_0411	M
SSA_0421	M
SSA_0467	M
SSA_0473	M
SSA_0517	M
SSA_0613	M
SSA_0659	M
SSA_0761	M
SSA_0854	M
SSA_1065	M
SSA_1099	M

SSA_1198	M
SSA_1313	M
SSA_1321	M
SSA_1405	M
SSA_1444	M
SSA_1541	M
SSA_1753	M
SSA_1794	M
SSA_1809	M
SSA_1828	M
SSA_1850	M
SSA_2184	M
SSA_2186	M
SSA_2271	M
SSA_2311	M
SSA_2333	M
SSA_2352	M
SSA_0017	N
SSA_0044	N
SSA_0045	N
SSA_0103	N
SSA_0165	N
SSA_0175	N
SSA_0191	N
SSA_0232	N
SSA_0273	N
SSA_0288	N
SSA_0291	N
SSA_0295	N
SSA_0310	N
SSA_0345	N
SSA_0346	N
SSA_0355	N
SSA_0366	N
SSA_0370	N
SSA_0386	N
SSA_0392	N
SSA_0393	N
SSA_0403	N
SSA_0405	N
SSA_0419	N
SSA_0429	N
SSA_0456	N
SSA_0492	N
SSA_0495	N
SSA_0549	N
SSA_0560	N
SSA_0588	N
SSA_0591	N
SSA_0657	N
SSA_0678	N
SSA_0711	N
SSA_0712	N
SSA_0743	N
SSA_0744	N
SSA_0765	N

SSA_0798	N
SSA_0802	N
SSA_0845	N
SSA_0860	N
SSA_0889	N
SSA_0892	N
SSA_0932	N
SSA_0935	N
SSA_0938	N
SSA_0961	N
SSA_0996	N
SSA_0998	N
SSA_1021	N
SSA_1038	N
SSA_1041	N
SSA_1055	N
SSA_1074	N
SSA_1083	N
SSA_1100	N
SSA_1102	N
SSA_1158	N
SSA_1168	N
SSA_1179	N
SSA_1181	N
SSA_1185	N
SSA_1197	N
SSA_1201	N
SSA_1274	N
SSA_1278	N
SSA_1297	N
SSA_1303	N
SSA_1311	N
SSA_1316	N
SSA_1337	N
SSA_1346	N
SSA_1389	N
SSA_1403	N
SSA_1421	N
SSA_1448	N
SSA_1471	N
SSA_1493	N
SSA_1496	N
SSA_1536	N
SSA_1540	N
SSA_1550	N
SSA_1553	N
SSA_1576	N
SSA_1592	N
SSA_1597	N
SSA_1618	N
SSA_1658	N
SSA_1663	N
SSA_1666	N
SSA_1669	N
SSA_1670	N
SSA_1693	N

SSA_1694	N
SSA_1740	N
SSA_1765	N
SSA_1776	N
SSA_1786	N
SSA_1788	N
SSA_1790	N
SSA_1804	N
SSA_1815	N
SSA_1829	N
SSA_1860	N
SSA_1874	N
SSA_1897	N
SSA_1901	N
SSA_1927	N
SSA_1982	N
SSA_2017	N
SSA_2020	N
SSA_2023	N
SSA_2074	N
SSA_2086	N
SSA_2093	N
SSA_2096	N
SSA_2103	N
SSA_2114	N
SSA_2170	N
SSA_2189	N
SSA_2237	N
SSA_2299	N
SSA_2317	N
SSA_2364	N
SSA_2377	N
SSA_0144	P
SSA_0236	P
SSA_0250	P
SSA_0252	P
SSA_0362	P
SSA_0502	P
SSA_0589	P
SSA_0674	P
SSA_0710	P
SSA_0875	P
SSA_0898	P
SSA_1093	P
SSA_1098	P
SSA_1150	P
SSA_1260	P
SSA_1292	P
SSA_1341	P
SSA_1372	P
SSA_1406	P
SSA_1412	P
SSA_1420	P
SSA_1455	P
SSA_1473	P
SSA_1499	P

SSA_1605	P
SSA_1607	P
SSA_1789	P
SSA_1884	P
SSA_1893	P
SSA_1957	P
SSA_2050	P
SSA_2076	P
SSA_2085	P
SSA_2276	P
SSA_0022	Q
SSA_0242	Q
SSA_0270	Q
SSA_0358	Q
SSA_0407	Q
SSA_0409	Q
SSA_0448	Q
SSA_0513	Q
SSA_0554	Q
SSA_0632	Q
SSA_0648	Q
SSA_0650	Q
SSA_0727	Q
SSA_0834	Q
SSA_0841	Q
SSA_0887	Q
SSA_0933	Q
SSA_0955	Q
SSA_1058	Q
SSA_1075	Q
SSA_1172	Q
SSA_1186	Q
SSA_1190	Q
SSA_1229	Q
SSA_1237	Q
SSA_1257	Q
SSA_1312	Q
SSA_1325	Q
SSA_1417	Q
SSA_1422	Q
SSA_1530	Q
SSA_1586	Q
SSA_1676	Q
SSA_1710	Q
SSA_1719	Q
SSA_1743	Q
SSA_1791	Q
SSA_1795	Q
SSA_1853	Q
SSA_1882	Q
SSA_1926	Q
SSA_2025	Q
SSA_2030	Q
SSA_2070	Q
SSA_2370	Q
SSA_0009	R

SSA_0012	R
SSA_0018	R
SSA_0060	R
SSA_0067	R
SSA_0090	R
SSA_0136	R
SSA_0216	R
SSA_0287	R
SSA_0292	R
SSA_0319	R
SSA_0328	R
SSA_0359	R
SSA_0441	R
SSA_0452	R
SSA_0469	R
SSA_0471	R
SSA_0482	R
SSA_0496	R
SSA_0508	R
SSA_0533	R
SSA_0585	R
SSA_0624	R
SSA_0627	R
SSA_0649	R
SSA_0689	R
SSA_0748	R
SSA_0757	R
SSA_0811	R
SSA_0843	R
SSA_0866	R
SSA_0881	R
SSA_0964	R
SSA_1027	R
SSA_1035	R
SSA_1051	R
SSA_1064	R
SSA_1068	R
SSA_1078	R
SSA_1171	R
SSA_1208	R
SSA_1227	R
SSA_1241	R
SSA_1245	R
SSA_1272	R
SSA_1283	R
SSA_1286	R
SSA_1301	R
SSA_1304	R
SSA_1318	R
SSA_1320	R
SSA_1335	R
SSA_1353	R
SSA_1398	R
SSA_1429	R
SSA_1439	R
SSA_1445	R

SSA_1467	R
SSA_1469	R
SSA_1492	R
SSA_1505	R
SSA_1525	R
SSA_1562	R
SSA_1574	R
SSA_1584	R
SSA_1617	R
SSA_1628	R
SSA_1644	R
SSA_1702	R
SSA_1754	R
SSA_1777	R
SSA_1807	R
SSA_1877	R
SSA_1891	R
SSA_1902	R
SSA_1929	R
SSA_1969	R
SSA_2000	R
SSA_2021	R
SSA_2026	R
SSA_2059	R
SSA_2123	R
SSA_2151	R
SSA_2155	R
SSA_2165	R
SSA_2166	R
SSA_2216	R
SSA_2242	R
SSA_2251	R
SSA_2256	R
SSA_2261	R
SSA_2267	R
SSA_2285	R
SSA_2303	R
SSA_2308	R
SSA_2320	R
SSA_2371	R
SSA_0027	S
SSA_0028	S
SSA_0040	S
SSA_0057	S
SSA_0071	S
SSA_0078	S
SSA_0081	S
SSA_0087	S
SSA_0088	S
SSA_0092	S
SSA_0104	S
SSA_0105	S
SSA_0140	S
SSA_0155	S
SSA_0180	S
SSA_0189	S

SSA_0192	S
SSA_0229	S
SSA_0243	S
SSA_0244	S
SSA_0276	S
SSA_0284	S
SSA_0305	S
SSA_0307	S
SSA_0331	S
SSA_0365	S
SSA_0369	S
SSA_0384	S
SSA_0387	S
SSA_0388	S
SSA_0389	S
SSA_0416	S
SSA_0427	S
SSA_0435	S
SSA_0446	S
SSA_0470	S
SSA_0474	S
SSA_0475	S
SSA_0518	S
SSA_0526	S
SSA_0530	S
SSA_0536	S
SSA_0541	S
SSA_0555	S
SSA_0594	S
SSA_0597	S
SSA_0635	S
SSA_0654	S
SSA_0658	S
SSA_0673	S
SSA_0675	S
SSA_0700	S
SSA_0703	S
SSA_0714	S
SSA_0716	S
SSA_0729	S
SSA_0737	S
SSA_0740	S
SSA_0767	S
SSA_0769	S
SSA_0781	S
SSA_0792	S
SSA_0810	S
SSA_0812	S
SSA_0813	S
SSA_0815	S
SSA_0820	S
SSA_0826	S
SSA_0827	S
SSA_0863	S
SSA_0879	S
SSA_0900	S

SSA_0907	S
SSA_0911	S
SSA_0920	S
SSA_0925	S
SSA_0934	S
SSA_0971	S
SSA_0985	S
SSA_0992	S
SSA_1008	S
SSA_1009	S
SSA_1030	S
SSA_1040	S
SSA_1043	S
SSA_1061	S
SSA_1085	S
SSA_1087	S
SSA_1090	S
SSA_1110	S
SSA_1127	S
SSA_1137	S
SSA_1138	S
SSA_1143	S
SSA_1182	S
SSA_1212	S
SSA_1217	S
SSA_1224	S
SSA_1242	S
SSA_1288	S
SSA_1305	S
SSA_1330	S
SSA_1343	S
SSA_1344	S
SSA_1350	S
SSA_1377	S
SSA_1386	S
SSA_1387	S
SSA_1401	S
SSA_1431	S
SSA_1443	S
SSA_1447	S
SSA_1465	S
SSA_1488	S
SSA_1497	S
SSA_1507	S
SSA_1515	S
SSA_1517	S
SSA_1521	S
SSA_1547	S
SSA_1559	S
SSA_1568	S
SSA_1594	S
SSA_1598	S
SSA_1603	S
SSA_1636	S
SSA_1662	S
SSA_1667	S

SSA_1686	S
SSA_1718	S
SSA_1725	S
SSA_1741	S
SSA_1745	S
SSA_1757	S
SSA_1771	S
SSA_1778	S
SSA_1822	S
SSA_1826	S
SSA_1839	S
SSA_1896	S
SSA_1912	S
SSA_1918	S
SSA_1919	S
SSA_1923	S
SSA_1952	S
SSA_1954	S
SSA_1956	S
SSA_1994	S
SSA_1998	S
SSA_2010	S
SSA_2013	S
SSA_2016	S
SSA_2027	S
SSA_2033	S
SSA_2038	S
SSA_2075	S
SSA_2081	S
SSA_2090	S
SSA_2101	S
SSA_2118	S
SSA_2120	S
SSA_2137	S
SSA_2146	S
SSA_2147	S
SSA_2148	S
SSA_2149	S
SSA_2154	S
SSA_2161	S
SSA_2174	S
SSA_2179	S
SSA_2182	S
SSA_2187	S
SSA_2188	S
SSA_2257	S
SSA_2265	S
SSA_2277	S
SSA_2291	S
SSA_2334	S
SSA_2336	S
SSA_2355	S
SSA_2359	S
SSA_2363	S
SSA_2374	S
SSA_2379	S

SSA_0031	T
SSA_0032	T
SSA_0054	T
SSA_0056	T
SSA_0072	T
SSA_0093	T
SSA_0095	T
SSA_0097	T
SSA_0101	T
SSA_0135	T
SSA_0153	T
SSA_0157	T
SSA_0209	T
SSA_0220	T
SSA_0221	T
SSA_0224	T
SSA_0230	T
SSA_0239	T
SSA_0254	T
SSA_0256	T
SSA_0263	T
SSA_0267	T
SSA_0290	T
SSA_0293	T
SSA_0297	T
SSA_0314	T
SSA_0332	T
SSA_0339	T
SSA_0341	T
SSA_0371	T
SSA_0391	T
SSA_0395	T
SSA_0431	T
SSA_0436	T
SSA_0457	T
SSA_0464	T
SSA_0465	T
SSA_0466	T
SSA_0511	T
SSA_0527	T
SSA_0535	T
SSA_0544	T
SSA_0551	T
SSA_0563	T
SSA_0577	T
SSA_0579	T
SSA_0583	T
SSA_0605	T
SSA_0610	T
SSA_0618	T
SSA_0620	T
SSA_0631	T
SSA_0638	T
SSA_0644	T
SSA_0647	T
SSA_0660	T

SSA_0665	T
SSA_0667	T
SSA_0676	T
SSA_0680	T
SSA_0682	T
SSA_0697	T
SSA_0698	T
SSA_0708	T
SSA_0728	T
SSA_0738	T
SSA_0760	T
SSA_0773	T
SSA_0774	T
SSA_0837	T
SSA_0851	T
SSA_0855	T
SSA_0856	T
SSA_0861	T
SSA_0868	T
SSA_0873	T
SSA_0884	T
SSA_0885	T
SSA_0891	T
SSA_0918	T
SSA_0950	T
SSA_0968	T
SSA_0977	T
SSA_0983	T
SSA_0990	T
SSA_1029	T
SSA_1033	T
SSA_1034	T
SSA_1039	T
SSA_1042	T
SSA_1073	T
SSA_1077	T
SSA_1130	T
SSA_1136	T
SSA_1149	T
SSA_1153	T
SSA_1162	T
SSA_1163	T
SSA_1170	T
SSA_1176	T
SSA_1183	T
SSA_1192	T
SSA_1202	T
SSA_1204	T
SSA_1210	T
SSA_1221	T
SSA_1222	T
SSA_1225	T
SSA_1240	T
SSA_1249	T
SSA_1259	T
SSA_1268	T

SSA_1270	T
SSA_1271	T
SSA_1282	T
SSA_1291	T
SSA_1299	T
SSA_1314	T
SSA_1315	T
SSA_1317	T
SSA_1322	T
SSA_1327	T
SSA_1332	T
SSA_1338	T
SSA_1349	T
SSA_1409	T
SSA_1410	T
SSA_1414	T
SSA_1418	T
SSA_1428	T
SSA_1435	T
SSA_1441	T
SSA_1446	T
SSA_2383	T
SSA_1478	T
SSA_1480	T
SSA_1523	T
SSA_1535	T
SSA_1548	T
SSA_1563	T
SSA_1575	T
SSA_1577	T
SSA_1582	T
SSA_1587	T
SSA_1595	T
SSA_1611	T
SSA_1624	T
SSA_1646	T
SSA_1650	T
SSA_1660	T
SSA_1664	T
SSA_1681	T
SSA_1692	T
SSA_1704	T
SSA_1715	T
SSA_1717	T
SSA_1751	T
SSA_1758	T
SSA_1773	T
SSA_1783	T
SSA_1801	T
SSA_1835	T
SSA_1854	T
SSA_1858	T
SSA_1873	T
SSA_1889	T
SSA_1910	T
SSA_1915	T

SSA_1916	T
SSA_1920	T
SSA_1928	T
SSA_1942	T
SSA_1944	T
SSA_1945	T
SSA_1955	T
SSA_1962	T
SSA_1979	T
SSA_2002	T
SSA_2035	T
SSA_2042	T
SSA_2047	T
SSA_2049	T
SSA_2083	T
SSA_2087	T
SSA_2102	T
SSA_2119	T
SSA_2130	T
SSA_2152	T
SSA_2162	T
SSA_2185	T
SSA_2193	T
SSA_2214	T
SSA_2221	T
SSA_2234	T
SSA_2239	T
SSA_2249	T
SSA_2253	T
SSA_2278	T
SSA_2292	T
SSA_2293	T
SSA_2316	T
SSA_2329	T
SSA_2337	T
SSA_2346	T
SSA_2351	T
SSA_2361	T
SSA_2367	T
SSA_0075	V
SSA_0077	V
SSA_0148	V
SSA_0163	V
SSA_0164	V
SSA_0171	V
SSA_0184	V
SSA_0215	V
SSA_0264	V
SSA_0283	V
SSA_0294	V
SSA_0315	V
SSA_0342	V
SSA_0357	V
SSA_0373	V
SSA_0417	V
SSA_0432	V

SSA_0454	V
SSA_0481	V
SSA_0486	V
SSA_0499	V
SSA_0558	V
SSA_0621	V
SSA_0639	V
SSA_0651	V
SSA_0663	V
SSA_0752	V
SSA_0790	V
SSA_0924	V
SSA_0970	V
SSA_0975	V
SSA_1007	V
SSA_1014	V
SSA_1215	V
SSA_1219	V
SSA_1231	V
SSA_1236	V
SSA_1243	V
SSA_1254	V
SSA_1258	V
SSA_1334	V
SSA_1354	V
SSA_1367	V
SSA_1449	V
SSA_1472	V
SSA_1590	V
SSA_1683	V
SSA_1696	V
SSA_1711	V
SSA_1713	V
SSA_1749	V
SSA_1755	V
SSA_1760	V
SSA_1767	V
SSA_1859	V
SSA_1868	V
SSA_1905	V
SSA_1924	V
SSA_1988	V
SSA_2009	V
SSA_2040	V
SSA_2052	V
SSA_2063	V
SSA_2092	V
SSA_2099	V
SSA_2105	V
SSA_2106	V
SSA_2133	V
SSA_2138	V
SSA_2141	V
SSA_2142	V
SSA_2156	V
SSA_2194	V

SSA_2215	V
SSA_2273	V
SSA_2274	V
SSA_2349	V
SSA_2354	V
SSA_1199	W
SSA_1774	W
SSA_1844	W
SSA_1973	W
SSA_2373	W
SSA_2390	Y
SSA_0151	Y
SSA_0183	Y
SSA_0214	Y
SSA_0246	Y
SSA_0258	Y
SSA_0259	Y
SSA_0262	Y
SSA_0322	Y
SSA_0483	Y
SSA_0514	Y
SSA_0662	Y
SSA_0833	Y
SSA_0867	Y
SSA_0910	Y
SSA_0923	Y
SSA_1079	Y
SSA_1119	Y
SSA_1218	Y
SSA_1583	Y
SSA_1610	Y
SSA_1613	Y
SSA_1638	Y
SSA_1695	Y
SSA_1825	Y
SSA_2079	Y
SSA_2220	Y
SSA_2225	Y
SSA_2254	Y
SSA_2328	Y

* The N-terminal amino acid (amino acid second to N-terminal methionine) for every protein was identified using Biopython scripts.

Supplementary Table 3. Distribution of amino acids in *S. sanguinis* proteins.

N-terminal amino acid	Essential		Non-essential		chi-square statistic	p-value	Statistical significance
	**count**	**percentage**	**count**	**percentage**			
**A**	37	16.97	167	8.14	18.8	0.000014	Yes
**C**	1	0.46	2	0.1	1.9485	0.162748	
**D**	7	3.21	69	3.36	0.014	0.905848	
**E**	11	5.05	120	5.85	0.2331	0.629206	
**F**	4	1.83	52	2.53	0.4005	0.5269	
**G**	7	3.21	51	2.49	0.417	0.5186	
**H**	1	0.46	17	0.83	0.2085	0.647964	
**I**	16	7.34	117	5.7	0.958	0.3276	
**K**	29	13.3	492	23.98	12.6962	0.000366	Yes
**L**	8	3.67	126	6.14	2.1655	0.014113	Yes
**M**	9	4.13	32	1.56	7.333	0.0068	Yes
**N**	17	7.8	127	6.19	0.859	0.3541	
**P**	3	1.38	34	1.66	0.097	0.7557	
**Q**	4	1.83	45	2.19	0.120	0.7294	
**R**	2	0.92	97	4.73	6.8571	0.008829	Yes
**S**	25	11.47	184	8.97	1.475	0.2246	
**T**	23	10.55	207	10.09	0.046	0.83	
**V**	10	4.59	78	3.8	0.327	0.5676	
**W**	0	0	5	0.24	0.2085	0.647964	
**Y**	4	1.83	30	1.46	0.1857	0.666512	
Total	218	100%	2052	100%			

Supplementary Table 4. Degradation-prone proteins

	Essential	Non-essential
**N-terminal amino acid**	**count**	**count**
**F**	4	52
**K**	29	492
**L**	8	126
**R**	2	97
**W**	0	5
**Y**	4	30
**Total**	47	802
**percentage**	21.56	39.08

Supplementary Table 5. Amino acid composition of essential proteins in *S. sanguinis.*

Amino acid	V	R	N	S	K	T	M	I	Q	H	P	L	W	C	Y	F	G	E	D	A	COG
SSA_0862	2.2	3.91	3.912	5.38	9.54	5.62	2.2	6.6	5.87	2.2	3.423	11.2	1.22	0	3.91	4.65	6.36	9.05	4.89	7.824	V
SSA_0133	3.13	11.7	3.125	3.91	7.03	10.9	3.13	7.03	2.34	0.78	2.344	8.59	0	0	3.91	1.56	4.69	9.38	3.91	12.5	J
SSA_0720	3.16	3.45	2.586	4.6	9.2	4.6	1.15	7.18	6.32	0.57	2.299	15.5	0	0.29	2.87	6.32	4.6	8.05	8.05	9.195	L
SSA_0338	3.83	3.06	4.082	7.65	7.14	4.59	1.28	6.63	4.34	1.79	3.571	11.7	0.51	0.51	4.59	4.08	6.38	7.14	6.12	10.97	I
SSA_0010	4.1	9.02	4.918	5.74	9.02	4.1	1.64	6.56	9.02	0	3.279	11.5	0.82	0	9.84	3.28	1.64	6.56	4.92	4.098	D
SSA_0997	4.14	4.32	5.396	6.47	5.76	6.12	1.98	8.45	4.86	1.62	2.878	10.3	0.54	0.72	1.98	3.42	5.58	8.45	5.94	11.15	L
SSA_0547	4.17	6.67	2.5	7.5	8.33	3.33	0.83	7.5	1.67	1.67	1.667	10	2.5	0	1.67	5.83	8.33	10.8	3.33	11.67	I
SSA_1302	4.17	6.25	1.667	4.17	5.42	4.58	3.75	7.08	2.08	4.17	4.583	10.4	0.42	0.42	5.83	2.92	6.67	12.1	7.08	6.25	J
SSA_0784	4.27	3.66	4.268	5.49	9.76	3.05	1.22	11	4.88	0	0	9.76	0.61	0	0.61	2.44	6.71	9.76	6.1	16.46	C
SSA_1940	4.32	3.7	3.704	11.1	4.94	6.48	2.47	7.72	4.01	1.23	2.16	10.2	0.93	0.93	1.54	4.32	8.33	5.56	6.48	9.877	I
SSA_0334	4.44	3.49	2.54	10.2	7.62	6.03	3.49	4.76	3.81	1.27	4.444	9.21	0.95	1.27	4.44	3.81	4.13	6.35	7.94	9.841	I
SSA_0999	4.5	3.54	3.859	7.07	6.75	5.47	1.93	8.68	5.47	0.64	3.537	9.32	1.93	0.96	4.18	4.82	7.72	7.4	5.47	6.752	H
SSA_1606	4.55	8.59	2.02	4.55	3.03	4.55	2.02	7.07	10.6	0.51	2.525	13.6	1.01	0	1.52	3.54	6.57	9.09	6.57	8.081	H
SSA_2136	4.55	27.3	2.273	4.55	13.6	4.55	4.55	2.27	2.27	4.55	2.273	4.55	0	0	2.27	2.27	6.82	0	0	11.36	J
SSA_1092	4.71	4.71	4.118	3.53	7.65	5.29	1.76	8.24	5.88	2.94	4.118	8.82	2.35	0	4.71	4.71	5.29	7.06	7.06	7.059	H
SSA_2144	4.74	5.15	4.33	3.3	6.8	5.57	2.68	7.42	3.09	2.27	4.124	8.66	1.44	0	4.54	4.12	6.6	11.5	5.98	7.629	J
SSA_1213	4.8	3.54	4.798	7.58	6.06	6.57	2.53	6.82	4.8	4.55	3.535	10.4	0	0.25	2.02	4.29	6.31	6.31	5.56	9.343	E
SSA_0571	4.82	3.77	5.66	5.24	7.76	4.19	2.1	7.76	4.4	1.47	4.193	7.76	0.84	0	4.19	3.77	7.34	9.64	5.45	9.644	J
SSA_0944	4.87	4.49	6.367	5.62	7.12	6.37	3.37	8.99	4.49	2.62	2.996	7.87	0.75	0.37	4.49	3.75	5.62	6.74	5.99	7.116	P
SSA_0106	4.9	8.82	2.941	2.94	9.8	10.8	2.94	9.8	1.96	2.94	4.902	8.82	0	0	2.94	0.98	2.94	6.86	4.9	9.804	J
SSA_2391	4.92	9.84	1.639	6.56	16.4	4.92	3.28	3.28	3.28	1.64	4.918	3.28	1.64	6.56	4.92	4.92	4.92	3.28	0	9.836	J
SSA_0013	4.94	5.88	4.706	7.06	8	3.06	1.88	7.06	7.29	3.53	2.118	12.5	0.24	0.24	3.06	6.59	4	7.53	5.65	4.706	D
SSA_0198	4.96	3.88	2.802	7.33	4.31	4.53	1.29	4.96	7.11	3.23	4.095	14.7	0.86	1.29	3.66	2.59	5.6	7.76	5.39	9.698	H
SSA_0335	5.01	3.83	1.475	8.55	6.78	3.83	3.24	6.19	5.01	0.59	2.95	12.7	0.88	1.47	4.13	2.95	7.37	6.78	5.6	10.62	I
SSA_0768	5.02	1.88	7.21	5.96	6.58	6.9	1.88	5.96	3.13	1.57	2.194	11	2.19	0	5.96	5.33	5.64	9.09	7.21	5.329	F
SSA_1498	5.04	11.8	4.202	3.36	12.6	2.52	3.36	4.2	2.52	1.68	0	10.9	0.84	0	5.88	2.52	5.88	2.52	4.2	15.97	J
SSA_1233	5.09	1.85	3.704	5.56	5.56	6.94	1.39	13	1.85	1.85	4.167	14.8	0.93	0	2.31	8.8	9.72	1.85	1.39	9.259	S
SSA_0846	5.14	5.52	3.461	5.14	7.11	4.4	2.71	5.8	4.77	2.15	3.555	11.6	0.56	0.28	4.4	6.17	5.89	7.67	6.36	7.297	L
SSA_1452	5.31	5.58	2.47	7.23	6.04	5.22	1.56	4.48	6.04	1.83	2.653	12.8	0.55	0.18	4.21	4.85	4.48	8.78	6.22	9.515	L
SSA_1806	5.33	3.05	7.614	8.88	10.4	3.3	1.02	7.36	4.57	1.27	2.284	9.14	0.76	0.51	4.57	6.85	4.06	7.87	6.6	4.569	L
SSA_0336	5.37	6.27	3.881	7.46	3.88	2.69	2.39	4.78	6.27	2.39	3.881	14	1.19	0.9	3.28	3.28	7.76	7.16	5.97	7.164	C
SSA_1091	5.38	4.3	7.527	3.58	6.45	4.3	2.51	7.89	4.66	2.51	3.584	8.96	2.15	0.72	4.66	5.73	4.66	6.45	7.53	6.452	F
SSA_0337	5.42	3.54	3.538	5.9	6.84	5.42	3.07	4.48	6.13	1.42	3.538	12.7	1.18	0.47	2.36	2.83	6.84	5.42	5.19	13.68	I
SSA_2262	5.52	4.45	5.338	5.87	7.47	3.38	1.6	7.47	3.91	1.07	3.381	9.96	1.25	0	3.91	4.27	6.05	8.01	7.65	9.431	J
SSA_1167	5.53	4.96	5.153	4.77	9.16	4.39	6.68	7.25	4.96	0.57	4.389	8.59	0	0	0.57	3.05	8.78	7.06	6.3	7.824	U
SSA_2356	5.54	7.1	4.878	6.87	4.88	5.1	1.11	10.2	4.43	0.44	1.774	9.76	0.22	0	3.1	2.66	6.21	9.53	6.65	9.534	L
SSA_1094	5.64	5.64	6.154	7.18	10.3	3.59	1.54	7.18	1.54	2.05	3.59	9.74	1.54	0	3.59	4.1	4.62	5.13	9.23	7.692	R
SSA_1529	5.65	5.85	3.831	2.42	6.05	6.25	3.02	8.06	3.63	3.02	3.629	7.86	0.2	0	2.82	5.65	7.06	10.9	6.45	7.661	J
SSA_1189	5.65	4.95	3.887	4.24	10.6	5.3	3.53	7.07	4.59	0.71	4.594	11.3	1.41	0.35	2.12	3.89	6.01	5.3	8.13	6.36	R
SSA_1811	5.7	4.22	4.43	6.33	5.49	4.85	2.53	7.38	3.38	1.48	4.008	7.81	1.05	0.42	5.27	3.59	8.23	6.75	6.96	10.13	G
SSA_0692	5.7	3.51	4.825	3.95	6.58	6.14	2.85	5.04	3.95	1.54	3.509	12.3	0.22	0.44	3.51	3.95	6.58	7.46	7.24	10.75	M
SSA_1721	5.74	6.08	4.392	8.45	5.07	3.72	1.69	5.41	9.46	1.69	2.027	11.8	0.68	2.03	2.03	6.42	3.38	8.45	3.72	7.77	L
SSA_0800	5.75	3.07	7.28	4.6	6.13	3.45	1.53	6.9	3.83	2.3	1.533	8.43	0	1.15	8.05	4.21	10.3	8.81	6.9	5.747	R
SSA_1419	5.75	3.1	5.752	5.75	7.52	7.08	1.33	4.42	5.31	1.77	3.097	13.3	1.77	0	1.77	4.87	3.54	8.85	6.19	8.85	L
SSA_1555	5.76	5.35	4.527	8.23	6.38	3.91	1.85	5.35	4.32	2.26	3.909	9.05	1.44	0	3.5	7.61	5.56	8.85	5.97	6.173	G
SSA_0001	5.78	4.44	5.556	5.33	7.33	6.67	1.11	9.33	4.67	2	3.333	10	0.89	0	2.44	5.11	5.56	7.56	6.67	6.222	L
SSA_0100	5.8	4.43	3.75	4.09	7.16	6.14	2.39	6.93	5.11	1.93	3.636	11.4	0.34	0	3.98	4.09	5.45	8.75	6.25	8.409	L
SSA_0787	5.8	4.1	5.119	6.83	5.8	5.8	4.1	8.19	5.12	2.39	2.389	8.19	0	0.68	3.75	2.39	4.44	7.85	5.8	11.26	C
SSA_0333	5.82	3.42	2.397	5.82	6.16	4.11	3.77	7.53	3.77	2.4	2.055	11.3	0.68	2.05	1.37	3.08	8.56	8.9	3.77	13.01	I
SSA_0770	5.84	4.17	5.981	7.65	6.12	5.29	2.36	7.23	3.48	1.81	3.477	8.21	0.42	0.7	5.98	4.87	6.12	7.79	5.42	7.093	F
SSA_1571	5.87	6.34	3.091	4.02	5.72	4.95	2.94	6.49	4.48	3.71	4.019	8.19	1.08	0.31	4.02	4.33	6.18	10	6.96	7.264	J
SSA_1931	5.92	4.88	3.484	5.23	6.62	7.67	4.18	4.88	3.14	2.44	3.833	8.71	0	1.39	2.09	5.57	8.01	7.32	5.23	9.408	I
SSA_0824	5.98	5.48	5.15	6.15	5.65	4.15	2.82	6.31	5.32	3.82	3.488	9.47	0.5	0.83	3.82	4.15	4.32	8.97	6.15	7.475	L
SSA_1086	6.02	6.77	2.757	4.51	5.76	3.51	1.5	5.26	4.01	1	3.008	13	0.75	0.75	2.26	6.27	5.01	10	8.27	9.524	J
SSA_0063	6.02	6.33	2.41	3.31	4.52	6.33	4.52	7.53	3.01	1.81	3.614	11.4	0	0	4.22	2.71	8.43	9.94	6.93	6.928	L
SSA_1800	6.08	4.28	4.73	6.31	4.95	5.86	1.8	7.66	3.38	3.6	2.703	7.43	0	0	5.41	6.76	7.88	6.31	7.21	7.658	M
SSA_1925	6.12	6.12	4.471	4.47	6.59	6.82	2.59	4.47	2.59	1.88	3.765	9.41	0.94	0.47	3.53	4	5.88	9.65	7.06	9.176	J

SSA_0785	6.18	3.37	3.371	8.43	10.7	2.81	1.12	10.1	7.87	2.25	1.685	11.8	0	0	1.69	5.06	3.93	10.7	4.49	4.494	C
SSA_1880	6.23	5.57	3.934	4.92	3.61	4.92	1.97	3.93	4.92	2.95	4.918	10.2	1.97	0.98	5.57	4.26	6.89	10.2	4.92	7.213	J
SSA_1930	6.25	7.03	4.688	3.91	5.08	3.52	3.52	7.03	4.69	1.56	3.516	10.2	0.78	0	1.95	3.91	9.77	8.98	3.91	9.766	I
SSA_0437	6.25	7.29	6.25	3.13	7.29	2.08	2.08	10.4	1.04	2.08	1.042	9.38	1.04	0	4.17	3.13	2.08	10.4	9.38	11.46	J
SSA_0912	6.3	6.02	2.579	5.44	6.3	4.87	4.3	6.59	5.73	2.58	3.438	8.31	0.29	1.15	2.29	4.87	8.02	9.17	5.73	6.017	J
SSA_2073	6.43	5.62	5.622	4.82	8.84	4.82	3.21	5.62	2.81	1.2	4.819	10.4	1.61	0	4.02	4.82	5.62	7.23	4.42	8.032	I
SSA_1903	6.44	6.82	6.439	6.06	3.79	6.82	3.79	4.55	5.3	1.89	4.167	11.7	3.03	0	6.44	1.89	6.44	3.79	6.06	4.545	M
SSA_1739	6.44	3.53	3.742	6.44	7.28	5.82	2.08	6.24	3.12	2.29	2.703	10.8	0	0.21	4.37	5.41	6.86	7.69	6.03	8.94	M
SSA_0869	6.46	4.62	3.385	6.15	6.15	6.46	4.31	4.31	4.92	2.15	2.462	9.23	1.23	0	3.69	3.69	7.08	9.54	8.62	5.538	J
SSA_0801	6.49	3.58	5.145	4.47	6.94	8.05	2.24	7.16	3.13	1.57	2.908	9.84	0.45	1.34	4.25	4.7	7.16	6.26	5.15	9.172	M
SSA_2183	6.52	3.7	4.565	5.87	7.39	4.57	1.52	6.3	3.48	1.3	3.261	9.13	1.3	0	5	4.78	7.39	7.61	6.96	9.348	G
SSA_0570	6.56	2.46	3.279	9.02	6.97	5.74	2.05	7.17	3.69	1.23	3.689	8.2	0.2	0	4.1	4.1	9.43	6.56	5.94	9.631	J
SSA_1863	6.57	4.01	2.555	5.47	7.3	4.38	1.46	6.2	4.74	1.82	3.285	8.39	1.09	0	3.65	4.01	7.66	8.03	6.93	12.41	H
SSA_1522	6.59	3.41	4.146	6.83	3.66	4.15	2.93	12.4	2.68	1.46	2.683	12.9	1.95	0	3.41	7.32	10	3.66	2.2	7.561	D
SSA_0128	6.6	5.19	5.66	2.83	6.13	5.66	1.89	8.49	4.72	3.3	3.774	9.43	0	0	2.83	2.83	7.55	7.55	8.96	6.604	F
SSA_1722	6.6	6.6	2.358	5.66	4.25	3.77	1.42	6.6	4.72	1.42	4.245	14.2	0.47	0	2.83	2.83	8.02	8.49	7.08	8.491	F
SSA_2005	6.63	5.57	4.775	5.31	7.43	5.04	1.33	3.18	4.51	1.86	3.979	4.77	0	2.12	3.98	4.51	14.3	6.9	6.37	7.427	O
SSA_1805	6.69	6.02	4.013	6.35	6.35	3.68	3.01	5.69	7.02	2.01	3.01	9.7	0.67	0	4.01	5.02	4.01	9.03	5.69	8.027	L
SSA_2368	6.7	2.23	1.676	3.35	6.15	6.7	5.59	11.2	1.12	1.68	2.793	13.4	1.68	0.56	3.91	8.38	5.59	2.79	3.35	11.17	I
SSA_0625	6.71	7.93	5.488	6.71	4.88	4.27	2.44	7.93	5.49	1.83	1.829	9.15	0	0	3.05	6.71	4.88	9.15	4.88	6.707	H
SSA_2375	6.74	4.99	3.812	3.81	6.16	4.69	4.11	6.16	5.57	2.35	4.985	9.09	0	0.29	4.4	3.52	7.62	7.04	6.74	7.918	J
SSA_1848	6.75	3.86	2.572	5.79	6.43	4.5	4.18	6.43	5.14	0.96	6.431	10.9	0.64	0.32	2.57	3.54	8.36	6.11	5.79	8.682	J
SSA_1232	6.78	5.39	4.931	4.78	8.63	7.09	2.47	5.08	2.16	1.54	2.773	9.24	0.77	0	3.24	3.54	9.55	7.55	7.09	7.396	L
SSA_2263	6.79	3.7	7.407	4.94	6.17	4.32	3.09	6.79	3.7	1.85	3.086	9.26	0	1.23	4.32	6.17	8.64	9.26	5.56	3.704	F
SSA_1500	6.82	5.68	2.273	2.84	14.8	3.41	3.98	8.52	7.39	0.57	4.545	6.82	0	0	1.14	4.55	5.11	6.82	7.39	7.386	J
SSA_0807	6.88	6.42	3.44	4.36	5.5	4.82	3.44	4.59	2.52	1.38	4.128	9.4	0.46	0	2.29	5.28	11	9.17	7.11	7.798	R
SSA_2169	6.9	3.76	2.821	4.39	10.3	5.33	2.19	7.52	3.76	2.19	4.702	10	0	0	2.51	4.08	7.21	7.84	7.84	6.583	M
SSA_0713	6.91	6.1	4.878	4.88	8.13	2.44	4.47	9.35	1.63	2.44	6.098	6.1	2.44	0.41	3.25	7.32	6.1	4.07	4.88	8.13	I
SSA_0886	6.91	4.38	3.917	4.61	5.76	6.22	1.84	7.6	2.76	0.92	2.765	6.91	0.46	0.23	4.38	3.69	10.4	8.53	6.68	11.06	G
SSA_0197	6.94	5.36	3.785	6.62	4.42	4.42	2.52	7.26	2.84	2.21	4.101	9.15	0.63	0.63	0.95	4.73	8.2	9.15	4.73	11.36	H
SSA_0771	6.94	2.78	6.944	4.17	9.72	6.94	2.78	5.56	5.56	2.78	4.167	6.94	0	2.78	2.78	6.94	4.17	6.94	5.56	5.556	O
SSA_1619	7.03	7.03	3.784	5.41	9.73	5.95	2.16	8.65	2.16	2.16	2.703	8.11	0	0	1.08	1.08	3.24	11.4	7.03	11.35	J
SSA_2284	7.04	6.1	3.756	5.4	5.4	5.4	2.11	4.93	4.46	1.17	4.225	9.39	0.23	0.47	5.16	4.69	7.04	8.92	5.63	8.451	J
SSA_0936	7.1	7.74	3.226	5.48	4.84	4.84	2.58	6.77	3.23	2.9	2.903	8.71	0.65	0	3.87	5.81	8.06	6.77	7.42	7.097	H
SSA_0756	7.11	3.9	3.67	4.82	6.19	5.28	2.41	6.31	3.9	2.64	3.555	7.91	1.15	0.11	2.64	4.24	7.45	9.98	5.85	10.89	J
SSA_1430	7.12	5.18	3.236	6.15	7.77	4.85	1.62	7.12	3.56	3.56	2.913	10	0.32	0.65	1.94	4.85	8.09	7.12	6.15	7.767	R
SSA_0783	7.14	2.1	6.303	4.62	4.2	8.4	4.62	7.56	2.1	1.26	2.941	11.8	2.1	0.84	4.62	8.82	7.56	4.2	1.68	7.143	C
SSA_0312	7.14	4.29	4.464	5.18	5.54	4.64	2.32	10.9	3.57	2.86	4.821	8.21	0	0.54	2.68	4.11	9.11	6.61	5.18	7.857	R
SSA_2044	7.16	5.59	4.474	3.58	6.04	6.71	1.79	6.49	2.46	2.68	2.685	5.82	1.57	0.45	3.8	5.82	6.49	8.28	7.61	10.51	J
SSA_1992	7.17	2.39	4.437	3.41	7.51	2.73	2.05	7.85	2.73	2.73	3.413	7.17	0.68	0.68	2.73	3.41	9.22	9.22	6.14	14.33	G
SSA_0289	7.17	3.82	4.182	4.18	7.05	5.97	1.91	5.38	2.75	2.03	4.779	8.24	2.99	0.12	4.66	3.46	6.33	9.2	6.33	9.438	J
SSA_1738	7.2	3.32	2.952	6.46	4.06	5.17	4.06	11.1	3.87	1.11	3.506	13.7	1.48	0	4.24	6.83	7.75	2.58	1.48	9.225	R
SSA_1226	7.23	6.13	4.902	6.13	7.35	5.64	2.45	8.21	2.33	1.35	3.431	8.82	0.49	0	3.43	3.68	5.27	8.46	6.99	7.721	L
SSA_2066	7.25	4.04	4.514	4.17	6.22	5.34	3.28	5.88	4.17	2.94	4.241	8.41	0.55	0.82	3.63	5.13	6.84	8.55	6.36	7.661	L
SSA_0543	7.27	6.67	4.529	5.84	6.44	5.13	3.1	6.67	3.81	2.15	2.622	8.22	0.24	0.6	3.93	2.38	6.08	8.82	8.1	7.39	U
SSA_0438	7.32	7.32	10.98	9.76	1.83	4.27	1.83	4.27	7.32	0	3.659	4.88	1.22	0	3.05	4.88	9.15	5.49	5.49	7.317	L
SSA_1935	7.32	2.93	5.122	5.61	6.34	6.1	1.95	6.59	2.2	2.93	4.39	5.61	0.49	0.73	3.66	4.39	10.2	7.56	4.39	11.46	IQ
SSA_1184	7.32	5.02	5.308	5.45	11.5	5.6	2.3	6.03	4.45	1.29	4.017	5.6	0.86	1.58	3.73	4.3	6.03	7.17	5.88	6.6	L
SSA_2350	7.35	8.57	5.306	5.31	6.53	4.49	2.04	4.9	4.49	1.22	3.673	12.2	0.82	0.41	3.27	5.31	7.35	7.35	2.86	6.531	J
SSA_0113	7.37	7.83	3.226	2.3	9.22	4.61	1.84	8.29	4.15	2.76	2.304	6.91	1.84	0	3.23	0.92	9.68	6.45	5.53	11.52	J
SSA_0688	7.39	4.78	4.783	4.78	8.26	3.48	1.3	4.78	3.04	3.04	3.913	9.13	2.61	0	2.61	3.91	5.65	8.7	8.7	9.13	G
SSA_0174	7.4	4.77	4.773	5.49	6.44	5.73	2.63	5.25	5.73	1.91	1.909	10	1.19	0	4.53	4.53	7.4	7.4	6.44	6.444	J
SSA_1864	7.41	4.94	5.761	3.29	6.58	5.76	2.06	6.17	4.94	1.44	3.086	9.88	1.03	0.41	5.14	4.12	6.17	6.38	7.2	8.23	H
SSA_0020	7.48	5.61	3.115	7.17	5.92	3.43	3.74	10.3	3.74	2.49	4.05	9.03	0	0.93	2.18	2.8	5.92	6.23	6.54	9.346	FE
SSA_1872	7.48	6.54	3.738	8.41	7.48	7.48	3.74	5.61	9.35	0	1.869	10.3	0	0	1.87	2.8	1.87	10.3	5.61	5.607	D
SSA_2360	7.51	4.02	3.485	4.02	6.97	5.36	3.49	4.02	5.9	1.88	3.217	7.24	0.8	1.34	5.36	5.09	10.5	5.63	7.77	6.434	J
SSA_1484	7.52	4.75	3.681	6.6	6.29	4.91	0.77	5.67	4.29	1.69	3.988	11.7	0.61	0.92	3.07	2.91	5.67	9.82	5.52	9.663	L
SSA_0111	7.53	7.53	1.075	4.3	15.1	6.45	3.23	5.38	2.15	4.3	4.301	3.23	1.08	0	4.3	4.3	7.53	5.38	6.45	6.452	J
SSA_1819	7.53	5.06	4.719	4.38	6.52	5.17	2.7	6.52	3.15	1.91	4.494	7.75	2.92	0.11	3.48	4.38	5.84	8.31	7.42	7.64	J

SSA_2007	7.55	3.12	4.105	4.43	8.7	6.9	1.97	6.73	4.27	0.66	3.12	7.55	0	0	1.15	2.63	7.88	8.54	8.7	11.99	O
SSA_0825	7.57	7.84	4.054	2.97	5.41	6.22	2.43	7.3	5.41	1.08	3.514	10.5	0.54	0	1.35	2.7	4.86	11.4	8.65	6.216	K
SSA_0782	7.58	3.03	3.03	6.06	3.03	4.55	6.06	9.09	3.03	0	1.515	16.7	0	3.03	1.52	7.58	9.09	4.55	0	10.61	C
SSA_0786	7.71	5.95	1.542	6.83	4.19	6.39	1.76	8.37	4.41	1.1	4.626	9.69	0	0.22	3.08	3.74	8.37	6.61	6.83	8.59	C
SSA_1310	7.78	8.89	5.556	7.78	11.1	5.56	2.22	5.56	2.22	1.11	4.444	7.78	1.11	0	3.33	3.33	6.67	5.56	5.56	4.444	J
SSA_1604	7.79	3.9	5.195	7.79	3.9	3.9	6.49	11.7	3.9	0	2.597	14.3	1.3	0	1.3	7.79	2.6	2.6	2.6	10.39	U
SSA_1879	7.81	5.01	4.124	4.57	5.74	5.15	2.5	4.71	3.09	1.77	2.798	11.8	0.44	0	2.06	5.3	5.6	9.87	6.77	10.9	J
SSA_1871	7.87	2.89	6.824	6.04	7.87	8.01	3.02	5.64	5.51	0.52	3.15	6.96	0.79	0	4.2	3.81	8.27	6.96	4.86	6.824	M
SSA_0131	7.87	8.66	3.937	7.87	7.87	6.3	2.36	6.3	1.57	3.94	5.512	3.15	0.79	0	0	2.36	8.66	4.72	1.57	16.54	J
SSA_2240	7.91	6.47	3.597	4.32	7.19	3.6	2.88	7.19	5.04	0.72	3.597	10.8	0	0	2.88	3.6	8.63	8.63	5.76	7.194	L
SSA_0002	7.94	4.23	7.143	9.26	5.03	10.1	0.79	9.79	3.17	1.32	3.439	9.26	0	0	1.06	5.56	3.97	7.67	5.03	5.291	L
SSA_0006	7.94	2.12	5.291	3.7	11.1	5.29	2.65	8.47	3.17	2.65	1.587	8.99	0	0.53	4.76	4.76	10.1	4.76	7.94	4.233	J
SSA_1703	7.96	3.75	3.754	5.26	6.76	4.8	3.3	5.56	3	2.1	4.054	9.76	1.65	0.3	5.26	3.6	6.61	8.41	7.06	7.057	J
SSA_2107	7.96	3.48	3.483	6.97	5.64	6.3	2.32	7.13	2.99	2.99	2.653	9.78	0.33	0.5	3.98	2.32	7.79	7.46	5.8	10.12	M
SSA_0878	8.01	5.55	4.16	5.08	6.01	6.32	2.47	6.78	4.16	2.62	2.928	6.93	0.31	0.15	3.54	3.7	8.47	9.4	6.16	7.242	L
SSA_1152	8.08	5.85	3.343	4.46	7.8	5.29	2.79	5.57	6.13	1.67	2.228	8.08	0.28	0	3.9	1.39	5.57	10.9	8.36	8.357	J
SSA_0200	8.15	4.81	2.222	4.44	5.93	4.07	1.48	6.3	5.93	1.85	5.556	13.3	2.22	0.74	3.33	2.59	4.44	9.26	5.93	7.407	H
SSA_0199	8.15	4.35	2.717	2.17	7.07	5.43	4.89	5.98	3.8	3.8	2.717	7.07	0.54	1.09	3.26	3.26	7.61	10.3	3.8	11.96	H
SSA_1062	8.25	8.25	4.124	5.15	10.3	5.15	2.06	3.09	5.15	3.09	1.031	7.22	0	0	2.06	3.09	15.5	2.06	5.15	9.278	J
SSA_2069	8.28	5.84	3.734	6.01	5.84	4.55	2.6	5.84	2.6	1.46	3.734	9.25	0.65	0.32	3.73	3.57	6.98	8.6	7.47	8.929	J
SSA_0177	8.31	7.49	3.868	3.79	6.26	6.01	3.37	7.16	2.96	1.89	4.362	8.72	0.58	0.82	2.22	2.8	7.65	8.89	5.27	7.572	K
SSA_1932	8.35	4.18	3.956	5.71	5.27	3.3	3.3	7.69	3.74	1.98	3.956	6.59	0	0.88	3.3	3.96	8.57	8.79	4.84	11.65	I
SSA_2109	8.37	5.05	3.463	4.62	5.63	6.78	3.32	6.64	3.61	1.88	4.185	6.64	0.58	0.14	2.74	3.9	7.65	10.1	5.92	8.802	J
SSA_1557	8.4	3.91	3.32	5.47	5.86	5.27	1.37	5.27	5.86	0.98	2.93	8.2	0.2	0	1.56	3.52	6.64	15.8	6.05	9.375	U
SSA_0652	8.44	2.22	5.333	5.56	6.44	5.33	2.89	5.78	4.22	1.56	3.333	9.78	0.67	0	2.22	3.78	8.22	6.89	5.78	11.56	M
SSA_1220	8.47	7.63	4.843	4.36	5.81	6.54	3.39	7.38	2.78	1.94	2.542	8.47	0.24	0	2.54	2.91	7.63	8.23	6.9	7.385	L
SSA_0691	8.5	3.68	3.116	6.52	6.52	3.97	3.97	5.67	3.68	0.57	3.966	9.92	0.57	0.57	3.97	4.25	7.37	9.07	5.95	8.215	M
SSA_0127	8.51	2.76	3.448	6.67	5.52	6.9	2.76	10.3	3.68	0	3.908	11.5	0.69	0.23	3.91	6.9	7.82	2.76	2.99	8.736	U
SSA_1865	8.55	5.26	3.289	4.28	4.61	5.26	1.64	7.24	2.96	1.97	2.303	6.58	0.66	0.66	3.29	4.61	10.2	9.21	6.91	10.53	O
SSA_1565	8.58	6.87	3.004	3	6.44	5.58	2.58	7.3	2.15	2.58	3.863	11.2	0.43	0	3	3.43	5.58	11.2	7.73	5.579	TK
SSA_0847	8.63	5.06	2.976	5.36	5.06	4.76	3.27	9.23	1.49	2.68	2.679	5.95	0.3	0	2.08	2.68	13.1	7.74	6.25	10.71	G
SSA_1047	8.64	5.98	4.651	4.98	4.65	3.99	2.66	7.97	3.99	2.99	2.99	8.64	0.33	1	2.66	4.65	10.6	7.97	3.65	6.977	M
SSA_0132	8.65	4.17	5.449	5.13	7.05	7.37	1.92	8.01	0.96	0.96	3.526	10.3	0	0.32	2.56	2.56	7.37	9.62	7.69	6.41	K
SSA_0661	8.71	4.41	3.656	5.91	5.48	5.59	1.72	5.91	4.09	2.26	4.194	8.06	2.47	0.54	4.09	4.3	5.91	8.82	6.13	7.742	J
SSA_1803	8.72	5.73	4.817	4.59	6.19	5.73	3.44	10.1	2.52	1.83	4.358	5.73	0.69	0	2.52	4.59	5.96	7.34	7.34	7.798	R
SSA_0114	8.76	10.2	0.73	3.65	12.4	3.65	4.38	5.11	1.46	2.92	3.65	3.65	2.19	0.73	2.19	3.65	10.9	8.76	0	10.95	J
SSA_0871	8.77	4.55	5.844	8.44	5.84	6.82	3.9	10.1	2.92	0.65	1.948	9.74	1.3	0	3.57	4.87	6.17	4.87	3.57	6.169	D
SSA_0112	8.77	7.89	8.772	7.02	9.65	5.26	2.63	7.89	0	0.88	4.386	5.26	0	0	0	3.51	4.39	6.14	1.75	15.79	J
SSA_1491	8.77	4.56	1.754	4.56	5.26	5.26	3.86	6.32	3.86	3.51	3.509	7.37	1.4	2.11	3.51	2.11	10.5	7.72	8.07	5.965	J
SSA_0176	8.78	6.65	4.437	4.97	5.32	4.79	3.02	6.39	3.19	1.95	4.614	7.54	0.44	0.18	3.11	3.02	8.16	8.34	7.99	7.098	K
SSA_0683	8.79	4.4	3.297	3.3	15.4	4.4	1.1	4.4	3.3	0	2.198	5.49	0	0	1.1	4.4	6.59	7.69	4.4	19.78	L
SSA_0440	8.86	13.9	3.797	3.8	11.4	6.33	3.8	5.06	3.8	0	2.532	5.06	0	0	3.8	3.8	5.06	5.06	5.06	8.861	J
SSA_1209	8.87	6.74	5.674	6.38	6.74	7.45	2.13	8.87	3.9	3.19	2.837	6.74	0.71	0.35	3.19	4.61	5.67	5.32	6.38	4.255	G
SSA_0941	8.87	1.02	6.143	10.6	7.17	6.14	2.73	5.8	3.75	0.68	1.365	7.85	1.37	0.34	2.39	2.39	9.56	5.8	6.83	9.215	P
SSA_2202	8.93	2.59	4.323	3.46	8.65	4.61	3.17	5.48	3.46	1.44	2.017	7.2	0.29	0	2.31	2.88	6.63	10.1	6.34	16.14	J
SSA_2110	8.97	12.2	5.769	3.21	5.13	6.41	3.85	3.85	2.56	1.92	3.846	7.69	1.28	0	1.92	2.56	5.77	7.69	4.49	10.9	J
SSA_0848	8.98	5.19	5.19	3.39	6.59	8.18	2.99	8.18	2.79	1	2.595	5.99	0.4	0.2	1.6	3.59	8.18	8.38	7.39	9.182	G
SSA_0653	8.99	3.37	3.371	6.46	7.58	3.93	1.97	5.34	5.62	2.53	2.809	11.2	0.84	0.28	3.93	4.78	7.87	5.62	5.34	8.146	M
SSA_0110	9.03	9.03	6.137	4.69	8.66	5.78	1.44	5.05	2.17	2.89	5.415	7.58	0.72	0.36	2.17	1.08	12.6	4.69	2.89	7.581	J
SSA_1520	9.05	5.53	2.764	3.52	5.03	7.29	3.02	8.54	2.76	3.02	5.025	6.78	0	0	2.76	2.51	8.79	9.55	7.54	6.533	J
SSA_2061	9.05	4.76	3.333	4.76	7.14	2.38	4.29	8.1	3.81	3.33	5.714	8.1	0	0.48	2.86	2.38	6.67	7.62	8.1	7.143	J
SSA_0120	9.09	6.06	6.061	3.03	10.6	3.79	1.52	10.6	3.03	0.76	3.03	9.09	0.76	0	2.27	2.27	9.09	8.33	4.55	6.061	J
SSA_0804	9.11	4.44	4.222	5.11	4.67	5.11	3.11	6	2.89	1.56	3.111	9.78	0	0	3.56	2	10.9	9.33	6.22	8.889	G
SSA_0943	9.18	3.06	2.721	10.2	3.4	5.78	1.02	10.2	2.38	2.72	3.401	12.9	1.7	0.34	3.4	4.76	8.5	3.4	1.36	9.524	P
SSA_0788	9.19	4.7	2.35	5.34	5.34	5.98	2.14	6.41	3.85	1.5	4.701	9.19	0.21	0	2.35	4.06	9.4	8.33	5.98	8.974	C
SSA_0548	9.21	4.07	2.439	6.78	4.61	5.15	1.36	6.78	3.25	3.25	4.065	10.8	1.36	0.81	2.71	2.71	7.86	6.23	5.96	10.57	M
SSA_0789	9.29	9.29	3.571	2.14	4.29	4.29	2.14	14.3	4.29	4.29	1.429	5.71	0.71	0	0.71	0.71	4.29	7.86	10	10.71	C
SSA_0302	9.3	2.26	4.271	4.02	8.79	4.27	1.26	6.28	1.76	0.75	3.518	9.3	0.75	0	1.51	3.77	11.6	8.04	7.04	11.56	G
SSA_1495	9.34	5.3	2.02	5.56	5.05	6.06	1.26	7.32	4.55	2.27	4.545	6.57	0.25	0	3.54	2.78	8.08	6.31	8.33	10.86	H

SSA_0656	9.41	6.12	4.235	5.18	3.53	6.82	2.12	6.82	3.76	0.47	4.235	6.35	0.47	0	0.47	3.06	10.8	9.18	5.88	11.06	D
SSA_0575	9.47	6.51	5.325	2.37	10.7	4.14	2.96	8.88	3.55	1.78	3.55	7.69	2.37	0	2.96	2.96	4.73	5.92	7.1	7.101	R
SSA_0914	9.49	4.12	4.12	6.74	5.24	5.62	1.75	6.12	3.12	1.12	3.87	10.2	0.5	0.5	2.62	3.37	6.62	8.99	5.62	10.24	J
SSA_1851	9.52	8.1	2.381	2.86	4.76	5.71	2.86	7.14	4.76	0.95	4.286	7.14	0	0	4.76	4.76	7.14	11	6.67	5.238	F
SSA_1620	9.54	4.98	4.149	4.15	5.81	5.81	4.56	8.3	4.15	1.24	2.49	7.47	0.41	0	2.07	1.66	9.96	6.22	7.05	9.959	F
SSA_0870	9.57	7.83	6.087	4.35	7.83	3.91	3.04	8.26	2.61	1.3	3.478	8.7	0.43	0	3.48	2.17	7.39	7.39	5.65	6.522	D
SSA_1938	9.57	3.7	4.321	3.7	6.79	4.63	3.09	8.33	3.09	1.85	2.778	5.86	0.62	0	1.23	3.09	11.1	7.1	5.25	13.89	R
SSA_2203	9.62	5.38	3.462	2.69	7.69	5	3.46	7.69	4.62	1.92	3.077	5.77	1.15	0	1.92	2.69	5.38	9.62	7.69	11.15	J
SSA_1748	9.65	2.57	5.466	7.07	4.18	4.5	2.25	6.43	2.57	2.25	3.215	9	0	0	2.89	4.18	5.79	9.65	5.14	13.18	C
SSA_1870	9.79	1.83	2.446	4.59	4.28	5.2	3.06	10.4	3.98	3.36	1.835	13.5	1.53	0	2.75	9.79	11	2.45	2.75	5.505	M
SSA_1936	9.84	3.28	5.328	6.15	5.74	5.33	3.28	8.2	3.69	0.41	2.049	9.02	0	0.41	1.23	3.69	9.84	5.74	4.51	12.3	IQR
SSA_1105	9.84	0.82	3.279	2.46	9.84	3.28	0.82	8.2	0	0	1.639	9.02	0	0	0	1.64	8.2	14.8	4.1	22.13	J
SSA_1934	9.88	1.23	3.704	6.79	6.79	3.09	3.7	5.56	4.94	0	9.877	6.17	0	0	1.23	3.09	5.56	9.88	4.94	13.58	I
SSA_1623	9.93	2.84	3.546	3.55	9.93	8.51	4.96	6.38	3.55	0	7.092	4.26	0	0	0.71	3.55	7.8	6.38	2.84	14.18	J
SSA_0119	10	5.56	4.444	5.56	10	5.56	2.78	5	1.11	0.56	3.889	10.6	0	0	2.22	4.44	7.78	7.78	5.56	7.222	J
SSA_0569	10	3	6	4	6	7	3	5	3	1	5	7	1	0	2	4	5	13	6	9	J
SSA_2034	10.1	4.05	3.378	4.73	10.8	8.11	3.38	4.73	2.7	3.38	4.73	8.11	0.68	0	3.38	2.03	8.78	4.73	4.05	8.108	J
SSA_2208	10.2	3.39	1.695	5.08	10.2	8.47	3.39	10.2	1.69	0	1.695	6.78	3.39	0	3.39	15.3	1.69	3.39	6.78	3.39	U
SSA_0226	10.2	4.07	5.185	5.93	5.93	6.67	2.96	7.96	2.04	0.19	3.148	7.96	0.19	0	0.93	1.67	8.89	9.07	5.56	11.48	O
SSA_1784	10.2	5.68	3.788	7.2	4.92	5.3	2.27	7.2	3.41	1.89	5.682	10.6	1.14	1.52	3.03	2.27	5.68	7.58	3.79	6.818	M
SSA_0859	10.2	2.36	3.937	5.51	7.09	3.15	1.97	4.33	3.15	1.18	3.543	6.69	0.79	1.57	2.76	3.94	7.48	7.09	6.69	16.54	G
SSA_1622	10.5	3.93	5.24	3.06	10.5	7.86	2.62	4.8	2.62	0	2.62	6.55	0.44	0	1.75	3.49	7.86	5.24	6.99	13.97	J
SSA_1642	10.7	3.49	6.972	4.79	5.66	7.84	1.74	8.06	3.7	3.05	2.832	6.1	0	0	2.83	2.83	9.37	6.75	5.23	8.061	M
SSA_1937	10.8	4.25	2.288	5.56	5.56	4.58	2.61	4.9	3.92	0.65	3.595	10.8	0	0.33	2.94	3.59	7.19	9.8	4.9	11.76	I
SSA_1939	10.8	0	1.351	5.41	5.41	6.76	1.35	8.11	4.05	0	1.351	9.46	0	0	1.35	5.41	4.05	20.3	9.46	5.405	IQ
SSA_2111	10.9	9.49	5.109	7.3	12.4	6.57	1.46	3.65	3.65	2.92	6.569	7.3	0	0	2.92	0.73	8.76	2.19	2.19	5.839	J
SSA_1265	11	12.6	2.362	3.15	8.66	6.3	1.57	11	3.15	1.57	3.937	5.51	0	0	3.15	2.36	8.66	6.3	2.36	6.299	J
SSA_0129	11.1	8.33	4.167	2.78	6.94	8.33	5.56	8.33	1.39	1.39	2.778	5.56	0	0	4.17	2.78	6.94	6.94	6.94	5.556	J
SSA_0109	11.2	3.06	5.102	2.04	13.3	8.16	2.04	6.12	2.04	1.02	2.041	5.1	0	0	4.08	4.08	6.12	9.18	3.06	12.24	J
SSA_2072	11.2	3	2.996	4.87	4.12	3.37	2.62	6.74	1.87	1.5	4.12	16.5	0.37	0	3	9.36	10.1	2.25	3.37	8.614	I
SSA_2108	11.3	3.88	4.776	3.58	6.57	8.06	2.39	5.37	2.69	1.49	2.985	6.87	0.6	0.6	2.39	3.28	10.1	5.97	6.87	10.15	G
SSA_1104	11.4	4.82	4.217	6.02	7.83	1.81	1.81	6.02	1.2	0	2.41	10.8	0	0	0.6	1.81	4.82	9.64	5.42	19.28	J
SSA_0117	11.5	9.02	2.459	4.92	9.84	5.74	1.64	9.02	1.64	0	3.279	4.92	0	0	0.82	3.28	11.5	4.92	5.74	9.836	J
SSA_0108	11.6	6.76	4.348	8.21	7.73	5.31	0.48	4.83	3.38	0.48	3.865	8.21	0.97	0	1.45	3.86	7.25	5.8	3.38	12.08	J
SSA_0123	11.9	10.2	3.39	5.08	10.2	5.93	0.85	4.24	2.54	1.69	1.695	6.78	0	0	2.54	2.54	9.32	4.24	5.93	11.02	J
SSA_1933	12.1	2.86	3.571	2.86	9.29	4.29	4.29	5.71	3.57	1.43	5	9.29	0	0	2.14	5	7.14	7.86	4.29	9.286	I
SSA_0122	12.4	6.74	3.933	5.62	10.1	6.74	2.25	5.62	1.69	1.69	5.056	6.18	0	0	2.25	2.25	11.2	10.1	2.25	3.933	J
SSA_2140	12.5	6.82	3.409	2.27	12.5	1.14	2.27	6.82	9.09	5.68	0	10.2	0	1.14	2.27	3.41	4.55	9.09	2.27	4.545	J
SSA_0107	13	5.77	5.288	3.37	10.1	6.25	2.4	6.25	3.37	1.92	4.808	4.81	0	0	0.96	3.37	12	5.29	3.37	7.692	J
SSA_1223	14.2	5.74	4.738	4.74	7.23	5.49	0.75	3.99	2.74	1.25	2.494	10.5	0.75	0.5	0.25	3.24	7.73	9.98	6.23	7.481	J
SSA_0124	15.2	7.32	3.659	4.27	7.32	4.27	1.22	4.88	1.22	0.61	3.049	8.54	0	0	0	2.44	11	8.54	3.05	13.41	J
SSA_0116	16.3	10.5	6.977	3.49	11.6	5.81	3.49	6.98	0	2.33	2.326	3.49	0	0	3.49	1.16	3.49	9.3	4.65	4.651	J
SSA_0225	18.3	2.15	4.301	2.15	8.6	3.23	1.08	3.23	3.23	1.08	1.075	10.8	0	0	1.08	1.08	11.8	12.9	3.23	10.75	O

Supplementary Table 6. Amino acid composition of non-essential proteins in *S. sanguinis*.

amino acids	V	R	N	S	K	T	M	I	Q	H	P	L	W	C	Y	F	G	E	D	A	COG
SSA_0898	0	0	3.225806	12.90323	3.225806	3.225806	3.225806	3.225806	9.677419	6.451613	6.451613	19.35484	0	0	3.225806	6.451613	0	6.451613	6.451613	6.451613	
SSA_1600	0	6.25	0	0	3.125	6.25	9.375	9.375	0	3.125	3.125	9.375	9.375	0	6.25	9.375	3.125	6.25	9.375	6.25	
SSA_2127	0	0	3.125	6.25	6.25	6.25	3.125	6.25	6.25	3.125	0	6.25	3.125	3.125	3.125	15.625	9.375	9.375	3.125	6.25	
SSA_1412	0	6.060606	3.030303	9.090909	9.090909	3.030303	3.030303	6.060606	6.060606	0	3.030303	9.090909	6.060606	0	0	9.090909	6.060606	9.090909	6.060606	6.060606	
SSA_1709	0	0	9.090909	15.15152	6.060606	6.060606	3.030303	6.060606	3.030303	3.030303	0	21.21212	0	3.030303	6.060606	6.060606	0	0	12.12121	0	
SSA_1733	0	0	2.941176	8.823529	14.70588	2.941176	2.941176	5.882353	5.882353	2.941176	2.941176	11.76471	0	2.941176	2.941176	20.58824	2.941176	2.941176	0	5.882353	
SSA_1089	0	0	5.714286	2.857143	14.28571	11.42857	2.857143	17.14286	2.857143	0	0	11.42857	0	2.857143	2.857143	11.42857	2.857143	5.714286	2.857143	2.857143	
SSA_1700	0	0	13.51351	0	16.21622	2.702703	2.702703	21.62162	0	0	0	21.62162	0	0	0	13.51351	2.702703	2.702703	2.702703	0	
SSA_0011	0	2.380952	4.761905	4.761905	14.28571	2.380952	2.380952	2.380952	9.52381	2.380952	0	16.66667	0	0	0	4.761905	2.380952	19.04762	4.761905	7.142857	
SSA_2162	0	6.382979	4.255319	10.6383	17.02128	2.12766	4.255319	14.89362	12.76596	2.12766	2.12766	6.382979	0	0	4.255319	4.255319	4.255319	2.12766	2.12766	0	
SSA_2233	0	12.76596	2.12766	8.510638	8.510638	4.255319	4.255319	6.382979	6.382979	0	0	6.382979	0	2.12766	6.382979	2.12766	4.255319	14.89362	2.12766	8.510638	
SSA_1915	0	3.703704	3.703704	5.555556	1.851852	5.555556	11.11111	11.11111	7.407407	1.851852	3.703704	14.81481	1.851852	0	3.703704	9.259259	7.407407	0	0	7.407407	
SSA_0887	3.030303	3.030303	3.030303	12.12121	12.12121	6.060606	6.060606	3.030303	6.060606	6.060606	0	6.060606	0	3.030303	6.060606	15.15152	3.030303	3.030303	3.030303	0	
SSA_0666	2.857143	2.857143	0	17.14286	11.42857	0	2.857143	5.714286	2.857143	5.714286	0	2.857143	0	0	14.28571	11.42857	5.714286	11.42857	2.857143	0	
SSA_0573	2.631579	7.894737	2.631579	10.52632	15.78947	5.263158	2.631579	10.52632	2.631579	0	0	15.78947	0	0	7.894737	0	5.263158	7.894737	2.631579	0	
SSA_0228	2.325581	2.325581	4.651163	4.651163	16.27907	4.651163	2.325581	4.651163	4.651163	4.651163	4.651163	11.62791	0	4.651163	2.325581	9.302326	2.325581	9.302326	0	4.651163	
SSA_1665	2.325581	2.325581	4.651163	0	0	4.651163	2.325581	2.325581	9.302326	2.325581	6.976744	30.23256	0	0	4.651163	9.302326	4.651163	4.651163	0	9.302326	
SSA_2335	2.325581	0	2.325581	2.325581	6.976744	4.651163	2.325581	4.651163	9.302326	2.325581	0	18.60465	0	0	11.62791	16.27907	11.62791	2.325581	0	2.325581	
SSA_2388	1.960784	7.843137	5.882353	5.882353	11.76471	1.960784	3.921569	11.76471	7.843137	0	1.960784	7.843137	0	1.960784	5.882353	3.921569	5.882353	7.843137	3.921569	1.960784	
SSA_2386	1.785714	0	3.571429	3.571429	8.928571	3.571429	1.785714	14.28571	3.571429	0	1.785714	12.5	1.785714	3.571429	7.142857	12.5	10.71429	1.785714	0	7.142857	S
SSA_2229	1.754386	7.017544	1.754386	7.017544	21.05263	1.754386	3.508772	1.754386	10.52632	3.508772	0	10.52632	0	0	1.754386	3.508772	7.017544	8.77193	3.508772	5.263158	
SSA_0142	1.694915	0	5.084746	22.0339	5.084746	11.86441	3.389831	5.084746	3.389831	0	1.694915	8.474576	0	3.389831	0	3.389831	10.16949	1.694915	0	13.55932	
SSA_1499	1.515152	15.15152	0	4.545455	18.18182	7.575758	4.545455	1.515152	3.030303	7.575758	1.515152	6.060606	0	0	1.515152	6.060606	10.60606	0	1.515152	9.090909	J
SSA_2103	1.515152	3.030303	3.030303	9.090909	7.575758	3.030303	3.030303	9.090909	9.090909	1.515152	3.030303	18.18182	0	0	3.030303	3.030303	3.030303	1.515152	9.090909	9.090909	
SSA_1617	1.408451	4.225352	4.225352	7.042254	7.042254	4.225352	2.816901	2.816901	2.816901	4.225352	4.225352	7.042254	2.816901	0	2.816901	11.26761	1.408451	11.26761	8.450704	9.859155	S
SSA_0313	1.315789	7.894737	3.947368	3.947368	7.894737	6.578947	1.315789	7.894737	2.631579	1.315789	2.631579	10.52632	0	0	6.578947	5.263158	2.631579	21.05263	1.315789	5.263158	S
SSA_1032	1.282051	6.410256	7.692308	10.25641	16.66667	2.564103	2.564103	6.410256	1.282051	2.564103	1.282051	6.410256	0	0	0	2.564103	1.282051	7.692308	2.564103	20.51282	J
SSA_1661	1.234568	7.407407	3.703704	9.876543	14.81481	2.469136	3.703704	11.11111	7.407407	2.469136	3.703704	6.17284	1.234568	1.234568	6.17284	3.703704	3.703704	3.703704	0	6.17284	
SSA_0699	1.219512	3.658537	7.317073	3.658537	9.756098	8.536585	4.878049	9.756098	3.658537	1.219512	1.219512	6.097561	1.219512	0	4.878049	4.878049	8.536585	10.97561	4.878049	3.658537	
SSA_1116	0.943396	0	0.943396	20.75472	20.75472	3.773585	16.03774	2.830189	0.943396	0	0	2.830189	0	0.943396	0	0.943396	0	2.830189	21.69811	3.773585	
SSA_0080	0.917431	4.587156	0.917431	10.09174	7.33945	2.752294	1.834862	7.33945	7.33945	1.834862	2.752294	16.51376	0.917431	0.917431	3.669725	5.504587	2.752294	9.174312	4.587156	8.256881	
SSA_2336	0.892857	3.571429	2.678571	10.71429	8.035714	8.928571	1.785714	10.71429	4.464286	2.678571	1.785714	11.60714	0.892857	0.892857	8.035714	1.785714	4.464286	8.928571	3.571429	3.571429	K
SSA_1031	0.578035	8.67052	5.780347	6.936416	8.67052	3.468208	3.468208	5.780347	5.780347	1.156069	4.046243	8.092486	4.624277	2.312139	5.202312	6.358382	4.046243	5.202312	5.202312	4.624277	F
SSA_0920	6.451613	0	12.90323	12.90323	9.677419	3.225806	3.225806	6.451613	0	0	0	9.677419	0	0	6.451613	9.677419	9.677419	6.451613	3.225806	0	
SSA_1672	6.25	0	0	3.125	9.375	3.125	3.125	9.375	0	0	3.125	28.125	3.125	3.125	3.125	9.375	0	3.125	6.25	6.25	
SSA_1191	6.060606	3.030303	6.060606	12.12121	21.21212	0	3.030303	0	0	0	0	6.060606	6.060606	0	6.060606	12.12121	0	9.090909	6.060606	3.030303	
SSA_2081	6.060606	3.030303	3.030303	9.090909	12.12121	0	3.030303	3.030303	6.060606	3.030303	9.090909	6.060606	3.030303	0	0	6.060606	6.060606	0	6.060606	15.15152	
SSA_0294	5.882353	0	2.941176	14.70588	8.823529	5.882353	5.882353	2.941176	2.941176	2.941176	2.941176	11.76471	0	2.941176	5.882353	2.941176	2.941176	11.76471	0	5.882353	E
SSA_1912	5.714286	2.857143	5.714286	14.28571	8.571429	0	2.857143	8.571429	2.857143	0	0	8.571429	2.857143	0	2.857143	17.14286	0	2.857143	2.857143	11.42857	
SSA_2054	5.714286	2.857143	0	8.571429	8.571429	8.571429	5.714286	5.714286	2.857143	0	0	5.714286	0	5.714286	2.857143	11.42857	8.571429	8.571429	5.714286	2.857143	
SSA_2394	5.714286	5.714286	8.571429	8.571429	2.857143	2.857143	2.857143	8.571429	5.714286	0	5.714286	8.571429	8.571429	0	2.857143	8.571429	8.571429	0	2.857143	2.857143	
SSA_0769	5.555556	2.777778	0	8.333333	0	8.333333	2.777778	11.11111	8.333333	8.333333	2.777778	2.777778	2.777778	0	5.555556	2.777778	5.555556	13.88889	5.555556	2.777778	
SSA_0697	5.405405	8.108108	0	5.405405	5.405405	2.702703	2.702703	16.21622	0	0	0	18.91892	0	0	2.702703	13.51351	2.702703	13.51351	0	2.702703	
SSA_1708	4.878049	2.439024	2.439024	12.19512	12.19512	4.878049	2.439024	4.878049	0	2.439024	0	19.5122	2.439024	0	4.878049	7.317073	4.878049	0	2.439024	9.756098	
SSA_1856	4.651163	6.976744	9.302326	11.62791	2.325581	4.651163	2.325581	6.976744	2.325581	0	2.325581	9.302326	0	2.325581	0	6.976744	2.325581	13.95349	6.976744	4.651163	
SSA_1552	4.444444	2.222222	2.222222	4.444444	24.44444	4.444444	6.666667	0	0	0	4.444444	4.444444	0	0	0	8.888889	0	17.77778	11.11111	4.444444	
SSA_1475	4.166667	2.083333	4.166667	6.25	10.41667	0	4.166667	8.333333	2.083333	2.083333	0	12.5	0	2.083333	2.083333	8.333333	6.25	12.5	4.166667	8.333333	
SSA_2344	4.081633	2.040816	4.081633	8.163265	4.081633	6.122449	2.040816	10.20408	8.163265	0	4.081633	8.163265	0	0	12.2449	4.081633	4.081633	6.122449	6.122449	6.122449	
SSA_0808	3.773585	7.54717	9.433962	3.773585	11.32075	0	1.886792	1.886792	0	0	3.773585	11.32075	1.886792	0	0	5.660377	3.773585	16.98113	5.660377	11.32075	
SSA_0369	3.508772	1.754386	7.017544	5.263158	7.017544	7.017544	5.263158	10.52632	1.754386	0	0	8.77193	0	1.754386	5.263158	3.508772	8.77193	5.263158	3.508772	14.03509	E
SSA_1877	3.448276	6.896552	1.724138	3.448276	10.34483	1.724138	3.448276	17.24138	1.724138	0	1.724138	12.06897	3.448276	1.724138	3.448276	8.62069	1.724138	3.448276	3.448276	10.34483	
SSA_0839	3.333333	3.333333	3.333333	1.666667	16.66667	1.666667	3.333333	10	0	1.666667	1.666667	18.33333	0	0	1.666667	5	10	6.666667	5	6.666667	
SSA_0732	3.278689	3.278689	6.557377	3.278689	11.47541	8.196721	4.918033	16.39344	1.639344	1.639344	1.639344	14.7541	0	0	0	3.278689	3.278689	4.918033	8.196721	3.278689	
SSA_1667	3.278689	3.278689	4.918033	14.7541	8.196721	9.836066	1.639344	4.918033	8.196721	1.639344	1.639344	8.196721	0	1.639344	0	3.278689	3.278689	6.557377	8.196721	6.557377	
SSA_1892	3.125	3.125	1.5625	3.125	17.1875	1.5625	4.6875	14.0625	0	0	4.6875	14.0625	0	0	1.5625	9.375	7.8125	4.6875	1.5625	7.8125	
SSA_2393	2.985075	10.44776	4.477612	7.462687	7.462687	2.985075	1.492537	7.462687	4.477612	1.492537	0	14.92537	0	0	1.492537	5.970149	1.492537	17.91045	1.492537	5.970149	K
SSA_1691	2.898551	1.449275	5.797101	2.898551	10.14493	5.797101	2.898551	13.04348	4.347826	0	1.449275	13.04348	0	2.898551	0	2.898551	5.797101	5.797101	8.695652	10.14493	K
SSA_1874	2.816901	5.633803	11.26761	5.633803	8.450704	5.633803	4.225352	5.633803	4.225352	0	2.816901	12.67606	1.408451	1.408451	1.408451	2.816901	1.408451	9.859155	7.042254	5.633803	K
SSA_0646	2.777778	6.944444	0	1.388889	12.5	2.777778	6.944444	8.333333	2.777778	1.388889	0	13.88889	0	0	8.333333	4.166667	4.166667	12.5	5.555556	5.555556	S
SSA_2131	2.777778	6.944444	6.944444	4.166667	5.555556	2.777778	2.777778	9.722222	4.166667	0	4.166667	15.27778	0	2.777778	1.388889	1.388889	2.777778	8.333333	8.333333	9.722222	K

SSA_1675	2.739726	5.479452	5.479452	5.479452	9.589041	10.9589	2.739726	12.32877	2.739726	2.739726	4.109589	9.589041	0	4.109589	0	2.739726	2.739726	6.849315	4.109589	5.479452	
SSA_2030	2.564103	3.846154	7.692308	10.25641	8.974359	2.564103	2.564103	7.692308	5.128205	0	2.564103	15.38462	0	0	5.128205	2.564103	5.128205	6.410256	6.410256	5.128205	
SSA_0282	1.869159	1.869159	1.869159	3.738318	7.476636	5.607477	3.738318	11.21495	7.476636	3.738318	0	13.08411	0	0.934579	0.934579	2.803738	5.607477	10.28037	7.476636	10.28037	G
SSA_1146	1.818182	2.727273	4.545455	9.090909	4.545455	2.727273	4.545455	7.272727	5.454545	4.545455	0	13.63636	0.909091	0	0.909091	2.727273	3.636364	11.81818	4.545455	14.54545	G
SSA_1294	1.801802	2.702703	4.504505	8.108108	6.306306	8.108108	0.900901	9.90991	7.207207	3.603604	0.900901	10.81081	0.900901	0	3.603604	3.603604	4.504505	8.108108	10.81081	3.603604	
SSA_0554	1.73913	6.956522	2.608696	6.086957	8.695652	3.478261	1.73913	2.608696	10.43478	3.478261	0	12.17391	2.608696	0.869565	5.217391	2.608696	2.608696	11.30435	8.695652	6.086957	
SSA_1320	1.652893	5.785124	2.479339	1.652893	5.785124	2.479339	2.479339	4.132231	6.61157	5.785124	6.61157	13.22314	2.479339	3.305785	7.438017	0.826446	5.785124	10.7438	4.132231	6.61157	
SSA_0937	1.503759	8.270677	3.759398	9.774436	3.759398	6.015038	3.007519	10.52632	4.511278	2.255639	5.263158	13.53383	0.75188	1.503759	0.75188	3.759398	2.255639	8.270677	6.015038	4.511278	P
SSA_1052	1.449275	0.724638	5.797101	18.11594	9.42029	20.28986	1.449275	7.971014	2.898551	0.724638	1.449275	2.173913	0	0	0.724638	0.724638	2.173913	5.072464	5.072464	13.76812	
SSA_1311	1.092896	2.73224	4.371585	6.557377	4.918033	6.010929	2.73224	8.196721	6.557377	2.185792	3.278689	10.92896	1.092896	0	5.464481	4.371585	8.196721	5.464481	6.010929	9.836066	R
SSA_0927	0.985222	6.403941	0.985222	5.91133	5.418719	3.940887	3.448276	2.955665	5.418719	3.448276	1.970443	15.27094	1.477833	1.477833	5.91133	4.926108	4.926108	9.359606	6.403941	9.359606	K
SSA_0609	0.97561	3.414634	4.390244	6.829268	3.414634	6.341463	2.926829	7.317073	10.73171	1.95122	3.414634	14.14634	0	0	3.902439	6.341463	3.902439	8.292683	3.414634	8.292683	K
SSA_2112	10	0	6.666667	0	16.66667	10	6.666667	13.33333	10	0	0	3.333333	0	3.333333	6.666667	0	0	3.333333	3.333333	6.666667	
SSA_0229	9.375	0	12.5	3.125	15.625	0	3.125	6.25	12.5	3.125	0	3.125	0	0	0	6.25	9.375	9.375	3.125	3.125	
SSA_1229	8.571429	5.714286	2.857143	5.714286	11.42857	0	5.714286	2.857143	2.857143	5.714286	5.714286	8.571429	0	0	5.714286	8.571429	5.714286	8.571429	2.857143	2.857143	
SSA_0643	7.5	2.5	5	5	20	2.5	5	12.5	2.5	0	0	12.5	0	0	2.5	10	2.5	0	7.5	2.5	
SSA_1926	7.317073	7.317073	4.878049	0	12.19512	4.878049	2.439024	9.756098	9.756098	2.439024	0	7.317073	0	0	4.878049	12.19512	2.439024	7.317073	0	4.878049	
SSA_1644	6.25	4.166667	4.166667	10.41667	10.41667	6.25	2.083333	4.166667	4.166667	2.083333	2.083333	12.5	0	2.083333	0	6.25	2.083333	6.25	8.333333	6.25	
SSA_2064	6	2	2	6	12	6	4	6	4	0	4	16	0	2	2	4	2	4	10	8	
SSA_1367	5.882353	5.882353	0	3.921569	5.882353	0	1.960784	9.803922	9.803922	7.843137	3.921569	5.882353	1.960784	1.960784	0	9.803922	3.921569	3.921569	9.803922	7.843137	
SSA_2024	5.454545	0	9.090909	3.636364	3.636364	5.454545	1.818182	5.454545	3.636364	3.636364	3.636364	5.454545	1.818182	0	9.090909	1.818182	3.636364	14.54545	1.818182	16.36364	
SSA_1673	5.357143	1.785714	7.142857	5.357143	10.71429	3.571429	3.571429	7.142857	5.357143	0	5.357143	12.5	0	1.785714	1.785714	8.928571	7.142857	5.357143	0	7.142857	
SSA_2071	5.357143	12.5	3.571429	7.142857	7.142857	1.785714	3.571429	0	5.357143	1.785714	1.785714	12.5	1.785714	1.785714	1.785714	12.5	3.571429	3.571429	5.357143	7.142857	
SSA_0889	4.761905	0	7.936508	1.587302	17.46032	0	4.761905	6.349206	3.174603	0	1.587302	7.936508	0	0	1.587302	6.349206	7.936508	9.52381	11.11111	7.936508	
SSA_2390	4.6875	7.8125	3.125	1.5625	14.0625	6.25	7.8125	10.9375	0	6.25	1.5625	1.5625	1.5625	4.6875	1.5625	3.125	4.6875	7.8125	4.6875	6.25	S
SSA_0729	4.545455	0	4.545455	10.60606	4.545455	9.090909	3.030303	4.545455	6.060606	1.515152	3.030303	16.66667	0	0	1.515152	4.545455	10.60606	3.030303	0	12.12121	
SSA_2047	4.166667	8.333333	1.388889	5.555556	11.11111	5.555556	4.166667	4.166667	2.777778	1.388889	5.555556	12.5	1.388889	0	1.388889	1.388889	4.166667	11.11111	8.333333	5.555556	S
SSA_2055	4.054054	9.459459	0	8.108108	25.67568	1.351351	2.702703	1.351351	14.86486	2.702703	1.351351	8.108108	0	0	0	2.702703	1.351351	9.459459	1.351351	5.405405	
SSA_1630	3.846154	2.564103	6.410256	1.282051	7.692308	6.410256	1.282051	10.25641	8.974359	3.846154	5.128205	3.846154	3.846154	5.128205	7.692308	3.846154	7.692308	6.410256	1.282051	2.564103	
SSA_1822	3.571429	4.761905	3.571429	3.571429	7.142857	1.190476	3.571429	2.380952	8.333333	1.190476	1.190476	9.52381	1.190476	0	7.142857	5.952381	5.952381	11.90476	7.142857	10.71429	
SSA_0293	3.529412	2.352941	5.882353	2.352941	10.58824	4.705882	2.352941	9.411765	7.058824	2.352941	3.529412	11.76471	0	2.352941	4.705882	5.882353	3.529412	2.352941	5.882353	9.411765	E
SSA_0709	3.448276	5.747126	2.298851	3.448276	12.64368	8.045977	3.448276	1.149425	5.747126	2.298851	3.448276	9.195402	0	1.149425	8.045977	1.149425	5.747126	6.896552	2.298851	13.7931	L
SSA_0978	3.409091	5.681818	2.272727	2.272727	9.090909	3.409091	1.136364	10.22727	0	3.409091	1.136364	18.18182	7.954545	2.272727	5.681818	3.409091	10.22727	1.136364	1.136364	7.954545	S
SSA_0618	3.092784	3.092784	4.123711	6.185567	6.185567	6.185567	4.123711	3.092784	12.37113	5.154639	3.092784	8.247423	0	1.030928	8.247423	8.247423	1.030928	5.154639	8.247423	3.092784	
SSA_1755	3.092784	3.092784	10.30928	7.216495	6.185567	8.247423	4.123711	5.154639	7.216495	0	3.092784	13.40206	1.030928	0	1.030928	2.061856	3.092784	6.185567	2.061856	13.40206	
SSA_1450	2.970297	4.950495	3.960396	5.940594	3.960396	1.980198	3.960396	3.960396	6.930693	1.980198	1.980198	12.87129	0	0.990099	4.950495	5.940594	3.960396	12.87129	7.920792	7.920792	
SSA_0246	2.803738	5.607477	6.542056	7.476636	9.345794	4.672897	7.476636	9.345794	1.869159	3.738318	1.869159	7.476636	0	0	1.869159	1.869159	8.411215	8.411215	7.476636	3.738318	
SSA_0954	2.777778	10.18519	6.481481	6.481481	11.11111	1.851852	2.777778	7.407407	4.62963	1.851852	1.851852	5.555556	1.851852	0.925926	5.555556	2.777778	0	17.59259	6.481481	1.851852	
SSA_1166	2.654867	4.424779	4.424779	5.309735	6.19469	3.539823	4.424779	9.734513	6.19469	0.884956	0.884956	8.849558	0	0	9.734513	4.424779	2.654867	10.61947	8.849558	6.19469	S
SSA_1754	2.564103	11.11111	3.418803	7.692308	9.401709	2.564103	4.273504	3.418803	5.128205	0.854701	0	10.25641	0	0	5.982906	4.273504	0	14.52991	8.547009	5.982906	
SSA_0306	2.479339	12.39669	4.958678	6.61157	7.438017	3.305785	4.958678	7.438017	4.132231	2.479339	3.305785	7.438017	0	0	4.958678	3.305785	4.132231	7.438017	5.785124	7.438017	K
SSA_2275	2.479339	6.61157	2.479339	9.917355	12.39669	3.305785	0.826446	4.132231	6.61157	3.305785	0.826446	18.18182	0.826446	0	0.826446	0.826446	3.305785	14.87603	4.132231	4.132231	
SSA_0620	2.419355	4.032258	1.612903	7.258065	6.451613	5.645161	2.419355	4.83871	11.29032	4.032258	2.419355	12.09677	0.806452	1.612903	3.225806	4.83871	5.645161	3.225806	8.064516	8.064516	
SSA_1570	2.272727	0.757576	4.545455	5.30303	9.848485	6.818182	3.030303	6.060606	3.030303	2.272727	3.787879	9.848485	0.757576	0.757576	8.333333	6.060606	5.30303	3.787879	12.87879	4.545455	
SSA_1628	2.158273	5.755396	5.035971	4.316547	4.316547	5.035971	1.438849	7.913669	7.913669	3.597122	4.316547	12.23022	2.158273	1.438849	2.877698	2.877698	7.913669	9.352518	1.438849	7.913669	LR
SSA_0139	2.040816	6.122449	2.721088	6.802721	7.482993	4.761905	2.721088	8.163265	6.802721	4.081633	2.721088	8.843537	2.721088	3.401361	2.040816	3.401361	4.081633	8.843537	4.761905	7.482993	K
SSA_0662	1.986755	3.97351	6.622517	7.94702	9.933775	3.97351	3.97351	5.960265	10.59603	0	1.986755	15.23179	0	1.324503	4.635762	3.97351	4.635762	4.635762	3.311258	5.298013	K
SSA_1292	1.886792	3.144654	1.886792	8.176101	6.289308	7.54717	0.628931	5.031447	8.805031	1.886792	4.402516	14.46541	0	0.628931	1.886792	3.144654	5.660377	7.54717	6.289308	10.69182	
SSA_1288	1.875	1.25	6.25	8.75	9.375	1.875	1.875	8.75	3.125	0	1.25	10.625	2.5	1.25	6.25	5	5	12.5	6.25	6.25	
SSA_1286	1.863354	1.242236	8.695652	6.21118	6.21118	3.726708	1.863354	9.937888	3.726708	1.242236	1.242236	13.04348	1.242236	1.863354	4.347826	5.590062	3.726708	9.937888	6.21118	8.074534	
SSA_1590	1.612903	5.376344	4.301075	8.064516	8.064516	4.83871	1.612903	11.82796	4.83871	2.150538	1.612903	10.75269	0.537634	0.537634	5.913978	4.301075	2.688172	9.677419	4.83871	6.451613	K
SSA_1801	1.363636	5.909091	5.454545	7.272727	10	5.454545	2.272727	5	6.818182	1.363636	7.272727	8.181818	0.454545	0	4.090909	3.181818	1.818182	7.727273	8.636364	7.727273	
SSA_1275	12.5	6.25	6.25	0	6.25	0	3.125	6.25	3.125	3.125	0	6.25	0	3.125	3.125	15.625	3.125	6.25	6.25	9.375	
SSA_0806	9.52381	2.380952	2.380952	4.761905	7.142857	7.142857	7.142857	11.90476	2.380952	0	0	9.52381	0	0	0	9.52381	9.52381	2.380952	2.380952	11.90476	
SSA_1164	9.302326	0	9.302326	16.27907	6.976744	2.325581	2.325581	2.325581	2.325581	2.325581	4.651163	16.27907	0	2.325581	4.651163	6.976744	0	0	6.976744	4.651163	
SSA_1472	7.142857	5.357143	5.357143	7.142857	10.71429	1.785714	1.785714	10.71429	0	3.571429	1.785714	16.07143	0	0	3.571429	1.785714	7.142857	3.571429	10.71429	1.785714	
SSA_0820	6.896552	17.24138	1.724138	6.896552	20.68966	6.896552	1.724138	0	1.724138	0	1.724138	5.172414	0	0	1.724138	5.172414	1.724138	8.62069	3.448276	8.62069	
SSA_0121	6.779661	1.694915	1.694915	5.084746	10.16949	3.389831	1.694915	5.084746	1.694915	0	1.694915	18.64407	0	1.694915	3.389831	3.389831	3.389831	11.86441	6.779661	11.86441	
SSA_0149	6.779661	5.084746	1.694915	5.084746	3.389831	3.389831	1.694915	5.084746	1.694915	3.389831	3.389831	25.42373	6.779661	0	8.474576	6.779661	6.779661	0	3.389831	1.694915	
SSA_2287	6.666667	11.66667	1.666667	6.666667	15	11.66667	1.666667	1.666667	0	3.333333	3.333333	1.666667	0	0	6.666667	1.666667	5	3.333333	3.333333	15	J
SSA_1133	6.451613	1.612903	4.83871	3.225806	12.90323	1.612903	3.225806	9.677419	1.612903	1.612903	4.83871	11.29032	0	0	0	4.83871	12.90323	12.90323	1.612903	4.83871	U
SSA_0171	6.349206	6.349206	9.52381	3.174603	9.52381	6.349206	6.349206	7.936508	6.349206	1.587302	0	14.28571	0	0	0	1.587302	3.174603	4.761905	4.761905	7.936508	K
SSA_1503	5.970149	2.985075	2.985075	1.492537	7.462687	4.477612	2.985075	7.462687	4.477612	1.492537	4.477612	10.44776	0	5.970149	4.477612	2.985075	4.477612	7.462687	11.9403	5.970149	C

SSA_1537	5.714286	2.857143	7.142857	7.142857	10	5.714286	2.857143	4.285714	1.428571	1.428571	2.857143	8.571429	2.857143	0	1.428571	7.142857	5.714286	11.42857	5.714286	5.714286	S
SSA_0734	5.479452	0	4.109589	6.849315	12.32877	4.109589	1.369863	5.479452	5.479452	2.739726	2.739726	12.32877	1.369863	0	4.109589	5.479452	8.219178	9.589041	1.369863	6.849315	K
SSA_0745	5.479452	1.369863	1.369863	8.219178	5.479452	2.739726	5.479452	6.849315	2.739726	0	2.739726	27.39726	2.739726	4.109589	2.739726	6.849315	6.849315	0	1.369863	5.479452	
SSA_2053	5.479452	1.369863	1.369863	2.739726	9.589041	6.849315	4.109589	4.109589	9.589041	1.369863	1.369863	8.219178	0	1.369863	9.589041	8.219178	5.479452	6.849315	8.219178	4.109589	
SSA_2296	5.405405	8.108108	6.756757	5.405405	8.108108	4.054054	6.756757	6.756757	5.405405	1.351351	1.351351	10.81081	0	0	1.351351	2.702703	2.702703	13.51351	2.702703	6.756757	K
SSA_1224	5.263158	1.315789	3.947368	7.894737	15.78947	3.947368	1.315789	11.84211	3.947368	0	0	6.578947	0	0	3.947368	3.947368	7.894737	9.210526	6.578947	6.578947	
SSA_2062	5.263158	5.263158	0	6.578947	1.315789	2.631579	5.263158	14.47368	1.315789	1.315789	2.631579	11.84211	3.947368	1.315789	0	7.894737	17.10526	1.315789	2.631579	7.894737	S
SSA_1254	5	7.5	2.5	7.5	5	3.75	6.25	7.5	2.5	5	0	7.5	0	1.25	6.25	5	6.25	11.25	6.25	3.75	
SSA_2094	4.705882	2.352941	5.882353	4.705882	10.58824	12.94118	4.705882	10.58824	3.529412	2.352941	1.176471	8.235294	1.176471	0	5.882353	0	4.705882	4.705882	3.529412	8.235294	
SSA_1657	4.494382	2.247191	6.741573	3.370787	11.23596	2.247191	3.370787	10.11236	3.370787	2.247191	2.247191	16.85393	5.617978	1.123596	2.247191	3.370787	2.247191	5.617978	2.247191	8.988764	
SSA_2283	4.210526	2.105263	6.315789	5.263158	4.210526	9.473684	1.052632	9.473684	9.473684	1.052632	2.105263	8.421053	1.052632	0	1.052632	4.210526	2.105263	9.473684	7.368421	11.57895	S
SSA_0224	4.166667	4.166667	3.125	3.125	4.166667	3.125	3.125	11.45833	4.166667	3.125	0	18.75	1.041667	0	5.208333	6.25	8.333333	7.291667	2.083333	7.291667	
SSA_0353	4.166667	5.208333	5.208333	5.208333	11.45833	6.25	4.166667	3.125	2.083333	2.083333	1.041667	12.5	0	0	2.083333	5.208333	2.083333	13.54167	7.291667	7.291667	
SSA_0952	4.081633	5.102041	1.020408	9.183673	7.142857	5.102041	2.040816	8.163265	5.102041	0	5.102041	10.20408	1.020408	0	1.020408	2.040816	4.081633	10.20408	4.081633	15.30612	
SSA_1638	3.921569	0.980392	2.941176	1.960784	6.862745	9.803922	2.941176	11.76471	1.960784	5.882353	0.980392	10.78431	6.862745	2.941176	5.882353	5.882353	9.803922	0.980392	2.941176	3.921569	S
SSA_0973	3.883495	7.76699	3.883495	1.941748	9.708738	3.883495	1.941748	3.883495	7.76699	1.941748	1.941748	10.67961	0	0.970874	7.76699	3.883495	0	12.62136	9.708738	5.825243	
SSA_2239	3.883495	0.970874	6.796117	2.912621	1.941748	5.825243	2.912621	6.796117	2.912621	4.854369	2.912621	7.76699	0.970874	0	1.941748	4.854369	5.825243	22.3301	9.708738	3.883495	S
SSA_0269	3.809524	1.904762	0.952381	4.761905	9.52381	3.809524	5.714286	4.761905	7.619048	4.761905	0	9.52381	0	0.952381	0.952381	4.761905	3.809524	10.47619	6.666667	15.2381	G
SSA_0988	3.809524	3.809524	3.809524	4.761905	7.619048	0	3.809524	4.761905	4.761905	0.952381	7.619048	14.28571	0	0.952381	7.619048	5.714286	6.666667	7.619048	3.809524	7.619048	K
SSA_0884	3.571429	1.785714	6.25	4.464286	8.928571	6.25	0.892857	7.142857	6.25	0.892857	1.785714	10.71429	1.785714	1.785714	5.357143	8.035714	3.571429	8.928571	7.142857	4.464286	
SSA_1441	3.508772	2.631579	0.877193	5.263158	7.894737	8.77193	2.631579	5.263158	6.140351	4.385965	1.754386	10.52632	3.508772	2.631579	3.508772	2.631579	7.017544	6.140351	7.894737	7.017544	E
SSA_0183	3.174603	3.968254	3.174603	4.761905	9.52381	3.174603	3.174603	3.174603	6.349206	1.587302	4.761905	8.730159	4.761905	3.968254	8.730159	3.174603	3.174603	8.730159	8.730159	3.174603	S
SSA_0750	3.125	1.5625	3.125	7.03125	13.28125	7.03125	4.6875	12.5	2.34375	0.78125	2.34375	10.9375	0	0.78125	2.34375	2.34375	7.03125	12.5	3.125	3.125	
SSA_2029	3.053435	4.580153	5.343511	5.343511	9.160305	4.580153	3.816794	5.343511	6.10687	2.290076	2.290076	11.45038	1.526718	0.763359	5.343511	6.870229	3.053435	9.923664	3.816794	5.343511	L
SSA_0705	3.030303	3.787879	5.30303	1.515152	4.545455	3.030303	3.030303	10.60606	4.545455	0.757576	2.272727	18.93939	4.545455	0.757576	5.30303	5.30303	9.090909	4.545455	1.515152	7.575758	
SSA_1088	2.941176	5.147059	4.411765	5.147059	9.558824	5.147059	2.205882	2.941176	5.147059	0.735294	2.205882	10.29412	0.735294	0.735294	10.29412	3.676471	6.617647	11.76471	4.411765	5.882353	E
SSA_1640	2.857143	7.142857	4.285714	8.571429	4.285714	6.428571	1.428571	8.571429	10	0.714286	3.571429	8.571429	2.142857	0	2.142857	4.285714	6.428571	8.571429	5	5	
SSA_1145	2.758621	4.137931	5.517241	6.896552	13.7931	8.275862	1.37931	7.586207	6.206897	2.068966	0.689655	10.34483	0.689655	0.689655	6.206897	2.758621	3.448276	10.34483	4.827586	1.37931	
SSA_1573	2.5	3.125	5	6.875	4.375	3.75	2.5	6.875	5	1.25	3.125	18.75	3.125	0	8.125	7.5	6.875	2.5	4.375	4.375	
SSA_0004	2.453988	0.613497	4.294479	6.134969	15.33742	8.588957	0.613497	6.134969	4.907975	0.613497	1.226994	11.04294	0	0.613497	6.134969	4.907975	4.294479	8.588957	6.134969	7.361963	S
SSA_2067	2.222222	1.111111	4.444444	9.444444	16.66667	5.555556	2.222222	7.222222	7.777778	1.111111	2.777778	10	0	1.111111	2.777778	4.444444	1.666667	9.444444	6.111111	3.888889	
SSA_1305	2.173913	7.608696	4.891304	6.521739	2.717391	1.630435	1.630435	7.065217	3.804348	2.717391	2.717391	7.065217	0.543478	0.543478	5.978261	11.95652	3.26087	13.04348	6.521739	7.608696	
SSA_0627	2.072539	11.9171	7.772021	9.84456	10.88083	1.554404	0.518135	4.145078	4.145078	1.554404	3.108808	5.181347	0.518135	1.036269	4.663212	6.217617	5.181347	9.84456	7.253886	2.590674	S
SSA_2135	1.95122	3.902439	6.341463	9.756098	8.780488	2.926829	2.926829	11.70732	6.829268	0.97561	2.926829	12.68293	0.487805	0.97561	4.390244	5.853659	2.439024	7.317073	5.365854	1.463415	K
SSA_2392	13.15789	13.15789	5.263158	2.631579	13.15789	0	5.263158	7.894737	5.263158	2.631579	10.52632	0	0	7.894737	2.631579	0	5.263158	2.631579	0	2.631579	J
SSA_0723	10.86957	2.173913	6.521739	8.695652	13.04348	4.347826	2.173913	6.521739	4.347826	2.173913	0	15.21739	4.347826	0	2.173913	4.347826	4.347826	0	4.347826	4.347826	
SSA_1317	10.41667	4.166667	2.083333	6.25	6.25	2.083333	2.083333	18.75	0	2.083333	4.166667	8.333333	0	2.083333	6.25	8.333333	2.083333	6.25	2.083333	6.25	
SSA_1760	9.615385	5.769231	1.923077	5.769231	7.692308	1.923077	9.615385	7.692308	3.846154	1.923077	0	13.46154	0	3.846154	5.769231	5.769231	5.769231	3.846154	3.846154	1.923077	
SSA_1422	8.77193	3.508772	7.017544	7.017544	7.017544	1.754386	7.017544	7.017544	5.263158	3.508772	3.508772	10.52632	1.754386	1.754386	1.754386	3.508772	1.754386	12.2807	3.508772	1.754386	
SSA_0792	8.333333	1.666667	6.666667	6.666667	5	3.333333	3.333333	11.66667	3.333333	0	1.666667	16.66667	1.666667	0	3.333333	6.666667	13.33333	5	0	1.666667	
SSA_0909	7.936508	4.761905	3.174603	1.587302	6.349206	0	4.761905	9.52381	9.52381	0	4.761905	6.349206	0	0	4.761905	4.761905	7.936508	9.52381	7.936508	6.349206	K
SSA_0115	7.352941	7.352941	2.941176	2.941176	16.17647	2.941176	1.470588	2.941176	8.823529	0	0	13.23529	0	0	0	4.411765	2.941176	16.17647	1.470588	8.823529	J
SSA_0445	7.246377	4.347826	2.898551	7.246377	10.14493	0	1.449275	11.5942	1.449275	2.898551	5.797101	14.49275	1.449275	2.898551	4.347826	10.14493	2.898551	1.449275	2.898551	4.347826	
SSA_0675	7.142857	0	2.857143	4.285714	14.28571	4.285714	5.714286	2.857143	5.714286	0	0	11.42857	0	0	0	2.857143	4.285714	18.57143	5.714286	10	L
SSA_0761	7.142857	10	7.142857	4.285714	4.285714	4.285714	2.857143	8.571429	4.285714	1.428571	1.428571	12.85714	0	0	1.428571	2.857143	4.285714	12.85714	1.428571	8.571429	K
SSA_1964	7.042254	5.633803	5.633803	8.450704	12.67606	4.225352	2.816901	8.450704	1.408451	1.408451	0	4.225352	1.408451	0	2.816901	5.633803	4.225352	9.859155	5.633803	8.450704	
SSA_0841	6.849315	2.739726	1.369863	9.589041	4.109589	8.219178	4.109589	12.32877	5.479452	1.369863	1.369863	21.91781	1.369863	0	2.739726	4.109589	2.739726	4.109589	0	5.479452	
SSA_1215	6.849315	2.739726	1.369863	6.849315	4.109589	9.589041	2.739726	2.739726	5.479452	1.369863	2.739726	16.43836	0	1.369863	9.589041	5.479452	5.479452	5.479452	8.219178	1.369863	
SSA_2032	6.849315	6.849315	4.109589	8.219178	10.9589	8.219178	1.369863	4.109589	2.739726	5.479452	4.109589	8.219178	0	0	1.369863	2.739726	4.109589	5.479452	4.109589	10.9589	L
SSA_1456	6.410256	1.282051	6.410256	7.692308	7.692308	7.692308	1.282051	10.25641	2.564103	1.282051	5.128205	6.410256	0	0	3.846154	6.410256	3.846154	10.25641	7.692308	3.846154	
SSA_2279	6.329114	11.39241	3.797468	0	7.594937	3.797468	1.265823	17.72152	2.531646	2.531646	2.531646	8.860759	0	0	2.531646	2.531646	6.329114	10.12658	8.860759	1.265823	S
SSA_1272	6.25	8.75	2.5	8.75	7.5	8.75	2.5	3.75	3.75	2.5	5	3.75	0	0	6.25	7.5	7.5	7.5	6.25	1.25	J
SSA_0027	6.097561	0	3.658537	6.097561	9.756098	2.439024	3.658537	13.41463	2.439024	4.878049	2.439024	8.536585	0	0	2.439024	3.658537	1.219512	15.85366	9.756098	3.658537	IQ
SSA_1878	5.882353	9.411765	2.352941	3.529412	12.94118	3.529412	1.176471	7.058824	3.529412	3.529412	3.529412	7.058824	0	0	2.352941	0	7.058824	15.29412	7.058824	4.705882	S
SSA_0599	5.813953	1.162791	5.813953	4.651163	5.813953	4.651163	9.302326	9.302326	2.325581	1.162791	3.488372	11.62791	2.325581	1.162791	3.488372	3.488372	6.976744	1.162791	3.488372	12.7907	
SSA_0730	5.617978	3.370787	4.494382	3.370787	10.11236	4.494382	1.123596	7.865169	3.370787	2.247191	2.247191	11.23596	1.123596	0	5.617978	2.247191	6.741573	11.23596	3.370787	10.11236	K
SSA_2355	5.494505	4.395604	4.395604	7.692308	9.89011	5.494505	3.296703	8.791209	4.395604	1.098901	2.197802	5.494505	0	0	6.593407	3.296703	6.593407	12.08791	5.494505	3.296703	S
SSA_2058	5.102041	12.2449	7.142857	3.061224	12.2449	3.061224	3.061224	8.163265	2.040816	5.102041	0	9.183673	1.020408	0	5.102041	0	5.102041	8.163265	4.081633	6.122449	J
SSA_1387	5.050505	1.010101	5.050505	6.060606	9.090909	1.010101	4.040404	9.090909	5.050505	0	5.050505	9.090909	1.010101	2.020202	8.080808	4.040404	7.070707	8.080808	7.070707	3.030303	S
SSA_1543	5	4	2	11	5	7	3	5	7	1	4	12	0	1	8	10	0	7	3	5	
SSA_0478	4.950495	0	1.980198	6.930693	10.89109	3.960396	0.990099	10.89109	4.950495	1.980198	4.950495	5.940594	0.990099	0	4.950495	5.940594	8.910891	7.920792	2.970297	9.90099	P
SSA_0562	4.854369	1.941748	3.883495	2.912621	5.825243	4.854369	4.854369	7.76699	2.912621	1.941748	2.912621	7.76699	0.970874	0.970874	5.825243	4.854369	6.796117	18.4466	8.737864	0.970874	
SSA_0972	4.854369	2.912621	3.883495	2.912621	9.708738	4.854369	4.854369	5.825243	7.76699	0.970874	2.912621	12.62136	0	0	1.941748	1.941748	4.854369	7.76699	5.825243	13.59223	

SSA_1278	4.807692	5.769231	3.846154	4.807692	8.653846	3.846154	2.884615	6.730769	6.730769	1.923077	2.884615	9.615385	0	1.923077	3.846154	2.884615	7.692308	8.653846	4.807692	7.692308	P
SSA_1694	4.761905	2.857143	1.904762	3.809524	6.666667	5.714286	4.761905	4.761905	1.904762	5.714286	0	13.33333	0	0.952381	2.857143	1.904762	5.714286	10.47619	5.714286	16.19048	G
SSA_2013	4.672897	8.411215	0.934579	5.607477	8.411215	7.476636	3.738318	7.476636	4.672897	0.934579	1.869159	12.14953	0	0.934579	5.607477	2.803738	7.476636	13.08411	1.869159	1.869159	K
SSA_1477	4.464286	5.357143	3.571429	1.785714	14.28571	6.25	3.571429	8.928571	3.571429	1.785714	4.464286	8.928571	0	4.464286	4.464286	3.571429	3.571429	5.357143	5.357143	6.25	L
SSA_1102	4.424779	3.539823	4.424779	8.849558	7.964602	6.19469	1.769912	7.079646	0.884956	0	7.079646	11.50442	3.539823	0.884956	2.654867	13.27434	4.424779	2.654867	5.309735	3.539823	
SSA_0190	4.385965	5.263158	1.754386	11.40351	13.15789	4.385965	2.631579	1.754386	4.385965	0.877193	0	7.894737	0	0	3.508772	5.263158	4.385965	11.40351	6.140351	11.40351	
SSA_0296	4.310345	4.310345	6.034483	8.62069	12.06897	3.448276	2.586207	5.172414	6.034483	1.724138	2.586207	15.51724	0	0	3.448276	4.310345	1.724138	9.482759	5.172414	3.448276	K
SSA_1379	4.310345	7.758621	3.448276	5.172414	6.896552	6.034483	3.448276	5.172414	6.034483	1.724138	4.310345	8.62069	0.862069	0.862069	3.448276	4.310345	4.310345	4.310345	13.7931	5.172414	
SSA_1540	4.273504	7.692308	2.564103	5.982906	4.273504	4.273504	1.709402	5.128205	5.128205	5.128205	4.273504	6.837607	0	9.401709	6.837607	5.128205	4.273504	5.982906	6.837607	4.273504	S
SSA_1712	4.273504	5.128205	4.273504	5.128205	7.692308	5.128205	1.709402	5.128205	6.837607	0.854701	3.418803	15.38462	0.854701	1.709402	3.418803	4.273504	5.128205	6.837607	7.692308	5.128205	P
SSA_1538	4.237288	11.01695	5.084746	5.084746	11.86441	4.237288	3.389831	13.55932	5.932203	0.847458	1.694915	7.627119	0	0	1.694915	4.237288	4.237288	6.779661	5.084746	3.389831	
SSA_1304	4.166667	7.5	1.666667	4.166667	5.833333	2.5	3.333333	3.333333	3.333333	5.833333	5.833333	11.66667	2.5	2.5	9.166667	0.833333	5.833333	10	3.333333	6.666667	
SSA_0327	4	1.6	7.2	7.2	10.4	4.8	3.2	8.8	4	0	3.2	7.2	0.8	0.8	6.4	4	8	8	6.4	4	
SSA_0816	4	8	3.2	9.6	8	6.4	2.4	4	3.2	1.6	4	13.6	1.6	0	1.6	4.8	6.4	9.6	4	4	K
SSA_1625	3.90625	7.03125	2.34375	0	6.25	5.46875	1.5625	7.03125	1.5625	7.03125	6.25	10.9375	0	0	2.34375	7.8125	4.6875	13.28125	7.8125	4.6875	E
SSA_2372	3.787879	5.30303	4.545455	5.30303	15.15152	6.060606	1.515152	8.333333	6.060606	0.757576	5.30303	9.090909	0	0	2.272727	3.030303	6.060606	9.848485	3.030303	4.545455	S
SSA_0248	3.731343	9.701493	2.985075	4.477612	8.208955	3.731343	2.238806	4.477612	6.716418	2.985075	0	7.462687	0	1.492537	7.462687	2.985075	2.985075	15.67164	9.701493	2.985075	
SSA_2247	3.676471	5.882353	2.941176	6.617647	9.558824	2.205882	2.205882	13.97059	4.411765	2.205882	0.735294	12.5	2.205882	0	3.676471	5.882353	2.941176	8.088235	4.411765	5.882353	K
SSA_0933	3.401361	6.122449	2.040816	6.122449	7.482993	4.761905	1.360544	4.081633	4.761905	2.721088	1.360544	14.28571	0	0.680272	4.761905	4.761905	7.482993	8.163265	2.721088	12.92517	R
SSA_1385	3.378378	6.081081	3.378378	4.054054	10.81081	7.432432	2.702703	11.48649	4.72973	2.027027	2.027027	12.16216	0.675676	0.675676	0.675676	3.378378	3.378378	6.081081	8.783784	6.081081	K
SSA_0239	3.333333	4	0.666667	2.666667	4.666667	4.666667	4.666667	7.333333	6	0.666667	4	11.33333	2	2	2	5.333333	7.333333	13.33333	8.666667	5.333333	F
SSA_0958	3.246753	0	11.68831	7.792208	9.090909	7.142857	5.194805	1.948052	12.33766	0.649351	6.493506	2.597403	1.298701	0	0.649351	0	14.28571	3.246753	5.194805	7.142857	
SSA_1414	3.205128	6.410256	5.128205	3.205128	8.333333	5.128205	1.282051	7.051282	3.205128	2.564103	3.205128	8.974359	3.205128	1.923077	3.846154	5.128205	7.692308	12.82051	5.128205	2.564103	F
SSA_1523	3.184713	2.547771	5.732484	3.821656	9.55414	7.006369	1.273885	7.006369	7.643312	0.636943	5.095541	8.917197	1.273885	1.910828	3.821656	7.006369	8.280255	7.006369	5.095541	3.184713	O
SSA_1287	3.125	1.875	5	8.125	9.375	4.375	1.875	5.625	1.25	1.875	2.5	14.375	0.625	0.625	8.75	5	1.875	12.5	8.125	3.125	
SSA_1250	2.840909	6.818182	7.954545	6.818182	10.79545	4.545455	2.272727	5.681818	6.25	1.136364	2.840909	9.659091	0	0.568182	3.977273	5.681818	3.977273	6.25	6.818182	5.113636	L
SSA_1876	2.777778	3.888889	1.111111	1.666667	9.444444	1.666667	3.333333	12.77778	5	1.666667	3.333333	13.33333	1.666667	0.555556	2.777778	7.777778	8.333333	6.666667	3.888889	8.333333	
SSA_1493	2.73224	6.010929	3.825137	6.010929	6.557377	4.918033	3.278689	7.103825	3.825137	2.185792	2.185792	7.650273	1.639344	1.092896	4.918033	8.743169	4.918033	10.92896	3.825137	7.650273	J
SSA_0064	2.645503	6.349206	4.232804	3.703704	6.878307	3.174603	2.116402	5.291005	4.232804	2.645503	6.349206	11.11111	3.174603	1.587302	4.761905	3.703704	3.174603	10.05291	6.878307	7.936508	S
SSA_1211	2.645503	3.703704	6.349206	4.232804	7.936508	5.291005	2.116402	8.465608	5.820106	3.703704	3.174603	12.69841	0	0	2.116402	5.820106	3.703704	13.75661	4.761905	3.703704	S
SSA_0145	2.5	4.5	6.5	7.5	5	6	1	9	6	1	5.5	9.5	2.5	0.5	3	6	2	5	7.5	9.5	K
SSA_1831	1.937984	3.100775	3.100775	7.364341	13.17829	4.651163	1.162791	6.976744	8.527132	1.550388	0.775194	13.17829	0.387597	0	6.20155	3.875969	3.100775	8.914729	6.976744	5.03876	R
SSA_2251	1.77305	4.964539	4.964539	6.028369	10.6383	5.319149	1.06383	10.6383	5.673759	1.06383	1.41844	12.41135	1.06383	2.12766	6.382979	3.546099	5.319149	8.156028	4.609929	2.836879	K
SSA_0180	12.76596	6.382979	6.382979	8.510638	2.12766	4.255319	4.255319	12.76596	0	2.12766	0	10.6383	0	0	6.382979	14.89362	0	2.12766	2.12766	4.255319	
SSA_1478	11.76471	5.882353	1.960784	9.803922	7.843137	11.76471	3.921569	3.921569	0	0	1.960784	7.843137	1.960784	1.960784	1.960784	5.882353	1.960784	9.803922	3.921569	5.882353	
SSA_0639	10.52632	1.754386	1.754386	5.263158	12.2807	0	5.263158	7.017544	3.508772	0	1.754386	17.54386	0	5.263158	1.754386	10.52632	7.017544	0	1.754386	7.017544	
SSA_1150	10	6.666667	3.333333	1.666667	6.666667	6.666667	5	5	6.666667	1.666667	6.666667	5	0	0	1.666667	5	6.666667	10	3.333333	8.333333	R
SSA_0998	9.52381	7.936508	4.761905	3.174603	3.174603	3.174603	7.936508	7.936508	3.174603	1.587302	1.587302	7.936508	1.587302	0	4.761905	9.52381	4.761905	4.761905	3.174603	9.52381	
SSA_1372	9.52381	1.587302	1.587302	4.761905	11.11111	9.52381	3.174603	4.761905	4.761905	1.587302	3.174603	12.69841	1.587302	0	0	3.174603	1.587302	7.936508	4.761905	12.69841	
SSA_1473	9.52381	3.174603	1.587302	9.52381	6.349206	4.761905	3.174603	9.52381	7.936508	1.587302	6.349206	6.349206	0	0	1.587302	1.587302	4.761905	4.761905	6.349206	11.11111	V
SSA_0169	9.375	0	1.5625	0	14.0625	1.5625	1.5625	10.9375	7.8125	1.5625	6.25	10.9375	0	0	3.125	3.125	6.25	10.9375	9.375	1.5625	
SSA_0362	8.108108	1.351351	2.702703	2.702703	9.459459	5.405405	4.054054	6.756757	5.405405	4.054054	4.054054	10.81081	0	0	1.351351	2.702703	8.108108	8.108108	6.756757	8.108108	O
SSA_1866	8.108108	5.405405	0	4.054054	5.405405	5.405405	4.054054	13.51351	2.702703	1.351351	1.351351	18.91892	1.351351	0	4.054054	2.702703	6.756757	2.702703	4.054054	8.108108	
SSA_0648	8	0	2.666667	10.66667	8	4	5.333333	17.33333	4	1.333333	0	17.33333	0	0	1.333333	2.666667	8	4	1.333333	4	
SSA_1786	7.317073	7.317073	4.878049	4.878049	9.756098	1.219512	3.658537	7.317073	8.536585	1.219512	2.439024	12.19512	0	0	1.219512	3.658537	4.878049	7.317073	0	12.19512	S
SSA_0799	7.058824	0	5.882353	10.58824	11.76471	5.882353	3.529412	10.58824	1.176471	2.352941	1.176471	22.35294	0	0	1.176471	5.882353	2.352941	4.705882	0	3.529412	S
SSA_0772	6.896552	1.149425	1.149425	9.195402	8.045977	5.747126	4.597701	8.045977	2.298851	2.298851	1.149425	6.896552	0	0	1.149425	2.298851	9.195402	6.896552	6.896552	16.09195	G
SSA_1907	6.315789	2.105263	5.263158	5.263158	11.57895	3.157895	1.052632	12.63158	12.63158	0	0	7.368421	1.052632	0	4.210526	2.105263	1.052632	9.473684	6.315789	8.421053	
SSA_1147	6.25	0	2.083333	7.291667	9.375	2.083333	7.291667	10.41667	6.25	0	3.125	9.375	0	2.083333	6.25	1.041667	5.208333	2.083333	10.41667	9.375	G
SSA_0326	6.060606	0	3.030303	4.040404	11.11111	6.060606	8.080808	2.020202	10.10101	1.010101	3.030303	9.090909	0	0	0	4.040404	5.050505	6.060606	7.070707	14.14141	S
SSA_2272	6.060606	3.030303	4.040404	5.050505	8.080808	4.040404	3.030303	7.070707	6.060606	0	4.040404	11.11111	1.010101	0	7.070707	5.050505	7.070707	10.10101	6.060606	2.020202	S
SSA_0283	5.825243	0	2.912621	4.854369	6.796117	3.883495	4.854369	5.825243	1.941748	1.941748	2.912621	13.59223	0	2.912621	3.883495	1.941748	6.796117	4.854369	7.76699	16.50485	G
SSA_1453	5.825243	4.854369	2.912621	2.912621	12.62136	5.825243	2.912621	7.76699	5.825243	1.941748	3.883495	6.796117	0.970874	0	1.941748	3.883495	2.912621	15.53398	1.941748	8.737864	
SSA_1850	5.769231	8.653846	0.961538	5.769231	12.5	2.884615	1.923077	6.730769	1.923077	1.923077	4.807692	8.653846	0	0	0.961538	0.961538	3.846154	20.19231	2.884615	8.653846	K
SSA_1833	5.714286	4.761905	7.619048	8.571429	5.714286	4.761905	3.809524	9.52381	2.857143	0	3.809524	8.571429	0.952381	0	4.761905	10.47619	4.761905	4.761905	6.666667	1.904762	
SSA_0089	5.660377	4.716981	2.830189	2.830189	5.660377	8.490566	1.886792	10.37736	3.773585	1.886792	4.716981	11.32075	0	0	3.773585	3.773585	5.660377	7.54717	6.603774	8.490566	C
SSA_0345	5.555556	7.407407	4.62963	7.407407	9.259259	2.777778	1.851852	5.555556	9.259259	0.925926	0.925926	12.96296	3.703704	0	1.851852	1.851852	9.259259	8.333333	0.925926	5.555556	S
SSA_0459	5.405405	0.900901	2.702703	9.90991	4.504505	3.603604	1.801802	12.61261	0.900901	0	2.702703	19.81982	0.900901	0	1.801802	6.306306	14.41441	0	0.900901	10.81081	
SSA_1465	5.357143	2.678571	4.464286	6.25	7.142857	3.571429	1.785714	6.25	11.60714	0	2.678571	10.71429	0	0	3.571429	5.357143	2.678571	8.928571	7.142857	9.821429	S
SSA_2389	5.357143	4.464286	8.035714	5.357143	8.928571	3.571429	1.785714	11.60714	6.25	1.785714	0	10.71429	0	3.571429	3.571429	5.357143	1.785714	6.25	6.25	5.357143	K
SSA_2289	5.357143	4.464286	8.035714	5.357143	8.928571	4.464286	1.785714	10.71429	6.25	1.785714	0	11.60714	0	3.571429	3.571429	4.464286	1.785714	6.25	6.25	5.357143	K
SSA_1624	5.217391	3.478261	0.869565	4.347826	6.956522	6.956522	2.608696	9.565217	1.73913	0.869565	2.608696	19.13043	0.869565	0	6.956522	14.78261	2.608696	0.869565	1.73913	7.826087	
SSA_0553	5.172414	2.586207	7.758621	6.896552	5.172414	10.34483	3.448276	6.034483	6.896552	2.586207	2.586207	8.62069	0	0	1.724138	1.724138	5.172414	5.172414	6.034483	12.06897	

SSA_1266	5.172414	2.586207	3.448276	5.172414	3.448276	6.034483	3.448276	7.758621	0.862069	0.862069	0.862069	18.96552	1.724138	2.586207	5.172414	6.896552	10.34483	4.310345	1.724138	8.62069	D
SSA_1971	5.172414	4.310345	2.586207	6.896552	3.448276	2.586207	3.448276	16.37931	0	0.862069	4.310345	15.51724	3.448276	0	5.172414	14.65517	4.310345	1.724138	3.448276	1.724138	
SSA_1668	5.128205	3.418803	3.418803	3.418803	10.25641	7.692308	1.709402	10.25641	3.418803	2.564103	4.273504	5.982906	0.854701	3.418803	2.564103	4.273504	11.11111	5.982906	4.273504	5.982906	F
SSA_2206	5.084746	9.322034	7.627119	5.084746	19.49153	4.237288	0.847458	5.084746	5.932203	1.694915	0.847458	16.10169	0	0	0.847458	0.847458	2.542373	5.084746	5.084746	4.237288	
SSA_1393	5.042017	6.722689	7.563025	5.042017	10.92437	5.882353	2.521008	6.722689	9.243697	1.680672	1.680672	9.243697	2.521008	0	1.680672	0.840336	0	14.28571	6.722689	1.680672	
SSA_1378	5	1.666667	5	4.166667	5	1.666667	0.833333	4.166667	5	3.333333	5.833333	7.5	0.833333	1.666667	5	6.666667	9.166667	9.166667	5	13.33333	E
SSA_1683	4.878049	5.691057	4.878049	8.130081	9.756098	6.504065	0.813008	6.504065	5.691057	2.439024	3.252033	8.130081	1.626016	1.626016	3.252033	4.065041	8.943089	6.504065	4.878049	2.439024	F
SSA_0556	4.8	1.6	3.2	4	7.2	4	4	8.8	3.2	1.6	3.2	8.8	0.8	0.8	6.4	6.4	7.2	15.2	8	0.8	
SSA_2042	4.477612	5.970149	5.970149	4.477612	5.970149	7.462687	4.477612	7.462687	2.985075	3.731343	0.746269	8.955224	0.746269	0.746269	4.477612	2.985075	5.970149	11.19403	2.985075	8.208955	S
SSA_0557	4.379562	2.919708	6.569343	5.839416	10.94891	8.029197	2.189781	6.569343	4.379562	2.189781	2.189781	5.109489	0.729927	0	6.569343	3.649635	4.379562	7.29927	7.29927	8.759124	
SSA_0359	4.347826	2.898551	5.072464	7.246377	11.5942	1.449275	1.449275	14.49275	5.072464	2.898551	4.347826	6.521739	0.724638	2.173913	3.623188	5.797101	2.898551	6.521739	6.521739	4.347826	
SSA_0308	4.316547	2.877698	1.438849	4.316547	10.07194	3.597122	1.438849	7.194245	6.47482	3.597122	1.438849	11.51079	0	0.719424	5.755396	4.316547	5.755396	10.79137	5.035971	9.352518	R
SSA_0065	4.285714	5	2.142857	6.428571	7.857143	4.285714	3.571429	7.142857	5	2.142857	4.285714	5.714286	2.142857	2.142857	4.285714	5.714286	5	6.428571	10	6.428571	T
SSA_2384	4.225352	3.521127	4.929577	4.225352	11.97183	7.746479	1.408451	8.450704	2.112676	1.408451	1.408451	8.450704	1.408451	4.225352	2.816901	4.929577	5.633803	8.450704	7.042254	5.633803	R
SSA_0984	4.137931	4.827586	4.137931	5.517241	9.655172	6.896552	1.37931	4.827586	6.896552	2.068966	0.689655	13.10345	1.37931	1.37931	3.448276	3.448276	6.206897	8.965517	4.137931	6.896552	K
SSA_2252	4.137931	2.758621	4.827586	6.896552	3.448276	3.448276	1.37931	12.41379	3.448276	2.068966	2.758621	17.24138	2.758621	2.758621	2.068966	11.03448	6.206897	2.068966	1.37931	6.896552	
SSA_1890	4	8	2	8.666667	4.666667	5.333333	2	6	6.666667	1.333333	3.333333	12.66667	0.666667	0	6.666667	5.333333	7.333333	6.666667	5.333333	3.333333	R
SSA_0460	3.947368	5.263158	3.947368	5.921053	11.84211	1.973684	5.921053	8.552632	5.921053	1.315789	1.973684	10.52632	0	0.657895	1.315789	3.947368	6.578947	10.52632	5.263158	4.605263	K
SSA_1435	3.921569	5.228758	3.921569	5.228758	5.882353	4.575163	2.614379	4.575163	6.535948	1.960784	1.30719	11.11111	1.30719	2.614379	3.921569	8.496732	6.535948	7.843137	5.882353	6.535948	
SSA_1285	3.797468	3.164557	5.696203	5.063291	11.39241	3.164557	3.797468	6.962025	3.164557	0	1.898734	9.493671	0.632911	0	5.696203	9.493671	4.43038	9.493671	7.594937	5.063291	
SSA_0835	3.773585	5.031447	3.144654	10.06289	6.918239	5.031447	3.144654	8.176101	4.402516	2.515723	3.144654	8.176101	1.886792	1.257862	6.289308	5.031447	5.031447	8.176101	4.402516	4.402516	
SSA_0016	3.726708	8.695652	4.347826	3.10559	13.04348	3.10559	2.484472	4.968944	3.726708	3.726708	0.621118	11.80124	2.484472	0.621118	5.590062	4.347826	2.484472	10.55901	8.695652	1.863354	
SSA_1314	3.658537	4.268293	3.658537	0.609756	12.80488	4.268293	1.829268	6.097561	4.878049	3.658537	5.487805	10.36585	0	4.878049	2.439024	5.487805	2.439024	7.926829	7.926829	7.317073	R
SSA_1583	3.592814	4.191617	2.994012	5.389222	6.586826	5.389222	0.598802	8.383234	4.790419	1.197605	4.191617	11.97605	1.197605	0.598802	5.988024	2.39521	10.17964	7.784431	3.592814	8.982036	L
SSA_0358	3.550296	1.775148	5.91716	8.87574	7.100592	6.508876	2.95858	10.05917	4.733728	2.95858	2.95858	6.508876	1.775148	1.183432	4.733728	8.284024	4.142012	5.91716	8.284024	1.775148	
SSA_1798	3.508772	3.508772	2.923977	7.602339	5.847953	4.093567	2.923977	7.602339	3.508772	2.923977	2.339181	11.11111	1.169591	0.584795	4.678363	3.508772	7.017544	11.11111	4.678363	9.356725	R
SSA_1858	3.428571	2.285714	6.857143	2.857143	9.142857	5.142857	2.285714	5.714286	6.857143	0	1.142857	9.142857	1.714286	0	6.285714	8.571429	4.571429	9.714286	6.857143	7.428571	S
SSA_1875	3.409091	4.545455	1.136364	4.545455	7.954545	4.545455	2.272727	10.79545	4.545455	2.840909	2.840909	13.63636	0.568182	0	3.409091	10.22727	5.681818	6.25	4.545455	6.25	
SSA_0561	3.314917	4.41989	3.867403	8.287293	12.70718	3.314917	1.104972	8.839779	3.867403	2.762431	3.314917	11.04972	2.209945	0.552486	3.867403	3.314917	3.867403	7.18232	6.629834	5.524862	J
SSA_2227	3.314917	8.287293	2.209945	6.077348	6.077348	3.867403	3.314917	5.524862	3.867403	1.104972	2.762431	11.60221	2.209945	0.552486	2.762431	2.762431	7.734807	10.49724	7.734807	7.734807	R
SSA_1924	3.243243	3.783784	3.243243	4.324324	8.108108	4.864865	2.702703	6.486486	5.405405	0.540541	2.702703	14.59459	0.540541	1.081081	7.027027	5.405405	6.486486	10.27027	4.864865	4.324324	K
SSA_0170	3.125	4.166667	4.6875	4.6875	9.375	3.645833	3.645833	10.41667	5.729167	0.520833	2.604167	9.895833	0.520833	1.5625	6.770833	6.770833	3.645833	10.41667	5.208333	2.604167	
SSA_1479	2.870813	7.177033	7.177033	5.263158	11.00478	4.30622	2.392344	8.133971	4.30622	4.784689	2.392344	13.39713	0.478469	0.478469	3.827751	7.655502	1.435407	4.784689	3.827751	4.30622	L
SSA_2342	2.531646	5.485232	2.953586	6.329114	6.329114	5.485232	2.953586	6.751055	4.219409	3.797468	3.797468	11.81435	0.843882	1.265823	7.172996	4.219409	3.375527	8.860759	6.751055	5.063291	P
SSA_0922	2.489627	4.564315	4.149378	4.564315	9.958506	5.394191	2.489627	7.053942	4.979253	3.319502	4.149378	10.78838	2.904564	2.074689	1.659751	3.73444	5.394191	8.713693	2.904564	8.713693	K
SSA_0606	2.42915	5.263158	4.048583	6.072874	6.072874	2.834008	2.834008	8.502024	8.097166	2.42915	2.834008	14.5749	0	1.214575	1.619433	3.238866	6.882591	5.263158	6.477733	9.311741	V
SSA_1830	2.325581	2.713178	1.937984	5.426357	5.813953	4.263566	0.775194	4.651163	8.527132	3.875969	2.713178	13.17829	1.162791	1.162791	6.20155	5.813953	4.651163	6.976744	6.589147	11.24031	J
SSA_0206	12.96296	0	1.851852	0	9.259259	1.851852	1.851852	14.81481	3.703704	1.851852	3.703704	16.66667	3.703704	1.851852	5.555556	5.555556	3.703704	3.703704	0	7.407407	
SSA_2147	10.44776	0	2.985075	4.477612	22.38806	4.477612	2.985075	4.477612	1.492537	0	0	1.492537	0	0	0	1.492537	10.44776	14.92537	7.462687	10.44776	
SSA_0041	9.333333	0	4	6.666667	8	2.666667	1.333333	8	4	2.666667	5.333333	9.333333	0	0	6.666667	4	1.333333	10.66667	6.666667	9.333333	
SSA_0670	9.210526	7.894737	5.263158	5.263158	2.631579	3.947368	3.947368	6.578947	0	1.315789	0	9.210526	0	0	6.578947	3.947368	5.263158	13.15789	6.578947	9.210526	S
SSA_0790	9.210526	3.947368	3.947368	6.578947	3.947368	2.631579	3.947368	9.210526	0	2.631579	0	15.78947	1.315789	1.315789	2.631579	15.78947	3.947368	3.947368	2.631579	6.578947	
SSA_1309	8.860759	6.329114	1.265823	5.063291	7.594937	7.594937	1.265823	16.4557	2.531646	1.265823	5.063291	7.594937	0	0	3.797468	1.265823	3.797468	7.594937	8.860759	3.797468	R
SSA_0585	7.865169	12.35955	2.247191	6.741573	6.741573	2.247191	1.123596	3.370787	3.370787	2.247191	4.494382	7.865169	2.247191	2.247191	6.741573	6.741573	6.741573	2.247191	6.741573	5.617978	
SSA_1253	7.865169	6.741573	4.494382	4.494382	12.35955	3.370787	2.247191	7.865169	2.247191	1.123596	0	10.11236	0	2.247191	5.617978	6.741573	4.494382	6.741573	7.865169	3.370787	L
SSA_1730	7.865169	7.865169	7.865169	3.370787	7.865169	0	1.123596	8.988764	6.741573	4.494382	1.123596	10.11236	0	0	8.988764	2.247191	3.370787	10.11236	4.494382	3.370787	S
SSA_0892	7.777778	0	4.444444	6.666667	11.11111	4.444444	3.333333	7.777778	3.333333	0	3.333333	3.333333	0	1.111111	1.111111	3.333333	10	11.11111	8.888889	8.888889	
SSA_1394	7.692308	4.395604	5.494505	8.791209	6.593407	4.395604	3.296703	7.692308	10.98901	1.098901	1.098901	3.296703	1.098901	1.098901	3.296703	3.296703	3.296703	6.593407	5.494505	10.98901	
SSA_0938	7.608696	2.173913	5.434783	13.04348	11.95652	1.086957	1.086957	2.173913	5.434783	1.086957	2.173913	8.695652	1.086957	0	6.521739	4.347826	6.521739	10.86957	1.086957	7.608696	S
SSA_1549	7.142857	4.081633	3.061224	4.081633	9.183673	2.040816	5.102041	11.22449	0	0	2.040816	11.22449	2.040816	1.020408	4.081633	8.163265	7.142857	8.163265	7.142857	3.061224	KT
SSA_1897	7.142857	5.102041	3.061224	7.142857	11.22449	6.122449	3.061224	5.102041	4.081633	1.020408	1.020408	13.26531	0	0	2.040816	3.061224	6.122449	6.122449	3.061224	12.2449	J
SSA_0664	7	6	7	7	6	6	2	13	4	3	2	8	0	0	3	3	1	8	5	9	
SSA_1759	6.930693	3.960396	6.930693	5.940594	8.910891	1.980198	2.970297	5.940594	1.980198	1.980198	1.980198	7.920792	0.990099	0	5.940594	2.970297	8.910891	11.88119	7.920792	3.960396	
SSA_0577	6.796117	4.854369	5.825243	6.796117	11.65049	5.825243	0.970874	11.65049	8.737864	1.941748	0.970874	11.65049	0	0	0.970874	0.970874	3.883495	7.76699	3.883495	4.854369	J
SSA_1720	6.666667	6.666667	5.714286	1.904762	10.47619	5.714286	1.904762	0.952381	1.904762	1.904762	0.952381	13.33333	0	2.857143	2.857143	5.714286	2.857143	12.38095	10.47619	4.761905	S
SSA_0685	6.603774	1.886792	2.830189	4.716981	4.716981	2.830189	2.830189	16.03774	0.943396	0	1.886792	16.03774	2.830189	0	3.773585	7.54717	14.15094	0	3.773585	6.603774	
SSA_0083	6.542056	3.738318	2.803738	1.869159	6.542056	9.345794	2.803738	1.869159	13.08411	0.934579	0	13.08411	0	0	2.803738	0	1.869159	14.01869	6.542056	12.14953	
SSA_0211	6.306306	6.306306	4.504505	4.504505	6.306306	3.603604	0.900901	9.009009	3.603604	0	4.504505	8.108108	1.801802	1.801802	3.603604	6.306306	7.207207	9.90991	7.207207	4.504505	OC
SSA_2285	6.19469	6.19469	2.654867	6.19469	6.19469	5.309735	2.654867	12.38938	0.884956	0.884956	3.539823	7.964602	1.769912	2.654867	2.654867	1.769912	3.539823	8.849558	9.734513	7.964602	R
SSA_1214	6.086957	3.478261	9.565217	8.695652	8.695652	5.217391	1.73913	6.956522	3.478261	2.608696	2.608696	11.30435	0	0.869565	3.478261	7.826087	4.347826	6.086957	4.347826	2.608696	
SSA_1562	5.882353	4.201681	0.840336	5.882353	7.563025	5.882353	2.521008	14.28571	0.840336	0.840336	5.042017	15.12605	1.680672	0.840336	5.882353	7.563025	6.722689	3.361345	1.680672	3.361345	S
SSA_1840	5.833333	6.666667	2.5	5.833333	10.83333	2.5	1.666667	10.83333	4.166667	3.333333	4.166667	8.333333	0.833333	0.833333	2.5	4.166667	9.166667	10	4.166667	1.666667	J
SSA_0155	5.785124	10.7438	4.958678	5.785124	6.61157	3.305785	3.305785	7.438017	3.305785	0.826446	4.132231	9.090909	0	0	4.958678	9.917355	7.438017	4.958678	1.652893	5.785124	

SSA_0368	5.737705	4.918033	4.918033	4.918033	13.11475	4.098361	0.819672	5.737705	7.377049	3.278689	1.639344	9.836066	0.819672	0.819672	6.557377	5.737705	3.278689	7.377049	7.377049	1.639344	
SSA_2387	5.737705	4.918033	7.377049	4.918033	9.016393	3.278689	2.459016	11.47541	5.737705	1.639344	0	10.65574	0	4.098361	3.278689	4.918033	3.278689	6.557377	5.737705	4.918033	K
SSA_2385	5.645161	0.806452	1.612903	5.645161	7.258065	4.83871	6.451613	15.32258	3.225806	0.806452	3.225806	10.48387	1.612903	2.419355	3.225806	8.870968	7.258065	1.612903	0	9.677419	
SSA_0647	5.555556	7.142857	4.761905	4.761905	7.936508	1.587302	1.587302	7.936508	5.555556	1.587302	1.587302	13.49206	3.174603	0	3.968254	3.174603	8.730159	3.968254	5.555556	7.936508	P
SSA_1707	5.46875	5.46875	0.78125	6.25	3.90625	1.5625	1.5625	5.46875	3.90625	3.125	6.25	11.71875	0	1.5625	5.46875	9.375	5.46875	10.9375	7.03125	4.6875	E
SSA_0244	5.384615	6.153846	4.615385	5.384615	12.30769	3.076923	5.384615	8.461538	1.538462	3.846154	2.307692	10.76923	0	0	2.307692	3.076923	10	6.153846	7.692308	1.538462	
SSA_0854	5.30303	2.272727	3.787879	9.090909	6.060606	4.545455	2.272727	9.090909	5.30303	2.272727	3.030303	11.36364	3.030303	1.515152	1.515152	3.030303	4.545455	12.87879	5.30303	3.787879	LR
SSA_0360	5.185185	0	4.444444	5.925926	8.148148	5.185185	3.703704	5.925926	4.444444	2.222222	3.703704	8.888889	2.962963	1.481481	2.962963	8.148148	5.925926	10.37037	7.407407	2.962963	
SSA_1993	5.147059	2.941176	2.205882	3.676471	3.676471	5.882353	0.735294	6.617647	9.558824	3.676471	2.205882	5.147059	2.205882	1.470588	6.617647	5.147059	2.941176	10.29412	10.29412	9.558824	S
SSA_0595	5.109489	4.379562	3.649635	4.379562	4.379562	2.189781	2.919708	4.379562	4.379562	1.459854	4.379562	11.67883	2.189781	0	2.189781	7.29927	6.569343	9.489051	9.489051	9.489051	S
SSA_1655	4.964539	7.801418	2.12766	4.964539	4.255319	1.41844	1.41844	4.964539	6.382979	2.836879	0.70922	12.05674	0.70922	2.836879	4.964539	4.255319	8.510638	7.092199	5.673759	12.05674	R
SSA_1332	4.929577	3.521127	4.929577	5.633803	9.15493	4.929577	3.521127	7.042254	3.521127	2.816901	3.521127	5.633803	2.112676	2.112676	7.042254	4.225352	5.633803	10.56338	5.633803	3.521127	
SSA_1550	4.827586	11.72414	4.137931	4.827586	7.586207	2.758621	1.37931	2.758621	4.137931	4.137931	2.068966	11.03448	1.37931	2.758621	6.896552	6.206897	8.275862	5.517241	4.137931	3.448276	S
SSA_1911	4.794521	4.109589	4.109589	3.424658	6.164384	4.794521	1.369863	4.794521	4.109589	1.369863	2.739726	17.12329	1.369863	0	3.424658	3.424658	12.32877	8.90411	8.219178	3.424658	R
SSA_0665	4.697987	4.026846	4.697987	2.013423	6.040268	6.040268	1.342282	4.697987	5.369128	2.684564	4.697987	8.053691	2.684564	0	6.040268	5.369128	7.38255	12.75168	3.355705	8.053691	LR
SSA_2291	4.697987	10.73826	4.026846	10.06711	11.4094	2.684564	2.684564	6.040268	4.026846	2.684564	1.342282	7.38255	0.671141	0.671141	4.697987	0	1.342282	12.75168	3.355705	8.724832	
SSA_0315	4.545455	5.844156	1.298701	9.74026	9.090909	1.948052	0.649351	5.194805	4.545455	1.298701	3.896104	16.23377	0.649351	0.649351	1.948052	3.896104	6.493506	11.68831	2.597403	7.792208	K
SSA_0918	4.516129	1.935484	8.387097	10.96774	3.870968	10.96774	0.645161	4.516129	11.6129	1.290323	1.290323	6.451613	0.645161	0.645161	1.290323	1.290323	8.387097	3.225806	9.032258	9.032258	
SSA_0240	4.487179	5.128205	3.205128	3.205128	7.051282	7.051282	3.846154	7.692308	5.128205	2.564103	2.564103	5.769231	1.923077	0.641026	6.410256	4.487179	7.692308	10.89744	5.128205	5.128205	R
SSA_1382	4.458599	6.369427	3.821656	9.55414	7.006369	4.458599	3.821656	5.095541	3.821656	1.273885	3.184713	8.917197	2.547771	1.273885	6.369427	2.547771	5.095541	8.917197	5.095541	6.369427	
SSA_1334	4.43038	2.531646	10.75949	4.43038	6.962025	4.43038	2.531646	5.063291	3.164557	1.265823	4.43038	12.65823	1.265823	0	5.063291	6.962025	5.063291	8.860759	6.962025	3.164557	
SSA_0423	4.402516	5.660377	3.773585	11.32075	5.031447	7.54717	4.402516	6.918239	1.886792	1.257862	3.773585	13.20755	1.886792	0	3.773585	7.54717	5.031447	3.773585	1.257862	7.54717	
SSA_0099	4.347826	3.10559	3.726708	5.590062	8.695652	6.21118	3.726708	9.937888	2.484472	1.242236	4.347826	11.80124	1.242236	0.621118	4.347826	12.42236	3.10559	6.21118	4.347826	2.484472	
SSA_1183	4.347826	4.968944	3.10559	4.968944	6.21118	3.10559	4.347826	6.832298	4.347826	3.10559	2.484472	10.55901	1.242236	0	3.10559	4.968944	6.21118	11.18012	8.074534	6.832298	
SSA_1527	4.347826	3.10559	3.726708	6.832298	11.80124	5.590062	1.863354	10.55901	5.590062	2.484472	3.726708	9.31677	0	0	1.863354	1.863354	6.832298	6.21118	6.21118	8.074534	S
SSA_0102	4.117647	7.058824	1.764706	5.882353	7.058824	7.058824	1.764706	4.117647	3.529412	1.764706	1.764706	10.58824	1.764706	0.588235	4.705882	5.882353	7.058824	10	7.058824	6.470588	F
SSA_1059	4.046243	5.780347	2.890173	5.780347	4.624277	2.890173	2.890173	9.82659	2.890173	1.734104	3.468208	13.2948	1.156069	1.734104	6.936416	10.98266	4.624277	2.890173	2.890173	8.67052	O
SSA_0714	4	5.142857	1.142857	6.857143	1.714286	2.857143	4	9.714286	4.571429	3.428571	2.857143	20	2.285714	1.142857	2.285714	9.142857	3.428571	4.571429	1.714286	9.142857	
SSA_0265	3.932584	10.67416	4.494382	5.05618	11.79775	3.370787	2.247191	4.494382	4.494382	0	2.247191	13.48315	1.685393	0	6.179775	1.123596	5.05618	12.35955	4.494382	2.808989	L
SSA_1361	3.932584	10.67416	4.494382	5.05618	11.79775	3.370787	2.247191	4.494382	4.494382	0	2.247191	13.48315	1.685393	0	6.179775	1.123596	5.05618	12.35955	4.494382	2.808989	L
SSA_1996	3.867403	3.867403	6.077348	3.867403	4.41989	2.209945	1.657459	6.077348	0.552486	0.552486	3.867403	6.629834	1.104972	0	4.972376	2.209945	3.867403	19.33702	17.12707	7.734807	K
SSA_2256	3.825137	5.464481	1.639344	6.557377	8.743169	4.918033	3.278689	6.557377	4.371585	2.185792	3.278689	18.57923	2.185792	0	2.73224	7.103825	6.557377	1.092896	3.278689	7.650273	
SSA_1774	3.804348	5.434783	5.978261	3.804348	7.608696	3.26087	5.434783	6.521739	3.804348	3.26087	4.347826	9.23913	1.086957	1.086957	4.891304	5.978261	4.347826	7.608696	6.521739	5.978261	J
SSA_0992	3.626943	6.735751	2.590674	5.699482	7.253886	3.626943	3.108808	3.626943	3.108808	3.626943	3.626943	12.95337	2.590674	0	6.735751	4.145078	5.181347	11.39896	5.181347	5.181347	S
SSA_0213	3.589744	3.589744	3.076923	12.82051	3.589744	5.128205	1.538462	8.205128	3.589744	4.615385	4.102564	10.76923	1.538462	1.025641	6.153846	7.692308	2.564103	3.076923	5.128205	8.205128	
SSA_1011	3.571429	4.081633	4.081633	3.061224	6.122449	3.571429	3.571429	10.20408	2.55102	1.530612	2.55102	19.38776	4.081633	1.020408	3.571429	5.612245	9.183673	3.061224	2.040816	7.142857	
SSA_0590	3.553299	4.060914	0.507614	7.614213	6.091371	3.553299	3.045685	6.091371	2.538071	1.015228	3.045685	18.27411	1.015228	1.015228	4.060914	10.6599	10.15228	2.030457	2.030457	9.64467	
SSA_0048	3.535354	3.535354	4.040404	6.060606	7.070707	7.070707	1.515152	8.080808	5.555556	3.030303	3.030303	12.12121	0.505051	0.505051	2.525253	7.575758	6.060606	9.090909	4.040404	5.050505	K
SSA_0266	3.465347	4.455446	4.455446	9.90099	10.39604	3.465347	3.465347	5.940594	7.425743	1.980198	3.465347	7.920792	0.49505	0.990099	6.435644	5.445545	5.445545	6.435644	3.960396	4.455446	L
SSA_1362	3.465347	4.950495	4.455446	9.90099	9.405941	3.465347	3.465347	5.940594	7.425743	1.980198	3.465347	7.920792	0.49505	0.990099	6.435644	5.445545	5.445545	7.425743	3.960396	3.960396	L
SSA_1528	3.317536	4.739336	5.21327	6.161137	4.739336	8.530806	1.421801	7.109005	4.739336	4.265403	3.317536	10.42654	1.421801	0	3.317536	5.21327	5.687204	9.478673	4.265403	6.635071	G
SSA_0105	3.301887	8.490566	1.886792	8.490566	4.245283	4.716981	0.943396	5.188679	4.245283	2.830189	3.773585	15.09434	0.943396	1.886792	3.773585	3.301887	4.716981	7.075472	6.603774	8.490566	F
SSA_0349	3.301887	3.773585	2.830189	6.603774	8.018868	6.603774	5.660377	6.132075	8.018868	1.886792	1.886792	10.84906	1.415094	0	1.415094	5.188679	5.188679	8.018868	5.660377	7.54717	K
SSA_1331	3.196347	2.283105	5.022831	5.936073	7.305936	5.022831	2.283105	7.762557	5.936073	2.283105	4.56621	9.589041	0.913242	0.913242	5.479452	5.022831	3.196347	10.50228	6.849315	5.936073	
SSA_1582	3.043478	5.217391	5.217391	6.521739	6.086957	4.347826	0.434783	7.826087	3.913043	3.043478	4.347826	10	2.173913	2.173913	7.391304	3.913043	7.391304	3.043478	6.521739	7.391304	R
SSA_1255	2.671756	4.961832	4.198473	12.21374	6.870229	1.908397	3.435115	7.633588	4.198473	2.290076	1.526718	11.45038	0.381679	1.145038	4.198473	7.251908	5.725191	6.10687	5.725191	6.10687	L
SSA_0252	2.641509	3.773585	4.150943	4.90566	7.169811	4.90566	2.264151	7.924528	7.924528	2.641509	3.396226	12.45283	0.754717	1.509434	5.283019	5.283019	2.641509	7.169811	7.169811	6.037736	
SSA_2022	2.517986	5.755396	2.877698	5.755396	5.395683	2.877698	2.158273	10.07194	10.79137	3.956835	3.597122	10.43165	0.359712	1.438849	5.395683	5.755396	2.877698	10.43165	4.316547	3.23741	K
SSA_0382	2.413793	3.793103	6.896552	6.206897	5.862069	3.793103	2.758621	8.275862	6.206897	5.517241	2.758621	10.34483	0.344828	1.034483	6.896552	5.517241	4.827586	5.517241	5.862069	5.172414	K
SSA_1434	14.28571	5.357143	1.785714	0	16.07143	3.571429	1.785714	3.571429	1.785714	1.785714	1.785714	1.785714	0	0	1.785714	3.571429	8.928571	8.928571	0	23.21429	
SSA_0125	13.33333	5	3.333333	8.333333	10	8.333333	3.333333	10	3.333333	1.666667	5	8.333333	0	0	0	0	6.666667	5	1.666667	6.666667	J
SSA_1627	13.33333	5	5	0	5	5	3.333333	8.333333	0	0	3.333333	11.66667	3.333333	3.333333	3.333333	1.666667	6.666667	3.333333	5	13.33333	KT
SSA_0232	13.11475	0	6.557377	9.836066	8.196721	6.557377	3.278689	9.836066	0	0	3.278689	16.39344	0	0	3.278689	6.557377	4.918033	1.639344	0	6.557377	
SSA_1980	12.90323	8.064516	6.451613	6.451613	16.12903	6.451613	3.225806	1.612903	3.225806	1.612903	3.225806	4.83871	1.612903	1.612903	1.612903	1.612903	6.451613	1.612903	1.612903	9.677419	J
SSA_1745	12.5	0	4.6875	3.125	17.1875	4.6875	1.5625	1.5625	1.5625	0	0	3.125	0	0	0	3.125	12.5	14.0625	6.25	14.0625	S
SSA_2271	11.42857	4.285714	0	2.857143	8.571429	4.285714	5.714286	4.285714	1.428571	0	2.857143	12.85714	0	1.428571	1.428571	12.85714	8.571429	2.857143	2.857143	11.42857	
SSA_0188	10.38961	7.792208	1.298701	3.896104	5.194805	3.896104	2.597403	7.792208	12.98701	2.597403	1.298701	14.28571	0	0	1.298701	1.298701	6.493506	7.792208	1.298701	7.792208	
SSA_1651	9.090909	1.136364	4.545455	3.409091	7.954545	9.090909	3.409091	4.545455	3.409091	0	4.545455	6.818182	3.409091	1.136364	3.409091	4.545455	9.090909	10.22727	5.681818	4.545455	
SSA_2019	9.090909	1.136364	5.681818	5.681818	6.818182	6.818182	4.545455	11.36364	4.545455	0	0	6.818182	0	0	2.272727	5.681818	5.681818	6.818182	6.818182	10.22727	T
SSA_2241	9.090909	6.818182	9.090909	4.545455	6.818182	4.545455	1.136364	5.681818	3.409091	0	3.409091	7.954545	0	0	9.090909	2.272727	6.818182	9.090909	7.954545	2.272727	S
SSA_0394	8.695652	1.086957	5.434783	4.347826	7.608696	3.26087	7.608696	7.608696	4.347826	0	4.347826	9.782609	2.173913	1.086957	4.347826	5.434783	6.521739	3.26087	1.086957	11.95652	
SSA_1791	8.695652	4.347826	4.347826	5.434783	6.521739	4.347826	4.347826	7.608696	5.434783	0	4.347826	5.434783	2.173913	0	3.26087	5.434783	10.86957	2.173913	7.608696	7.608696	C
SSA_2092	8.695652	2.173913	7.608696	8.695652	7.608696	5.434783	5.434783	6.521739	2.173913	1.086957	0	13.04348	0	2.173913	1.086957	1.086957	9.782609	6.521739	5.434783	5.434783	G

SSA_0249	8.510638	3.191489	4.255319	6.382979	12.76596	5.319149	1.06383	6.382979	9.574468	0	0	9.574468	1.06383	0	1.06383	1.06383	8.510638	4.255319	7.446809	9.574468	
SSA_0210	8.421053	6.315789	2.105263	5.263158	6.315789	5.263158	1.052632	4.210526	4.210526	1.052632	0	12.63158	0	1.052632	4.210526	5.263158	7.368421	10.52632	5.263158	9.473684	
SSA_1440	7.692308	3.846154	2.884615	3.846154	10.57692	5.769231	1.923077	3.846154	3.846154	2.884615	1.923077	11.53846	0	0	5.769231	0	4.807692	13.46154	5.769231	9.615385	E
SSA_1397	7.407407	4.62963	5.555556	5.555556	9.259259	7.407407	2.777778	8.333333	5.555556	1.851852	2.777778	10.18519	0	0.925926	5.555556	1.851852	1.851852	8.333333	3.703704	6.481481	
SSA_1368	7.33945	1.834862	3.669725	6.422018	11.00917	9.174312	2.752294	7.33945	3.669725	0.917431	1.834862	8.256881	2.752294	0	1.834862	6.422018	6.422018	4.587156	6.422018	7.33945	V
SSA_0143	7.207207	1.801802	5.405405	9.009009	4.504505	4.504505	0.900901	2.702703	3.603604	0	0.900901	7.207207	1.801802	0	6.306306	5.405405	16.21622	4.504505	9.90991	8.108108	
SSA_0826	7.142857	4.464286	1.785714	3.571429	5.357143	8.928571	4.464286	9.821429	2.678571	0	4.464286	9.821429	1.785714	0.892857	4.464286	0.892857	4.464286	11.60714	8.035714	5.357143	R
SSA_0392	6.956522	2.608696	4.347826	4.347826	4.347826	3.478261	2.608696	9.565217	6.086957	3.478261	3.478261	12.17391	0.869565	1.73913	2.608696	4.347826	6.086957	6.086957	9.565217	5.217391	S
SSA_1895	6.896552	6.034483	6.034483	3.448276	11.2069	6.896552	6.034483	6.896552	5.172414	0.862069	1.724138	8.62069	0	0	3.448276	1.724138	6.034483	6.896552	7.758621	4.310345	J
SSA_0968	6.666667	1.666667	7.5	5.833333	11.66667	4.166667	1.666667	6.666667	6.666667	2.5	1.666667	6.666667	2.5	0	0.833333	4.166667	10	12.5	5.833333	0.833333	
SSA_1857	6.666667	6.666667	2.5	6.666667	9.166667	6.666667	1.666667	5.833333	6.666667	0	3.333333	7.5	0	0	2.5	5.833333	1.666667	11.66667	7.5	7.5	D
SSA_1921	6.666667	2.5	4.166667	5.833333	13.33333	7.5	0.833333	8.333333	3.333333	1.666667	1.666667	8.333333	1.666667	0	2.5	5.833333	10	5	4.166667	6.666667	S
SSA_1652	6.451613	5.645161	4.83871	3.225806	4.032258	1.612903	5.645161	4.032258	6.451613	2.419355	3.225806	9.677419	0	1.612903	4.032258	6.451613	9.677419	10.48387	5.645161	4.83871	J
SSA_0559	6.4	4.8	4	3.2	5.6	5.6	4	8.8	0.8	0.8	2.4	10.4	0.8	0.8	5.6	6.4	5.6	16	5.6	2.4	
SSA_0962	6.25	3.125	3.125	3.125	7.03125	3.90625	1.5625	3.125	0.78125	3.90625	6.25	10.15625	0	0.78125	8.59375	5.46875	8.59375	9.375	7.8125	7.03125	E
SSA_2276	6.25	1.5625	3.90625	16.40625	3.125	9.375	2.34375	6.25	3.125	0.78125	4.6875	3.90625	0	0	0.78125	4.6875	5.46875	9.375	3.90625	14.0625	
SSA_2196	6.015038	0	2.255639	9.022556	13.53383	5.263158	3.007519	5.263158	5.263158	0.75188	1.503759	7.518797	0.75188	0.75188	3.007519	3.759398	7.518797	8.270677	6.766917	9.774436	
SSA_0926	5.797101	5.797101	2.898551	3.623188	3.623188	3.623188	2.173913	5.797101	10.86957	2.173913	2.898551	11.5942	0.724638	0.724638	6.521739	5.797101	5.797101	5.072464	7.971014	6.521739	R
SSA_0187	5.714286	5	2.857143	8.571429	6.428571	5	1.428571	4.285714	10	1.428571	1.428571	15.71429	0	0	2.857143	7.857143	7.857143	7.142857	2.142857	4.285714	NU
SSA_1296	5.594406	5.594406	4.895105	14.68531	7.692308	2.097902	1.398601	6.293706	3.496503	2.097902	0.699301	7.692308	0	0	7.692308	4.895105	5.594406	6.993007	9.090909	3.496503	
SSA_1496	5.594406	2.097902	6.993007	5.594406	4.895105	4.895105	3.496503	8.391608	2.797203	0	3.496503	16.08392	1.398601	0.699301	3.496503	7.692308	7.692308	4.195804	2.797203	7.692308	
SSA_0207	5.555556	7.638889	3.472222	4.861111	4.861111	6.25	3.472222	9.722222	4.166667	1.388889	0.694444	22.22222	0.694444	0.694444	3.472222	4.861111	4.166667	4.861111	3.472222	3.472222	
SSA_0509	5.555556	3.472222	3.472222	3.472222	8.333333	6.944444	2.777778	8.333333	6.25	0.694444	2.083333	8.333333	0	0	3.472222	5.555556	6.944444	9.027778	4.861111	10.41667	E
SSA_1502	5.517241	2.758621	4.137931	11.03448	4.827586	8.965517	2.068966	5.517241	3.448276	0.689655	3.448276	7.586207	1.37931	1.37931	2.758621	2.758621	7.586207	9.655172	3.448276	11.03448	
SSA_0135	5.442177	2.721088	3.401361	6.122449	8.163265	6.802721	2.040816	5.442177	8.163265	5.442177	1.360544	12.92517	0	0.680272	1.360544	3.401361	2.040816	9.52381	3.401361	11.56463	K
SSA_1982	5.405405	6.081081	2.702703	8.783784	7.432432	5.405405	1.351351	8.783784	4.054054	2.702703	1.351351	10.13514	0	0.675676	6.081081	5.405405	1.351351	10.81081	5.405405	6.081081	KT
SSA_2315	5.263158	3.289474	3.289474	5.921053	9.868421	10.52632	0.657895	7.894737	4.605263	1.973684	4.605263	8.552632	1.315789	0	2.631579	1.973684	6.578947	4.605263	4.605263	11.84211	NU
SSA_1601	5.16129	7.741935	5.806452	1.935484	12.90323	5.16129	2.580645	6.451613	5.16129	2.580645	1.935484	9.032258	1.290323	0	3.225806	1.290323	7.741935	7.741935	4.516129	7.741935	O
SSA_0238	5.128205	2.564103	4.487179	4.487179	4.487179	2.564103	2.564103	3.205128	1.282051	0.641026	3.846154	8.974359	1.282051	0	6.410256	8.974359	5.128205	14.10256	7.692308	12.17949	
SSA_1438	5.128205	5.769231	0.641026	7.692308	5.128205	1.923077	4.487179	5.769231	5.128205	0.641026	5.769231	14.10256	1.282051	0.641026	5.128205	4.487179	4.487179	8.333333	8.333333	5.128205	R
SSA_2197	5.128205	3.205128	4.487179	6.410256	1.282051	4.487179	2.564103	9.615385	2.564103	1.282051	3.846154	20.51282	1.923077	1.282051	5.769231	8.974359	4.487179	1.282051	1.282051	9.615385	R
SSA_1289	5.063291	4.43038	5.063291	5.696203	10.75949	3.164557	3.797468	6.962025	2.531646	0	1.898734	9.493671	0.632911	0	5.696203	9.493671	4.43038	10.12658	7.594937	3.164557	
SSA_2380	5.031447	3.773585	4.402516	4.402516	11.32075	3.773585	5.031447	8.176101	5.031447	1.886792	3.144654	11.94969	0	0.628931	3.144654	5.031447	6.289308	8.176101	3.144654	5.660377	S
SSA_2312	4.938272	4.938272	2.469136	9.259259	8.024691	5.555556	4.320988	8.641975	4.320988	1.851852	3.08642	6.17284	1.234568	0	4.320988	2.469136	9.259259	6.17284	4.320988	8.641975	
SSA_1613	4.848485	3.636364	2.424242	3.636364	4.242424	5.454545	3.636364	5.454545	3.636364	3.636364	2.424242	9.69697	0	0	4.848485	4.848485	5.454545	18.18182	7.272727	6.666667	R
SSA_2228	4.848485	7.272727	3.636364	4.242424	4.848485	1.212121	1.212121	6.060606	4.848485	0.606061	2.424242	12.72727	1.212121	0.606061	3.030303	4.242424	11.51515	9.090909	6.666667	9.69697	R
SSA_2345	4.790419	6.586826	5.389222	5.389222	7.784431	4.790419	2.994012	5.988024	8.383234	3.592814	0.598802	10.77844	0.598802	0.598802	4.191617	7.185629	2.994012	6.586826	3.592814	7.185629	K
SSA_1747	4.705882	2.941176	2.941176	6.470588	4.117647	2.941176	1.176471	8.235294	5.882353	2.941176	4.705882	7.058824	0.588235	0.588235	7.058824	7.058824	7.058824	5.294118	10	8.235294	
SSA_2159	4.678363	7.602339	1.754386	6.432749	4.093567	4.093567	2.339181	4.678363	5.263158	1.754386	4.093567	11.11111	0.584795	1.169591	4.093567	7.602339	4.093567	9.94152	3.508772	11.11111	G
SSA_2322	4.624277	4.046243	2.890173	5.780347	8.092486	6.936416	1.734104	6.358382	8.092486	2.890173	2.312139	12.71676	1.156069	0.578035	4.046243	7.514451	1.156069	7.514451	6.358382	5.202312	K
SSA_0616	4.545455	1.704545	5.113636	4.545455	5.681818	2.840909	2.840909	7.386364	6.818182	0.568182	3.409091	11.93182	0.568182	0	2.840909	3.977273	5.681818	14.77273	6.818182	7.954545	R
SSA_0045	4.494382	3.370787	6.179775	6.179775	9.550562	3.370787	2.247191	10.67416	3.932584	2.247191	1.685393	14.60674	1.685393	1.123596	7.865169	5.05618	1.685393	6.179775	3.932584	3.932584	
SSA_1389	4.444444	3.888889	6.666667	5.555556	8.333333	4.444444	1.111111	11.66667	3.333333	0	3.888889	12.77778	1.666667	1.666667	3.888889	11.11111	5	3.888889	2.777778	3.888889	
SSA_1455	4.444444	2.777778	6.111111	6.111111	11.11111	5.555556	1.666667	1.666667	5.555556	4.444444	3.333333	11.11111	3.888889	0.555556	4.444444	4.444444	2.777778	8.333333	5.555556	6.111111	S
SSA_1336	4.41989	2.209945	7.734807	6.629834	9.392265	3.867403	1.657459	7.18232	1.657459	0.552486	1.657459	14.36464	1.104972	1.104972	7.18232	3.314917	3.867403	9.944751	6.629834	5.524862	R
SSA_1308	4.371585	7.650273	1.639344	6.557377	6.557377	2.185792	2.185792	6.010929	8.743169	1.639344	2.185792	10.38251	1.092896	0.546448	3.278689	2.73224	3.825137	8.743169	6.010929	13.6612	
SSA_2253	4.347826	2.717391	6.521739	3.804348	9.23913	5.434783	3.26087	4.891304	5.434783	2.717391	2.717391	8.152174	2.717391	2.173913	4.347826	6.521739	4.891304	5.978261	6.521739	7.608696	L
SSA_2243	4.210526	2.631579	6.842105	5.789474	9.473684	6.315789	3.684211	5.789474	6.842105	1.052632	2.105263	11.05263	0	0.526316	2.631579	2.631579	5.789474	11.05263	4.736842	6.842105	
SSA_1281	4.123711	2.57732	3.608247	2.57732	8.247423	3.608247	2.57732	6.701031	7.216495	3.608247	8.247423	9.793814	2.57732	0	4.639175	5.154639	5.670103	5.670103	6.701031	6.701031	L
SSA_1710	4.060914	4.568528	2.538071	6.091371	6.598985	3.045685	2.538071	12.69036	6.598985	1.522843	3.045685	13.19797	1.522843	0.507614	3.553299	6.598985	5.076142	6.091371	4.568528	5.583756	K
SSA_1192	4.020101	3.015075	3.015075	7.035176	11.55779	4.020101	1.507538	8.040201	1.507538	3.015075	1.507538	16.0804	1.507538	0.502513	4.020101	4.020101	6.030151	10.05025	5.527638	4.020101	
SSA_1335	3.960396	3.465347	5.940594	8.415842	6.930693	4.455446	3.465347	5.445545	3.960396	2.475248	2.970297	15.34653	0.990099	1.980198	3.960396	2.475248	4.950495	6.930693	4.950495	6.930693	R
SSA_0098	3.921569	3.431373	3.431373	3.921569	6.372549	5.392157	1.960784	9.313725	4.411765	3.431373	1.470588	9.803922	0.980392	0.490196	5.882353	3.921569	6.862745	8.333333	7.843137	8.823529	R
SSA_1603	3.864734	5.31401	3.864734	9.661836	7.729469	1.449275	2.415459	8.695652	4.830918	1.932367	2.898551	9.178744	3.864734	0	6.763285	5.31401	3.381643	6.763285	8.21256	3.864734	
SSA_1103	3.846154	6.25	4.807692	7.692308	4.807692	2.884615	3.365385	8.653846	1.442308	2.403846	1.923077	11.53846	1.442308	2.403846	6.25	9.134615	4.326923	6.25	6.25	4.326923	
SSA_1645	3.846154	2.884615	2.403846	6.730769	9.615385	4.807692	4.807692	5.288462	7.211538	0.961538	0.961538	12.5	0.961538	0.480769	3.365385	5.769231	5.288462	11.05769	4.326923	6.730769	K
SSA_1078	3.827751	2.392344	4.30622	3.349282	6.220096	3.827751	3.349282	7.655502	2.392344	2.392344	4.30622	17.70335	1.435407	0.478469	4.784689	12.44019	3.827751	2.870813	2.870813	9.569378	S
SSA_0195	3.773585	4.716981	2.358491	7.54717	3.301887	1.886792	4.245283	5.188679	4.716981	2.358491	3.301887	25.4717	0.471698	2.830189	2.830189	7.54717	5.188679	3.773585	0.943396	7.54717	
SSA_1746	3.686636	7.834101	3.225806	4.147465	6.912442	4.147465	1.843318	6.912442	6.451613	0.460829	3.225806	15.20737	0	0	6.451613	5.529954	2.304147	8.75576	7.834101	5.069124	
SSA_1514	3.088803	3.474903	6.177606	7.335907	8.108108	6.949807	3.088803	7.335907	3.474903	1.544402	3.474903	8.494208	2.702703	2.316602	6.563707	4.247104	3.861004	6.563707	4.633205	6.563707	M
SSA_2382	3.088803	5.405405	4.247104	7.335907	11.58301	1.158301	1.544402	11.19691	6.563707	0.772201	3.474903	12.35521	0.3861	0.3861	2.702703	3.474903	3.474903	12.74131	3.088803	5.019305	K
SSA_2187	2.846975	6.049822	3.202847	6.049822	8.896797	3.202847	0.711744	10.32028	7.47331	1.779359	2.491103	9.252669	1.067616	0	3.558719	2.491103	3.914591	10.67616	7.117438	8.896797	U
SSA_0141	12.5	1.388889	2.777778	5.555556	6.944444	8.333333	2.777778	1.388889	8.333333	2.777778	1.388889	8.333333	0	2.777778	4.166667	1.388889	2.777778	8.333333	6.944444	11.11111	P

SSA_1244	11.39241	3.797468	3.797468	10.12658	10.12658	1.265823	8.860759	11.39241	3.797468	0	1.265823	7.594937	1.265823	2.531646	2.531646	2.531646	3.797468	7.594937	2.531646	3.797468	
SSA_2332	11.39241	5.063291	5.063291	3.797468	2.531646	3.797468	6.329114	8.860759	0	0	1.265823	7.594937	1.265823	0	0	5.063291	5.063291	15.18987	12.65823	5.063291	IQ
SSA_2028	9.782609	0	1.086957	7.608696	17.3913	2.173913	1.086957	0	4.347826	1.086957	0	15.21739	0	0	4.347826	2.173913	8.695652	7.608696	6.521739	10.86957	
SSA_0357	8.653846	1.923077	1.923077	1.923077	7.692308	6.730769	3.846154	6.730769	4.807692	1.923077	3.846154	9.615385	1.923077	1.923077	0	3.846154	5.769231	9.615385	6.730769	10.57692	O
SSA_0558	8.653846	1.923077	5.769231	7.692308	8.653846	6.730769	2.884615	8.653846	1.923077	0.961538	3.846154	1.923077	0	0	4.807692	2.884615	12.5	8.653846	4.807692	6.730769	
SSA_1647	7.826087	1.73913	3.478261	2.608696	6.956522	2.608696	3.478261	1.73913	6.956522	0.869565	1.73913	12.17391	0	1.73913	8.695652	4.347826	1.73913	10.43478	15.65217	5.217391	
SSA_1347	7.758621	1.724138	7.758621	1.724138	12.93103	3.448276	2.586207	6.034483	1.724138	0.862069	5.172414	6.896552	0.862069	4.310345	2.586207	1.724138	7.758621	9.482759	7.758621	6.896552	P
SSA_0596	7.563025	4.201681	2.521008	6.722689	9.243697	4.201681	5.882353	4.201681	6.722689	1.680672	2.521008	10.92437	0.840336	0	4.201681	2.521008	7.563025	10.08403	2.521008	5.882353	
SSA_0414	7.317073	1.626016	3.252033	8.943089	8.130081	0	3.252033	8.943089	0.813008	2.439024	3.252033	17.88618	2.439024	0.813008	2.439024	5.691057	7.317073	3.252033	4.065041	8.130081	
SSA_0560	7.317073	2.439024	5.691057	9.756098	6.504065	4.878049	2.439024	10.56911	2.439024	0.813008	3.252033	6.504065	0.813008	0	6.504065	8.943089	4.878049	9.756098	4.878049	1.626016	
SSA_0051	7.258065	1.612903	4.83871	6.451613	4.032258	3.225806	2.419355	8.870968	4.032258	0.806452	1.612903	12.90323	0	0	1.612903	4.032258	8.064516	4.83871	9.677419	13.70968	S
SSA_2068	7.142857	5.555556	1.587302	8.730159	7.142857	7.142857	1.587302	6.349206	4.761905	0.793651	1.587302	11.90476	0.793651	0	1.587302	5.555556	5.555556	7.936508	6.349206	7.936508	
SSA_1914	7.086614	2.362205	2.362205	7.874016	5.511811	3.937008	3.937008	6.299213	3.937008	1.574803	3.149606	21.25984	3.937008	0.787402	3.149606	7.874016	3.937008	1.574803	5.511811	3.937008	S
SSA_0915	6.923077	3.846154	4.615385	8.461538	6.923077	3.846154	1.538462	3.076923	10	3.076923	2.307692	12.30769	1.538462	0.769231	5.384615	3.076923	3.846154	6.153846	4.615385	7.692308	S
SSA_2244	6.818182	6.060606	5.30303	6.818182	9.090909	6.818182	1.515152	9.090909	2.272727	0.757576	2.272727	11.36364	0.757576	1.515152	3.030303	0.757576	4.545455	9.090909	5.30303	6.818182	P
SSA_2122	6.666667	2.962963	2.962963	11.85185	5.185185	4.444444	1.481481	2.962963	1.481481	1.481481	2.962963	25.18519	2.962963	0	2.962963	12.59259	6.666667	0.740741	0	4.444444	
SSA_0458	6.617647	5.147059	3.676471	4.411765	4.411765	4.411765	2.941176	8.088235	9.558824	1.470588	3.676471	13.23529	0.735294	2.205882	3.676471	2.941176	5.882353	6.617647	5.147059	5.147059	J
SSA_1222	6.521739	5.072464	2.173913	5.797101	7.246377	4.347826	2.173913	12.31884	2.898551	2.898551	4.347826	9.42029	1.449275	0	3.623188	2.898551	3.623188	10.86957	7.971014	4.347826	F
SSA_0166	6.382979	3.546099	4.964539	7.092199	2.12766	2.12766	2.836879	4.964539	3.546099	3.546099	2.836879	18.43972	1.41844	0.70922	2.12766	8.510638	4.255319	9.219858	6.382979	4.964539	
SSA_0189	6.338028	6.338028	2.816901	5.633803	7.746479	2.816901	2.112676	2.816901	8.450704	2.112676	0.704225	14.78873	0.704225	0	2.816901	5.633803	7.042254	8.450704	7.042254	5.633803	U
SSA_0678	6.293706	6.293706	5.594406	6.993007	5.594406	6.993007	4.195804	10.48951	2.097902	2.797203	2.097902	10.48951	0.699301	0.699301	2.097902	2.097902	3.496503	8.391608	5.594406	6.993007	K
SSA_0969	6.206897	2.758621	4.827586	5.517241	8.965517	4.137931	2.068966	10.34483	9.655172	1.37931	2.068966	6.896552	2.068966	0	2.068966	2.758621	8.965517	8.965517	6.206897	4.137931	
SSA_0452	6.081081	5.405405	2.702703	8.108108	7.432432	4.054054	2.027027	6.756757	4.72973	0.675676	1.351351	12.16216	0.675676	0.675676	2.027027	5.405405	4.72973	8.783784	8.108108	8.108108	K
SSA_0641	6.040268	1.342282	2.684564	8.053691	8.053691	3.355705	2.684564	6.040268	4.697987	3.355705	4.026846	11.4094	0.671141	0	5.369128	4.026846	6.711409	8.724832	6.040268	6.711409	S
SSA_2314	6.040268	3.355705	5.369128	8.053691	9.395973	7.38255	0.671141	7.38255	6.040268	1.342282	4.697987	8.724832	1.342282	0	2.684564	2.013423	7.38255	4.026846	4.026846	10.06711	NU
SSA_0172	6	4	4.666667	7.333333	8.666667	2	4	6.666667	6	2	2.666667	10	0.666667	1.333333	1.333333	3.333333	4	9.333333	4.666667	11.33333	K
SSA_1676	5.960265	4.635762	3.311258	9.271523	6.622517	7.94702	3.311258	9.271523	5.960265	1.324503	2.649007	10.59603	0	0	3.97351	2.649007	3.97351	5.298013	7.284768	5.960265	KT
SSA_0255	5.882353	4.575163	4.575163	4.575163	8.496732	7.189542	3.267974	7.843137	6.535948	2.614379	1.960784	14.37908	1.30719	0	1.960784	3.921569	4.575163	5.882353	5.882353	4.575163	K
SSA_1350	5.806452	5.806452	1.290323	8.387097	5.806452	5.16129	3.225806	6.451613	2.580645	3.870968	3.225806	9.677419	2.580645	1.290323	6.451613	1.935484	7.741935	10.96774	5.806452	1.935484	F
SSA_1608	5.769231	4.487179	4.487179	6.410256	5.128205	4.487179	2.564103	5.128205	2.564103	1.923077	1.282051	12.17949	0.641026	0	4.487179	6.410256	7.692308	11.53846	7.051282	5.769231	F
SSA_1463	5.696203	5.696203	2.531646	5.063291	3.797468	4.43038	2.531646	6.329114	10.75949	0.632911	1.265823	11.39241	0.632911	0.632911	2.531646	4.43038	5.063291	8.860759	7.594937	10.12658	E
SSA_2235	5.660377	1.257862	3.773585	16.98113	3.773585	8.176101	1.257862	5.031447	10.06289	0.628931	2.515723	6.918239	0	0.628931	1.886792	1.257862	6.289308	6.918239	6.289308	10.69182	
SSA_0370	5.590062	4.347826	3.726708	6.832298	4.347826	4.347826	2.484472	3.726708	6.21118	3.726708	3.726708	11.18012	0.621118	0.621118	6.21118	3.726708	6.832298	7.453416	4.347826	9.937888	E
SSA_2330	5.590062	6.21118	2.484472	4.347826	1.863354	4.968944	2.484472	9.31677	7.453416	3.10559	4.968944	3.726708	1.242236	1.242236	5.590062	4.968944	8.695652	5.590062	7.453416	8.695652	
SSA_0623	5.555556	5.555556	5.555556	3.08642	5.555556	4.320988	4.320988	9.259259	1.851852	1.234568	4.938272	9.876543	1.851852	1.851852	4.938272	9.259259	4.320988	6.17284	4.938272	5.555556	
SSA_0580	5.389222	2.39521	4.790419	4.790419	9.580838	4.191617	2.39521	8.982036	5.389222	1.197605	1.796407	7.784431	1.197605	1.197605	4.191617	7.185629	5.389222	7.784431	8.982036	5.389222	Q
SSA_1782	5.202312	5.202312	4.624277	5.780347	4.046243	3.468208	2.312139	9.248555	4.046243	5.202312	3.468208	8.092486	1.156069	0.578035	4.046243	5.202312	6.936416	6.358382	9.248555	5.780347	R
SSA_1338	5.142857	2.857143	3.428571	10.85714	5.714286	5.142857	1.714286	9.714286	4	1.714286	4	8	1.714286	0	5.714286	5.142857	6.857143	7.428571	4.571429	6.285714	
SSA_1110	4.891304	7.065217	4.891304	3.804348	3.804348	3.804348	3.26087	4.891304	2.717391	3.804348	2.173913	7.065217	0	0.543478	5.978261	12.5	3.804348	14.67391	4.891304	5.434783	
SSA_0633	4.812834	3.743316	3.208556	5.347594	5.347594	1.604278	3.208556	9.625668	4.278075	2.673797	5.882353	8.55615	0.534759	0.534759	4.278075	4.278075	9.090909	6.951872	6.951872	9.090909	EH
SSA_1117	4.812834	0.534759	3.208556	12.83422	14.4385	4.812834	2.673797	4.812834	2.673797	0.534759	1.604278	10.69519	1.604278	2.139037	4.278075	3.743316	7.486631	4.812834	8.02139	4.278075	O
SSA_0047	4.787234	4.255319	3.191489	4.787234	9.574468	5.319149	2.659574	8.510638	5.851064	3.191489	4.787234	10.10638	2.12766	0.531915	3.723404	5.319149	6.382979	4.255319	5.851064	4.787234	R
SSA_0144	4.736842	2.631579	4.210526	6.315789	6.315789	6.315789	3.684211	10	5.263158	2.105263	6.315789	6.842105	2.631579	0.526316	4.210526	6.842105	2.105263	8.947368	4.736842	5.263158	K
SSA_1030	4.712042	3.664921	5.759162	9.424084	14.65969	4.188482	2.617801	7.329843	4.188482	0	1.570681	10.4712	1.570681	0	3.141361	5.759162	2.094241	10.4712	2.094241	6.282723	K
SSA_0489	4.639175	7.216495	1.030928	4.639175	3.092784	5.154639	0.515464	5.154639	8.247423	2.57732	3.092784	13.91753	2.57732	2.061856	2.061856	3.608247	5.670103	11.34021	4.639175	8.762887	H
SSA_0087	4.591837	7.142857	0.510204	9.693878	7.142857	1.530612	1.020408	3.571429	11.22449	1.530612	1.530612	12.2449	1.020408	0	2.55102	4.081633	3.061224	12.2449	5.612245	9.693878	C
SSA_0981	4.591837	3.061224	6.122449	3.061224	6.632653	6.122449	2.040816	7.142857	5.102041	1.530612	4.591837	9.693878	2.040816	0	5.612245	5.612245	7.142857	6.122449	8.673469	5.102041	E
SSA_2294	4.477612	2.985075	8.457711	6.965174	6.467662	5.472637	1.99005	5.970149	1.492537	3.9801	3.9801	7.960199	1.492537	0.497512	4.477612	7.960199	3.482587	11.9403	5.970149	3.9801	
SSA_0923	4.455446	4.950495	1.980198	5.445545	6.435644	4.455446	2.475248	3.960396	6.435644	3.960396	2.475248	15.34653	0.990099	0.990099	4.950495	5.445545	2.970297	10.39604	6.435644	5.445545	K
SSA_0949	4.455446	1.980198	3.960396	7.425743	11.38614	4.950495	1.980198	9.90099	2.970297	0.49505	3.465347	8.415842	0	0	7.425743	5.445545	10.39604	7.920792	5.445545	1.980198	
SSA_2325	4.455446	5.940594	3.960396	5.445545	5.940594	5.445545	2.970297	3.960396	4.455446	3.465347	4.455446	12.37624	1.485149	1.485149	3.465347	1.485149	8.910891	5.940594	5.445545	8.910891	L
SSA_0441	4.411765	6.372549	2.941176	5.882353	6.862745	5.882353	2.941176	7.843137	5.392157	2.45098	2.45098	11.76471	0	0.980392	4.901961	5.392157	3.431373	11.27451	4.411765	4.411765	K
SSA_0668	4.411765	4.901961	5.392157	3.431373	7.843137	4.901961	4.411765	6.862745	6.372549	1.470588	4.901961	7.843137	0.980392	1.470588	4.901961	4.901961	4.411765	9.313725	4.411765	6.862745	S
SSA_2137	4.285714	6.190476	1.904762	7.142857	5.714286	4.285714	2.857143	5.238095	9.047619	1.428571	4.761905	11.42857	0.952381	0	4.761905	4.761905	4.761905	8.571429	3.333333	8.571429	
SSA_2143	4.265403	2.369668	4.265403	14.69194	8.056872	9.004739	1.421801	6.161137	4.739336	0.473934	2.843602	4.739336	1.421801	0.473934	1.895735	2.369668	8.530806	7.582938	6.635071	8.056872	
SSA_1853	4.245283	4.245283	3.301887	8.490566	6.603774	6.603774	2.830189	8.962264	3.301887	4.245283	3.301887	7.54717	1.886792	2.830189	1.886792	5.660377	6.132075	7.54717	5.660377	4.716981	T
SSA_2363	4.186047	2.325581	2.790698	9.302326	7.44186	4.186047	0.930233	8.372093	4.651163	2.325581	4.186047	11.62791	0.930233	0.465116	3.255814	6.046512	7.906977	9.302326	5.116279	4.651163	R
SSA_1197	4.166667	5.092593	5.092593	3.703704	11.57407	3.240741	2.314815	5.092593	3.240741	0.462963	4.62963	12.03704	3.240741	0.462963	3.703704	5.555556	2.777778	7.407407	6.944444	9.259259	L
SSA_0989	4.072398	6.334842	4.072398	7.692308	4.524887	2.714932	3.61991	8.144796	6.78733	1.357466	3.167421	12.21719	0.904977	0	3.61991	2.714932	5.429864	7.692308	5.882353	9.049774	
SSA_1835	4.072398	5.429864	3.61991	5.429864	8.597285	5.882353	1.357466	5.882353	8.144796	0.904977	2.262443	10.40724	0.452489	4.524887	3.167421	6.334842	5.429864	9.049774	3.61991	5.429864	R
SSA_1474	4.017857	4.910714	4.910714	6.25	8.928571	8.035714	1.339286	8.035714	4.017857	0.892857	3.125	9.375	0	0.892857	6.696429	6.25	4.910714	8.035714	5.803571	3.571429	
SSA_1095	3.87931	3.017241	6.465517	8.189655	4.741379	6.465517	2.155172	6.465517	6.465517	1.293103	3.448276	6.465517	1.293103	0	7.758621	3.448276	7.758621	3.87931	6.896552	9.913793	NU
SSA_1249	3.813559	5.084746	5.508475	8.474576	9.322034	7.627119	1.271186	8.474576	0.847458	0.423729	2.966102	7.627119	0.423729	0.423729	4.237288	5.508475	8.050847	11.01695	4.237288	4.661017	L

SSA_1252	3.688525	5.327869	2.04918	10.2459	6.557377	4.508197	3.278689	6.147541	6.557377	2.459016	2.459016	13.52459	0.409836	0.819672	3.688525	5.327869	5.327869	10.65574	2.868852	4.098361	S
SSA_0990	3.673469	2.857143	3.673469	6.938776	3.673469	2.44898	4.081633	9.387755	5.306122	2.44898	2.44898	19.59184	1.22449	0.408163	4.081633	8.979592	6.122449	2.857143	2.040816	7.755102	
SSA_1028	3.501946	5.058366	2.723735	5.836576	9.727626	4.280156	1.167315	8.560311	5.447471	1.167315	5.447471	11.28405	0.77821	0	5.058366	3.11284	4.669261	8.171206	7.003891	7.003891	K
SSA_1916	3.333333	2.222222	8.888889	3.703704	8.888889	5.925926	2.592593	5.925926	3.703704	1.481481	3.703704	12.22222	0.740741	0.37037	3.703704	5.185185	4.814815	7.037037	7.037037	8.518519	R
SSA_0403	3.249097	2.166065	3.249097	6.137184	5.776173	5.054152	1.444043	9.025271	2.527076	0.722022	4.33213	14.80144	4.693141	1.083032	4.693141	9.747292	7.581227	3.610108	2.888087	7.220217	
SSA_0615	3.114187	4.152249	3.460208	8.304498	6.228374	5.190311	1.038062	5.536332	6.920415	3.460208	0.692042	13.14879	0.692042	2.076125	3.806228	6.574394	3.460208	10.0346	4.49827	7.612457	
SSA_0367	3	2.333333	3	5.333333	6.333333	5.333333	2.666667	7	6	1.666667	5.666667	12	1.666667	1	5.666667	3.666667	7.333333	7.666667	4.666667	8	O
SSA_2038	2.8125	5.9375	4.375	6.25	3.125	5.9375	1.5625	6.25	5.3125	3.125	1.875	18.4375	0.9375	0.625	3.75	5.3125	3.75	6.5625	4.0625	10	S
SSA_0009	11.36364	7.954545	2.272727	5.681818	15.90909	4.545455	3.409091	6.818182	0	0	1.136364	9.090909	0	0	2.272727	1.136364	3.409091	9.090909	9.090909	6.818182	J
SSA_0651	11.23596	2.247191	3.370787	4.494382	7.865169	3.370787	2.247191	13.48315	1.123596	0	4.494382	14.60674	0	0	3.370787	10.11236	6.741573	3.370787	2.247191	5.617978	
SSA_1899	10.20408	9.183673	5.102041	4.081633	13.26531	2.040816	2.040816	5.102041	3.061224	1.020408	2.040816	8.163265	0	0	3.061224	4.081633	5.102041	8.163265	7.142857	7.142857	K
SSA_2052	9.615385	1.923077	0.961538	2.884615	8.653846	6.730769	3.846154	7.692308	5.769231	0.961538	3.846154	7.692308	1.923077	1.923077	0	3.846154	6.730769	7.692308	8.653846	8.653846	O
SSA_0268	9.52381	1.904762	0.952381	5.714286	9.52381	3.809524	8.571429	3.809524	3.809524	0	3.809524	13.33333	0	0.952381	2.857143	0.952381	5.714286	9.52381	5.714286	9.52381	G
SSA_1941	9.345794	11.21495	6.542056	5.607477	4.672897	8.411215	5.607477	3.738318	3.738318	3.738318	2.803738	12.14953	0	0	2.803738	1.869159	5.607477	6.542056	4.672897	0.934579	K
SSA_1205	8.695652	5.217391	3.478261	6.086957	6.086957	7.826087	1.73913	5.217391	5.217391	1.73913	2.608696	4.347826	0	2.608696	1.73913	7.826087	3.478261	7.826087	5.217391	13.04348	
SSA_1669	8.695652	0.869565	0.869565	5.217391	2.608696	3.478261	6.086957	8.695652	1.73913	0.869565	2.608696	20.86957	0.869565	1.73913	1.73913	6.086957	12.17391	0.869565	2.608696	11.30435	
SSA_1515	8.403361	1.680672	4.201681	10.08403	6.722689	1.680672	5.042017	15.96639	5.882353	0.840336	0.840336	13.44538	1.680672	0	0.840336	10.08403	1.680672	5.882353	1.680672	3.361345	S
SSA_1083	8.064516	4.83871	6.451613	4.032258	3.225806	7.258065	2.419355	8.064516	3.225806	4.032258	3.225806	8.870968	0.806452	0.806452	1.612903	7.258065	3.225806	13.70968	4.83871	4.032258	S
SSA_0538	7.936508	5.555556	1.587302	7.142857	5.555556	3.174603	0.793651	7.142857	2.380952	2.380952	6.349206	11.11111	0	0.793651	3.968254	3.968254	5.555556	5.555556	7.936508	11.11111	
SSA_0245	7.692308	2.307692	3.846154	7.692308	10	2.307692	6.923077	12.30769	3.846154	3.076923	1.538462	6.923077	0	0.769231	1.538462	2.307692	10	7.692308	5.384615	3.846154	
SSA_0865	7.692308	3.076923	1.538462	4.615385	7.692308	6.923077	3.846154	6.923077	10	4.615385	1.538462	8.461538	1.538462	0	4.615385	6.153846	5.384615	6.923077	6.923077	1.538462	S
SSA_1906	7.352941	2.941176	4.411765	4.411765	5.147059	5.882353	2.941176	8.088235	2.941176	4.411765	5.147059	6.617647	0	1.470588	1.470588	5.147059	3.676471	6.617647	8.088235	13.23529	FGR
SSA_0066	7.29927	2.189781	5.109489	3.649635	8.759124	9.489051	2.189781	4.379562	7.29927	0.729927	0.729927	7.29927	0.729927	0	5.839416	5.839416	15.32847	4.379562	5.109489	3.649635	S
SSA_0309	7.194245	2.158273	3.597122	8.633094	2.877698	1.438849	2.158273	8.633094	7.194245	2.877698	4.316547	7.194245	0.719424	0.719424	3.597122	8.633094	3.597122	11.51079	6.47482	6.47482	
SSA_1381	7.194245	7.913669	2.158273	3.597122	8.633094	2.877698	1.438849	8.633094	4.316547	2.158273	2.877698	11.51079	0	0.719424	3.597122	4.316547	6.47482	14.38849	4.316547	2.877698	
SSA_1646	6.711409	2.013423	2.013423	4.697987	6.711409	4.026846	1.342282	4.026846	5.369128	1.342282	4.026846	9.395973	2.013423	1.342282	3.355705	5.369128	5.369128	12.08054	7.38255	11.4094	S
SSA_2357	6.666667	4	1.333333	4	15.33333	4	1.333333	10.66667	4.666667	1.333333	2.666667	6.666667	0	0	1.333333	3.333333	8.666667	10	4	10	J
SSA_0970	6.578947	1.973684	8.552632	3.289474	9.210526	7.236842	2.631579	7.236842	5.263158	1.973684	3.947368	9.868421	1.315789	0	2.631579	3.289474	7.894737	7.894737	5.263158	3.947368	
SSA_2281	6.578947	2.631579	3.289474	9.210526	8.552632	4.605263	1.973684	5.263158	7.894737	0.657895	1.973684	11.84211	0.657895	0	2.631579	6.578947	5.263158	7.894737	5.263158	7.236842	
SSA_0604	6.535948	4.575163	1.30719	3.921569	10.45752	4.575163	3.921569	5.228758	4.575163	3.267974	1.960784	9.150327	1.30719	0.653595	5.228758	5.228758	2.614379	8.496732	5.228758	11.76471	
SSA_2134	6.493506	2.597403	6.493506	7.792208	9.090909	3.246753	1.948052	12.33766	6.493506	1.298701	2.597403	9.090909	0	0	5.194805	6.493506	1.948052	7.792208	4.545455	4.545455	H
SSA_2184	6.410256	7.692308	3.846154	1.923077	4.487179	3.205128	4.487179	8.333333	3.846154	3.846154	1.923077	7.692308	0.641026	2.564103	1.923077	3.205128	6.410256	13.46154	2.564103	11.53846	FJ
SSA_1280	6.17284	2.469136	5.555556	5.555556	7.407407	7.407407	4.938272	4.938272	4.320988	1.851852	3.08642	11.11111	0	0	4.320988	6.17284	4.320988	8.024691	8.024691	4.320988	S
SSA_0018	6.024096	1.807229	4.819277	7.831325	1.807229	4.216867	2.409639	12.04819	3.012048	2.409639	3.012048	23.49398	0.60241	1.807229	3.012048	12.6506	2.409639	1.807229	2.409639	2.409639	M
SSA_2091	6.024096	3.012048	3.012048	9.638554	5.421687	4.819277	3.012048	7.228916	3.614458	1.204819	5.421687	11.44578	0.60241	0.60241	3.614458	1.807229	4.819277	10.84337	4.216867	9.638554	GT
SSA_2255	5.952381	1.785714	2.97619	8.928571	14.28571	2.380952	1.190476	5.952381	3.571429	0	2.380952	12.5	2.380952	0	3.571429	6.547619	3.571429	9.52381	4.761905	7.738095	K
SSA_0913	5.91716	3.550296	1.775148	2.95858	8.87574	5.325444	1.775148	2.95858	4.733728	2.366864	1.183432	9.467456	2.95858	0	4.733728	8.87574	6.508876	13.60947	5.325444	7.100592	R
SSA_0536	5.847953	5.847953	5.263158	7.602339	8.77193	2.339181	0.584795	7.602339	4.093567	0.584795	3.508772	8.187135	0.584795	0.584795	4.093567	1.754386	5.263158	10.52632	5.847953	11.11111	Q
SSA_1910	5.780347	5.202312	3.468208	4.624277	4.046243	2.312139	2.890173	6.936416	5.202312	2.312139	0	13.87283	1.156069	0.578035	3.468208	4.624277	6.936416	11.56069	5.202312	9.82659	J
SSA_1196	5.747126	6.321839	2.298851	4.597701	7.471264	1.724138	2.298851	4.597701	7.471264	3.448276	1.724138	9.770115	3.448276	0.574713	4.597701	6.896552	5.747126	8.045977	8.045977	5.172414	J
SSA_1690	5.714286	1.142857	6.857143	8.571429	6.857143	5.714286	2.285714	12.57143	1.142857	0	2.285714	17.14286	2.285714	0	2.857143	6.285714	4.571429	2.857143	3.428571	7.428571	
SSA_0995	5.617978	5.05618	4.494382	5.617978	10.11236	3.370787	3.932584	5.617978	3.932584	0.561798	3.370787	8.988764	1.685393	1.685393	8.426966	2.247191	7.865169	6.179775	5.617978	5.617978	R
SSA_0629	5.586592	3.351955	5.586592	5.027933	6.145251	5.027933	2.234637	8.938547	5.027933	0	3.910615	16.75978	3.351955	1.675978	2.234637	7.821229	7.26257	3.351955	3.351955	3.351955	V
SSA_2242	5.555556	1.111111	3.888889	10	10.55556	6.111111	2.222222	6.666667	4.444444	1.111111	2.777778	12.22222	0	0.555556	2.777778	1.666667	5	10	6.111111	7.222222	
SSA_1353	5.524862	4.41989	6.077348	7.734807	7.18232	3.867403	1.657459	3.314917	4.972376	1.104972	2.762431	11.60221	1.657459	0	4.972376	7.18232	5.524862	8.287293	5.524862	6.629834	S
SSA_1202	5.464481	2.185792	4.918033	3.825137	7.650273	10.38251	3.278689	8.743169	4.371585	1.639344	4.918033	10.92896	0.546448	0.546448	1.092896	2.73224	3.278689	5.464481	4.918033	13.11475	H
SSA_1641	5.464481	3.278689	4.371585	2.73224	7.103825	5.464481	1.639344	7.103825	7.103825	1.639344	3.825137	9.289617	0.546448	1.092896	4.371585	3.278689	5.464481	13.6612	4.371585	8.196721	LR
SSA_1328	5.434783	6.521739	5.978261	4.891304	8.152174	4.347826	1.630435	7.065217	3.26087	0.543478	3.804348	9.782609	1.086957	1.630435	8.152174	4.891304	2.717391	10.86957	7.608696	1.630435	
SSA_1614	5.291005	6.349206	5.820106	4.232804	8.465608	6.878307	3.174603	6.349206	0.529101	3.174603	2.645503	9.52381	1.058201	0	6.349206	3.174603	5.291005	8.994709	4.761905	7.936508	J
SSA_0050	5.208333	2.604167	1.041667	8.854167	8.333333	3.125	4.6875	5.208333	4.166667	1.041667	2.604167	8.854167	1.041667	0	1.5625	2.604167	10.9375	6.25	8.333333	13.54167	G
SSA_1923	5.208333	3.645833	4.166667	4.166667	10.41667	4.6875	2.083333	6.770833	4.6875	1.5625	3.125	11.45833	1.5625	1.041667	4.6875	5.729167	4.6875	7.291667	4.6875	8.333333	R
SSA_1396	5.154639	3.092784	3.092784	6.701031	4.123711	4.123711	2.061856	10.82474	2.57732	2.57732	2.57732	16.49485	3.608247	0.515464	6.701031	9.793814	6.185567	1.546392	2.57732	5.670103	
SSA_0310	5.128205	5.641026	4.102564	7.179487	7.692308	3.589744	2.051282	4.615385	4.102564	3.076923	0.512821	13.33333	1.538462	0.512821	1.025641	5.128205	6.666667	11.79487	3.589744	8.717949	K
SSA_0948	5.076142	2.030457	6.091371	3.553299	10.6599	7.106599	1.522843	10.15228	1.522843	2.030457	3.553299	9.64467	0	0	6.598985	3.045685	9.64467	7.106599	7.106599	3.553299	
SSA_0421	5.050505	3.535354	3.030303	3.535354	6.565657	7.070707	4.040404	2.020202	6.565657	2.525253	2.525253	9.090909	2.525253	1.515152	4.040404	6.565657	8.585859	6.565657	8.585859	6.060606	G
SSA_0431	5.025126	3.015075	3.517588	9.045226	3.015075	10.55276	2.01005	5.527638	6.030151	4.020101	4.020101	12.0603	1.005025	0.502513	3.517588	4.522613	5.527638	7.035176	6.030151	4.020101	S
SSA_1337	5.025126	2.512563	5.025126	11.05528	10.55276	3.015075	2.512563	10.55276	4.020101	2.01005	3.015075	11.55779	1.005025	0.502513	2.01005	6.030151	3.517588	9.045226	3.517588	3.517588	
SSA_1859	5	4	4	8	9	3	4	9.5	4.5	4.5	6	7.5	0	0.5	7	4	5.5	7.5	2	4.5	R
SSA_2226	4.975124	4.477612	4.975124	5.472637	6.965174	3.9801	3.9801	5.970149	5.472637	0.497512	3.482587	12.43781	2.985075	1.492537	3.482587	3.482587	7.462687	8.457711	6.965174	2.985075	O
SSA_0642	4.545455	4.545455	1.818182	7.272727	3.181818	3.181818	2.272727	10.45455	3.181818	1.363636	3.181818	19.54545	0.454545	4.090909	2.727273	10.45455	5.909091	1.818182	2.727273	7.272727	NOU
SSA_0090	4.329004	8.658009	4.329004	5.194805	6.060606	3.463203	2.164502	4.761905	6.493506	0.865801	4.761905	9.52381	2.597403	0.4329	5.194805	3.896104	5.194805	7.359307	7.792208	6.926407	J
SSA_1293	4.329004	5.194805	3.896104	6.926407	6.926407	3.463203	1.731602	7.359307	5.627706	1.731602	4.761905	12.12121	0.865801	1.731602	7.359307	3.463203	2.164502	8.658009	5.627706	6.060606	
SSA_0160	4.273504	5.982906	8.119658	8.547009	8.974359	5.982906	2.991453	6.837607	6.410256	0.42735	1.709402	8.547009	0.854701	0	2.991453	3.418803	5.128205	10.68376	4.700855	3.418803	

SSA_1664	4.065041	2.439024	3.252033	4.065041	12.19512	3.658537	3.252033	6.910569	3.658537	2.03252	2.845528	9.756098	1.626016	1.219512	4.878049	4.878049	6.504065	4.878049	10.1626	7.723577	H
SSA_1648	4.048583	2.834008	2.42915	6.882591	4.8583	2.42915	1.619433	4.453441	8.097166	0.809717	3.643725	9.716599	2.834008	1.214575	5.263158	7.287449	7.287449	8.502024	6.072874	9.716599	M
SSA_0311	4.032258	5.241935	2.822581	6.854839	8.870968	4.032258	2.419355	4.435484	4.032258	1.209677	3.225806	12.09677	2.419355	1.612903	3.629032	5.241935	3.629032	10.8871	6.048387	7.258065	L
SSA_1333	4.032258	3.225806	4.032258	5.241935	8.467742	3.629032	1.209677	8.870968	3.225806	2.016129	3.629032	10.08065	2.822581	0.806452	8.467742	3.629032	4.83871	9.677419	6.854839	5.241935	
SSA_2328	4.032258	4.032258	2.016129	7.66129	9.274194	2.822581	1.612903	9.677419	7.258065	3.225806	2.822581	10.8871	0.806452	0	3.225806	5.241935	3.629032	6.451613	7.66129	7.66129	KG
SSA_1439	4.016064	3.614458	2.409639	4.819277	5.62249	2.409639	1.606426	5.220884	3.212851	4.819277	3.614458	12.4498	0	0.803213	7.630522	5.62249	6.024096	10.44177	8.433735	7.228916	ER
SSA_1572	3.952569	5.928854	3.162055	6.324111	5.928854	4.347826	4.347826	6.324111	5.533597	1.581028	5.533597	12.25296	1.581028	1.976285	3.162055	3.557312	5.533597	9.090909	4.743083	5.13834	H
SSA_1525	3.508772	2.45614	4.210526	9.473684	8.77193	6.666667	1.754386	7.719298	6.315789	2.45614	3.157895	6.666667	1.052632	0	5.964912	4.210526	4.210526	7.368421	5.614035	8.421053	M
SSA_0103	3.496503	3.496503	3.846154	8.041958	5.594406	7.342657	2.447552	9.440559	5.244755	1.048951	3.146853	16.43357	0.699301	0.34965	5.244755	6.293706	4.895105	2.447552	2.447552	8.041958	S
SSA_2216	3.257329	5.863192	3.257329	6.514658	9.446254	4.560261	3.908795	5.537459	2.605863	2.605863	3.908795	10.74919	1.302932	0.651466	6.840391	4.885993	5.863192	7.166124	7.166124	3.908795	M
SSA_2181	3.215434	4.180064	3.536977	8.038585	7.073955	6.109325	0.96463	5.466238	5.466238	2.250804	4.180064	7.717042	1.607717	0	4.501608	4.180064	7.395498	9.324759	9.003215	5.787781	
SSA_0191	3.115265	2.492212	4.984424	5.607477	5.919003	4.672897	2.180685	7.788162	6.853583	3.115265	3.738318	12.77259	0.934579	0	4.049844	4.984424	3.738318	7.165109	7.165109	8.722741	L
SSA_1170	2.923977	1.754386	10.52632	7.894737	11.9883	6.140351	1.461988	9.064327	2.339181	1.169591	3.216374	9.94152	1.461988	0.584795	5.263158	4.97076	4.093567	7.894737	5.847953	1.461988	
SSA_0900	2.444988	3.91198	2.200489	4.156479	3.422983	3.91198	0.733496	3.178484	6.845966	0.733496	2.444988	16.62592	0.488998	0.244499	6.601467	3.91198	3.178484	13.20293	7.334963	14.42543	G
SSA_0043	14.66667	0	4	4	9.333333	2.666667	1.333333	5.333333	4	2.666667	4	10.66667	0	0	5.333333	2.666667	1.333333	12	8	8	
SSA_1268	12.64368	6.896552	4.597701	3.448276	9.195402	6.896552	2.298851	6.896552	5.747126	0	0	9.195402	0	0	1.149425	3.448276	1.149425	9.195402	10.34483	6.896552	E
SSA_1276	11.45833	2.083333	4.166667	7.291667	5.208333	5.208333	2.083333	3.125	3.125	6.25	5.208333	10.41667	0	1.041667	1.041667	5.208333	6.25	10.41667	4.166667	6.25	S
SSA_1771	10.78431	7.843137	4.901961	5.882353	4.901961	0.980392	0.980392	4.901961	3.921569	2.941176	3.921569	12.7451	2.941176	1.960784	2.941176	0	9.803922	4.901961	4.901961	7.843137	L
SSA_0163	9.90991	9.90991	1.801802	6.306306	10.81081	3.603604	1.801802	3.603604	4.504505	2.702703	1.801802	8.108108	1.801802	0	9.009009	2.702703	2.702703	9.009009	6.306306	3.603604	
SSA_2201	9.482759	2.586207	2.586207	5.172414	6.034483	6.034483	3.448276	7.758621	2.586207	1.724138	3.448276	17.24138	2.586207	0.862069	5.172414	7.758621	1.724138	4.310345	2.586207	6.896552	
SSA_0663	9.322034	5.084746	0.847458	3.389831	5.932203	2.542373	1.694915	7.627119	8.474576	4.237288	2.542373	6.779661	0.847458	0.847458	5.084746	4.237288	7.627119	13.55932	2.542373	6.779661	R
SSA_0815	9.322034	0.847458	2.542373	6.779661	2.542373	6.779661	3.389831	8.474576	5.084746	0	2.542373	16.94915	0.847458	0	4.237288	7.627119	6.779661	3.389831	0.847458	11.01695	S
SSA_0795	9.090909	1.652893	6.61157	5.785124	3.305785	0.826446	4.132231	12.39669	5.785124	2.479339	2.479339	12.39669	3.305785	0	1.652893	11.57025	6.61157	3.305785	0	6.61157	
SSA_1267	8.870968	2.419355	1.612903	9.677419	5.645161	4.032258	2.419355	6.451613	3.225806	0	0.806452	20.96774	1.612903	0.806452	4.83871	3.225806	11.29032	1.612903	2.419355	8.064516	D
SSA_0822	8.661417	0.787402	5.511811	3.937008	9.448819	3.937008	0.787402	10.23622	3.149606	0	3.149606	14.96063	0.787402	0	0.787402	5.511811	5.511811	8.661417	2.362205	11.81102	M
SSA_0214	8.396947	9.160305	4.580153	7.633588	4.580153	6.10687	0.763359	2.290076	2.290076	2.290076	1.526718	11.45038	0.763359	0.763359	3.816794	2.290076	5.343511	11.45038	3.053435	11.45038	L
SSA_0126	7.534247	7.534247	4.794521	6.164384	10.9589	4.794521	0.684932	5.479452	3.424658	0.684932	2.739726	8.219178	0	0	0.684932	3.424658	15.06849	7.534247	1.369863	8.90411	J
SSA_2313	7.482993	3.401361	2.040816	8.843537	10.88435	6.122449	0.680272	8.843537	5.442177	2.040816	4.081633	8.163265	1.360544	0	2.040816	2.721088	5.442177	4.761905	5.442177	10.20408	NU
SSA_0885	7.38255	3.355705	4.026846	11.4094	9.395973	2.684564	3.355705	6.711409	6.711409	2.013423	4.026846	8.053691	1.342282	2.013423	1.342282	4.697987	4.026846	8.053691	4.697987	4.697987	S
SSA_1488	7.38255	6.711409	4.697987	5.369128	10.06711	2.684564	1.342282	7.38255	5.369128	0	1.342282	10.06711	1.342282	0	3.355705	2.684564	5.369128	12.08054	3.355705	9.395973	K
SSA_1586	7.333333	3.333333	3.333333	8	7.333333	5.333333	2.666667	8.666667	5.333333	2	2.666667	10.66667	0	1.333333	0.666667	2	6.666667	6	6	10.66667	K
SSA_1986	7.236842	1.973684	1.973684	7.236842	9.868421	2.631579	1.315789	5.921053	5.921053	1.315789	3.947368	9.210526	2.631579	1.973684	5.263158	3.947368	4.605263	9.868421	3.947368	9.210526	E
SSA_2200	7.142857	7.142857	1.948052	9.090909	6.493506	3.896104	3.246753	10.38961	5.844156	0.649351	0.649351	8.441558	0	0.649351	3.246753	3.246753	9.090909	7.142857	5.844156	5.844156	K
SSA_1020	7.096774	0.645161	3.225806	2.580645	4.516129	1.290323	3.225806	5.806452	3.225806	1.290323	5.806452	20	0.645161	1.290323	10.32258	9.677419	6.451613	7.096774	0	5.806452	
SSA_0743	7.051282	5.128205	3.846154	6.410256	6.410256	4.487179	3.846154	7.692308	5.769231	1.923077	3.846154	11.53846	0.641026	2.564103	1.923077	4.487179	3.205128	7.692308	5.769231	5.769231	K
SSA_0281	6.918239	3.773585	3.773585	8.805031	8.805031	4.402516	1.257862	13.20755	2.515723	0	1.257862	15.09434	4.402516	0	3.144654	8.805031	3.773585	3.144654	1.257862	5.660377	
SSA_2000	6.875	1.875	3.125	3.75	6.25	4.375	3.75	6.875	2.5	1.25	2.5	13.125	0.625	0.625	3.75	10	12.5	2.5	1.25	12.5	S
SSA_1354	6.666667	3.636364	2.424242	4.242424	7.878788	3.636364	3.636364	7.878788	4.848485	0.606061	1.212121	6.666667	1.818182	0.606061	6.060606	6.060606	6.666667	9.69697	6.060606	9.69697	R
SSA_0780	6.626506	4.819277	1.204819	6.024096	4.819277	4.216867	2.409639	6.626506	3.012048	1.204819	4.819277	17.46988	3.012048	1.807229	4.819277	7.831325	9.638554	1.204819	1.807229	6.626506	
SSA_1799	6.470588	3.529412	3.529412	6.470588	2.941176	2.941176	1.764706	7.647059	6.470588	2.941176	4.705882	10	1.764706	0.588235	2.352941	4.117647	7.647059	9.411765	5.294118	9.411765	R
SSA_1284	6.214689	2.259887	5.649718	2.259887	8.474576	3.389831	1.694915	12.42938	2.824859	3.389831	3.954802	10.16949	1.129944	0.564972	5.084746	7.344633	7.909605	6.779661	5.084746	3.389831	S
SSA_2008	6.179775	5.617978	3.932584	3.370787	6.179775	2.247191	2.808989	5.05618	6.741573	4.494382	3.932584	7.865169	0	0	2.808989	1.685393	2.808989	15.16854	6.741573	12.35955	O
SSA_0763	6.111111	1.666667	3.333333	4.444444	7.222222	5	2.777778	9.444444	1.666667	3.333333	1.666667	15.55556	0.555556	0.555556	3.888889	9.444444	6.666667	3.888889	3.333333	9.444444	
SSA_1388	6.077348	3.314917	3.314917	8.287293	9.944751	4.41989	1.104972	6.077348	2.762431	2.762431	2.209945	17.12707	0.552486	0	8.287293	8.839779	4.972376	3.867403	2.209945	3.867403	
SSA_1327	6.010929	3.825137	4.918033	5.464481	8.743169	3.825137	1.092896	7.103825	5.464481	1.092896	3.278689	12.02186	0.546448	0.546448	6.557377	5.464481	2.73224	10.38251	6.557377	4.371585	
SSA_2133	5.913978	4.301075	5.376344	8.602151	8.602151	3.225806	3.225806	7.526882	3.225806	2.688172	2.150538	9.139785	2.688172	1.075269	3.763441	3.763441	3.763441	9.677419	6.989247	4.301075	L
SSA_2012	5.789474	5.263158	1.578947	6.315789	8.947368	2.631579	3.684211	6.842105	4.736842	1.578947	3.157895	12.10526	2.631579	2.631579	5.789474	6.842105	1.052632	6.315789	5.263158	6.842105	
SSA_1424	5.612245	4.081633	4.081633	7.142857	5.612245	3.571429	2.040816	4.591837	5.612245	3.061224	2.55102	14.28571	1.530612	0	3.061224	4.081633	4.591837	10.71429	6.122449	7.653061	R
SSA_1731	5.612245	4.081633	5.102041	8.163265	4.081633	6.122449	5.612245	9.693878	4.591837	1.530612	2.55102	8.163265	0.510204	0	3.571429	2.040816	7.653061	6.632653	6.122449	8.163265	OU
SSA_2364	5.612245	1.530612	7.142857	9.693878	3.571429	10.71429	2.040816	4.081633	3.061224	0.510204	3.061224	6.122449	2.040816	0	3.571429	1.020408	9.183673	5.102041	2.55102	19.38776	M
SSA_2337	5.527638	1.507538	2.01005	6.030151	6.532663	5.527638	2.01005	8.040201	2.01005	1.005025	3.517588	14.07035	0.502513	0	3.015075	9.045226	7.035176	6.532663	4.020101	12.0603	S
SSA_0721	5.472637	1.99005	5.472637	3.9801	5.472637	6.467662	1.492537	5.970149	1.492537	4.477612	4.975124	8.955224	2.487562	0	4.477612	3.9801	4.975124	8.955224	5.970149	12.93532	P
SSA_0694	5.418719	4.926108	2.463054	4.926108	7.389163	5.91133	0.492611	5.418719	5.91133	0.492611	2.955665	13.30049	1.477833	1.477833	2.463054	5.91133	5.418719	11.82266	6.896552	4.926108	F
SSA_1670	5.418719	4.433498	3.940887	6.896552	8.374384	2.463054	2.955665	8.374384	4.926108	2.463054	1.477833	5.91133	0.985222	0	5.91133	6.896552	4.926108	11.82266	3.940887	7.881773	K
SSA_0253	5.392157	2.941176	3.921569	7.352941	3.921569	4.411765	2.45098	8.333333	3.921569	0.980392	2.45098	20.58824	3.431373	0.490196	1.470588	8.823529	6.862745	2.45098	1.470588	8.333333	
SSA_0390	5.238095	3.809524	5.714286	3.333333	4.761905	4.285714	1.428571	7.142857	8.571429	0.952381	3.809524	16.19048	0.47619	0	6.190476	2.380952	4.285714	7.619048	5.714286	8.095238	E
SSA_2120	5.164319	4.225352	4.225352	7.981221	7.042254	7.042254	0.938967	6.103286	4.225352	2.347418	3.755869	10.79812	0	1.408451	3.755869	5.164319	5.633803	8.920188	4.694836	6.57277	R
SSA_0256	5.116279	3.255814	2.790698	3.255814	8.372093	6.046512	2.790698	7.906977	5.581395	3.255814	3.72093	13.02326	0	0.930233	5.581395	2.325581	4.186047	10.69767	5.581395	5.581395	K
SSA_0766	5.069124	1.382488	3.686636	6.451613	5.069124	4.147465	1.843318	8.75576	5.069124	0.921659	3.225806	20.2765	1.382488	0	5.069124	11.52074	4.147465	1.843318	1.382488	8.75576	
SSA_0894	4.910714	4.017857	4.017857	6.696429	8.482143	5.357143	3.571429	8.035714	5.803571	1.785714	4.017857	11.16071	0	1.339286	1.339286	4.017857	8.035714	7.142857	5.803571	4.464286	V
SSA_1814	4.867257	7.522124	9.292035	6.637168	3.539823	5.309735	2.212389	8.40708	2.212389	1.327434	2.654867	12.38938	3.539823	0.442478	6.19469	3.539823	3.982301	6.637168	5.752212	3.539823	
SSA_0410	4.782609	3.478261	2.608696	10	2.608696	4.347826	2.608696	10	3.913043	1.304348	2.173913	17.3913	1.304348	0.869565	3.043478	12.17391	6.086957	3.478261	1.73913	6.086957	
SSA_0845	4.680851	3.829787	5.957447	7.234043	5.531915	3.829787	1.702128	11.06383	7.234043	2.978723	5.531915	11.48936	0	0	5.531915	2.978723	5.957447	2.978723	6.808511	4.680851	V

SSA_1121	4.680851	1.702128	2.12766	6.382979	4.680851	4.255319	4.255319	10.21277	3.829787	1.702128	3.404255	17.02128	1.276596	0.851064	2.553191	10.21277	11.91489	1.702128	0.851064	6.382979	O
SSA_0605	4.64135	4.219409	5.485232	2.953586	8.860759	3.797468	1.687764	5.907173	5.485232	1.265823	5.485232	14.34599	0.421941	0.421941	3.375527	5.063291	4.64135	8.016878	5.485232	8.438819	M
SSA_1395	4.545455	4.132231	1.652893	6.198347	8.264463	5.371901	3.305785	6.61157	4.958678	1.652893	4.958678	11.57025	0.413223	0	4.132231	4.958678	3.719008	9.090909	4.132231	10.33058	S
SSA_1846	4.471545	4.471545	6.910569	4.878049	3.658537	6.504065	2.439024	9.349593	5.691057	2.03252	1.219512	9.349593	0.813008	0.406504	2.439024	2.03252	8.130081	8.130081	8.943089	8.130081	T
SSA_0348	4.435484	2.822581	1.209677	7.258065	3.629032	7.66129	1.209677	8.870968	3.629032	2.016129	4.83871	16.12903	4.435484	0.806452	2.822581	7.66129	8.064516	3.225806	0.806452	8.467742	
SSA_2152	4.365079	5.15873	5.555556	4.365079	7.142857	7.142857	3.571429	7.142857	2.380952	3.174603	3.174603	13.49206	0	0	1.984127	3.968254	7.539683	7.539683	5.15873	7.142857	R
SSA_0601	4.347826	5.13834	3.952569	4.347826	4.347826	5.533597	2.371542	8.300395	2.371542	1.976285	2.766798	9.486166	0.790514	2.371542	1.976285	5.533597	4.743083	10.27668	5.928854	13.43874	F
SSA_1162	4.347826	3.162055	3.557312	4.743083	4.743083	6.324111	2.766798	6.324111	1.976285	2.371542	3.557312	23.32016	2.371542	0.790514	5.533597	7.905138	5.13834	4.347826	1.581028	5.13834	
SSA_1418	4.198473	2.290076	3.053435	5.343511	7.633588	3.435115	1.526718	6.10687	4.961832	1.145038	5.725191	10.68702	2.290076	0	4.961832	5.343511	8.015267	6.48855	5.343511	11.45038	O
SSA_0584	4.11985	0.749064	3.745318	8.988764	10.86142	7.490637	3.745318	2.621723	5.617978	1.123596	1.123596	11.23596	0.749064	0.374532	3.745318	3.370787	7.865169	9.737828	8.2397	4.494382	
SSA_1406	3.846154	7.692308	2.097902	5.244755	4.895105	2.447552	1.398601	8.391608	7.342657	1.398601	3.846154	9.090909	2.447552	1.398601	7.342657	4.895105	5.594406	6.993007	8.391608	5.244755	M
SSA_1001	3.793103	3.793103	3.103448	8.275862	7.586207	4.827586	2.413793	5.172414	6.896552	2.758621	3.793103	10.34483	0.689655	1.034483	8.62069	5.862069	6.206897	5.862069	3.793103	5.172414	K
SSA_1370	3.536977	2.250804	3.536977	7.717042	7.073955	4.180064	2.572347	8.038585	2.893891	0.96463	4.180064	14.791	3.858521	1.286174	3.858521	8.360129	7.395498	3.858521	2.250804	7.395498	R
SSA_0447	3.503185	3.503185	6.050955	7.324841	5.095541	8.280255	4.458599	12.42038	3.184713	1.910828	2.547771	10.50955	0.636943	0.318471	3.503185	5.095541	1.910828	9.872611	3.821656	6.050955	P
SSA_1598	3.503185	2.547771	6.369427	6.687898	12.10191	4.77707	1.273885	8.917197	5.095541	1.273885	3.821656	8.280255	1.273885	0	6.369427	5.414013	5.414013	7.961783	6.687898	2.229299	
SSA_0931	3.395062	3.703704	4.62963	13.88889	6.790123	2.160494	0.925926	9.259259	4.62963	2.160494	4.012346	7.098765	1.54321	0	4.62963	6.790123	4.938272	8.641975	6.17284	4.62963	
SSA_1017	3.098592	3.380282	2.253521	6.760563	6.478873	3.661972	1.690141	5.915493	5.633803	2.816901	5.352113	11.83099	1.126761	0.84507	6.197183	7.042254	5.915493	7.605634	4.507042	7.887324	
SSA_1824	15.78947	2.631579	7.894737	2.631579	6.578947	3.947368	3.947368	9.210526	2.631579	0	5.263158	10.52632	0	0	1.315789	1.315789	9.210526	7.894737	5.263158	3.947368	
SSA_2150	15.18987	0	1.265823	10.12658	5.063291	2.531646	3.797468	11.39241	1.265823	0	2.531646	11.39241	2.531646	0	0	2.531646	18.98734	0	0	11.39241	S
SSA_0157	12.76596	1.06383	3.191489	5.319149	4.255319	5.319149	5.319149	7.446809	6.382979	1.06383	3.191489	7.446809	1.06383	0	1.06383	7.446809	10.6383	1.06383	2.12766	13.82979	
SSA_0320	10.71429	2.678571	1.785714	6.25	7.142857	5.357143	1.785714	9.821429	0.892857	0	6.25	19.64286	1.785714	0	5.357143	7.142857	4.464286	0.892857	0.892857	7.142857	S
SSA_2207	10.25641	5.128205	4.273504	7.692308	3.418803	1.709402	0.854701	3.418803	5.128205	0.854701	2.564103	29.91453	0	0	0	0	2.564103	8.547009	6.837607	6.837607	
SSA_0130	9.917355	12.39669	4.958678	3.305785	9.917355	4.132231	1.652893	9.917355	1.652893	0.826446	2.479339	6.61157	0	0	2.479339	0	9.090909	5.785124	5.785124	9.090909	J
SSA_0809	9.52381	1.587302	3.174603	3.968254	6.349206	7.936508	0.793651	10.31746	3.968254	1.587302	4.761905	6.349206	0	0.793651	1.587302	5.555556	5.555556	8.730159	4.761905	12.69841	J
SSA_1953	8.275862	2.068966	3.448276	7.586207	8.965517	4.827586	2.758621	4.137931	4.827586	3.448276	2.068966	8.965517	0.689655	2.068966	0.689655	3.448276	6.206897	6.896552	7.586207	11.03448	C
SSA_1486	8.219178	3.424658	8.219178	4.109589	7.534247	8.90411	1.369863	8.90411	4.109589	0	0.684932	14.38356	0.684932	0	2.739726	10.9589	3.424658	2.054795	2.739726	7.534247	S
SSA_0251	8.163265	5.442177	4.761905	6.122449	6.122449	4.081633	4.081633	8.843537	6.802721	0.680272	4.081633	8.163265	0	0	1.360544	4.081633	6.802721	4.081633	8.843537	7.482993	J
SSA_0686	8.163265	3.401361	4.081633	2.721088	5.442177	6.802721	3.401361	6.802721	8.163265	6.122449	3.401361	6.802721	0	2.721088	4.081633	3.401361	4.761905	7.482993	6.802721	5.442177	P
SSA_1352	8.108108	3.378378	5.405405	6.081081	5.405405	2.027027	2.027027	8.108108	0.675676	3.378378	2.702703	15.54054	5.405405	0.675676	3.378378	14.18919	2.702703	3.378378	3.378378	4.054054	
SSA_0531	8.053691	6.040268	0.671141	4.697987	5.369128	6.711409	2.684564	6.040268	2.013423	1.342282	4.697987	11.4094	1.342282	0	3.355705	2.684564	8.724832	6.711409	11.4094	6.040268	E
SSA_0317	8	5.333333	2	5.333333	6.666667	3.333333	2.666667	4.666667	4	1.333333	3.333333	10.66667	2	0.666667	3.333333	4.666667	1.333333	12.66667	5.333333	12.66667	R
SSA_1818	7.94702	8.609272	4.635762	3.97351	13.24503	3.311258	2.649007	6.622517	2.649007	1.986755	2.649007	5.298013	3.97351	1.986755	1.986755	3.311258	5.298013	9.271523	6.622517	3.97351	L
SSA_1236	7.792208	2.597403	2.597403	1.298701	7.792208	7.142857	1.298701	3.896104	2.597403	1.948052	5.194805	9.74026	3.246753	1.948052	3.896104	6.493506	7.792208	11.03896	7.792208	3.896104	F
SSA_2113	7.792208	2.597403	1.948052	11.03896	5.844156	4.545455	1.948052	5.844156	11.03896	0.649351	2.597403	8.441558	0.649351	0	0	2.597403	3.246753	10.38961	2.597403	16.23377	
SSA_0755	7.453416	4.347826	4.347826	9.31677	7.453416	6.21118	1.242236	7.453416	5.590062	1.242236	1.242236	10.55901	0.621118	0.621118	5.590062	7.453416	4.968944	5.590062	7.453416	1.242236	S
SSA_0039	7.407407	3.703704	1.234568	6.790123	5.555556	5.555556	4.320988	8.641975	3.08642	1.851852	4.320988	8.024691	0.617284	0	1.234568	1.851852	8.641975	7.407407	3.703704	16.04938	F
SSA_0903	7.361963	3.680982	4.294479	6.748466	5.521472	1.840491	1.840491	3.067485	7.361963	2.453988	2.453988	13.49693	1.840491	1.226994	4.907975	6.134969	6.134969	9.202454	5.521472	4.907975	R
SSA_1789	7.361963	7.97546	3.680982	5.521472	12.26994	4.294479	3.067485	4.907975	0.613497	6.134969	4.907975	7.361963	3.680982	0	4.907975	3.680982	3.067485	6.134969	3.067485	7.361963	R
SSA_0324	7.317073	4.878049	3.04878	3.658537	12.80488	4.268293	1.829268	6.097561	7.926829	0	1.829268	10.97561	0.609756	0	1.219512	1.829268	3.04878	15.2439	6.097561	7.317073	
SSA_0731	7.100592	3.550296	4.733728	4.142012	4.733728	4.142012	2.95858	10.65089	0.591716	1.183432	6.508876	17.75148	2.95858	0	5.325444	7.692308	3.550296	3.550296	0.591716	8.284024	
SSA_0644	7.017544	2.339181	2.923977	3.508772	6.432749	9.356725	3.508772	5.847953	4.678363	2.923977	2.339181	9.356725	1.754386	0	3.508772	3.508772	6.432749	8.77193	6.432749	9.356725	P
SSA_1485	7.017544	2.923977	4.093567	5.847953	4.093567	5.847953	4.093567	11.69591	3.508772	1.169591	1.754386	11.11111	0.584795	0.584795	5.847953	9.356725	7.017544	2.339181	1.754386	9.356725	S
SSA_1832	6.779661	6.214689	6.779661	3.389831	8.474576	5.084746	2.259887	7.344633	1.129944	3.389831	2.259887	7.909605	2.824859	0.564972	7.909605	3.389831	4.519774	6.214689	9.60452	3.954802	J
SSA_0876	6.593407	4.395604	2.197802	3.846154	4.945055	5.494505	3.296703	5.494505	4.395604	1.098901	2.747253	11.53846	0.549451	1.648352	3.296703	3.846154	8.791209	3.296703	10.43956	12.08791	R
SSA_1769	6.557377	3.278689	3.825137	1.639344	4.918033	6.557377	2.73224	6.010929	7.103825	2.73224	3.278689	11.47541	0	0.546448	3.278689	4.371585	7.103825	6.010929	6.557377	12.02186	J
SSA_0567	6.486486	3.783784	3.783784	5.405405	4.864865	5.945946	1.081081	8.108108	3.243243	4.324324	3.243243	8.108108	0.540541	0.540541	3.783784	5.405405	5.405405	4.324324	9.72973	11.89189	Q
SSA_1689	6.451613	1.612903	5.376344	18.27957	10.75269	5.376344	1.612903	3.225806	3.763441	0.537634	2.688172	8.602151	0	0.537634	2.688172	3.225806	2.150538	9.139785	5.913978	8.064516	
SSA_1821	6.417112	8.55615	2.673797	3.208556	4.812834	4.278075	0.534759	6.417112	2.139037	3.208556	4.812834	11.22995	1.069519	1.069519	5.347594	2.673797	6.951872	10.16043	6.951872	7.486631	J
SSA_1778	6.349206	4.232804	3.174603	6.878307	5.291005	5.820106	1.058201	7.936508	3.703704	1.058201	4.232804	14.28571	0	0	3.703704	2.645503	4.232804	12.16931	5.291005	7.936508	K
SSA_0482	6.282723	10.99476	2.094241	4.188482	4.712042	4.188482	3.664921	7.329843	1.570681	3.141361	4.188482	9.424084	0.52356	1.570681	5.759162	3.141361	5.235602	9.424084	7.329843	5.235602	S
SSA_1122	6.282723	1.04712	0.52356	8.900524	9.947644	7.853403	3.141361	4.712042	6.282723	0	5.235602	6.282723	1.570681	1.570681	2.094241	5.759162	6.282723	8.376963	4.712042	9.424084	OC
SSA_2308	6.217617	4.663212	8.290155	5.699482	9.326425	4.663212	2.590674	7.772021	3.108808	2.590674	1.554404	8.290155	0	0	1.554404	7.772021	2.590674	9.326425	6.735751	7.253886	
SSA_1445	6.185567	4.639175	4.639175	5.154639	5.154639	6.185567	2.57732	5.154639	4.123711	4.639175	2.061856	12.37113	0.515464	0	1.030928	5.670103	7.731959	8.247423	5.670103	8.247423	E
SSA_1154	6.122449	4.591837	4.591837	6.122449	5.102041	5.612245	2.040816	8.163265	5.612245	0.510204	5.102041	11.22449	0.510204	0	3.571429	3.571429	7.142857	7.142857	5.102041	8.163265	J
SSA_0947	6.091371	1.522843	5.076142	7.106599	11.16751	5.583756	1.522843	9.137056	3.045685	0	3.553299	9.64467	0.507614	0	5.583756	3.553299	6.598985	9.137056	7.106599	4.060914	
SSA_2125	6.060606	8.585859	3.030303	4.040404	6.060606	4.545455	3.030303	5.555556	5.050505	1.515152	2.020202	11.61616	0	0.505051	3.535354	3.535354	8.080808	10.10101	7.575758	5.555556	L
SSA_0513	6.030151	6.532663	4.522613	4.020101	6.030151	6.532663	1.507538	8.542714	4.522613	2.01005	2.01005	8.542714	2.01005	0.502513	4.522613	3.517588	6.030151	6.532663	8.040201	8.040201	S
SSA_0422	6	5.5	1.5	4	4	5	2.5	2.5	6.5	2.5	4.5	10	1	2	4.5	5.5	10	9.5	4.5	8.5	G
SSA_0165	5.970149	5.472637	6.965174	9.452736	7.960199	6.965174	3.482587	5.970149	3.482587	1.492537	2.985075	6.965174	0	0.995025	4.477612	2.985075	4.975124	7.462687	4.477612	7.462687	
SSA_1125	5.970149	3.9801	4.975124	6.467662	7.960199	2.985075	2.487562	7.960199	3.482587	1.99005	4.477612	13.43284	0.995025	0	2.487562	3.9801	3.9801	6.467662	7.462687	8.457711	R
SSA_1976	5.940594	3.465347	3.465347	16.83168	8.415842	6.930693	2.475248	7.425743	2.970297	1.980198	2.475248	9.405941	0	0.49505	1.485149	3.960396	3.960396	9.405941	3.960396	4.950495	
SSA_1198	5.91133	3.940887	4.433498	9.852217	9.359606	4.926108	2.463054	6.403941	2.955665	1.477833	1.477833	11.82266	0.985222	0.985222	3.448276	2.463054	5.418719	8.866995	7.389163	5.418719	
SSA_1735	5.91133	3.940887	5.418719	7.389163	4.926108	3.448276	1.970443	6.896552	6.403941	3.940887	3.448276	11.33005	4.433498	0	1.970443	4.926108	3.940887	9.359606	4.926108	5.418719	O

SSA_0993	5.882353	2.941176	2.45098	6.862745	9.313725	3.431373	2.941176	8.333333	3.921569	1.960784	3.431373	14.21569	2.941176	0.490196	5.392157	4.901961	4.411765	5.392157	5.392157	5.392157	H
SSA_1795	5.825243	8.252427	2.912621	4.368932	7.281553	2.912621	2.427184	9.708738	6.796117	2.427184	2.912621	7.281553	0.485437	2.912621	4.854369	1.941748	4.368932	10.67961	5.825243	5.825243	S
SSA_1315	5.607477	3.271028	4.205607	13.5514	8.411215	7.943925	2.803738	6.542056	2.336449	0.46729	0.934579	5.607477	2.336449	0.46729	5.140187	3.738318	7.476636	5.607477	9.345794	4.205607	
SSA_1199	5.581395	4.651163	2.790698	4.186047	6.976744	4.186047	3.255814	5.581395	4.186047	1.860465	3.255814	9.767442	2.325581	0.465116	7.906977	4.186047	6.046512	6.511628	5.581395	10.69767	M
SSA_1569	5.479452	3.652968	3.652968	7.305936	4.109589	6.392694	1.826484	13.69863	5.936073	0	2.283105	14.61187	1.369863	0	2.739726	8.219178	5.022831	3.652968	0.913242	9.13242	E
SSA_1643	5.479452	3.196347	1.369863	6.849315	11.41553	6.849315	3.196347	4.109589	2.283105	2.283105	1.369863	7.762557	0	0.456621	7.305936	4.56621	4.109589	8.675799	12.32877	6.392694	
SSA_1323	5.454545	5.909091	3.181818	10.45455	5.909091	3.181818	2.272727	5.454545	4.545455	2.727273	3.181818	14.54545	1.363636	1.818182	3.636364	3.636364	4.545455	6.363636	7.272727	4.545455	
SSA_0881	5.429864	4.072398	3.167421	6.78733	9.954751	7.692308	2.262443	10.85973	3.61991	2.262443	2.262443	7.692308	0.904977	0.452489	5.429864	4.524887	5.429864	8.144796	7.239819	1.809955	
SSA_0925	5.429864	4.977376	1.357466	5.429864	8.597285	7.239819	1.357466	6.334842	6.78733	2.714932	3.167421	14.93213	0	0	1.809955	3.61991	6.78733	6.78733	6.78733	5.882353	V
SSA_0286	5.405405	3.153153	4.504505	4.504505	7.207207	5.405405	1.801802	10.81081	3.153153	1.351351	4.054054	6.306306	0.900901	0.45045	3.603604	4.054054	5.855856	6.756757	5.405405	15.31532	G
SSA_0446	5.357143	4.017857	3.571429	8.482143	6.696429	7.142857	2.678571	7.589286	3.571429	1.339286	4.464286	14.28571	1.339286	0	4.017857	4.910714	5.803571	4.017857	4.017857	6.696429	S
SSA_1218	5.286344	6.167401	3.964758	9.69163	4.845815	3.964758	5.286344	9.251101	7.929515	3.0837	2.202643	11.89427	0	0.881057	3.0837	1.762115	4.405286	8.370044	3.524229	4.405286	L
SSA_1650	5.172414	2.155172	3.017241	4.741379	6.465517	7.327586	3.017241	6.465517	3.017241	1.293103	3.448276	11.2069	1.724138	0.862069	2.586207	4.310345	8.62069	5.603448	7.327586	11.63793	IQR
SSA_0201	5.106383	5.531915	1.702128	7.234043	5.106383	5.957447	2.12766	7.659574	4.680851	2.978723	2.978723	13.19149	0.425532	0.425532	1.276596	3.829787	9.787234	5.957447	7.234043	6.808511	V
SSA_0649	4.979253	4.564315	3.319502	6.639004	9.128631	4.979253	1.659751	6.224066	7.053942	2.489627	3.319502	13.69295	0	0.414938	4.149378	3.319502	6.639004	5.809129	6.224066	5.394191	J
SSA_1883	4.958678	1.239669	6.198347	10.7438	13.63636	2.066116	1.239669	7.024793	6.61157	1.652893	2.066116	8.264463	0.413223	0.826446	4.545455	8.264463	4.545455	7.024793	3.719008	4.958678	
SSA_1780	4.938272	6.17284	3.292181	7.407407	7.407407	2.880658	1.646091	7.407407	8.641975	0.823045	2.057613	13.99177	0.823045	0	4.526749	4.938272	4.115226	9.876543	3.703704	5.349794	L
SSA_1277	4.878049	2.03252	6.504065	11.78862	7.317073	3.252033	1.219512	3.658537	6.097561	1.219512	2.03252	8.130081	0.813008	0.406504	8.536585	2.03252	9.349593	7.317073	4.878049	8.536585	M
SSA_0850	4.8583	2.42915	6.477733	7.692308	11.33603	7.287449	1.214575	7.287449	2.024291	2.024291	1.619433	10.93117	1.214575	0.404858	5.263158	3.643725	3.643725	8.906883	8.097166	3.643725	
SSA_0991	4.743083	2.766798	8.300395	4.347826	7.509881	5.533597	2.766798	3.952569	4.743083	2.766798	3.162055	11.85771	2.371542	0	5.13834	2.371542	7.905138	7.114625	5.928854	6.719368	
SSA_2204	4.724409	2.362205	5.511811	16.14173	8.661417	8.661417	2.755906	5.11811	5.11811	0.787402	4.330709	5.511811	0.393701	0.787402	3.543307	4.330709	3.149606	4.330709	7.874016	5.905512	
SSA_1325	4.6875	8.203125	2.734375	5.859375	3.515625	1.953125	2.34375	6.25	7.421875	0.390625	3.515625	17.57813	1.5625	1.953125	3.515625	1.171875	5.859375	5.078125	3.90625	12.5	R
SSA_0025	4.669261	3.891051	5.058366	5.836576	7.003891	3.11284	1.945525	5.836576	4.669261	3.11284	3.501946	13.61868	0.389105	1.55642	4.280156	7.392996	4.280156	5.058366	8.949416	5.836576	L
SSA_2145	4.633205	4.247104	6.177606	7.722008	6.949807	3.088803	3.088803	3.474903	2.316602	1.930502	4.247104	10.42471	3.861004	0.3861	6.177606	3.861004	5.791506	7.722008	5.791506	8.108108	R
SSA_1741	4.597701	4.214559	2.298851	6.51341	4.597701	7.662835	3.065134	10.34483	7.662835	1.915709	2.681992	11.49425	1.149425	1.532567	2.298851	2.298851	6.130268	4.980843	7.279693	7.279693	PH
SSA_0503	4.562738	4.562738	1.901141	7.984791	6.463878	4.942966	4.562738	7.224335	6.844106	2.281369	3.802281	14.44867	0.760456	0.760456	2.661597	1.901141	7.224335	9.125475	2.281369	5.703422	EP
SSA_1563	4.511278	3.759398	3.759398	6.766917	7.142857	6.390977	3.759398	6.390977	3.007519	4.511278	1.879699	9.774436	0.75188	0.75188	3.759398	3.383459	7.894737	5.639098	9.022556	7.142857	R
SSA_0594	4.494382	5.617978	2.621723	8.988764	7.490637	4.868914	3.370787	4.494382	4.494382	2.621723	5.243446	11.23596	0.374532	1.123596	5.243446	4.494382	5.243446	8.614232	4.494382	4.868914	K
SSA_1717	4.494382	4.494382	5.617978	5.617978	10.11236	4.868914	2.996255	7.116105	1.872659	2.247191	4.494382	8.614232	2.621723	0.374532	5.243446	3.745318	7.865169	6.367041	6.367041	4.868914	L
SSA_1629	4.411765	1.470588	5.514706	11.02941	10.66176	5.882353	2.205882	6.25	2.941176	0.735294	3.308824	6.985294	0.367647	0.367647	3.308824	4.044118	9.191176	7.720588	7.352941	6.25	O
SSA_1187	4.285714	5.714286	4.285714	4.285714	8.214286	4.285714	5.714286	6.785714	3.214286	1.785714	1.785714	13.21429	0.357143	1.071429	3.571429	4.642857	3.214286	11.42857	6.428571	5.714286	K
SSA_0081	4.240283	5.300353	4.240283	7.067138	6.007067	4.59364	2.826855	8.480565	8.127208	2.120141	2.120141	10.60071	0.706714	1.060071	3.180212	6.007067	4.59364	7.067138	5.65371	6.007067	K
SSA_1480	4.240283	2.826855	5.300353	7.420495	5.65371	5.300353	3.533569	6.713781	5.65371	4.59364	6.360424	8.127208	1.060071	1.060071	4.946996	6.007067	6.360424	3.886926	5.65371	5.300353	R
SSA_1888	3.858521	2.893891	5.787781	7.073955	12.21865	2.250804	0.643087	7.717042	3.215434	2.250804	2.572347	10.61093	0.643087	0.96463	5.144695	8.360129	3.858521	7.073955	9.003215	3.858521	
SSA_1096	3.809524	3.809524	4.126984	6.666667	4.126984	4.761905	0.31746	5.714286	6.666667	1.269841	5.079365	13.01587	1.269841	1.269841	3.809524	3.174603	7.301587	9.52381	5.079365	9.206349	E
SSA_1365	3.809524	2.539683	1.904762	6.031746	8.888889	7.619048	2.857143	6.349206	5.396825	1.587302	3.809524	10.79365	1.269841	0.31746	5.079365	4.126984	6.984127	6.666667	6.984127	6.984127	V
SSA_2339	3.785489	2.208202	3.154574	7.570978	5.362776	7.255521	2.208202	6.309148	2.839117	3.154574	5.047319	11.35647	1.26183	0.946372	5.047319	4.731861	5.362776	7.570978	6.309148	8.51735	V
SSA_1649	3.680982	2.760736	3.680982	5.828221	10.7362	5.214724	3.067485	5.214724	2.453988	2.147239	3.374233	11.34969	2.453988	0.613497	3.98773	5.214724	7.055215	9.202454	5.828221	6.134969	C
SSA_2338	3.468208	1.156069	7.514451	17.63006	6.936416	7.225434	0.578035	6.936416	3.468208	0.867052	1.445087	12.71676	0	0.289017	0.867052	3.179191	9.537572	4.624277	5.491329	6.069364	
SSA_0258	3.287671	7.671233	4.931507	7.123288	6.027397	3.013699	1.09589	4.657534	9.041096	1.643836	4.931507	8.493151	2.465753	0.273973	3.835616	6.575342	5.205479	5.753425	4.931507	9.041096	
SSA_0861	2.919708	3.406326	3.406326	6.326034	8.515815	6.082725	2.43309	4.622871	8.759124	0.729927	3.892944	10.46229	0.243309	0.243309	5.839416	7.055961	6.569343	6.326034	6.569343	5.596107	V
SSA_0529	13.97849	3.225806	5.376344	6.451613	6.451613	3.225806	4.301075	5.376344	3.225806	2.150538	2.150538	8.602151	1.075269	1.075269	2.150538	2.150538	7.526882	6.451613	8.602151	6.451613	QC
SSA_0186	12.38095	0	5.714286	8.571429	11.42857	6.666667	1.904762	2.857143	5.714286	0.952381	0.952381	13.33333	0	0	3.809524	2.857143	3.809524	5.714286	2.857143	10.47619	U
SSA_1190	11.60714	7.142857	1.785714	4.464286	5.357143	4.464286	3.571429	7.142857	7.142857	3.571429	0.892857	8.928571	0	0	5.357143	1.785714	4.464286	8.035714	6.25	8.035714	F
SSA_2033	10	10.76923	3.076923	5.384615	9.230769	4.615385	0.769231	3.846154	4.615385	1.538462	3.846154	6.923077	0	0	3.076923	3.076923	10	2.307692	5.384615	11.53846	J
SSA_0057	9.701493	1.492537	3.731343	5.223881	5.223881	4.477612	4.477612	5.223881	1.492537	1.492537	4.477612	10.44776	0	1.492537	2.238806	5.223881	6.716418	11.9403	7.462687	7.462687	G
SSA_2383	9.558824	3.676471	6.617647	8.088235	8.823529	4.411765	2.941176	7.352941	4.411765	1.470588	3.676471	10.29412	0.735294	0.735294	4.411765	6.617647	1.470588	5.882353	2.941176	5.882353	R
SSA_0254	9.219858	5.673759	2.12766	4.255319	7.801418	2.12766	2.836879	2.836879	9.219858	2.836879	4.964539	6.382979	1.41844	2.12766	7.092199	7.092199	7.092199	5.673759	4.964539	4.255319	
SSA_0101	8.965517	8.275862	2.758621	6.206897	4.827586	4.827586	2.068966	7.586207	4.137931	2.068966	4.137931	7.586207	0.689655	0.689655	3.448276	4.137931	4.827586	7.586207	8.275862	6.896552	R
SSA_0151	8.965517	6.206897	1.37931	6.896552	3.448276	2.068966	2.758621	15.17241	4.137931	0.689655	1.37931	13.7931	0.689655	0	6.896552	5.517241	7.586207	5.517241	0.689655	6.206897	
SSA_2161	8.724832	7.38255	4.697987	6.040268	4.026846	6.040268	2.684564	6.040268	2.684564	0.671141	1.342282	7.38255	1.342282	2.013423	6.040268	6.711409	6.040268	11.4094	7.38255	1.342282	F
SSA_1330	8.387097	6.451613	4.516129	7.741935	7.096774	1.935484	2.580645	5.16129	1.935484	0.645161	3.870968	8.387097	0	0	9.677419	3.870968	7.096774	7.741935	9.032258	3.870968	
SSA_1807	8.280255	10.82803	3.821656	9.55414	9.55414	4.458599	1.273885	7.006369	4.458599	0.636943	1.273885	4.458599	0	2.547771	2.547771	3.184713	5.095541	12.10191	6.369427	2.547771	K
SSA_1544	8.125	0.625	2.5	7.5	11.875	6.875	0.625	5.625	5	1.25	1.875	2.5	0	0	1.875	5.625	4.375	19.375	6.25	8.125	
SSA_1384	8.024691	3.08642	3.08642	10.49383	9.259259	2.469136	1.851852	6.790123	6.17284	0	1.851852	8.024691	1.234568	0	6.17284	4.938272	8.024691	6.790123	3.703704	8.024691	S
SSA_0299	7.784431	4.191617	4.191617	4.790419	2.994012	6.586826	2.994012	7.185629	3.592814	0.598802	3.592814	11.97605	1.197605	0.598802	3.592814	3.592814	5.988024	7.784431	1.197605	15.56886	S
SSA_1349	7.602339	7.017544	2.923977	5.263158	2.923977	5.263158	1.169591	4.093567	9.356725	4.093567	1.754386	14.03509	0	1.169591	4.093567	2.923977	4.678363	6.432749	7.017544	8.187135	R
SSA_2036	7.514451	7.514451	5.202312	5.202312	5.780347	5.780347	3.468208	2.890173	5.780347	0	3.468208	10.40462	1.156069	0.578035	3.468208	4.624277	3.468208	9.248555	6.358382	8.092486	R
SSA_0014	7.065217	2.173913	4.891304	3.804348	8.695652	5.434783	2.173913	12.5	3.26087	2.173913	2.717391	8.695652	0	1.086957	4.347826	3.804348	7.065217	6.521739	9.23913	4.347826	F
SSA_0849	7.027027	6.486486	4.324324	7.027027	9.189189	3.783784	2.702703	8.108108	3.243243	0.540541	1.081081	10.27027	0	0	6.486486	4.324324	5.945946	5.945946	8.648649	4.864865	U
SSA_1929	6.989247	4.301075	8.064516	2.688172	3.225806	10.21505	1.612903	6.451613	2.688172	1.612903	4.83871	10.21505	0	1.612903	3.225806	3.225806	5.376344	6.989247	4.83871	11.82796	S
SSA_0288	6.951872	3.743316	6.417112	8.55615	4.278075	5.882353	1.069519	12.29947	3.208556	1.604278	3.208556	12.83422	1.069519	1.069519	6.417112	5.882353	6.417112	2.139037	2.673797	4.278075	O
SSA_0491	6.842105	5.789474	1.578947	3.684211	2.631579	5.263158	2.105263	3.684211	9.473684	2.105263	3.684211	10.52632	3.684211	1.578947	3.684211	4.210526	5.789474	7.368421	7.894737	8.421053	G

SSA_0877	6.806283	3.141361	3.664921	4.712042	6.282723	4.712042	2.094241	4.712042	5.235602	2.094241	3.141361	10.4712	0.52356	0	4.712042	6.282723	6.806283	6.806283	8.900524	8.900524	R
SSA_1380	6.598985	3.553299	2.030457	6.091371	6.598985	6.598985	2.030457	6.598985	5.076142	3.045685	3.045685	9.137056	2.030457	0.507614	3.045685	5.583756	6.598985	10.15228	6.091371	5.583756	
SSA_0690	6.565657	5.050505	4.040404	7.575758	4.040404	7.070707	3.030303	9.090909	2.020202	1.515152	3.535354	10.10101	0	2.020202	3.535354	1.515152	6.060606	6.565657	8.080808	8.585859	L
SSA_2220	6.467662	4.975124	2.487562	3.9801	9.452736	4.975124	2.487562	7.462687	2.985075	2.487562	4.975124	11.9403	1.99005	0.497512	2.985075	6.467662	8.955224	4.975124	6.467662	2.985075	M
SSA_2249	6.280193	2.415459	5.797101	6.280193	11.5942	6.280193	1.449275	12.07729	2.415459	0.966184	1.449275	10.14493	0.483092	0	4.830918	1.932367	7.246377	6.280193	6.280193	5.797101	V
SSA_0351	6.220096	3.349282	2.870813	5.741627	10.04785	6.220096	1.435407	6.220096	3.827751	0.956938	3.827751	10.52632	1.435407	0	4.30622	7.177033	6.698565	4.784689	10.04785	4.30622	U
SSA_1240	6.220096	2.870813	3.349282	3.349282	7.655502	6.220096	1.435407	8.61244	2.392344	1.435407	5.263158	8.133971	0.956938	0	3.349282	3.349282	7.655502	9.569378	4.784689	13.39713	F
SSA_1391	6.190476	5.238095	4.761905	6.190476	9.047619	3.809524	3.809524	8.095238	2.857143	1.904762	1.904762	11.42857	2.380952	0.47619	7.142857	2.857143	3.809524	8.571429	3.333333	6.190476	
SSA_0872	6.132075	3.301887	3.773585	5.660377	4.716981	6.603774	1.415094	6.132075	1.415094	5.188679	5.660377	12.26415	0.943396	0.943396	4.716981	4.245283	6.132075	7.54717	6.603774	6.603774	R
SSA_0387	6.103286	3.755869	7.042254	8.450704	6.57277	4.225352	1.408451	7.511737	7.511737	1.877934	3.286385	9.859155	0.469484	0	3.286385	5.164319	5.164319	7.511737	3.286385	7.511737	P
SSA_0526	6.103286	5.633803	3.755869	5.633803	5.633803	5.633803	1.877934	6.57277	7.981221	1.877934	2.347418	11.73709	0.938967	2.347418	3.286385	3.286385	3.286385	7.981221	7.042254	7.042254	E
SSA_1884	5.936073	5.022831	9.589041	7.762557	5.936073	4.109589	0.913242	10.9589	3.196347	1.826484	5.022831	6.849315	1.826484	2.739726	4.56621	5.022831	3.652968	5.936073	6.849315	2.283105	
SSA_0397	5.882353	4.977376	4.072398	4.977376	3.61991	4.524887	4.072398	12.66968	1.809955	2.262443	5.429864	11.76471	2.262443	1.809955	6.78733	7.692308	5.429864	3.61991	1.809955	4.524887	
SSA_0880	5.777778	2.222222	2.666667	8	11.55556	7.111111	1.333333	8.888889	2.222222	0.444444	2.666667	9.333333	0.444444	0	8.444444	4	4.888889	9.777778	7.555556	2.666667	
SSA_0069	5.752212	3.097345	3.097345	9.292035	3.982301	3.982301	1.769912	7.079646	1.327434	0.884956	2.654867	23.45133	2.212389	0.884956	4.424779	4.424779	11.06195	2.212389	1.769912	6.637168	
SSA_1217	5.652174	3.043478	3.043478	6.086957	4.782609	5.652174	2.173913	10.86957	4.347826	3.478261	4.347826	7.391304	0.869565	0.869565	3.913043	4.347826	8.695652	7.826087	7.391304	5.217391	R
SSA_2065	5.652174	3.478261	3.913043	4.782609	8.695652	5.652174	1.73913	5.652174	3.043478	3.478261	3.913043	11.73913	2.608696	1.73913	3.478261	6.521739	6.521739	5.652174	7.826087	3.913043	H
SSA_2259	5.627706	4.329004	5.627706	6.493506	9.090909	4.761905	3.030303	7.359307	3.463203	0.4329	2.164502	7.359307	0.4329	3.463203	3.896104	6.926407	6.926407	6.926407	4.329004	7.359307	S
SSA_0042	5.603448	1.293103	1.724138	4.310345	7.758621	7.758621	1.724138	10.77586	6.034483	2.155172	1.724138	11.2069	1.293103	1.293103	3.87931	1.293103	7.327586	7.327586	6.465517	9.051724	M
SSA_1561	5.603448	5.172414	3.448276	6.465517	8.189655	3.87931	1.293103	4.310345	4.741379	1.724138	1.724138	12.5	0	0.862069	3.017241	6.034483	7.327586	10.77586	5.172414	7.758621	K
SSA_0765	5.579399	3.433476	5.150215	5.579399	5.150215	3.433476	3.433476	9.871245	3.433476	1.716738	3.433476	16.7382	0.858369	0	3.004292	9.871245	6.008584	2.575107	1.287554	9.44206	R
SSA_1794	5.555556	3.846154	2.564103	7.264957	5.555556	6.410256	5.128205	5.982906	4.273504	2.564103	1.709402	12.82051	0.854701	0	2.136752	2.991453	5.982906	9.401709	7.264957	7.692308	TK
SSA_0746	5.531915	1.276596	4.680851	6.808511	6.808511	5.106383	2.12766	6.382979	5.957447	2.553191	4.255319	10.6383	0	0	2.553191	4.680851	7.659574	8.93617	5.106383	8.93617	G
SSA_0442	5.485232	3.375527	2.531646	6.329114	8.860759	2.953586	2.531646	7.594937	3.797468	2.953586	2.953586	12.23629	0	1.265823	3.797468	4.64135	6.751055	7.594937	6.329114	8.016878	V
SSA_0486	5.394191	2.074689	0.829876	8.713693	5.809129	4.979253	1.244813	6.639004	8.713693	2.904564	6.224066	8.713693	2.904564	1.244813	2.904564	3.73444	3.73444	7.883817	4.149378	11.20332	H
SSA_0331	5.327869	4.098361	4.918033	4.918033	7.377049	4.098361	1.639344	5.327869	5.737705	1.639344	3.688525	12.29508	1.229508	0	1.229508	6.147541	3.688525	12.70492	4.098361	9.836066	
SSA_1119	5.327869	3.278689	2.459016	4.508197	9.016393	2.868852	2.868852	6.557377	7.786885	1.639344	2.868852	12.70492	0.409836	0.409836	4.098361	4.918033	4.918033	8.606557	7.377049	7.377049	T
SSA_1064	5.284553	5.284553	6.097561	5.691057	7.317073	6.504065	1.219512	6.504065	2.439024	0.813008	4.878049	4.065041	0.813008	0	10.1626	5.284553	7.317073	7.723577	6.910569	5.691057	R
SSA_2198	5.263158	6.072874	2.42915	5.668016	5.263158	1.619433	1.214575	6.882591	4.453441	2.024291	4.048583	11.74089	2.834008	1.619433	3.238866	2.42915	3.643725	10.12146	8.502024	10.93117	
SSA_0153	5.241935	4.83871	2.016129	8.467742	7.258065	3.629032	1.612903	6.451613	6.048387	0.806452	2.016129	13.70968	1.612903	2.016129	4.435484	7.258065	4.83871	4.032258	6.048387	7.66129	
SSA_2002	5.220884	7.630522	3.212851	4.016064	5.62249	6.827309	2.008032	6.827309	4.016064	4.016064	4.417671	7.630522	0	0	4.417671	5.62249	8.032129	8.433735	6.024096	6.024096	J
SSA_1238	5.2	4.4	2.8	5.2	7.6	4.4	2	8.4	4.4	2.8	7.2	9.6	2.8	1.2	3.2	4	4	8	4.4	8.4	
SSA_1958	5.2	3.2	1.2	6.8	7.6	4.8	3.6	5.2	3.6	1.2	3.2	11.6	0.4	0.4	4	5.2	2	12.8	10	8	OTN
SSA_1257	5.13834	7.114625	4.743083	8.300395	3.557312	2.371542	2.371542	6.719368	5.533597	3.162055	2.766798	13.04348	0.395257	0.395257	3.557312	5.533597	1.581028	9.486166	6.324111	7.905138	
SSA_0202	4.961832	2.290076	3.053435	8.396947	3.435115	6.10687	4.580153	4.198473	2.671756	1.526718	4.198473	22.1374	2.290076	1.145038	3.435115	4.198473	8.015267	3.435115	1.526718	8.396947	
SSA_1763	4.924242	5.681818	1.136364	6.060606	6.439394	5.30303	2.651515	8.333333	5.681818	1.893939	3.030303	14.01515	1.136364	0	2.651515	2.272727	8.333333	9.848485	4.924242	5.681818	P
SSA_1364	4.797048	4.059041	4.797048	5.904059	5.166052	3.690037	2.583026	7.380074	5.166052	1.476015	3.321033	13.28413	2.95203	0.369004	4.797048	7.380074	9.9631	2.95203	2.95203	7.01107	
SSA_1889	4.659498	5.017921	3.584229	7.885305	10.39427	10.75269	1.792115	3.942652	2.508961	2.508961	3.225806	9.318996	0.358423	0	3.584229	4.301075	2.867384	8.602151	4.659498	10.03584	
SSA_1891	4.626335	4.626335	5.338078	3.202847	4.626335	3.914591	1.779359	5.69395	3.558719	2.135231	5.338078	11.3879	2.491103	0.711744	3.558719	3.558719	7.829181	8.540925	5.338078	11.74377	R
SSA_1587	4.609929	7.446809	3.191489	4.964539	2.48227	3.546099	1.06383	7.446809	5.319149	5.319149	3.191489	14.53901	1.41844	0.70922	3.546099	2.836879	6.028369	8.865248	7.092199	6.382979	R
SSA_0710	4.513889	2.777778	5.902778	6.597222	5.208333	4.513889	1.736111	11.11111	6.25	4.513889	3.819444	7.291667	0.694444	1.041667	2.777778	5.555556	5.208333	5.902778	7.638889	6.944444	R
SSA_1792	4.234528	3.908795	3.583062	6.514658	8.469055	2.931596	3.583062	10.09772	7.491857	0.651466	4.560261	9.120521	1.302932	0.651466	2.931596	6.514658	8.143322	4.234528	2.28013	8.794788	U
SSA_1706	4.037267	4.968944	5.279503	6.521739	5.900621	8.695652	1.863354	6.521739	4.347826	1.552795	4.037267	8.695652	2.173913	0	4.347826	6.21118	4.968944	5.279503	6.21118	8.385093	L
SSA_2211	4.012346	4.62963	4.012346	7.407407	3.703704	5.246914	3.08642	9.876543	1.851852	2.160494	3.395062	14.50617	0.617284	0.925926	6.17284	11.7284	4.012346	2.160494	2.469136	8.024691	S
SSA_1247	3.439153	5.291005	4.761905	3.703704	9.52381	7.407407	1.851852	6.084656	3.174603	2.645503	4.497354	8.201058	1.322751	0.529101	4.497354	5.820106	9.259259	7.936508	5.820106	4.232804	L
SSA_0515	12.96296	1.851852	0.925926	6.481481	3.703704	7.407407	1.851852	11.11111	4.62963	0.925926	2.777778	8.333333	0	0	0.925926	3.703704	7.407407	7.407407	5.555556	12.03704	E
SSA_2261	9.655172	6.206897	2.758621	3.448276	6.896552	8.275862	2.068966	6.206897	6.206897	2.068966	0	11.03448	0	0.689655	2.068966	2.758621	5.517241	11.03448	4.827586	8.275862	K
SSA_1705	9.271523	2.649007	4.635762	9.271523	7.284768	5.298013	1.986755	7.284768	4.635762	1.324503	0.662252	10.59603	0.662252	0	4.635762	5.960265	3.97351	5.960265	6.622517	7.284768	
SSA_1781	9.150327	3.267974	1.960784	5.882353	7.189542	7.843137	5.882353	6.535948	1.960784	5.228758	2.614379	11.11111	0	0	3.921569	4.575163	3.921569	7.189542	8.496732	3.267974	K
SSA_1069	9.032258	1.935484	3.870968	3.870968	6.451613	3.870968	3.870968	10.32258	4.516129	1.935484	1.290323	15.48387	1.290323	0	3.225806	8.387097	7.096774	1.935484	4.516129	7.096774	MU
SSA_2130	8.860759	3.797468	2.531646	5.696203	3.797468	6.962025	3.797468	11.39241	3.797468	0.632911	2.531646	16.4557	1.898734	1.898734	3.797468	5.063291	5.696203	3.164557	2.531646	5.696203	
SSA_1390	8.695652	2.484472	7.453416	6.21118	9.31677	3.726708	1.242236	4.968944	5.590062	1.863354	2.484472	10.55901	2.484472	0.621118	4.347826	4.347826	6.832298	6.21118	5.590062	4.968944	
SSA_1504	8.641975	4.320988	2.469136	8.024691	8.024691	5.555556	3.08642	9.259259	1.851852	2.469136	3.703704	8.641975	1.851852	0.617284	6.17284	8.641975	4.938272	4.320988	3.703704	3.703704	
SSA_0735	8.484848	3.030303	4.848485	4.242424	6.666667	4.242424	4.242424	9.69697	1.818182	1.818182	3.636364	15.15152	3.030303	0	4.242424	9.090909	4.242424	2.424242	2.424242	6.666667	
SSA_0996	8.484848	1.212121	4.848485	6.666667	6.666667	5.454545	1.818182	4.848485	4.848485	1.818182	1.818182	13.33333	0.606061	1.818182	3.030303	4.242424	7.878788	8.484848	5.454545	6.666667	T
SSA_1698	8.187135	2.339181	5.263158	2.923977	8.187135	5.847953	1.754386	8.187135	1.169591	4.678363	1.754386	5.847953	0.584795	2.339181	3.508772	4.678363	9.356725	8.77193	6.432749	8.187135	G
SSA_2264	8.139535	0.581395	3.488372	12.2093	8.139535	4.069767	1.162791	9.302326	3.488372	0.581395	1.162791	13.95349	0.581395	0	4.651163	8.139535	4.651163	4.069767	2.325581	9.302326	
SSA_2045	8.092486	4.046243	2.312139	4.624277	3.468208	4.046243	2.312139	6.358382	8.092486	3.468208	3.468208	9.248555	0.578035	0.578035	5.202312	7.514451	8.092486	4.624277	9.82659	4.046243	H
SSA_0305	8.045977	4.597701	4.022989	5.747126	6.896552	6.896552	4.022989	9.195402	1.149425	0.574713	1.149425	12.06897	1.149425	0.574713	2.873563	10.91954	8.045977	2.873563	3.448276	5.747126	S
SSA_1348	8.045977	1.724138	1.724138	6.321839	5.172414	6.321839	4.022989	12.06897	2.298851	2.873563	5.172414	13.21839	0.574713	0.574713	2.873563	9.195402	5.172414	0.574713	1.724138	10.34483	
SSA_2171	7.865169	3.370787	1.123596	8.426966	7.303371	4.494382	2.808989	5.617978	7.303371	2.808989	2.808989	8.988764	0.561798	0	6.179775	4.494382	6.741573	6.741573	6.179775	6.179775	H
SSA_1834	7.777778	6.666667	3.333333	6.666667	9.444444	5	2.222222	7.777778	3.333333	1.111111	1.666667	9.444444	0	0	3.888889	2.777778	3.333333	11.11111	8.888889	5.555556	J
SSA_1536	7.734807	0.552486	1.657459	7.734807	4.972376	4.41989	1.657459	9.392265	1.657459	1.657459	6.077348	16.0221	0.552486	0	2.762431	11.04972	9.392265	1.104972	2.209945	9.392265	R

SSA_0354	7.650273	2.185792	3.278689	8.743169	3.278689	5.464481	3.825137	11.47541	3.825137	0	3.278689	11.47541	2.185792	1.092896	7.103825	7.650273	6.010929	1.092896	1.639344	8.743169	R
SSA_0539	7.608696	4.347826	3.26087	8.695652	4.891304	4.891304	4.347826	4.891304	6.521739	0	3.26087	10.86957	1.630435	1.086957	3.804348	0.543478	7.065217	7.608696	5.434783	9.23913	R
SSA_1186	7.486631	3.208556	6.951872	4.812834	4.812834	3.208556	2.139037	8.02139	3.208556	1.604278	5.882353	8.55615	1.604278	1.604278	3.208556	3.208556	10.16043	7.486631	5.347594	7.486631	R
SSA_0469	7.407407	5.820106	4.232804	4.232804	4.761905	4.232804	3.174603	6.878307	5.820106	2.116402	3.174603	8.465608	1.587302	1.587302	1.058201	3.703704	7.936508	10.58201	4.232804	8.994709	H
SSA_2297	7.070707	2.020202	2.020202	5.555556	7.070707	4.545455	3.535354	12.62626	2.020202	0.505051	3.030303	12.62626	3.030303	0.505051	3.030303	7.070707	9.090909	3.030303	1.515152	10.10101	
SSA_2293	6.965174	6.467662	5.970149	8.457711	5.472637	6.965174	1.492537	9.452736	6.467662	2.985075	1.99005	11.44279	1.492537	0.995025	3.482587	6.965174	2.487562	3.9801	2.985075	3.482587	
SSA_1758	6.930693	4.455446	4.950495	6.435644	7.920792	4.950495	0.990099	9.90099	3.465347	2.970297	2.475248	8.910891	0.49505	0.49505	6.930693	3.960396	7.920792	7.920792	4.950495	2.970297	
SSA_0433	6.730769	1.923077	3.365385	6.25	8.173077	4.807692	2.884615	3.365385	4.807692	0.480769	1.923077	12.5	0.480769	0.961538	1.923077	2.403846	6.730769	8.653846	5.769231	15.86538	E
SSA_0574	6.730769	2.403846	1.923077	5.288462	5.769231	5.769231	3.846154	12.98077	4.326923	0.961538	4.326923	9.615385	2.884615	1.923077	5.288462	7.692308	6.730769	1.442308	1.923077	8.173077	S
SSA_1585	6.698565	2.870813	3.349282	6.220096	6.698565	4.784689	2.870813	5.741627	2.392344	2.392344	2.870813	10.52632	0.478469	0	3.349282	3.827751	7.177033	5.263158	9.090909	13.39713	R
SSA_1837	6.666667	4.285714	1.904762	4.761905	9.52381	5.714286	2.857143	6.190476	2.857143	2.380952	1.428571	10	0	1.428571	3.333333	5.714286	8.095238	10.95238	4.761905	7.142857	S
SSA_1902	6.603774	4.716981	5.188679	5.188679	7.075472	3.301887	1.415094	5.660377	4.245283	2.830189	3.773585	9.433962	1.886792	0	5.188679	5.188679	7.075472	8.018868	6.603774	6.603774	R
SSA_0507	6.57277	2.816901	3.755869	5.633803	4.225352	3.286385	3.755869	11.26761	4.225352	2.816901	1.877934	17.84038	1.408451	1.408451	4.694836	10.79812	3.755869	3.286385	1.408451	5.164319	
SSA_0966	6.511628	7.44186	2.790698	3.72093	6.511628	6.511628	3.255814	11.62791	3.72093	2.325581	4.186047	6.511628	0	0	4.186047	3.255814	5.581395	8.837209	8.837209	4.186047	F
SSA_1237	6.451613	2.304147	4.147465	7.373272	5.990783	3.225806	0.460829	7.373272	5.069124	3.686636	7.373272	10.13825	2.304147	0	2.764977	4.608295	6.451613	4.608295	5.529954	10.13825	L
SSA_1404	6.278027	4.035874	2.690583	7.174888	9.865471	3.139013	2.242152	3.587444	6.726457	2.690583	1.345291	9.41704	1.793722	1.345291	5.381166	5.829596	5.381166	7.623318	7.174888	6.278027	S
SSA_2273	6.278027	4.484305	2.690583	7.623318	8.96861	6.278027	2.690583	8.520179	1.793722	0.896861	2.690583	13.45291	0.896861	0.896861	4.932735	9.41704	7.174888	5.381166	2.690583	2.242152	
SSA_0622	6.222222	4.444444	3.111111	8	6.666667	4.888889	1.777778	4	4	2.666667	4	11.55556	1.333333	0.444444	7.555556	3.111111	5.333333	8.444444	5.777778	6.666667	K
SSA_1788	6.167401	2.643172	5.286344	9.251101	2.643172	4.405286	3.524229	13.65639	2.643172	0.881057	1.321586	12.77533	1.321586	0.440529	3.524229	7.488987	8.810573	0.881057	3.0837	9.251101	R
SSA_0736	6.140351	6.140351	1.754386	7.017544	8.333333	4.824561	2.631579	6.140351	4.824561	1.754386	3.070175	11.40351	0	0.877193	6.140351	7.017544	3.508772	7.894737	6.140351	4.385965	T
SSA_0476	6.008584	1.287554	1.716738	8.154506	7.725322	6.866953	3.433476	6.866953	3.862661	1.716738	4.72103	13.30472	0.429185	0.858369	3.004292	3.862661	6.008584	8.583691	4.291845	7.296137	H
SSA_1804	5.882353	5.882353	4.201681	6.722689	5.462185	4.201681	1.260504	5.882353	6.722689	1.680672	3.361345	8.823529	0.840336	0	4.201681	4.621849	5.462185	9.663866	4.201681	10.92437	C
SSA_0910	5.809129	3.73444	2.074689	4.979253	8.298755	7.053942	4.149378	5.809129	4.149378	1.244813	2.074689	11.61826	0.829876	0.829876	2.489627	2.904564	7.46888	11.20332	4.564315	8.713693	V
SSA_2010	5.785124	2.479339	2.892562	8.677686	4.545455	3.305785	5.785124	9.504132	2.892562	1.652893	2.892562	18.59504	2.066116	0.413223	2.892562	8.677686	5.785124	2.479339	2.066116	6.61157	
SSA_1535	5.761317	3.292181	2.469136	5.349794	5.349794	3.703704	1.234568	6.995885	6.17284	4.526749	5.761317	10.69959	2.469136	0	2.057613	5.349794	6.584362	8.641975	7.407407	6.17284	R
SSA_0322	5.714286	4.489796	3.673469	4.489796	5.714286	4.489796	2.040816	4.489796	9.795918	3.265306	2.040816	10.20408	0	0.408163	6.122449	3.673469	5.714286	10.20408	4.081633	9.387755	K
SSA_1180	5.691057	3.658537	5.284553	5.691057	3.252033	2.439024	3.252033	6.097561	2.845528	5.284553	3.658537	5.691057	4.878049	1.219512	4.878049	7.317073	7.723577	6.504065	6.910569	7.723577	S
SSA_0321	5.645161	4.435484	4.435484	3.629032	10.48387	5.241935	5.645161	4.435484	4.435484	1.209677	1.612903	10.48387	0.806452	1.612903	2.419355	4.435484	8.064516	6.451613	6.048387	8.467742	H
SSA_0711	5.645161	5.645161	5.645161	6.048387	7.258065	3.225806	2.822581	8.064516	5.645161	2.419355	4.032258	12.09677	0	1.209677	3.225806	3.225806	5.241935	6.451613	6.048387	6.048387	R
SSA_0728	5.577689	3.187251	4.780876	6.374502	3.585657	3.585657	1.992032	6.374502	3.585657	1.992032	1.992032	21.51394	3.187251	0	1.593625	8.76494	7.968127	3.187251	1.992032	8.76494	R
SSA_1579	5.577689	3.585657	3.187251	5.577689	7.968127	4.38247	1.992032	8.366534	8.366534	3.585657	3.187251	13.54582	0.398406	1.195219	5.179283	1.593625	6.374502	4.38247	5.577689	5.976096	PH
SSA_1743	5.577689	2.390438	2.788845	7.968127	3.187251	5.976096	2.390438	9.163347	3.984064	1.992032	2.788845	21.11554	1.195219	1.195219	2.390438	4.780876	9.561753	1.195219	0.796813	9.561753	P
SSA_0411	5.555556	1.984127	3.968254	11.11111	3.968254	6.349206	2.777778	9.52381	1.587302	1.587302	1.587302	14.68254	1.190476	1.190476	3.968254	10.71429	6.349206	0.793651	2.777778	8.333333	
SSA_1881	5.533597	3.557312	4.347826	5.928854	5.533597	5.13834	1.581028	5.928854	5.13834	3.162055	1.976285	10.67194	3.162055	0.395257	6.324111	6.324111	2.766798	8.695652	7.905138	5.928854	
SSA_0034	5.511811	3.937008	11.02362	8.267717	7.480315	5.511811	1.968504	11.02362	3.937008	0.787402	1.574803	9.055118	0.393701	0.787402	5.11811	5.11811	3.543307	6.299213	6.692913	1.968504	
SSA_1188	5.511811	4.330709	2.755906	4.330709	10.62992	2.755906	2.755906	8.267717	4.724409	1.181102	4.330709	10.23622	0	0.393701	3.937008	1.574803	5.511811	7.480315	7.480315	11.81102	L
SSA_1765	5.511811	3.543307	7.874016	3.937008	9.055118	4.330709	3.149606	6.692913	2.755906	0.787402	4.330709	9.448819	1.574803	1.181102	4.724409	4.724409	7.086614	9.84252	5.905512	3.543307	H
SSA_2046	5.511811	5.11811	1.574803	5.11811	5.905512	5.11811	2.362205	8.267717	2.362205	3.149606	3.937008	7.874016	1.574803	0.787402	4.330709	4.330709	5.905512	9.84252	6.692913	10.23622	F
SSA_1987	5.363985	2.681992	2.681992	5.363985	4.980843	5.363985	1.915709	11.49425	3.831418	1.915709	3.448276	14.55939	2.298851	0	5.363985	10.34483	6.51341	3.065134	3.065134	5.747126	R
SSA_1316	5.323194	3.041825	3.422053	6.463878	6.844106	5.323194	3.041825	9.505703	4.942966	0.760456	1.140684	13.30798	5.323194	0.760456	6.463878	4.942966	8.365019	3.422053	2.281369	5.323194	H
SSA_2365	5.30303	6.060606	3.030303	7.19697	5.30303	5.681818	3.409091	10.60606	2.272727	0.757576	3.409091	17.04545	0.757576	0	1.136364	9.469697	6.060606	2.272727	3.030303	7.19697	P
SSA_0498	5.204461	2.230483	2.973978	9.29368	4.089219	5.576208	3.345725	11.15242	4.089219	0.371747	4.089219	14.86989	1.858736	0.743494	2.973978	5.947955	8.178439	2.230483	2.60223	8.178439	EP
SSA_0426	5.128205	1.831502	2.197802	6.959707	4.395604	6.959707	2.197802	8.424908	2.564103	1.465201	3.663004	16.84982	1.831502	0	4.761905	6.593407	7.692308	4.761905	1.831502	9.89011	S
SSA_0056	5.109489	1.824818	4.379562	5.109489	6.20438	5.474453	6.20438	9.489051	3.649635	0.729927	3.649635	9.489051	2.554745	0	2.919708	8.394161	9.124088	4.379562	3.284672	8.029197	G
SSA_0916	5.090909	3.636364	2.909091	5.090909	6.181818	2.545455	2.909091	7.272727	5.818182	2.181818	4	11.63636	0.363636	1.090909	4.363636	5.454545	8.363636	7.272727	5.090909	8.727273	R
SSA_0868	4.895105	6.293706	3.496503	4.895105	5.594406	3.846154	1.748252	5.244755	7.692308	5.594406	3.496503	12.23776	1.048951	0	4.195804	8.041958	4.195804	6.993007	6.643357	3.846154	L
SSA_0676	4.810997	3.780069	1.718213	5.841924	5.841924	4.123711	2.061856	6.52921	5.841924	1.718213	2.405498	15.12027	0	0	2.405498	4.123711	8.247423	7.560137	7.90378	9.965636	H
SSA_2041	4.810997	4.123711	3.092784	5.841924	5.154639	4.123711	1.030928	3.780069	8.934708	3.436426	3.436426	18.5567	2.749141	0.687285	3.780069	3.780069	2.405498	7.90378	5.498282	6.872852	TQ
SSA_1433	4.761905	7.482993	3.061224	5.102041	4.421769	3.401361	2.040816	8.163265	5.782313	2.040816	3.401361	10.54422	1.020408	0.680272	4.421769	3.061224	7.482993	9.863946	5.442177	7.823129	J
SSA_1610	4.651163	6.976744	3.654485	4.651163	5.647841	2.990033	4.651163	6.312292	4.651163	1.993355	4.651163	11.96013	0.664452	0.332226	4.651163	2.990033	5.980066	7.973422	6.976744	7.641196	S
SSA_0812	4.620462	6.60066	4.950495	8.580858	6.270627	4.620462	3.30033	7.260726	3.630363	2.310231	1.320132	13.20132	0	0	2.640264	2.640264	3.960396	7.590759	6.60066	9.90099	S
SSA_0332	4.501608	4.501608	3.215434	4.180064	5.144695	6.109325	1.286174	4.501608	7.073955	2.572347	4.180064	13.82637	1.607717	0.321543	3.858521	5.787781	7.717042	6.752412	5.787781	7.073955	V
SSA_2177	4.281346	5.198777	2.752294	13.45566	9.174312	6.422018	0.30581	4.281346	7.033639	0.611621	2.140673	7.95107	1.834862	0	5.198777	4.281346	5.810398	7.64526	6.116208	5.504587	
SSA_0205	4.057971	3.188406	4.347826	7.826087	5.217391	3.768116	1.73913	5.217391	8.115942	3.768116	1.449275	17.10145	1.15942	0.869565	3.188406	5.797101	4.057971	7.536232	4.637681	6.956522	T
SSA_0525	16.48352	2.197802	4.395604	1.098901	5.494505	4.395604	3.296703	5.494505	1.098901	2.197802	3.296703	7.692308	0	0	0	0	10.98901	7.692308	3.296703	20.87912	QC
SSA_0524	16.12903	3.225806	4.301075	1.075269	3.225806	4.301075	4.301075	5.376344	2.150538	3.225806	3.225806	6.451613	0	0	0	0	10.75269	8.602151	3.225806	20.43011	QC
SSA_2074	13.51351	1.801802	1.801802	2.702703	6.306306	8.108108	4.504505	6.306306	5.405405	0	3.603604	9.009009	0	0	3.603604	2.702703	9.009009	9.90991	5.405405	6.306306	U
SSA_1037	11.62791	2.325581	3.100775	5.426357	6.20155	8.527132	2.325581	3.875969	1.550388	0.775194	3.875969	8.527132	0	2.325581	3.100775	6.20155	6.20155	8.527132	4.651163	10.85271	F
SSA_2327	11.45038	0	7.633588	20.61069	6.870229	4.580153	0.763359	2.290076	2.290076	0.763359	1.526718	5.343511	0	0.763359	0	2.290076	12.21374	5.343511	6.870229	8.396947	F
SSA_1545	11.36364	2.272727	5.30303	7.575758	9.848485	9.848485	1.515152	7.575758	1.515152	0.757576	0.757576	13.63636	0	0	1.515152	3.030303	3.787879	3.787879	6.060606	9.848485	R
SSA_1612	11.36364	3.787879	5.30303	7.575758	6.060606	2.272727	2.272727	9.090909	0.757576	1.515152	0.757576	10.60606	3.030303	0	0.757576	9.090909	4.545455	4.545455	5.30303	11.36364	M
SSA_0219	10.34483	0	0.689655	8.275862	4.827586	4.827586	3.448276	4.137931	4.137931	0.689655	0.689655	14.48276	0	0	0.689655	2.758621	10.34483	4.827586	11.72414	13.10345	G
SSA_1270	10.27397	1.369863	1.369863	4.794521	4.794521	7.534247	2.739726	4.109589	0	0	0.684932	7.534247	0	1.369863	3.424658	5.479452	6.849315	10.9589	14.38356	12.32877	C

SSA_2160	10.20408	4.761905	4.081633	5.442177	5.442177	4.761905	2.040816	5.442177	2.721088	2.040816	4.761905	8.163265	0	0	4.081633	4.081633	10.88435	8.163265	5.442177	7.482993	F
SSA_0073	10	2	5.333333	6	8.666667	5.333333	1.333333	6.666667	2.666667	2.666667	4.666667	10.66667	0	1.333333	4	5.333333	6	8.666667	6.666667	2	G
SSA_0563	10	4	3.333333	6	8	6	2.666667	6	2.666667	2.666667	2	11.33333	0	0	2.666667	2	5.333333	9.333333	7.333333	8.666667	T
SSA_2248	9.202454	1.226994	7.361963	6.748466	14.11043	4.907975	1.226994	8.588957	2.453988	0	1.226994	6.134969	0.613497	0	3.680982	6.748466	6.134969	9.202454	5.521472	4.907975	
SSA_0220	9.146341	3.04878	4.268293	5.487805	10.97561	4.878049	1.829268	7.317073	3.658537	0.609756	3.658537	4.268293	1.829268	0.609756	3.04878	5.487805	7.926829	6.707317	6.707317	8.536585	G
SSA_0259	8.522727	2.840909	5.681818	5.681818	5.113636	9.090909	1.704545	5.113636	2.840909	2.272727	2.840909	9.659091	1.704545	1.136364	3.409091	4.545455	4.545455	5.113636	8.522727	9.659091	O
SSA_0624	8.379888	6.145251	1.117318	3.351955	7.821229	5.027933	3.351955	6.703911	3.351955	0	3.910615	10.61453	0.558659	1.117318	3.351955	2.793296	9.497207	10.61453	6.145251	6.145251	L
SSA_0021	7.936508	0.529101	5.291005	14.81481	10.58201	4.232804	1.587302	1.058201	4.232804	0	6.878307	4.761905	0.529101	0	1.587302	1.587302	3.703704	8.994709	4.761905	16.93122	
SSA_0610	7.936508	3.174603	6.349206	6.878307	6.878307	6.878307	1.587302	6.349206	5.820106	0	3.174603	9.52381	0.529101	0	4.232804	4.761905	4.761905	6.878307	3.703704	10.58201	S
SSA_1151	7.894737	4.736842	3.684211	4.210526	8.421053	4.736842	3.157895	8.421053	4.210526	3.157895	2.631579	7.368421	0	2.105263	5.789474	3.684211	6.842105	6.842105	6.842105	5.263158	F
SSA_0516	7.853403	5.235602	1.570681	5.235602	9.424084	1.570681	4.712042	9.947644	3.664921	1.04712	3.141361	12.56545	0	1.570681	3.141361	1.04712	3.664921	6.806283	9.424084	8.376963	T
SSA_2254	7.653061	0.510204	3.061224	5.102041	10.20408	4.591837	2.55102	7.653061	5.102041	1.530612	2.55102	9.693878	0	0	5.612245	3.061224	6.122449	9.183673	3.061224	12.7551	L
SSA_0579	7.537688	5.025126	3.015075	4.020101	7.537688	4.522613	3.015075	5.025126	2.01005	3.517588	2.512563	10.55276	1.005025	0.502513	5.527638	2.01005	6.532663	10.55276	6.532663	9.045226	H
SSA_0658	7.537688	8.040201	3.517588	4.522613	7.035176	5.025126	2.01005	5.025126	7.035176	1.005025	8.542714	7.537688	0	0.502513	3.517588	3.015075	1.507538	8.542714	7.537688	8.542714	S
SSA_2301	7.537688	4.020101	3.517588	10.55276	8.542714	4.522613	2.512563	5.527638	3.015075	2.01005	3.517588	6.532663	0.502513	0	3.015075	6.532663	7.537688	2.512563	6.030151	12.0603	G
SSA_1283	7.352941	5.882353	5.882353	3.921569	7.843137	4.901961	1.960784	5.882353	4.411765	1.960784	4.901961	10.29412	0.490196	0	2.45098	4.901961	3.921569	7.352941	6.372549	9.313725	C
SSA_1013	7.142857	3.809524	2.380952	5.238095	3.809524	3.333333	3.333333	7.142857	2.380952	1.428571	4.285714	17.14286	1.428571	1.904762	4.285714	7.142857	6.666667	2.380952	2.857143	11.90476	I
SSA_1922	7.142857	6.666667	1.904762	4.761905	7.619048	4.285714	2.857143	4.285714	3.809524	3.333333	1.904762	12.38095	1.428571	1.428571	3.333333	6.190476	7.619048	6.666667	3.809524	8.571429	R
SSA_2006	7.142857	2.857143	5.238095	6.190476	6.190476	4.285714	1.428571	7.142857	3.809524	1.428571	3.333333	12.85714	0.47619	1.428571	3.333333	7.142857	4.285714	8.571429	5.238095	7.619048	R
SSA_2158	7.142857	3.809524	4.285714	8.095238	5.238095	3.333333	1.904762	4.285714	8.095238	0.47619	3.809524	15.71429	0.952381	1.428571	3.333333	4.761905	4.285714	8.095238	3.333333	7.619048	H
SSA_2354	7.142857	2.380952	4.285714	7.142857	6.666667	4.761905	1.428571	9.047619	3.809524	2.380952	2.380952	11.90476	1.904762	0.952381	5.714286	3.809524	4.761905	10.95238	3.333333	5.238095	R
SSA_1405	7.109005	3.317536	2.843602	2.843602	4.265403	3.791469	4.739336	8.530806	5.21327	2.843602	3.317536	9.004739	0.947867	1.421801	2.369668	3.791469	9.478673	8.530806	6.161137	9.478673	P
SSA_0882	6.976744	6.046512	2.790698	0.930233	6.046512	4.186047	4.186047	6.976744	4.186047	1.395349	2.325581	12.55814	0.465116	0.465116	1.860465	2.325581	9.302326	9.302326	6.511628	11.16279	E
SSA_1398	6.78733	1.357466	2.262443	7.239819	5.429864	4.072398	4.524887	12.21719	2.262443	3.167421	2.262443	12.66968	2.262443	0.452489	7.239819	6.334842	8.144796	1.809955	0.452489	9.049774	R
SSA_1210	6.726457	8.520179	4.035874	3.139013	6.278027	4.035874	3.139013	9.41704	5.381166	2.242152	2.690583	7.623318	0.896861	0.44843	4.484305	2.690583	4.932735	12.10762	6.726457	4.484305	S
SSA_1470	6.666667	3.555556	4.444444	8	5.777778	4	4.444444	7.111111	3.555556	1.333333	3.555556	8.888889	0.888889	0	3.111111	5.777778	4.444444	11.55556	6.222222	6.666667	E
SSA_0401	6.637168	3.982301	4.424779	6.19469	7.522124	4.867257	1.327434	6.19469	3.982301	2.654867	2.654867	15.04425	0.884956	0.442478	3.539823	3.097345	5.309735	11.50442	4.867257	4.867257	TK
SSA_0752	6.60793	5.286344	3.964758	5.286344	5.726872	4.405286	3.0837	6.167401	4.845815	1.321586	5.286344	13.65639	0	0	2.643172	4.405286	5.286344	7.488987	7.488987	7.048458	R
SSA_1431	6.60793	3.524229	3.524229	8.370044	5.726872	4.405286	1.762115	3.964758	7.048458	0.440529	5.286344	10.13216	1.321586	0.440529	6.167401	5.726872	5.286344	7.929515	3.964758	8.370044	G
SSA_1417	6.550218	6.550218	3.056769	5.676856	6.550218	2.183406	1.310044	6.550218	6.9869	1.746725	3.056769	13.10044	0.873362	0	1.310044	3.49345	6.550218	12.22707	3.056769	9.170306	R
SSA_1531	6.465517	4.310345	4.310345	6.465517	8.62069	4.741379	3.017241	7.327586	4.741379	1.724138	3.017241	12.93103	0	0	1.724138	2.586207	8.62069	9.051724	3.87931	6.465517	V
SSA_2016	6.437768	6.866953	4.291845	7.296137	3.433476	5.579399	1.716738	6.866953	2.575107	1.287554	3.004292	9.012876	2.145923	0.858369	3.433476	3.433476	12.44635	8.154506	5.150215	6.008584	G
SSA_0028	6.382979	3.829787	4.680851	4.680851	10.21277	3.404255	0.851064	8.085106	3.829787	1.702128	1.702128	9.361702	1.276596	0.425532	2.553191	7.234043	6.382979	7.659574	9.361702	6.382979	F
SSA_0136	6.355932	6.355932	4.237288	6.779661	7.627119	4.237288	2.542373	5.508475	2.966102	5.932203	3.813559	6.779661	1.271186	0.423729	3.813559	4.661017	7.20339	7.20339	5.932203	6.355932	P
SSA_1115	6.355932	1.694915	2.542373	6.779661	5.508475	3.389831	3.813559	8.050847	3.813559	0.423729	3.813559	17.79661	1.694915	1.271186	2.966102	7.627119	12.28814	0	1.694915	8.474576	O
SSA_0052	6.302521	4.621849	5.042017	4.621849	9.243697	6.722689	1.680672	9.663866	5.882353	2.941176	3.781513	5.462185	0	0.420168	6.722689	5.882353	3.781513	7.142857	7.142857	2.941176	K
SSA_0794	6.276151	4.60251	4.60251	8.368201	3.34728	10.04184	2.09205	6.694561	5.439331	3.34728	1.25523	7.112971	1.67364	0	3.76569	5.020921	6.276151	5.857741	5.857741	8.368201	O
SSA_2351	6.25	4.166667	2.083333	5.416667	7.5	4.583333	2.083333	7.5	6.25	2.5	2.083333	16.66667	0.833333	0	3.333333	2.916667	6.666667	7.916667	5.416667	5.833333	P
SSA_0490	6.198347	4.545455	1.239669	6.61157	3.305785	4.545455	2.892562	7.024793	1.652893	1.239669	2.892562	20.66116	1.652893	0	5.785124	6.198347	11.15702	2.066116	3.305785	7.024793	H
SSA_0986	6.198347	5.371901	2.892562	4.958678	5.371901	5.785124	3.719008	8.264463	4.958678	1.239669	4.132231	11.15702	0.413223	0	2.479339	2.892562	7.024793	8.264463	7.438017	7.438017	E
SSA_2224	6.17284	4.938272	3.292181	6.17284	9.053498	4.115226	3.292181	6.584362	2.880658	3.703704	3.703704	10.69959	0	0.823045	3.703704	5.349794	5.761317	7.81893	5.761317	6.17284	GM
SSA_0911	6.097561	2.845528	2.845528	10.56911	3.252033	2.845528	5.284553	7.723577	1.626016	0.813008	4.471545	16.66667	1.219512	0.406504	2.03252	10.1626	7.317073	1.626016	1.626016	10.56911	S
SSA_1766	6.072874	2.42915	3.238866	8.502024	4.453441	5.668016	2.834008	9.716599	0.809717	0.404858	2.42915	14.5749	2.834008	2.024291	4.453441	8.502024	8.097166	2.834008	2.834008	7.287449	R
SSA_1230	6.048387	4.83871	3.629032	5.241935	11.29032	8.064516	1.209677	3.225806	5.645161	2.419355	1.209677	12.5	0	0	4.032258	2.822581	3.225806	8.870968	5.241935	10.48387	
SSA_1429	5.976096	4.38247	5.179283	7.968127	7.569721	3.585657	3.585657	7.569721	1.992032	1.593625	3.187251	10.75697	0	0	3.585657	5.976096	7.171315	5.577689	5.179283	9.163347	R
SSA_1682	5.976096	3.984064	3.984064	5.577689	3.585657	3.585657	4.38247	5.577689	2.788845	2.390438	2.390438	17.13147	2.788845	1.195219	3.984064	10.35857	7.968127	1.992032	2.390438	7.968127	
SSA_1726	5.905512	4.330709	5.905512	1.968504	6.692913	5.905512	2.362205	6.299213	1.574803	2.362205	3.149606	15.74803	0	0	3.149606	3.543307	8.267717	9.055118	4.330709	9.448819	E
SSA_1050	5.836576	2.33463	3.501946	7.003891	3.501946	5.058366	3.891051	10.50584	3.11284	1.167315	2.33463	14.00778	0	0	5.447471	10.50584	4.669261	3.501946	3.501946	10.11673	E
SSA_0638	5.791506	2.702703	1.930502	5.791506	6.563707	5.405405	1.158301	9.65251	3.474903	3.088803	5.019305	12.74131	0	0	2.702703	4.247104	8.108108	6.949807	6.563707	8.108108	E
SSA_0480	5.535055	2.583026	3.690037	5.166052	6.642066	4.797048	4.428044	6.273063	7.380074	2.583026	3.690037	11.43911	0.738007	0.738007	4.059041	3.690037	7.749077	5.904059	6.642066	6.273063	P
SSA_2194	5.535055	3.321033	4.059041	7.380074	5.904059	4.797048	1.845018	5.904059	5.535055	1.845018	2.214022	13.28413	5.535055	0.738007	5.166052	5.535055	7.380074	2.95203	2.583026	8.487085	H
SSA_1055	5.514706	4.044118	4.044118	8.455882	8.455882	5.147059	1.102941	8.823529	2.205882	1.470588	4.779412	12.86765	0	0.367647	4.411765	4.044118	4.044118	8.088235	6.25	5.882353	S
SSA_0389	5.434783	5.072464	2.898551	5.072464	4.710145	3.985507	6.15942	6.521739	5.072464	1.449275	2.536232	11.95652	1.086957	0	2.536232	3.985507	7.971014	9.42029	5.797101	8.333333	R
SSA_1005	5.415162	1.805054	5.415162	5.776173	5.415162	6.498195	5.415162	12.99639	2.527076	0.361011	4.693141	10.83032	1.805054	0	5.776173	7.581227	6.137184	1.444043	2.888087	7.220217	G
SSA_0173	5.376344	3.942652	4.301075	5.017921	8.602151	3.225806	1.075269	7.168459	8.960573	3.225806	3.942652	11.82796	0.716846	1.433692	3.584229	3.942652	2.150538	6.09319	6.09319	9.318996	H
SSA_1185	5.357143	4.285714	5	6.071429	7.5	2.857143	1.785714	7.142857	3.214286	2.142857	3.928571	11.78571	0.357143	2.142857	3.214286	4.285714	8.214286	7.142857	5.357143	8.214286	LU
SSA_1400	5.208333	2.083333	1.388889	5.555556	6.597222	6.25	2.430556	4.861111	5.208333	1.736111	4.166667	14.58333	0.694444	1.041667	3.125	6.597222	5.902778	7.638889	6.944444	7.986111	H
SSA_1679	5.208333	5.555556	3.472222	4.861111	5.902778	6.944444	2.083333	7.291667	6.944444	1.041667	3.472222	12.5	0.694444	0.694444	2.777778	2.777778	7.986111	6.597222	5.208333	7.986111	V
SSA_0276	5.190311	6.228374	3.114187	6.920415	3.114187	3.806228	1.384083	6.920415	5.882353	2.076125	2.076125	10.72664	1.384083	2.422145	8.304498	4.152249	4.49827	8.99654	6.228374	6.574394	M
SSA_1411	5.190311	3.806228	4.152249	4.49827	5.536332	4.152249	3.114187	7.612457	3.806228	1.730104	5.190311	10.72664	0.346021	0	5.536332	3.114187	8.99654	8.650519	5.882353	7.958478	M
SSA_2017	5.033557	3.691275	2.348993	7.04698	3.691275	4.026846	1.342282	8.389262	3.691275	1.342282	1.342282	13.42282	0.671141	2.348993	5.033557	7.38255	8.389262	6.711409	5.704698	8.389262	R
SSA_0640	5.016722	5.016722	5.351171	7.692308	8.026756	7.023411	2.341137	9.0301	3.344482	2.006689	3.67893	10.36789	1.003344	0.334448	5.351171	5.016722	4.682274	4.682274	6.688963	3.344482	K
SSA_1248	4.966887	2.980132	3.97351	8.940397	9.271523	4.966887	1.655629	3.642384	3.97351	1.655629	3.642384	11.25828	0.331126	0.662252	5.298013	7.284768	6.622517	5.960265	6.291391	6.622517	L

SSA_1927	4.918033	3.934426	2.295082	7.213115	2.95082	3.934426	1.639344	4.262295	2.95082	1.639344	4.590164	25.57377	3.934426	0.983607	5.901639	6.229508	4.918033	0.983607	1.639344	9.508197	P
SSA_1312	4.87013	2.922078	5.519481	8.441558	7.792208	2.272727	4.220779	5.519481	1.948052	3.571429	2.922078	10.06494	0.649351	1.298701	7.467532	6.493506	5.194805	6.493506	7.467532	4.87013	V
SSA_1436	4.87013	4.87013	3.246753	7.142857	8.441558	4.545455	1.298701	8.116883	5.194805	1.623377	3.571429	11.68831	0.649351	0.324675	4.545455	6.493506	3.246753	9.415584	6.168831	4.545455	K
SSA_1813	4.854369	3.883495	7.76699	7.76699	6.796117	4.530744	2.265372	10.03236	4.20712	1.618123	1.941748	9.708738	0	1.294498	5.177994	8.414239	3.883495	7.76699	5.177994	2.912621	
SSA_1126	4.823151	3.536977	4.501608	9.967846	3.858521	5.787781	1.286174	8.681672	5.144695	3.536977	2.893891	14.14791	0.96463	1.607717	2.250804	2.250804	6.752412	7.073955	4.823151	6.109325	H
SSA_0749	4.746835	5.379747	3.481013	4.113924	6.64557	2.848101	1.898734	3.481013	10.44304	3.164557	3.164557	13.92405	1.582278	2.531646	5.063291	5.696203	3.481013	7.594937	4.113924	6.64557	R
SSA_1820	4.731861	5.047319	5.047319	5.993691	10.41009	3.470032	3.470032	16.40379	1.892744	0.946372	1.26183	13.88013	1.892744	0	5.047319	8.832808	3.470032	2.208202	3.154574	2.839117	
SSA_1653	4.6875	2.5	5.9375	7.5	9.6875	4.0625	1.5625	6.5625	4.375	2.5	3.125	8.75	1.875	0	7.5	4.375	4.0625	8.4375	8.125	4.375	
SSA_1597	4.658385	4.037267	4.658385	6.521739	9.937888	4.658385	0.931677	8.385093	4.347826	2.484472	4.037267	8.074534	1.552795	0	5.279503	5.590062	5.279503	9.31677	7.453416	2.795031	
SSA_1599	4.62963	3.395062	6.17284	4.938272	11.11111	5.555556	3.08642	6.790123	4.012346	0.925926	2.160494	8.333333	0.925926	0	7.407407	5.864198	6.481481	9.259259	6.17284	2.777778	
SSA_0168	4.601227	5.521472	2.760736	7.668712	6.441718	2.453988	1.226994	5.214724	8.588957	1.840491	3.98773	13.80368	1.226994	0.613497	7.055215	6.441718	2.453988	7.97546	5.521472	4.601227	
SSA_0182	4.385965	2.046784	7.017544	6.432749	9.649123	5.847953	2.923977	4.093567	6.432749	0.584795	2.046784	8.77193	1.754386	0.292398	7.017544	3.216374	4.385965	4.97076	7.309942	10.81871	G
SSA_0158	3.778338	2.518892	4.030227	9.823678	8.060453	4.534005	1.511335	6.04534	4.534005	1.007557	3.778338	9.823678	0.251889	0	4.030227	5.037783	7.808564	10.07557	6.297229	7.052897	
SSA_1825	3.157895	5.052632	3.157895	6.315789	4.421053	4.210526	2.947368	8	9.052632	3.789474	1.473684	13.89474	1.263158	1.052632	5.052632	5.052632	4.842105	6.736842	5.894737	4.631579	K
SSA_0581	13.22314	3.305785	4.132231	4.132231	5.785124	3.305785	2.479339	2.479339	2.479339	3.305785	0	11.57025	1.652893	0	1.652893	1.652893	7.438017	11.57025	5.785124	14.04959	S
SSA_0842	12.69841	2.380952	3.174603	4.761905	3.174603	1.587302	4.761905	12.69841	3.174603	1.587302	4.761905	17.46032	0	0.793651	2.380952	6.349206	7.142857	3.174603	2.380952	5.555556	R
SSA_0483	11.18881	4.895105	4.195804	5.594406	9.090909	5.594406	2.797203	7.692308	5.594406	0.699301	4.195804	6.993007	0.699301	0.699301	2.097902	2.097902	4.895105	4.895105	5.594406	10.48951	H
SSA_0339	10.32258	1.935484	2.580645	6.451613	4.516129	7.096774	3.225806	12.90323	3.870968	1.290323	3.225806	16.77419	0.645161	0.645161	4.516129	3.870968	5.806452	0	0.645161	9.677419	S
SSA_0054	10.12658	5.696203	5.063291	5.063291	7.594937	5.063291	3.164557	7.594937	2.531646	1.898734	3.797468	11.39241	0.632911	0	0.632911	1.898734	5.696203	6.329114	6.329114	9.493671	G
SSA_0086	10.06289	1.257862	1.257862	4.402516	4.402516	3.773585	3.144654	6.289308	5.031447	1.257862	3.144654	12.57862	0	0	3.773585	7.54717	11.94969	4.402516	0	15.72327	C
SSA_1548	9.815951	4.907975	6.134969	4.907975	4.907975	2.453988	1.226994	2.453988	5.521472	3.680982	4.294479	11.65644	1.226994	2.453988	3.680982	4.294479	6.748466	9.202454	6.134969	4.294479	F
SSA_1421	9.411765	4.705882	1.764706	1.764706	6.470588	4.705882	3.529412	8.823529	1.176471	1.176471	4.705882	7.647059	0	1.176471	5.882353	2.941176	10	7.647059	7.647059	8.823529	F
SSA_1303	9.302326	4.069767	5.813953	1.744186	9.302326	1.744186	2.325581	8.139535	2.906977	1.744186	2.906977	9.302326	0.581395	0	3.488372	5.813953	7.55814	9.883721	9.302326	4.069767	J
SSA_1399	8.510638	3.191489	2.12766	4.255319	5.851064	5.319149	1.06383	9.574468	4.255319	1.595745	3.191489	16.48936	0	0.531915	2.12766	7.978723	7.446809	2.659574	1.595745	12.23404	S
SSA_0505	8.376963	6.282723	3.664921	8.376963	6.806283	6.806283	3.664921	6.806283	2.617801	1.04712	2.617801	5.759162	0	1.570681	4.188482	5.759162	6.806283	7.329843	7.329843	4.188482	R
SSA_2149	8.290155	4.145078	3.108808	9.326425	10.36269	4.145078	2.072539	9.84456	2.590674	1.554404	3.108808	14.50777	1.554404	1.036269	2.072539	5.699482	5.181347	4.145078	2.590674	4.663212	
SSA_1410	8.121827	4.060914	5.583756	4.568528	7.614213	3.045685	1.522843	3.045685	3.045685	1.015228	5.583756	9.64467	2.538071	0	3.553299	6.598985	7.614213	8.629442	7.106599	7.106599	M
SSA_1768	8.080808	5.555556	3.535354	4.545455	9.090909	6.565657	4.545455	8.585859	4.545455	2.525253	0.505051	9.59596	0	0.505051	4.040404	5.555556	2.525253	9.59596	5.050505	5.050505	K
SSA_2288	7.843137	1.960784	4.901961	6.372549	4.901961	3.921569	2.941176	11.27451	1.960784	0	1.960784	19.17647	0.980392	0.490196	3.921569	7.352941	7.352941	2.45098	3.921569	6.372549	P
SSA_0405	7.804878	3.902439	1.95122	9.268293	5.853659	2.926829	1.95122	8.780488	5.853659	0.487805	3.902439	16.58537	1.463415	0.97561	2.926829	5.853659	6.829268	3.902439	4.390244	4.390244	K
SSA_2026	7.692308	2.403846	4.807692	6.25	4.807692	3.846154	3.846154	11.05769	1.923077	0	1.923077	18.26923	0.961538	0.961538	3.846154	7.692308	7.211538	2.403846	3.846154	6.25	P
SSA_1054	7.655502	4.784689	2.392344	8.133971	6.698565	6.698565	2.870813	8.61244	2.392344	1.913876	2.870813	12.91866	0	0.956938	2.870813	3.827751	2.870813	6.698565	8.133971	6.698565	R
SSA_1711	7.655502	5.263158	2.870813	3.827751	7.655502	3.349282	3.349282	5.263158	3.827751	3.827751	6.698565	8.61244	0.478469	1.913876	2.870813	4.784689	3.827751	5.741627	6.220096	11.96172	L
SSA_1346	7.619048	4.285714	2.857143	4.285714	6.190476	4.761905	4.285714	10	0.952381	0.952381	4.285714	13.33333	1.904762	1.428571	7.619048	10.47619	5.238095	0.952381	3.333333	5.238095	
SSA_1568	7.079646	3.097345	3.539823	7.964602	3.097345	5.309735	2.212389	10.17699	4.424779	0.884956	3.982301	11.50442	1.769912	0.884956	5.309735	8.40708	7.079646	2.654867	0.884956	9.734513	E
SSA_0695	7.017544	3.508772	3.070175	5.701754	2.631579	6.578947	4.385965	6.578947	3.070175	2.192982	5.263158	15.78947	2.192982	1.754386	7.894737	8.77193	5.701754	1.315789	1.754386	4.824561	S
SSA_2118	7.017544	3.947368	3.508772	5.263158	7.017544	3.070175	2.631579	5.263158	1.315789	1.754386	5.263158	11.40351	0	1.754386	4.385965	7.45614	6.140351	6.578947	8.77193	7.45614	H
SSA_0502	6.9869	2.183406	3.056769	5.676856	3.930131	5.676856	4.366812	11.35371	3.49345	2.620087	2.620087	14.41048	3.056769	0.436681	2.620087	5.676856	7.860262	3.930131	1.746725	8.296943	EP
SSA_2099	6.956522	3.913043	3.043478	5.652174	1.73913	6.521739	2.608696	8.695652	3.478261	0	3.913043	17.82609	2.608696	0.434783	3.478261	5.652174	6.956522	3.913043	1.73913	10.86957	E
SSA_0408	6.926407	1.731602	3.896104	7.792208	3.896104	8.658009	2.164502	9.090909	4.329004	1.298701	0.4329	16.01732	1.298701	1.298701	3.463203	10.82251	4.761905	0.865801	2.164502	9.090909	P
SSA_0204	6.896552	8.62069	1.724138	5.603448	5.603448	3.87931	1.724138	6.465517	3.87931	1.724138	3.448276	12.5	0.431034	2.155172	5.172414	4.310345	3.87931	11.63793	4.741379	5.603448	TK
SSA_1165	6.896552	6.896552	2.155172	6.034483	7.758621	6.896552	2.155172	7.758621	5.172414	2.155172	3.017241	7.327586	0.431034	0	4.741379	4.741379	5.603448	9.482759	5.172414	5.603448	K
SSA_1487	6.808511	4.255319	4.680851	6.808511	6.808511	3.829787	1.276596	6.808511	4.255319	0.851064	3.829787	8.93617	1.276596	1.276596	2.978723	4.680851	6.382979	11.48936	5.106383	7.659574	S
SSA_0504	6.779661	4.237288	1.271186	6.355932	8.050847	2.118644	1.694915	7.627119	5.508475	2.966102	3.813559	15.25424	0.423729	1.694915	2.542373	4.237288	8.050847	6.355932	6.779661	4.237288	EP
SSA_0203	6.666667	2.916667	2.083333	8.75	4.166667	3.333333	2.5	5.833333	2.916667	0.833333	2.083333	22.08333	2.5	0.416667	4.166667	9.166667	5.833333	2.5	2.083333	9.166667	
SSA_0818	6.584362	7.81893	4.115226	5.349794	8.641975	4.938272	3.703704	6.17284	5.761317	0.411523	2.880658	7.81893	0.411523	0	6.995885	2.469136	4.526749	5.761317	9.876543	5.761317	P
SSA_0687	6.477733	5.263158	2.42915	8.097166	5.263158	4.048583	1.619433	8.906883	3.643725	2.024291	4.453441	13.76518	4.453441	2.024291	3.643725	7.692308	4.8583	5.668016	3.238866	2.42915	
SSA_2080	6.477733	5.668016	5.263158	6.882591	6.882591	2.024291	1.619433	5.668016	3.238866	0.809717	2.42915	14.5749	0.809717	0.809717	2.42915	4.453441	5.263158	10.12146	6.072874	8.502024	G
SSA_1997	6.425703	2.409639	2.409639	7.228916	7.228916	4.417671	1.204819	8.032129	1.204819	2.008032	2.811245	21.28514	1.204819	0	2.811245	10.44177	5.220884	2.409639	2.409639	8.835341	
SSA_2192	6.425703	2.811245	6.425703	14.45783	6.425703	8.433735	2.008032	1.606426	11.64659	0	3.614458	4.417671	0.401606	0.803213	2.811245	1.204819	6.827309	2.409639	3.614458	13.65462	U
SSA_1701	6.374502	4.780876	3.585657	6.374502	7.968127	8.76494	3.984064	8.366534	3.984064	1.593625	1.593625	8.76494	0	0.796813	2.390438	3.984064	5.976096	10.75697	4.780876	5.179283	KG
SSA_0672	6.225681	4.280156	4.669261	5.058366	4.669261	3.501946	1.55642	5.058366	7.003891	2.33463	3.891051	12.06226	0	1.55642	4.669261	7.003891	5.447471	5.836576	7.782101	7.392996	R
SSA_0983	6.177606	4.633205	3.088803	5.405405	8.494208	3.088803	3.474903	7.335907	4.247104	1.158301	3.861004	6.949807	1.930502	1.930502	3.474903	2.702703	10.42471	8.494208	6.949807	6.177606	J
SSA_1546	6.130268	6.896552	2.298851	3.831418	3.448276	1.149425	2.681992	14.94253	2.681992	0.766284	4.214559	9.961686	2.681992	0.766284	6.896552	5.747126	8.429119	3.448276	3.831418	9.195402	M
SSA_2014	6.10687	4.198473	3.435115	9.923664	7.633588	3.816794	1.145038	5.343511	6.870229	1.526718	3.053435	11.0687	0.763359	0.381679	3.435115	1.908397	6.48855	9.160305	4.198473	9.541985	M
SSA_0867	6.08365	6.844106	3.802281	7.604563	5.323194	2.661597	0.760456	2.281369	6.844106	3.422053	3.041825	14.44867	1.520913	1.140684	5.323194	5.703422	3.422053	7.224335	5.703422	6.844106	
SSA_1098	6.037736	1.886792	5.660377	6.415094	6.037736	4.528302	2.264151	9.056604	0.754717	0.754717	1.886792	12.07547	1.886792	0.754717	2.641509	10.18868	7.54717	3.773585	2.264151	13.58491	P
SSA_0277	5.992509	3.370787	4.494382	4.494382	7.490637	5.992509	1.872659	8.2397	4.11985	4.11985	3.745318	8.2397	0.374532	1.498127	3.370787	7.490637	5.992509	8.988764	5.243446	4.868914	O
SSA_0934	5.992509	4.11985	2.621723	5.243446	8.2397	6.741573	3.370787	6.741573	3.745318	2.621723	2.996255	11.98502	0.749064	1.123596	4.11985	4.11985	7.116105	5.617978	5.617978	7.116105	R
SSA_1074	5.992509	4.494382	3.745318	7.116105	3.745318	3.370787	1.872659	4.868914	5.617978	1.123596	2.996255	14.60674	0.374532	0.749064	1.498127	2.996255	7.865169	8.988764	3.745318	14.23221	E
SSA_0939	5.904059	4.797048	3.690037	4.059041	7.01107	3.321033	2.95203	5.166052	5.166052	1.476015	2.95203	10.3321	1.476015	1.107011	4.428044	6.273063	7.380074	4.059041	8.487085	9.9631	G
SSA_0957	5.904059	4.797048	4.059041	9.9631	7.01107	3.321033	2.214022	8.487085	2.214022	2.214022	2.583026	11.43911	0.738007	1.107011	3.690037	5.904059	5.904059	6.642066	4.428044	7.380074	R
SSA_1169	5.904059	5.535055	7.380074	7.01107	8.856089	2.95203	2.95203	7.01107	4.428044	5.904059	4.059041	11.43911	0.369004	0	2.583026	5.166052	6.642066	2.95203	3.690037	5.166052	R

SSA_2139	5.904059	4.059041	2.214022	5.904059	9.225092	6.642066	2.95203	5.166052	5.535055	1.107011	4.059041	14.02214	1.476015	0.369004	2.95203	8.118081	5.166052	4.797048	1.845018	8.487085	U
SSA_2189	5.882353	3.676471	2.941176	6.985294	3.308824	8.455882	1.470588	4.411765	2.941176	1.470588	3.308824	13.60294	1.470588	1.470588	3.676471	2.573529	8.823529	5.882353	6.617647	11.02941	O
SSA_1153	5.797101	3.985507	3.623188	7.246377	6.15942	2.898551	1.449275	6.15942	5.797101	1.086957	3.26087	13.4058	0.362319	0.724638	3.26087	3.26087	5.797101	9.057971	7.246377	9.42029	J
SSA_1462	5.776173	2.888087	2.166065	5.054152	6.498195	5.415162	1.083032	7.942238	6.137184	2.888087	5.054152	8.66426	0.722022	0	5.054152	5.054152	4.33213	8.303249	4.693141	12.27437	E
SSA_0603	5.755396	6.115108	3.23741	7.553957	4.316547	4.316547	2.877698	6.47482	3.956835	2.158273	2.158273	16.90647	1.438849	0.719424	5.035971	7.553957	5.035971	2.877698	2.158273	9.352518	P
SSA_1300	5.755396	2.517986	4.316547	5.035971	6.115108	7.913669	3.956835	10.79137	1.798561	0.359712	3.956835	12.94964	1.798561	0.359712	5.395683	8.992806	5.755396	1.438849	2.517986	8.273381	G
SSA_2348	5.69395	3.914591	2.491103	4.626335	6.761566	2.846975	2.491103	6.049822	5.69395	3.558719	4.982206	12.81139	0.711744	1.067616	5.338078	5.69395	6.761566	6.405694	4.982206	7.117438	R
SSA_2182	5.614035	4.561404	4.210526	7.719298	8.421053	5.263158	1.052632	6.315789	8.070175	0.350877	2.807018	8.77193	1.403509	0	3.859649	3.508772	9.122807	7.368421	7.719298	3.859649	
SSA_0797	5.574913	4.529617	3.135889	5.226481	8.013937	1.393728	3.832753	4.878049	6.968641	3.135889	1.045296	11.49826	0.696864	1.393728	7.317073	6.271777	5.226481	8.362369	5.923345	5.574913	
SSA_1129	5.517241	2.068966	4.482759	6.551724	10.34483	8.62069	2.413793	5.172414	3.448276	1.034483	1.724138	10.34483	0.689655	0.344828	2.758621	3.793103	5.517241	11.72414	7.241379	6.206897	P
SSA_0295	5.498282	3.780069	2.749141	6.872852	7.216495	2.749141	1.030928	8.591065	7.216495	4.810997	5.154639	13.7457	0	0.343643	2.405498	6.52921	3.436426	7.560137	5.841924	4.467354	K
SSA_2101	5.442177	1.360544	1.70068	7.142857	11.90476	6.122449	0.340136	6.462585	2.721088	1.360544	3.401361	6.122449	1.70068	0	6.462585	4.421769	7.482993	8.503401	7.142857	10.20408	ET
SSA_0667	5.351171	7.023411	1.003344	6.688963	8.026756	3.67893	1.672241	5.016722	5.016722	1.003344	2.341137	14.04682	1.337793	1.337793	3.344482	6.688963	4.682274	10.36789	6.020067	5.351171	K
SSA_2056	5.194805	2.597403	2.272727	7.142857	6.818182	6.818182	2.272727	6.168831	5.194805	2.272727	5.519481	9.74026	0.649351	0.324675	5.194805	4.545455	5.519481	5.844156	6.818182	9.090909	I
SSA_1518	5.079365	2.857143	7.301587	6.349206	9.52381	4.444444	1.904762	9.206349	3.492063	0.31746	1.904762	11.11111	0.634921	1.587302	6.666667	6.349206	5.396825	5.396825	6.031746	4.444444	M
SSA_0274	4.923077	4.615385	5.538462	7.076923	6.769231	4	0.615385	6.461538	10.46154	2.153846	4.923077	7.692308	2.153846	2.153846	6.153846	6.153846	5.846154	4.615385	3.692308	4	L
SSA_1744	4.790419	3.892216	3.293413	8.682635	3.892216	6.586826	2.694611	9.88024	2.694611	1.796407	2.994012	18.56287	0.898204	0.299401	2.694611	5.389222	8.682635	2.095808	2.694611	7.48503	P
SSA_2083	4.719764	4.424779	5.309735	4.129794	5.014749	4.719764	1.474926	7.374631	2.949853	3.539823	5.014749	9.439528	1.474926	0.589971	4.719764	5.309735	6.784661	5.899705	8.554572	8.554572	R
SSA_2373	4.395604	4.67033	5.21978	6.868132	8.241758	6.043956	1.373626	9.065934	6.593407	3.296703	2.747253	14.01099	0.549451	0.274725	2.472527	4.67033	4.120879	4.945055	7.417582	3.021978	L
SSA_0230	4.199475	2.099738	4.986877	7.086614	4.199475	6.036745	2.887139	11.81102	2.624672	2.624672	2.099738	14.17323	1.574803	0.524934	6.56168	10.76115	4.461942	2.624672	2.887139	5.774278	S
SSA_2237	3.902439	5.853659	3.902439	6.585366	2.682927	5.365854	1.95122	6.829268	7.317073	1.95122	2.682927	13.41463	0.731707	0.731707	3.170732	4.878049	8.536585	7.804878	3.902439	7.804878	H
SSA_0451	13.17829	2.325581	2.325581	7.751938	6.20155	3.875969	3.100775	6.976744	1.550388	1.550388	3.875969	8.527132	0	0	3.100775	3.100775	5.426357	7.751938	7.751938	11.62791	S
SSA_1699	11.88811	3.496503	5.594406	2.797203	7.692308	3.496503	6.293706	6.993007	2.097902	1.398601	0.699301	4.895105	0	0.699301	2.097902	3.496503	11.18881	6.993007	6.993007	11.18881	G
SSA_1497	10.96774	4.516129	4.516129	3.225806	7.096774	5.16129	0.645161	5.16129	5.16129	3.870968	1.935484	9.677419	0.645161	4.516129	5.16129	1.935484	5.806452	5.16129	5.806452	9.032258	F
SSA_1969	10.75949	11.39241	3.797468	6.962025	3.797468	7.594937	1.898734	12.02532	4.43038	0.632911	3.797468	6.962025	0	0	0.632911	1.898734	4.43038	6.329114	5.063291	7.594937	E
SSA_1796	10.625	3.125	1.875	6.875	10	6.25	1.25	7.5	2.5	0	2.5	5.625	0	0	3.75	1.25	6.875	16.25	4.375	9.375	K
SSA_2154	10.30303	4.848485	3.030303	4.848485	4.242424	6.666667	3.636364	4.848485	4.242424	4.242424	3.030303	9.69697	0	1.212121	2.424242	3.636364	6.060606	6.666667	7.878788	8.484848	P
SSA_1386	9.340659	2.747253	4.945055	7.692308	4.945055	3.296703	1.098901	4.395604	3.296703	1.098901	6.043956	10.98901	2.197802	0	2.197802	3.846154	5.494505	6.043956	5.494505	14.83516	R
SSA_1862	9.139785	4.301075	4.301075	5.913978	6.451613	3.763441	3.763441	4.301075	4.83871	2.150538	3.225806	8.602151	2.688172	1.075269	4.83871	3.225806	6.451613	6.451613	5.913978	8.602151	J
SSA_0522	9.090909	4.278075	3.208556	5.347594	11.22995	6.417112	1.069519	5.347594	2.673797	1.069519	3.208556	11.76471	0	0	2.673797	1.069519	4.278075	11.76471	3.743316	11.76471	QC
SSA_0301	8.900524	0.52356	9.424084	6.806283	12.56545	7.329843	1.04712	7.329843	3.664921	1.570681	2.617801	6.806283	0.52356	0	3.141361	5.235602	7.853403	5.759162	4.188482	4.712042	
SSA_1034	8.717949	3.589744	4.102564	6.153846	8.205128	3.076923	2.564103	6.666667	4.102564	3.589744	2.564103	9.230769	0	1.025641	3.589744	3.589744	8.205128	7.179487	5.641026	8.205128	J
SSA_1972	8.542714	5.025126	4.020101	5.025126	6.532663	5.527638	3.517588	5.025126	6.532663	0.502513	3.015075	13.06533	1.005025	0.502513	3.015075	1.005025	4.020101	8.542714	6.030151	9.547739	TK
SSA_1134	8.415842	3.465347	1.980198	4.455446	7.920792	5.940594	2.475248	10.39604	4.950495	0.49505	0.990099	8.415842	0	0	1.485149	5.445545	8.910891	7.920792	5.445545	10.89109	F
SSA_1444	8.21256	4.347826	2.415459	5.797101	1.932367	2.898551	3.381643	5.31401	6.280193	1.932367	5.797101	12.07729	0.483092	1.449275	4.347826	2.415459	10.62802	6.280193	4.830918	9.178744	E
SSA_0468	8.095238	3.809524	1.428571	6.666667	5.714286	1.428571	3.333333	7.619048	2.857143	0.952381	5.714286	11.90476	0.47619	0	5.714286	2.857143	8.571429	9.52381	6.190476	7.142857	H
SSA_1172	8.018868	2.830189	6.132075	6.132075	9.433962	2.830189	2.830189	4.245283	3.301887	1.415094	6.132075	9.433962	0.943396	0.943396	4.245283	6.603774	4.716981	8.962264	5.188679	5.660377	
SSA_0528	7.906977	4.186047	1.395349	5.581395	11.16279	3.255814	1.860465	6.046512	5.581395	1.395349	3.72093	13.95349	0	0.930233	2.790698	2.790698	5.581395	11.16279	4.186047	6.511628	G
SSA_0521	7.762557	2.739726	4.56621	8.675799	2.739726	2.739726	1.369863	5.022831	0.913242	1.369863	4.109589	8.675799	0	1.826484	4.56621	2.283105	8.675799	5.936073	6.849315	19.17808	E
SSA_0304	7.692308	0.904977	9.502262	5.429864	2.714932	8.144796	1.809955	4.524887	4.977376	0.904977	4.977376	4.072398	2.714932	0.452489	6.78733	3.167421	9.502262	3.167421	4.977376	13.57466	R
SSA_2361	7.623318	3.587444	6.726457	8.071749	4.932735	7.623318	3.139013	13.00448	2.690583	4.035874	4.484305	4.484305	0.44843	0.44843	0.896861	4.035874	7.174888	5.829596	4.932735	5.829596	E
SSA_2170	7.488987	2.643172	3.0837	7.929515	1.762115	3.964758	3.524229	11.45374	3.524229	2.202643	3.0837	14.09692	0.881057	0.440529	4.845815	9.69163	8.370044	1.762115	1.762115	7.488987	R
SSA_0316	7.45614	3.508772	3.508772	5.701754	3.947368	6.140351	3.070175	5.701754	5.263158	2.192982	5.263158	12.7193	0.438596	0	2.192982	2.631579	4.385965	6.578947	5.263158	14.03509	O
SSA_0696	7.45614	2.631579	3.508772	7.894737	6.578947	3.070175	2.631579	6.578947	2.192982	2.192982	5.701754	15.78947	3.070175	0.438596	6.578947	9.210526	4.385965	0.438596	3.070175	6.578947	
SSA_2015	7.327586	6.465517	2.586207	4.741379	3.017241	6.465517	3.448276	5.603448	1.724138	2.586207	1.724138	8.189655	2.155172	0.862069	3.017241	4.310345	12.93103	10.34483	5.172414	7.327586	G
SSA_1516	7.264957	6.410256	2.991453	7.264957	5.982906	4.700855	2.991453	11.53846	2.991453	2.136752	2.991453	6.837607	0	0.42735	5.128205	4.273504	5.982906	6.410256	6.837607	6.837607	R
SSA_0680	7.024793	4.132231	4.132231	3.305785	4.545455	5.785124	2.479339	4.545455	3.719008	4.132231	2.892562	10.7438	2.066116	0.826446	3.719008	4.132231	10.7438	7.024793	8.264463	5.785124	T
SSA_2059	7.024793	4.958678	2.066116	2.892562	8.677686	5.785124	2.066116	4.132231	5.785124	2.066116	2.892562	15.70248	0.413223	0.826446	1.652893	2.479339	6.61157	9.504132	5.785124	8.677686	J
SSA_0582	6.967213	2.868852	1.639344	5.737705	4.918033	5.327869	2.459016	5.327869	4.508197	3.278689	2.459016	7.786885	1.229508	1.229508	5.737705	7.377049	5.737705	9.836066	9.836066	5.737705	H
SSA_1264	6.938776	2.857143	3.673469	6.530612	6.938776	2.44898	2.040816	5.306122	5.306122	1.632653	2.857143	15.10204	1.632653	2.040816	5.306122	2.857143	5.714286	7.346939	5.714286	7.755102	R
SSA_1790	6.910569	4.065041	2.845528	10.56911	4.878049	3.658537	2.439024	8.130081	2.845528	2.845528	2.845528	9.349593	0.406504	0	2.845528	4.878049	6.097561	7.317073	6.097561	10.97561	J
SSA_1962	6.910569	4.065041	5.284553	6.910569	8.536585	6.097561	3.658537	6.910569	4.878049	1.219512	2.845528	9.756098	0.406504	0	2.03252	4.471545	6.910569	10.1626	3.658537	5.284553	E
SSA_1867	6.882591	5.263158	2.42915	8.906883	8.906883	4.048583	2.024291	6.477733	4.048583	2.42915	2.834008	12.14575	0	0	1.619433	4.453441	6.072874	7.287449	6.882591	7.287449	E
SSA_0707	6.854839	4.032258	7.258065	6.451613	9.274194	6.048387	2.016129	9.274194	2.822581	2.016129	0.806452	8.870968	0	0.403226	3.225806	3.629032	7.258065	6.451613	7.258065	6.048387	KG
SSA_0262	6.827309	2.409639	2.409639	6.024096	8.032129	5.220884	2.811245	10.04016	6.425703	2.409639	3.212851	12.4498	0.401606	1.204819	3.212851	3.614458	7.228916	5.62249	5.62249	4.819277	P
SSA_1660	6.719368	5.13834	3.557312	6.719368	7.509881	7.905138	3.162055	7.114625	5.13834	1.185771	2.371542	11.06719	0	0.395257	1.581028	4.743083	5.928854	5.928854	6.719368	7.114625	V
SSA_1077	6.640625	2.734375	4.6875	1.171875	5.46875	9.765625	1.953125	8.984375	2.34375	1.953125	5.859375	9.765625	0.78125	0	4.296875	2.734375	7.8125	8.984375	5.46875	8.59375	G
SSA_0945	6.614786	4.280156	4.669261	7.003891	8.171206	7.392996	3.501946	7.392996	3.11284	1.167315	4.669261	9.727626	0.389105	0.389105	3.501946	3.11284	7.392996	7.392996	5.058366	5.058366	P
SSA_0341	6.589147	3.875969	6.20155	6.976744	4.263566	6.20155	3.875969	8.914729	2.325581	1.162791	2.325581	10.46512	0.387597	0.775194	2.713178	7.364341	6.589147	4.651163	4.263566	10.07752	S
SSA_1740	6.51341	3.831418	2.681992	6.130268	5.363985	6.896552	4.597701	5.363985	1.915709	1.149425	3.065134	15.70881	3.065134	0	6.51341	9.961686	5.363985	3.448276	2.681992	5.747126	
SSA_0566	6.48855	6.870229	5.725191	5.343511	5.343511	6.48855	1.908397	9.923664	3.053435	1.145038	2.671756	10.30534	0.381679	0.763359	3.053435	3.435115	4.961832	8.396947	6.870229	6.870229	K
SSA_0346	6.439394	1.893939	3.409091	4.545455	4.545455	6.818182	2.272727	6.060606	3.787879	2.272727	5.30303	14.01515	4.924242	2.272727	2.272727	8.333333	9.090909	2.272727	0.757576	8.712121	
SSA_1905	6.415094	3.396226	3.396226	4.150943	7.54717	4.90566	3.396226	8.679245	3.396226	1.509434	4.528302	12.45283	0.377358	1.132075	2.641509	5.283019	6.792453	8.679245	5.660377	5.660377	V

SSA_0844	6.137184	2.527076	3.610108	7.942238	4.693141	4.693141	3.249097	11.91336	1.805054	1.083032	3.249097	15.52347	2.166065	1.083032	5.776173	11.19134	5.415162	2.527076	1.805054	3.610108	
SSA_0022	6.071429	6.785714	2.857143	8.571429	7.5	6.785714	2.142857	3.928571	3.928571	2.142857	3.214286	7.857143	0.357143	0.714286	6.428571	5.357143	3.928571	7.857143	8.214286	5.357143	S
SSA_1158	5.985915	1.760563	7.746479	5.985915	8.098592	5.28169	2.816901	5.28169	7.042254	2.464789	2.816901	11.26761	0.352113	0	4.225352	3.521127	7.746479	6.338028	4.929577	6.338028	R
SSA_2124	5.985915	3.873239	3.873239	7.746479	11.26761	3.873239	3.169014	5.985915	5.28169	1.056338	1.760563	11.26761	0.352113	1.056338	5.28169	3.521127	4.929577	8.450704	4.225352	7.042254	IQR
SSA_0793	5.944056	4.895105	6.993007	9.090909	6.643357	6.293706	0.34965	4.195804	6.993007	1.398601	3.146853	8.391608	2.097902	0	3.496503	2.447552	9.090909	4.545455	4.545455	9.440559	
SSA_1718	5.944056	6.293706	8.041958	6.643357	5.944056	4.895105	1.748252	5.244755	3.146853	0.699301	3.846154	9.440559	0.34965	0	7.342657	5.944056	5.594406	8.391608	5.594406	4.895105	L
SSA_1595	5.923345	4.878049	3.832753	3.484321	5.226481	5.574913	3.135889	3.832753	4.529617	2.787456	4.878049	10.45296	1.74216	1.393728	3.832753	4.529617	5.923345	11.14983	5.226481	7.665505	P
SSA_2089	5.923345	4.529617	2.787456	5.574913	6.271777	3.484321	2.787456	5.574913	4.529617	1.74216	4.529617	9.407666	2.090592	1.74216	4.529617	4.878049	4.878049	10.80139	5.226481	8.710801	G
SSA_1719	5.882353	3.460208	3.114187	6.228374	6.920415	4.49827	2.076125	7.612457	5.190311	3.460208	4.844291	10.72664	0	0.346021	3.806228	2.768166	6.574394	8.304498	4.844291	9.342561	R
SSA_1975	5.841924	3.436426	3.436426	6.52921	7.560137	7.560137	1.718213	7.216495	7.216495	2.405498	2.749141	12.37113	0.687285	1.030928	2.061856	4.123711	5.154639	8.934708	5.498282	4.467354	V
SSA_1458	5.821918	3.082192	5.479452	5.479452	8.561644	2.39726	2.39726	9.589041	4.109589	1.712329	3.424658	6.164384	3.082192	1.369863	5.136986	5.479452	5.479452	8.561644	9.246575	3.424658	R
SSA_2176	5.743243	5.743243	4.054054	8.783784	9.121622	4.054054	0.675676	3.716216	7.77027	1.351351	1.689189	11.48649	1.351351	0	5.067568	5.067568	6.756757	9.459459	5.405405	2.702703	
SSA_0409	5.629139	3.311258	5.298013	5.629139	7.94702	5.298013	1.655629	9.271523	7.94702	2.317881	1.986755	11.92053	0.331126	0.331126	4.304636	2.980132	6.622517	5.298013	6.291391	5.629139	V
SSA_0407	5.483871	3.870968	4.516129	5.16129	8.709677	5.483871	1.290323	10.64516	5.483871	2.903226	2.580645	10.64516	0.322581	0.322581	4.516129	2.580645	9.032258	6.774194	4.83871	4.83871	V
SSA_1271	5.466238	4.501608	2.250804	5.787781	5.144695	5.466238	2.572347	8.038585	5.144695	3.215434	3.536977	9.003215	0.643087	0.321543	4.823151	2.893891	6.109325	8.038585	8.038585	9.003215	R
SSA_0292	5.246914	3.395062	3.703704	5.864198	6.17284	8.333333	3.08642	5.864198	4.320988	1.54321	3.703704	12.34568	0.617284	0.308642	4.62963	5.555556	5.555556	7.716049	5.246914	6.790123	K
SSA_1448	5.167173	5.775076	1.519757	6.079027	4.863222	5.775076	1.519757	5.775076	6.079027	2.735562	3.343465	11.8541	0.303951	0	3.951368	6.079027	6.68693	7.294833	7.902736	7.294833	E
SSA_1989	5.151515	4.848485	3.030303	5.151515	7.575758	6.969697	2.727273	8.787879	5.757576	2.121212	3.333333	10.60606	0.606061	0.606061	1.818182	5.757576	6.363636	6.969697	8.484848	3.333333	R
SSA_2352	5.105105	0.600601	5.405405	7.807808	12.01201	3.003003	2.402402	7.207207	3.603604	1.201201	3.603604	6.306306	2.102102	0.3003	5.105105	3.903904	6.906907	5.705706	9.009009	8.708709	P
SSA_2179	5.04451	0.593472	4.747774	11.27596	5.341246	3.857567	2.373887	3.264095	8.902077	1.186944	7.418398	8.011869	0.890208	0	3.264095	4.747774	10.68249	8.308605	4.747774	5.341246	
SSA_1231	4.607046	4.878049	2.98103	7.04607	7.859079	3.794038	1.897019	13.82114	2.98103	1.626016	1.897019	15.71816	2.710027	1.626016	5.420054	7.588076	2.98103	4.607046	3.252033	2.710027	
SSA_0104	4.473684	6.052632	4.473684	6.578947	4.473684	6.315789	4.210526	5.789474	3.947368	2.894737	5	8.684211	0.789474	1.578947	3.947368	5.263158	8.157895	6.052632	6.315789	5	J
SSA_0917	4.392765	3.617571	3.100775	4.651163	7.49354	5.426357	0.775194	5.684755	4.134367	3.100775	2.067183	9.819121	2.067183	1.033592	4.909561	4.134367	7.49354	9.302326	8.268734	8.527132	E
SSA_0932	4.018913	4.491726	5.437352	5.437352	10.16548	4.964539	2.364066	4.728132	8.037825	1.182033	1.182033	10.40189	0	0.945626	3.073286	4.491726	2.600473	13.71158	4.964539	7.801418	S
SSA_1061	17.30769	1.923077	3.846154	2.884615	13.46154	7.692308	0.961538	4.807692	6.730769	1.923077	2.884615	2.884615	0	0	4.807692	0.961538	11.53846	5.769231	1.923077	7.692308	J
SSA_1979	14.87603	0.826446	6.61157	4.132231	9.917355	6.61157	1.652893	9.917355	3.305785	0.826446	0	4.958678	0	0	3.305785	2.479339	9.090909	6.61157	5.785124	9.090909	S
SSA_0725	11.18012	1.863354	2.484472	5.590062	4.968944	2.484472	1.863354	11.80124	3.10559	1.242236	6.21118	16.14907	2.484472	0.621118	5.590062	7.453416	4.968944	1.863354	1.863354	6.21118	
SSA_0708	10.16949	2.259887	4.519774	3.954802	7.344633	1.694915	3.954802	6.779661	1.694915	2.824859	3.389831	19.20904	1.129944	1.129944	4.519774	7.909605	4.519774	5.084746	2.259887	5.649718	S
SSA_1901	10.16949	6.214689	1.129944	5.084746	7.909605	4.519774	1.694915	6.214689	2.259887	1.694915	5.649718	11.86441	0	0.564972	5.084746	3.389831	3.954802	10.16949	6.779661	5.649718	S
SSA_0636	9.944751	3.867403	1.657459	4.972376	4.41989	2.762431	0.552486	5.524862	8.287293	1.657459	3.867403	8.287293	1.104972	0.552486	2.209945	6.077348	9.392265	6.077348	6.629834	12.1547	E
SSA_2079	9.89011	4.395604	7.692308	5.494505	4.395604	4.395604	1.098901	6.593407	3.296703	1.648352	4.945055	5.494505	1.098901	2.197802	3.846154	4.395604	10.43956	4.945055	6.043956	7.692308	R
SSA_1773	9.72973	2.702703	4.324324	5.945946	7.027027	4.324324	2.702703	9.189189	0.540541	0	3.243243	21.62162	1.621622	0	4.324324	5.945946	3.783784	2.162162	2.162162	8.648649	S
SSA_0033	9.625668	4.278075	2.139037	5.347594	5.347594	2.673797	1.069519	7.486631	3.743316	3.208556	3.743316	7.486631	1.069519	0.534759	4.278075	4.812834	7.486631	9.625668	6.417112	9.625668	F
SSA_1203	9.625668	0.534759	3.743316	8.02139	6.417112	4.812834	2.673797	14.97326	1.604278	1.069519	5.882353	13.90374	0	0.534759	2.673797	5.347594	6.951872	1.069519	0.534759	9.625668	S
SSA_1157	9	3	6	5.5	8	6	3.5	5	5.5	2	1	9.5	1	0	7.5	3.5	6.5	6	5	6.5	M
SSA_1757	8.910891	5.445545	4.950495	8.415842	5.445545	2.475248	2.475248	5.445545	4.455446	1.980198	2.475248	9.405941	0.49505	0.49505	5.940594	5.445545	8.415842	9.405941	4.455446	3.465347	
SSA_0994	8.737864	1.456311	4.368932	4.368932	8.252427	4.368932	1.456311	4.368932	6.31068	0.970874	1.456311	12.62136	0	0.970874	4.854369	4.854369	6.796117	8.737864	6.796117	8.252427	E
SSA_0093	8.695652	8.21256	4.830918	5.31401	7.246377	4.830918	6.280193	4.830918	2.415459	2.898551	2.898551	11.11111	0	0.483092	2.415459	1.449275	1.932367	11.5942	6.280193	6.280193	C
SSA_0366	8.653846	1.923077	5.288462	8.173077	9.615385	7.692308	1.442308	5.769231	3.365385	2.403846	2.403846	10.09615	0.961538	0	3.846154	3.846154	6.25	8.173077	5.769231	4.326923	
SSA_1842	8.571429	3.809524	2.857143	5.238095	8.095238	3.809524	2.857143	8.095238	2.857143	3.809524	2.380952	11.90476	0.47619	0	3.333333	1.904762	4.285714	11.42857	5.714286	8.571429	TK
SSA_1447	8.411215	5.140187	2.803738	7.009346	6.542056	5.607477	1.869159	7.943925	2.336449	2.336449	4.205607	10.28037	0	0.934579	2.336449	5.607477	7.009346	5.140187	7.476636	7.009346	E
SSA_1772	8.333333	3.703704	1.851852	5.555556	7.87037	5.092593	2.777778	8.796296	5.092593	1.851852	3.703704	15.27778	1.851852	0	4.166667	6.944444	6.944444	1.851852	2.314815	6.018519	I
SSA_0896	8.256881	4.12844	3.211009	4.587156	7.798165	7.33945	3.211009	8.715596	4.12844	1.834862	4.12844	11.46789	0.917431	0.917431	3.211009	2.752294	5.504587	7.798165	7.33945	2.752294	TK
SSA_1981	8.181818	3.181818	6.363636	7.727273	4.545455	3.636364	2.727273	8.181818	0.909091	0.454545	2.272727	9.090909	1.363636	0.909091	4.090909	9.545455	8.636364	3.181818	5	10	
SSA_2090	8.144796	4.072398	2.262443	4.072398	6.334842	5.882353	1.809955	7.692308	4.977376	1.357466	1.809955	8.144796	2.262443	1.809955	1.357466	2.714932	9.049774	7.692308	6.334842	12.21719	G
SSA_0657	8.071749	3.587444	5.381166	4.932735	9.41704	3.587444	2.690583	6.278027	6.278027	4.035874	1.793722	10.3139	0.44843	0.896861	2.690583	4.484305	3.587444	10.3139	4.484305	6.726457	R
SSA_0479	8.035714	5.357143	3.125	6.25	4.017857	4.464286	2.232143	5.803571	5.803571	2.232143	2.678571	20.08929	1.339286	0.446429	6.25	8.035714	2.678571	2.232143	3.125	5.803571	P
SSA_0217	7.929515	4.405286	3.0837	5.726872	7.048458	6.167401	3.0837	11.45374	4.845815	1.321586	1.762115	8.370044	0.881057	0.440529	2.202643	3.964758	6.167401	7.048458	6.60793	7.488987	TK
SSA_2155	7.792208	3.030303	6.493506	9.090909	2.597403	6.926407	3.030303	4.329004	8.225108	1.731602	2.597403	9.52381	0.865801	0	3.030303	5.627706	11.68831	4.329004	2.597403	6.493506	S
SSA_1113	7.758621	5.603448	3.448276	4.741379	6.465517	5.172414	3.448276	7.327586	3.448276	2.155172	3.017241	11.63793	1.293103	0.431034	3.448276	4.310345	6.465517	7.758621	7.758621	4.310345	TK
SSA_1256	7.346939	3.265306	2.857143	6.530612	6.122449	3.673469	1.632653	6.122449	5.714286	3.265306	3.265306	11.02041	0	1.632653	4.489796	4.897959	7.346939	6.122449	6.530612	8.163265	K
SSA_1076	7.317073	4.878049	4.065041	6.097561	6.097561	4.065041	5.284553	6.910569	4.065041	2.439024	4.065041	8.943089	1.219512	0.406504	4.878049	4.471545	4.878049	6.504065	8.536585	4.878049	I
SSA_2077	7.317073	3.252033	4.471545	7.317073	4.065041	4.878049	2.439024	10.56911	2.845528	2.845528	2.845528	9.756098	4.878049	0.406504	7.317073	10.56911	4.878049	2.439024	2.439024	4.471545	R
SSA_0242	7.2	4	1.6	5.6	8	4	1.2	5.2	4.8	0.4	4	12.4	1.2	0.4	1.2	4.8	7.6	7.6	6.4	12.4	S
SSA_0495	7.03125	6.25	1.5625	8.984375	5.078125	3.90625	1.171875	6.640625	8.984375	1.5625	2.734375	12.10938	0	1.5625	3.90625	2.734375	5.859375	6.640625	7.03125	6.25	R
SSA_1956	7.03125	3.515625	4.6875	3.515625	7.03125	4.296875	5.859375	6.25	1.5625	1.171875	2.734375	12.10938	0	0	2.734375	3.125	9.765625	12.89063	4.296875	7.421875	O
SSA_1681	6.949807	5.019305	4.247104	6.563707	6.949807	8.108108	3.088803	6.949807	5.405405	1.158301	2.316602	10.81081	0	0.3861	1.930502	4.633205	6.177606	6.177606	6.177606	6.949807	V
SSA_1416	6.792453	3.018868	2.264151	4.528302	6.037736	5.283019	1.886792	9.433962	4.90566	3.018868	2.264151	9.811321	1.509434	1.132075	3.018868	3.396226	7.169811	8.301887	6.792453	9.433962	S
SSA_1558	6.617647	2.941176	4.044118	4.779412	5.882353	5.882353	2.573529	6.25	4.411765	1.470588	2.941176	10.66176	1.102941	1.102941	3.308824	3.308824	5.147059	8.823529	8.823529	9.926471	R
SSA_1181	6.569343	4.744526	5.109489	6.934307	7.29927	4.014599	2.554745	8.394161	6.20438	2.919708	2.919708	5.839416	1.824818	0.729927	5.474453	5.109489	6.20438	6.20438	5.474453	5.474453	R
SSA_0588	6.545455	2.545455	5.090909	8.363636	10.90909	6.545455	0.363636	6.909091	4.363636	0.363636	2.909091	8	0.363636	0.363636	5.818182	3.272727	6.909091	7.636364	7.272727	5.454545	ET
SSA_1313	6.498195	3.971119	2.527076	8.66426	6.859206	3.249097	2.888087	5.415162	3.971119	2.166065	3.971119	8.66426	1.083032	0.722022	5.415162	5.054152	5.776173	8.66426	5.054152	9.386282	S
SSA_1695	6.498195	5.776173	4.693141	6.498195	6.137184	3.249097	1.805054	8.66426	4.693141	3.249097	3.249097	9.386282	0	0.361011	7.581227	3.249097	3.249097	7.942238	7.581227	6.137184	K

SSA_2011	6.451613	3.225806	2.508961	6.451613	8.602151	8.243728	2.508961	6.451613	3.225806	1.792115	2.508961	14.69534	0	0.716846	2.150538	3.584229	8.243728	8.960573	5.376344	4.301075	V
SSA_0673	6.428571	3.214286	2.857143	6.428571	4.285714	5.714286	2.857143	6.071429	7.142857	2.142857	4.285714	11.78571	0.714286	0	2.142857	3.571429	8.928571	5.714286	3.928571	11.78571	G
SSA_0418	6.382979	5.319149	2.12766	10.28369	4.609929	3.546099	1.41844	2.12766	8.156028	2.48227	3.191489	13.82979	1.06383	0.70922	4.255319	5.673759	8.156028	7.801418	2.836879	6.028369	K
SSA_1618	6.338028	5.28169	4.225352	4.577465	8.098592	5.633803	2.816901	5.28169	5.633803	0.704225	3.873239	10.91549	1.408451	0	3.521127	4.929577	8.450704	6.338028	6.690141	5.28169	S
SSA_2369	6.338028	3.169014	4.929577	14.78873	8.098592	6.690141	1.056338	5.28169	2.816901	0.352113	2.464789	10.56338	0.704225	0	3.873239	3.521127	4.929577	6.690141	4.577465	9.15493	S
SSA_1014	6.206897	7.586207	1.37931	4.482759	6.206897	5.862069	1.034483	5.172414	5.517241	1.37931	2.758621	15.17241	0.344828	1.724138	5.517241	3.103448	7.931034	5.517241	6.206897	6.896552	I
SSA_0402	6.185567	4.467354	2.749141	6.872852	5.841924	6.185567	2.405498	7.560137	7.90378	1.718213	2.061856	14.08935	0.343643	0.343643	2.749141	3.092784	4.467354	7.90378	5.498282	7.560137	T
SSA_0417	6.164384	4.109589	5.479452	4.452055	4.794521	5.479452	1.712329	8.561644	4.452055	4.109589	6.164384	9.246575	0	1.369863	3.424658	5.479452	3.424658	4.794521	7.534247	9.246575	E
SSA_0597	5.901639	3.278689	0.983607	6.557377	5.901639	4.590164	3.278689	5.245902	6.229508	1.639344	4.590164	10.81967	0.983607	0	6.557377	4.262295	7.540984	8.196721	6.885246	6.557377	
SSA_1033	5.882353	3.921569	7.51634	6.20915	6.862745	6.862745	1.30719	8.169935	4.575163	2.941176	3.594771	10.13072	0.980392	0	3.921569	6.535948	2.941176	8.169935	5.882353	3.594771	H
SSA_0260	5.825243	0.970874	6.472492	7.443366	12.62136	4.854369	1.618123	7.443366	1.618123	1.294498	3.883495	8.090615	0.970874	0.970874	4.530744	2.912621	5.177994	9.385113	6.796117	7.119741	P
SSA_1815	5.825243	2.588997	5.177994	9.061489	7.119741	5.501618	3.236246	10.03236	3.559871	1.618123	2.588997	9.708738	0.970874	0.647249	2.912621	5.177994	5.177994	8.737864	7.76699	2.588997	
SSA_0961	5.769231	3.205128	4.166667	8.012821	7.371795	3.846154	3.205128	6.730769	5.128205	3.205128	3.846154	12.82051	1.923077	0.961538	3.846154	3.525641	5.128205	5.128205	4.807692	7.371795	H
SSA_0464	5.696203	2.848101	1.265823	6.012658	4.746835	6.329114	2.848101	7.278481	3.164557	1.898734	4.113924	15.82278	2.848101	0.949367	5.063291	4.113924	7.911392	4.746835	3.481013	8.860759	H
SSA_1596	5.642633	1.880878	6.896552	9.090909	10.34483	3.761755	1.567398	6.269592	4.702194	0.940439	3.134796	11.28527	1.253918	0	8.15047	3.448276	5.329154	5.642633	5.329154	5.329154	
SSA_0279	5.572755	4.643963	4.024768	7.739938	7.120743	4.024768	2.47678	6.811146	3.405573	3.095975	1.23839	11.45511	0.928793	0.928793	4.024768	4.024768	6.501548	7.430341	6.501548	8.049536	K
SSA_1517	5.572755	2.786378	6.501548	8.049536	9.907121	2.786378	1.23839	10.52632	4.643963	0.619195	2.167183	10.52632	1.547988	0.619195	6.811146	5.263158	4.95356	6.19195	6.19195	3.095975	M
SSA_0874	5.521472	1.533742	1.840491	10.7362	4.601227	4.294479	2.453988	11.34969	3.374233	0.306748	3.374233	12.57669	1.533742	0.920245	3.067485	9.509202	7.668712	1.533742	1.533742	12.26994	S
SSA_1684	5.521472	3.067485	2.147239	7.055215	7.668712	3.680982	0.306748	5.828221	7.055215	2.147239	0.920245	17.79141	0.920245	0	5.521472	6.748466	4.907975	7.055215	5.521472	6.134969	T
SSA_0167	5.405405	0.900901	19.81982	4.504505	4.804805	4.504505	0.600601	1.801802	5.705706	0	18.31832	3.003003	0	0	0.3003	0.900901	13.51351	1.201201	6.906907	7.807808	S
SSA_0634	5.389222	3.892216	3.592814	7.784431	5.988024	5.988024	4.191617	7.48503	3.892216	1.796407	2.994012	11.37725	0	0.299401	1.796407	2.994012	9.580838	6.886228	4.191617	9.88024	E
SSA_1904	5.172414	5.45977	2.298851	3.735632	5.45977	4.022989	1.436782	5.45977	5.172414	1.149425	2.298851	21.55172	1.436782	0.862069	4.022989	10.34483	4.597701	3.16092	2.586207	9.770115	U
SSA_0343	5.084746	5.649718	3.107345	6.779661	9.887006	5.367232	1.977401	8.757062	2.542373	2.542373	3.389831	9.60452	0.282486	0.564972	3.954802	3.672316	6.779661	8.19209	5.367232	6.497175	L
SSA_1847	5.070423	5.352113	2.535211	6.760563	7.887324	3.943662	1.408451	8.450704	3.661972	2.535211	2.816901	11.26761	0.28169	1.408451	2.535211	4.788732	5.352113	9.295775	6.478873	8.169014	J
SSA_0541	4.511278	2.506266	4.761905	8.270677	6.015038	2.506266	2.255639	7.769424	5.764411	2.756892	2.506266	13.78446	0.501253	1.002506	2.506266	4.761905	7.518797	7.017544	5.012531	8.270677	C
SSA_1812	4.411765	5.147059	7.843137	8.088235	8.333333	5.637255	2.205882	8.578431	3.676471	1.960784	2.205882	10.78431	1.22549	0.490196	3.921569	4.166667	3.186275	6.862745	5.637255	5.637255	L
SSA_2295	4.275534	5.225653	6.413302	5.463183	11.40143	7.60095	1.900238	8.551069	1.662708	2.850356	3.087886	10.92637	1.425178	0.712589	3.325416	2.850356	5.463183	5.700713	5.700713	5.463183	L
SSA_0940	4.147465	3.917051	4.608295	5.760369	7.603687	5.069124	2.073733	4.147465	4.608295	1.382488	5.069124	10.59908	1.382488	0.921659	2.995392	6.221198	8.525346	7.834101	4.37788	8.75576	J
SSA_1401	4.100228	5.01139	3.189066	7.517084	7.744875	3.416856	1.366743	6.150342	7.061503	2.277904	3.872437	14.3508	0	0.683371	7.744875	3.644647	2.505695	7.517084	5.922551	5.922551	KE
SSA_1764	3.461538	3.076923	3.461538	5.576923	6.346154	6.346154	1.923077	8.846154	3.653846	1.730769	3.461538	16.73077	2.115385	0.769231	6.153846	8.461538	5.384615	3.076923	2.692308	6.730769	
SSA_1345	11.04651	9.302326	3.488372	1.744186	4.651163	8.72093	2.325581	12.2093	3.488372	2.325581	3.488372	6.976744	0	0	1.744186	0.581395	5.232558	7.55814	9.883721	5.232558	F
SSA_2205	10.67416	3.932584	7.865169	3.932584	6.179775	8.426966	3.932584	7.303371	4.494382	0.561798	2.808989	6.741573	1.123596	0	2.808989	4.494382	6.741573	9.550562	5.617978	2.808989	K
SSA_0450	10.21505	3.225806	3.225806	4.301075	6.451613	11.82796	3.225806	6.989247	3.763441	1.075269	4.301075	5.913978	0	0	4.301075	3.763441	6.989247	9.677419	4.83871	5.913978	J
SSA_1999	10.16043	3.208556	1.604278	3.208556	6.951872	3.208556	1.604278	9.625668	4.278075	3.208556	2.673797	11.76471	0	0.534759	1.069519	2.139037	10.69519	6.951872	5.882353	11.22995	S
SSA_1060	9.5	4	4.5	6.5	6	5	1	8	2	4	2.5	11.5	1.5	0	5	6.5	6.5	7.5	4	4.5	
SSA_1732	9.090909	4.30622	0.478469	3.349282	6.698565	5.263158	2.870813	8.133971	2.870813	3.349282	5.263158	12.44019	0	0.478469	1.913876	2.392344	8.133971	6.220096	8.133971	8.61244	F
SSA_0578	9.047619	4.285714	1.904762	2.857143	6.190476	3.809524	3.333333	7.142857	5.238095	2.380952	7.142857	12.38095	0.952381	0	5.238095	2.857143	6.190476	9.047619	7.142857	2.857143	H
SSA_1704	9.047619	2.380952	7.142857	5.238095	5.714286	4.761905	0.952381	12.38095	5.238095	0.952381	2.380952	13.33333	1.428571	0.47619	3.333333	2.380952	7.619048	4.761905	4.285714	6.190476	S
SSA_1216	8.837209	5.581395	6.046512	5.581395	7.906977	5.581395	3.255814	7.906977	3.72093	1.860465	2.790698	7.906977	0	0.465116	3.72093	5.116279	6.511628	5.116279	5.116279	6.976744	R
SSA_0967	8.558559	4.504505	3.603604	4.504505	4.504505	7.207207	3.603604	6.756757	2.702703	2.252252	2.252252	12.61261	0.900901	1.351351	3.153153	10.81081	7.657658	4.054054	0.900901	8.108108	S
SSA_0381	8.520179	3.139013	4.484305	6.726457	8.96861	2.242152	0.44843	9.41704	3.139013	2.242152	2.690583	9.41704	0.896861	0.44843	7.623318	7.623318	6.726457	5.829596	7.174888	2.242152	
SSA_0959	8.482143	2.678571	3.571429	4.910714	8.928571	6.25	2.232143	4.017857	2.678571	0.892857	1.785714	15.17857	0.446429	0	3.571429	5.803571	7.142857	6.696429	9.821429	4.910714	TK
SSA_0715	8.40708	2.212389	3.097345	8.40708	9.734513	4.424779	0.442478	6.637168	5.752212	1.327434	1.327434	6.637168	0	0	2.212389	1.769912	9.734513	10.17699	5.752212	11.9469	L
SSA_0819	8.370044	3.0837	2.643172	9.69163	7.048458	7.488987	2.643172	9.69163	3.0837	0	2.202643	11.89427	0.440529	0.440529	3.0837	7.048458	5.286344	3.964758	3.964758	7.929515	M
SSA_1995	8.333333	4.385965	3.947368	6.140351	7.894737	2.631579	1.754386	10.08772	2.631579	3.508772	3.508772	8.77193	0.877193	0.877193	2.631579	3.508772	4.824561	9.649123	7.017544	7.017544	S
SSA_2164	8.296943	5.240175	6.113537	5.676856	4.366812	3.930131	1.746725	7.860262	5.676856	3.49345	3.930131	9.606987	0.873362	1.310044	1.746725	4.366812	7.423581	7.423581	6.113537	4.803493	R
SSA_0319	8.225108	2.164502	3.463203	6.493506	3.030303	5.194805	6.060606	6.493506	4.329004	1.298701	2.597403	15.58442	0.865801	1.298701	2.597403	6.926407	9.52381	2.164502	2.164502	9.52381	E
SSA_2222	8.189655	3.87931	5.603448	7.758621	6.465517	6.896552	2.155172	6.034483	3.87931	1.293103	2.155172	10.77586	0	0.862069	4.741379	2.155172	8.189655	3.87931	6.034483	9.051724	D
SSA_1428	8.050847	2.118644	4.237288	5.932203	6.779661	2.118644	2.542373	5.508475	8.050847	2.966102	3.813559	10.59322	1.271186	0.847458	4.661017	5.084746	5.508475	7.20339	5.084746	7.627119	H
SSA_1725	8.050847	5.084746	4.661017	5.932203	5.508475	4.661017	3.389831	6.779661	5.932203	0.847458	3.389831	11.86441	0	0	1.271186	3.389831	9.745763	8.474576	2.966102	8.050847	E
SSA_1779	8.050847	3.813559	2.118644	4.661017	8.474576	3.813559	3.813559	5.932203	5.508475	1.694915	1.694915	14.40678	0	0.423729	4.661017	3.389831	2.966102	11.44068	7.20339	5.932203	S
SSA_2156	7.983193	4.201681	7.983193	7.983193	1.260504	5.462185	3.361345	6.722689	5.882353	2.521008	2.10084	7.983193	0.840336	0	2.941176	4.201681	10.08403	6.302521	3.361345	8.823529	S
SSA_2214	7.916667	3.75	3.75	5.416667	6.25	7.083333	2.083333	10	4.166667	3.333333	3.75	7.083333	0.416667	1.25	2.916667	4.583333	7.083333	5	6.25	7.916667	I
SSA_2378	7.480315	5.11811	6.299213	4.724409	10.23622	6.299213	1.968504	9.84252	3.149606	2.362205	1.574803	6.299213	0	0.393701	5.905512	5.905512	4.330709	7.480315	7.480315	3.149606	KT
SSA_0635	7.45098	3.921569	3.921569	5.882353	6.27451	5.490196	1.568627	7.843137	4.705882	2.352941	2.352941	12.15686	0	0.392157	3.529412	3.529412	4.313725	10.19608	4.705882	9.411765	E
SSA_1258	7.19697	3.030303	4.545455	5.30303	4.924242	5.30303	4.166667	7.575758	2.651515	2.272727	3.030303	8.712121	0.757576	1.136364	4.166667	4.924242	7.19697	6.060606	5.681818	11.36364	F
SSA_1049	7.089552	3.358209	5.597015	5.970149	2.985075	8.208955	3.358209	9.328358	4.477612	0.746269	3.731343	14.92537	2.985075	0	3.358209	9.328358	6.343284	2.238806	1.865672	4.104478	E
SSA_1227	6.985294	3.676471	2.573529	3.308824	7.352941	5.882353	2.573529	3.676471	4.044118	3.308824	3.676471	11.02941	2.205882	1.102941	4.779412	5.514706	3.308824	6.617647	8.823529	9.558824	
SSA_1554	6.985294	1.838235	2.941176	6.985294	6.617647	8.823529	3.308824	7.352941	4.411765	3.308824	3.308824	9.191176	1.102941	0.367647	6.617647	6.617647	5.882353	5.882353	4.044118	4.411765	S
SSA_0291	6.909091	5.090909	4.363636	6.545455	7.636364	3.636364	4.363636	7.272727	2.181818	1.090909	2.909091	7.272727	1.090909	0.363636	4	3.272727	9.454545	5.818182	4.363636	12.36364	R
SSA_1029	6.785714	2.5	3.571429	7.5	7.857143	4.642857	2.5	7.5	5.714286	1.071429	2.5	11.42857	0.357143	1.428571	2.5	3.571429	6.785714	8.571429	5.357143	7.857143	S
SSA_1454	6.761566	1.423488	5.338078	6.405694	13.16726	6.049822	2.135231	4.982206	2.846975	1.067616	1.779359	8.896797	0.711744	1.423488	4.270463	5.69395	5.338078	7.117438	8.896797	5.69395	
SSA_0802	6.737589	3.546099	4.255319	6.028369	5.673759	6.737589	1.41844	12.76596	3.191489	1.06383	3.191489	9.219858	1.77305	0.35461	3.546099	5.673759	7.092199	5.319149	3.191489	9.219858	S

SSA_1084	6.737589	1.77305	3.546099	7.801418	6.028369	4.609929	2.12766	9.219858	3.900709	2.12766	3.546099	12.05674	0.35461	0	3.191489	3.900709	8.156028	7.446809	5.319149	8.156028	S
SSA_0261	6.620209	1.74216	2.787456	8.362369	3.135889	4.878049	3.484321	14.63415	2.439024	1.393728	2.439024	16.02787	0.348432	0.348432	2.787456	7.317073	8.362369	0.696864	2.439024	9.756098	P
SSA_0612	6.484642	5.119454	5.119454	8.532423	7.167235	3.754266	2.730375	5.802048	4.095563	1.706485	1.365188	12.28669	0.682594	2.389078	5.119454	5.802048	2.730375	7.849829	5.119454	6.143345	S
SSA_1245	6.291391	3.97351	5.960265	7.94702	6.953642	4.304636	2.317881	8.609272	3.97351	3.311258	2.649007	11.5894	0.331126	0	3.97351	5.629139	3.642384	8.278146	5.629139	4.635762	K
SSA_1990	6.229508	1.967213	3.606557	7.213115	10.81967	5.901639	2.295082	5.245902	5.901639	1.967213	3.606557	10.16393	0.655738	0.327869	3.278689	3.278689	4.590164	9.836066	4.590164	8.52459	P
SSA_0373	6.089744	2.564103	5.128205	4.487179	6.410256	6.410256	2.24359	6.730769	3.525641	1.602564	5.128205	8.974359	0	1.602564	4.166667	6.089744	8.653846	8.653846	3.846154	7.692308	F
SSA_1420	6.070288	4.472843	4.472843	7.028754	4.792332	5.750799	2.875399	4.792332	3.194888	4.153355	4.153355	9.904153	1.916933	0.638978	5.111821	6.070288	3.514377	7.028754	7.98722	6.070288	E
SSA_2116	6.050955	3.821656	4.458599	4.458599	7.961783	4.458599	4.458599	6.050955	3.821656	3.503185	4.458599	10.50955	0.636943	0	3.821656	4.140127	5.732484	8.917197	5.732484	7.006369	R
SSA_1483	6.012658	3.481013	5.063291	4.113924	8.227848	6.012658	2.531646	7.911392	2.531646	1.582278	3.797468	9.810127	0	0	3.481013	4.113924	8.544304	6.962025	6.012658	9.810127	IR
SSA_1873	6.012658	5.696203	2.531646	5.379747	8.544304	4.113924	1.265823	6.962025	5.379747	3.481013	4.113924	9.810127	0	0.316456	2.848101	4.113924	5.063291	8.544304	6.962025	8.860759	M
SSA_2217	5.956113	5.956113	3.761755	5.956113	6.269592	5.329154	1.567398	6.269592	8.15047	2.194357	2.194357	13.16614	1.567398	1.253918	4.702194	3.761755	4.075235	4.388715	6.896552	6.583072	M
SSA_0352	5.900621	1.552795	5.279503	9.31677	8.074534	2.795031	0.931677	5.590062	7.142857	3.10559	4.037267	10.86957	0.310559	0	3.726708	6.521739	7.142857	5.279503	2.795031	9.627329	L
SSA_1156	5.864198	2.469136	7.407407	6.790123	10.18519	4.62963	0.617284	7.716049	4.62963	1.54321	3.08642	10.49383	0.308642	0	2.469136	6.17284	3.08642	8.950617	7.716049	5.864198	
SSA_0088	5.637982	4.451039	3.264095	5.934718	3.560831	5.04451	4.154303	3.857567	6.824926	1.48368	2.967359	15.727	1.186944	0.593472	5.04451	3.560831	4.451039	8.605341	5.637982	8.011869	C
SSA_1843	5.60472	6.19469	4.129794	4.424779	5.60472	4.719764	2.064897	8.849558	5.60472	3.539823	2.359882	15.33923	0.294985	0	2.064897	5.014749	4.424779	8.259587	7.079646	4.424779	T
SSA_1114	5.523256	4.069767	2.906977	8.139535	5.523256	5.523256	2.616279	10.17442	5.232558	1.744186	2.034884	13.0814	0.290698	0.290698	2.616279	4.360465	5.813953	9.593023	4.94186	5.523256	T
SSA_2195	5.397727	6.25	2.556818	6.25	6.25	5.397727	1.704545	4.829545	4.545455	1.420455	3.125	9.943182	1.136364	0.568182	7.386364	4.545455	5.113636	7.386364	8.238636	7.954545	H
SSA_1051	5.337079	2.247191	6.179775	4.494382	7.865169	5.337079	4.213483	7.022472	4.494382	1.123596	4.494382	8.707865	1.966292	0	6.741573	3.370787	6.179775	7.865169	5.898876	6.460674	E
SSA_0510	5.21978	6.868132	3.571429	6.593407	3.296703	3.846154	1.648352	4.395604	4.120879	2.747253	4.945055	13.18681	1.098901	1.923077	4.120879	5.494505	3.571429	8.516484	5.769231	9.065934	E
SSA_0267	4.773869	4.522613	3.768844	8.542714	5.025126	5.025126	1.005025	10.55276	5.276382	2.512563	3.768844	11.05528	0.251256	2.01005	5.276382	2.512563	5.527638	7.286432	4.522613	6.78392	KG
SSA_0506	4.761905	3.759398	5.263158	5.012531	8.02005	4.010025	2.255639	9.022556	4.010025	2.506266	4.511278	9.774436	1.002506	2.506266	2.506266	4.010025	7.26817	7.26817	5.764411	6.766917	C
SSA_1686	4.70297	3.960396	3.960396	8.168317	7.178218	4.455446	3.960396	5.445545	5.19802	1.485149	3.217822	12.62376	1.237624	0.49505	3.217822	4.455446	4.207921	6.683168	8.910891	6.435644	Q
SSA_2278	4.534606	3.818616	2.625298	9.546539	8.114558	5.48926	1.193317	5.966587	4.057279	1.193317	2.625298	12.88783	0.954654	0.238663	4.057279	4.295943	3.102625	10.73986	6.921241	7.637232	S
SSA_0236	4.50237	5.687204	4.50237	6.635071	5.924171	4.976303	2.132701	7.582938	3.080569	2.132701	4.739336	11.84834	0.236967	0	3.317536	3.080569	6.398104	5.21327	8.530806	9.478673	L
SSA_2331	4.50237	4.028436	4.976303	6.872038	7.819905	4.265403	3.791469	2.843602	7.345972	1.184834	4.50237	7.819905	1.658768	0.236967	5.450237	7.819905	4.265403	5.924171	5.21327	9.478673	M
SSA_0508	4.3379	2.511416	5.479452	6.621005	9.589041	7.762557	2.054795	6.621005	4.3379	0.456621	2.739726	8.675799	0.913242	0.684932	5.707763	2.511416	6.621005	7.305936	8.219178	6.849315	
SSA_0621	4.03397	6.581741	3.609342	7.430998	6.794055	4.03397	1.910828	8.280255	5.732484	2.123142	4.03397	10.4034	1.061571	0.849257	4.246285	4.883227	6.157113	4.246285	6.157113	7.430998	L
SSA_0592	11.11111	3.888889	5	4.444444	4.444444	5	2.222222	12.22222	2.777778	0.555556	3.888889	7.777778	2.222222	0	1.111111	7.777778	11.66667	2.222222	2.222222	9.444444	S
SSA_1408	10.05025	1.507538	6.532663	13.06533	6.030151	3.517588	3.015075	7.537688	7.537688	0.502513	3.015075	13.06533	1.005025	0	1.507538	3.015075	5.527638	4.020101	3.015075	6.532663	
SSA_0212	9.615385	3.846154	4.807692	3.365385	5.769231	2.884615	3.365385	6.25	6.25	1.442308	5.288462	7.692308	0.961538	0.961538	1.442308	4.326923	8.173077	7.211538	6.25	10.09615	R
SSA_0162	9.009009	5.855856	3.153153	6.306306	7.207207	9.009009	1.801802	4.504505	1.801802	0.900901	2.252252	16.21622	1.801802	0.900901	4.504505	7.657658	5.405405	3.603604	2.252252	5.855856	
SSA_1501	8.888889	5.333333	2.666667	5.333333	6.666667	4.888889	1.333333	8.888889	4	2.666667	3.111111	7.111111	0.444444	0	3.111111	2.222222	6.222222	9.333333	7.555556	10.22222	F
SSA_1201	8.810573	3.0837	5.286344	6.60793	7.929515	9.251101	1.762115	8.370044	3.524229	3.964758	3.524229	11.45374	0.440529	0.440529	1.321586	1.762115	3.964758	6.167401	4.845815	7.488987	H
SSA_2098	8.77193	3.508772	3.508772	7.45614	3.947368	5.263158	1.754386	11.40351	3.947368	0.438596	3.508772	16.22807	0.877193	0.438596	5.263158	7.017544	4.385965	2.192982	1.754386	8.333333	E
SSA_0377	8.695652	5.217391	2.173913	3.043478	0.869565	7.391304	2.173913	11.73913	3.913043	0.869565	3.913043	14.34783	1.304348	0	2.608696	7.826087	10.86957	2.173913	2.608696	8.26087	P
SSA_0071	8.62069	5.172414	0.431034	3.87931	6.465517	5.172414	1.293103	11.2069	4.310345	1.724138	5.172414	7.758621	0	1.293103	3.017241	2.155172	8.189655	7.327586	6.896552	9.913793	G
SSA_1589	8.583691	4.291845	7.296137	4.72103	4.291845	5.150215	2.575107	8.583691	6.008584	2.145923	3.004292	8.154506	0	0.429185	3.004292	2.575107	6.866953	5.579399	7.725322	9.012876	V
SSA_0406	8.333333	2.5	5	6.666667	4.166667	6.666667	3.75	10	2.5	0.416667	1.666667	12.08333	1.666667	1.666667	5.833333	8.75	6.666667	1.25	3.333333	7.083333	
SSA_0472	8.298755	4.149378	2.489627	4.979253	6.224066	5.809129	4.979253	8.713693	1.659751	1.244813	2.904564	10.37344	0.414938	1.659751	3.319502	0.829876	9.543568	7.053942	7.46888	7.883817	H
SSA_1443	8.264463	4.545455	2.066116	4.958678	6.61157	3.305785	2.479339	9.504132	4.958678	1.239669	1.652893	9.504132	0.413223	0	2.479339	2.479339	10.7438	7.85124	6.198347	10.7438	E
SSA_2037	8.264463	2.479339	5.371901	4.545455	8.677686	7.024793	2.479339	7.85124	3.305785	2.892562	3.305785	9.504132	1.239669	0.413223	1.652893	2.479339	7.438017	5.371901	5.785124	9.917355	J
SSA_0278	8.097166	6.072874	2.834008	6.072874	9.716599	6.477733	2.42915	8.502024	3.643725	1.619433	0.809717	9.311741	0	1.214575	2.42915	4.453441	5.263158	7.692308	7.692308	5.668016	KG
SSA_1026	8.097166	3.238866	3.643725	6.477733	6.477733	5.263158	2.024291	6.072874	4.048583	3.238866	2.834008	12.14575	0.809717	0.404858	2.42915	4.453441	7.692308	8.097166	6.072874	6.477733	V
SSA_1080	8.097166	4.048583	4.453441	6.882591	8.097166	6.882591	1.619433	9.716599	4.048583	3.238866	1.619433	9.311741	0	0	0.809717	3.238866	5.263158	10.12146	4.8583	7.692308	KG
SSA_1219	7.968127	3.187251	6.374502	7.171315	8.366534	7.968127	3.585657	8.366534	5.179283	1.195219	2.390438	6.374502	0.796813	0.398406	3.585657	3.585657	6.374502	5.577689	4.38247	7.171315	M
SSA_1355	7.751938	1.550388	4.263566	5.426357	10.46512	6.589147	1.937984	7.364341	3.488372	1.550388	3.488372	10.46512	1.162791	0	5.03876	5.426357	4.263566	7.751938	8.139535	3.875969	
SSA_0659	7.662835	5.747126	3.831418	8.812261	8.045977	3.065134	2.298851	4.980843	6.51341	2.681992	2.298851	13.02682	0	0	3.831418	5.747126	3.831418	5.747126	5.747126	6.130268	S
SSA_1868	7.462687	3.358209	3.731343	5.223881	4.477612	5.223881	2.61194	10.8209	3.731343	0	4.104478	11.56716	1.119403	0	3.731343	7.089552	6.716418	5.597015	1.865672	11.56716	E
SSA_1749	7.434944	4.089219	4.089219	2.973978	8.921933	7.063197	2.973978	5.947955	2.973978	2.973978	3.717472	10.03717	1.486989	2.230483	3.345725	4.089219	7.063197	7.806691	6.319703	4.460967	O
SSA_2218	7.352941	6.617647	4.411765	4.779412	6.25	3.676471	4.044118	5.514706	6.25	2.205882	4.779412	10.29412	1.470588	1.102941	4.411765	4.779412	4.779412	6.617647	5.514706	5.147059	M
SSA_0727	7.29927	3.649635	2.189781	5.474453	4.379562	5.109489	2.919708	10.94891	2.554745	1.094891	4.014599	12.40876	3.649635	0.729927	4.014599	5.839416	9.124088	2.919708	1.094891	10.58394	R
SSA_0827	7.168459	3.942652	4.301075	5.017921	5.376344	3.942652	1.433692	4.301075	4.301075	1.792115	3.584229	8.960573	2.150538	0.358423	6.451613	5.734767	5.734767	11.46953	9.318996	4.659498	
SSA_1004	6.944444	1.388889	4.861111	4.861111	5.555556	5.555556	3.472222	10.06944	3.125	0	3.472222	12.84722	2.430556	0.347222	3.472222	10.41667	8.680556	2.430556	1.041667	9.027778	G
SSA_1526	6.896552	2.413793	4.482759	7.241379	3.448276	3.448276	2.758621	11.37931	0.689655	1.724138	3.448276	18.27586	1.724138	0	3.793103	6.206897	10.68966	3.448276	1.724138	6.206897	S
SSA_2190	6.896552	3.793103	4.137931	5.517241	5.862069	7.241379	1.724138	6.896552	5.862069	1.034483	3.103448	8.965517	0	1.37931	2.758621	3.448276	7.586207	9.310345	5.862069	8.62069	O
SSA_2362	6.896552	3.448276	3.448276	7.586207	4.137931	4.482759	3.448276	5.517241	2.758621	1.034483	2.758621	10.68966	0	2.068966	2.068966	2.758621	9.655172	7.931034	3.793103	15.51724	E
SSA_2321	6.872852	1.030928	4.467354	7.216495	5.498282	4.810997	2.061856	9.621993	2.749141	3.092784	2.061856	14.77663	1.718213	0.687285	2.405498	5.841924	5.841924	6.52921	5.154639	7.560137	P
SSA_0810	6.756757	6.081081	5.405405	5.743243	6.756757	5.067568	2.364865	5.067568	4.391892	1.689189	4.391892	11.82432	0.337838	0.337838	2.702703	5.405405	5.405405	6.756757	7.77027	5.743243	R
SSA_0611	6.711409	6.375839	3.355705	7.38255	2.348993	3.355705	4.697987	9.060403	3.020134	2.348993	4.026846	12.08054	1.006711	0	2.348993	2.684564	7.04698	6.040268	4.026846	12.08054	O
SSA_0485	6.535948	5.555556	1.30719	3.921569	6.862745	3.921569	2.614379	4.901961	8.823529	1.30719	3.267974	13.0719	0.653595	1.960784	0.980392	2.287582	7.51634	7.189542	5.555556	11.76471	H
SSA_1509	6.430868	6.752412	5.144695	5.144695	5.466238	5.787781	1.92926	5.466238	6.752412	2.250804	2.250804	12.21865	2.250804	0.321543	4.180064	4.180064	3.858521	6.752412	6.752412	6.109325	M
SSA_1340	6.410256	3.205128	3.525641	9.615385	6.730769	4.487179	1.282051	4.487179	6.730769	1.923077	3.846154	10.57692	0.961538	0.961538	2.884615	3.525641	4.166667	8.974359	6.089744	9.615385	P
SSA_1920	6.389776	2.236422	3.194888	5.43131	6.709265	5.43131	3.194888	8.306709	4.153355	1.277955	3.514377	12.14058	3.194888	0.319489	2.555911	4.792332	8.945687	4.472843	3.833866	9.904153	G

SSA_2346	6.369427	3.503185	3.503185	8.280255	6.050955	6.369427	4.458599	7.324841	2.866242	2.547771	3.503185	10.50955	0.318471	1.273885	2.866242	3.184713	6.369427	7.006369	4.77707	8.917197	I
SSA_1767	6.309148	4.416404	8.51735	5.678233	9.463722	4.731861	1.577287	8.51735	1.577287	2.523659	2.839117	12.93375	0.315457	0.315457	3.785489	1.577287	6.940063	10.09464	3.154574	4.731861	V
SSA_1042	6.23053	3.115265	6.853583	7.788162	8.722741	9.034268	2.803738	3.115265	4.361371	2.180685	3.738318	11.83801	0.623053	0	3.115265	2.492212	4.361371	4.984424	6.853583	7.788162	G
SSA_1542	6.19195	3.405573	5.263158	5.572755	5.882353	4.024768	2.786378	7.430341	2.786378	4.643963	3.095975	9.287926	0.619195	0.619195	4.643963	6.19195	4.95356	8.359133	7.430341	6.811146	O
SSA_0891	5.952381	3.27381	5.059524	4.464286	4.166667	6.845238	0.297619	5.654762	4.166667	5.357143	3.27381	8.928571	0	1.488095	5.952381	3.27381	8.333333	5.357143	7.738095	10.41667	G
SSA_1656	5.952381	1.785714	5.357143	6.25	9.22619	5.357143	1.488095	3.571429	5.357143	0.297619	4.761905	12.79762	0.595238	0.892857	5.059524	3.571429	7.440476	7.440476	5.952381	6.845238	M
SSA_0544	5.830904	6.122449	6.413994	4.373178	5.539359	5.247813	2.915452	6.705539	4.373178	1.457726	4.081633	9.620991	0.58309	0.58309	4.373178	2.623907	6.997085	7.87172	6.413994	7.87172	E
SSA_2086	5.830904	3.790087	4.373178	5.539359	4.664723	5.830904	1.457726	5.247813	3.498542	2.915452	3.206997	9.329446	2.040816	1.457726	4.081633	6.413994	7.580175	7.580175	6.997085	8.163265	
SSA_1321	5.479452	4.657534	2.739726	5.753425	6.30137	2.739726	3.013699	9.041096	4.109589	2.465753	5.479452	8.219178	1.09589	1.369863	4.383562	6.027397	3.835616	13.42466	5.753425	4.109589	H
SSA_2292	5.464481	3.825137	6.010929	7.103825	4.918033	4.918033	1.36612	9.289617	6.010929	3.825137	5.191257	12.56831	0.819672	0.819672	3.551913	5.464481	4.644809	3.551913	5.737705	4.918033	D
SSA_0760	5.263158	3.421053	2.894737	6.842105	5	4.210526	2.368421	7.105263	6.052632	1.578947	3.157895	10.52632	0.263158	0.526316	3.157895	5.263158	10	7.894737	3.421053	11.05263	E
SSA_1206	5.181347	4.145078	4.663212	4.92228	5.440415	2.331606	3.108808	5.440415	6.735751	2.331606	6.476684	8.80829	2.849741	0	3.88601	5.440415	5.440415	8.549223	6.476684	7.772021	L
SSA_2347	4.651163	7.674419	3.023256	5.813953	5.348837	3.72093	2.55814	6.976744	6.27907	2.093023	3.255814	9.767442	0.465116	1.395349	3.488372	6.744186	5.348837	7.674419	6.046512	7.674419	H
SSA_2178	4.597701	4.137931	5.977011	15.63218	7.816092	4.137931	1.149425	5.057471	3.908046	1.609195	2.298851	9.195402	0.689655	0	3.448276	3.908046	5.747126	9.655172	5.977011	5.057471	
SSA_0838	4.474273	4.9217	3.579418	6.263982	4.026846	4.474273	1.565996	5.592841	8.053691	3.355705	3.355705	13.64653	0.671141	0.447427	4.9217	4.697987	5.592841	6.263982	7.158837	6.935123	
SSA_2141	4.347826	4.347826	3.478261	7.608696	5	4.347826	3.478261	5.434783	5.434783	3.26087	2.391304	13.26087	0.652174	0.652174	3.695652	4.130435	6.521739	8.043478	5.652174	8.26087	E
SSA_2151	4.056795	2.636917	7.302231	9.330629	7.302231	3.853955	2.028398	9.533469	3.651116	4.056795	2.231237	12.17039	0.811359	1.014199	4.868154	8.11359	1.622718	7.505071	4.868154	3.042596	K
SSA_0834	3.929273	6.679764	3.143418	5.89391	5.89391	3.536346	3.732809	5.89391	3.339882	1.964637	3.929273	10.21611	1.178782	0.785855	5.500982	6.286837	8.05501	7.858546	6.679764	5.500982	
SSA_0118	20.79208	2.970297	7.920792	1.980198	16.83168	0.990099	0.990099	3.960396	2.970297	1.980198	3.960396	3.960396	0	0	1.980198	1.980198	9.90099	5.940594	4.950495	5.940594	J
SSA_2031	10.39604	2.970297	3.960396	2.970297	7.425743	6.435644	0.990099	4.950495	5.445545	0.49505	0.49505	8.910891	0.49505	0	3.465347	3.960396	7.425743	9.405941	6.435644	13.36634	
SSA_2048	10.2439	5.365854	0.487805	5.853659	6.341463	4.878049	1.463415	6.829268	1.95122	6.829268	3.902439	7.317073	2.439024	0	1.95122	2.439024	10.2439	8.292683	3.902439	9.268293	E
SSA_0300	9.813084	0	8.878505	8.411215	9.813084	9.813084	0.46729	6.074766	3.271028	0	2.336449	6.074766	1.401869	0	4.205607	3.738318	8.411215	7.476636	4.672897	5.140187	
SSA_1036	9.545455	2.272727	2.727273	5	9.090909	6.363636	2.272727	5.909091	2.272727	0.454545	2.272727	7.272727	0.454545	1.818182	2.272727	3.181818	9.090909	8.636364	6.363636	12.72727	F
SSA_1685	9.210526	4.385965	5.263158	4.385965	6.578947	3.947368	4.385965	7.45614	3.947368	3.070175	2.192982	11.84211	1.315789	0.438596	1.754386	6.140351	6.578947	6.140351	8.333333	2.631579	TK
SSA_1241	9.130435	3.478261	2.173913	5.217391	6.521739	4.782609	3.478261	7.391304	4.347826	3.043478	4.347826	6.521739	0.869565	1.304348	2.608696	3.913043	6.956522	8.26087	7.391304	8.26087	F
SSA_0465	9.051724	3.87931	3.448276	7.327586	6.465517	4.741379	2.155172	10.34483	3.448276	1.293103	3.017241	8.189655	0	0.431034	3.87931	3.017241	6.896552	9.913793	4.741379	7.758621	H
SSA_1360	8.536585	3.252033	4.878049	3.658537	6.910569	5.691057	4.471545	4.878049	3.658537	2.439024	4.065041	10.97561	0	0.406504	1.219512	5.284553	7.723577	6.504065	9.349593	6.097561	E
SSA_2302	8.536585	1.626016	1.626016	8.130081	5.284553	2.845528	3.252033	9.349593	1.626016	0.813008	3.658537	14.63415	2.439024	2.439024	4.878049	8.536585	8.130081	3.252033	2.439024	6.504065	NOU
SSA_2126	8.203125	5.078125	1.953125	3.90625	5.859375	6.25	2.734375	5.46875	3.515625	3.125	4.296875	8.984375	0.78125	0	4.296875	3.515625	6.25	10.54688	5.859375	9.375	L
SSA_0660	7.954545	6.060606	4.166667	6.060606	6.060606	5.681818	1.136364	4.924242	4.924242	2.272727	1.515152	7.954545	0.378788	0	1.893939	3.030303	1.893939	15.90909	7.954545	10.22727	D
SSA_1567	7.777778	1.851852	5.185185	8.888889	10.37037	7.407407	0.740741	7.777778	4.814815	0.740741	2.962963	11.11111	0.37037	0	5.185185	2.222222	5.925926	3.703704	7.777778	5.185185	ET
SSA_1319	7.749077	2.583026	4.059041	7.380074	4.797048	3.321033	3.321033	5.535055	5.535055	1.845018	2.95203	9.225092	0.738007	1.845018	3.690037	4.059041	6.642066	8.856089	6.273063	9.594096	R
SSA_1988	7.720588	2.573529	3.308824	9.558824	4.411765	3.308824	3.676471	10.66176	3.676471	0.367647	2.573529	15.07353	2.205882	0.735294	3.676471	10.66176	6.617647	2.205882	2.205882	4.779412	R
SSA_1607	7.664234	8.759124	1.459854	2.189781	6.934307	6.569343	1.824818	4.744526	3.649635	2.554745	6.20438	11.31387	0.364964	1.459854	4.014599	3.649635	9.489051	8.029197	3.284672	5.839416	L
SSA_2329	7.636364	2.545455	4.363636	4.363636	6.909091	4	3.636364	8	5.090909	2.545455	5.818182	12	0.727273	0.363636	3.272727	2.181818	4.727273	6.909091	6.545455	8.363636	R
SSA_1678	7.47331	3.914591	4.626335	6.405694	3.558719	6.405694	4.626335	8.540925	4.270463	0.355872	2.491103	16.72598	1.779359	0.355872	3.202847	6.405694	5.69395	2.846975	3.202847	7.117438	S
SSA_1469	7.394366	3.169014	5.28169	5.985915	5.985915	4.577465	3.873239	7.394366	5.28169	1.408451	4.225352	9.859155	1.408451	0.352113	3.521127	3.521127	7.042254	6.338028	3.873239	9.507042	E
SSA_0511	7.291667	5.208333	2.430556	9.722222	4.513889	4.513889	1.736111	2.777778	6.597222	0.694444	5.208333	14.58333	1.041667	2.083333	3.472222	2.083333	5.555556	9.375	3.819444	7.291667	Q
SSA_0375	7.241379	1.37931	6.896552	7.241379	11.03448	5.172414	1.37931	5.517241	2.758621	0.689655	2.758621	8.275862	1.724138	0.344828	3.448276	2.068966	5.862069	7.586207	7.931034	10.68966	P
SSA_1631	7.118644	5.762712	4.067797	6.779661	8.135593	6.440678	1.355932	8.474576	3.050847	3.389831	4.067797	12.20339	0.677966	1.355932	4.40678	1.694915	6.779661	4.745763	4.745763	4.745763	M
SSA_2210	7.023411	6.354515	3.67893	3.010033	8.695652	5.685619	1.672241	5.685619	3.67893	5.685619	5.351171	12.04013	0.668896	2.006689	3.010033	4.347826	5.351171	6.688963	4.347826	5.016722	J
SSA_0412	6.953642	4.966887	3.642384	6.622517	6.622517	5.298013	1.655629	9.602649	4.966887	3.311258	1.986755	12.25166	0.331126	0.331126	3.642384	3.642384	5.960265	8.940397	4.635762	4.635762	V
SSA_0487	6.501548	6.19195	2.167183	5.882353	4.643963	4.024768	2.786378	6.19195	4.643963	1.23839	3.71517	8.978328	0.309598	1.23839	2.786378	5.263158	7.120743	8.978328	5.882353	11.45511	H
SSA_1079	6.422018	2.752294	3.058104	6.422018	5.504587	4.892966	4.587156	8.256881	3.058104	0.917431	3.363914	12.84404	1.529052	0.917431	4.892966	10.39755	7.033639	3.975535	1.834862	7.33945	
SSA_1000	6.306306	3.603604	1.501502	8.708709	7.507508	6.906907	2.702703	7.507508	6.606607	1.501502	3.003003	8.708709	0.3003	0.900901	4.504505	3.903904	4.804805	6.906907	7.507508	6.606607	K
SSA_0185	6.287425	3.592814	2.994012	8.383234	5.988024	2.39521	3.293413	6.287425	5.389222	1.497006	2.994012	17.06587	0.898204	0	4.491018	6.586826	5.988024	7.185629	1.497006	7.185629	NU
SSA_0965	6.25	4.166667	3.571429	6.547619	5.952381	4.166667	2.083333	5.952381	5.059524	2.97619	2.678571	8.928571	0.595238	0.297619	4.464286	5.952381	5.654762	6.547619	5.654762	12.5	R
SSA_0154	6.104651	3.488372	8.139535	6.104651	9.883721	5.813953	3.197674	6.976744	5.232558	0.872093	1.162791	9.302326	0.581395	0	3.197674	3.77907	2.616279	10.17442	6.395349	6.976744	K
SSA_0314	6	5.714286	4	6.285714	6.857143	5.428571	2.571429	5.428571	3.714286	2.857143	4.285714	10.28571	0.571429	0.857143	4.571429	4.285714	6	7.142857	5.142857	8	MG
SSA_1171	5.898876	5.898876	5.05618	6.460674	9.550562	7.022472	2.247191	5.05618	2.808989	1.685393	2.52809	12.35955	0.561798	0	5.898876	3.370787	2.52809	8.707865	4.775281	7.58427	L
SSA_1973	5.737705	4.644809	4.098361	6.557377	5.464481	4.644809	2.459016	8.469945	4.918033	2.185792	2.185792	13.38798	2.185792	0.546448	3.551913	6.284153	4.644809	6.830601	4.371585	6.830601	T
SSA_0512	5.706522	3.804348	2.173913	4.347826	6.793478	6.521739	3.26087	6.25	3.26087	2.717391	3.26087	12.22826	0	2.98913	3.532609	2.173913	9.51087	5.978261	6.25	9.23913	H
SSA_1466	5.706522	5.706522	3.26087	5.706522	5.163043	4.891304	2.173913	8.967391	3.532609	2.98913	2.98913	9.51087	0.543478	0	2.173913	4.347826	5.434783	9.51087	6.25	11.1413	E
SSA_0703	5.645161	5.107527	2.956989	6.989247	4.83871	4.83871	2.688172	6.989247	4.301075	4.569892	2.419355	10.48387	0.806452	1.88172	4.83871	2.956989	5.107527	8.333333	4.83871	9.408602	C
SSA_1075	5.585106	4.255319	3.989362	5.053191	7.978723	4.255319	2.925532	6.648936	3.457447	2.393617	3.457447	10.90426	0.265957	0.797872	5.319149	4.521277	6.648936	9.574468	5.585106	6.382979	H
SSA_2173	5.570292	3.448276	2.917772	2.65252	4.244032	7.161804	3.183024	5.835544	3.448276	3.97878	4.244032	11.67109	1.061008	0.530504	2.65252	5.30504	9.549072	7.95756	6.896552	7.692308	R
SSA_0323	5.370844	2.557545	3.836317	7.161125	8.439898	7.928389	1.790281	6.138107	2.813299	3.580563	3.580563	11.50895	0.511509	1.023018	1.534527	5.626598	10.23018	5.626598	4.603581	6.138107	R
SSA_1662	5.316456	4.050633	1.265823	7.088608	5.316456	4.556962	1.265823	6.835443	4.810127	3.797468	4.303797	10.12658	0.253165	0.253165	4.556962	3.797468	10.12658	7.848101	6.075949	8.35443	C
SSA_0654	5.236908	1.496259	4.239401	8.977556	9.725686	5.985037	1.745636	6.483791	5.236908	1.995012	4.239401	7.98005	0.498753	0	3.491272	2.244389	4.239401	16.45885	3.990025	5.735661	M
SSA_1130	5.223881	4.726368	3.731343	5.223881	9.452736	6.218905	2.487562	3.731343	3.9801	1.243781	3.233831	8.208955	1.492537	0.746269	2.985075	6.965174	8.955224	6.218905	8.706468	6.467662	P
SSA_0857	5.159705	2.702703	4.422604	7.616708	5.159705	5.896806	2.211302	8.108108	1.965602	0.737101	3.194103	18.42752	1.965602	1.228501	4.914005	9.336609	4.176904	3.685504	1.228501	7.862408	
SSA_2117	5.023923	6.45933	4.30622	7.894737	6.45933	6.937799	1.435407	5.263158	6.45933	1.435407	1.913876	14.35407	0.239234	0	2.631579	3.349282	4.784689	10.76555	5.502392	4.784689	S
SSA_2290	4.393305	4.60251	6.694561	7.949791	6.276151	6.066946	2.719665	10.04184	4.60251	0.627615	2.51046	9.832636	1.046025	0.627615	4.393305	5.648536	4.1841	8.577406	6.066946	3.138075	

SSA_0234	12.8655	2.339181	2.923977	4.678363	2.339181	2.339181	1.169591	11.11111	0.584795	0.584795	4.093567	18.12865	2.339181	0	4.093567	11.69591	5.847953	1.169591	1.169591	10.52632	S
SSA_2223	9.52381	5.194805	3.463203	7.359307	6.060606	6.493506	2.597403	10.38961	4.329004	0.4329	3.463203	10.82251	0.4329	0	1.731602	3.463203	3.463203	7.792208	4.761905	8.225108	M
SSA_1828	9.401709	0.854701	4.273504	4.273504	3.418803	5.555556	2.136752	7.264957	1.709402	1.709402	5.982906	15.81197	2.136752	0	2.136752	5.982906	13.67521	2.136752	1.709402	9.82906	G
SSA_1132	8.8	6	2	7.2	3.6	6	1.2	6.8	2.4	0.8	4	18.8	1.2	1.2	4.4	9.6	2.4	3.6	2.4	7.6	U
SSA_2353	8.8	2.8	0.8	5.2	4.8	4.8	4.8	11.2	4	1.2	4	17.2	3.6	0.8	3.2	4.8	6.8	2	2.8	6.4	P
SSA_2097	8.661417	4.724409	2.755906	7.480315	7.086614	3.543307	2.362205	6.692913	5.11811	2.755906	3.543307	11.41732	0	0.393701	2.755906	3.937008	6.299213	8.661417	4.724409	7.086614	E
SSA_1085	8.627451	3.529412	1.960784	4.705882	6.27451	5.098039	2.745098	5.098039	4.313725	2.745098	3.529412	9.803922	0.392157	0.392157	1.960784	5.098039	9.019608	9.411765	4.705882	10.58824	E
SSA_2001	8.59375	2.34375	3.515625	7.8125	6.25	3.90625	1.5625	6.640625	5.078125	2.34375	3.125	11.71875	0	1.171875	2.734375	4.296875	6.640625	7.421875	5.078125	9.765625	H
SSA_0470	8.560311	3.891051	1.167315	8.171206	7.782101	4.280156	2.723735	7.392996	3.11284	2.723735	3.891051	7.003891	0.389105	0.77821	4.280156	3.891051	8.171206	8.560311	5.447471	7.782101	H
SSA_0494	8.461538	3.846154	2.692308	7.307692	7.692308	2.692308	3.461538	4.615385	5.769231	3.076923	4.615385	15.76923	0.384615	0.769231	1.538462	2.307692	6.538462	9.230769	2.692308	6.538462	EP
SSA_0222	7.913669	1.798561	2.517986	3.23741	7.194245	5.755396	3.597122	6.834532	2.877698	1.438849	3.23741	12.94964	1.798561	0.719424	3.956835	4.316547	11.8705	5.395683	2.877698	9.71223	G
SSA_2219	7.692308	6.293706	4.195804	9.440559	7.692308	3.846154	2.447552	5.244755	6.293706	1.748252	3.146853	9.440559	0.699301	0.34965	4.895105	4.545455	4.895105	8.041958	4.895105	4.195804	MG
SSA_1067	7.638889	3.472222	3.472222	5.902778	5.555556	2.430556	1.736111	3.472222	6.944444	0.347222	3.819444	12.5	0.347222	0.347222	5.555556	5.902778	6.25	9.722222	6.25	8.333333	R
SSA_2153	7.638889	2.777778	3.125	5.902778	3.125	6.25	3.819444	9.722222	2.430556	0.347222	2.430556	16.66667	0.694444	1.041667	2.083333	5.208333	11.80556	2.083333	2.430556	10.41667	R
SSA_1728	7.612457	3.114187	4.152249	5.190311	3.460208	4.152249	4.152249	13.14879	2.422145	0.346021	2.422145	14.53287	0	0	3.806228	6.574394	10.72664	1.730104	1.730104	10.72664	E
SSA_0935	7.534247	7.191781	1.369863	6.164384	6.506849	6.164384	3.082192	5.821918	2.739726	1.027397	3.082192	9.931507	0	0.684932	3.082192	4.109589	7.534247	10.9589	5.821918	7.191781	J
SSA_0079	7.482993	2.040816	4.421769	3.061224	5.442177	3.741497	2.380952	8.163265	3.741497	3.401361	3.401361	7.482993	0.340136	2.721088	3.741497	3.401361	12.92517	7.482993	3.741497	10.88435	KG
SSA_0767	7.457627	1.694915	5.762712	6.779661	6.440678	5.084746	1.355932	6.779661	1.694915	1.694915	3.389831	10.84746	0.338983	0.338983	3.389831	4.745763	8.474576	8.813559	8.135593	6.779661	IR
SSA_1208	7.457627	5.084746	3.050847	5.423729	8.135593	3.728814	1.016949	7.457627	3.728814	6.101695	3.389831	9.491525	0	0.338983	4.40678	4.745763	6.779661	7.79661	6.101695	5.762712	J
SSA_1723	7.457627	2.372881	3.389831	5.084746	5.762712	7.118644	2.711864	11.52542	2.711864	1.355932	2.372881	12.88136	0.677966	0.677966	3.728814	7.457627	9.491525	3.050847	3.728814	6.440678	S
SSA_2300	7.236842	4.276316	4.276316	8.223684	2.960526	2.631579	5.263158	8.552632	1.973684	0.986842	2.631579	16.11842	1.644737	0.328947	3.947368	11.18421	6.25	1.973684	3.618421	5.921053	P
SSA_0856	7.142857	5.194805	4.220779	6.818182	4.545455	4.545455	3.246753	8.766234	2.597403	0.974026	3.896104	10.06494	1.623377	0.974026	3.571429	7.467532	6.493506	8.116883	4.220779	5.519481	M
SSA_1358	7.096774	1.935484	2.903226	6.451613	3.870968	3.225806	2.258065	6.774194	2.258065	0.645161	3.870968	21.29032	2.580645	0	2.258065	8.387097	10.64516	3.225806	1.935484	8.387097	
SSA_0374	7.073955	4.823151	3.215434	3.858521	4.501608	5.466238	1.286174	4.501608	5.787781	3.215434	3.858521	7.717042	0.643087	1.286174	7.717042	4.180064	8.360129	10.61093	5.144695	6.752412	O
SSA_0396	6.984127	3.492063	3.492063	4.126984	8.888889	5.079365	1.904762	7.619048	5.714286	2.539683	4.126984	10.79365	0.634921	0	3.492063	4.444444	6.031746	7.619048	4.444444	8.571429	R
SSA_1357	6.984127	2.539683	3.492063	7.936508	4.126984	3.174603	2.222222	11.11111	3.809524	0.952381	2.857143	13.33333	2.539683	0.634921	3.174603	9.206349	10.47619	1.904762	1.904762	7.619048	
SSA_1770	6.984127	7.301587	4.444444	3.809524	5.079365	5.714286	3.492063	6.666667	3.809524	3.809524	4.126984	8.571429	0.952381	2.539683	4.126984	2.857143	6.349206	8.888889	5.714286	4.761905	R
SSA_0454	6.853583	3.115265	5.29595	7.788162	7.476636	4.672897	1.869159	9.657321	4.049844	1.246106	4.984424	10.28037	0	0.311526	3.738318	2.803738	6.23053	4.984424	6.542056	8.099688	K
SSA_1178	6.832298	3.416149	5.590062	4.658385	7.453416	3.416149	4.347826	6.832298	2.173913	2.795031	2.795031	7.453416	0.931677	0.931677	2.795031	3.416149	10.55901	9.937888	4.347826	9.31677	C
SSA_2267	6.727829	5.198777	4.281346	6.727829	7.033639	4.281346	1.223242	8.868502	5.198777	1.223242	3.975535	10.09174	0	0.30581	4.587156	4.892966	4.281346	6.116208	8.562691	6.422018	K
SSA_0062	6.321839	3.16092	5.747126	6.609195	4.022989	6.896552	0.862069	5.45977	6.321839	2.586207	4.310345	8.045977	0.574713	0.862069	4.022989	5.172414	9.195402	4.597701	7.471264	7.758621	G
SSA_1409	6.321839	3.16092	6.034483	5.45977	7.471264	6.609195	0.574713	7.183908	1.724138	3.735632	3.735632	6.896552	2.011494	0.287356	5.172414	3.16092	7.758621	7.758621	7.183908	7.758621	M
SSA_1449	6.25	4.261364	4.545455	5.965909	3.125	3.693182	1.420455	6.25	5.113636	1.136364	5.113636	11.93182	0.852273	0.568182	4.545455	5.113636	6.25	7.386364	6.818182	9.659091	E
SSA_2303	6.214689	4.237288	4.80226	8.474576	6.779661	6.214689	1.977401	5.649718	5.084746	1.129944	4.519774	10.16949	0.564972	0	3.954802	5.649718	4.80226	7.627119	4.80226	7.344633	V
SSA_1945	6.197183	3.943662	2.253521	6.197183	6.478873	6.760563	2.535211	9.859155	3.380282	1.971831	4.788732	7.887324	1.126761	0.56338	3.380282	3.380282	6.197183	7.887324	6.760563	8.450704	EP
SSA_1715	6.060606	3.581267	4.683196	7.438017	6.61157	3.581267	3.030303	6.336088	3.581267	0.826446	5.23416	9.090909	0.550964	0	4.958678	5.23416	6.336088	7.713499	5.509642	9.641873	HE
SSA_0718	6.043956	1.373626	3.571429	6.318681	1.098901	2.197802	1.098901	5.769231	21.42857	0.824176	7.967033	5.21978	1.648352	0.824176	3.296703	8.241758	9.065934	3.571429	2.747253	7.692308	S
SSA_0044	5.820106	2.910053	7.142857	7.142857	11.64021	6.084656	2.116402	8.994709	4.761905	1.322751	1.851852	11.64021	0.26455	0.793651	4.497354	4.761905	2.645503	8.465608	4.232804	2.910053	
SSA_0235	5.804749	6.596306	3.957784	5.804749	9.762533	5.277045	2.110818	8.443272	3.957784	2.902375	2.110818	11.08179	1.319261	0.527704	5.013193	3.957784	5.277045	7.915567	4.74934	3.430079	L
SSA_1510	5.759162	3.403141	4.188482	3.664921	8.900524	4.973822	1.570681	6.806283	4.973822	2.879581	3.141361	7.591623	1.570681	1.04712	5.759162	6.282723	6.544503	8.115183	5.235602	7.591623	M
SSA_0964	5.699482	5.181347	3.108808	5.440415	7.772021	5.699482	2.590674	6.735751	5.440415	2.072539	3.626943	13.98964	0.518135	0.777202	3.108808	4.404145	4.663212	7.512953	5.440415	6.217617	LKJ
SSA_1471	5.684755	4.392765	4.909561	6.718346	7.751938	4.392765	1.29199	4.134367	5.426357	2.067183	1.808786	10.59432	0.775194	0.258398	4.134367	8.527132	6.976744	5.167959	5.943152	9.043928	R
SSA_0023	5.641026	2.820513	3.333333	4.615385	6.153846	5.641026	2.307692	7.948718	4.358974	1.282051	4.358974	9.230769	0.25641	0.25641	5.384615	4.615385	6.153846	8.205128	5.384615	12.05128	E
SSA_1326	5.405405	3.194103	4.668305	4.914005	4.914005	7.371007	3.439803	7.371007	4.176904	2.457002	4.914005	6.879607	0	0	3.931204	5.159705	8.599509	8.353808	6.879607	7.371007	E
SSA_0484	5.275779	5.515588	2.398082	5.755396	6.954436	4.556355	1.678657	8.153477	5.755396	3.117506	2.398082	13.66906	0.719424	0.719424	2.877698	3.357314	5.275779	9.592326	5.035971	7.194245	H
SSA_1322	5.152225	5.854801	2.34192	4.449649	6.557377	5.620609	1.639344	6.791569	4.683841	1.639344	5.620609	12.64637	1.405152	1.873536	3.981265	4.449649	6.088993	6.323185	6.323185	6.557377	GC
SSA_0388	3.900709	4.432624	3.723404	9.574468	4.964539	3.191489	1.77305	7.624113	4.964539	1.950355	3.014184	13.12057	0.886525	0.531915	4.787234	5.141844	5.496454	6.028369	5.673759	9.219858	L
SSA_0527	11.05769	5.288462	3.365385	4.326923	7.692308	4.326923	1.442308	5.769231	4.807692	4.326923	4.326923	8.653846	0	1.442308	1.442308	3.365385	8.173077	7.692308	5.769231	6.730769	Q
SSA_0946	10.59908	5.990783	5.069124	4.147465	5.990783	4.147465	2.764977	7.834101	5.069124	1.382488	0.921659	7.834101	0.460829	0.460829	4.147465	3.225806	3.686636	11.52074	7.373272	7.373272	P
SSA_0535	10.36036	4.054054	3.153153	6.306306	8.108108	7.657658	2.252252	6.306306	4.504505	1.801802	6.756757	7.657658	0	0.45045	2.252252	1.351351	5.855856	9.009009	3.153153	9.009009	
SSA_1810	10.04367	6.9869	3.056769	6.550218	5.240175	3.930131	2.183406	7.423581	5.240175	1.310044	1.746725	12.22707	0.436681	0.436681	4.803493	3.056769	3.930131	8.296943	6.550218	6.550218	TK
SSA_0466	9.82906	2.136752	2.991453	6.837607	8.974359	4.700855	1.709402	5.982906	3.418803	1.709402	3.846154	8.119658	0	0.42735	3.846154	5.555556	6.837607	7.692308	5.555556	9.82906	H
SSA_2088	9.745763	5.508475	3.389831	4.661017	5.084746	5.508475	2.118644	4.237288	1.694915	4.237288	8.050847	6.779661	1.271186	0	5.508475	2.966102	8.050847	8.474576	4.661017	8.050847	G
SSA_1753	9.663866	5.882353	2.521008	5.462185	10.5042	3.781513	1.680672	5.882353	8.403361	1.680672	1.680672	9.663866	0.420168	0	4.621849	4.201681	3.361345	6.722689	9.663866	4.201681	K
SSA_0901	9.623431	4.1841	2.51046	4.1841	5.857741	4.1841	4.60251	5.020921	3.76569	2.92887	3.76569	7.949791	0.41841	0	4.1841	5.439331	8.786611	9.623431	7.112971	5.857741	Q
SSA_0473	9.236948	4.819277	1.204819	7.630522	5.62249	5.62249	2.409639	8.032129	3.614458	1.204819	3.212851	10.84337	0	1.606426	2.811245	2.409639	7.228916	8.032129	6.425703	8.032129	H
SSA_0762	9.055118	3.937008	5.11811	5.905512	5.905512	4.724409	1.181102	10.62992	3.937008	1.181102	2.362205	13.77953	1.181102	0.393701	7.480315	7.086614	3.937008	5.511811	1.574803	5.11811	
SSA_0985	9.055118	4.330709	5.905512	2.362205	3.937008	4.330709	1.181102	5.905512	7.874016	3.937008	1.574803	5.11811	1.574803	0	4.724409	6.299213	8.267717	8.661417	7.874016	7.086614	S
SSA_2193	8.846154	4.615385	2.307692	4.230769	8.461538	5.384615	1.923077	5	4.615385	1.538462	5.384615	9.230769	1.153846	0.769231	5.384615	4.615385	5	8.846154	6.153846	6.538462	F
SSA_1942	8.745247	4.18251	3.802281	5.703422	7.604563	5.323194	3.422053	4.562738	1.901141	0.760456	2.661597	10.26616	0.760456	1.140684	2.281369	5.323194	6.844106	9.125475	4.562738	11.02662	I
SSA_1508	8.58209	3.731343	5.223881	4.477612	5.597015	2.985075	5.223881	9.701493	1.865672	1.492537	3.731343	16.41791	1.492537	0.373134	2.985075	9.328358	5.597015	2.238806	2.985075	5.970149	GM
SSA_1259	8.518519	3.333333	3.333333	4.074074	5.925926	7.037037	2.592593	5.925926	1.111111	2.222222	4.444444	9.62963	0.37037	0.37037	3.333333	3.703704	11.85185	8.888889	4.074074	9.259259	F
SSA_2114	8.185053	4.626335	5.69395	9.964413	6.405694	6.049822	2.135231	11.3879	1.423488	0.355872	2.135231	8.896797	0	0.355872	2.846975	3.558719	7.47331	6.049822	6.049822	6.405694	F

SSA_1044	7.986111	5.208333	4.166667	8.333333	6.944444	3.125	1.388889	8.680556	4.513889	1.736111	5.208333	10.06944	0.347222	0	3.472222	2.777778	5.208333	5.902778	5.208333	9.722222	E
SSA_0701	7.615894	4.635762	7.615894	5.960265	6.622517	5.629139	3.642384	7.94702	3.642384	1.986755	1.655629	12.25166	1.324503	0	4.966887	3.642384	1.986755	6.622517	5.960265	6.291391	P
SSA_1946	7.467532	3.896104	4.545455	9.415584	4.220779	8.116883	3.896104	12.33766	2.597403	0.324675	3.571429	9.090909	2.272727	0	4.220779	6.818182	5.844156	1.298701	4.545455	5.519481	EP
SSA_0272	7.443366	4.530744	3.883495	6.796117	3.883495	2.588997	3.236246	4.530744	6.472492	3.883495	5.501618	11.65049	0.970874	0.970874	3.883495	4.20712	5.177994	6.472492	5.177994	8.737864	R
SSA_1839	7.443366	3.236246	3.883495	5.501618	5.501618	5.177994	1.618123	8.737864	3.236246	0.970874	4.530744	7.443366	0.647249	0	2.912621	2.912621	11.97411	8.737864	3.236246	12.29773	E
SSA_1193	7.395498	2.893891	4.180064	3.215434	5.466238	5.466238	2.572347	8.360129	1.607717	3.536977	6.430868	8.038585	0	0.643087	3.536977	4.501608	6.752412	8.360129	5.787781	11.25402	EM
SSA_1742	7.395498	0.96463	4.501608	8.038585	11.25402	4.823151	0.96463	6.752412	3.536977	0.96463	3.858521	9.324759	1.286174	0.321543	5.466238	4.180064	6.109325	6.752412	7.395498	6.109325	P
SSA_0184	7.348243	7.98722	1.277955	6.389776	4.472843	3.833866	2.875399	7.028754	7.028754	2.236422	1.597444	10.54313	0.638978	1.277955	4.153355	3.833866	7.667732	8.945687	4.792332	6.070288	NU
SSA_2191	7.255521	4.416404	5.362776	5.047319	5.993691	4.416404	3.785489	7.570978	3.785489	3.154574	3.154574	8.832808	0.630915	0.315457	2.208202	2.523659	7.255521	7.886435	5.362776	11.04101	J
SSA_0813	7.142857	2.484472	4.037267	7.453416	6.521739	5.590062	0.621118	7.453416	4.658385	3.416149	4.037267	10.55901	0.621118	0.931677	3.10559	3.416149	8.385093	7.453416	4.658385	7.453416	O
SSA_1161	7.055215	0.613497	9.509202	7.668712	7.668712	7.668712	1.840491	6.441718	4.907975	0.306748	2.760736	9.815951	0.306748	0	3.374233	3.374233	6.134969	4.907975	6.134969	9.509202	S
SSA_1783	6.886228	4.191617	4.191617	4.491018	7.185629	6.586826	2.39521	4.790419	2.694611	2.39521	3.293413	9.281437	1.497006	0	3.293413	5.688623	7.185629	10.17964	6.886228	6.886228	F
SSA_0744	6.725146	5.263158	2.631579	5.555556	4.678363	6.432749	1.754386	5.263158	4.093567	2.923977	3.80117	11.11111	0.877193	0	2.923977	5.555556	7.894737	8.187135	5.555556	8.77193	J
SSA_2105	6.609195	6.609195	3.16092	6.321839	4.310345	2.873563	1.436782	7.471264	3.735632	2.586207	2.873563	7.758621	1.436782	0.574713	3.16092	5.45977	8.045977	9.770115	5.747126	10.05747	H
SSA_0449	6.515581	5.09915	1.983003	4.815864	4.815864	6.232295	2.832861	10.48159	2.832861	1.983003	2.549575	7.932011	0	1.133144	4.532578	5.09915	7.082153	8.215297	7.648725	8.215297	E
SSA_1855	5.989583	5.46875	3.385417	5.208333	8.333333	4.6875	2.604167	6.770833	4.947917	1.041667	3.645833	9.114583	1.5625	1.041667	2.604167	4.427083	7.03125	6.770833	6.25	9.114583	L
SSA_1179	5.927835	3.608247	3.865979	2.57732	7.989691	4.639175	2.835052	4.639175	4.123711	1.804124	4.896907	7.989691	0.515464	0.515464	6.185567	7.216495	5.670103	7.989691	7.474227	9.536082	I
SSA_2323	5.927835	2.061856	2.57732	7.731959	4.123711	6.701031	3.608247	8.505155	3.608247	1.030928	5.154639	19.58763	1.546392	0.773196	2.061856	5.927835	7.731959	1.28866	1.28866	8.762887	P
SSA_1511	5.542169	4.578313	5.301205	6.024096	6.746988	4.578313	1.445783	7.46988	4.578313	1.927711	4.578313	10.36145	1.445783	0.722892	4.096386	4.096386	5.301205	7.710843	5.301205	8.192771	M
SSA_0851	5.502392	4.066986	4.545455	8.373206	5.980861	4.784689	2.631579	10.28708	4.30622	2.15311	2.870813	10.52632	0.717703	0	2.392344	5.502392	4.066986	5.980861	6.698565	8.61244	P
SSA_1702	5.463183	3.325416	6.413302	10.68884	9.738717	4.988124	1.425178	8.551069	4.038005	0.950119	2.375297	4.750594	1.900238	0.475059	5.463183	5.463183	7.83848	5.700713	7.60095	2.850356	
SSA_2132	4.883227	3.184713	1.4862	8.917197	4.458599	3.821656	1.061571	8.917197	5.095541	0.424628	3.397028	16.98514	1.910828	1.273885	4.670913	9.129512	5.095541	2.335456	2.972399	9.978769	
SSA_0164	4.80167	1.25261	5.219207	11.69102	7.098121	6.680585	1.461378	5.427975	2.505219	1.878914	4.592902	5.845511	1.878914	1.461378	2.922756	4.384134	12.31733	4.80167	5.219207	8.559499	NU
SSA_0413	4.013962	5.410122	3.141361	4.188482	6.457243	8.027923	1.396161	6.282723	7.678883	1.745201	3.315881	11.51832	1.04712	1.22164	4.886562	2.792321	5.584642	9.424084	4.188482	7.678883	EH
SSA_1724	11.00917	4.12844	5.045872	4.587156	7.33945	6.880734	3.211009	8.715596	2.752294	1.834862	2.293578	10.55046	0	0	2.752294	2.752294	5.045872	8.715596	5.504587	6.880734	R
SSA_2119	10.9589	2.739726	5.022831	5.022831	5.022831	3.652968	2.739726	7.762557	2.739726	5.022831	4.56621	6.849315	0	0.456621	1.369863	5.022831	7.762557	6.392694	6.392694	10.50228	G
SSA_0193	10.66667	2.222222	3.111111	5.777778	4.888889	3.111111	4.444444	11.11111	4	1.777778	2.222222	15.11111	1.333333	0	2.222222	8.444444	7.111111	3.555556	0.888889	8	
SSA_0540	10.12658	0.843882	5.063291	3.375527	2.531646	6.329114	2.531646	6.751055	1.265823	1.265823	5.907173	10.97046	2.109705	0	3.375527	8.438819	12.65823	3.375527	0.843882	12.23629	G
SSA_0586	10.08403	2.521008	5.042017	4.621849	7.983193	6.302521	1.680672	5.042017	2.941176	0.840336	2.521008	5.462185	0.420168	0	2.941176	4.621849	8.403361	8.823529	9.243697	10.5042	S
SSA_0843	10.08403	1.680672	1.680672	5.462185	5.042017	8.823529	2.941176	7.142857	0.840336	2.10084	4.621849	16.38655	0.420168	0	3.361345	5.882353	9.663866	0.420168	2.10084	11.34454	M
SSA_1974	9.836066	1.639344	2.868852	11.47541	5.327869	3.278689	6.967213	8.606557	4.098361	1.639344	3.688525	15.98361	1.639344	0.409836	4.098361	5.737705	5.327869	1.639344	1.229508	4.508197	V
SSA_0759	9.795918	1.632653	2.44898	4.897959	8.163265	5.306122	2.44898	8.979592	7.346939	0.816327	2.040816	14.28571	0.408163	0	1.632653	0.816327	9.387755	5.306122	5.714286	8.571429	E
SSA_1566	9.486166	3.162055	5.533597	4.347826	7.509881	4.347826	4.347826	5.928854	3.557312	2.766798	3.557312	10.27668	0.395257	0	2.371542	4.347826	7.114625	6.719368	6.719368	7.509881	E
SSA_0700	9.448819	2.755906	3.149606	6.692913	5.11811	5.511811	2.755906	12.99213	3.149606	1.574803	1.181102	11.41732	2.755906	1.574803	4.724409	8.661417	7.480315	2.362205	1.968504	4.724409	
SSA_0475	9.411765	3.921569	1.176471	5.098039	6.27451	4.313725	1.960784	5.098039	5.490196	3.529412	3.921569	9.019608	0.392157	0.784314	5.882353	3.529412	6.27451	12.15686	3.921569	7.843137	H
SSA_0179	9.160305	3.435115	5.725191	4.580153	5.343511	3.053435	2.290076	6.10687	6.10687	3.053435	1.145038	14.50382	1.908397	0	4.580153	7.633588	3.053435	5.725191	6.10687	6.48855	
SSA_1559	9.090909	2.651515	4.545455	4.545455	6.439394	4.545455	3.409091	6.818182	3.030303	2.272727	1.893939	9.469697	0.378788	0.378788	3.787879	5.30303	6.439394	8.333333	8.333333	8.333333	R
SSA_0863	8.921933	2.230483	4.832714	3.717472	9.29368	5.947955	2.973978	8.178439	2.60223	1.486989	3.717472	10.03717	0.371747	0.371747	4.089219	1.486989	5.947955	9.665428	5.576208	8.550186	R
SSA_0017	8.856089	2.583026	6.273063	12.54613	8.487085	5.535055	2.214022	7.380074	3.321033	0	1.845018	9.594096	0.738007	0	2.214022	2.95203	5.166052	7.380074	5.535055	7.380074	M
SSA_1808	8.856089	2.583026	3.321033	5.166052	5.166052	4.797048	1.476015	4.797048	6.273063	3.690037	2.583026	11.07011	2.583026	1.107011	2.95203	3.321033	5.904059	10.3321	5.904059	8.118081	R
SSA_0365	8.362369	4.878049	4.529617	6.968641	4.181185	3.832753	2.787456	10.80139	4.181185	1.74216	3.832753	13.93728	0.696864	0	3.484321	4.529617	7.665505	3.832753	4.878049	4.878049	M
SSA_0264	8.053691	5.033557	3.691275	7.38255	4.362416	3.020134	2.684564	10.73826	5.033557	1.677852	3.020134	7.718121	0	1.006711	0.671141	3.691275	8.724832	7.04698	6.040268	10.40268	E
SSA_0055	7.973422	3.322259	1.993355	5.647841	2.325581	7.641196	1.993355	6.976744	2.990033	0.996678	2.990033	13.95349	1.328904	0.332226	3.654485	5.980066	10.63123	2.990033	3.322259	12.95681	G
SSA_1045	7.920792	2.640264	5.280528	6.930693	4.290429	3.630363	1.320132	7.260726	6.60066	0.660066	1.650165	12.21122	0.330033	0.990099	4.620462	6.930693	8.580858	5.280528	3.30033	9.570957	
SSA_0078	7.868852	4.590164	4.262295	5.245902	5.245902	3.606557	2.295082	7.868852	4.262295	0.983607	4.590164	9.508197	0.327869	0.983607	4.590164	2.95082	8.196721	8.52459	3.934426	10.16393	EM
SSA_1716	7.792208	4.545455	4.87013	7.142857	8.441558	4.220779	1.623377	4.545455	3.246753	1.623377	2.922078	11.68831	1.623377	0.324675	4.87013	5.844156	4.545455	7.467532	7.467532	5.194805	
SSA_1944	7.792208	4.545455	3.571429	5.844156	8.441558	3.246753	1.298701	8.766234	3.896104	2.597403	3.896104	8.441558	0.324675	0	3.896104	4.545455	6.168831	9.415584	6.168831	7.142857	R
SSA_0420	7.741935	5.16129	2.580645	4.193548	6.451613	2.580645	2.903226	6.129032	2.580645	2.903226	2.580645	10.32258	0.967742	0.967742	3.548387	3.870968	10.64516	8.387097	7.096774	8.387097	R
SSA_1965	7.741935	4.516129	5.483871	5.806452	6.774194	5.16129	2.258065	10.64516	5.483871	0.645161	2.258065	9.677419	0	0	3.225806	4.193548	3.870968	8.709677	4.193548	9.354839	O
SSA_1242	7.692308	2.884615	5.128205	5.448718	5.448718	5.128205	2.884615	7.371795	2.884615	0.641026	7.051282	9.294872	0.320513	0.961538	2.884615	3.205128	8.974359	6.089744	3.525641	12.17949	F
SSA_1090	7.523511	2.821317	4.388715	4.702194	5.956113	5.329154	1.567398	9.090909	2.507837	1.253918	2.194357	10.03135	0.940439	1.253918	2.194357	2.821317	12.85266	6.583072	6.583072	9.404389	KG
SSA_0496	7.33945	3.975535	3.058104	8.868502	3.669725	3.975535	2.446483	5.198777	6.727829	2.446483	4.281346	11.31498	0.917431	2.140673	5.504587	2.752294	6.422018	7.33945	4.892966	6.727829	R
SSA_2087	7.33945	4.892966	5.504587	7.033639	7.64526	7.033639	2.752294	7.95107	5.504587	0.611621	2.752294	9.480122	0.30581	0.611621	3.669725	3.669725	5.198777	7.033639	5.504587	5.504587	K
SSA_0026	7.228916	3.915663	3.915663	6.626506	8.13253	4.819277	2.710843	6.024096	2.108434	1.506024	2.409639	9.939759	0.301205	0	2.108434	3.012048	10.84337	6.024096	6.927711	11.44578	I
SSA_1489	7.142857	3.571429	7.142857	6.845238	12.20238	3.869048	2.083333	6.547619	3.27381	0.892857	3.571429	6.25	1.488095	0.297619	7.440476	5.357143	2.97619	8.928571	5.357143	4.761905	
SSA_1225	7.079646	3.539823	3.244838	4.424779	5.309735	6.784661	1.474926	6.19469	2.654867	2.359882	6.489676	8.849558	0.884956	0.589971	5.60472	4.719764	10.0295	6.784661	5.60472	7.374631	EH
SSA_0546	6.997085	5.539359	6.413994	5.247813	5.830904	5.247813	2.623907	6.705539	4.956268	2.332362	3.206997	9.037901	0.58309	0.58309	4.373178	2.915452	7.28863	8.163265	5.539359	6.413994	E
SSA_0977	6.956522	4.637681	2.608696	5.797101	4.927536	4.347826	2.608696	7.826087	2.898551	2.028986	4.347826	9.855072	0.289855	0	2.318841	2.898551	8.115942	7.826087	6.086957	13.62319	CE
SSA_1138	6.722689	4.201681	4.201681	4.761905	6.722689	4.761905	2.80112	4.201681	2.521008	2.521008	2.240896	7.002801	1.120448	1.120448	3.361345	4.481793	9.803922	9.803922	6.722689	10.92437	C
SSA_1577	6.666667	2.777778	5.277778	3.888889	5.277778	5.555556	3.333333	6.944444	3.333333	3.055556	4.444444	8.611111	0.277778	0.833333	4.444444	5.833333	7.222222	8.333333	5.833333	8.055556	E
SSA_0583	6.593407	4.945055	3.021978	6.043956	5.769231	4.395604	1.923077	5.21978	6.868132	2.197802	3.846154	11.53846	1.098901	0.549451	3.846154	4.395604	7.142857	6.868132	4.120879	9.615385	R
SSA_0094	6.575342	1.369863	6.575342	4.931507	2.191781	9.041096	1.643836	4.657534	6.575342	1.369863	7.123288	5.205479	0.821918	0	3.013699	1.643836	5.205479	8.767123	4.383562	18.90411	S
SSA_1072	6.504065	3.523035	3.794038	8.672087	8.130081	5.691057	1.626016	5.420054	4.065041	1.084011	1.897019	13.00813	0.542005	0.271003	2.168022	2.710027	8.401084	5.149051	6.233062	11.11111	E

SSA_1605	6.504065	4.878049	3.523035	7.859079	2.710027	3.252033	4.336043	7.588076	2.710027	0.813008	3.794038	14.36314	0.542005	0.271003	4.336043	8.130081	10.84011	2.710027	1.355014	9.485095	G
SSA_0072	6.366048	5.039788	4.509284	4.244032	6.896552	4.509284	2.917772	8.222812	3.713528	1.591512	3.183024	8.753316	0	0.530504	3.448276	4.509284	7.692308	7.427056	7.161804	9.28382	G
SSA_1324	6.060606	5.30303	3.787879	6.565657	5.30303	3.535354	1.767677	8.333333	4.040404	1.262626	4.545455	16.16162	2.525253	0.757576	4.79798	5.555556	3.282828	5.050505	5.555556	5.808081	M
SSA_1687	6.030151	2.261307	4.020101	4.522613	7.537688	5.025126	2.512563	8.291457	3.517588	3.517588	2.763819	11.80905	1.507538	0.251256	6.532663	7.035176	8.291457	3.015075	3.768844	7.788945	P
SSA_1260	5.955335	4.466501	6.451613	4.218362	4.466501	5.955335	2.48139	7.940447	1.240695	3.473945	5.955335	7.444169	0.496278	0.496278	2.977667	4.962779	9.181141	7.444169	7.19603	7.19603	G
SSA_2370	5.568445	2.552204	4.640371	6.728538	5.568445	7.192575	2.552204	6.49652	4.640371	2.784223	3.712297	10.67285	0.232019	0.232019	3.480278	7.192575	5.568445	9.048724	6.264501	4.87239	R
SSA_2050	5.298013	2.649007	3.532009	5.298013	4.415011	8.388521	2.428256	9.271523	2.649007	1.545254	4.19426	16.77704	0.883002	0.883002	1.986755	9.050773	6.843267	1.986755	2.428256	9.492274	G
SSA_0395	5.010438	4.592902	4.384134	3.131524	6.889353	5.010438	2.922756	5.636743	1.878914	2.296451	3.549061	7.515658	2.505219	1.670146	5.636743	5.219207	8.559499	9.185804	6.680585	7.724426	G
SSA_1854	4.968944	2.898551	5.590062	10.76605	8.488613	5.797101	0.414079	4.140787	5.383023	0.828157	4.554865	7.039337	0.414079	0.207039	3.726708	3.519669	8.281573	8.281573	5.383023	9.31677	S
SSA_2266	4.771372	5.168986	3.578529	5.367793	5.367793	5.5666	3.180915	5.765408	2.584493	1.988072	4.17495	7.157058	2.982107	0.397614	5.367793	5.5666	5.765408	7.753479	8.151093	9.343936	G
SSA_0724	4.562738	3.422053	5.323194	8.174905	6.463878	4.942966	2.281369	7.794677	5.703422	1.711027	1.330798	12.35741	0.95057	1.330798	3.422053	7.984791	3.802281	5.13308	6.08365	7.224335	V
SSA_0398	4.054054	3.547297	4.222973	6.587838	6.25	7.77027	3.209459	6.925676	3.716216	1.52027	3.716216	12.66892	2.027027	0	5.067568	4.72973	7.094595	5.743243	5.236486	5.912162	V
SSA_1639	10.86957	3.478261	1.73913	4.782609	5.652174	4.782609	3.913043	7.391304	3.043478	4.347826	2.173913	8.695652	0	0	3.043478	3.913043	7.826087	6.956522	6.086957	11.30435	F
SSA_0532	10.68376	4.273504	4.273504	6.410256	3.418803	5.982906	2.136752	7.264957	2.564103	1.709402	2.991453	5.128205	0	0.854701	2.991453	2.564103	12.39316	5.982906	5.555556	12.82051	E
SSA_0477	9.615385	1.153846	2.692308	8.461538	6.538462	6.153846	3.461538	7.692308	1.538462	1.538462	3.846154	14.23077	0.769231	1.538462	1.923077	8.076923	7.692308	3.076923	1.153846	8.846154	P
SSA_1919	9.328358	1.492537	1.865672	5.223881	2.61194	2.61194	4.850746	9.328358	2.61194	1.119403	2.985075	12.31343	1.119403	0.373134	1.119403	4.104478	12.68657	2.985075	2.985075	18.28358	G
SSA_0677	9.225092	5.904059	1.476015	4.797048	7.380074	3.690037	2.583026	4.428044	5.904059	1.107011	2.583026	9.9631	0.369004	0	2.583026	5.166052	8.118081	8.118081	5.904059	10.70111	J
SSA_1592	9.157509	3.296703	4.029304	5.860806	5.128205	5.860806	0.732601	8.058608	1.465201	1.098901	1.465201	12.82051	1.465201	0.3663	4.029304	9.52381	8.424908	4.761905	2.930403	9.52381	S
SSA_0076	9.025271	2.166065	3.971119	7.942238	4.33213	6.137184	2.166065	15.16245	1.444043	1.083032	4.33213	10.1083	1.805054	0	3.610108	6.859206	5.776173	2.166065	2.888087	9.025271	G
SSA_0682	9.025271	3.249097	2.527076	7.942238	7.220217	5.415162	2.888087	8.303249	4.693141	1.444043	3.249097	9.747292	0.722022	0	1.805054	2.166065	6.498195	9.025271	5.415162	8.66426	S
SSA_0221	8.896797	1.067616	1.423488	6.405694	4.626335	6.405694	3.202847	9.608541	1.423488	1.423488	4.270463	12.09964	1.779359	0.355872	2.846975	5.69395	10.67616	2.846975	3.558719	11.3879	G
SSA_0858	8.802817	3.521127	4.577465	3.169014	7.042254	9.507042	2.112676	3.521127	3.873239	1.760563	3.169014	5.985915	1.760563	0.352113	5.633803	4.577465	5.28169	9.15493	7.042254	9.15493	M
SSA_2035	8.710801	3.484321	1.74216	6.271777	5.923345	7.665505	3.135889	5.923345	4.529617	1.393728	3.484321	11.14983	0.348432	0	1.74216	2.439024	7.665505	7.665505	6.271777	10.45296	S
SSA_1329	8.561644	3.767123	2.739726	8.90411	4.794521	3.767123	1.027397	6.849315	6.164384	2.39726	4.109589	13.0137	0.342466	0.684932	5.136986	4.452055	4.794521	7.876712	5.821918	4.794521	
SSA_0424	8.250825	2.310231	7.590759	13.20132	4.620462	9.570957	1.650165	7.590759	2.310231	0.330033	0.990099	5.610561	0	0	6.60066	2.640264	7.260726	5.280528	5.610561	8.580858	G
SSA_0520	8.169935	3.921569	2.614379	4.575163	9.150327	4.901961	2.941176	7.189542	4.575163	0.980392	4.248366	7.189542	0.326797	0.653595	2.287582	3.921569	6.535948	8.169935	6.862745	10.78431	E
SSA_1068	8.064516	5.806452	5.16129	6.451613	5.806452	6.451613	2.903226	5.483871	5.806452	1.612903	2.903226	8.064516	0	0	5.16129	6.129032	4.516129	9.354839	5.483871	4.83871	K
SSA_1547	8.038585	7.073955	2.250804	7.717042	6.752412	4.501608	4.180064	8.360129	3.215434	1.92926	2.572347	9.646302	0.643087	0	3.858521	1.607717	6.752412	8.360129	6.752412	5.787781	T
SSA_0781	7.98722	3.194888	2.875399	7.028754	5.43131	5.111821	0.958466	5.750799	3.514377	3.514377	4.472843	10.22364	2.236422	0.319489	3.833866	3.833866	8.306709	7.667732	7.348243	6.389776	G
SSA_0241	7.861635	3.144654	2.515723	4.716981	4.08805	5.660377	2.515723	7.861635	3.773585	1.572327	2.515723	9.119497	1.886792	0.314465	3.144654	2.515723	8.805031	11.32075	7.232704	9.433962	J
SSA_0551	7.8125	2.1875	4.0625	4.0625	6.25	7.1875	3.75	6.875	3.4375	2.8125	4.6875	10.625	0	0.3125	3.125	3.4375	8.4375	5.625	6.25	9.0625	EJ
SSA_1279	7.8125	4.0625	3.75	5.3125	4.6875	6.25	2.1875	8.125	3.75	2.1875	3.75	9.0625	0.9375	0.625	2.8125	5	8.75	9.6875	5	6.25	CR
SSA_1584	7.763975	6.21118	3.10559	5.590062	5.279503	3.10559	1.242236	4.037267	5.590062	3.416149	2.795031	12.1118	1.552795	0	4.347826	2.795031	7.763975	8.385093	5.590062	9.31677	R
SSA_0924	7.440476	2.97619	2.678571	8.333333	3.869048	4.166667	2.97619	9.52381	5.952381	0.595238	2.380952	13.09524	1.190476	0	3.571429	5.654762	7.738095	4.761905	4.761905	8.333333	M
SSA_0757	7.352941	3.823529	3.823529	5.588235	5	4.411765	1.764706	7.352941	6.176471	2.941176	5	10.58824	0.294118	0.882353	4.411765	4.411765	6.176471	5.294118	5.882353	8.823529	E
SSA_1581	7.309942	0.877193	6.725146	8.77193	10.81871	7.894737	3.216374	6.432749	4.385965	0	2.923977	7.894737	0.292398	0.584795	3.508772	1.754386	3.80117	7.017544	7.309942	8.479532	P
SSA_1928	7.062147	5.367232	4.519774	8.19209	2.259887	2.824859	0.282486	4.80226	5.084746	1.694915	2.824859	13.27684	1.694915	0.564972	2.259887	5.932203	7.627119	9.60452	4.80226	9.322034	I
SSA_2317	7.062147	6.779661	4.80226	4.237288	4.237288	7.909605	3.954802	8.474576	5.084746	2.824859	3.107345	11.01695	0	0.847458	1.977401	1.694915	7.627119	6.779661	4.80226	6.779661	NU
SSA_2129	6.925208	3.047091	3.047091	6.925208	9.418283	3.3241	1.385042	4.155125	4.98615	1.939058	3.3241	16.89751	1.108033	0.831025	4.98615	3.878116	2.770083	9.972299	6.648199	4.432133	K
SSA_0287	6.868132	3.021978	2.747253	4.67033	5.494505	6.043956	1.373626	7.692308	4.120879	3.021978	2.747253	10.16484	0.274725	0.824176	3.296703	3.571429	8.516484	7.692308	5.21978	12.63736	C
SSA_0290	6.56168	2.362205	4.724409	6.299213	3.674541	7.349081	5.249344	9.186352	2.099738	4.724409	2.099738	14.17323	1.312336	0.262467	4.986877	9.973753	5.249344	2.887139	1.574803	5.249344	S
SSA_1003	5.966587	2.147971	5.48926	5.727924	8.830549	6.205251	2.147971	4.534606	2.863962	1.193317	3.818616	7.637232	2.147971	0.238663	4.77327	4.295943	8.114558	6.443914	6.921241	10.50119	G
SSA_0608	5.854801	3.044496	1.873536	9.367681	3.981265	4.449649	2.34192	9.836066	2.107728	0.936768	2.107728	15.92506	2.107728	1.405152	3.044496	7.494145	11.24122	1.17096	3.044496	8.665105	
SSA_1135	5.592841	3.131991	2.46085	7.158837	3.579418	4.697987	2.908277	8.277405	2.684564	1.789709	3.803132	17.44966	1.565996	0.223714	5.145414	7.606264	8.501119	2.237136	3.131991	8.053691	V
SSA_0960	5.399568	4.319654	5.831533	9.935205	8.855292	6.047516	2.591793	7.343413	3.671706	0.863931	3.671706	9.287257	0.215983	0.215983	3.887689	5.831533	3.887689	5.399568	7.343413	5.399568	T
SSA_1373	4.92126	5.511811	2.755906	6.299213	8.858268	6.102362	1.377953	4.724409	6.102362	2.952756	2.952756	14.76378	0.590551	0.590551	2.559055	3.149606	4.92126	8.464567	5.905512	6.496063	R
SSA_2307	4.734848	1.704545	7.765152	15.7197	7.19697	10.79545	1.325758	4.356061	4.356061	0.378788	3.030303	8.143939	0.94697	0.189394	3.598485	2.651515	7.386364	6.25	3.219697	6.25	G
SSA_1506	4.496403	4.856115	3.776978	8.453237	5.57554	4.136691	1.978417	4.136691	6.834532	2.877698	3.956835	14.56835	1.798561	0.719424	4.316547	6.47482	3.417266	6.294964	6.654676	4.676259	M
SSA_1777	10.7438	8.264463	4.132231	4.545455	10.7438	5.371901	1.239669	7.024793	3.719008	2.479339	3.719008	8.677686	0.413223	0	3.305785	1.239669	7.438017	7.438017	4.958678	4.545455	J
SSA_0733	9.923664	3.053435	2.671756	3.435115	4.580153	3.435115	1.145038	9.923664	4.580153	2.290076	3.435115	17.17557	2.290076	0	3.816794	4.580153	6.48855	3.816794	1.908397	11.45038	S
SSA_0137	9.701493	2.985075	2.238806	9.701493	2.985075	6.343284	5.223881	10.8209	1.492537	0.373134	2.61194	15.29851	0	0	2.985075	7.835821	5.970149	1.865672	2.238806	9.328358	P
SSA_2366	9.318996	3.225806	2.867384	6.451613	6.09319	5.376344	2.867384	6.09319	4.301075	2.508961	4.301075	10.39427	0	0	1.792115	5.734767	8.960573	7.885305	5.376344	6.451613	P
SSA_0671	9.15493	4.577465	4.225352	3.873239	7.042254	5.28169	3.521127	7.042254	3.521127	2.816901	5.28169	9.859155	0.704225	0	1.408451	2.464789	8.450704	7.394366	4.577465	8.802817	H
SSA_2367	8.813559	5.084746	4.40678	7.118644	5.762712	4.745763	2.711864	6.101695	6.101695	0.677966	3.050847	12.20339	1.016949	0	1.694915	2.372881	6.440678	9.152542	7.79661	4.745763	P
SSA_2175	8.724832	5.033557	3.355705	11.74497	9.060403	6.375839	2.013423	5.033557	6.711409	0	3.020134	9.395973	1.006711	0.33557	3.691275	2.348993	6.040268	6.711409	5.369128	4.026846	
SSA_0434	8.695652	5.351171	5.351171	4.347826	6.020067	3.67893	2.006689	8.026756	2.675585	2.006689	4.682274	8.361204	0.334448	1.003344	3.67893	3.344482	7.35786	8.695652	5.685619	8.695652	E
SSA_1611	8.695652	5.016722	3.010033	4.347826	9.698997	5.016722	5.016722	9.0301	3.67893	2.341137	3.67893	7.35786	0.668896	0	1.672241	4.347826	6.020067	7.023411	8.361204	5.016722	R
SSA_0329	8.637874	1.328904	4.318937	5.980066	3.986711	5.315615	2.325581	6.312292	3.654485	2.325581	5.315615	10.63123	1.993355	0	2.657807	6.312292	11.29568	3.322259	1.66113	12.62458	G
SSA_0500	8.306709	4.153355	3.194888	10.86262	5.111821	4.472843	2.236422	5.43131	4.792332	0.638978	4.153355	20.44728	1.916933	0.638978	2.555911	5.750799	5.111821	2.555911	2.555911	5.111821	EP
SSA_1212	8.176101	4.716981	5.031447	7.54717	6.603774	3.773585	1.886792	9.433962	2.515723	1.886792	4.08805	7.861635	0	0.943396	3.459119	3.459119	6.289308	6.603774	6.603774	9.119497	FE
SSA_1696	7.97546	3.374233	3.067485	5.521472	8.588957	4.601227	1.840491	3.680982	4.294479	1.226994	3.067485	9.509202	1.226994	0.920245	4.294479	3.98773	5.521472	10.42945	5.828221	11.04294	G
SSA_0497	7.854985	3.625378	3.021148	5.438066	3.625378	5.135952	2.416918	9.365559	3.021148	2.114804	5.135952	16.91843	1.510574	0.302115	3.625378	5.740181	8.459215	4.229607	1.510574	6.94864	EP
SSA_2102	7.807808	4.204204	3.003003	4.204204	5.105105	3.303303	0.600601	8.708709	3.903904	1.801802	3.303303	8.708709	1.801802	0.600601	2.702703	4.504505	8.408408	7.507508	6.906907	12.91291	H

SSA_0753	7.761194	0.298507	3.880597	11.9403	14.02985	5.970149	1.19403	5.074627	4.179104	0	0.895522	4.477612	0.298507	0.298507	3.58209	3.58209	4.179104	8.955224	6.865672	12.53731	O
SSA_0738	7.692308	4.43787	4.43787	3.846154	6.508876	5.91716	2.662722	4.142012	2.366864	3.254438	2.366864	10.35503	0.887574	0.591716	2.95858	5.621302	7.100592	9.467456	5.91716	9.467456	E
SSA_2106	7.449857	5.157593	1.719198	4.87106	6.017192	5.444126	2.005731	6.017192	4.297994	2.578797	4.584527	10.02865	1.432665	0	4.011461	3.438395	8.022923	9.74212	4.87106	8.309456	C
SSA_1415	7.142857	4.945055	1.098901	5.769231	6.593407	4.67033	1.098901	3.846154	5.21978	0.824176	4.120879	12.91209	0.824176	0.274725	4.120879	3.571429	8.791209	6.868132	7.142857	10.16484	E
SSA_0492	7.084469	6.811989	2.452316	5.99455	5.449591	3.269755	1.362398	7.084469	6.26703	1.634877	5.177112	9.809264	0.817439	0.27248	2.997275	3.814714	7.084469	6.811989	7.084469	8.719346	C
SSA_1057	6.842105	3.421053	2.105263	8.947368	7.631579	7.368421	1.842105	8.684211	3.947368	2.105263	3.684211	8.157895	0.789474	0.263158	2.368421	5	6.578947	6.578947	5.263158	8.421053	E
SSA_0722	6.735751	3.626943	4.92228	5.440415	7.512953	5.699482	4.145078	6.476684	4.663212	0.518135	3.367876	8.031088	0.518135	0.518135	7.253886	3.626943	5.699482	9.067358	4.92228	7.253886	V
SSA_1008	6.632653	5.867347	5.612245	5.102041	4.591837	4.336735	1.785714	5.102041	2.806122	2.806122	1.785714	11.22449	0.765306	0.510204	3.571429	4.591837	9.183673	8.928571	6.122449	8.673469	G
SSA_0704	6.532663	3.517588	3.266332	4.271357	7.035176	4.271357	1.005025	8.040201	4.522613	1.758794	4.020101	10.30151	1.507538	1.005025	2.512563	3.768844	8.291457	7.537688	6.281407	10.55276	C
SSA_1787	6.25	4.086538	4.567308	4.326923	5.528846	5.048077	2.644231	6.009615	3.365385	1.923077	4.567308	8.894231	0.240385	0.961538	4.567308	5.769231	8.413462	9.134615	5.048077	8.653846	E
SSA_1494	6.205251	3.818616	4.534606	5.011933	4.534606	6.682578	3.579952	11.21718	3.818616	2.147971	4.057279	8.830549	0	0.238663	3.102625	1.431981	10.02387	5.727924	5.250597	9.785203	M
SSA_2324	6.190476	4.52381	3.809524	4.52381	6.428571	5.238095	2.380952	4.52381	5.47619	2.142857	5	10.2381	0.714286	1.904762	5.238095	4.047619	5.952381	7.857143	9.047619	4.761905	M
SSA_0046	6.032483	7.424594	4.176334	4.87239	5.104408	6.49652	3.24826	8.12065	3.016241	2.320186	3.480278	6.960557	1.62413	0.232019	3.016241	4.408353	5.336427	10.90487	4.87239	8.352668	F
SSA_2340	5.990783	1.382488	5.990783	11.98157	5.760369	11.98157	1.382488	8.294931	2.073733	0.691244	0.921659	5.760369	0	0.230415	2.073733	1.152074	11.52074	3.225806	8.75576	10.82949	
SSA_1377	5.803571	4.6875	2.901786	4.464286	6.25	5.133929	3.125	6.25	2.678571	2.678571	4.241071	7.8125	0.892857	0.223214	4.910714	6.25	7.8125	8.928571	7.366071	7.589286	J
SSA_1823	5.739514	5.077263	2.649007	6.622517	5.518764	3.311258	2.869757	6.181015	5.077263	2.869757	4.19426	7.94702	0.883002	0.441501	2.428256	6.401766	4.635762	11.47903	7.94702	7.726269	S
SSA_2376	5.726872	3.303965	6.387665	7.268722	8.14978	3.744493	2.202643	6.60793	3.0837	1.982379	1.982379	12.11454	1.321586	0.220264	1.982379	4.845815	5.726872	8.590308	8.810573	5.947137	R
SSA_1262	5.689278	5.251641	6.126915	4.595186	5.032823	7.002188	2.407002	9.846827	4.595186	1.094092	2.407002	12.25383	0.218818	0.218818	1.094092	2.407002	5.908096	9.846827	6.78337	7.221007	R
SSA_1149	5.591398	6.021505	5.376344	4.301075	5.16129	3.870968	1.505376	7.741935	3.655914	2.795699	3.44086	8.387097	3.010753	0.430108	6.236559	4.731183	7.526882	7.526882	7.311828	5.376344	G
SSA_0138	5.2	1.4	3.8	5.4	10.4	6.4	1.8	4	3.2	2.8	3.2	8.6	0.8	0.2	6.4	3.4	5.8	9.8	7.2	10.2	R
SSA_0987	5.13834	2.173913	4.347826	3.952569	7.312253	6.521739	2.766798	7.509881	3.952569	1.383399	4.347826	15.21739	0.790514	0	3.162055	4.150198	7.312253	5.928854	4.150198	9.881423	M
SSA_0833	4.942966	5.893536	3.802281	4.752852	4.562738	3.422053	1.330798	8.174905	7.224335	3.041825	3.992395	11.21673	1.901141	0.190114	6.273764	4.752852	4.18251	7.794677	7.984791	4.562738	
SSA_1844	11.68831	3.463203	5.194805	3.896104	6.060606	4.329004	3.030303	9.090909	3.463203	3.030303	2.164502	14.71861	0.865801	0.4329	4.761905	6.060606	3.896104	5.194805	4.761905	3.896104	S
SSA_1243	10.07463	4.477612	1.865672	6.343284	4.477612	3.358209	4.850746	7.089552	5.597015	1.865672	4.104478	7.462687	0	2.61194	3.358209	3.358209	9.328358	5.597015	7.089552	7.089552	HC
SSA_1959	9.642857	2.5	2.5	5	5	3.928571	2.5	11.07143	3.571429	1.428571	3.214286	11.78571	1.785714	0	3.571429	8.571429	9.642857	3.571429	2.5	8.214286	V
SSA_2123	9.310345	5.172414	4.137931	5.862069	6.896552	5.517241	2.413793	7.241379	2.758621	1.034483	4.482759	8.965517	0.344828	0.344828	2.758621	6.206897	3.793103	7.586207	5.172414	10	J
SSA_1081	8.910891	2.970297	3.960396	6.930693	7.590759	6.270627	1.320132	8.250825	2.640264	0.990099	2.310231	8.250825	0.330033	0.660066	2.310231	4.950495	10.56106	7.590759	6.930693	6.270627	G
SSA_1490	8.910891	3.30033	3.960396	7.920792	5.940594	4.290429	3.960396	6.930693	2.970297	1.650165	2.640264	14.85149	1.320132	0.660066	4.290429	9.90099	3.30033	3.630363	3.30033	6.270627	S
SSA_1343	8.852459	5.245902	3.606557	6.557377	5.57377	4.918033	3.934426	6.229508	3.606557	3.934426	2.622951	8.852459	0.327869	0.655738	3.278689	4.262295	5.901639	9.508197	6.229508	5.901639	F
SSA_2085	8.490566	4.402516	3.773585	4.402516	7.54717	4.716981	2.830189	5.974843	1.572327	2.201258	4.402516	9.433962	0.628931	0	3.773585	4.08805	8.176101	9.119497	5.345912	9.119497	CHR
SSA_0593	8.231707	6.402439	5.182927	3.04878	6.707317	3.658537	2.134146	5.182927	2.134146	2.439024	3.353659	7.317073	1.52439	2.439024	5.182927	3.963415	7.926829	10.06098	8.231707	4.878049	R
SSA_1173	8.206687	5.471125	5.775076	5.471125	7.902736	3.951368	1.215805	9.118541	1.215805	1.823708	1.519757	6.990881	0.911854	0.607903	4.559271	5.471125	6.382979	10.94225	6.68693	5.775076	H
SSA_0070	8.108108	5.105105	3.303303	4.204204	5.105105	4.504505	2.402402	4.504505	3.903904	2.402402	3.303303	10.51051	1.501502	1.201201	5.405405	5.705706	7.807808	6.606607	7.507508	6.906907	Q
SSA_0831	8.083832	3.892216	4.191617	8.682635	4.491018	2.694611	2.994012	5.688623	3.892216	1.497006	5.688623	11.07784	1.497006	0	5.988024	4.491018	5.688623	8.083832	5.08982	6.287425	M
SSA_2168	7.941176	3.823529	5.882353	4.411765	6.176471	6.470588	1.764706	9.117647	2.647059	2.941176	2.058824	8.235294	1.176471	0.294118	3.529412	1.764706	7.647059	8.823529	4.705882	10.58824	C
SSA_0626	7.78098	2.305476	4.899135	6.051873	9.221902	8.069164	2.017291	7.492795	3.746398	0.288184	2.881844	6.628242	0.864553	0	4.034582	4.322767	8.933718	6.916427	6.051873	7.492795	T
SSA_2213	7.627119	5.367232	4.519774	7.909605	5.367232	5.649718	2.824859	5.649718	3.389831	1.694915	1.977401	11.01695	0.564972	1.129944	4.80226	2.824859	5.084746	7.627119	6.214689	8.757062	MG
SSA_0077	7.356948	3.269755	4.359673	4.359673	6.53951	4.359673	3.814714	5.722071	2.997275	4.087193	2.452316	6.811989	1.362398	2.179837	3.542234	3.814714	8.446866	10.35422	5.722071	8.446866	R
SSA_1118	7.317073	3.252033	4.065041	7.04607	10.2981	5.149051	1.084011	5.149051	3.523035	1.897019	4.065041	6.504065	1.084011	1.355014	6.504065	3.794038	7.317073	8.401084	6.504065	5.691057	O
SSA_1007	7.142857	5.555556	5.026455	5.291005	6.878307	5.291005	3.439153	6.613757	2.116402	2.116402	3.703704	7.936508	0	0.529101	3.174603	5.026455	7.671958	7.407407	6.878307	8.201058	G
SSA_2245	7.068063	3.664921	3.141361	5.235602	9.424084	3.664921	2.356021	8.115183	3.664921	1.04712	2.879581	7.591623	0.26178	0	2.356021	2.356021	10.20942	8.638743	7.591623	10.73298	L
SSA_1893	7.049608	2.872063	3.133159	4.177546	5.221932	6.005222	3.394256	5.483029	3.133159	3.394256	3.655352	9.399478	0.78329	1.044386	4.699739	3.133159	10.70496	6.527415	7.049608	9.138381	G
SSA_1737	7.012987	3.896104	3.896104	6.753247	4.935065	8.311688	1.818182	5.194805	2.597403	2.077922	3.896104	11.16883	0.519481	0	4.155844	4.415584	6.493506	7.012987	6.233766	9.61039	E
SSA_1729	6.994819	0.518135	3.626943	6.217617	10.62176	9.067358	1.554404	5.699482	3.367876	0.518135	2.072539	4.663212	0	0.259067	3.88601	4.663212	8.031088	6.476684	6.994819	14.76684	E
SSA_1342	6.958763	4.896907	4.123711	4.639175	4.123711	5.670103	3.608247	7.474227	4.639175	3.608247	4.896907	7.989691	0.515464	1.28866	3.608247	4.123711	8.762887	6.185567	6.185567	6.701031	EF
SSA_1376	6.835443	2.278481	3.037975	10.37975	4.303797	4.303797	4.810127	8.860759	3.544304	1.265823	2.531646	13.41772	2.78481	0.506329	5.063291	7.341772	6.329114	3.291139	2.025316	7.088608	G
SSA_0832	6.633907	3.931204	3.439803	7.125307	3.685504	4.668305	6.633907	10.07371	2.948403	0.4914	3.685504	15.72482	1.965602	0	4.422604	5.896806	5.651106	3.194103	2.702703	7.125307	U
SSA_2128	6.53753	1.452785	4.358354	10.41162	4.116223	3.1477	3.631961	8.474576	4.358354	0.968523	2.663438	20.09685	1.210654	1.210654	2.905569	6.779661	5.569007	2.663438	2.179177	7.263923	
SSA_2371	6.443914	5.966587	5.011933	6.921241	6.682578	3.579952	3.102625	5.966587	5.966587	2.147971	1.670644	11.69451	0.238663	0	3.341289	6.682578	4.534606	8.591885	4.295943	7.159905	R
SSA_0216	6.17849	6.17849	4.805492	8.237986	4.805492	2.745995	2.745995	10.98398	1.830664	2.745995	1.830664	12.81465	0.915332	0.228833	5.263158	4.805492	5.034325	7.093822	5.034325	5.720824	T
SSA_2379	6.081081	4.72973	3.828829	9.234234	5.630631	5.18018	2.027027	11.48649	3.828829	2.027027	1.576577	12.38739	0.675676	1.126126	3.603604	8.783784	2.702703	6.531532	4.954955	3.603604	T
SSA_1869	6.040268	6.935123	2.908277	4.474273	10.29083	6.263982	2.013423	7.606264	5.369128	1.565996	3.803132	8.501119	0.447427	0	2.684564	4.250559	5.592841	6.263982	6.935123	8.053691	LKJ
SSA_1460	6.013363	5.122494	4.899777	5.567929	8.017817	6.23608	3.11804	3.786192	5.122494	1.55902	3.786192	9.35412	0.445434	0	4.67706	4.231626	8.017817	6.23608	8.908686	4.899777	K
SSA_1299	5.973451	1.327434	4.646018	7.300885	5.088496	5.752212	3.539823	9.734513	3.982301	1.327434	3.761062	13.49558	1.99115	0.663717	2.433628	8.628319	7.079646	1.99115	3.097345	8.185841	G
SSA_1366	5.934066	4.175824	3.516484	7.692308	5.274725	5.274725	2.637363	4.835165	4.615385	2.197802	3.296703	13.84615	1.978022	0.43956	5.274725	5.714286	7.912088	3.956044	5.274725	6.153846	V
SSA_0380	5.869565	3.695652	2.826087	5.217391	6.086957	5.434783	3.26087	5.434783	6.086957	2.826087	4.347826	9.347826	2.173913	0	4.347826	3.913043	7.608696	8.695652	6.304348	6.521739	M
SSA_0383	5.636743	4.175365	5.427975	4.80167	5.010438	4.175365	1.670146	5.219207	3.131524	2.296451	5.427975	9.60334	1.670146	0.417537	6.471816	5.010438	8.768267	6.680585	7.098121	7.306889	G
SSA_0455	5.578512	3.305785	5.578512	4.545455	6.818182	4.752066	1.446281	4.958678	4.545455	1.859504	4.958678	8.264463	1.652893	1.033058	5.578512	5.991736	7.231405	5.991736	8.677686	7.231405	G
SSA_2082	5.325444	4.33925	3.155819	5.91716	4.733728	5.128205	2.564103	6.508876	5.522682	1.775148	2.761341	9.861933	1.380671	0.986193	3.944773	4.33925	7.692308	8.87574	4.930966	10.25641	G
SSA_0602	4.812834	4.278075	2.495544	6.417112	5.525847	4.634581	1.960784	8.02139	6.951872	1.782531	3.743316	13.19073	0.713012	0.534759	2.139037	3.386809	6.951872	8.55615	7.130125	6.773619	P
SSA_1402	4.663212	6.217617	3.454231	7.081174	4.145078	5.354059	2.936097	8.462867	6.735751	1.554404	1.899827	13.12608	0.518135	0.172712	4.145078	6.390328	5.699482	5.008636	4.490501	7.944732	V
SSA_0271	4.560811	4.391892	3.547297	4.391892	6.418919	4.560811	3.209459	5.405405	4.391892	2.195946	5.067568	9.797297	2.027027	1.182432	5.912162	5.912162	6.925676	9.121622	5.743243	5.236486	G
SSA_0053	4.537815	4.87395	4.201681	4.033613	4.87395	4.033613	3.361345	5.378151	5.210084	2.689076	4.87395	10.7563	2.352941	1.008403	4.87395	5.714286	7.89916	7.226891	6.890756	5.210084	G

SSA_0726	4.507513	2.671119	3.839733	7.011686	8.013356	7.011686	1.168614	4.841402	3.839733	2.170284	3.338898	12.18698	2.003339	0.33389	5.676127	6.510851	7.846411	4.173623	5.008347	7.846411	V
SSA_1056	4.245283	3.773585	3.773585	6.761006	7.075472	4.245283	1.72956	6.918239	5.660377	2.987421	3.144654	12.42138	1.100629	1.257862	4.402516	5.345912	6.132075	8.647799	5.031447	5.345912	G
SSA_1442	11.11111	3.968254	1.587302	5.952381	6.349206	4.761905	2.777778	5.15873	3.571429	1.587302	2.380952	8.333333	0.793651	1.587302	1.587302	3.174603	9.126984	6.349206	7.539683	12.30159	E
SSA_1070	9.395973	6.040268	2.684564	3.691275	7.04698	4.697987	1.677852	6.040268	4.697987	4.026846	4.362416	9.395973	0.33557	0	3.020134	2.013423	8.389262	8.053691	5.704698	8.724832	J
SSA_1616	9.333333	5.666667	2.333333	2.666667	7	7.333333	3.333333	9.666667	4.666667	1.333333	4.333333	8.666667	0	0.333333	2.666667	3	6	8	6	7.666667	T
SSA_1671	8.832808	3.470032	6.309148	6.309148	7.570978	4.100946	1.892744	8.51735	4.416404	2.523659	2.208202	9.77918	0.315457	0	6.624606	4.100946	5.678233	5.362776	4.416404	7.570978	
SSA_1968	8.235294	2.941176	3.529412	1.764706	7.352941	4.117647	2.647059	5.588235	2.941176	2.058824	3.235294	6.764706	0.294118	0.882353	4.411765	5	9.411765	9.117647	7.352941	12.35294	EH
SSA_2349	8.163265	6.413994	3.498542	4.373178	4.664723	4.373178	2.915452	5.247813	5.539359	0.874636	4.081633	9.620991	0.58309	1.166181	5.247813	5.247813	7.580175	8.45481	3.206997	8.746356	MG
SSA_2009	8.139535	5.523256	4.069767	7.55814	4.651163	6.104651	1.744186	6.686047	5.813953	2.616279	2.325581	11.9186	0	0.581395	2.906977	6.686047	3.488372	6.395349	6.976744	5.813953	K
SSA_0883	7.526882	4.301075	2.419355	5.107527	4.032258	3.225806	2.419355	5.913978	4.569892	1.344086	5.107527	11.02151	0.268817	0.806452	1.075269	4.032258	11.82796	5.645161	6.989247	12.36559	G
SSA_1762	7.486631	1.871658	6.417112	6.951872	4.812834	4.812834	4.010695	13.90374	4.278075	1.069519	3.743316	16.31016	0.802139	0	4.545455	4.545455	5.614973	1.871658	2.406417	4.545455	R
SSA_0514	7.368421	2.631579	3.421053	7.368421	7.368421	6.578947	1.842105	7.105263	2.631579	2.894737	4.473684	8.947368	0	0.526316	3.421053	3.421053	6.315789	5.526316	5.789474	12.36842	C
SSA_0587	7.368421	3.421053	3.421053	6.052632	3.947368	5.263158	2.368421	4.473684	5.263158	3.684211	4.736842	11.57895	0.526316	0.263158	2.631579	5	7.368421	7.894737	4.473684	10.26316	R
SSA_1713	7.161125	4.603581	7.672634	5.626598	5.626598	5.370844	1.278772	9.462916	3.324808	1.278772	3.069054	7.928389	0.511509	0.255754	2.557545	4.092072	7.41688	5.626598	6.649616	10.48593	HE
SSA_0798	7.124682	2.798982	4.834606	7.888041	3.816794	4.580153	2.544529	9.669211	4.580153	1.272265	3.053435	14.75827	0.254453	0.763359	4.3257	7.888041	6.10687	2.035623	3.053435	8.651399	G
SSA_0386	7.070707	6.565657	4.545455	3.282828	6.313131	6.313131	3.282828	7.575758	5.050505	1.767677	2.777778	11.11111	0	0	1.010101	3.282828	6.060606	8.333333	7.828283	7.828283	E
SSA_0019	6.930693	2.475248	6.188119	9.90099	6.188119	6.435644	0.742574	4.950495	8.663366	0.247525	2.227723	5.445545	1.485149	0.49505	2.475248	0.49505	4.950495	8.663366	1.980198	19.05941	S
SSA_0012	6.557377	1.639344	6.088993	6.557377	8.430913	6.557377	1.639344	8.899297	6.088993	1.17096	2.810304	5.854801	0.702576	0	6.557377	3.981265	4.918033	6.323185	7.962529	7.259953	V
SSA_1836	6.481481	7.638889	2.546296	4.861111	6.481481	5.555556	1.388889	5.787037	6.25	1.62037	3.703704	12.5	0.462963	2.083333	2.777778	5.092593	5.324074	8.101852	4.62963	6.712963	L
SSA_1861	6.306306	3.603604	4.504505	7.657658	7.432432	4.504505	3.603604	2.927928	4.954955	1.801802	3.153153	9.684685	2.027027	0.225225	3.828829	4.954955	5.405405	7.882883	6.306306	9.234234	E
SSA_0247	6.263982	4.9217	4.026846	7.38255	8.277405	5.145414	3.131991	6.263982	4.250559	1.118568	2.013423	8.724832	1.342282	0.223714	4.697987	1.342282	7.829978	7.829978	7.606264	7.606264	
SSA_0632	6.19469	5.530973	3.318584	6.415929	5.088496	3.761062	3.761062	7.079646	4.20354	2.212389	4.20354	8.628319	0	0.221239	5.752212	3.539823	6.637168	8.40708	5.752212	9.292035	EH
SSA_0963	6.021505	2.150538	3.655914	6.021505	9.032258	6.666667	2.150538	7.741935	6.021505	3.010753	3.225806	8.817204	0.860215	0	3.870968	3.870968	5.376344	7.741935	6.666667	7.096774	G
SSA_1692	5.982906	3.205128	4.700855	3.632479	7.478632	4.273504	1.709402	6.196581	2.350427	4.273504	4.487179	6.196581	2.136752	0.42735	7.692308	5.34188	7.905983	7.264957	7.905983	6.837607	G
SSA_0356	5.932203	4.872881	5.932203	7.20339	4.237288	6.991525	1.694915	5.29661	3.813559	2.754237	5.508475	6.991525	1.90678	0.635593	5.084746	4.449153	5.720339	6.355932	7.838983	6.779661	E
SSA_0517	5.857741	3.974895	4.811715	6.694561	6.066946	5.439331	1.67364	6.694561	6.694561	4.1841	2.92887	11.92469	0	1.25523	3.138075	3.76569	4.60251	8.786611	5.648536	5.857741	T
SSA_0706	5.737705	8.606557	3.483607	4.918033	10.2459	5.327869	2.04918	6.352459	3.483607	1.229508	3.688525	7.786885	0	0.204918	1.844262	4.508197	9.016393	7.991803	6.557377	6.967213	LKJ
SSA_1024	5.737705	3.688525	5.942623	4.303279	6.352459	4.098361	1.639344	4.918033	3.688525	2.459016	2.868852	7.581967	3.07377	0.409836	5.532787	6.352459	7.377049	7.172131	9.836066	6.967213	G
SSA_0499	5.62249	3.212851	4.819277	5.220884	10.44177	6.024096	2.409639	5.823293	3.012048	1.807229	4.819277	6.2249	2.208835	0	4.618474	4.417671	5.421687	7.630522	9.638554	6.626506	E
SSA_1826	5.577689	2.988048	5.179283	5.776892	6.175299	6.374502	2.589641	7.768924	5.378486	1.195219	2.589641	7.569721	2.390438	0	3.585657	4.183267	8.366534	7.768924	4.98008	9.561753	C
SSA_1756	5.555556	3.769841	3.769841	6.944444	7.34127	6.746032	1.984127	7.34127	5.952381	1.785714	2.97619	5.753968	1.190476	0.198413	4.960317	3.968254	6.150794	7.738095	6.349206	9.52381	
SSA_0400	4.721754	3.878583	3.878583	5.227656	6.408094	7.588533	2.360877	6.408094	5.059022	1.349073	3.878583	11.46712	2.023609	0	4.721754	5.39629	7.082631	5.39629	5.564924	7.588533	V
SSA_1369	4.705882	3.361345	3.697479	7.394958	6.05042	6.554622	3.02521	6.386555	4.537815	1.848739	3.529412	12.60504	2.184874	0.168067	4.87395	4.87395	8.403361	4.705882	5.546218	5.546218	V
SSA_2051	4.682274	4.180602	3.511706	6.354515	5.518395	4.51505	2.842809	4.682274	4.849498	3.010033	2.173913	8.862876	1.170569	0.167224	5.685619	6.020067	5.183946	9.0301	7.525084	10.03344	E
SSA_1985	3.910615	1.815642	6.424581	17.4581	1.955307	10.05587	0.698324	4.748603	0.837989	0.558659	4.608939	7.402235	0.27933	0	1.117318	2.653631	9.497207	4.329609	12.84916	8.798883	
SSA_1306	13.12217	2.262443	3.61991	4.977376	4.072398	4.977376	2.262443	9.502262	4.524887	2.262443	1.809955	9.954751	0.904977	0.904977	1.357466	2.714932	6.334842	8.597285	7.692308	8.144796	P
SSA_0803	11.55378	2.788845	7.569721	13.94422	7.171315	11.55378	1.593625	5.976096	2.788845	0	3.187251	5.577689	0	0	3.585657	3.187251	4.780876	6.772908	2.390438	5.577689	S
SSA_1413	10.58394	1.824818	1.824818	7.29927	3.284672	3.284672	4.379562	6.20438	2.189781	1.459854	4.744526	12.40876	1.824818	0.364964	1.459854	6.569343	9.489051	4.014599	2.919708	13.86861	P
SSA_0591	10.32028	4.270463	4.982206	6.405694	5.338078	6.761566	1.067616	8.185053	3.202847	2.846975	2.846975	6.761566	1.067616	0.355872	4.982206	4.626335	8.185053	7.47331	4.626335	5.69395	S
SSA_2269	9.324759	4.180064	4.180064	7.395498	6.109325	8.360129	2.893891	10.28939	2.250804	2.893891	2.250804	7.073955	0.643087	0	3.858521	8.360129	6.752412	3.215434	2.572347	7.395498	S
SSA_1221	8.841463	2.743902	4.573171	5.792683	6.097561	3.963415	0.914634	7.926829	5.182927	1.829268	3.353659	7.621951	1.219512	0.304878	2.743902	3.963415	7.621951	6.097561	5.792683	13.41463	C
SSA_0061	8.405797	3.768116	2.898551	6.956522	6.376812	3.478261	1.15942	4.347826	4.927536	1.73913	4.057971	10.43478	1.73913	0.869565	2.898551	4.347826	5.507246	7.826087	7.826087	10.43478	G
SSA_1578	8.333333	3.16092	2.873563	8.908046	2.873563	6.609195	3.16092	10.34483	1.436782	0.574713	3.16092	14.08046	1.436782	1.724138	2.298851	7.183908	8.908046	1.436782	1.724138	9.770115	P
SSA_0425	8.309456	4.297994	4.297994	6.590258	6.590258	6.590258	2.292264	8.882521	4.011461	2.005731	2.578797	11.17479	0.573066	0.286533	4.297994	6.876791	5.157593	3.438395	4.584527	7.163324	M
SSA_1038	8.262108	1.709402	5.982906	5.982906	9.401709	6.837607	0.2849	4.558405	4.273504	0	0.854701	5.128205	1.139601	0.2849	3.133903	3.703704	11.39601	5.698006	7.977208	13.39031	R
SSA_0209	8.19209	4.237288	1.977401	6.497175	3.954802	6.214689	2.259887	6.497175	3.672316	2.824859	5.084746	7.627119	0.564972	0.847458	3.107345	4.237288	11.01695	5.649718	5.932203	9.60452	G
SSA_2076	7.988981	3.856749	5.785124	3.856749	6.336088	4.132231	3.305785	7.162534	2.754821	3.305785	4.958678	6.060606	3.305785	1.101928	3.305785	4.958678	6.887052	7.988981	6.61157	6.336088	R
SSA_1022	7.816712	6.469003	3.234501	4.851752	5.390836	2.695418	2.96496	5.121294	5.121294	2.156334	2.695418	12.66846	1.617251	0.808625	4.043127	4.312668	6.469003	8.894879	7.008086	5.660377	M
SSA_2025	7.671958	2.645503	5.291005	6.613757	7.671958	7.407407	2.116402	9.259259	4.761905	1.058201	3.174603	8.730159	0.529101	1.058201	1.851852	5.026455	7.671958	6.349206	4.232804	6.878307	S
SSA_0428	7.651715	4.221636	5.013193	5.804749	5.013193	2.638522	2.37467	5.804749	5.013193	3.166227	3.693931	10.81794	0.791557	0.527704	4.74934	5.540897	7.651715	2.902375	8.707124	7.915567	E
SSA_0758	7.304786	1.763224	3.778338	6.549118	6.297229	7.556675	3.526448	7.304786	4.282116	1.259446	2.770781	8.564232	1.007557	1.511335	1.763224	2.770781	8.060453	5.541562	6.801008	11.5869	E
SSA_0564	7.178218	5.19802	5.693069	5.445545	5.19802	1.980198	3.960396	8.168317	4.207921	2.722772	4.70297	9.405941	0.990099	0.990099	3.960396	3.712871	6.930693	5.445545	7.673267	6.435644	E
SSA_1058	7.178218	5.445545	2.970297	5.693069	6.930693	6.435644	3.465347	9.653465	4.70297	1.237624	4.950495	6.683168	0	0.49505	3.465347	3.465347	5.940594	9.158416	4.70297	7.425743	H
SSA_0637	7.125307	4.668305	3.194103	3.685504	5.651106	4.422604	2.457002	4.422604	4.422604	2.702703	3.685504	9.090909	0.2457	0.737101	3.685504	3.439803	9.82801	7.616708	5.896806	13.02211	E
SSA_1140	6.921241	2.863962	3.579952	7.637232	5.48926	6.443914	2.147971	6.921241	4.77327	1.670644	4.295943	10.26253	0.71599	0.238663	2.147971	1.909308	9.069212	7.159905	4.77327	10.97852	C
SSA_1519	6.839623	3.301887	4.481132	9.433962	4.716981	5.660377	1.886792	12.26415	3.301887	1.179245	2.122642	14.85849	1.179245	0.707547	3.773585	9.90566	3.773585	2.358491	3.066038	5.188679	R
SSA_0488	6.712963	3.703704	2.546296	6.712963	4.398148	4.62963	3.009259	4.861111	4.398148	2.314815	4.166667	8.564815	0.462963	1.157407	3.935185	5.787037	9.953704	6.712963	4.861111	11.11111	H
SSA_0864	6.69746	6.004619	4.387991	6.235566	4.387991	5.311778	2.771363	6.235566	5.773672	4.387991	3.926097	8.775982	0.461894	0.461894	5.542725	6.69746	4.618938	5.542725	6.928406	4.849885	R
SSA_1574	6.590909	5	2.045455	6.363636	7.045455	5.681818	2.045455	10.22727	2.727273	1.818182	3.863636	9.772727	0.454545	0.227273	5.227273	4.318182	5	8.181818	5.909091	7.5	M
SSA_1182	6.531532	4.72973	4.054054	4.72973	6.306306	4.72973	3.603604	5.630631	3.378378	2.027027	5.18018	8.333333	0.225225	0.45045	4.72973	4.054054	7.882883	8.333333	5.18018	9.90991	J
SSA_0552	6.236559	4.301075	2.365591	8.602151	7.956989	4.086022	2.580645	6.666667	4.731183	3.225806	3.655914	7.741935	0.645161	0	2.580645	5.806452	5.806452	8.172043	7.096774	7.741935	S
SSA_0950	6.196581	2.991453	5.769231	5.982906	8.547009	7.264957	2.564103	5.769231	5.128205	2.136752	2.777778	6.196581	0.854701	0	4.91453	3.205128	6.410256	7.692308	6.623932	8.974359	
SSA_1282	6.196581	2.564103	3.418803	5.128205	6.410256	5.555556	1.923077	4.91453	1.923077	3.205128	4.273504	8.119658	0.42735	0.42735	3.846154	5.982906	9.401709	7.692308	8.547009	10.04274	E

SSA_2309	6.170213	3.617021	6.808511	8.510638	7.87234	4.042553	2.553191	5.531915	4.042553	1.276596	2.978723	9.787234	0.851064	0.212766	3.404255	5.106383	5.744681	6.808511	6.808511	7.87234	NU
SSA_0907	5.282332	5.64663	4.371585	6.010929	8.014572	7.468124	1.639344	6.739526	6.557377	2.185792	3.278689	11.11111	0.182149	0	2.185792	4.553734	4.735883	9.289617	4.553734	6.193078	K
SSA_1168	5.150977	3.552398	8.348135	5.861456	8.348135	4.618117	1.598579	7.815275	3.552398	1.776199	2.841918	9.769094	1.953819	0.71048	4.973357	6.216696	4.262877	6.749556	6.394316	5.506217	
SSA_1261	13.33333	3.111111	2.666667	4.444444	7.555556	4	2.666667	5.777778	4.888889	1.333333	1.777778	7.555556	0.444444	0	2.222222	4.888889	10.22222	8.888889	6.666667	7.555556	G
SSA_2174	12.93103	3.448276	5.172414	3.448276	6.034483	4.310345	2.586207	10.34483	3.017241	0.862069	3.87931	6.034483	0.862069	0	0.431034	1.293103	10.34483	6.896552	5.603448	12.5	E
SSA_0075	10.20408	4.081633	5.102041	5.102041	4.081633	5.782313	2.040816	9.863946	2.040816	1.360544	4.421769	11.90476	3.741497	0.680272	3.401361	9.863946	5.782313	1.70068	2.040816	6.802721	G
SSA_0457	10.03344	4.682274	5.016722	3.67893	4.013378	7.692308	1.672241	5.685619	2.341137	2.341137	5.016722	6.020067	0.668896	1.003344	4.013378	5.351171	11.37124	6.020067	4.347826	9.0301	KG
SSA_1015	10.03344	5.016722	4.013378	3.67893	5.016722	6.688963	4.013378	7.692308	1.337793	1.672241	3.67893	14.38127	1.672241	0.668896	5.351171	10.03344	2.675585	4.013378	3.67893	4.682274	H
SSA_1207	9.230769	4.615385	3.076923	5.538462	6.461538	4.615385	2.153846	7.692308	3.076923	0.923077	4.923077	8.615385	0.307692	1.230769	2.769231	4	7.384615	7.384615	6.153846	9.846154	C
SSA_0215	9.146341	3.04878	4.573171	6.402439	9.146341	4.878049	3.04878	9.45122	7.621951	0.914634	3.658537	8.536585	0.609756	0	3.353659	2.743902	5.487805	4.573171	6.707317	6.097561	G
SSA_0049	9.118541	1.823708	2.12766	5.775076	6.68693	4.559271	3.951368	7.598784	2.735562	2.12766	3.343465	8.206687	0.607903	0.303951	2.431611	3.039514	10.94225	8.206687	6.079027	10.33435	G
SSA_1918	8.982036	3.293413	5.389222	5.08982	6.886228	3.892216	3.293413	7.185629	3.293413	1.497006	5.08982	7.48503	0.598802	0	0.898204	3.293413	6.586826	6.586826	7.48503	13.17365	G
SSA_2021	8.72093	2.906977	4.069767	6.104651	4.651163	6.976744	3.488372	7.848837	2.325581	1.744186	4.069767	5.813953	1.453488	2.325581	5.523256	2.034884	7.55814	7.848837	6.104651	8.430233	S
SSA_2298	8.72093	4.360465	4.360465	6.686047	7.267442	6.104651	2.906977	7.55814	2.906977	1.162791	4.360465	8.139535	0	0	4.069767	2.906977	9.593023	7.55814	4.651163	6.686047	T
SSA_0364	8.695652	2.608696	5.217391	9.565217	3.478261	5.507246	2.608696	9.565217	3.188406	0.57971	3.768116	13.91304	0.869565	1.449275	2.028986	4.057971	6.956522	2.318841	2.608696	11.01449	E
SSA_1010	8.645533	4.034582	3.746398	5.18732	5.18732	4.610951	2.881844	6.340058	2.305476	3.746398	5.763689	6.628242	1.152738	0.576369	3.170029	4.610951	7.78098	6.340058	7.492795	9.798271	M
SSA_0376	8.450704	2.253521	5.915493	4.788732	7.605634	5.633803	2.253521	10.14085	7.605634	3.380282	2.253521	10.42254	0	0	1.971831	1.971831	5.633803	5.352113	5.915493	8.450704	P
SSA_0776	7.894737	3.421053	7.105263	9.210526	5.526316	2.894737	2.631579	8.947368	2.631579	1.842105	3.157895	6.315789	1.578947	0.526316	4.473684	2.631579	8.947368	6.578947	6.842105	6.842105	G
SSA_1736	7.731959	4.896907	5.154639	3.865979	5.670103	4.123711	1.030928	5.412371	4.639175	2.835052	4.381443	10.05155	1.804124	1.030928	4.123711	5.154639	5.412371	9.020619	6.185567	7.474227	E
SSA_1432	7.281553	5.339806	4.368932	6.553398	7.76699	6.067961	2.669903	6.31068	4.126214	1.941748	2.427184	11.8932	0.485437	0.242718	2.427184	3.15534	5.097087	9.708738	5.825243	6.31068	R
SSA_1909	7.281553	2.669903	6.31068	10.67961	5.339806	9.466019	3.640777	6.067961	5.097087	0.728155	3.640777	8.252427	0.485437	0	4.854369	1.213592	7.038835	5.582524	5.339806	6.31068	K
SSA_0614	7.263923	2.179177	4.842615	10.65375	5.326877	4.600484	2.663438	8.232446	3.1477	0.968523	3.1477	17.91768	1.210654	0.484262	2.421308	8.716707	3.389831	1.937046	2.663438	8.232446	
SSA_1124	7.246377	3.140097	6.038647	5.072464	5.555556	5.555556	2.173913	5.555556	3.381643	2.415459	6.038647	6.763285	1.449275	0.483092	4.347826	4.589372	9.178744	6.763285	7.004831	7.246377	S
SSA_2333	7.246377	4.347826	3.623188	5.31401	5.797101	3.623188	4.589372	5.797101	1.449275	2.415459	3.864734	15.45894	2.898551	0.241546	5.072464	10.62802	6.763285	3.140097	3.381643	4.347826	M
SSA_0436	7.125891	3.800475	2.375297	4.513064	5.938242	6.650831	3.325416	5.225653	2.850356	2.612827	3.562945	10.68884	0.23753	1.425178	3.562945	3.800475	8.07601	7.83848	6.650831	9.738717	Q
SSA_2246	6.78733	4.072398	4.072398	8.371041	5.656109	7.013575	1.58371	5.656109	4.298643	0.678733	3.61991	11.76471	0	0.226244	1.809955	4.977376	8.597285	7.239819	4.298643	9.276018	R
SSA_0741	6.741573	3.595506	2.022472	5.842697	4.94382	5.617978	1.797753	3.595506	4.044944	1.348315	5.168539	11.68539	0.674157	0.898876	4.044944	4.719101	8.764045	8.089888	6.516854	9.88764	E
SSA_0589	6.521739	4.782609	4.130435	6.73913	4.565217	4.782609	2.391304	6.304348	4.130435	2.391304	4.347826	9.782609	0.652174	0	5	3.695652	7.826087	8.26087	6.521739	7.173913	E
SSA_1006	6.237006	3.742204	5.405405	5.821206	6.860707	4.365904	1.663202	5.821206	4.365904	1.871102	2.702703	9.56341	1.247401	0.2079	6.029106	5.821206	4.989605	8.316008	8.731809	6.237006	G
SSA_0817	6.185567	5.154639	2.474227	8.865979	5.154639	7.835052	1.85567	5.773196	5.56701	2.268041	3.092784	11.75258	1.237113	0.824742	2.680412	5.979381	5.360825	5.154639	5.360825	7.42268	KT
SSA_0208	6.072874	4.251012	5.870445	4.65587	7.08502	5.060729	3.036437	8.299595	3.441296	2.834008	4.65587	9.919028	0.202429	0.202429	4.453441	2.631579	6.275304	6.882591	6.275304	7.894737	S
SSA_1239	5.859375	3.710938	3.710938	6.445313	6.25	4.101563	2.929688	6.25	4.101563	2.148438	3.125	14.84375	2.148438	0.390625	5.273438	10.15625	5.078125	4.882813	2.148438	6.445313	M
SSA_2231	5.825243	3.495146	3.300971	9.320388	4.466019	5.048544	1.359223	4.854369	5.436893	0.970874	3.300971	17.28155	1.747573	0.194175	5.825243	7.184466	6.796117	3.300971	2.912621	7.378641	M
SSA_0971	5.154639	4.123711	6.357388	5.32646	8.247423	6.185567	2.233677	4.982818	3.264605	1.718213	3.264605	8.934708	0.687285	0	3.092784	5.670103	6.701031	6.357388	8.934708	8.762887	
SSA_0325	5.136986	3.938356	3.938356	6.506849	5.650685	5.136986	3.082192	6.849315	4.623288	1.027397	2.568493	15.92466	1.369863	0.171233	5.308219	7.705479	4.280822	4.109589	5.479452	7.191781	C
SSA_1371	5.059022	3.035413	3.372681	8.263069	7.082631	8.431703	2.866779	5.059022	3.372681	1.686341	2.866779	11.12985	1.854975	0.168634	4.721754	6.23946	7.757167	4.721754	4.890388	7.419899	V
SSA_0751	5	3.5	3.833333	6.166667	6.333333	5.5	1.833333	4	5	3	1.666667	10	0.666667	0	7.166667	5.333333	4.333333	10.5	6.333333	9.833333	E
SSA_0796	4.754358	5.229794	4.912837	6.81458	6.339144	5.071315	2.377179	5.546751	6.497623	0.792393	1.901743	12.99525	0.633914	0	3.011094	3.328051	5.546751	10.61807	6.497623	7.131537	R
SSA_0233	10.40268	1.677852	2.013423	8.389262	3.355705	6.040268	3.355705	11.4094	4.697987	0.33557	3.355705	15.77181	0	0.671141	2.684564	7.04698	7.718121	2.684564	1.342282	7.04698	R
SSA_1697	10.03236	1.618123	6.472492	7.443366	5.825243	5.177994	1.618123	7.119741	3.236246	1.941748	3.559871	11.00324	0.647249	0.647249	2.265372	2.265372	7.76699	7.76699	5.501618	8.090615	G
SSA_0739	9.84127	1.904762	5.714286	5.079365	7.619048	3.492063	0.952381	6.666667	4.761905	1.269841	4.444444	9.52381	0.634921	0	1.269841	3.174603	8.571429	8.253968	5.396825	11.42857	E
SSA_1176	9.627329	4.347826	3.10559	6.21118	5.900621	4.968944	4.968944	7.142857	2.173913	1.242236	5.590062	5.590062	0.310559	0.931677	2.173913	3.416149	9.006211	8.074534	5.900621	9.31677	C
SSA_0921	8.985507	5.217391	2.608696	4.347826	4.347826	5.217391	2.608696	6.956522	3.768116	2.318841	2.898551	6.086957	0.869565	2.318841	2.608696	2.608696	11.30435	5.217391	8.115942	11.5942	ER
SSA_0040	8.539945	3.030303	4.132231	6.336088	4.958678	4.132231	2.203857	7.988981	3.856749	3.305785	3.856749	8.539945	0	1.37741	2.754821	3.305785	6.61157	6.61157	7.713499	10.7438	F
SSA_0576	8.423913	4.347826	2.98913	4.347826	8.423913	5.978261	0.543478	7.336957	3.26087	2.98913	4.619565	9.782609	0.815217	1.358696	2.717391	4.076087	8.695652	6.25	7.608696	5.434783	R
SSA_0148	8.244681	5.319149	4.787234	4.787234	6.648936	5.319149	2.925532	6.914894	2.12766	1.861702	3.989362	7.978723	0	0.531915	3.457447	4.521277	7.712766	7.978723	6.382979	8.510638	G
SSA_0178	8.051948	4.675325	3.376623	3.376623	6.753247	8.051948	3.636364	5.714286	4.935065	3.116883	4.675325	9.090909	0.25974	0	2.597403	3.376623	4.155844	8.051948	7.012987	9.090909	M
SSA_2060	7.969152	3.598972	1.799486	11.82519	3.856041	5.398458	3.341902	8.48329	3.856041	0.257069	2.570694	16.96658	1.028278	0.514139	2.570694	6.683805	7.969152	3.598972	0.771208	6.940874	G
SSA_0631	7.888041	4.071247	1.78117	4.834606	6.10687	5.089059	2.544529	5.089059	3.307888	2.798982	3.307888	7.888041	0.254453	0.508906	4.580153	3.562341	9.923664	7.888041	6.615776	11.95929	E
SSA_1101	7.711443	3.233831	4.726368	8.955224	8.457711	5.472637	2.736318	8.955224	5.721393	1.243781	2.736318	10.199	0.995025	0.248756	3.9801	1.243781	5.223881	5.472637	5.970149	6.716418	V
SSA_0895	7.654321	2.469136	3.950617	7.654321	7.901235	4.444444	2.469136	10.8642	4.938272	0.740741	3.209877	10.37037	0.246914	0.493827	3.209877	4.691358	8.148148	5.925926	3.950617	6.666667	V
SSA_1093	7.579462	4.889976	4.889976	3.91198	6.112469	3.667482	2.200489	10.02445	5.623472	0.977995	3.178484	8.801956	0	0.977995	2.200489	3.178484	6.356968	10.51345	6.601467	8.312958	O
SSA_2063	7.506053	3.389831	5.811138	5.569007	5.811138	4.842615	2.421308	5.084746	2.905569	2.421308	3.874092	7.990315	1.694915	0.484262	2.421308	3.874092	6.779661	8.474576	6.2954	12.34867	E
SSA_2188	7.416268	3.110048	6.698565	6.45933	7.894737	6.698565	2.870813	6.698565	9.808612	0.239234	1.913876	9.808612	0.239234	0	1.196172	2.631579	3.110048	9.090909	4.784689	9.330144	P
SSA_0378	7.328605	3.782506	4.018913	5.673759	3.546099	6.146572	2.364066	5.200946	4.018913	2.600473	3.546099	11.82033	0.945626	1.41844	3.782506	2.836879	6.619385	7.801418	6.855792	9.692671	FR
SSA_0855	7.276995	3.286385	3.755869	6.103286	4.225352	8.450704	0.938967	6.807512	2.816901	3.286385	4.694836	9.859155	0.234742	0	2.816901	5.399061	7.042254	6.338028	6.103286	10.56338	E
SSA_0218	7.259953	3.044496	3.044496	9.836066	6.557377	4.449649	4.215457	4.683841	5.854801	2.107728	3.747073	9.601874	1.873536	0.702576	4.449649	3.512881	4.683841	6.088993	7.259953	7.025761	G
SSA_1761	6.950673	3.587444	5.605381	5.381166	4.932735	5.829596	3.811659	8.96861	2.914798	0.44843	2.690583	12.55605	0.672646	0.224215	2.466368	4.26009	5.156951	10.08969	7.399103	6.053812	R
SSA_0307	6.919643	4.241071	5.357143	4.6875	5.580357	5.133929	3.571429	5.803571	1.339286	2.678571	5.133929	8.928571	1.116071	0.669643	4.017857	5.133929	6.696429	8.258929	6.696429	8.035714	E
SSA_1143	6.904232	2.672606	3.340757	6.013363	3.563474	4.899777	3.11804	9.576837	4.454343	0.890869	2.895323	16.9265	1.781737	0.890869	2.227171	6.458797	8.017817	2.895323	1.781737	10.69042	V
SSA_0519	6.798246	2.850877	5.482456	4.605263	6.359649	6.798246	4.166667	6.140351	3.070175	2.631579	2.850877	8.552632	1.096491	1.096491	3.070175	3.947368	7.894737	7.675439	6.359649	8.552632	E
SSA_1269	6.73913	3.695652	5.434783	8.478261	6.086957	5.217391	3.043478	6.73913	3.478261	2.173913	3.478261	6.521739	1.086957	1.086957	4.347826	4.782609	7.608696	9.130435	5.217391	5.652174	O
SSA_1246	6.526316	4	5.684211	5.263158	12	5.052632	1.684211	7.578947	4.631579	1.684211	2.105263	8.842105	0	0.631579	5.684211	4.210526	4.210526	8.421053	6.947368	4.842105	

SSA_1634	6.378601	3.703704	5.967078	8.024691	6.995885	12.34568	1.646091	5.349794	3.497942	1.028807	3.497942	9.053498	0.617284	0	4.938272	3.292181	7.407407	3.909465	5.144033	7.201646	M
SSA_1392	5.709024	2.578269	5.709024	9.576427	8.287293	3.867403	2.578269	6.998158	6.26151	1.104972	1.289134	7.918969	0.552486	0.184162	5.524862	3.130755	7.18232	8.103131	6.445672	6.998158	R
SSA_0679	5.615942	5.797101	3.804348	7.608696	5.615942	4.166667	2.898551	6.884058	5.797101	1.992754	1.086957	11.77536	0.181159	0	2.355072	3.804348	5.978261	10.50725	6.521739	7.608696	L
SSA_1403	5.344828	5.689655	3.448276	6.724138	4.310345	5.517241	2.068966	8.103448	5.172414	2.068966	2.068966	14.13793	1.034483	0.172414	3.965517	4.482759	7.241379	6.724138	4.137931	7.586207	V
SSA_0555	5.317324	4.459691	6.174957	7.375643	7.03259	5.317324	1.715266	6.174957	4.974271	1.543739	3.430532	6.689537	0.857633	0.343053	6.003431	3.259005	4.974271	8.747856	6.346484	9.262436	
SSA_1144	4.74732	2.603369	4.287902	4.287902	6.278714	4.287902	2.45023	8.422665	4.594181	2.756508	5.972435	7.350689	1.990812	1.225115	6.584992	5.972435	5.972435	8.575804	6.125574	5.513017	G
SSA_2299	10.81081	4.72973	2.027027	7.094595	2.364865	3.378378	2.702703	6.756757	2.027027	0.675676	2.702703	16.55405	2.027027	1.013514	5.743243	10.81081	6.418919	2.364865	4.054054	5.743243	
SSA_0908	9.552239	0.298507	5.074627	8.059701	10.74627	6.567164	2.38806	7.462687	3.283582	0.597015	3.880597	7.462687	0.298507	0	2.686567	1.19403	8.358209	5.373134	6.567164	10.14925	R
SSA_0318	9.52381	2.97619	3.571429	6.25	5.059524	3.571429	2.678571	5.357143	3.27381	2.97619	3.571429	10.41667	0.595238	0.892857	2.678571	3.27381	7.142857	8.630952	4.761905	12.79762	O
SSA_0628	8.839779	6.353591	6.629834	4.972376	6.629834	4.696133	3.038674	6.629834	5.248619	1.933702	2.762431	8.287293	0.828729	1.657459	3.59116	3.59116	5.248619	7.734807	5.248619	6.077348	R
SSA_2040	8.510638	4.787234	4.521277	4.521277	7.978723	5.585106	2.925532	7.180851	2.393617	2.12766	3.723404	7.978723	0	0.531915	3.457447	4.521277	7.712766	7.712766	6.648936	7.180851	G
SSA_1954	7.804878	3.902439	3.658537	3.658537	4.146341	4.878049	2.439024	5.365854	5.121951	3.170732	3.658537	9.02439	0.97561	0.731707	3.902439	4.878049	6.585366	7.560976	6.097561	12.43902	E
SSA_1298	7.655502	0.478469	5.023923	9.569378	10.04785	5.502392	1.435407	3.110048	5.263158	0.478469	2.631579	7.177033	1.196172	0.239234	3.827751	3.827751	7.655502	6.698565	6.698565	11.48325	G
SSA_2070	7.655502	2.870813	5.023923	5.263158	6.45933	6.698565	2.631579	10.76555	2.631579	1.435407	3.349282	9.569378	1.435407	0	2.392344	5.502392	8.851675	4.30622	5.263158	7.894737	M
SSA_1235	7.582938	2.132701	3.554502	5.450237	6.161137	6.161137	2.606635	6.872038	3.554502	4.50237	3.554502	9.478673	0	0.473934	0.7109	4.739336	8.293839	6.635071	6.398104	11.13744	F
SSA_0330	7.494145	4.449649	3.512881	6.323185	6.557377	4.449649	1.639344	8.665105	3.981265	2.576112	2.107728	13.11475	0.702576	1.17096	5.386417	5.152225	8.430913	4.449649	4.449649	5.386417	S
SSA_1464	7.494145	5.386417	2.576112	6.088993	4.918033	6.323185	3.512881	9.601874	4.918033	1.639344	3.747073	8.899297	0.234192	0	0.468384	2.576112	10.07026	8.196721	5.620609	7.728337	E
SSA_1446	7.44186	4.418605	2.55814	5.813953	4.186047	4.418605	2.093023	8.837209	5.581395	1.627907	4.186047	7.674419	0	0	3.953488	2.55814	6.976744	9.534884	5.813953	12.32558	E
SSA_2311	7.207207	3.378378	6.531532	7.882883	9.459459	6.531532	2.027027	8.108108	4.72973	0	2.702703	7.882883	1.351351	0	3.603604	3.378378	8.558559	4.504505	6.531532	5.630631	U
SSA_1307	7.126949	3.563474	3.11804	6.458797	4.008909	9.131403	3.11804	6.904232	3.340757	2.004454	2.227171	16.70379	0.890869	0.890869	2.004454	8.685969	9.576837	1.336303	2.672606	6.23608	P
SSA_1564	7.126949	5.790646	5.122494	6.681514	5.122494	6.904232	2.227171	10.02227	4.008909	1.113586	2.449889	10.46771	0.668151	0	2.449889	3.563474	4.899777	7.7951	9.131403	4.454343	T
SSA_0747	7.095344	2.882483	6.651885	6.208426	9.312639	6.430155	3.104213	5.764967	2.882483	1.108647	3.991131	7.982262	0.886918	0	5.543237	2.882483	5.764967	6.651885	5.986696	8.86918	M
SSA_0897	7.002188	5.47046	3.501094	8.971554	7.439825	4.376368	2.188184	6.564551	5.689278	1.31291	2.188184	12.69147	0.218818	0.437637	4.376368	3.938731	4.814004	8.971554	4.376368	5.47046	T
SSA_0092	6.896552	6.25	3.663793	5.172414	4.525862	5.387931	2.801724	7.112069	4.525862	1.508621	4.956897	9.913793	0.215517	0.215517	3.663793	3.448276	7.758621	8.836207	5.818966	7.327586	C
SSA_1952	6.808511	2.978723	4.255319	7.87234	7.021277	5.531915	3.404255	6.170213	2.978723	2.12766	4.468085	6.382979	1.276596	0.425532	4.893617	4.042553	6.382979	9.361702	5.531915	8.085106	O
SSA_1632	6.680585	2.087683	6.05428	7.306889	6.889353	12.73486	1.25261	6.263048	4.384134	0.835073	2.713987	7.933194	0.208768	0	5.219207	3.966597	8.350731	3.340292	5.845511	7.933194	M
SSA_1633	6.680585	2.922756	6.680585	10.43841	6.471816	12.5261	1.461378	6.05428	3.340292	0.626305	1.878914	8.350731	0.417537	0	4.592902	3.340292	7.515658	3.340292	5.219207	8.141962	M
SSA_1009	6.490872	3.853955	3.853955	5.679513	5.882353	4.868154	1.825558	6.693712	5.679513	3.042596	4.868154	8.72211	1.014199	0.811359	3.448276	4.462475	5.679513	9.127789	6.085193	7.910751	G
SSA_0568	5.536332	6.228374	2.941176	4.67128	5.882353	3.633218	2.595156	4.49827	5.536332	2.595156	3.287197	12.62976	0.692042	0.692042	2.768166	5.536332	6.747405	10.0346	6.401384	7.093426	J
SSA_0928	5.442177	4.761905	2.891156	6.802721	3.911565	5.782313	3.061224	9.52381	3.061224	1.870748	2.891156	12.2449	1.70068	0.680272	3.571429	4.591837	7.653061	5.782313	5.442177	8.333333	V
SSA_1087	5.144695	4.823151	3.376206	6.752412	7.395498	5.787781	1.286174	6.430868	6.109325	1.286174	1.607717	13.66559	0.643087	0.160772	3.215434	4.340836	6.270096	9.646302	7.55627	4.501608	R
SSA_2186	4.260985	3.861518	4.527297	6.524634	4.527297	4.793609	2.396804	5.992011	5.326232	3.195739	4.394141	12.38349	0.932091	0.932091	4.394141	3.861518	5.459387	7.723036	6.258322	8.255659	H
SSA_1553	10.96346	2.990033	2.990033	8.9701	3.986711	3.654485	2.325581	8.637874	2.657807	0.996678	4.651163	13.95349	0.996678	1.328904	2.657807	10.63123	5.647841	2.657807	2.325581	6.976744	R
SSA_1727	10.41009	2.523659	4.100946	6.940063	3.785489	6.624606	2.839117	12.6183	1.892744	0.315457	2.208202	13.56467	0.946372	0	2.523659	5.047319	11.04101	2.523659	1.26183	8.832808	E
SSA_1041	10.37736	1.572327	3.459119	7.861635	3.773585	5.031447	2.830189	8.805031	2.201258	1.572327	3.773585	12.26415	0.943396	0.314465	3.144654	7.54717	10.06289	1.257862	1.886792	11.32075	R
SSA_1576	9.88024	5.389222	5.08982	8.383234	8.083832	6.586826	2.095808	6.886228	1.796407	1.497006	2.095808	8.083832	0	0	3.892216	2.694611	5.988024	6.586826	7.784431	7.185629	K
SSA_0298	9.620991	3.498542	3.498542	6.413994	3.790087	7.580175	3.206997	9.329446	2.915452	0	4.664723	14.28571	1.166181	0.58309	3.790087	7.580175	6.413994	2.623907	2.040816	6.997085	R
SSA_0471	8.991826	4.904632	2.724796	5.99455	5.99455	5.722071	1.362398	6.811989	4.087193	0.27248	3.814714	10.35422	0.544959	1.089918	2.452316	2.997275	7.084469	7.356948	5.722071	11.71662	H
SSA_0005	8.894879	5.121294	4.043127	3.773585	7.54717	5.660377	1.886792	6.738544	2.156334	1.078167	2.96496	7.54717	0.269542	0	2.425876	4.851752	6.738544	11.32075	6.738544	10.24259	J
SSA_1048	8.571429	6.233766	5.194805	3.636364	4.935065	4.155844	2.597403	8.831169	3.636364	2.077922	3.376623	9.090909	0.519481	0	2.077922	5.194805	6.233766	12.72727	6.493506	4.415584	E
SSA_0748	8.48329	1.285347	2.570694	7.197943	5.398458	3.856041	4.884319	7.455013	4.113111	2.056555	2.313625	16.96658	1.028278	0.771208	5.141388	6.683805	7.712082	1.285347	2.827763	7.969152	G
SSA_1575	8.418367	5.612245	2.55102	5.867347	6.632653	3.061224	2.55102	6.122449	3.316327	2.295918	3.571429	9.438776	1.020408	1.27551	5.612245	7.142857	7.142857	6.377551	5.357143	6.632653	M
SSA_1383	8.396947	3.562341	4.071247	5.343511	5.089059	9.414758	2.798982	5.597964	4.071247	0.254453	4.834606	7.888041	0.508906	0	4.580153	4.071247	6.615776	8.142494	3.816794	10.94148	E
SSA_0192	8.312343	4.785894	3.778338	6.549118	6.549118	5.541562	2.267003	9.06801	2.267003	2.267003	3.778338	7.808564	0.503778	0.503778	3.02267	3.02267	7.808564	7.304786	7.052897	7.808564	C
SSA_1507	8.229426	3.491272	6.483791	7.23192	8.229426	4.738155	2.244389	6.982544	5.236908	1.246883	2.493766	7.98005	0.498753	0.74813	3.241895	4.239401	6.733167	6.982544	7.23192	5.735661	GM
SSA_0737	8.06846	6.601467	4.889976	3.178484	6.845966	4.645477	2.200489	6.601467	1.95599	2.200489	3.91198	10.26895	0.244499	0.488998	3.667482	4.156479	6.845966	11.24694	6.112469	5.867971	E
SSA_0875	8.04878	2.926829	2.926829	6.829268	3.902439	5.609756	3.170732	10	4.390244	1.463415	3.902439	16.09756	1.463415	0	4.146341	7.317073	8.292683	2.195122	2.682927	4.634146	D
SSA_1967	7.932692	4.086538	4.807692	4.567308	6.971154	4.567308	1.923077	8.894231	3.846154	1.682692	3.605769	6.490385	0	0.961538	4.326923	4.086538	8.413462	6.971154	6.490385	9.375	E
SSA_1951	7.875895	2.147971	6.205251	8.114558	8.114558	9.307876	2.625298	5.727924	2.625298	0.477327	2.386635	7.637232	0.71599	0.238663	4.057279	3.818616	5.48926	6.682578	5.966587	9.785203	M
SSA_0231	7.692308	3.962704	2.797203	5.594406	6.993007	6.060606	1.398601	7.925408	2.331002	1.398601	2.797203	15.15152	0.699301	0.932401	4.895105	5.594406	8.158508	4.662005	4.895105	6.060606	
SSA_1541	7.692308	7.459207	3.496503	5.361305	4.662005	4.662005	4.195804	5.128205	2.797203	2.331002	3.263403	6.526807	0.932401	1.165501	3.962704	3.962704	7.226107	9.79021	4.895105	10.48951	O
SSA_1148	7.586207	0.91954	3.678161	5.977011	4.597701	5.057471	4.367816	9.195402	4.137931	0.689655	5.057471	11.95402	1.83908	0.91954	3.908046	8.275862	7.586207	2.988506	2.068966	9.195402	G
SSA_0074	7.366071	1.339286	6.026786	7.142857	8.928571	5.803571	3.125	5.580357	4.464286	1.785714	4.464286	8.035714	1.5625	0.223214	3.794643	4.241071	6.696429	6.026786	6.696429	6.696429	G
SSA_0463	7.300885	3.982301	2.433628	4.867257	5.973451	4.867257	2.433628	4.20354	3.539823	2.876106	4.20354	9.955752	0.884956	0.663717	4.867257	4.867257	8.40708	6.19469	7.964602	9.513274	H
SSA_0273	7.006369	3.397028	4.246285	8.492569	5.944798	3.609342	2.335456	2.547771	7.855626	0.424628	5.732484	7.218684	2.760085	0.212314	1.910828	2.123142	8.067941	6.794055	5.307856	14.01274	I
SSA_2310	6.666667	4.848485	9.292929	6.464646	7.070707	7.070707	1.616162	7.878788	4.040404	1.616162	2.424242	7.878788	0.606061	0	4.040404	4.242424	7.878788	4.242424	5.252525	6.868687	NU
SSA_1636	6.432749	5.263158	4.288499	4.288499	6.432749	3.898635	2.923977	5.45809	5.263158	1.949318	2.923977	11.50097	0.974659	0.584795	3.703704	3.898635	6.62768	9.356725	6.822612	7.407407	R
SSA_1961	6.333973	3.262956	4.414587	5.758157	6.142035	8.06142	3.262956	9.980806	5.566219	0.191939	2.495202	7.485605	0.767754	0	4.03071	4.798464	7.293666	5.566219	4.798464	9.788868	E
SSA_1852	6.145251	8.007449	3.538175	6.517691	7.635009	3.910615	2.048417	8.938547	3.724395	2.048417	2.048417	8.379888	0	0	1.862197	1.675978	4.655493	11.91806	6.145251	10.80074	R
SSA_1751	5.861456	3.552398	4.973357	4.795737	5.683837	5.150977	3.374778	5.328597	4.440497	2.131439	4.795737	8.170515	2.309059	0.53286	5.150977	5.683837	6.927176	6.749556	8.703375	5.683837	G
SSA_0775	5.188679	4.08805	5.660377	5.031447	6.132075	4.402516	3.144654	5.345912	3.616352	2.987421	3.773585	8.333333	3.616352	0.471698	5.345912	5.974843	7.54717	8.176101	6.446541	4.716981	G
SSA_1274	5.116279	2.945736	12.71318	4.806202	6.356589	5.116279	0.775194	2.635659	2.170543	0.930233	8.992248	4.496124	2.325581	0	12.09302	2.015504	6.511628	7.286822	6.511628	6.20155	M
SSA_2257	5.100464	5.564142	3.554869	6.955178	6.646059	4.173107	2.318393	7.882535	5.100464	2.318393	4.173107	9.891808	0.15456	0.618238	3.554869	3.709428	5.100464	9.891808	5.255023	8.037094	L

SSA_1476	4.514364	3.009576	6.566347	9.439124	5.745554	6.566347	2.188782	5.608755	4.240766	1.367989	3.556772	10.53352	0.957592	0	6.976744	6.29275	6.019152	4.377565	5.471956	6.566347	M
SSA_1251	4.313725	4.96732	4.836601	6.405229	6.797386	3.006536	1.437908	6.013072	4.183007	1.568627	1.960784	10.98039	0.784314	0.784314	4.836601	6.27451	7.058824	8.888889	6.27451	8.627451	R
SSA_0942	11.14754	3.934426	3.606557	6.557377	4.590164	8.196721	4.262295	7.868852	1.639344	0.327869	3.934426	11.47541	1.311475	0.327869	1.311475	7.540984	8.852459	2.95082	0.983607	9.180328	P
SSA_0811	10.46154	4	3.692308	6.769231	4.923077	6.153846	3.076923	6.461538	4.615385	3.076923	4	9.230769	0	0.307692	2.769231	4	8.307692	7.076923	6.461538	4.615385	S
SSA_0530	9.340659	0.824176	3.021978	3.571429	4.945055	4.120879	5.21978	10.98901	1.373626	0.274725	4.945055	12.63736	0.274725	0.549451	1.098901	6.318681	11.81319	3.571429	2.747253	12.36264	E
SSA_1040	9.340659	3.571429	4.395604	8.241758	3.021978	4.120879	3.846154	9.89011	2.472527	0.549451	3.296703	11.53846	1.373626	0	2.472527	6.043956	10.71429	3.296703	0.824176	10.98901	R
SSA_0467	9.066667	4.266667	3.733333	4.533333	5.866667	8.533333	3.733333	7.2	2.4	2.133333	3.466667	7.2	0.533333	1.066667	2.666667	2.4	10.13333	8.533333	3.733333	8.8	H
SSA_0150	8.436725	6.451613	4.218362	5.459057	7.19603	4.218362	0.992556	6.947891	4.466501	2.233251	3.473945	7.940447	0.248139	0.496278	4.962779	4.466501	6.947891	7.692308	6.699752	6.451613	
SSA_1793	8.272506	4.86618	6.082725	8.272506	7.785888	5.352798	1.216545	7.29927	4.379562	0.973236	1.216545	10.21898	0.729927	0.973236	5.109489	5.839416	4.379562	6.569343	5.109489	5.352798	T
SSA_1900	8.095238	6.428571	3.809524	5.952381	5.952381	4.285714	2.619048	7.619048	2.619048	1.428571	2.857143	5	0.238095	0	2.619048	4.285714	5.47619	13.09524	6.666667	10.95238	K
SSA_0814	7.762557	2.739726	5.251142	3.652968	6.392694	7.534247	1.826484	7.990868	3.424658	1.141553	4.109589	9.817352	0	0.456621	2.968037	3.652968	8.675799	7.305936	4.794521	10.50228	C
SSA_2215	7.709751	4.081633	3.401361	8.61678	4.761905	5.21542	1.814059	7.936508	1.587302	0.907029	2.040816	15.87302	0.680272	0.226757	7.256236	10.6576	6.575964	2.040816	3.174603	5.442177	
SSA_1658	7.296137	3.004292	2.7897	7.939914	5.150215	5.579399	2.575107	9.871245	1.072961	0.429185	3.862661	12.87554	1.287554	1.072961	3.862661	6.437768	8.798283	2.7897	1.93133	11.37339	E
SSA_1947	6.827309	3.614458	5.02008	8.232932	6.827309	6.827309	2.811245	8.835341	3.614458	0.803213	4.618474	8.634538	1.204819	0.200803	5.421687	5.421687	6.425703	4.016064	4.016064	6.626506	EP
SSA_1131	6.786427	1.397206	3.193613	8.582834	7.984032	5.988024	3.393214	7.185629	3.792415	1.596806	2.195609	12.57485	1.596806	0	1.996008	5.389222	5.588822	7.185629	3.592814	9.98004	P
SSA_2334	6.589147	2.713178	3.682171	6.20155	5.620155	5.813953	2.713178	6.782946	5.03876	1.937984	6.007752	8.914729	0.581395	0.387597	3.875969	5.426357	6.007752	6.589147	6.589147	8.527132	Q
SSA_0443	6.343284	4.104478	3.171642	9.141791	3.544776	2.985075	3.544776	6.156716	2.425373	1.492537	3.358209	23.3209	1.679104	1.30597	4.291045	6.529851	6.529851	3.171642	1.30597	5.597015	
SSA_0297	6.284658	3.881701	4.621072	3.327172	5.545287	6.839187	2.218115	6.284658	3.881701	1.109057	4.990758	10.16636	0.739372	0.369686	4.251386	4.251386	8.133087	7.208872	6.839187	9.057301	C
SSA_1593	6.137184	3.068592	4.693141	7.400722	6.859206	5.595668	1.98556	6.31769	3.429603	1.444043	4.693141	7.039711	1.263538	0.180505	4.33213	4.151625	6.137184	8.66426	7.942238	8.66426	E
SSA_2148	17.85714	1.530612	5.102041	7.142857	11.73469	3.061224	1.020408	3.571429	4.081633	1.530612	0.510204	5.102041	0	0	1.530612	2.55102	6.632653	10.71429	7.653061	8.673469	S
SSA_0032	10.29412	2.647059	2.647059	3.235294	7.058824	3.529412	3.235294	6.764706	2.058824	2.058824	3.529412	9.117647	0	0.882353	3.529412	2.941176	11.47059	10.29412	5.588235	9.117647	F
SSA_2138	10.26393	4.398827	5.278592	6.744868	6.744868	5.278592	1.466276	8.504399	5.571848	1.759531	2.639296	5.278592	0	0	3.225806	1.759531	5.278592	10.85044	6.744868	8.211144	R
SSA_1139	10.20408	3.498542	2.915452	4.081633	6.122449	5.830904	3.790087	6.705539	1.457726	1.457726	4.956268	7.87172	0.58309	1.166181	4.081633	2.915452	9.037901	6.997085	7.28863	9.037901	C
SSA_1175	10.08646	3.746398	5.18732	6.051873	7.204611	7.204611	5.18732	7.204611	2.305476	1.729107	5.18732	9.221902	0.288184	0	2.305476	2.017291	6.340058	6.340058	5.763689	6.628242	C
SSA_1468	9.859155	2.816901	3.380282	5.633803	7.042254	4.507042	2.816901	6.197183	3.661972	3.380282	3.661972	10.14085	1.408451	0.56338	2.816901	3.098592	9.577465	7.887324	4.507042	7.042254	E
SSA_0415	9.722222	2.5	2.777778	7.777778	5.277778	4.722222	2.222222	10.27778	4.444444	0.277778	3.611111	15.55556	0.833333	0.555556	2.5	6.666667	7.5	3.333333	1.388889	8.055556	R
SSA_0777	9.234828	4.485488	5.277045	8.707124	6.860158	6.332454	1.055409	7.387863	2.902375	2.902375	2.638522	7.915567	0.527704	0.263852	5.540897	3.957784	6.332454	7.387863	5.804749	4.485488	G
SSA_2316	8.578431	3.676471	4.166667	6.617647	7.107843	4.901961	4.411765	7.107843	4.656863	0.245098	3.676471	12.9902	0.245098	0.490196	2.45098	3.676471	6.862745	6.617647	2.941176	8.578431	NU
SSA_1512	8.515815	2.919708	5.109489	4.136253	4.86618	5.109489	4.379562	7.542579	3.649635	0.973236	4.136253	15.08516	1.216545	0	4.86618	9.245742	6.569343	2.43309	2.43309	6.812652	S
SSA_0037	8.333333	2.380952	2.619048	4.52381	7.380952	5	1.904762	5.952381	3.333333	0.952381	4.52381	8.571429	0.714286	0	3.571429	4.52381	10.2381	7.380952	7.380952	10.71429	F
SSA_1073	8.333333	4.52381	2.380952	6.190476	6.428571	7.142857	2.857143	6.428571	4.761905	1.904762	2.142857	9.52381	0.238095	0.714286	2.619048	1.666667	5.952381	7.857143	6.190476	12.14286	E
SSA_1955	8.333333	4.047619	4.285714	5.47619	5	4.285714	2.142857	8.333333	4.761905	2.857143	3.095238	8.809524	0.714286	0.238095	2.857143	3.571429	7.380952	7.380952	8.095238	8.333333	O
SSA_0674	7.847534	7.847534	2.914798	6.053812	6.502242	4.484305	1.569507	6.950673	7.174888	2.017937	3.363229	11.21076	0.44843	0	3.587444	2.914798	5.605381	8.520179	4.26009	6.726457	L
SSA_0371	7.8125	4.241071	5.357143	4.464286	5.803571	5.803571	1.785714	5.803571	3.794643	1.116071	3.348214	7.142857	0.669643	0.669643	4.464286	4.464286	10.9375	8.258929	4.017857	10.04464	E
SSA_0031	7.306889	6.05428	3.966597	7.724426	3.757829	4.175365	2.296451	6.263048	3.966597	3.131524	3.131524	8.768267	0.626305	1.25261	4.175365	3.966597	8.768267	8.141962	5.219207	7.306889	F
SSA_0837	6.916996	5.335968	2.964427	5.533597	3.952569	4.545455	1.383399	6.719368	7.905138	1.976285	2.766798	8.695652	0.988142	0.395257	6.521739	5.13834	5.533597	7.70751	6.916996	8.102767	M
SSA_0698	6.809339	4.669261	4.280156	5.252918	7.392996	6.031128	3.696498	5.058366	3.696498	1.55642	4.085603	7.587549	0.77821	0	2.918288	7.198444	7.198444	9.922179	6.031128	5.836576	J
SSA_0393	6.679389	3.625954	5.725191	5.152672	5.725191	5.152672	3.244275	8.396947	5.725191	2.480916	1.526718	12.40458	0.381679	1.145038	3.244275	6.679389	6.48855	4.961832	5.152672	6.10687	V
SSA_1363	5.912162	3.716216	3.378378	6.587838	6.587838	7.094595	2.027027	4.898649	4.391892	1.52027	3.040541	12.83784	2.533784	0.168919	4.72973	5.067568	7.939189	4.560811	5.067568	7.939189	V
SSA_1957	9.278351	4.123711	2.835052	5.927835	3.350515	5.670103	2.835052	10.05155	1.546392	2.061856	5.154639	16.49485	1.546392	0	2.835052	7.216495	8.762887	1.546392	3.350515	5.412371	M
SSA_0060	9.183673	4.846939	2.295918	6.122449	4.846939	5.612245	1.785714	5.357143	6.887755	1.27551	3.826531	10.96939	0.510204	0.255102	3.826531	4.336735	5.612245	5.867347	6.887755	9.693878	M
SSA_1155	8.571429	3.095238	4.52381	5	5.47619	5	1.904762	7.142857	4.285714	2.619048	3.809524	7.619048	0.238095	0.238095	3.095238	4.285714	7.380952	7.857143	5.238095	12.61905	E
SSA_0097	8.470588	4.941176	2.588235	4.941176	2.117647	6.823529	4.705882	8	1.882353	0.470588	2.352941	17.41176	1.882353	0.235294	2.823529	4.705882	10.82353	2.588235	2.588235	9.647059	V
SSA_0778	7.563025	4.201681	3.151261	4.831933	4.411765	4.621849	2.521008	4.621849	3.991597	2.941176	3.781513	10.5042	2.10084	0.630252	5.252101	6.512605	7.983193	6.092437	7.352941	6.932773	G
SSA_0429	6.976744	4.263566	4.457364	7.170543	5.232558	5.426357	1.937984	7.945736	3.875969	2.713178	3.875969	10.65891	0	1.162791	2.325581	2.51938	7.55814	8.139535	4.651163	9.108527	E
SSA_2078	6.509946	5.244123	3.435805	6.690778	5.786618	4.882459	2.169982	6.690778	6.148282	2.712477	2.531646	15.37071	0.542495	1.084991	5.063291	3.435805	2.893309	8.860759	5.244123	4.701627	K
SSA_2274	6.47482	2.338129	6.834532	8.093525	8.992806	5.935252	2.877698	6.654676	3.597122	0.539568	2.338129	6.834532	0.899281	0.179856	3.417266	3.057554	8.81295	6.294964	6.47482	9.352518	
SSA_1021	10.63218	4.310345	3.448276	6.321839	4.310345	6.896552	2.011494	4.885057	4.022989	3.735632	2.873563	7.758621	0.287356	0.862069	4.022989	3.16092	8.62069	7.183908	6.609195	8.045977	G
SSA_2157	9.319899	5.037783	4.534005	6.297229	5.037783	7.808564	2.770781	7.556675	4.282116	1.007557	3.274559	9.823678	0	0.251889	1.763224	3.526448	8.816121	8.564232	3.778338	6.549118	O
SSA_2142	9.273183	2.756892	4.260652	4.761905	7.518797	4.260652	0.75188	6.265664	2.005013	2.756892	4.260652	9.774436	1.754386	1.002506	3.759398	2.255639	6.265664	7.518797	7.769424	11.02757	E
SSA_0893	8.788599	2.612827	6.175772	8.07601	10.68884	8.551069	2.850356	3.562945	6.888361	0.475059	2.137767	6.175772	0.712589	0.23753	2.850356	1.662708	6.413302	8.313539	4.988124	7.83848	M
SSA_0888	8.295964	2.242152	4.26009	5.829596	3.811659	6.053812	3.363229	8.96861	4.932735	1.345291	2.914798	14.57399	0.44843	0.224215	2.466368	5.156951	4.70852	6.950673	6.278027	7.174888	P
SSA_1775	8.240535	4.231626	7.126949	4.454343	6.458797	4.899777	2.449889	10.69042	4.008909	2.672606	2.227171	9.35412	0	0.890869	2.227171	4.67706	6.23608	6.23608	5.790646	7.126949	P
SSA_0980	8.043478	4.130435	2.826087	4.565217	6.304348	5	3.913043	6.304348	3.26087	3.26087	5.434783	5	1.086957	1.521739	2.608696	4.782609	9.347826	5.434783	7.391304	9.782609	E
SSA_0474	7.724426	4.80167	2.296451	5.219207	6.263048	5.010438	2.087683	6.05428	4.175365	2.087683	4.175365	11.27349	1.25261	0.835073	1.461378	3.966597	7.933194	7.933194	5.427975	10.02088	H
SSA_0095	7.489879	2.226721	3.846154	5.465587	5.870445	7.692308	2.024291	5.060729	4.65587	2.42915	3.238866	9.716599	0	0.404858	4.453441	4.453441	5.668016	5.465587	7.287449	12.55061	E
SSA_0263	5.873016	3.492063	4.126984	3.809524	6.666667	4.761905	1.587302	5.079365	4.126984	1.746032	4.920635	8.253968	2.698413	0	4.761905	5.079365	4.920635	7.301587	8.888889	11.90476	O
SSA_2359	5.826772	5.511811	4.094488	5.511811	7.716535	5.511811	3.464567	8.818898	3.622047	1.732283	3.622047	7.401575	0.472441	0.629921	3.307087	2.362205	7.401575	9.133858	5.669291	8.188976	D
SSA_1194	10.61453	3.072626	3.351955	6.145251	6.424581	5.586592	2.793296	6.98324	4.189944	1.955307	4.469274	6.424581	0.837989	1.117318	2.793296	2.793296	6.424581	6.98324	5.586592	11.45251	E
SSA_1532	9.819121	1.550388	6.718346	8.78553	7.235142	8.268734	1.033592	4.909561	5.684755	0.516796	2.325581	6.718346	0.775194	0	3.100775	1.29199	8.268734	5.943152	6.20155	10.85271	M
SSA_1492	8.816705	6.960557	4.87239	6.49652	4.640371	7.888631	4.408353	8.816705	5.104408	1.62413	2.088167	10.44084	0	0	2.552204	3.24826	5.336427	6.960557	4.87239	4.87239	K
SSA_2212	8.775982	5.311778	3.464203	5.773672	4.387991	4.157044	4.618938	8.314088	2.309469	0.230947	1.385681	18.01386	1.385681	0.230947	6.69746	7.3903	5.080831	3.233256	2.771363	6.466513	V
SSA_1111	8.597285	1.131222	3.393665	5.656109	4.524887	7.013575	2.036199	10.63348	3.167421	1.131222	5.20362	13.12217	0.678733	0.452489	3.61991	7.466063	8.371041	2.262443	0.678733	10.85973	E

SSA_0363	8.539326	3.820225	3.146067	5.168539	2.47191	6.292135	2.696629	8.988764	2.696629	0.898876	3.370787	13.93258	1.573034	0.674157	2.247191	6.741573	9.662921	2.921348	1.797753	12.35955	E
SSA_1943	8.26087	4.130435	5	7.173913	5.869565	4.347826	2.391304	9.130435	3.26087	3.043478	4.565217	7.826087	0.434783	0.217391	3.043478	3.478261	6.956522	7.826087	5.217391	7.826087	E
SSA_1913	8.033827	1.057082	3.805497	5.285412	4.016913	8.033827	3.171247	10.57082	1.691332	0.845666	2.959831	10.57082	0.845666	0.634249	1.902748	6.976744	10.35941	2.325581	3.382664	13.53066	R
SSA_2374	7.707911	4.868154	3.853955	5.070994	5.070994	5.882353	2.636917	8.51927	2.636917	2.231237	3.448276	7.099391	0.20284	0.40568	2.231237	3.448276	10.14199	6.896552	6.085193	11.56187	F
SSA_1797	7.61523	2.204409	5.811623	9.018036	9.218437	7.214429	2.204409	4.008016	4.408818	0.601202	4.008016	8.617234	0	0	4.809619	2.60521	5.01002	8.416834	5.210421	9.018036	R
SSA_2234	7.436399	4.892368	4.500978	4.500978	6.457926	3.913894	4.305284	9.197652	1.761252	1.565558	3.522505	9.393346	1.174168	0.195695	6.066536	5.870841	6.262231	7.240705	5.675147	6.066536	I
SSA_0879	6.620209	4.181185	6.445993	6.620209	5.749129	3.658537	1.045296	7.142857	5.923345	2.264808	1.393728	11.67247	0.522648	0	3.135889	3.484321	2.61324	14.45993	5.052265	8.013937	D
SSA_1374	6.574394	5.190311	3.460208	7.093426	2.249135	6.401384	2.076125	8.304498	8.304498	1.730104	2.768166	13.66782	0.519031	0.17301	3.460208	5.190311	5.536332	4.152249	4.49827	8.650519	V
SSA_1816	5.828221	3.834356	7.055215	9.969325	8.128834	3.98773	1.533742	8.742331	2.453988	1.07362	4.141104	9.662577	1.226994	1.07362	3.680982	3.98773	6.134969	7.208589	6.748466	3.527607	
SSA_0791	9.15493	5.164319	3.521127	4.460094	5.633803	6.103286	5.399061	5.633803	3.755869	2.112676	3.286385	9.15493	0.469484	0.234742	1.643192	2.347418	8.685446	8.215962	4.694836	10.32864	M
SSA_0181	8.944954	3.669725	5.504587	5.045872	6.192661	5.504587	3.440367	7.33945	4.357798	1.834862	2.522936	10.55046	2.522936	1.146789	3.899083	6.192661	4.587156	4.357798	4.587156	7.798165	M
SSA_0430	8.666667	1.777778	3.333333	6.666667	5.555556	4	3.111111	11.77778	2.222222	0	4	11.33333	0.888889	0.666667	3.333333	6.888889	8.888889	4.222222	1.555556	11.11111	E
SSA_1063	8.666667	4	3.333333	8.888889	7.555556	11.11111	0.666667	5.555556	2.444444	0.666667	4.888889	6.888889	0.444444	0	3.111111	3.333333	6.444444	6.888889	5.555556	9.555556	H
SSA_1829	8.552632	6.578947	3.070175	3.72807	8.991228	5.263158	2.192982	6.359649	4.385965	1.535088	3.72807	8.552632	0.438596	1.754386	5.482456	3.72807	5.921053	7.45614	5.04386	7.236842	J
SSA_1841	8.369099	3.218884	2.7897	4.935622	6.223176	4.506438	2.145923	6.652361	3.862661	2.575107	4.72103	7.939914	0.858369	0	3.218884	4.72103	9.44206	7.725322	7.939914	8.154506	O
SSA_1621	8.074534	1.656315	4.761905	6.21118	6.004141	4.140787	4.554865	8.695652	3.519669	0.414079	4.140787	13.04348	3.10559	0.414079	3.726708	6.004141	8.281573	2.070393	3.10559	8.074534	E
SSA_2225	7.768924	3.984064	6.374502	8.964143	7.370518	5.976096	3.187251	5.976096	3.585657	2.191235	1.792829	11.35458	0.199203	0	4.38247	2.788845	6.374502	4.38247	5.976096	7.370518	K
SSA_0493	7.632094	1.565558	3.522505	7.632094	10.76321	6.066536	1.761252	4.305284	2.348337	0.978474	4.892368	7.04501	0.782779	0.195695	5.479452	3.913894	6.262231	8.023483	5.870841	10.9589	E
SSA_1204	6.818182	3.321678	3.496503	5.06993	6.818182	5.769231	2.097902	6.818182	2.447552	1.573427	3.671329	6.818182	0.34965	0.174825	4.370629	4.895105	7.167832	8.566434	6.818182	12.93706	G
SSA_2167	6.782609	4.695652	3.826087	8.521739	4.521739	4.869565	4.173913	9.73913	4.695652	0.869565	2.782609	11.30435	1.043478	0	2.26087	5.043478	7.826087	6.26087	3.826087	6.956522	V
SSA_2305	6.759099	4.159445	2.946274	8.665511	5.025997	5.025997	2.079723	3.986135	6.412478	1.039861	3.812825	8.492201	0.519931	0.17331	1.733102	4.679376	4.332756	11.26516	5.199307	13.69151	
SSA_1457	6.678082	3.767123	3.938356	6.335616	6.335616	3.082192	2.568493	5.650685	4.965753	3.424658	3.938356	9.589041	2.226027	1.19863	5.479452	5.479452	4.965753	6.164384	8.90411	5.308219	G
SSA_1949	5.945122	1.676829	5.945122	9.29878	9.146341	7.012195	0.762195	4.115854	3.810976	0.609756	3.353659	6.707317	1.676829	0.152439	5.030488	4.268293	6.707317	7.164634	7.926829	8.689024	E
SSA_2165	5.936073	1.978691	5.175038	8.371385	8.980213	6.697108	1.217656	3.805175	4.414003	1.217656	3.04414	7.914764	1.369863	0.152207	4.870624	3.348554	6.544901	6.697108	8.066971	10.19787	E
SSA_1291	5.547653	4.694168	5.12091	7.112376	9.103841	7.396871	1.280228	4.836415	5.12091	1.70697	2.133713	7.539118	1.991465	0	3.556188	3.129445	8.250356	7.681366	5.547653	8.250356	S
SSA_1002	5.416667	4.861111	5.416667	6.25	4.444444	5.555556	1.944444	5.972222	5.277778	3.055556	3.055556	11.25	1.388889	0.277778	4.722222	3.888889	7.083333	7.5	5.694444	6.944444	G
SSA_0328	5.11811	4.593176	6.036745	6.430446	4.986877	7.217848	1.574803	3.937008	5.774278	2.755906	3.937008	11.54856	1.44357	0.524934	4.593176	5.11811	5.511811	6.299213	7.742782	4.855643	R
SSA_0873	4.791155	4.422604	3.439803	6.265356	6.879607	4.545455	0.859951	5.528256	6.756757	2.702703	2.702703	15.60197	0.614251	0.4914	3.071253	5.282555	4.299754	8.108108	6.265356	7.371007	KL
SSA_1984	3.919598	1.20603	4.221106	25.82915	3.819095	7.638191	0.80402	2.713568	1.708543	0.603015	3.015075	4.422111	1.507538	0	1.60804	5.527638	14.27136	2.311558	9.849246	5.025126	J
SSA_1467	10.30928	6.185567	3.608247	4.896907	6.443299	5.927835	2.835052	6.443299	2.57732	1.804124	4.123711	6.958763	0.773196	0	2.061856	2.57732	10.82474	8.762887	5.412371	7.474227	E
SSA_2381	10.25641	1.282051	7.692308	14.35897	6.666667	8.974359	1.538462	7.948718	3.846154	0.512821	2.051282	7.179487	0	0	2.564103	1.282051	8.974359	4.615385	5.384615	4.871795	O
SSA_0899	10.20408	2.040816	3.316327	6.377551	4.846939	4.336735	2.806122	11.73469	3.826531	1.020408	3.571429	11.9898	2.040816	0	3.316327	7.908163	6.377551	3.571429	2.806122	7.908163	R
SSA_1035	9.411765	3.529412	1.176471	5.411765	6.823529	6.117647	3.529412	8.235294	4.470588	0.705882	1.647059	9.176471	0.470588	0.235294	1.882353	3.294118	9.411765	7.529412	7.294118	9.647059	F
SSA_2185	9.302326	6.511628	4.186047	6.27907	5.348837	5.813953	1.162791	7.906977	2.790698	1.860465	3.953488	7.906977	0.930233	0.697674	3.953488	3.023256	10	6.744186	7.44186	4.186047	F
SSA_2258	9.174312	3.669725	5.275229	6.192661	5.045872	5.963303	3.211009	6.422018	6.422018	1.376147	5.045872	5.963303	1.146789	0.229358	2.981651	4.816514	8.486239	5.275229	5.504587	7.798165	S
SSA_1776	8.350731	2.296451	3.757829	7.098121	4.384134	5.636743	2.713987	9.812109	1.461378	1.670146	2.922756	15.03132	1.25261	0.835073	3.966597	7.933194	8.977035	2.087683	2.713987	7.098121	P
SSA_0617	8.130081	5.691057	4.065041	2.439024	8.943089	3.658537	2.845528	6.504065	7.113821	0.203252	1.829268	7.520325	0.406504	0	0.813008	2.845528	4.471545	12.39837	5.894309	14.22764	S
SSA_1533	7.797271	3.508772	3.898635	5.263158	6.62768	5.847953	2.14425	8.382066	3.703704	3.118908	3.118908	6.62768	0.584795	0.779727	3.898635	4.288499	9.551657	6.237817	5.263158	9.356725	C
SSA_1128	7.722008	2.895753	2.509653	6.949807	4.247104	4.054054	3.861004	8.301158	3.281853	1.544402	3.667954	16.40927	1.544402	0.19305	3.474903	5.598456	9.459459	5.019305	2.316602	6.949807	P
SSA_1120	7.054674	4.409171	2.821869	7.583774	6.525573	4.938272	2.998236	6.878307	8.289242	1.587302	2.469136	12.52205	1.058201	0.176367	4.409171	2.998236	5.114638	7.054674	5.643739	5.467372	T
SSA_1481	6.688963	3.177258	3.010033	6.856187	7.692308	7.023411	1.839465	4.180602	4.849498	2.341137	3.177258	12.04013	2.341137	0.334448	5.016722	5.016722	7.190635	4.682274	5.016722	7.525084	V
SSA_1066	6.125574	1.531394	6.891271	8.882083	8.575804	7.503828	1.071975	3.675345	4.594181	0.612557	3.215926	7.503828	1.378254	0.153139	6.584992	3.369066	6.278714	5.513017	7.810107	8.728943	E
SSA_0435	5.91716	5.029586	5.029586	4.733728	5.473373	5.177515	2.95858	7.39645	2.95858	2.514793	2.95858	7.544379	1.47929	1.183432	4.43787	3.402367	9.467456	7.840237	6.508876	7.988166	E
SSA_1594	5.805515	1.886792	6.240929	6.095791	11.90131	4.644412	2.177068	4.934688	4.208999	1.161103	3.193033	7.837446	1.596517	0	5.660377	4.354136	4.78955	9.5791	6.676343	7.256894	O
SSA_0350	5.076142	4.568528	3.93401	6.091371	5.71066	5.964467	1.649746	8.629442	5.076142	1.77665	3.93401	10.53299	0.380711	0.380711	3.553299	3.93401	5.456853	8.629442	7.233503	7.48731	L
SSA_0572	11.78161	3.16092	2.011494	3.735632	6.609195	4.022989	1.149425	7.471264	3.448276	2.298851	4.597701	8.045977	0.574713	0.862069	3.16092	2.873563	8.908046	9.195402	4.885057	11.2069	ER
SSA_1530	9.71564	3.554502	4.50237	8.056872	4.976303	6.161137	3.554502	8.767773	4.028436	0.473934	2.132701	9.241706	0.473934	0	3.554502	3.080569	8.767773	6.398104	3.317536	9.241706	V
SSA_0270	9.050773	1.545254	4.415011	6.401766	4.415011	5.518764	4.415011	10.59603	2.649007	0.662252	5.518764	10.81678	3.090508	0.220751	3.090508	7.284768	6.843267	1.766004	2.869757	8.830022	G
SSA_1459	8.991228	5.04386	5.04386	3.947368	6.798246	4.385965	2.412281	7.45614	5.04386	1.535088	4.166667	9.429825	0.438596	0.657895	3.72807	3.947368	6.140351	7.45614	5.701754	7.675439	J
SSA_0774	8.649789	3.797468	4.008439	5.063291	6.118143	4.219409	2.109705	9.07173	3.375527	1.265823	4.43038	6.962025	1.054852	0.421941	2.320675	3.586498	8.860759	8.227848	4.219409	12.23629	C
SSA_2084	8.488613	1.863354	3.933747	6.625259	4.761905	4.347826	3.519669	9.937888	1.242236	0.414079	4.140787	13.45756	2.277433	0	2.484472	7.660455	8.902692	4.554865	2.277433	9.109731	G
SSA_2093	8.453608	1.649485	4.329897	3.71134	4.536082	5.154639	1.85567	8.247423	4.123711	1.030928	3.71134	12.98969	1.237113	0.618557	3.298969	9.690722	9.484536	2.061856	2.886598	10.92784	S
SSA_0481	8.216433	5.410822	3.006012	5.01002	5.811623	3.206413	2.60521	7.61523	5.811623	1.202405	4.008016	10.82164	0.200401	0.801603	2.805611	2.60521	8.016032	9.018036	7.61523	6.212425	H
SSA_1039	8.023483	4.500978	4.305284	6.066536	7.436399	4.892368	2.935421	10.37182	3.522505	1.761252	2.935421	9.197652	0	0	1.761252	1.565558	8.610568	9.001957	4.892368	8.219178	R
SSA_0035	7.884615	4.423077	4.230769	4.615385	6.346154	5.384615	1.730769	8.076923	4.230769	1.923077	3.461538	8.076923	0.769231	0.192308	2.692308	3.269231	6.923077	6.730769	7.5	11.53846	F
SSA_1375	7.068966	5.862069	3.275862	7.241379	3.62069	6.896552	2.068966	6.37931	7.241379	1.37931	2.758621	15.51724	1.206897	0	2.586207	4.137931	6.37931	4.827586	3.103448	8.448276	V
SSA_1948	6.202723	1.815431	5.446293	7.413011	9.833585	8.16944	0.605144	2.874433	4.387292	0.907716	3.782148	8.018154	1.512859	0.151286	4.689864	4.08472	5.597579	6.656581	8.472012	9.379728	E
SSA_2268	5.924855	3.901734	5.635838	6.213873	6.069364	4.046243	1.734104	7.225434	5.635838	3.612717	4.046243	9.248555	1.156069	0.578035	5.635838	4.768786	4.768786	5.491329	7.080925	7.225434	G
SSA_1860	5.750351	2.945302	7.012623	9.817672	5.329593	8.5554	3.366059	4.067321	4.347826	0.981767	3.225806	6.87237	0.701262	0.140252	8.274895	2.384292	6.732118	5.049088	5.750351	8.695652	M
SSA_0607	5.269923	3.341902	4.113111	8.226221	6.55527	6.55527	2.699229	6.55527	6.683805	1.542416	3.213368	16.19537	0.771208	0.771208	3.470437	5.141388	4.884319	3.727506	3.984576	6.298201	Q
SSA_1615	11.35135	3.243243	2.702703	3.513514	5.135135	7.297297	2.162162	5.945946	3.783784	2.702703	4.594595	9.459459	0.27027	0	2.432432	1.891892	10.54054	6.216216	4.864865	11.89189	E
SSA_1127	9.170306	2.620087	5.240175	5.240175	7.641921	4.803493	2.838428	6.768559	3.71179	2.183406	2.401747	7.860262	0.218341	1.091703	3.71179	4.148472	7.423581	6.9869	6.768559	9.170306	R
SSA_1163	8.076923	4.615385	3.269231	5.384615	5.576923	5.769231	2.307692	9.038462	3.846154	2.115385	3.846154	8.076923	0.961538	0.769231	2.692308	3.846154	9.423077	6.923077	7.692308	5.769231	F

SSA_0773	7.279029	3.812825	4.159445	3.812825	6.065858	5.892548	4.679376	5.545927	3.639515	1.213172	3.119584	9.012132	0.34662	0.34662	2.426343	3.639515	5.892548	9.532062	6.585789	12.99827	G
SSA_0161	6.511628	4.496124	6.046512	7.131783	6.20155	6.511628	2.790698	5.891473	5.581395	3.100775	3.100775	10.07752	0.775194	0.155039	3.72093	4.651163	6.20155	5.891473	6.20155	4.96124	
SSA_0085	6.451613	3.225806	2.764977	6.758833	5.376344	3.225806	1.382488	4.915515	5.222734	1.536098	3.686636	17.05069	0.921659	0.30722	2.611367	6.605223	6.912442	8.141321	3.840246	9.06298	C
SSA_0716	5.630027	5.227882	2.412869	8.176944	4.289544	4.423592	1.742627	6.970509	5.630027	1.474531	3.887399	16.35389	1.474531	0.134048	3.217158	7.908847	6.16622	4.959786	4.423592	5.495979	R
SSA_0852	5.511811	6.167979	6.167979	6.692913	5.774278	5.511811	2.230971	5.774278	4.330709	1.312336	3.28084	10.10499	0.787402	0.524934	4.068241	3.149606	4.986877	8.005249	6.56168	9.055118	L
SSA_0565	4.878049	2.787456	5.574913	6.504065	8.246225	6.852497	1.74216	1.858304	10.68525	0.929152	1.509872	7.781649	0.58072	0	2.439024	1.16144	3.135889	7.665505	4.878049	20.78978	R
SSA_0427	4.150198	5.039526	3.359684	5.434783	6.422925	3.754941	1.87747	6.422925	7.312253	2.865613	2.470356	14.32806	0.6917	0.790514	5.632411	4.347826	4.347826	8.992095	5.237154	6.521739	R
SSA_0284	9.930716	0.230947	2.771363	6.235566	4.849885	3.002309	3.926097	11.3164	4.618938	0.692841	4.849885	9.699769	1.385681	0.230947	2.771363	8.314088	9.237875	2.771363	2.078522	11.08545	G
SSA_2018	9.662921	2.696629	3.370787	4.94382	6.516854	6.292135	3.595506	9.662921	2.921348	0.674157	3.370787	6.516854	0.224719	1.123596	0.674157	2.921348	9.662921	6.516854	5.842697	12.80899	S
SSA_0518	9.014675	2.725367	3.144654	6.918239	6.289308	5.660377	1.048218	10.69182	5.24109	1.886792	2.93501	9.014675	0.209644	1.048218	3.144654	2.93501	7.756813	6.079665	6.918239	7.337526	E
SSA_0740	8.548708	1.590457	2.186879	7.355865	4.771372	6.759443	4.572565	10.33797	2.186879	0.397614	3.976143	12.52485	1.789264	0.198807	2.584493	5.5666	9.940358	3.379722	2.584493	8.747515	S
SSA_2270	7.363014	5.993151	3.082192	4.452055	5.993151	4.794521	2.568493	4.109589	4.280822	1.712329	4.109589	11.81507	0.856164	0.171233	2.910959	4.452055	5.993151	9.246575	7.534247	8.561644	J
SSA_2265	5.710491	3.320053	5.710491	4.913679	6.507304	4.780876	1.992032	7.835325	3.320053	2.921647	2.788845	11.95219	1.062417	0.398406	4.648074	3.585657	5.312085	7.569721	7.304117	8.366534	G
SSA_1137	9.865471	3.811659	2.690583	4.932735	6.278027	4.484305	1.569507	6.726457	4.484305	2.017937	3.363229	10.98655	0.224215	0.672646	3.811659	2.914798	8.071749	8.744395	4.484305	9.865471	C
SSA_0523	8.943089	3.252033	3.658537	5.894309	8.536585	4.878049	2.439024	7.113821	2.845528	1.422764	4.471545	8.536585	0.406504	1.219512	1.626016	2.642276	7.926829	8.536585	4.065041	11.58537	C
SSA_1112	8.380952	2.095238	6.095238	6.666667	9.333333	7.238095	2.285714	2.285714	4.761905	1.333333	5.714286	9.904762	0.571429	0	2.857143	2.285714	7.428571	6.095238	6.666667	8	
SSA_0805	7.885305	2.508961	11.46953	6.09319	6.272401	10.21505	1.433692	3.584229	4.121864	0.358423	5.376344	8.064516	0.716846	0	3.405018	4.121864	9.139785	4.301075	5.555556	5.376344	M
SSA_0929	7.57315	4.475043	3.442341	6.884682	5.507745	6.196213	3.786575	9.638554	4.302926	1.204819	2.065404	11.53184	1.032702	0.688468	3.270224	4.130809	7.056799	5.507745	5.335628	6.36833	V
SSA_0391	7.445008	3.384095	6.091371	3.891709	6.937394	4.906937	2.199662	6.76819	3.21489	1.522843	4.399323	6.937394	1.353638	0.507614	3.891709	4.399323	7.614213	7.783418	5.922166	10.8291	EH
SSA_1297	7.201309	5.728314	4.582651	4.418985	9.165303	3.764321	1.800327	7.528642	6.710311	1.636661	4.582651	9.165303	0.163666	0.981997	3.600655	4.418985	5.400982	7.528642	6.219313	5.400982	L
SSA_2304	12.46537	1.939058	3.047091	5.263158	6.648199	4.155125	1.385042	6.648199	3.047091	1.108033	2.770083	8.587258	0	0.554017	0.831025	3.047091	5.263158	14.40443	8.310249	10.52632	S
SSA_1998	10.53864	3.512881	3.044496	4.215457	7.728337	4.918033	1.639344	4.449649	3.747073	1.405152	2.810304	7.259953	0.234192	0	2.107728	3.981265	5.854801	14.28571	7.259953	11.00703	O
SSA_0655	9.955752	4.867257	3.761062	5.309735	4.424779	5.530973	2.654867	9.292035	4.646018	1.548673	5.309735	7.079646	0	0	1.99115	3.097345	8.628319	9.070796	5.088496	7.743363	D
SSA_2230	6.155951	5.608755	5.745554	4.651163	6.976744	6.155951	1.915185	6.155951	3.283174	1.778386	4.514364	6.976744	0.957592	1.641587	5.608755	4.377565	6.429549	7.797538	6.29275	6.976744	F
SSA_0419	6.040268	7.248322	3.355705	6.577181	2.95302	4.832215	1.47651	3.758389	5.637584	2.416107	4.42953	11.27517	2.147651	0.536913	4.966443	5.100671	6.979866	8.053691	5.637584	6.577181	G
SSA_1849	5.660377	6.037736	3.899371	6.163522	5.660377	5.031447	2.138365	5.91195	7.295597	2.138365	3.773585	10.81761	0.503145	1.006289	4.150943	3.647799	5.660377	6.792453	5.786164	7.924528	L
SSA_1016	5.441354	5.199516	2.539299	6.771463	5.320435	5.078597	2.660218	6.166868	8.585248	1.571947	4.111245	11.4873	1.330109	0.846433	2.781137	4.474002	5.562273	8.34341	4.111245	7.617896	G
SSA_1344	10.77283	0	2.34192	6.557377	5.152225	4.918033	3.747073	10.30445	2.34192	1.405152	4.918033	13.11475	0.468384	0	3.512881	5.152225	10.77283	1.639344	3.747073	9.133489	F
SSA_1043	10.74766	2.803738	4.205607	5.607477	6.542056	5.140187	2.102804	7.009346	3.037383	1.869159	2.803738	8.878505	0.233645	0	2.102804	4.439252	7.71028	7.71028	5.841121	11.21495	E
SSA_2318	8.214286	5.535714	3.75	5.178571	6.25	7.321429	2.5	10.35714	3.392857	1.607143	3.035714	7.678571	0	0.714286	3.214286	2.142857	6.607143	9.642857	5.357143	7.5	NU
SSA_0067	7.65391	3.161398	3.161398	7.65391	4.326123	6.156406	2.828619	7.487521	3.660566	1.830283	3.161398	13.64393	1.331115	0.166389	4.326123	6.988353	7.487521	3.993344	3.826955	7.154742	I
SSA_1827	7.565789	5.098684	5.098684	7.401316	6.085526	5.263158	2.138158	4.440789	2.302632	1.480263	3.453947	11.01974	0.493421	0	2.960526	3.618421	7.565789	8.717105	6.907895	8.388158	C
SSA_1356	6.94864	6.797583	3.776435	4.682779	5.287009	5.287009	3.625378	7.854985	4.229607	1.963746	3.323263	8.308157	0	0.151057	3.625378	3.776435	6.042296	10.57402	7.55287	6.193353	L
SSA_1027	8.623853	3.119266	4.40367	7.522936	4.954128	3.486239	4.40367	9.174312	2.93578	1.284404	3.119266	16.14679	1.284404	0	4.770642	7.706422	5.321101	2.752294	1.46789	7.522936	R
SSA_1970	8.303887	2.826855	3.003534	4.59364	7.067138	6.183746	3.003534	9.010601	4.416961	2.473498	4.946996	6.890459	0.530035	0.353357	3.35689	3.35689	8.833922	6.713781	5.477032	8.657244	EH
SSA_1960	7.448494	4.278922	5.229794	7.92393	4.437401	3.645008	2.060222	5.229794	3.645008	1.743265	2.852615	11.8859	1.267829	0.633914	5.546751	4.912837	8.557845	4.754358	6.656101	7.290016	S
SSA_0036	7.241911	3.697997	5.701079	8.474576	5.701079	9.861325	0.770416	4.776579	4.776579	2.927581	4.160247	4.776579	2.619414	0.308166	5.855162	2.773498	8.474576	4.776579	5.546995	6.779661	R
SSA_1950	7.19755	1.531394	6.125574	9.647779	9.188361	5.972435	1.071975	4.594181	4.74732	0.765697	2.756508	6.431853	1.378254	0.153139	5.666156	3.828484	5.206738	5.513017	8.882083	9.341501	E
SSA_2027	6.841339	4.075691	3.63901	8.588064	7.714702	5.094614	2.765648	7.423581	4.657933	1.746725	1.746725	13.68268	0.436681	0.873362	3.202329	4.075691	5.676856	6.695779	5.822416	5.240175	P
SSA_0384	6.629055	3.949224	3.526093	7.052186	9.449929	4.795487	1.692525	5.641749	5.218618	1.551481	3.949224	7.757405	0.846262	0.564175	4.654443	3.808181	6.06488	8.744711	7.898449	6.205924	D
SSA_1012	5.655836	4.693141	2.647413	5.89651	4.933815	4.211793	2.045728	5.174489	7.942238	1.805054	3.730445	13.23706	2.166065	1.323706	3.730445	3.369434	5.655836	8.66426	5.294826	7.821901	G
SSA_1994	8.938547	4.841713	3.538175	4.655493	6.145251	5.214153	2.420857	6.517691	4.283054	2.420857	4.469274	8.752328	0.372439	0.931099	3.538175	3.165736	7.44879	7.07635	6.703911	8.566108	F
SSA_0385	8.362369	1.393728	4.703833	6.620209	7.491289	7.317073	4.006969	7.491289	3.484321	0.87108	4.355401	10.97561	2.439024	0	1.916376	3.658537	6.620209	5.226481	4.529617	8.536585	E
SSA_2250	7.272727	1.515152	5.757576	8.787879	8.181818	5.454545	2.727273	12.12121	4.090909	0.606061	2.575758	11.81818	0.757576	0.30303	4.393939	5.30303	5.151515	5.151515	3.939394	4.090909	S
SSA_1359	6.648199	1.800554	3.878116	7.34072	9.279778	7.063712	3.047091	8.725762	4.01662	0.277008	3.047091	6.925208	0.692521	0	3.739612	4.98615	7.34072	4.98615	5.955679	10.24931	E
SSA_0836	6.052963	5.674653	3.278689	5.54855	6.431274	5.044136	3.152585	5.92686	6.30517	2.143758	2.648172	11.0971	0.504414	0.126103	4.665826	3.404792	5.296343	9.079445	5.422446	8.196721	U
SSA_0533	8.860759	5.424955	4.882459	5.786618	5.063291	5.424955	3.254973	5.424955	4.520796	0.723327	4.159132	7.052441	0.904159	0.904159	2.169982	3.797468	7.414105	7.9566	6.509946	9.764919	Q
SSA_1200	8.81295	3.956835	4.676259	3.23741	6.654676	6.47482	1.978417	8.453237	1.618705	1.798561	4.676259	9.172662	0.539568	0.719424	2.158273	2.697842	8.273381	6.834532	6.115108	11.15108	F
SSA_0432	8.797127	2.513465	4.308797	4.308797	8.078995	4.308797	1.795332	7.181329	2.513465	2.513465	3.770197	9.694794	0.359066	0.897666	1.974865	2.872531	9.874327	5.745063	6.463196	12.02873	F
SSA_1693	8.626761	1.93662	5.809859	4.401408	5.105634	5.633803	2.640845	8.098592	2.112676	0.704225	4.929577	10.21127	1.056338	0.528169	2.640845	7.746479	7.922535	5.105634	2.992958	11.79577	G
SSA_2286	8.566434	3.321678	2.797203	4.895105	6.643357	5.944056	4.370629	6.118881	1.573427	2.447552	5.244755	7.167832	0.699301	1.048951	2.097902	2.272727	11.36364	5.594406	7.167832	10.66434	EG
SSA_1107	8.477509	4.844291	3.287197	5.536332	5.363322	6.055363	2.941176	7.612457	4.844291	1.038062	3.114187	11.07266	1.038062	0	3.460208	5.536332	5.709343	7.266436	4.67128	8.131488	V
SSA_1318	8.032787	4.918033	3.442623	5.081967	7.04918	4.262295	2.786885	8.52459	3.934426	0.983607	3.934426	9.180328	0	0.491803	3.114754	3.934426	5.901639	7.868852	7.704918	8.852459	M
SSA_0091	7.865169	6.099518	2.728732	6.260032	4.012841	5.617978	2.728732	7.383628	5.29695	1.284109	4.012841	8.82825	1.123596	0.321027	2.568218	3.5313	8.82825	8.186196	5.136437	8.186196	C
SSA_0156	7.680251	5.172414	4.231975	4.231975	8.934169	6.426332	1.880878	8.46395	3.448276	0.940439	3.291536	8.934169	0.15674	0.15674	2.821317	4.231975	6.112853	10.03135	7.053292	5.799373	O
SSA_2075	7.446809	3.951368	3.799392	5.015198	4.559271	5.319149	1.519757	4.711246	3.191489	2.431611	4.255319	8.358663	1.06383	0.151976	4.103343	3.951368	8.81459	7.294833	6.079027	13.98176	G
SSA_2209	6.603774	2.96496	6.469003	7.008086	6.199461	8.894879	2.021563	4.716981	4.851752	0.673854	3.09973	7.681941	0.943396	0	4.986523	3.908356	8.894879	5.795148	5.390836	8.894879	M
SSA_1917	14.74926	2.949853	2.949853	3.539823	5.60472	5.60472	1.769912	5.014749	3.834808	2.359882	2.949853	6.489676	0.589971	2.359882	1.769912	2.949853	10.91445	7.079646	5.899705	10.61947	R
SSA_0462	8.547009	2.735043	4.957265	6.324786	4.615385	5.641026	3.247863	8.547009	5.641026	1.709402	2.051282	11.28205	0.512821	0	3.247863	5.128205	6.837607	4.615385	5.470085	8.888889	V
SSA_0227	8.103728	3.079417	6.482982	9.23825	7.13128	7.779579	0.810373	6.158833	3.403566	1.134522	3.403566	5.834684	0.972447	0	4.213938	3.727715	8.265802	5.024311	7.13128	8.103728	M
SSA_0159	7.598784	2.583587	6.838906	8.966565	6.838906	4.559271	1.975684	6.534954	3.343465	0.455927	2.431611	8.358663	1.215805	0	3.191489	4.863222	11.8541	3.343465	3.799392	11.2462	U
SSA_0015	7.587253	5.159332	4.552352	6.221548	7.283763	4.552352	3.186646	7.283763	3.793627	1.365706	3.186646	7.587253	0.151745	0	2.124431	4.097117	8.497724	9.711684	5.007587	8.649469	O
SSA_0669	6.613757	4.89418	4.89418	5.026455	7.010582	5.15873	2.248677	7.671958	5.291005	2.116402	4.365079	8.597884	0	0.529101	2.248677	3.174603	8.333333	7.936508	7.407407	6.481481	O

SSA_0355	6.435006	5.791506	5.148005	5.920206	6.692407	5.019305	1.801802	6.949807	5.791506	1.930502	2.702703	12.09781	0.3861	0	1.673102	3.346203	5.534106	9.90991	5.148005	7.722008	L
SSA_1521	5.274262	4.957806	5.907173	7.911392	5.907173	5.590717	2.42616	8.016878	4.535865	1.898734	2.531646	11.18143	0.949367	0.527426	4.535865	3.586498	4.64135	8.649789	5.590717	5.379747	C
SSA_0461	8.673469	4.761905	3.061224	7.653061	4.931973	6.462585	3.061224	7.482993	4.931973	0.85034	2.721088	11.90476	0.85034	0	2.55102	4.761905	5.952381	6.122449	5.102041	8.163265	V
SSA_1423	6.891892	3.108108	3.918919	4.459459	6.351351	4.72973	2.027027	5.945946	4.864865	1.621622	3.648649	12.83784	0.405405	0.27027	4.054054	4.189189	6.216216	8.513514	7.567568	8.378378	L
SSA_1136	6.827309	6.69344	3.748327	4.283802	5.756359	4.685408	1.338688	7.228916	6.827309	2.008032	3.078983	12.58367	0.267738	0.267738	2.811245	3.614458	6.157965	8.835341	5.62249	7.362784	O
SSA_1482	6.666667	4.052288	4.575163	6.405229	4.575163	5.359477	2.48366	5.490196	5.620915	3.921569	3.398693	7.320261	1.960784	0.653595	6.013072	4.444444	6.797386	5.882353	8.104575	6.27451	G
SSA_0779	6.390977	5.388471	5.012531	4.385965	6.766917	4.511278	2.005013	8.145363	3.508772	2.506266	3.007519	9.774436	1.503759	0	4.511278	4.010025	5.513784	8.145363	7.518797	7.393484	G
SSA_0285	6.273063	4.920049	4.428044	5.658057	6.888069	5.535055	2.460025	6.273063	3.075031	2.214022	4.059041	8.856089	0.738007	0.98401	4.797048	3.690037	6.642066	7.626076	6.396064	8.487085	C
SSA_1109	8.783784	3.716216	5.067568	6.081081	5.574324	4.898649	2.871622	7.601351	7.094595	1.182432	1.858108	12.33108	0.675676	0	3.716216	5.236486	5.912162	4.898649	4.898649	7.601351	V
SSA_0549	7.749627	5.514158	2.384501	4.321908	6.85544	5.067064	3.129657	5.067064	5.961252	1.937407	3.874814	12.22057	0.894188	0	2.831595	3.725782	6.557377	7.749627	5.812221	8.345753	LK
SSA_0689	7.55814	3.052326	5.523256	9.011628	6.976744	8.866279	2.325581	5.232558	4.651163	1.162791	2.761628	7.994186	0.436047	0	4.360465	3.197674	7.267442	5.668605	6.395349	7.55814	M
SSA_0175	6.933333	2.666667	6.933333	8.666667	6	7.066667	3.066667	6	3.866667	1.2	3.333333	6.266667	1.066667	0	5.333333	2.266667	10	4.666667	5.733333	8.933333	M
SSA_1505	6.175772	2.969121	5.344418	7.83848	6.769596	5.225653	2.850356	5.938242	3.800475	1.900238	4.038005	14.60808	1.068884	0.118765	5.581948	7.482185	4.750594	4.750594	4.394299	4.394299	M
SSA_1053	5.956472	4.238259	3.436426	5.727377	6.185567	4.810997	2.520046	6.185567	5.956472	1.832761	4.467354	9.965636	0.343643	1.260023	2.863688	4.810997	6.9874	7.90378	5.383734	9.163803	G
SSA_1659	8.006042	2.265861	4.833837	6.042296	7.250755	4.380665	3.625378	9.063444	4.682779	0.60423	2.114804	14.04834	0	0.453172	4.984894	7.703927	7.099698	3.625378	2.870091	6.344411	Q
SSA_1635	7.692308	3.628447	7.692308	7.256894	6.386067	10.15965	1.596517	5.079826	5.079826	1.306241	3.773585	8.563135	1.306241	0	3.918723	4.499274	5.079826	5.515239	6.82148	4.644412	
SSA_2096	7.44382	5.898876	6.601124	3.792135	6.460674	5.337079	2.106742	6.601124	4.353933	1.544944	2.808989	8.707865	0	0	2.52809	2.668539	7.58427	8.567416	7.303371	9.691011	O
SSA_2199	6.551298	5.562423	2.966625	6.05686	6.674907	5.067985	2.719407	5.315204	4.697157	1.97775	2.966625	11.61928	0.247219	0	2.472188	3.461063	7.169345	8.776267	6.304079	9.394314	O
SSA_1991	6.54321	3.333333	5.185185	6.91358	7.777778	5.925926	0.864198	6.049383	5.185185	3.209877	5.679012	8.641975	0.493827	0	5.061728	1.851852	5.679012	7.283951	6.91358	7.407407	
SSA_2260	6.242638	4.829211	2.944641	6.242638	5.064782	4.829211	3.180212	6.007067	5.418139	2.709069	3.651355	11.66078	0.353357	0.117786	3.651355	3.180212	6.124853	8.951708	5.182568	9.658422	L
SSA_0152	5.578947	5.894737	5.263158	8.526316	7.157895	5.157895	1.894737	6.421053	3.368421	1.473684	3.368421	9.894737	0.842105	0.105263	4.315789	5.789474	5.368421	7.263158	6.526316	5.789474	U
SSA_1174	9.507042	2.288732	4.049296	4.049296	7.394366	5.28169	3.521127	8.978873	1.056338	1.760563	3.521127	8.098592	0.176056	0.352113	2.112676	1.93662	10.56338	10.56338	4.753521	10.03521	C
SSA_1845	8.752026	4.376013	3.241491	11.50729	7.293355	6.320908	2.269044	6.482982	4.376013	1.458671	4.862237	6.969206	0	0	3.889789	1.944895	5.024311	6.807131	5.672609	8.752026	RTKL
SSA_0650	8.206687	5.6231	3.495441	5.015198	5.927052	7.446809	3.191489	8.054711	3.799392	1.215805	4.103343	8.510638	0.303951	0.455927	1.975684	3.951368	6.838906	9.118541	6.838906	5.927052	T
SSA_1100	7.594937	4.500703	2.390999	8.157525	5.063291	4.500703	1.547117	8.438819	4.922644	2.109705	3.094233	13.22082	0.703235	0.421941	3.094233	5.766526	6.610408	5.907173	4.078762	7.876231	V
SSA_0134	6.716418	2.61194	6.716418	8.58209	6.59204	6.716418	2.985075	6.218905	3.9801	1.243781	3.233831	6.59204	1.368159	0	4.975124	2.736318	9.825871	4.850746	5.472637	8.58209	M
SSA_1301	6.330598	3.282532	7.151231	4.806565	6.799531	7.151231	0.937866	4.454865	7.620164	1.172333	2.461899	7.854631	0.3517	0.117233	1.875733	1.524033	4.572098	8.089097	3.985932	19.46073	S
SSA_1978	9.927798	1.98556	4.512635	6.137184	5.595668	6.31769	3.610108	6.137184	3.971119	1.624549	2.888087	7.039711	0	0.180505	2.34657	3.610108	8.122744	9.927798	5.595668	10.46931	R
SSA_1082	8.320726	1.664145	3.32829	5.748865	6.202723	4.84115	3.630862	6.959153	2.723147	1.059002	3.933434	11.34644	0	0.151286	2.420575	4.236006	11.04387	5.446293	4.236006	12.70802	G
SSA_2358	8.005822	4.803493	4.949054	8.879185	5.240175	4.512373	4.221252	8.005822	3.49345	1.746725	1.455604	8.879185	0.291121	0.291121	3.056769	5.531295	5.822416	6.404658	6.841339	7.569141	T
SSA_0146	6.900878	2.38394	3.764115	8.155583	7.904642	6.148055	0.501882	4.265997	5.144291	1.380176	4.015056	7.528231	0.250941	0.125471	0.878294	1.882058	4.516939	11.79423	6.900878	15.55834	L
SSA_2343	6.815366	1.239157	5.576208	12.39157	5.204461	8.921933	2.230483	4.337051	5.947955	0.495663	1.982652	12.39157	0.743494	0	1.982652	1.982652	8.302354	2.973978	4.956629	11.52416	S
SSA_2377	6.387921	2.555168	6.387921	8.130081	5.691057	7.665505	2.206736	7.200929	3.368177	1.393728	2.9036	12.31127	1.277584	0.348432	5.458769	9.872242	4.994193	3.252033	4.529617	4.065041	S
SSA_1817	6.077348	5.19337	7.734807	8.508287	7.513812	4.861878	2.209945	8.729282	3.425414	1.21547	3.314917	9.281768	1.436464	0.110497	4.198895	3.756906	4.41989	7.403315	6.187845	4.41989	
SSA_1339	4.65707	3.556308	3.640982	8.636749	6.604572	4.572396	1.016088	4.403048	8.298052	3.048264	5.08044	8.890771	0.254022	0.084674	5.249788	2.96359	6.181202	6.689246	7.19729	8.975445	
SSA_2146	10.09009	5.765766	4.324324	5.765766	7.027027	3.963964	2.162162	8.288288	2.162162	1.801802	2.882883	8.648649	0.36036	0	2.702703	4.144144	7.747748	7.207207	7.027027	7.927928	R
SSA_0975	9.79021	2.447552	4.72028	6.643357	6.118881	6.468531	0.524476	7.342657	3.321678	2.972028	2.622378	7.517483	0.34965	0.699301	1.748252	5.594406	6.468531	8.216783	6.993007	9.440559	E
SSA_1666	8.682171	3.410853	6.356589	6.666667	6.821705	8.837209	1.085271	4.806202	3.255814	1.085271	3.100775	7.44186	1.085271	0.620155	3.410853	3.875969	7.906977	6.046512	7.286822	8.217054	M
SSA_0416	7.466667	4.666667	3.6	4.533333	7.066667	4.933333	0.933333	6.266667	2.933333	2	4.666667	10.4	1.6	0.4	2.933333	5.066667	5.733333	9.2	6.533333	9.066667	E
SSA_0342	7.263294	5.188067	5.317769	5.317769	5.577173	7.003891	2.594034	5.577173	2.594034	2.204929	3.372244	7.782101	1.167315	0.907912	5.836576	3.761349	6.744488	7.133593	7.652399	7.003891	C
SSA_1680	8.558559	3.303303	3.903904	6.756757	7.057057	7.507508	3.303303	6.156156	3.903904	1.201201	1.951952	15.61562	0.45045	0.3003	5.105105	4.654655	6.156156	3.603604	3.603604	6.906907	
SSA_1551	8.028169	5.070423	3.380282	6.056338	7.746479	4.647887	0.985915	7.605634	3.661972	1.690141	3.239437	11.12676	0.28169	0	2.112676	3.239437	5.211268	9.577465	5.915493	10.42254	K
SSA_1234	7.927677	2.225313	5.563282	4.867872	7.927677	7.788595	0.973574	5.006954	3.477051	1.668985	6.258693	6.954103	0.139082	0	3.059805	2.642559	7.510431	6.815021	6.119611	13.07371	F
SSA_0250	7.713126	6.08931	4.330176	4.600812	9.47226	5.006766	2.70636	7.171854	3.653586	2.976996	2.435724	7.577808	0.811908	0.811908	4.05954	2.841678	6.359946	8.79567	5.548038	7.036536	TK
SSA_0955	6.737589	3.900709	4.137116	6.146572	6.264775	5.791962	2.12766	4.964539	3.900709	1.891253	3.073286	10.04728	2.12766	0.236407	3.782506	4.491726	5.082742	8.392435	6.737589	10.16548	E
SSA_1802	5.517909	5.227493	2.613746	7.260407	6.485963	3.872217	2.226525	6.389158	5.808325	1.548887	2.904163	11.61665	0.580833	0.484027	3.775411	6.292352	5.227493	9.486931	6.873185	5.808325	KL
SSA_2039	9.965636	2.920962	3.436426	7.731959	4.639175	6.701031	3.092784	8.591065	2.57732	2.405498	3.608247	12.37113	1.546392	0.171821	4.639175	4.639175	6.872852	3.092784	2.061856	8.934708	E
SSA_1591	8.504399	2.492669	5.278592	8.944282	6.891496	7.478006	2.199413	4.692082	4.83871	1.759531	4.545455	7.038123	1.026393	0.146628	3.665689	3.958944	5.278592	5.42522	7.038123	8.797654	E
SSA_2020	5.603865	3.091787	5.700483	5.024155	7.149758	6.47343	1.835749	4.7343	4.541063	1.545894	5.507246	9.275362	1.256039	0	4.444444	3.768116	7.149758	5.31401	7.246377	10.33816	D
SSA_2166	10.01698	4.414261	3.395586	6.960951	3.904924	6.451613	3.395586	7.979626	5.772496	1.867572	2.37691	10.01698	0.509338	0.169779	2.716469	5.942275	6.112054	5.093379	5.942275	6.960951	V
SSA_2221	9.833333	4.166667	3.166667	6.5	7.5	5.833333	2.5	9	4.166667	1.666667	3	9.833333	0.166667	0.666667	3.833333	3.5	6.666667	6.666667	4.666667	6.666667	MG
SSA_0140	8.02139	2.673797	2.406417	8.02139	5.080214	5.080214	3.074866	6.818182	4.812834	1.470588	3.475936	13.50267	0.40107	0.534759	1.871658	2.941176	7.754011	6.283422	4.411765	11.36364	P
SSA_1560	5.093379	5.687606	3.904924	7.894737	7.640068	4.75382	1.358234	5.178268	7.300509	0.933786	1.358234	12.64856	0.08489	0	1.782683	3.140917	4.159593	11.71477	6.196944	9.168081	D
SSA_1023	6.84624	4.377104	4.489338	8.305275	7.070707	5.611672	2.244669	6.17284	3.928171	1.683502	5.948373	6.621773	0.561167	0	5.274972	3.928171	6.17284	8.754209	7.070707	4.938272	
SSA_0537	10.03236	4.20712	3.236246	5.66343	4.530744	5.66343	2.10356	10.84142	3.721683	1.941748	3.559871	8.414239	0.161812	0	1.941748	2.427184	9.223301	6.957929	6.148867	9.223301	FP
SSA_1626	8.093995	4.830287	3.263708	6.527415	6.657963	4.177546	2.349869	6.527415	2.741514	0.913838	4.046997	10.57441	0.522193	0	2.219321	4.699739	6.657963	9.138381	5.874674	10.18277	D
SSA_0712	7.958922	3.594352	3.851091	7.830552	4.878049	6.161746	2.053915	9.114249	3.722721	1.797176	3.209243	12.7086	0.770218	0.770218	2.182285	5.006418	5.776637	5.648267	4.749679	8.215661	P
SSA_1588	6.835722	4.520397	4.079383	8.710033	8.158765	4.851158	2.205072	6.174201	4.63065	1.323043	2.75634	12.56891	0.441014	0	3.858875	4.189636	5.953693	5.953693	4.410143	8.379272	V
SSA_1513	6.243706	5.337362	4.330312	6.646526	6.847936	3.826788	1.611279	7.35146	5.538771	1.812689	2.719033	11.07754	0.805639	0.805639	5.941591	3.927492	4.128902	7.452165	7.55287	6.042296	M
SSA_0243	7.845579	2.864259	6.973848	4.856787	6.351183	8.219178	1.120797	6.102117	4.109589	1.369863	5.479452	7.721046	0.622665	0	3.860523	2.49066	7.47198	5.603985	6.351183	10.58531	F
SSA_2049	8.731241	4.502046	2.319236	4.502046	7.366985	5.047749	2.455662	7.230559	3.683492	2.592087	4.229195	7.230559	0.136426	0.136426	2.592087	3.001364	7.639836	9.822647	6.548431	10.23192	J
SSA_1752	9.461426	2.328967	3.202329	6.404658	5.385735	5.676856	2.765648	8.296943	3.347889	1.746725	4.949054	9.606987	1.455604	0.291121	1.892285	5.676856	9.315866	5.240175	3.347889	9.606987	G
SSA_1451	5.310458	6.372549	3.431373	7.679739	5.718954	3.513072	1.879085	5.473856	6.862745	1.470588	2.859477	11.60131	0.490196	0.163399	4.003268	4.901961	4.411765	8.74183	6.454248	8.660131	L

SSA_0379	10.26439	1.55521	2.177294	4.821151	5.598756	5.754277	2.799378	8.242613	2.643857	1.244168	4.821151	11.04199	0.622084	0.466563	2.177294	6.065319	9.486781	4.199067	4.66563	11.35303	G
SSA_1602	8.567775	6.265985	3.324808	6.777494	9.462916	4.347826	2.685422	6.521739	3.196931	2.685422	2.813299	7.033248	0	0.127877	3.069054	4.092072	5.882353	9.71867	6.777494	6.649616	K
SSA_0008	5.741217	5.055698	4.884319	6.341045	7.626392	4.113111	1.628106	8.48329	5.826907	1.885176	2.399314	10.62554	0.17138	0.17138	4.45587	4.712939	4.970009	8.311911	6.940874	5.655527	LK
SSA_1750	9.078772	3.204272	5.874499	9.479306	6.809079	5.607477	1.068091	3.738318	5.473965	1.201602	5.340454	7.076101	0.267023	0	3.204272	3.471295	7.610147	6.275033	5.607477	9.612817	R
SSA_0068	7.692308	4.411765	4.18552	5.090498	7.013575	5.769231	2.941176	6.108597	3.054299	2.60181	4.298643	6.561086	0.678733	1.357466	3.054299	4.18552	6.447964	8.031674	5.090498	11.42534	C
SSA_0456	10.84906	1.572327	3.773585	4.08805	5.031447	7.389937	2.044025	6.761006	2.515723	1.257862	3.773585	10.37736	0.943396	0.314465	2.044025	5.974843	10.69182	4.716981	4.559748	11.32075	G
SSA_1019	8.560794	3.101737	6.327543	6.203474	7.940447	11.29032	0.744417	3.225806	2.35732	0.496278	5.210918	6.203474	0.992556	0	4.218362	4.094293	7.19603	5.955335	7.07196	8.808933	M
SSA_0702	7.89177	3.945885	5.29876	5.975197	5.29876	4.735062	2.029312	6.087937	4.058625	1.578354	4.396843	10.03382	0.563698	0.563698	4.622322	3.382187	8.117249	7.215333	5.975197	8.229989	C
SSA_1065	7.634409	3.225806	5.053763	7.526882	5.913978	5.698925	2.150538	5.806452	5.698925	0.860215	4.946237	9.892473	0.322581	0	2.473118	2.795699	6.989247	5.053763	6.451613	11.50538	G
SSA_0956	5.178702	4.01167	4.449307	7.075128	7.148067	5.324581	0.510576	3.574034	8.825675	0.875274	6.564551	8.971554	0.364697	0.072939	5.032823	1.823487	2.552881	11.81619	3.428155	12.39971	L
SSA_1896	7.849462	7.204301	5.16129	4.623656	8.817204	4.946237	2.043011	5.913978	5.16129	1.505376	4.83871	4.516129	0.215054	0	1.290323	2.473118	6.88172	9.354839	5.806452	11.39785	J
SSA_0448	7.847296	5.620361	4.029692	6.150583	6.150583	5.726405	1.378579	7.211029	3.605514	2.651113	3.923648	9.013786	0.212089	1.16649	2.969247	3.49947	9.013786	6.78685	6.468717	6.574761	L
SSA_0453	6.001622	3.325223	5.352798	7.623682	7.948094	6.48824	1.540957	4.298459	4.541768	1.540957	4.298459	7.380373	1.216545	0	4.622871	4.136253	7.055961	5.67721	7.866991	9.083536	G
SSA_0830	7.35568	4.283054	4.096834	6.331471	4.562384	3.538175	2.048417	6.238361	5.493482	2.979516	3.724395	10.80074	1.862197	1.210428	4.934823	4.562384	5.027933	7.635009	6.98324	6.331471	M
SSA_1734	9.029345	3.498871	3.273138	6.884876	5.417607	6.433409	3.273138	8.465011	2.934537	0.790068	3.273138	12.64108	0.902935	0.451467	2.934537	4.063205	5.191874	7.336343	6.207675	6.997743	P
SSA_2282	8.21501	1.926978	8.01217	10.34483	7.200811	6.795132	1.419878	5.578093	5.375254	0.40568	1.724138	10.75051	0.30426	0	3.042596	3.651116	5.070994	5.780933	5.375254	9.026369	S
SSA_1809	11.23288	1.917808	3.561644	6.027397	5.479452	6.027397	3.835616	7.39726	2.465753	1.643836	3.561644	7.945205	1.232877	0.136986	2.60274	5.479452	9.863014	4.931507	4.246575	10.41096	G
SSA_1341	7.743154	4.34372	2.832861	6.326723	5.760151	5.09915	2.644004	7.837583	3.777148	1.416431	4.532578	8.78187	0.188857	0.944287	3.399433	3.588291	7.082153	8.78187	5.85458	9.065156	EF
SSA_1099	5.822613	3.114421	6.702776	9.817197	5.958023	5.484089	1.354096	5.551794	3.317536	0.947867	2.775897	9.614083	0.541638	0	3.588355	3.859174	10.62965	5.754909	9.140149	6.025728	Q
SSA_0257	9.0625	3.229167	6.25	7.1875	5.520833	8.333333	0.833333	3.958333	6.666667	2.604167	4.583333	4.791667	1.5625	0.208333	5	2.5	7.5	3.854167	6.666667	9.6875	
SSA_0866	9.761388	3.362256	3.253796	5.965293	6.182213	5.748373	2.603037	8.02603	3.470716	1.409978	3.253796	10.30369	0.542299	0.10846	2.060738	3.362256	7.266811	6.616052	6.290672	10.41215	P
SSA_0684	7.069914	1.413983	4.399057	5.891595	9.033778	11.86174	0.942655	3.299293	3.299293	1.492537	5.420267	6.755695	1.806756	0.078555	2.985075	4.320503	6.677141	7.148468	7.227023	8.876669	
SSA_2277	6.309362	4.68114	3.799186	5.766621	5.766621	5.223881	2.238806	6.445047	5.970149	2.713704	4.816825	13.02578	0.949796	0.135685	4.002714	3.188602	5.698779	8.005427	5.698779	5.563094	D
SSA_0613	5.931122	3.571429	6.951531	4.783163	8.482143	6.82398	1.977041	4.145408	6.25	1.084184	2.359694	6.25	1.658163	0	5.420918	3.69898	7.844388	5.420918	7.270408	10.07653	R
SSA_0030	8.199357	3.617363	3.536977	6.189711	5.948553	5.22508	1.688103	6.109325	4.823151	1.607717	3.938907	7.958199	0.562701	0.401929	3.054662	4.903537	7.958199	8.118971	6.109325	10.04823	F
SSA_0303	6.772908	2.456839	5.312085	5.245684	8.432935	9.030544	0.863214	3.519256	4.847278	0.664011	5.577689	6.108898	0.398406	0.066401	4.913679	2.788845	5.444887	7.171315	6.042497	14.34263	D
SSA_2320	9.364261	2.491409	4.810997	11.68385	6.52921	7.216495	0.601375	4.639175	3.694158	0.859107	5.756014	7.989691	0.515464	0	3.006873	4.381443	7.646048	6.185567	7.130584	5.498282	
SSA_2023	8.469751	3.41637	5.69395	7.188612	6.83274	5.338078	1.067616	3.41637	5.69395	1.209964	3.701068	8.113879	1.708185	0.284698	4.483986	3.772242	7.259786	6.192171	7.615658	8.540925	G
SSA_0829	7.569231	1.907692	1.169231	39.07692	1.846154	12.92308	0.184615	1.784615	1.169231	0.430769	1.046154	2.769231	0.184615	0	0.553846	0.984615	1.784615	3.261538	1.6	19.75385	
SSA_1882	8.366534	3.452855	4.648074	8.233732	7.702523	7.104914	0.929615	3.718459	4.316069	1.128818	4.183267	6.772908	0.464807	0	4.714475	3.187251	7.038513	5.843293	6.706507	11.48738	O
SSA_0860	9.502598	4.67706	8.166295	8.908686	5.567929	6.087602	0.519673	5.270973	6.087602	2.37565	3.043801	4.380104	1.781737	0.148478	4.528582	1.707498	9.651076	4.528582	6.161841	6.904232	M
SSA_2121	8.520179	2.114029	5.893658	6.470211	9.545163	10.05766	0.896861	4.740551	4.868674	0.320307	4.099936	5.188981	1.665599	0.064061	4.228059	2.306214	7.174888	6.534273	7.303011	8.007687	M
SSA_1663	9.199206	1.786896	5.294507	5.559232	9.596294	10.58901	0.463269	5.493051	2.581072	1.323627	3.309067	5.029782	0.860357	0.066181	2.97816	3.375248	9.463931	7.743216	7.875579	7.41231	M
SSA_2004	7.510504	3.728992	5.777311	6.670168	6.722689	6.302521	1.365546	3.939076	5.357143	1.207983	3.781513	9.138655	0.840336	0	4.411765	3.466387	7.720588	6.77521	6.197479	9.086134	S
SSA_1106	8.057631	3.148346	7.097118	7.257204	7.950907	5.976521	1.334045	5.229456	3.895411	1.227321	3.788687	8.271078	0.533618	0	5.229456	3.094984	6.616862	8.271078	5.65635	7.363927	S
SSA_0905	8.850458	3.153611	4.730417	5.84944	5.493388	12.76704	0.915565	4.069176	4.933876	0.813835	8.341811	3.814852	0.508647	0.050865	2.441506	3.560529	9.969481	5.239064	6.71414	7.782299	
SSA_1018	7.909419	4.627502	5.710535	6.202822	6.366918	8.598622	1.279947	4.890056	4.758779	0.78766	4.561864	7.8766	0.39383	0	4.824417	2.395799	5.644897	9.090909	6.005907	8.073515	S
SSA_0906	9.55414	2.472836	4.496066	6.144623	6.631697	14.31248	1.011615	3.596853	5.020607	0.524541	8.017984	3.709254	0.412139	0.037467	2.322967	3.934058	9.891345	5.395279	6.444361	6.069689	
SSA_0904	10.23411	2.64214	4.280936	4.749164	7.257525	13.64548	0.535117	3.511706	4.715719	0.568562	8.494983	3.377926	0.334448	0.033445	2.274247	4.381271	10.53512	5.719064	5.886288	6.822742	
